# Revealing Different Roles of the mTOR-Targets S6K1 and S6K2 in Breast Cancer by Expression Profiling and Structural Analysis

**DOI:** 10.1371/journal.pone.0145013

**Published:** 2015-12-23

**Authors:** Elin Karlsson, Ivana Magić, Josefine Bostner, Christine Dyrager, Fredrik Lysholm, Anna-Lotta Hallbeck, Olle Stål, Patrik Lundström

**Affiliations:** 1 Department of Clinical and Experimental Medicine, and Department of Oncology, Linköping University, SE-58185, Linköping, Sweden; 2 Division of Chemistry, Department of Physics, Chemistry and Biology, Linköping University, SE-58183, Linköping, Sweden; 3 Division of Bioinformatics and SeRC (Swedish e-Science Research Centre), Department of Physics, Chemistry and Biology, Linköping University, SE-581 83, Linköping, Sweden; University of Medicine, Greifswald, GERMANY

## Abstract

**Background:**

The AKT/mTORC1/S6K pathway is frequently overstimulated in breast cancer, constituting a promising therapeutic target. The benefit from mTOR inhibitors varies, likely as a consequence of tumour heterogeneity, and upregulation of several compensatory feed-back mechanisms. The mTORC1 downstream effectors S6K1, S6K2, and 4EBP1 are amplified and overexpressed in breast cancer, associated with a poor outcome and divergent endocrine treatment benefit. S6K1 and S6K2 share high sequence homology, but evidence of partly distinct biological functions is emerging. The aim of this work was to explore possible different roles and treatment target potentials of S6K1 and S6K2 in breast cancer.

**Materials and methods:**

Whole-genome expression profiles were compared for breast tumours expressing high levels of S6K1, S6K2 or 4EBP1, using public datasets, as well as after *in vitro* siRNA downregulation of S6K1 and/or S6K2 in ZR751 breast cancer cells. *In silico* homology modelling of the S6K2 kinase domain was used to evaluate its possible structural divergences to S6K1.

**Results:**

Genome expression profiles were highly different in S6K1 and S6K2 high tumours, whereas S6K2 and 4EBP1 profiles showed significant overlaps, both correlated to genes involved in cell cycle progression, among these the master regulator E2F1. S6K2 and 4EBP1 were inversely associated with IGF1 levels, and their prognostic value was shown to be restricted to tumours positive for IGFR and/or HER2. *In vitro*, S6K1 and S6K2 silencing resulted in upregulation of genes in the mTORC1 and mTORC2 complexes. Isoform-specific silencing also showed distinct patterns, e.g. S6K2 downregulation lead to upregulation of several cell cycle associated genes. Structural analyses of the S6K2 kinase domain showed unique structure patterns, deviating from those of S6K1, facilitating the development of isoform-specific inhibitors. Our data support emerging proposals of distinct biological features of S6K1 and S6K2, suggesting their importance as separate oncogenes and clinical markers, where specific targeting in different breast cancer subtypes could facilitate further individualised therapies.

## Introduction

The prognosis of breast cancer patients has been considerably improved in the latest 25 years, as a result of better diagnostics and treatment regimens. A future goal is a more individualised therapy, to further increase breast cancer survival, and also to decrease the risk of severe side-effects. For this purpose, additional tumour specific clinical markers and treatment targets are needed. The mammalian target of rapamycin (mTOR) is involved in many mechanisms of tumour progression [1]. mTOR exists in two cellular complexes, referred to as mTORC1 and mTORC2. Under normal circumstances, mTORC1 acts as a main signal integrator regulating cellular growth, homeostasis and metabolism. Less is known about mTORC2, which has been implicated in regulation of cytoskeletal dynamics, through activation of Rho GTPases and PKCα, and has also been revealed as the kinase responsible for phosphorylating AKT at Ser473, thereby promoting its activation [2].

Two major regulators of mTOR function, the RAS/MAPK and PI3K/AKT signalling pathways are constitutively activated in many cancers and are suggested as key drivers of breast tumour growth, interplaying with growth factor and steroid hormone signalling [1,3]. Deregulations in downstream mTOR-related pathways diminish the effects of common adjuvant breast cancer treatments [4]. Consequently, the mTOR/S6K/4EBP1 pathway has emerged as a new promising treatment target for several malignancies. The combination of mTOR inhibitors with endocrine therapy in second-line treatment of oestrogen receptor (ER) positive breast cancer has been shown successful [5], and this treatment regimen is now clinically approved.

Studies in recent years have indicated a clinical significance for alterations downstream of mTOR in malignancies. Well-known substrates of mTOR are the S6 kinases (S6K1 and S6K2) and the 4E binding protein 1 (4EBP1), which are mainly involved in the translational machinery, but have also been associated with transcriptional regulation [6]. We and others have shown that S6K1 and S6K2 gene amplification and overexpression may have prognostic and treatment predictive value in breast cancer [7–10]. The mTOR target 4EBP1 was initially considered a tumour suppressor gene, as a result of its role in negatively regulating the translational machinery through binding to EIF4E [11]. However, recent data has indicated that 4EBP1 may possess additional oncogenic roles under some circumstances. The chromosomal regions 11q13 and 8p12, harbouring the S6K2 and 4EBP1 genes are commonly co-amplified, and mRNA levels of S6K2 and 4EBP1 are highly correlated and associated with a poor prognosis, indicating that S6K2 and 4EBP1 may have synergistic tumourigenic effects [8,12]. Phosphorylation of the mTOR target 4EBP1 has been identified as a marker of poor prognosis in several malignancies, including breast cancer [8,11]. In addition, S6K2 and 4EBP1 have been implicated as markers of endocrine therapy resistance in breast cancer [8,10,13].

The physiological and cellular roles of S6K1 have been well investigated but less is known about S6K2. Recent studies have shown that S6K2 may have additional cellular functions, independent of those of S6K1 which may be of relevance for therapeutic purposes [14]. An arising problem when using mTOR antagonists is the phenomenon of counteracting feed-back mechanisms, where the most well-known involves S6K1, diminishing AKT signalling through inhibition of IRS1 and IRS2 [6]. On the contrary, data has suggested that S6K2 is not involved in the negative feedback loop, and can instead promote AKT signalling [15]. As a consequence, S6K2 could be a new, interesting target for this pathway in breast cancer.

S6K1 and S6K2 share high sequence homology, with approximately 80% identical residues in their kinase domains and were discovered as a result of their roles in regulating translation through phosphorylation of the ribosomal protein S6 [6,16–18]. Differences between S6K1 and S6K2 are mainly found in the regions N- and C-terminal of the kinase domain, which are probably important for localisation, regulation and function of the proteins. Whereas S6K1 contains a C-terminal PDZ binding domain [19], S6K2, harbours a C-terminal proline-rich domain allowing interactions with proteins containing SH3 or WW domains [20]. Both S6K1 and S6K2 exist in different isoforms as a result of alternative translational starting sites. For S6K1, p70 that is localized mainly in the cytoplasm is the predominant form. The p85 isoform, contains an additional 23 amino acid nuclear localisation sequence (NLS), targeting this isoform mainly to the nucleus [21]. The two isoforms of S6K2 are termed p54 and p56S6K2, where p54 is the predominant form. Both S6K2 isoforms contain a C-terminal NLS, and p56S6K2 also contains an N-terminal NLS [21], localising them mainly to the nucleus.

The aim of the present study was to investigate differences between the mTOR targets S6K1 and S6K2 and their individual potentials as new clinical targets in breast cancer, as well as further explore the importance of the S6K2/4EBP1 co-expression. Breast tumours expressing high levels of S6K1, S6K2 or 4EBP1 were first portrayed on a genome-wide scale in order to get further knowledge about the clinical feature of these tumours and to evaluate possible different roles between S6K1 and S6K2 in this context. Second, transcriptome analysis on a breast cancer cell line after knock-down of S6K1 and S6K2 individually or simultaneously was performed to evaluate different impact in global mRNA expression of S6K1 and S6K2. Finally, *in silico* three-dimensional structures of S6K2 were generated using homology modelling and the models were compared to the previously known crystal structures of S6K1 [22,23].

Comparisons of S6K1 and S6K2 revealed significant differences that could be of importance for divergences in regulation and function of the two kinases and also useful for future development of isoform-specific inhibitors.

## Methods and Materials

### S6K1, S6K2 and 4EBP1 global mRNA correlations in public datasets

To explore and compare the global expression profiles for tumours harbouring high levels of S6K1, S6K2 or 4EBP1 respectively, a public available dataset encompassing pre-processed mRNA expression data was downloaded for the van de Vijver cohort (n = 295) (http://bioinformatics.nki.nl/data.php). Student’s t-test was used to calculate the transcripts significantly differing between the cohorts of patients with highest compared to lowest quartile expression of S6K1, S6K2 or 4EBP1, respectively. No assumptions about the variances were made in the statistical test. The significance level was set to p<0.01/25000 = 4·10^−7^, as explained below.

For confirmation of findings, two further datasets, referred to as the Uppsala cohort (n = 236) (NCBI/GEO: GSE3494) and the Karolinska Institute cohort (n = 159) (NCBI/GEO: GSE1456) were used. The patient characteristics are shortly described in **S1 Table** and were previously presented in detail, as well as data processing [24–26]. For the Karolinska and Uppsala cohorts, in case of several array probes for each gene, a mean expression value was used.

### Cell culture and siRNA

To investigate global transcriptional regulation by S6K1 and S6K2 respectively, with focus on breast cancer, siRNA was used to knock-down S6K1, S6K2 or both kinases simultaneously in a human cell line, and expression arrays were used to screen for transcriptional alterations. The breast cancer cell line ZR751 (ATCC) was chosen since it has been shown to express high levels of both S6K1 and S6K2 (The cancer cell line encyclopaedia, CCLE, broadinstiture.org [27] and unpublished observations). The cells were cultured in phenol-red free Optimem (Gibco), supplemented with 4% Fetal bovine serum (Gibco) until reaching approximately 80% confluence and thereafter seeded into 15 cm^2^ flasks. The cells were nucleofected using the Amaxa cell line optimisation nucleofector kit, according to manufacturer’s instructions (Lonza). Optimal downregulation of S6K1 and S6K2 were reached 72 h after transfection with S6K1 siRNA 110802 and s12284 (Ambion, Life Technologies) and S6K2 siRNA 471 (Ambion, Life Technologies), respectively. As a control for transfection, scramble siRNA (Ambion, Life Technologies) was used. To confirm results from the array analysis, the experiment was repeated with ZR751 as well as the cell line BT474 (ATCC), also expressing high levels of S6K1 and S6K2, and expression levels of certain transcripts were analysed with real-time PCR.

### RNA preparation and Real-Time PCR

RNA was isolated with the mirVana^TM^ miRNA isolation kit (Ambion, Life Technologies) according to instructions provided by the manufacturer. Purified RNA was dissolved in nuclease-free water with addition of RNAsin Ribonuclease inhibitor (Promega) and stored at -70°C. RNA integrity numbers (RIN) and concentrations were assessed with an Agilent 2100 Bioanalyzer (Agilent Technologies). All samples RIN values reached ≥ 9. For confirming experiments with real-time PCR, RNA was prepared using the RNAqueous® Total RNA Isolation Kit (Ambion, Life Technologies).

Real-Time PCR was used to confirm downregulation of S6K1 and S6K2 as well as compensatory upregulation of RICTOR, RPTOR and PTEN. Reverse transcription was performed using the high-capacity cDNA reverse transcription kit (Applied Biosystems) with 200 ng total RNA in reactions of 20 μl according to manufacturer’s instructions. mRNA expression of S6K1 and S6K2 was quantified with fast real-time PCR using an ABI Prism 7900ht (Applied Biosystems). TaqMan assays (Applied Biosystems) for S6K1 (Hs00177357_m1), S6K2 (Hs00177689_m1), (RICTOR Hs00380903_m1), RPTOR (Hs00375332_m1), PTEN (Hs02621230_s1), and the endogenous control ACTB (part no 4310881E) were handled according to the manufacturer’s instructions. Quantitative PCR was performed in triplicates with 10 μl reaction volume, in 1× TaqMan fast universal master mix (Applied Biosystems) using the thermal conditions: 95°C for 20 s; 40 cycles of 95°C for 1 s, and 60°C for 20 s. To confirm specificity, reactions without reverse transcriptase (-RT), as well as no template controls (NTC) were included on each plate. Median value was taken from the triplicates and relative expression was calculated with the ΔΔCt method, using control siRNA as the calibrator.

### Affymetrix Genechip Gene 2.0

Affymetrix GeneChip Gene 2.0 expression arrays (Affymetrix) were used to profile alterations in global mRNA expression after silencing of S6K1, S6K2 or S6K1+S6K2 in triplicates. The assay was performed according to manufacturer’s protocols with 250 ng total RNA as starting material. Briefly, RNA was reverse transcribed into first and second strand cDNA, thereafter converted to cRNA and finally sscDNA, using Ambion, Life Technologies, WT Expression Kit (Ambion, Life Technologies). The sscDNA was fragmented and labelled with GeneChip WT Terminal labelling and hybridisation kit (Affymetrix), and hybridised to the arrays. Affymetrix Scanner 3000 7G was used to scan the arrays and generated data were converted to cell intensity files in Affymetrix Genotyping Console. Cell intensity data were processed by the software Genespring (Agilent Technologies). Raw data was normalised to the median of the control samples using the Robust Multi-array Average (RMA) summarisation algorithm [28]. Paired two-sided t-tests were used to estimate significant differences in gene expression after downregulation of S6K1 and/or S6K2 in comparison to scrambled siRNA. Only results from annotated genes were reported. As a significance limit p<0.05 was used.

### Homology modelling of the S6K2 kinase domain and structural comparison with S6K1

The 80% homology between S6K1 and S6K2 kinase domains, allowed us using *in silico* homology modelling [29] to predict the three-dimensional structure of the S6K2 kinase domain using the crystal structures of S6K1 as templates. Initially, the primary structures of S6K1 and S6K2 were compared using the basic local alignment search tool (BLAST, NCBI) and the NCBI protein data base was used to pinpoint the different domain structures on the aligned primary sequences. The three-dimensional structure of the S6K2 kinase domain was modelled according to standard procedures with the two different S6K1 crystal structures, (PDB ID: 3A62 and 4L3J) [22,23] as templates, using the software’s ICM (Molsoft) and Prime (Prime version 3.1, Schrödinger), respectively. Briefly, the sequences of the templates and S6K2 were initially aligned using Prime and the alignment was then refined using ClustalW. Then a secondary structure prediction was performed and subsequently the three-dimensional model was built using an energy-based algorithm. The last step was refinement of loops that lacked electron density in the template (one in this case). The ligands that were co-crystallised with S6K1 were removed prior to homology modelling. The proteins were visualised using PyMol (Schrödinger) and the pairwise root-mean-square-deviation (RMSD) between the structures of the template (S6K1) and the model (S6K2) was calculated using the SuperPose web server [30].

### Statistics and bioinformatics

The public web server g:Profiler [31,32] was used to predict pathways and cellular functions enriched in the array data from van de Vijver comparisons as well as siRNA knock-down. Hierarchical filtering on best parent group was used and only significant enriched profiles were shown.

Associations between different variables were assessed by Spearman’s rank order correlation where mentioned. In the survival analyses, the variable S6K2 and/or 4EBP1 was defined as highest quartile expression of at least one of the genes, as previously described [8]. HER2 positivity was set to the group with the highest 15% expression levels, whereas high IGF1R or IGF2R was defined as highest quartile expression of the genes, respectively. The Kaplan-Meier product limit method was used to estimate the cumulative probabilities of breast cancer specific survival (BCS), and differences between the curves were evaluated with the Log-rank test. For multivariate analysis of event rates, Cox proportional hazard regression was used. Survival analyses were performed with Statistica 12 (Statsoft). Differences between gene expression levels in the real-time PCR experiments were visualised and analysed with GraphPad prism (GraphPad Software, Inc, La Jolla, CA, USA), using one-sided, paired t-tests. In all analyses, p<0.05 was considered statistically significant if nothing else is specified.

## Results

### Expression profiles of S6K1 or S6K2 high tumours reveal few genes strongly correlated to both S6K1 and S6K2

We have previously shown that high mRNA levels of S6K2 and/or 4EBP1 are associated with a poor outcome in breast cancer, independent of other clinicopathological factors, whereas this was less prominent for high S6K1 levels [8]. Here, we investigated the whole-genome expression profiles of S6K1, S6K2 and 4EBP1 high tumours to get further knowledge of the molecular biological features of these tumours, using the publically available van de Vijver dataset.

In total, in the van de Vijver cohort, expression values for 24491 different transcripts are available. To minimise false correlations, the significance level was set to p<4⋅10^−7^. The rational for this is a significance level of p<0.01 for each gene compensated by the presence of ≈25000 t-tests. Using this threshold, S6K1 mRNA levels were significantly correlated to 616 other transcripts (497 positively, 119 inversely, **S2 Table**). S6K2 was significantly associated with 212 transcripts (144 positively, 68 inversely, **S3 Table**), and 4EBP1 with 965 transcripts (538 positively, 427 inversely, **S4 Table**).

S6K1 mRNA was positively associated with RPS6 and PIK3CA mRNA in the PI3K/AKT/mTOR pathway **(S2 Table)**, whereas no positive correlations to S6K2 for these genes were found (p = 0.41 and inverse correlation p = 0.026). S6K1 positively associated genes included genes involved in cell cycle/mitosis, e.g. Cyclin E and the centromere proteins CENPC1, CENPE, CENPF, intracellular transport, RNA binding, intracellular part/cytoplasmic proteins as well as BAZ1A and SMARCA5 in the WCRF complex, essential for chromatin remodelling (**S2 Table)**. Positive correlations were also found between S6K1 and mRNA levels for DNA topoisomerase 2-beta (TOP2B), RAD51 homolog C (RAD51C), breast carcinoma amplified sequence 1 (BCAS1, located at 20q13), and borderline with amplified in breast cancer 1 (AIB1/NCOA3, located at 20q12) (p = 6.72·10–7). S6K1 was negatively associated with among others the p90 ribosomal S6 kinase RPS6KA4 and the transcription factor STAT5A.

Only two genes, SWI/SNF related, matrix associated, actin dependent regulator of chromatin (SMARCD1), and DDB1 and CUL4 associated factor 7 (Han11) were significantly associated with both S6K1 and S6K2. Similarly, only two genes were shared between the S6K1 and 4EBP1 expression profiles, Histone H2A.Z (H2AFZ) and Mitotic spindle assembly checkpoint protein MAD2A (MAD2L1) were positively correlated to both S6K1 and 4EBP1. Twenty-three transcripts were positively correlated to S6K1, but inversely associated with 4EBP1 expression, whereas two genes were positively associated with 4EBP1, but inversely correlated to to S6K1 (**S5 Table**).

### S6K2 and 4EBP1 high tumours have overlapping expression profiles, enriched for cell cycle associated genes and E2F1 transcription regulated genes

**Fig 1:**
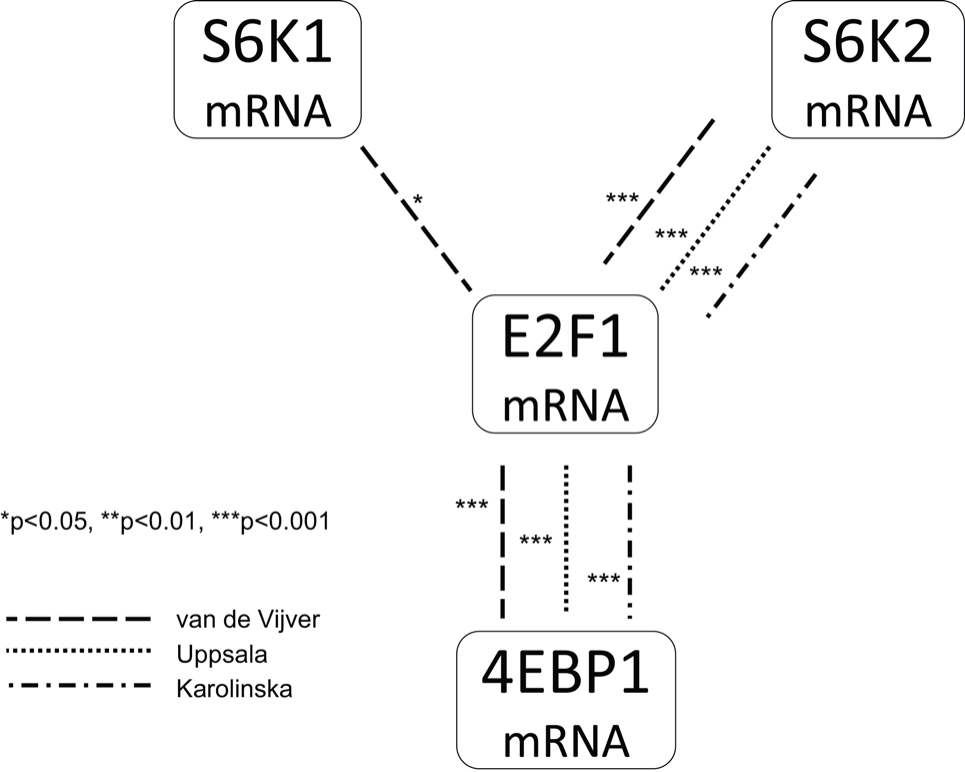
Spearman’s rank order correlation evaluating associations between S6K1, S6K2, 4EBP1 and E2F1 mRNA expression (continuous values) in three breast cancer cohorts.

Earlier studies have shown a co-amplification and co-expression of S6K2 and 4EBP1 [8,12]. Accordingly, the expression profiles for S6K2 and 4EBP1 high tumours were highly overlapping, with 120 common genes, whereof 75 transcripts positively correlated and 45 transcripts inversely correlated to both S6K2 and 4EBP1 **(Tables 1–3)**. The probability that this high degree of overlap is due to chance is virtually zero as gauged by a run test for randomness of two related samples. Positively associated genes included several genes involved in cell cycle progression; among others CCNB1, CCNB2, CENPA, CDC20 and CDC25B, oocyte maturation and meiosis, metabolism; including pituitary tumour-transforming (PTTG1, PTTG2 and PTTG3) and mevalonate (diphospho) decarboxylase (MVD) involved in cholesterol synthesis, as well as the cell-cycle regulating transcription factor E2F1 and several of its targets. The strong correlation to E2F1 was confirmed in two additional data sets **(Fig 1)**. S6K2 and 4EBP1 were both inversely associated with IGF1 mRNA expression which is further evaluated in later paragraphs.

**Table 1:** Genes significantly positively correlated to both S6K2 and 4EBP1 and comparisons with correlations to S6K1^1^.

Gene	S6K2 t-statistic	S6K2 p-value	4EBP1 t-statistic	4EBP1 p-value	S6K1 t-statistic	S6K1 p-value
NM_000485__APRT	9.22	2.97E-16	6.99	9.07E-11	0.76	0.4487
NM_002461__MVD	8.67	7.23E-15	5.42	2.40E-07	-0.28	0.7832
NM_002466__MYBL2	7.29	1.72E-11	6.98	9.26E-11	0.71	0.4792
M96577__E2F1	7.24	2.37E-11	8.01	3.18E-13	2.03	0.0445
NM_014501__E2-EPF	7.07	5.92E-11	7.85	8.16E-13	1.97	0.0506
NM_002708__PPP1CA	6.82	2.25E-10	7.11	4.76E-11	0.42	0.6787
NM_003258__TK1	6.73	3.45E-10	9.42	8.70E-17	2.70	0.0078
NM_004219__PTTG1	6.68	4.53E-10	6.78	2.78E-10	1.92	0.0568
NM_003093__SNRPC	6.60	6.92E-10	5.88	2.71E-08	1.79	0.0748
NM_006819__STIP1	6.60	7.03E-10	6.51	1.13E-09	0.99	0.3227
NM_003158__STK6	6.46	1.47E-09	6.96	1.05E-10	2.90	0.0043
NM_002720__PPP4C	6.30	3.30E-09	5.84	3.15E-08	-0.43	0.6660
NM_001034__RRM2	6.26	4.01E-09	7.85	8.08E-13	1.48	0.1404
NM_003548__H4F2	6.23	4.54E-09	6.09	9.37E-09	3.13	0.0021
NM_005412__SHMT2	6.22	4.97E-09	9.08	6.48E-16	-1.46	0.1470
NM_004701__CCNB2	6.18	5.90E-09	8.72	5.37E-15	2.35	0.0199
NM_018683__ZNF313	6.17	6.39E-09	7.90	5.93E-13	0.14	0.8918
NM_020187__DC12	6.12	8.15E-09	6.19	5.80E-09	-2.13	0.0346
NM_001916__CYC1	5.92	2.20E-08	6.58	7.61E-10	0.10	0.9188
NM_004111__FEN1	5.89	2.49E-08	7.30	1.65E-11	2.85	0.0050
NM_003600__STK15	5.89	2.57E-08	7.64	2.62E-12	2.50	0.0135
NM_004217__STK12	5.88	2.68E-08	7.75	1.36E-12	0.71	0.4778
M55914__MPB1	5.86	2.96E-08	6.99	8.97E-11	-4.24	<0.0001
NM_003765__STX10	5.83	3.31E-08	5.59	1.09E-07	-3.37	0.0010
NM_003504__CDC45L	5.83	3.44E-08	7.75	1.40E-12	1.71	0.0886
NM_004461__FARSL	5.81	3.71E-08	5.58	1.13E-07	-3.46	0.0007
NM_001168__BIRC5	5.81	3.82E-08	8.82	3.00E-15	2.56	0.0116
NM_020675__AD024	5.80	3.88E-08	7.28	1.91E-11	4.36	<0.0001
NM_006607__PTTG2	5.80	3.99E-08	5.64	8.49E-08	2.73	0.0071
NM_003544__H4FI	5.80	4.00E-08	5.49	1.69E-07	1.30	0.1969
NM_004553__NDUFS6	5.79	4.05E-08	6.09	9.49E-09	1.69	0.0931
NM_002707__PPM1G	5.78	4.25E-08	7.80	1.03E-12	-2.01	0.0466
Contig56843_RC__CCNB1	5.76	4.76E-08	6.10	8.81E-09	1.65	0.1021
NM_007103__NDUFV1	5.72	5.70E-08	5.82	3.55E-08	-3.30	0.0012
NM_002131__HMGIY	5.72	5.74E-08	5.75	5.09E-08	-0.59	0.5567
NM_007274__HBACH	5.69	6.53E-08	8.14	1.57E-13	-0.24	0.8106
NM_014272__ADAMTS7	5.67	7.19E-08	6.22	4.88E-09	-2.76	0.0064
NM_001809__CENPA	5.64	8.53E-08	7.99	3.70E-13	2.72	0.0073
NM_005945__MPB1	5.61	9.88E-08	7.05	6.62E-11	-4.54	<0.0001
U96131__TRIP13	5.61	9.97E-08	10.86	1.53E-20	0.47	0.6418
NM_005192__CDKN3	5.58	1.14E-07	5.94	1.95E-08	3.19	0.0018
NM_004203__PKMYT1	5.57	1.20E-07	7.68	2.01E-12	1.45	0.1499
NM_018455__BM039	5.53	1.40E-07	6.74	3.39E-10	1.62	0.1066
NM_001255__CDC20	5.52	1.53E-07	8.25	8.23E-14	-0.75	0.4542
NM_021000__PTTG3	5.49	1.75E-07	6.36	2.40E-09	1.08	0.2802
NM_004358__CDC25B	5.48	1.80E-07	6.51	1.09E-09	0.68	0.4973
NM_001428__ENO1	5.47	1.87E-07	5.99	1.54E-08	-4.71	<0.0001
NM_004596__SNRPA	5.44	2.16E-07	7.43	7.99E-12	-3.45	0.0007
NM_002875__RAD51	5.44	2.16E-07	7.73	1.56E-12	1.77	0.0786
D14678__KNSL2	5.42	2.37E-07	6.35	2.55E-09	0.42	0.6772
NM_005371__METTL1	5.42	2.41E-07	6.73	3.48E-10	0.50	0.6207
NM_005480__TROAP	5.41	2.52E-07	5.48	1.83E-07	1.50	0.1368
NM_003491__ARD1	5.41	2.55E-07	6.90	1.43E-10	-0.97	0.3325
NM_005796__PP15	5.38	2.92E-07	8.12	1.72E-13	-0.39	0.7003
NM_002904__RDBP	5.37	3.06E-07	7.53	4.69E-12	0.04	0.9653
NM_013299__HSU79266	5.35	3.37E-07	6.53	1.02E-09	-0.11	0.9130
NM_014176__HSPC150	5.32	3.72E-07	5.45	2.12E-07	3.87	0.0002
NM_000291__PGK1	5.31	3.92E-07	8.76	4.24E-15	0.28	0.7761

^1^Only annotated genes are shown.

**Table 2:** Genes significantly inversely correlated to both S6K2 and 4EBP1 and comparisons with correlations to S6K1^1^.

Gene	S6K2 t-statistic	S6K2 p-value	4EBP1 t-statistic	4EBP1 p-value	S6K1 t-statistic	S6K1 p-value
X57025__IGF1	-7.61	3.01E-12	-5.37	3.01E-07	0.27	0.7882
NM_013231__FLRT2	-7.09	5.33E-11	-6.37	2.24E-09	-3.44	0.0008
NM_005780__LHFP	-6.62	6.40E-10	-6.70	4.14E-10	0.25	0.8020
NM_004684__SPARCL1	-6.48	1.33E-09	-7.98	3.72E-13	1.51	0.1323
NM_001920__DCN	-6.45	1.56E-09	-6.72	3.73E-10	-2.08	0.0388
NM_000313__PROS1	-6.14	7.26E-09	-5.98	1.62E-08	-0.15	0.8820
D50406__RECK	-6.03	1.29E-08	-5.57	1.15E-07	-2.16	0.0325
NM_003014__SFRP4	-6.01	1.40E-08	-5.94	1.97E-08	-2.36	0.0200
NM_004538__NAP1L3	-5.99	1.56E-08	-5.53	1.42E-07	-0.62	0.5389
NM_004787__SLIT2	-5.76	4.74E-08	-6.42	1.81E-09	-2.71	0.0074
NM_012429__SEC14L2	-5.74	5.21E-08	-5.47	1.87E-07	-3.38	0.0009
NM_001393__ECM2	-5.71	6.17E-08	-6.67	4.92E-10	-0.44	0.6618

^1^Only annotated genes are shown.

**Table 3:** Pathways enriched among genes related to S6K2 and 4EBP1.

S6K2 and 4EBP1 positive correlations pathways			
p-value	Term	Term description	Genes
4.82e-02	GO:0048609	multicellular organismal reproductive process	*APRT*, *CCNB1*, *CDC25B*, *E2F1*, *PTTG1*, *TK1*, *TRIP13*, *ZNF313*
9.39e-08	GO:0022402	cell cycle process	*BIRC5*, *CCNB1*, *CCNB2*, *CDC20*, *CDC25B*, *CDKN3*, *CENPA*, *E2F1*, *FEN1*, *MYBL2*, *PKMYT1*, *PPM1G*, *PTTG1*, *RAD51*, *RRM2*, *TRIP13*
4.86e-02	GO:0016043	cellular component organization	*BIRC5*, *CCNB1*, *CCNB2*, *CDC20*, *CDC25B*, *CENPA*, *ENO1*, *FEN1*, *MYBL2*, *NDUFS6*, *PKMYT1*, *PPP4C*, *PTTG1*, *PTTG2*, *RAD51*, *RRM2*, *SHMT2*, *SNRPC*, *TK1*, *TRIP13*
5.34e-03	GO:0001556	oocyte maturation	*CCNB1*, *CDC25B*, *TRIP13*
2.99e-02	GO:0007144	female meiosis I	*CDC25B*, *TRIP13*
4.18e-02	GO:0044237	cellular metabolic process	*APRT*, *BIRC5*, *CCNB1*, *CCNB2*, *CDC20*, *CDC25B*, *CDKN3*, *CENPA*, *CYC1*, *E2F1*, *ENO1*, *FEN1*, *METTL1*, *MVD*, *MYBL2*, *NDUFS6*, *NDUFV1*, *PKMYT1*, *PPM1G*, *PPP4C*, *PTTG1*, *PTTG2*, *RAD51*, *RRM2*, *SHMT2*, *SNRPA*, *SNRPC*, *TK1*, *TRIP13*
1.37e-02	GO:0006259	DNA metabolic process	*CCNB1*, *CENPA*, *FEN1*, *PPP4C*, *PTTG1*, *PTTG2*, *RAD51*, *RRM2*, *TK1*, *TRIP13*
1.29e-02	GO:0005488	binding	*ADAMTS7*, *APRT*, *BIRC5*, *CCNB1*, *CCNB2*, *CDC20*, *CDC25B*, *CDKN3*, *CENPA*, *CYC1*, *E2F1*, *ENO1*, *FEN1*, *METTL1*, *MVD*, *MYBL2*, *NDUFV1*, *NGB*, *PKMYT1*, *PPM1G*, *PPP4C*, *PTTG1*, *PTTG2*, *RAD51*, *RRM2*, *SHMT2*, *SNRPA*, *SNRPC*, *STIP1*, *STX10*, *TK1*, *TRIP13*, *TROAP*, *ZNF313*
7.43e-07	BIOGRID	BioGRID interaction data	*APRT*, *BIRC5*, *CCNB1*, *CCNB2*, *CDC20*, *CDC25B*, *CDKN3*, *CENPA*, *CYC1*, *E2F1*, *ENO1*, *FEN1*, *METTL1*, *MVD*
2.81e-07	KEGG:04110	cell cycle	*CCNB1*, *CCNB2*, *CDC20*, *CDC25B*, *E2F1*, *PKMYT1*, *PTTG1*, *PTTG2*
8.36e-05	KEGG:04114	oocyte meiosis	*CCNB1*, *CCNB2*, *CDC20*, *PKMYT1*, *PTTG1*, *PTTG2*
1.08e-02	KEGG:04914	progesterone-mediated oocyte maturation	*CCNB1*, *CCNB2*, *CDC25B*, *PKMYT1*
2.83e-04	REAC:69273	cyclin A/B1 associated events during G2/M transition	*CCNB1*, *CCNB2*, *CDC25B*, *PKMYT1*
9.84e-05	REAC:69278	cell cycle, mitotic	*BIRC5*, *CCNB1*, *CCNB2*, *CDC20*, *CDC25B*, *CENPA*, *E2F1*, *FEN1*, *PKMYT1*, *PTTG1*, *RRM2*
1.60e-02	TF:M00428_3	Factor: E2F-1; motif: NKTSSCGC; match class: 3	*APRT*, *CCNB1*, *CDC20*, *CDC25B*, *CENPA*, *CYC1*, *E2F1*, *ENO1*, *FEN1*, *METTL1*, *MVD*, *MYBL2*, *NDUFS6*, *NDUFV1*, *PKMYT1*, *PPM1G*, *PPP4C*, *RRM2*, *SHMT2*, *SNRPA*, *SNRPC*, *STIP1*, *TK1*, *TRIP13*, *TROAP*, *ZNF313*
**S6K2 and 4EBP1 inverse correlations pathways**			
**p-value**	**Term**	**Term description**	**Genes**
3.26e-03	GO:0044421	extracellular region part	*DCN*, *ECM2*, *FLRT2*, *IGF1*, *SFRP4*, *SLIT2*, *SPARCL1*
5.00e-02	REAC:159729	pro-protein S is transported from the endoplasmic reticulum to the Golgi apparatus	*PROS1*
2.67e-02	REAC:114611	exocytosis of Alpha granule	*IGF1*, *PROS1*

Factors positively correlated to S6K2, but not 4EBP1 included oncostatin M (OSM) and factors regulated by miR715 and miR702. S6K2 was furthermore inversely associated to caveolin1 (CAV1), an activator of RAS/MAPK signalling **(S6 Table)**.

### 4EBP1 is inversely correlated to ESR1 mRNA expression

4EBP1 mRNA levels were highly variable between tumours and were therefore significantly associated with several transcripts. Genes positively correlated to 4EBP1 only, included further cell cycle/mitosis and check-point associated genes, among others CCNA2, CHEK1 and mini-chromosome-maintains (MCM) proteins MCM2, MCM5, MCM6 and MCM7 included in the replication initiation complex. 4EBP1 was also positively associated with genes coupled to diabetes pathways, genes regulated by the transcription factors E2F, USF and SP1, the oncogenes HRAS and HOXB13 and several genes involved in negative regulation of translational initiation (EIF4EBP2, EIF2B3, EIF2C2) **(S7 Table)**.

4EBP1 mRNA expression was inversely correlated to genes involved in extra-cellular-matrix organisation and cell adhesion, including the laminins LAMA2 and LAMC1, collagen (COLA5) and thrombospondin-4 (THBS4). Enriched genes also included factors involved in cancer pathways and PI3K/AKT signalling, including IRS1 **(S2 Table)**, BCL2, TGFB3 and FGF18 **(S7 Table)**. One of the top-genes inversely correlated to 4EBP1 was ESR1, encoding ERα. High expression of 4EBP1 at the protein level has previously been shown as a marker of less benefit from ER-targeted therapies [8,13]. We therefore speculated that 4EBP1 upregulation may be one mechanism behind ER downregulation and endocrine resistance in breast cancer. In the next step, we searched for differences in the 4EBP1 expression profiles between ER positive and negative tumours. As a result of a lower number of patients in the ER negative subcohort, few genes reached the significance level (data not shown), however one of the genes strongest correlated to 4EBP1 in the ER negative group, but not the ER positive group was ESRRA (p = 2.07⋅10^−5^). When investigating the correlations to cell cycle associated genes, this was most prominent in the ER positive subgroup (data not shown).

### S6K2 and 4EBP1 are inversely correlated to IGF1 and IGF2 and are associated with poor prognosis in IGFR and/or HER2 positive tumours

**Fig 2:**
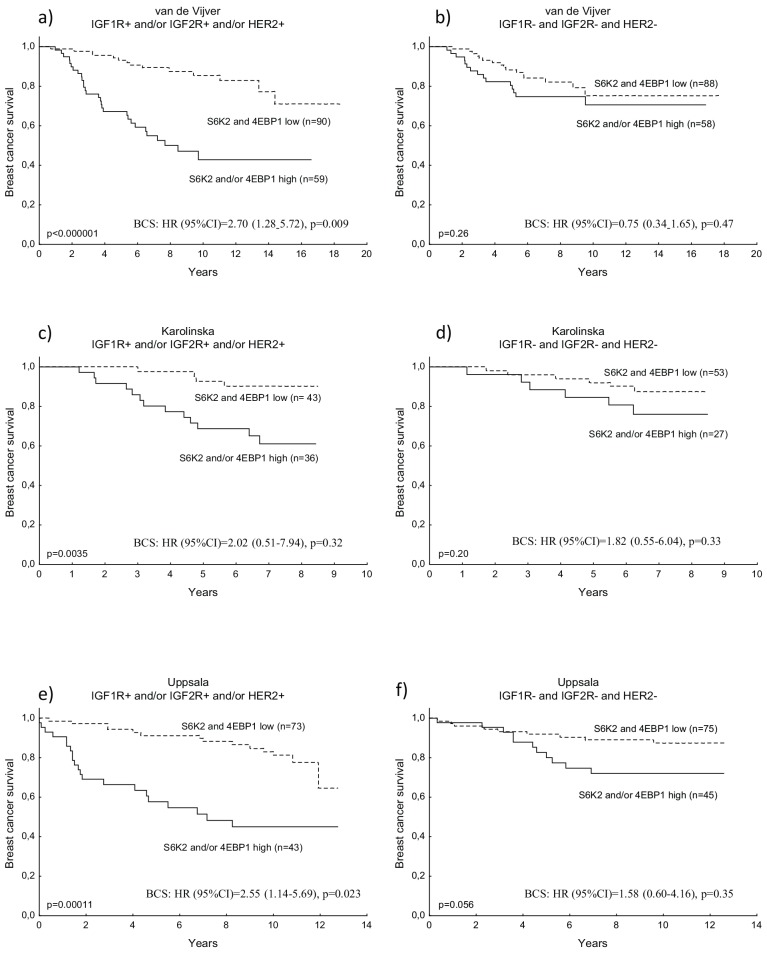
Kaplan-Meier curves and multivariate Cox regression of breast cancer survival (BCS) in relation to S6K2 and/or 4EBP1 mRNA, in the van de Vijver, Karolinska and Uppsala patient cohorts respectively. IGF1R and/or IGF2R and/or HER2 high **(a, c, e)**; IGF1R and IGF2R and HER2 low **(b, d, f)**. The Cox analysis included the following variables: adjuvant chemotherapy treatment, endocrine treatment, lymph node status, tumour size (not available for van de Vijver), ER status and E2F1.

One of the main genes inversely correlated to S6K2 and 4EBP1 mRNA expression was IGF1. Therefore, the associations of further members of the IGFR pathway with S6K1, S6K2 and 4EBP1 were evaluated in the van de Vijver cohort as well as the Karolinska and Uppsala cohorts. IGF1 was confirmed strongly inversely associated with both S6K2 and 4EBP1 in the three datasets **(Table 4)**. This could also be seen for IGF2, as well as IGF1R. The IGF pathway is a main activator of AKT/mTOR signalling and autocrine stimulation of IGF is common in transformed cells [33]. The inverse correlation between IGF1 and S6K2/4EBP1 may reflect an alternative mechanism in driving malignancy. High expression of S6K2 and/or 4EBP1 has earlier been shown predictive of a poor outcome in breast cancer [8]. Here, we show that this is restricted to tumours expressing high levels of IGF1R, IGF2R or HER2, whereas no prognostic significance could be seen in the IGFR/HER2 negative group **(Fig 2)**. For S6K1, there was no prognostic value in these subgroups (data not shown). The prognostic value of S6K2 and/or 4EBP1 in IGFR/HER2 positive tumours could be confirmed in two additional public datasets, and was also independent of the strong proliferation marker E2F1 **(Fig 2)**.

**Table 4:** Spearman’s rank order correlation evaluating associations between S6K1, S6K2, 4EBP1 and mRNA expression of factors in the IGFR signalling pathway (continuous values) in three breast cancer cohorts^1^,^2^.

		IRS1	IRS2	IRS4	IGF1	IGF2	IGFR1	IGFR2	INSR
**S6K1**	*van de Vijver*	-0.09	-0.05	0.09	0.02	**-0.23**	0.02	-0.08	0.03
	*Karolinska*	0.12	-0.11	**-0.21**	-0.13	-0.18	0.05	0.08	0.01
	*Uppsala*	-0.05	-0.10	-0.10	-0.13	-0.14	0.03	***0.25***	-0.04
**S6K2**	*van de Vijver*	**-0.20**	***0.22***	0.10	**-0.43**	**-0.28**	-0.06	0.02	0.14
	*Karolinska*	-0.12	-0.16	0.13	**-0.30**	**-0.29**	**-0.23**	0.18	-0.04
	*Uppsala*	**-0.31**	**-0.19**	0.03	**-0.27**	**-0.24**	**-0.22**	-0.06	**-0.20**
**4EBP1**	*van de Vijver*	**-0.32**	0.08	0.14	**-0.36**	**-0.27**	**-0.18**	0.08	0.03
	*Karolinska*	**-0.25**	**-0.36**	0.00	**-0.49**	**-0.46**	**-0.30**	***0.24***	-0.11
	*Uppsala*	**-0.19**	-0.14	-0.03	**-0.48**	**-0.43**	**-0.26**	-0.06	-0.14

^1^Spearman’s rank order correlation for p<0.01 are shown in bold font.

^2^Positive correlations are italicized.

### S6K1 and S6K2 silencing leads to a compensatory transcriptional upregulation of mTORC1, as well as mTORC2

Global expression profiling revealed large divergence between S6K1 and S6K2 positive tumours, suggesting different roles in carcinogenesis. Consequently, targeting the two S6 kinase isoforms respectively, may lead to different clinical responses, which also could be tumour subtype specific. In a next step, S6K1 and S6K2 were individually or simultaneously silenced in the breast cancer cell line ZR751, expressing high levels of both S6K1 and S6K2, and possible different effects on the transcriptome were investigated. Due to few replicates (n = 3) a significance level of p<0.05 was used. S6K1 and S6K2 were successfully silenced by approximately 80% as could be confirmed by Real-Time PCR **(S1 Fig)**. Also the protein expression levels were significantly reduced (data not shown).

S6K1 and S6K2 are highly homologous and expected to have both overlapping and separate cellular functions. Real-Time PCR data showed significant upregulation of S6K2 after S6K1 silencing **(S1 Fig)**. The opposite compensatory mechanism could be seen with microarray data where S6K1 was upregulated after S6K2 knock-down **(Table 5)**.

**Table 5:** Factors in the mTOR pathway significantly altered after S6K1 and/or S6K2 downregulation in ZR751 cells according to expression arrays.

Gene	S6K1 siRNA	S6K2siRNA	S6K1+S6K2siRNA
*EIF2AK2*		up	up
*EIF4B*			up
*EIF4E*			up
*EIF4EBP1*	down		
*EIF4G2*	down		down
*EIF5A2*			up
*EIF5B*		up	
*EIF6*			down
*ERBB3*		up	
*IGF2R*		up	
*MAPKAP1*			up
*MTOR*			up
*NGDN* ^1^			up
*PIK3R3*		up	
*PTEN*		up	up
*RICTOR*			up
*RPS6KA3*			up
*RPS6KB1*	down	up	down
*RPS6KB2*	up	down	down
*RPS6KC1*			up
*RPTOR*	up	up	

^1^Neurigudin, an EIF4E binding protein.

In a first analysis, genes commonly altered by silencing of S6K1 as well as of S6K2 were evaluated **(Table A in S8 Table)**. Genes upregulated with both S6K1 and S6K2 knock-down included the mTORC1 factor Raptor, (which also reached borderline significance for double knock-down, p = 0.051) **(Table 5)**, as well as the adhesion molecules CDH1, TLN1 (talin1) and CADM4 and the cell cycle associated genes RAD51C and TOP2B. The gene BCAS1 was significantly downregulated by both S6K1 and S6K2 siRNA, respectively, and this was also borderline significant with simultaneous S6K1 and S6K2 silencing (p = 0.075). As mentioned in a previous section, RAD51C, TOP2B and BCAS1 were all positively correlated to S6K1 in the van de Vijver cohort. Also, the translational factor EIF3G was significantly upregulated after S6K1 or S6K2 knock-down and a similar tendency could be seen with double siRNA. Upregulated genes included factors involved in cellular macromolecular metabolic process, nuclear lumen and targets of miR140. The oncostatin M receptor (OSMR) was significantly upregulated by S6K1 silencing and reached borderline significance with S6K2 knock-down (p = 0.07).

Since S6K1 and S6K2 are assumed to have some compensatory mechanisms, silencing of both isoforms may have other cellular effects than single down-regulation. Genes and pathways altered after double, but not single knock-down are presented in **S9 Table**. These include the mTORC2 factors; mTOR, Rictor (reaching borderline significance for single knock-down, S6K1 p = 0.06, S6K2 p = 0.08) and MAPKAP1 **(Table 5)**, genes involved in the translational machinery (RPS6KA3, RPS6KC1, EIF4E, EIF4B, EIF5A2, EIF6 (downregulated), the protein tyrosine phosphatase (PTP) family genes PTPN2 and PTPN21, the Myc-associated factor MAX, JAK1, genes in the MAPK pathway and EFNB2 (ephrin B2).

### Silencing of S6K1 and S6K2 show differences in expression profiles

Several transcripts were altered in response to S6K1 or S6K2 silencing exclusively. Genes upregulated by S6K1siRNA, but not S6K2siRNA, included EIF4E2 **(Table 5),** Cyclin D1 (CCND1), the Fibroblast growth factor receptor 1 (FGFR1) and Insulin-related receptor 1 (INSRR), the mitogen activated kinases MAP3K4 and MAPK3. Downregulated genes after S6K1 knock-down included EIF4EBP1 and EIF4G2 **(Table 5),** as well as the adaptor GAB3 **(S10 Table).** Enriched upregulated genes were coupled to membrane-bounded organelle, targets of miR144 and the transcription factors USF and CDPCR1.

S6K2 downregulation resulted in upregulation of genes enriched in several cellular processes **(S11 Table)**. These represented cell cycle/mitosis; among others CCNE1, CCNE2, CCNG2, CCNL1, CCNT1, CDK12 and CDKN3, prostate cancer; including the androgen receptor (AR), metabolism as well as targets of the transcription factors E2F, AP2, SP1, ETF and Pax3. Other upregulated genes upon S6K2 knock-down included the PI3K/AKT associated genes IGF2R, PTEN and PIK3R3, EIF2AK2 and EIF5B **(Table 5)**, the oncogenes ABL1 and MDM2, and the tumour suppressor candidates DGDHS, TSSC1 and TUSC3. Downregulated genes after S6K2siRNA included genes in ion-and monoamine transport as well as ClassA1 Rhodopsin-like receptors **(S11 Table)**. Downregulated genes of note included ABL2 and PTPN22. Eight genes were downregulated after S6K1 silencing, but upregulated with S6K2siRNA **(Table 6)**.

**Table 6:** Genes that are significantly (p<0.05) downregulated by S6K1 siRNA, and upregulated by S6K2 siRNA.

Transcripts Cluster Id	Gene description	Gene symbol	S6K1 p-value	S6K2 p-value
16944724	disrupted in renal carcinoma 2	*DIRC2*	0.0434	0.0429
16761830	endoplasmic reticulum protein 27	*ERP27*	0.0053	0.0362
16696979	glutamate-ammonia ligase	*GLUL*	0.0337	0.0215
16851427	Cdk5 and Abl enzyme substrate 1	*CABLES1*	0.0231	0.0028
17117692	proteasome (prosome, macropain) 26S subunit, non-ATPase, 7	*PSMD7*	0.0480	0.0162
16836626	ribosomal protein S6 kinase, 70kDa	*RPS6KB1*	0.0000	0.0449
16967863	amphiregulin	*AREG*	0.0163	0.0239
16904193	integrin, beta 6	*ITGB6*	0.0101	0.0401

The compensatory upregulation of S6K1, S6K2, RPTOR, RICTOR and PTEN after S6 kinase knock-down was further analysed by real-time PCR in the cell lines ZR751 and BT474. After S6K1 knock-down, S6K2 was significantly upregulated in both cell lines. In ZR751, RPTOR and RICTOR was significantly upregulated after S6K2 silencing and a similar tendency could be seen after S6K1 siRNA. In BT474, PTEN was significantly upregulated after S6K2 silencing **(S1 Fig)**.

### Sequence alignment and homology modelling of the S6K2 kinase domain reveal differences from S6K1, facilitating design of isoform specific inhibitors

Whole-genome profiling of S6K1 and S6K2 high tumours, as well as after *in vitro* knock-down of the two kinases both resulted in significant differences, suggesting that isoform specific targeting of S6K1 or S6K2 may be valuable to allow further individualised breast cancer treatment regimens. In 2010, the first crystal structure of the S6K1 kinase domain was solved [22], promoting design of an inhibitor specific for this isoform [34]. Recently, the crystal structure of S6K1 bound with its specific inhibitor was reported [23]. However, the three-dimensional structure of S6K2 is still unknown and no inhibitors specific for S6K2 are yet available [14]. Consequently, we developed *in silico* structures of the S6K2 kinase domain using homology modelling with the S6K1 crystal structures as templates.

To evaluate structural similarities and differences between S6K1 and S6K2, the primary structures were initially aligned using BLAST. S6K1 and S6K2 are both composed of 15 exons, where 1–14 are homologous, resulting from gene duplication. In contrast, exon 15 is different between the two kinases; S6K1 has a C-terminal PDZ-binding motif whereas S6K2 contains a proline-rich domain that likely interacts with SH3 and/or WW domains **(S2 Fig)**. The amino acid sequences in the kinase domains of S6K1 and S6K2 are highly similar with 80% identical amino acids. The residues in the active site, the substrate binding site and a hydrophobic motif, C-terminal of the proper kinase domain, are identical in the two kinases **(S3 Fig)**. The ATP binding site however contains one amino acid substitution. Whereas the amino acid at position 151 of S6K1 is tyrosine, the amino acid at the corresponding position of S6K2 is cysteine **(S3 Fig and S4 Fig)**. To our knowledge S6K2 and the 35% homologous SGK494 are the only member of the AGC family of kinases to have a non-aromatic residue at this site, (data not shown), facilitating development of S6K2-specific inhibitors. Two interesting amino acid substitutions are also present in the activation loop. D223 and T225 for S6K1 are substituted for glutamic acid and alanine in S6K2, possibly allowing different modes of kinase activation **(S3 Fig and S4 Fig)**.

The S6K1 crystal structures by Sunami *et al*., included residues 75–399 (52–376) of S6K1, bound to the unselective kinase inhibitor staurosporin. Structures for both unphosphorylated and phosphorylated T252 were presented [22]. The more recent structures by Wang *et al*. involved residues 52–379 or 52–394, bound to the specific S6K1 inhibitor PF-4708671. Several different structures involving different phosphorylations and mutations were solved [23]. Here, we developed two *in silico* structures of the S6K2 kinase domain using homology modelling with two different S6K1 crystal structures, bound to staurosporin (PDB ID: 3A62) or PF-4708671 (PDB ID: 4L3J), respectively, as templates.

**Fig 3:**
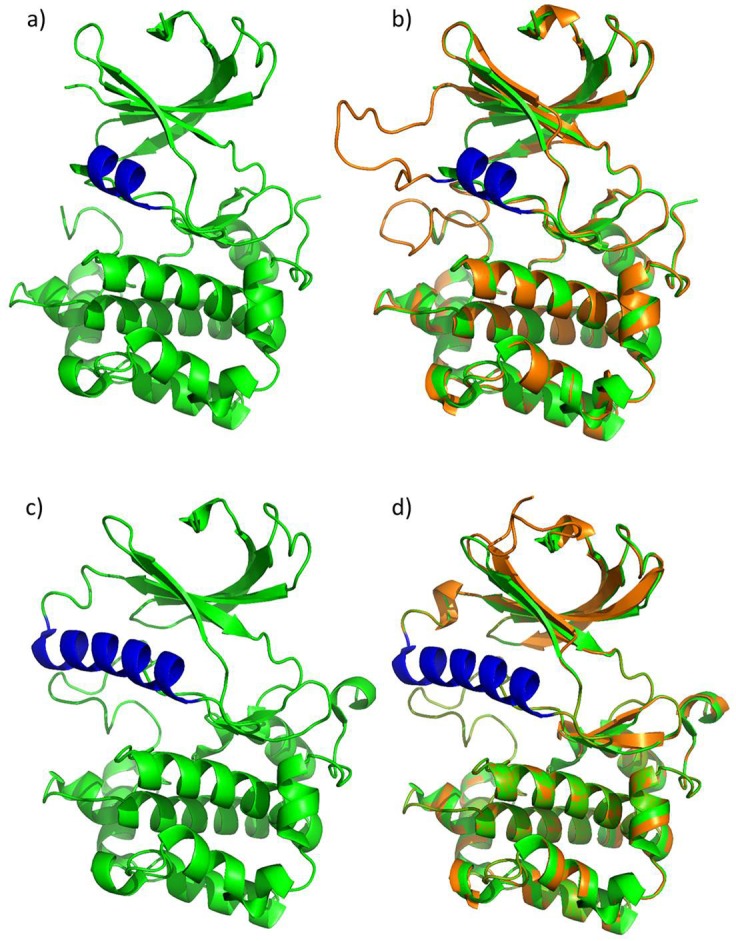
Structural comparison of crystal structures of S6K1 and homology models of S6K2. **(a)** Crystal structure of S6K1 (PDB ID: 3A62) with the αC helix highlighted in blue. **(b)** Overlay of the structure in (a) and a homology model of S6K2 (orange) based on this structure. **(c)** Crystal structure of S6K1 (PDB ID: 4L3J). **(d)** Overlay of the structure in (c) and a homology model of S6K2 based on this structure. The colouring is the same as in the previous panels. The images were rendered in PyMol (Schrödinger LLC). See **S4 Fig** for alternative representations of these images where also key residues are shown.

The S6K1 kinase structure bound to staurosporin **(Fig 3A)** shows relatively low electron density in the αC-helix as well as the activation loop both regulating kinase activity, indicating a highly dynamic structure, although the phosphorylation at T229 partly stabilised the structure. When S6K2 was modelled against this structure, the αC-helix was assumed to be even shorter in S6K2 (residues R120-S125) than in S6K1 (residues A111-E126) **(Fig 3B)**, possibly as a result of differences in neighbouring amino acids, leading to an even more dynamic structure of S6K2. In addition, there were apparent conformation differences in the activation loop, which tentatively are caused by minor sequence variations. The RMSD between model and template is 0.64 Å (backbone), 0.72 Å (all heavy atoms) suggesting that the overall structures are very similar as shown in **Fig 3B**.

The S6K1 kinase structure bound to the specific inhibitor PF-4708671 **(Fig 3C)** shows significantly higher electron density in the αC-helix compared to the staurosporin bound structure, suggesting that the inhibitor stabilises the kinase domain in a non-activating conformation. Consequently, when using this structure as a template, also S6K2 showed a longer, apparently less dynamic, αC-helix **(Fig 3D).** Also in this case the RMSD, 0.16 Å (backbone), 0.31 Å (all heavy atoms), between model and template indicated very minor overall structural differences.

Altogether, the structure comparisons between S6K1 and S6K2 indicate highly similar secondary and tertiary structures. The similarities are likely more pronounced than in reality and the modelled structures are clearly biased towards the used template. It is for instance noteworthy that the two different S6K1 structures that were used as templates deviate more from each other with an RMSD of 1.25 Å (backbone), 1.35 Å (all heavy atoms) than the respective templates and the models. However, even if the structures of S6K1 and S6K2 kinase domains are as similar as the models suggest, there are differences that can be exploited for development of S6K2 specific inhibitors. For instance, the twist of the β-strand following the αC-helix is different from the template in both models. This β-strand is also displaced relative to the templates. Similar differences are also true for several other β-strands in the N-terminal lobe and, reassuringly, this is also true for both models. The most interesting difference is however the already mentioned unique presence of the amino acid cysteine in S6K2 at a position in the ATP-binding pocket occupied by tyrosine in S6K1. This creates a significantly different environment in the region of the protein where most kinase inhibitors are designed to bind in two important ways. First, the surface and volume of this area of the binding pocket changes, explaining why S6K2 cannot accommodate the S6K1 specific inhibitor PF-4708671 efficiently and suggests how a potent S6K2 inhibitor could be designed. Secondly, the reactivity of the cysteine side-chain might be exploited in the design of a specific S6K2 inhibitor.

## Discussion

The mTOR pathway has emerged as an important target in breast cancer therapy, and the first generation of mTOR inhibitors has recently reached the clinic [5]. Although the PI3K/AKT/mTOR pathway is upregulated in a significant proportion of breast cancers, and has been shown a driver of malignancy, far from all patients benefit from treatment with mTOR pathway inhibitors. Most challenging is likely the upregulation of an extensive compensatory feed-back system. Evidently, more knowledge about the mTOR signalling network is needed to improve patient specific treatments targeting this pathway. In this study, we focus on the downstream mTOR effectors S6K1, S6K2 and 4EBP1 that have been shown amplified and overexpressed in breast cancer and associated with an adverse prognosis, and also implicated as markers of outcome after endocrine treatment.

S6K1 and S6K2 are highly homologous and are assumed to have both overlapping as well as divergent cellular functions. Downregulation of either isoform has been shown to lead to compensatory upregulation of the other isoform [35,36]. In the present study, S6K2 mRNA levels were increased after S6K1 downregulation. Silencing of both isoforms lead to upregulation of RPS6KA3 and RPS6KC1, which is also likely a compensatory mechanism for maintaining rpS6 phosphorylation. In addition, a significantly increased expression of Raptor in the mTORC1 complex can be detected after siRNA knock-down of S6K2. Interestingly, double S6K1/S6K2 silencing also lead to increased expression of factors involved in the mTORC2 complex, among those mTOR and Rictor, suggesting a compensatory cross-talk between the two mTOR complexes. Supporting this finding, mTORC1-activated S6K1 has recently been shown able to phosphorylate Rictor, possibly negatively regulating the mTORC2 activity [37].

S6K1 and S6K2 are also assumed to possess certain distinct cellular roles. Accordingly, knock-down of S6K1 or S6K2 had significantly different effects on the genome-wide expression profile of the ZR751 breast cancer cell line. Eight genes were downregulated after S6K1 silencing, but upregulated after S6K2 knock-down, among these the EGFR ligand amphiregulin (AREG), the glutamate-ammonia ligase (GLUL) involved in glutamine synthesis, and the CDK5 and ABL1 enzyme substrate 1 (CABLES1), implicated as a tumour suppressor [38]. S6K2 downregulation also resulted in upregulation of genes connected to metabolism as well as cell cycle progression/checkpoint regulation, which was not seen after S6K1 silencing.

In line with the divergent roles of S6K1 and S6K2, mRNA portraying of S6K1 or S6K2 high expressing tumours revealed large differences in whole genome profiles, with only two overlapping genes, with our stated threshold. These genes, both located at 17q23 in proximity of S6K1, include SMARCD1, involved in transcriptional regulation of certain genes by altering the chromatin structure, and DDB1 and CUL4 associated factor 7 (Han11), a scaffold protein for protein complexes involved in kinase signalling.

One of the top genes inversely correlated to both S6K2 and 4EBP1 was IGF1. The IGFs are main activators of AKT/mTOR signalling [33], indicating that the inverse relationship may reflect a compensatory relationship and mutually exclusive IGF1 or S6K2/4EBP1 levels. IGF1 and IGF2 can signal in an endocrine, paracrine or autocrine manner [33]. IGF1 and IGF2 bind and activate the receptor tyrosine kinase IGF1R, leading to homodimerisation as well as heterodimerisation with the insulin receptor (INSR), subsequent IRS1 recruiting and downstream PI3K/AKT/mTOR activation. IGF2R, in turn, is considered an inhibitor of IGF signalling by binding IGF2, promoting its degradation.

We have previously shown a strong correlation between high levels of S6K2/4EBP1 and poor breast cancer outcome [8]. Interestingly, in the present study, the prognostic value of S6K2/4EBP1 in breast cancer was mainly restricted to tumours expressing high levels of IGFRs or HER2, indicating an important role in growth factor driven tumours. IGF1R and IGF2R are thought to have opposite roles in IGFR signalling, however, addition of IGF2R to the IGF1R and HER2 positive subgroup improved the prognostic value of S6K2/4EBP1, through reasons that need to be further evaluated. ER cross-talk to growth factor receptors as IGFR and HER2 is one suggested mechanism behind endocrine resistance in breast cancer [39–42].

The whole genome-profiles of S6K2 and 4EBP1 high tumours included several genes connected to cell cycle progression, with one of the top genes being the master regulator E2F1. The PI3K/AKT/mTOR/S6K pathway is known to positively regulate translation of cell cycle promoting genes, including cyclin D1 and cMyc [43]. However, the S6 kinases have also been implicated in nuclear processes. The predominant S6K2 isoform p54S6K2 is shuttled between cytoplasm and nucleus, and in response to growth factors, phosphorylation of S473 in the C-terminal domain by PKC inactivates the NLS, rendering S6K2 to be present in the cytoplasm. The S6 kinases, in particular S6K2 have been implicated in cytoskeletal dynamics in the context of cell cycle regulation. Recent data have shown that in mid G1 phase, p70S6K1 becomes phosphorylated by mTOR at T389 and translocated to the nucleus [44]. S6K2, but not S6K1, has been reported to localise to the centrosome during all steps of the cell cycle, suggesting that it may be involved in cytoskeletal regulation through centrosome signalling [45].

The S6 kinases may also be connected to regulation of transcription. S6K1 has been shown to phosphorylate the transcription factor cAMP-responsive element modulator (CREM)τ, [46] as well as ERα [47]. Whether S6K2 is involved in regulation of transcription factor activity remains unknown. However, SH3-binding motifs, which can be found in S6K2, are shown to facilitate interaction of cofactors with ERα [20], allowing to speculate that S6K2 also has the potential to act as a cofactor of ERα. Indeed, a recent study has shown that nuclear S6K2, but not S6K1, associates with several RNA-binding proteins, including the heterogeneous ribonucleoproteins (hnRNPs) [48]. HnRNPs are chromatin-associated nuclear proteins that bind specifically to different RNA sequences and are involved in several steps of RNA processing, including splicing, polyadenylation, mRNA stability and translational regulation [49]. Upon serum stimulation, mTOR associates with the hnRNP-S6K2 complex and activates S6K2 which promotes cell proliferation through unknown mechanisms [48].

Regarding 4EBP1 in the nucleus, a previous study estimated that approximately 30% of the 4EBP1 expressed in cells is located in the nucleus, where it has a role in regulating the availability of EIF4E for the cytoplasmic translational machinery, by retaining EIF4E in the nucleus [50]. High nuclear levels of 4EBP1 would thus inhibit translation and subsequent proliferation, which may explain that the prognostic value of 4EBP1 seems to be dependent of the cellular location of the protein [8].

The connection of E2F1 to S6K2 and 4EBP1, but less to S6K1 remains to be investigated. In our study, S6K2 silencing resulted in a tendency to an upregulation of E2F1 (p = 0.08), as well as several targets of E2F transcription factor activity, which could not be seen for S6K1 and double knock-down. In breast cancer, E2F1 has been implicated in tumour progression through oestrogen dependent as well as independent mechanisms. E2F1 activity is regulated by upstream growth factors signalling pathways, and Ras/MAPK as well as PI3K/AKT pathways have been shown to inhibit E2F1 proapoptotic gene transcription [51]. In addition, AKT has been shown a transcriptional target of E2F1, suggesting positive feedback inhibiting apoptosis. Recent studies have linked E2F1 to mTOR and ER signalling. E2F1 can activate mTORC1 and downstream signalling probably by recruiting mTORC1 to late endosomes, independent of AKT and insulin signalling [52]. ERα was shown to regulate E2F1 expression in late G1, promoting S-phase entry [53]. E2F1 has been shown upregulated in response to tamoxifen binding and recruitment of ER to the E2F1 promoter in tamoxifen resistant cells [54].

The correlation between S6K2 and 4EBP1 in the present study has also been shown at the protein level, however, the relation between S6K2, 4EBP1 and clinicopathological factors seems to be highly dependent on its cellular localisation, but also the molecular and the clinicopathological context [8].

Cytoplasmic p4EBP1 is associated with high grade and poor prognosis, whereas nuclear p4EBP1 is rather associated with a favourable prognosis and low grade. Also S6K2, especially nuclear, is inversely correlated to grade. In addition, nuclear S6K2 was correlated to ER-positivity, whereas cytoplasmic 4EBP1 as well as mRNA in the present study, has been linked to ER-negativity. Altogether, one may speculate that regulation of expression levels, subcellular localisation and phosphorylation states of S6K2 and 4EBP1 may contribute in balance of the cell cycle through transcriptional and translational control of cell cycle regulating genes. In a future study, downregulation of S6K2 and 4EBP1, in particularly nuclear or cytoplasmic specific downregulation respectively, would be interesting to further study this issue.

We have when performed genome-wide comparison of S6K1 and S6K2 positive tumours as well as in breast cancer cells after S6K1 or S6K2 *in vitro* silencing, revealed major significant differences supporting that these tumours should in part be considered as clinically different groups. Consequently, in addition to present day’s mTOR targeted therapies, specific inhibitors for the two p70 S6K isoforms should be of great value. S6K1 and S6K2 belong to the AGC (cAMP-dependent, cGMP-dependent and protein kinase C) family of kinases, also including among others PKA, AKT, PDK1, SGK and p90S6K. The AGC family members have a high homology in their catalytic domains and become active after phosphorylation by PDK1 at a Tyr residue in the activation loop [21]. In our study, homology modelling and structural comparisons of the S6K1 and S6K2 kinase domains have verified subtle but important differences that in part may explain their functional divergences. These include possible differences in the αC-helix, which is essential for the catalytic activity of a kinase [55]. Differences in the amino acid composition in the ATP-binding pocket would also facilitate design of isoform specific inhibitors, since the majority of kinase inhibitors are directed towards this site. The partly unstructured C-terminal regions, with no homology between the isoforms, likely explain most of the different features between the proteins. Indeed exon duplication with addition of a novel exon, encoding a specific structural unit, is considered a common evolutionary strategy to easily develop proteins with novel functions [56].

In conclusion, our study sheds further light on downstream mTOR signalling in breast cancer, supporting that S6K1 and S6K2 signalling may in part possess different roles in tumourigenesis. S6K2, together with 4EBP1 was shown linked to cell cycle regulation through E2F1. In addition, the earlier reported prognostic value of S6K2 and 4EBP1 was found restricted to the IGFR/HER2 positive subgroup, whereas S6K1, in turn, had no prognostic value in this group. A structural comparison of the S6K1 and S6K2 kinase domains revealed significant differences that could be of importance for differences in regulation and function of the two kinases and also useful for future development of isoform-specific inhibitors. The emerging different roles of S6K1 and S6K2 suggest that specific targeting of either isoform may be valuable in different tumour subtypes, and in comparison to present day’s mTOR inhibitors, further promote individualised therapies.

## Supporting Information

S1 Fig Confirmation of S6K1 and S6K2 downregulation at the mRNA level after siRNA treatment for 72 h as well as changes in the expression of mTOR pathway genes. (DOCX)

S2 Fig Sequence alignment and gene organization of S6K1 and S6K2. (DOCX)

S3 Fig Comparison of S6K1 and S6K2 primary structure. (DOCX)

S4 Fig Structural comparison of crystal structures of S6K1 and homology models of S6K2. (DOCX)

S1 Table Patient characteristics of the different cohorts included in this study. (DOCX)

S2 Table Genes and pathways correlated to S6K1 in the van de Vijver cohort. Genes positively correlated to S6K1 and comparison with S6K2 (**Table A**). Pathways positively correlated to S6K1 (**Table B**). Genes inversely correlated to S6K1 and comparison to S6K2 (**Table C**). Pathways inversely correlated to S6K1 (**Table D**)
(DOCX)

S3 Table Genes and pathways correlated with S6K2 in the van de Vijver cohort. Genes positively correlated to S6K2 and comparison with S6K1 (**Table A**). Pathways positively correlated to S6K2 (**Table B**). Genes inversely correlated to S6K2 and comparison to S6K1 (**Table C**). Pathways inversely correlated to S6K2 (**Table D**).
(DOCX)

S4 Table Genes and pathways correlated to 4EBP1 in the van de Vijver cohort. Genes positively correlated to 4EBP1 (**Table A**). Pathways positively correlated to 4EBP1 (**Table B**). Genes inversely correlated to 4EBP1 (**Table C**). Pathways inversely correlated to 4EBP1 (**Table D**).
(DOCX)

S5 Table Genes correlated to both S6K1 and 4EBP1 in the van de Vijver cohort. (DOCX)

S6 Table Genes and pathways correlated to S6K2 but not to S6K1 or 4EBP1 in the van de Vijver cohort. Genes positively correlated to S6K2 only (**Table A**). Genes inversely correlated to S6K2 only (**Table B**). Pathways positively correlated to S6K2 only (**Table C**). Pathways inversely correlated to S6K2 only (**Table D**).
(DOCX)

S7 Table Genes and pathways correlated to 4EBP1 but not to S6K1 or S6K2 in the van de Vijver cohort. Genes positively correlated to 4EBP1 only (**Table A**). Pathways correlated positively to 4EBP1 only (**Table B**). Genes inversely correlated to 4EBP1 only (**Table C**). Pathways inversely correlated to 4EBP1 only (**Table D**).
(DOCX)

S8 Table Alteration in gene expression and associated pathways following S6K1 and S6K2 siRNA silencing. Genes upregulated in response to both S6K1 and S6K2 siRNA (**Table A**). Pathways upregulated in response to both S6K1 and S6K2 siRNA (**Table B**). Genes downregulated in response to both S6K1 and S6K2 siRNA (**Table C**).
(DOCX)

S9 Table Alteration in gene expression and associated pathways following double S6K1/S6K2 siRNA but not single siRNA silencing. Genes upregulated in response to double S6K1/S6K2 siRNA, but not to single siRNA (**Table A**). Pathways upregulated in response to S6K1/S6K2 siRNA, but not to single siRNA (**Table B**). Genes downregulated in response to double S6K1/S6K2 siRNA, but not to single siRNA (**Table C**). Pathways downregulated in response to double S6K1/S6K2 siRNA, but not to single siRNA (**Table D**).
(DOCX)

S10 Table Alteration in gene expression and associated pathways following S6K1 siRNA but not S6K2 siRNA silencing. Genes upregulated in response to S6K1 siRNA, but not to S6K2 siRNA (**Table A**). Pathways upregulated in response to S6K1 siRNA, but not to S6K2 siRNA (**Table B**). Genes downregulated in response to S6K1 siRNA, but not to S6K2 siRNA (**Table C**). Pathways significantly downregulated in response to S6K1 siRNA, but not to S6K2 siRNA (**Table D**).
(DOCX)

S11 Table Alteration in gene expression and associated pathways following S6K2 siRNA but not S6K1 siRNA silencing. Genes upregulated in response to S6K2 siRNA, but not to S6K1 siRNA (**Table A**). Pathways upregulated in response to S6K2 siRNA, but not to S6K1 siRNA (**Table B**). Genes upregulated in response to S6K2 siRNA, but not to S6K1 siRNA (**Table C**). Pathways downregulated in response to S6K2 siRNA, but not to S6K1 siRNA (**Table D**).
(DOCX)
