# Supplementary material for: Revealing Different Roles of the mTOR-Targets S6K1 and S6K2 in Breast Cancer by Expression Profiling and Structural Analysis
Source: PLoS One. 2015 Dec 23;10(12):e0145013. doi: 10.1371/journal.pone.0145013 (PMC4689523; doi:10.1371/journal.pone.0145013)
Supplement: S1 Fig — (DOCX) [file pone.0145013.s001.docx]

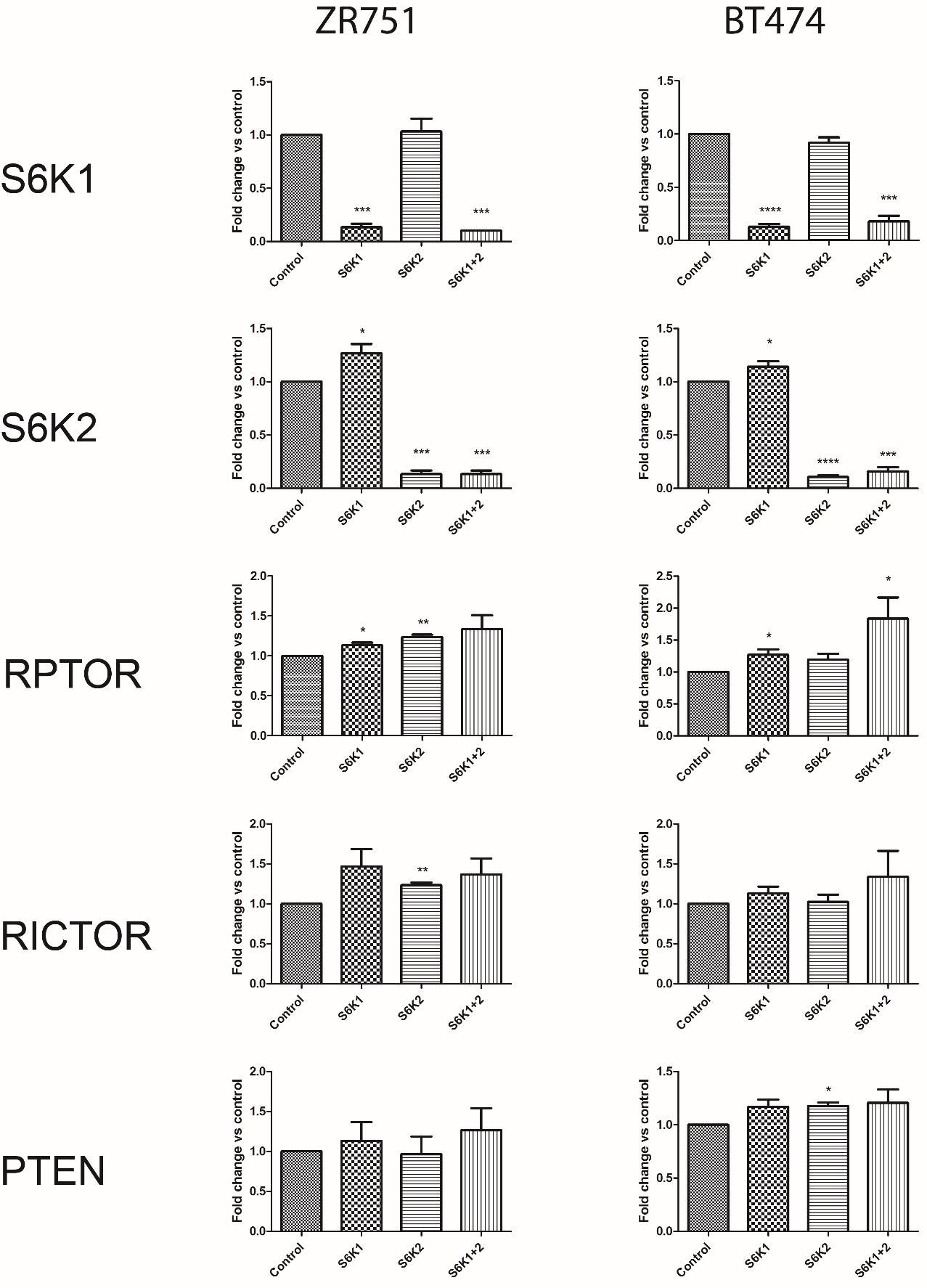


**S1 Fig.** **Confirmation of S6K1 and S6K2 downregulation at the mRNA level and changes in the expression of mTOR pathway genes after siRNA treatment for 72 h.** Expression of S6K1, S6K2, RPTOR, RICTOR and PTEN in the cell lines ZR751 and BT474 was analysed by real-time PCR.
