# Supplementary material for: Revealing Different Roles of the mTOR-Targets S6K1 and S6K2 in Breast Cancer by Expression Profiling and Structural Analysis
Source: PLoS One. 2015 Dec 23;10(12):e0145013. doi: 10.1371/journal.pone.0145013 (PMC4689523; doi:10.1371/journal.pone.0145013)
Supplement: S2 Fig — (DOCX) [file pone.0145013.s002.docx]

S6K1 1 MAGVFDIDLD-------------QPEDAGSEDELEEGGQLNESMDHGGVGPYELGMEHCE 47

MA VFD+DL+ P DA EL G + P + H E

S6K2 1 MAAVFDLDLETEEGSEGEGEPELSPADACPLAELRAAG----------LEP----VGHYE 46

S6K1 48 KF**E**ISETSVNRGPEKIRPECFELLRVLGKGGYGKVFQVRKVTGANTGKIFAMKVLKKAMI 107

+ E++ETSVN GPE+I P CFELLRVLGKGGYGKVFQVRKV G N GKI+AMKVL+KA I

S6K2 47 EVEL**T**ETSVNVGPERIGPHCFELLRVLGKGGYGKVFQVRKVQGTNLGKIYAMKVLRKAKI 106

S6K1 108 VRNAKDTAHTKAERNILEEVKHPFIVDLIYAFQTGGKLYLILEYLSGGELFMQLEREGIF 167

VRNAKDTAHT+AERNILE VKHPFIV+L YAFQTGGKLYLILE LSGGELF LEREGIF

S6K2 107 VRNAKDTAHTRAERNILESVKHPFIVELAYAFQTGGKLYLILECLSGGELFTHLEREGIF 166

S6K1 168 MEDTACFYLAEISMALGHLHQKGIIYRDLKPENIMLNHQGHVKLTDFGLCKESIHDGTVT 227

+EDTACFYLAEI++ALGHLH +GIIYRDLKPENIML+ QGH+KLTDFGLCKESIH+G VT

S6K2 167 LEDTACFYLAEITLALGHLHSQGIIYRDLKPENIMLSSQGHIKLTDFGLCKESIHEGAVT 226

S6K1 228 HTFCGTIEYMAPEILMRSGHNRAVDWWSLGALMYDMLTGAPPFTGENRKKTIDKILKCKL 387

HTFCGTIEYMAPEIL+RSGHNRAVDWWSLGALMYDMLTG+PPFT ENRKKT+DKI++ KL

S6K2 227 HTFCGTIEYMAPEILVRSGHNRAVDWWSLGALMYDMLTGSPPFTAENRKKTMDKIIRGKL 286

S6K1 288 NLPPYLTQEARDLLKKLLKRNAASRLGAGPGDAGEVQAHPFFRHINWEELLARKVEPPFK 347

LPPYLT +ARDL+KK LKRN + R+G GPGDA +VQ HPFFRH+NW++LLA +V+PPF+

S6K2 287 ALPPYLTPDARDLVKKFLKRNPSQRIGGGPGDAADVQRHPFFRHMNWDDLLAWRVDPPFR 346

S6K1 348 PLLQSEEDVSQFDSKFTRQTPVDSPDDST**L**SESANQVFLGFTYVAPSVLESVKEKFSFEP 407

P LQSEEDVSQFD++FTRQTPVDSPDD+ LSESANQ FLGFTYVAPSVL+S+KE FSF+P

S6K2 347 PCLQSEEDVSQFDTRFTRQTPVDSPDDT**A**LSESANQAFLGFTYVAPSVLDSIKEGFSFQP 406

S6K1 408 KIRSPRRFIGSPRTPVSPVKFSP 430

K+RSPRR SPR PVSP+KFSP

S6K2 407 KLRSPRRLNSSPRAPVSPLKFSP 429

| Exon number | S6K1 (p70)^1^ | S6K2 (p54)^1^ | Exon length S6K1 | Exon length S6K2 | Kinase domain |
| --- | --- | --- | --- | --- | --- |
| 1 | 1-24 | 1-26 | 23 | 25 |  |
| 2 | 25-41 | 27-40 | 16 | 13 |  |
| 3 | **42-81** | **41-80** | **39** | 39 | x |
| 4 | **82-104** | **81-103** | **22** | 22 | x |
| 5 | **105-154** | **104-153** | **49** | 49 | x |
| 6 | **155-173** | **153-172** | **18** | 18 | x |
| 7 | **174-207** | **173-206** | **33** | 33 | x |
| 8 | **208-237** | **207-236** | **29** | 29 | x |
| 9 | **238-267** | **237-266** | **29** | 29 | x |
| 10 | **268-303** | **267-302** | **35** | 35 | x |
| 11 | **304-324** | **303-323** | **20** | 20 | x |
| 12 | **325-350** | **324-349** | **25** | 25 | x |
| 13 | **351-386** | **350-385** | **35** | 35 | x |
| 14 | **387-424** | **386-423** | **37** | 37 | x |
| 15 | 425-502 | 424-482 | 77 | 58 |  |

^1^For the variants p85 (S6K1) and p56 (S6K2) there are 23 and 13 additional amino acid residues NLS included in first exon, respectively.

**S2 Fig. Sequence alignment and gene organization of S6K1 and S6K2.**
