# Supplementary material for: Revealing Different Roles of the mTOR-Targets S6K1 and S6K2 in Breast Cancer by Expression Profiling and Structural Analysis
Source: PLoS One. 2015 Dec 23;10(12):e0145013. doi: 10.1371/journal.pone.0145013 (PMC4689523; doi:10.1371/journal.pone.0145013)
Supplement: S3 Fig — (DOCX) [file pone.0145013.s003.docx]

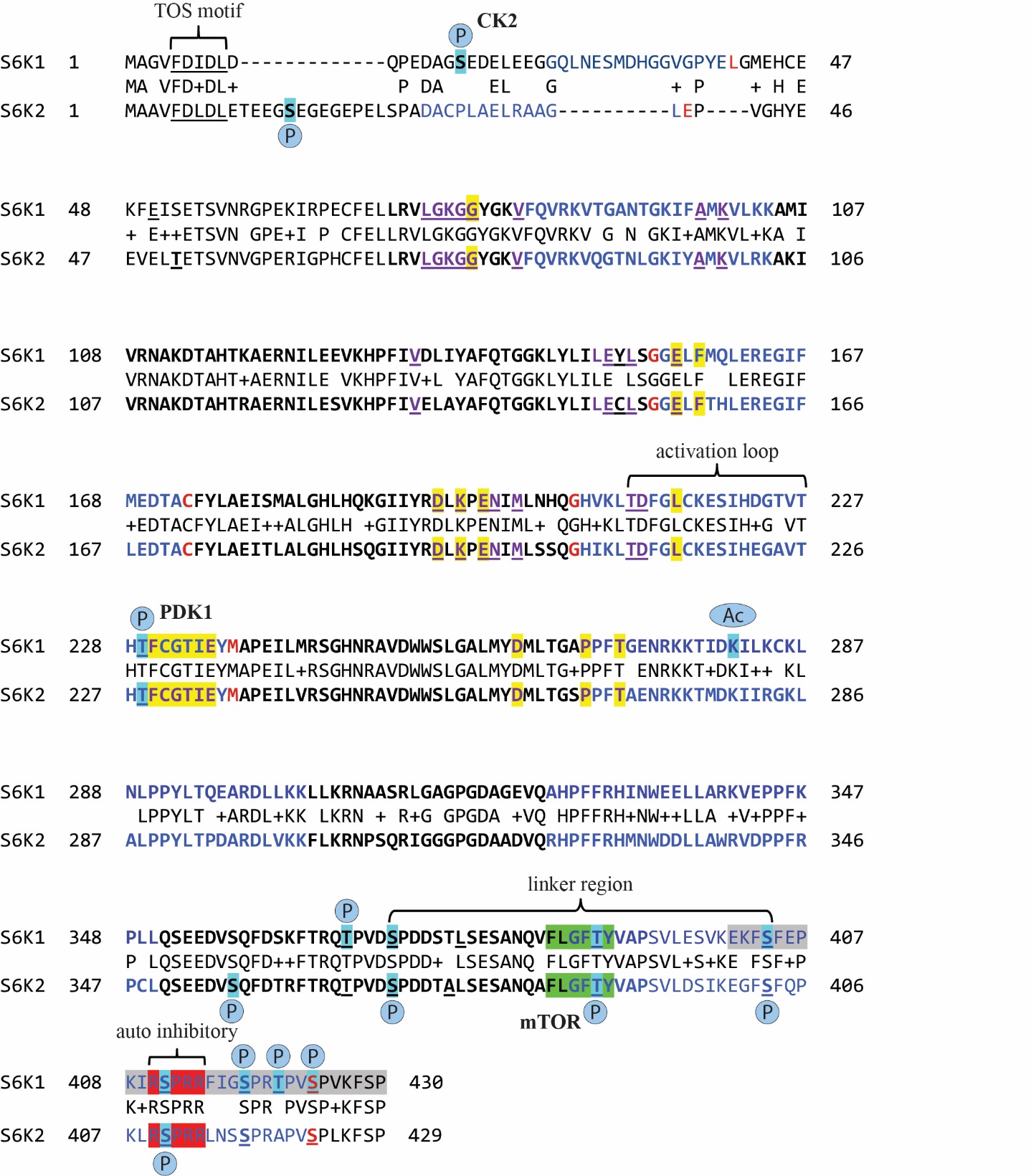


**S3 Fig.** **Comparison of S6K1 and S6K2 primary structure.** Catalytic domain (bold), active site (purple), ATP-binding site (underlined), substrate binding site (yellow highlight), hydrophobic motif (green highlight), autoinhibitory domain (grey background, red specific motif).
