# Supplementary material for: Revealing Different Roles of the mTOR-Targets S6K1 and S6K2 in Breast Cancer by Expression Profiling and Structural Analysis
Source: PLoS One. 2015 Dec 23;10(12):e0145013. doi: 10.1371/journal.pone.0145013 (PMC4689523; doi:10.1371/journal.pone.0145013)
Supplement: S4 Fig — (DOCX) [file pone.0145013.s004.docx]

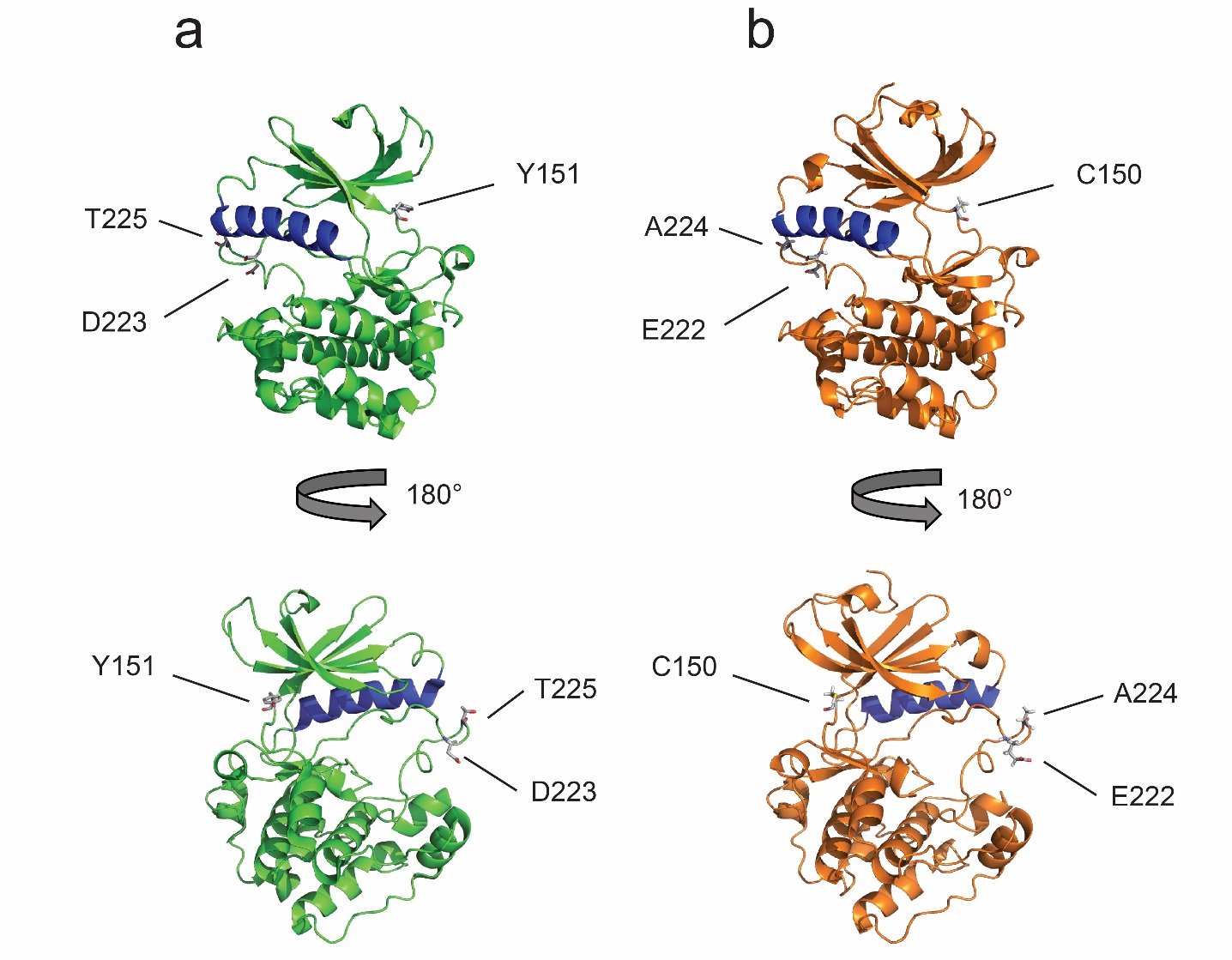


**S4 Fig.** **Structural comparison of crystal structures of S6K1 and homology models of S6K2.** The αC helix is shown in blue and the side chains of key residues mentioned in the text are rendered as sticks. **(a)** Crystal structure of S6K1 (PDB ID: 4L3J). **(b)** Homology model of S6K2 based on the structure in (a).
