# Supplementary material for: Revealing Different Roles of the mTOR-Targets S6K1 and S6K2 in Breast Cancer by Expression Profiling and Structural Analysis
Source: PLoS One. 2015 Dec 23;10(12):e0145013. doi: 10.1371/journal.pone.0145013 (PMC4689523; doi:10.1371/journal.pone.0145013)
Supplement: S1 Table — (DOCX) [file pone.0145013.s005.docx]

**S1 Table.** **Patient characteristics of the different cohorts included in this study.**

|  | ***Van de Vijver***  *(n=295)* | | ***Uppsala***  *(n=251)* | ***Karolinska***  *(n=159)* | |  |  |  |
| --- | --- | --- | --- | --- | --- | --- | --- | --- |
|  |  |  |  |  |  |  |  |  |
| ***Tumour size*** |  | |  |  | |  |  |  |
| ***< 20 mm*** | 155 (53.0) | | 127 (50.6) | 97 (61.0) | |  |  |  |
| ***>20 mm*** | 140 (47.0) | | 124 (49.4) | 60 (37.7) | |  |  |  |
| *missing* | 0 (0) | | 0 (0) | 2 (1.3) | |  |  |  |
|  |  | |  |  | |  |  |  |
| ***Lymph node status*** |  | |  |  | |  |  |  |
| ***-*** | 151 (51.2) | | 158 (62.9) | 94 (59.1) | |  |  |  |
| ***+*** | 144 (48.8) | | 84 (33.5) | 60 (37.7) | |  |  |  |
| *missing* | 0 (0) | | 9 (3.6) | 5 (3.1) | |  |  |  |
|  |  | |  |  | |  |  |  |
| ***Grade*** |  | |  |  | |  |  |  |
| ***1*** | 75 (25.4) | | 67 (26.7) | 28 (17.6) | |  |  |  |
| ***2*** | 101 (34.2) | | 128 (51.0) | 58 (36.5) | |  |  |  |
| ***3*** | 119 (40.3) | | 54 (21.5) | 61 (38.4) | |  |  |  |
| *missing* | 0 (0) | | 2 (0.8) | 12 (7.5) | |  |  |  |
|  |  | |  |  | |  |  |  |
| ***ER*** |  | |  |  | |  |  |  |
| ***-*** | 69 (23.4) | | 34 (13.5) | 29 (18.2) | |  |  |  |
| ***+*** | 226 (76.6) | | 213 (84.9) | 130 (81.8) | |  |  |  |
| *missing* | 0 (0) | | 4 (1.6) | 0 (0) | |  |  |  |
|  |  | |  |  | |  |  |  |
| ***PgR*** | N/A | |  | N/A | |  |  |  |
| ***-*** |  | | 61 (24.3) |  | |  |  |  |
| ***+*** |  | | 190 (75.7) |  | |  |  |  |
| *missing* |  | | 0 (0) |  | |  |  |  |
|  |  | |  |  | |  |  |  |
| ***HER2*** | N/A | | N/A | N/A | |  |  |  |
| ***-*** |  | |  |  | |  |  |  |
| ***+*** |  | |  |  | |  |  |  |
| *missing* |  | |  |  | |  |  |  |
|  |  |  | | |  | |  |  |
|  |  |  | | |  | |  |  |
