# Supplementary material for: Revealing Different Roles of the mTOR-Targets S6K1 and S6K2 in Breast Cancer by Expression Profiling and Structural Analysis
Source: PLoS One. 2015 Dec 23;10(12):e0145013. doi: 10.1371/journal.pone.0145013 (PMC4689523; doi:10.1371/journal.pone.0145013)
Supplement: S2 Table — Genes positively correlated to S6K1 and comparison with S6K2 (Table A). Pathways positively correlated to S6K1 (Table B). Genes inversely correlated to S6K1 and comparison to S6K2 (Table C). Pathways inversely correlated to S6K1 (Table D) (DOCX) [file pone.0145013.s006.docx]

**Table A.** **Genes positively correlated to S6K1 and comparison with S6K2.**

| Genes correlated with S6K1 | S6K  t-statistic | S6K1  p-value | S6K2  t-statistic | S6K2  p-value |
| --- | --- | --- | --- | --- |
| **NM_003161__RPS6KB1** | 20.6821 | 1.22E-45 | 1.63322 | 0.104563 |
| **Contig27624_RC** | 12.7438 | 1.27E-25 | 0.717793 | 0.474024 |
| **NM_016424__LUC7A** | 11.426 | 4.14E-22 | -1.42976 | 0.154908 |
| **NM_016077__LOC51651** | 11.2309 | 1.37E-21 | 2.93808 | 0.003836 |
| **NM_004459__FALZ** | 11.1384 | 2.42E-21 | -0.08264 | 0.934253 |
| **NM_006380__APPBP2** | 11.0792 | 3.47E-21 | 1.1419 | 0.255352 |
| **NM_005121__TRAP240** | 10.0525 | 1.80E-18 | 2.25901 | 0.025354 |
| **Contig45862_RC** | 9.91625 | 4.11E-18 | -1.54947 | 0.123419 |
| **AJ012755** | 9.48201 | 5.58E-17 | -2.81241 | 0.005589 |
| **AF161410** | 9.35394 | 1.20E-16 | 0.620866 | 0.535649 |
| **Contig50106_RC** | 9.266 | 2.02E-16 | 1.24926 | 0.213556 |
| **AF146277__CD2AP** | 9.25837 | 2.11E-16 | -1.05115 | 0.294915 |
| **NM_016283__LOC51578** | 9.20446 | 2.91E-16 | -0.0575 | 0.954227 |
| **NM_002816__PSMD12** | 9.0842 | 5.94E-16 | 1.09904 | 0.273547 |
| **Contig48799_RC** | 8.9986 | 9.85E-16 | -1.46912 | 0.143937 |
| **NM_021047__LOC56242** | 8.97261 | 1.15E-15 | -1.73406 | 0.085004 |
| **NM_007043__HRB2** | 8.87128 | 2.08E-15 | -0.38305 | 0.702235 |
| **NM_004713__SDCCAG1** | 8.86651 | 2.14E-15 | -2.24688 | 0.026137 |
| **Contig52296_RC** | 8.85715 | 2.26E-15 | -0.48774 | 0.626459 |
| **Contig24351_RC** | 8.7972 | 3.22E-15 | -2.18962 | 0.030129 |
| **Contig49685_RC** | 8.75281 | 4.18E-15 | -1.9461 | 0.05355 |
| **NM_016570__LOC51290** | 8.7409 | 4.48E-15 | -0.46831 | 0.640258 |
| **Contig53038_RC** | 8.71916 | 5.08E-15 | -2.67672 | 0.008279 |
| **NM_014497__NP220** | 8.68405 | 6.24E-15 | -1.61284 | 0.108924 |
| **NM_005000__NDUFA5** | 8.6395 | 8.09E-15 | -2.87663 | 0.004618 |
| **NM_016648__HDCMA18P** | 8.60861 | 9.69E-15 | -2.10474 | 0.037015 |
| **Contig56469_RC** | 8.59162 | 1.07E-14 | 0.111927 | 0.911034 |
| **AF162667__TLK2** | 8.56375 | 1.26E-14 | 3.16484 | 0.001886 |
| **AB033113__KIAA1287** | 8.52685 | 1.56E-14 | 1.05484 | 0.293229 |
| **Contig52421_RC** | 8.42518 | 2.81E-14 | 0.943289 | 0.347081 |
| **NM_004859__CLTC** | 8.41792 | 2.93E-14 | 2.09833 | 0.037586 |
| **NM_003423__ZNF43** | 8.286 | 6.27E-14 | -1.79781 | 0.07426 |
| **NM_003262__TLOC1** | 8.2829 | 6.38E-14 | -1.58035 | 0.116176 |
| **NM_006107__OA48-18** | 8.26188 | 7.20E-14 | -1.75328 | 0.081638 |
| **NM_020198__GK001** | 8.23413 | 8.45E-14 | 2.1236 | 0.035378 |
| **NM_016627__LOC51321** | 8.22753 | 8.77E-14 | 1.94274 | 0.053959 |
| **NM_002956__RSN** | 8.20849 | 9.79E-14 | -3.16501 | 0.001885 |
| **NM_004505__USP6** | 8.2018 | 1.02E-13 | -2.05543 | 0.041607 |
| **AF155117** | 8.18281 | 1.13E-13 | 1.99179 | 0.048247 |
| **AF227899__KIAA0117** | 8.10524 | 1.77E-13 | -2.70878 | 0.007555 |
| **Contig39797_RC** | 8.01374 | 2.97E-13 | 1.73403 | 0.085009 |
| **Contig37858_RC** | 7.97224 | 3.77E-13 | -2.54472 | 0.011968 |
| **AF049524__HYPA** | 7.95255 | 4.21E-13 | 0.230636 | 0.817918 |
| **NM_004986__KTN1** | 7.90149 | 5.62E-13 | -1.6178 | 0.107849 |
| **NM_003135__SRP19** | 7.89897 | 5.70E-13 | 0.608305 | 0.543924 |
| **NM_004396__DDX5** | 7.89028 | 5.99E-13 | -0.46706 | 0.64115 |
| **Contig37826** | 7.86995 | 6.72E-13 | -2.79199 | 0.005935 |
| **NM_002431__MNAT1** | 7.86569 | 6.88E-13 | 0.328589 | 0.742934 |
| **NM_017892__FLJ20585** | 7.86006 | 7.10E-13 | -1.33261 | 0.184722 |
| **NM_002013__FKBP3** | 7.84683 | 7.65E-13 | 0.746937 | 0.456295 |
| **Contig55769_RC__FLJ22087** | 7.82008 | 8.90E-13 | 2.28021 | 0.024033 |
| **Contig53658_RC** | 7.80783 | 9.53E-13 | -1.70888 | 0.089584 |
| **NM_003428__ZNF84** | 7.79763 | 1.01E-12 | -1.67084 | 0.096881 |
| **AL133031** | 7.79594 | 1.02E-12 | -0.86578 | 0.388023 |
| **Contig30350_RC** | 7.75623 | 1.27E-12 | -1.11273 | 0.267641 |
| **NM_001263__CDS1** | 7.74414 | 1.36E-12 | 0.366021 | 0.714876 |
| **NM_006024__TAX1BP1** | 7.71544 | 1.60E-12 | -1.06566 | 0.288325 |
| **NM_004600__SSA2** | 7.71255 | 1.63E-12 | 0.823267 | 0.41169 |
| **AL080192** | 7.69535 | 1.79E-12 | -1.62941 | 0.105367 |
| **X98494__MPHOSPH10** | 7.66028 | 2.18E-12 | 0.191676 | 0.848261 |
| **Contig51453_RC** | 7.62251 | 2.69E-12 | -2.08213 | 0.039064 |
| **NM_004645__COIL** | 7.62232 | 2.69E-12 | 1.74047 | 0.083869 |
| **NM_016125__LOC51136** | 7.62069 | 2.72E-12 | 2.13071 | 0.034777 |
| **NM_016220__ZFD25** | 7.61802 | 2.76E-12 | -2.16216 | 0.032224 |
| **NM_001656__ARFD1** | 7.61577 | 2.80E-12 | -3.91507 | 0.000138 |
| **AL133580__HRIHFB2072** | 7.60414 | 2.98E-12 | -0.23083 | 0.817769 |
| **Contig54661_RC** | 7.59006 | 3.23E-12 | -1.42484 | 0.156323 |
| **NM_003430__ZNF91** | 7.58626 | 3.29E-12 | -1.55452 | 0.122211 |
| **NM_014781__KIAA0203** | 7.55499 | 3.92E-12 | -2.82056 | 0.005457 |
| **NM_001270__CHD1** | 7.55226 | 3.98E-12 | -1.861 | 0.064742 |
| **AB040937__KIAA1504** | 7.52895 | 4.53E-12 | -0.39641 | 0.69238 |
| **NM_004702__CCNE2** | 7.49668 | 5.42E-12 | 4.01573 | 9.42E-05 |
| **Contig41244_RC** | 7.49487 | 5.47E-12 | -1.23122 | 0.220207 |
| **AL050378** | 7.47245 | 6.19E-12 | -0.70206 | 0.483755 |
| **NM_020640__RP42** | 7.46234 | 6.55E-12 | -0.06389 | 0.949148 |
| **Contig41078_RC** | 7.45699 | 6.74E-12 | 0.549662 | 0.583385 |
| **Contig17400_RC** | 7.45017 | 7.00E-12 | 1.24569 | 0.21486 |
| **NM_016227__CH1** | 7.42792 | 7.92E-12 | -1.07244 | 0.28528 |
| **NM_014283__CH1** | 7.42584 | 8.01E-12 | -1.28291 | 0.201543 |
| **Contig33121_RC** | 7.40718 | 8.88E-12 | 2.52851 | 0.01251 |
| **Contig51791_RC** | 7.39659 | 9.41E-12 | -3.3305 | 0.001096 |
| **NM_004365__CETN3** | 7.37155 | 1.08E-11 | -0.29326 | 0.76974 |
| **NM_004521__KIF5B** | 7.37095 | 1.08E-11 | 1.31253 | 0.191387 |
| **NM_005760__CBF2** | 7.36748 | 1.10E-11 | -0.20248 | 0.83982 |
| **AB020705__MUL** | 7.36674 | 1.11E-11 | 1.32824 | 0.186157 |
| **NM_005749__TOB1** | 7.34612 | 1.24E-11 | 0.9632 | 0.337029 |
| **NM_016649__HDCMC28P** | 7.34037 | 1.28E-11 | -0.66368 | 0.507937 |
| **Contig57142_RC** | 7.33958 | 1.29E-11 | -2.45103 | 0.015418 |
| **AL050205** | 7.33425 | 1.33E-11 | -1.52348 | 0.129787 |
| **NM_004487__GOLGB1** | 7.31644 | 1.46E-11 | -1.15923 | 0.248242 |
| **NM_001788__CDC10** | 7.29045 | 1.68E-11 | -3.33811 | 0.001069 |
| **NM_004719__SFRS2IP** | 7.28742 | 1.71E-11 | -1.66301 | 0.098441 |
| **AK001452** | 7.27457 | 1.84E-11 | -1.64191 | 0.102746 |
| **NM_015462__DKFZP586L0724** | 7.27457 | 1.84E-11 | 2.14611 | 0.033506 |
| **NM_018002__OXR1** | 7.27404 | 1.84E-11 | -1.33945 | 0.182491 |
| **Contig58637_RC** | 7.26017 | 1.99E-11 | -1.663 | 0.098443 |
| **NM_016504__LOC51264** | 7.25655 | 2.03E-11 | 2.68547 | 0.008076 |
| **NM_002734__PRKAR1A** | 7.25024 | 2.10E-11 | -1.00874 | 0.314757 |
| **NM_000269__NME1** | 7.24737 | 2.13E-11 | 4.63313 | 7.87E-06 |
| **NM_014810__KIAA0480** | 7.24372 | 2.17E-11 | -2.17493 | 0.031234 |
| **NM_014673__KIAA0103** | 7.23418 | 2.29E-11 | 0.230222 | 0.818239 |
| **Contig35800_RC** | 7.23022 | 2.34E-11 | 0.030299 | 0.97587 |
| **NM_016163__KIAA0917** | 7.21296 | 2.57E-11 | -2.20661 | 0.028893 |
| **NM_001813__CENPE** | 7.21016 | 2.61E-11 | 2.50156 | 0.013461 |
| **AK001595** | 7.20664 | 2.66E-11 | 2.05405 | 0.041742 |
| **NM_014399__NET-6** | 7.20515 | 2.68E-11 | -1.00823 | 0.315001 |
| **NM_003601__SMARCA5** | 7.18196 | 3.04E-11 | 0.385286 | 0.700582 |
| **Contig49684_RC** | 7.16296 | 3.37E-11 | -1.37279 | 0.171908 |
| **Contig48422_RC** | 7.15633 | 3.50E-11 | -1.15433 | 0.250238 |
| **NM_017944__FLJ20727** | 7.13904 | 3.84E-11 | -1.59662 | 0.112498 |
| **AK001672__KIAA1596** | 7.13763 | 3.87E-11 | -2.02259 | 0.044929 |
| **NM_002687__PNN** | 7.13177 | 4.00E-11 | -2.37241 | 0.018967 |
| **NM_002873__RAD17** | 7.11887 | 4.29E-11 | -3.01223 | 0.003054 |
| **NM_004239__TRIP11** | 7.11329 | 4.42E-11 | -1.39374 | 0.1655 |
| **NM_013254__TBK1** | 7.11009 | 4.49E-11 | -2.04035 | 0.043105 |
| **AB014548__KIAA0648** | 7.10651 | 4.58E-11 | -1.73667 | 0.08454 |
| **Contig51828_RC** | 7.07739 | 5.36E-11 | -2.3895 | 0.018139 |
| **NM_007114__TMF1** | 7.07453 | 5.45E-11 | -1.09908 | 0.27353 |
| **NM_004898__CLOCK** | 7.07277 | 5.50E-11 | -0.03285 | 0.973838 |
| **AL133064** | 7.06753 | 5.66E-11 | -1.34158 | 0.181801 |
| **NM_005827__UGTREL1** | 7.05464 | 6.06E-11 | 3.44799 | 0.000737 |
| **NM_014645__KIAA0635** | 7.05279 | 6.12E-11 | -0.67135 | 0.503049 |
| **NM_002788__PSMA3** | 7.05134 | 6.17E-11 | 2.69145 | 0.007939 |
| **Contig40090_RC** | 7.04747 | 6.30E-11 | 0.35181 | 0.725485 |
| **Contig50134_RC** | 7.04664 | 6.33E-11 | 0.425062 | 0.671413 |
| **Contig58256_RC__S164** | 7.03333 | 6.80E-11 | -2.10196 | 0.037262 |
| **NM_018466__MDS031** | 7.02379 | 7.16E-11 | 0.452684 | 0.651444 |
| **NM_003666__BLZF1** | 7.01304 | 7.58E-11 | -1.91179 | 0.057849 |
| **AB033066__KIAA1240** | 7.00466 | 7.93E-11 | -1.01329 | 0.312587 |
| **NM_013450__BAZ2B** | 6.97711 | 9.20E-11 | -2.72494 | 0.007212 |
| **NM_015153__KIAA0244** | 6.96868 | 9.62E-11 | -3.21595 | 0.001599 |
| **NM_017631__FLJ20035** | 6.96201 | 9.97E-11 | 0.403813 | 0.686937 |
| **NM_014500__HTATSF1** | 6.94587 | 1.09E-10 | -1.1875 | 0.236946 |
| **Contig37941_RC** | 6.93508 | 1.15E-10 | -3.94421 | 0.000124 |
| **Contig1946_RC** | 6.92919 | 1.19E-10 | -2.79393 | 0.005902 |
| **Contig43688_RC** | 6.90865 | 1.33E-10 | 1.53253 | 0.127541 |
| **Contig27142_RC** | 6.90791 | 1.33E-10 | -1.96024 | 0.051859 |
| **NM_004792__CYP** | 6.89059 | 1.46E-10 | -1.80948 | 0.07242 |
| **Contig331_RC** | 6.88865 | 1.48E-10 | 0.234749 | 0.81473 |
| **Contig44903__LOC51174** | 6.88861 | 1.48E-10 | 3.05755 | 0.002652 |
| **NM_017934__FLJ20705** | 6.88138 | 1.53E-10 | -1.64214 | 0.102699 |
| **Contig43083_RC** | 6.86739 | 1.65E-10 | -2.07153 | 0.040057 |
| **NM_018695__LOC55914** | 6.85675 | 1.75E-10 | -3.14056 | 0.002039 |
| **NM_002979__SCP2** | 6.84013 | 1.91E-10 | -1.97932 | 0.049648 |
| **Contig37371_RC** | 6.83663 | 1.95E-10 | -1.67798 | 0.095476 |
| **Contig36432_RC** | 6.82666 | 2.05E-10 | -0.45921 | 0.646765 |
| **NM_006218__PIK3CA** | 6.82372 | 2.08E-10 | -2.2541 | 0.025668 |
| **NM_005463__HNRPDL** | 6.8125 | 2.21E-10 | -0.30595 | 0.760076 |
| **NM_004888__ATP6J** | 6.80363 | 2.32E-10 | 0.546592 | 0.585488 |
| **Contig43330_RC** | 6.79638 | 2.41E-10 | 2.08178 | 0.039096 |
| **AF007217__TRIP11** | 6.78047 | 2.62E-10 | -1.732 | 0.085371 |
| **NM_003092__SNRPB2** | 6.76559 | 2.84E-10 | 0.932786 | 0.35246 |
| **NM_002078__GOLGA4** | 6.75888 | 2.94E-10 | -2.46252 | 0.014952 |
| **AL110161** | 6.75572 | 2.99E-10 | -1.4667 | 0.144594 |
| **NM_019002__ETAA16** | 6.75162 | 3.05E-10 | -1.54966 | 0.123373 |
| **X62534__HMG2** | 6.75156 | 3.05E-10 | 1.54881 | 0.123578 |
| **Contig49197_RC** | 6.74858 | 3.10E-10 | -2.83766 | 0.005187 |
| **NM_018169__FLJ10652** | 6.74698 | 3.13E-10 | -2.22585 | 0.027546 |
| **NM_002876__RAD51C** | 6.73751 | 3.29E-10 | 2.09368 | 0.038005 |
| **NM_001812__CENPC1** | 6.72166 | 3.57E-10 | -2.4162 | 0.01691 |
| **NM_014739__KIAA0164** | 6.71564 | 3.69E-10 | -2.1171 | 0.035935 |
| **NM_014597__HSU15552** | 6.70036 | 4.00E-10 | -1.17901 | 0.240299 |
| **AL137753__KIAA1033** | 6.69517 | 4.11E-10 | -2.14993 | 0.033197 |
| **NM_018346__FLJ11164** | 6.68841 | 4.26E-10 | -1.19023 | 0.235875 |
| **X15341__COX6A1** | 6.68138 | 4.42E-10 | 3.33052 | 0.001096 |
| **NM_006447__USP16** | 6.66843 | 4.73E-10 | -1.33024 | 0.185499 |
| **Contig45642_RC** | 6.6666 | 4.77E-10 | -0.64028 | 0.522985 |
| **AL133117** | 6.66588 | 4.79E-10 | -1.91917 | 0.056901 |
| **Contig44712_RC** | 6.65732 | 5.01E-10 | -0.30124 | 0.763659 |
| **Contig57081_RC** | 6.6529 | 5.13E-10 | 0.242308 | 0.808879 |
| **NM_014720__KIAA0204** | 6.65015 | 5.20E-10 | -2.56715 | 0.011253 |
| **AL049309** | 6.63601 | 5.60E-10 | -3.82093 | 0.000196 |
| **NM_002805__PSMC5** | 6.63501 | 5.63E-10 | 4.73674 | 5.07E-06 |
| **NM_006425__SLU7** | 6.634 | 5.66E-10 | -0.63956 | 0.523452 |
| **NM_013293__HSU53209** | 6.63372 | 5.67E-10 | -0.22837 | 0.819674 |
| **NM_018333__FLJ11128** | 6.6193 | 6.12E-10 | -1.39379 | 0.165485 |
| **AK001883__FLJ11021** | 6.61054 | 6.40E-10 | -1.19234 | 0.235049 |
| **NM_005694__COX17** | 6.59652 | 6.89E-10 | 1.43465 | 0.153511 |
| **NM_017922__FLJ20666** | 6.59132 | 7.08E-10 | -2.32124 | 0.021648 |
| **AK001758__LOC57187** | 6.58506 | 7.31E-10 | -0.97819 | 0.329585 |
| **Contig36013_RC** | 6.58379 | 7.36E-10 | -0.52259 | 0.602049 |
| **NM_013448__BAZ1A** | 6.57691 | 7.63E-10 | -0.95552 | 0.340886 |
| **NM_014877__KIAA0054** | 6.57574 | 7.68E-10 | -0.56615 | 0.572157 |
| **Contig36843_RC** | 6.56893 | 7.95E-10 | -3.08569 | 0.002427 |
| **NM_000489__ATRX** | 6.56881 | 7.96E-10 | -1.16735 | 0.244959 |
| **NM_017957__FLJ20778** | 6.56781 | 8.00E-10 | 1.99849 | 0.047508 |
| **AL161991__DKFZP761C169** | 6.56757 | 8.01E-10 | -2.06806 | 0.040387 |
| **NM_002892__RBBP1** | 6.56563 | 8.09E-10 | -1.28338 | 0.201378 |
| **Contig50355_RC** | 6.56306 | 8.20E-10 | -3.61985 | 0.000405 |
| **Contig37262** | 6.55572 | 8.52E-10 | 0.058932 | 0.953086 |
| **NM_004607__TBCA** | 6.55454 | 8.57E-10 | 0.858823 | 0.391836 |
| **Contig12742_RC** | 6.55283 | 8.65E-10 | 1.88525 | 0.06137 |
| **NM_015046__KIAA0625** | 6.54959 | 8.79E-10 | -1.45539 | 0.147694 |
| **NM_006520__TCTE1L** | 6.54887 | 8.83E-10 | -2.05358 | 0.041789 |
| **Contig45397_RC** | 6.54429 | 9.04E-10 | -2.30623 | 0.022496 |
| **NM_003859__DPM1** | 6.5053 | 1.11E-09 | 0.837658 | 0.403583 |
| **NM_004346__CASP3** | 6.50469 | 1.11E-09 | 0.284213 | 0.776647 |
| **NM_006554__MTX2** | 6.49519 | 1.17E-09 | -0.12364 | 0.901773 |
| **NM_004373__COX6A1** | 6.4927 | 1.18E-09 | 3.40183 | 0.000862 |
| **Contig54534_RC** | 6.47376 | 1.30E-09 | -4.43452 | 1.80E-05 |
| **NM_007054__KIF3A** | 6.46496 | 1.36E-09 | -1.52541 | 0.129305 |
| **Contig55789_RC__RPL6** | 6.45743 | 1.42E-09 | -0.96002 | 0.33862 |
| **AL117608__DKFZP564O1863** | 6.44945 | 1.48E-09 | -1.57841 | 0.116621 |
| **NM_017847__FLJ20505** | 6.43939 | 1.56E-09 | -0.90326 | 0.367866 |
| **AL133577** | 6.42348 | 1.69E-09 | -1.1022 | 0.272176 |
| **Contig48000_RC__ABCA5** | 6.42242 | 1.70E-09 | -2.93268 | 0.003899 |
| **AB014566__KIAA0666** | 6.42069 | 1.71E-09 | -0.45877 | 0.647074 |
| **NM_005385__NKTR** | 6.41456 | 1.77E-09 | -2.73492 | 0.007008 |
| **NM_007373__SHOC2** | 6.41367 | 1.78E-09 | -1.59962 | 0.11183 |
| **NM_003537__H3FL** | 6.40985 | 1.81E-09 | 3.3373 | 0.001072 |
| **X98260__ZRF1** | 6.40791 | 1.83E-09 | -1.50534 | 0.134382 |
| **NM_006603__STAG2** | 6.40369 | 1.87E-09 | -1.68444 | 0.094219 |
| **Contig36140_RC** | 6.40203 | 1.89E-09 | -2.2686 | 0.024749 |
| **Contig29349_RC** | 6.39747 | 1.93E-09 | -0.33036 | 0.741596 |
| **AF040964** | 6.39277 | 1.98E-09 | -1.57235 | 0.118019 |
| **NM_018098__FLJ10461** | 6.39188 | 1.99E-09 | 1.78054 | 0.077053 |
| **NM_018046__FLJ10283** | 6.37903 | 2.12E-09 | -1.88992 | 0.060737 |
| **Contig21130_RC** | 6.37845 | 2.13E-09 | -1.63039 | 0.10516 |
| **Contig31986_RC** | 6.37614 | 2.15E-09 | 0.022731 | 0.981896 |
| **AB037799__KIAA1378** | 6.37059 | 2.22E-09 | 0.036953 | 0.970572 |
| **NM_013303__HSU84971** | 6.35424 | 2.41E-09 | 2.39757 | 0.01776 |
| **NM_016360__LOC51204** | 6.34772 | 2.49E-09 | 4.43953 | 1.76E-05 |
| **Contig5455_RC** | 6.34685 | 2.50E-09 | 0.895107 | 0.372193 |
| **NM_014684__KIAA0373** | 6.34592 | 2.51E-09 | -0.83209 | 0.406709 |
| **Contig31165_RC** | 6.33777 | 2.62E-09 | -1.72464 | 0.086694 |
| **Contig55474_RC** | 6.33517 | 2.66E-09 | 2.35394 | 0.019899 |
| **NM_003642__HAT1** | 6.33345 | 2.68E-09 | -1.39936 | 0.163812 |
| **Contig51208__NEDD1** | 6.33021 | 2.72E-09 | -0.60866 | 0.543687 |
| **AL133611** | 6.32871 | 2.74E-09 | -1.35298 | 0.178139 |
| **NM_014733__KIAA0305** | 6.31947 | 2.88E-09 | -3.3711 | 0.000957 |
| **Contig41498_RC** | 6.31758 | 2.90E-09 | -0.31154 | 0.755829 |
| **Contig56737_RC** | 6.31663 | 2.92E-09 | 0.090929 | 0.927672 |
| **NM_004707__APG12L** | 6.31361 | 2.96E-09 | 3.00768 | 0.003098 |
| **NM_014373__GPCR150** | 6.30858 | 3.04E-09 | -0.88182 | 0.379312 |
| **NM_005324__H3F3B** | 6.29284 | 3.30E-09 | 1.58964 | 0.114064 |
| **AB018256__KIAA0713** | 6.28389 | 3.45E-09 | -2.61413 | 0.009877 |
| **Contig898_RC** | 6.2786 | 3.54E-09 | -4.52221 | 1.25E-05 |
| **NM_015888__HOOK1** | 6.26937 | 3.71E-09 | -0.17601 | 0.860531 |
| **Contig52570_RC** | 6.26931 | 3.71E-09 | -4.44082 | 1.75E-05 |
| **NM_016122__LOC51134** | 6.26806 | 3.74E-09 | -1.81036 | 0.072282 |
| **NM_018314__FLJ11068** | 6.25308 | 4.03E-09 | 1.24877 | 0.213734 |
| **AL079292__LOC54505** | 6.25144 | 4.07E-09 | -1.11316 | 0.267457 |
| **AB037829__RENT2** | 6.2422 | 4.26E-09 | 0.162499 | 0.871136 |
| **NM_003563__SPOP** | 6.23178 | 4.49E-09 | 0.981257 | 0.328078 |
| **Contig36530_RC** | 6.23112 | 4.51E-09 | -0.39724 | 0.691766 |
| **AB018319__KIAA0776** | 6.22907 | 4.55E-09 | -2.13131 | 0.034727 |
| **Contig55353_RC** | 6.22555 | 4.64E-09 | -4.69052 | 6.17E-06 |
| **AB011118__KIAA0546** | 6.22279 | 4.70E-09 | -2.76391 | 0.006443 |
| **NM_007276__CBX3** | 6.21997 | 4.77E-09 | -1.96831 | 0.050914 |
| **NM_005445__CSPG6** | 6.20918 | 5.04E-09 | -1.0639 | 0.289119 |
| **NM_014827__KIAA0663** | 6.19371 | 5.45E-09 | -1.69622 | 0.091961 |
| **Contig42174** | 6.18992 | 5.55E-09 | -2.37376 | 0.0189 |
| **Contig45024_RC** | 6.17831 | 5.89E-09 | 0.902813 | 0.368102 |
| **AB011102__KIAA0530** | 6.17825 | 5.89E-09 | -1.76068 | 0.080372 |
| **NM_003142__SSB** | 6.17273 | 6.05E-09 | -1.1365 | 0.257597 |
| **NM_004236__TRIP15** | 6.1551 | 6.62E-09 | 0.095415 | 0.924115 |
| **Contig55214_RC__KIAA1565** | 6.15099 | 6.75E-09 | -3.23299 | 0.001513 |
| **NM_003620__PPM1D** | 6.14655 | 6.91E-09 | 1.37292 | 0.171868 |
| **NM_006356__ATP5JD** | 6.14453 | 6.98E-09 | 4.7419 | 4.96E-06 |
| **Contig43376_RC** | 6.14363 | 7.01E-09 | -1.20433 | 0.230398 |
| **Contig56848_RC** | 6.13262 | 7.41E-09 | -1.50237 | 0.135147 |
| **NM_005486__TOM1L1** | 6.13029 | 7.49E-09 | 0.551481 | 0.582141 |
| **NM_016106__KIAA0917** | 6.12637 | 7.64E-09 | -2.34918 | 0.020146 |
| **X78926__ZNF268** | 6.10994 | 8.30E-09 | -1.62345 | 0.106636 |
| **AI669022_RC** | 6.10619 | 8.46E-09 | -2.22803 | 0.027397 |
| **AF078164** | 6.1005 | 8.70E-09 | 0.529127 | 0.597515 |
| **X68060__TOP2B** | 6.09674 | 8.87E-09 | -1.21121 | 0.227758 |
| **NM_004902__CC1.3** | 6.09503 | 8.94E-09 | -2.43457 | 0.016108 |
| **AI205537_RC** | 6.09263 | 9.05E-09 | -1.3553 | 0.177401 |
| **D26069__KIAA0041** | 6.09033 | 9.15E-09 | -2.21997 | 0.027952 |
| **NM_005034__POLR2K** | 6.08638 | 9.34E-09 | 1.27887 | 0.202958 |
| **NM_016097__LOC51124** | 6.08627 | 9.34E-09 | -2.04144 | 0.042996 |
| **X15183__HSPCA** | 6.08314 | 9.49E-09 | 1.44693 | 0.150046 |
| **NM_016001__LOC51096** | 6.07776 | 9.75E-09 | 2.11369 | 0.03623 |
| **Contig53866_RC** | 6.06668 | 1.03E-08 | -2.56553 | 0.011303 |
| **Contig51328_RC** | 6.0657 | 1.04E-08 | -1.84548 | 0.06698 |
| **AJ002572__N143** | 6.06115 | 1.06E-08 | -1.34179 | 0.181733 |
| **AL117611** | 6.05677 | 1.08E-08 | -2.43459 | 0.016107 |
| **NM_004876__ZNF254** | 6.05662 | 1.08E-08 | -1.34029 | 0.182219 |
| **NM_012433__SF3B1** | 6.05434 | 1.10E-08 | -2.33282 | 0.021014 |
| **NM_003372__VBP1** | 6.04935 | 1.12E-08 | 1.52482 | 0.129453 |
| **NM_015934__NOP5/NOP58** | 6.04925 | 1.12E-08 | 0.505391 | 0.614041 |
| **NM_018149__FLJ10587** | 6.04119 | 1.17E-08 | 2.18773 | 0.030269 |
| **AL161968__APG12L** | 6.03714 | 1.19E-08 | -1.4176 | 0.158424 |
| **AL050171__DKFZP586F1122** | 6.03577 | 1.20E-08 | -3.05162 | 0.002702 |
| **Contig52881_RC** | 6.02938 | 1.24E-08 | -2.97645 | 0.003411 |
| **AL050050__DKFZP566D133** | 6.02574 | 1.26E-08 | -1.87414 | 0.062896 |
| **NM_005509__DMXL1** | 6.01584 | 1.33E-08 | -3.0442 | 0.002765 |
| **NM_020189__DC6** | 6.01021 | 1.36E-08 | 3.44751 | 0.000738 |
| **NM_014635__KIAA0336** | 6.00358 | 1.41E-08 | -4.47325 | 1.53E-05 |
| **NM_016058__LOC51002** | 6.0021 | 1.42E-08 | -0.15881 | 0.874037 |
| **X69804__SSB** | 5.99782 | 1.45E-08 | -1.56589 | 0.119525 |
| **Contig64688** | 5.98285 | 1.56E-08 | 3.38893 | 0.000901 |
| **Contig46258_RC** | 5.98138 | 1.57E-08 | -2.37655 | 0.018763 |
| **NM_006830__UQCR** | 5.97724 | 1.61E-08 | 0.747294 | 0.45608 |
| **NM_002913__RFC1** | 5.96734 | 1.69E-08 | -1.42625 | 0.155916 |
| **NM_003136__SRP54** | 5.967 | 1.69E-08 | 0.434898 | 0.664274 |
| **NM_017635__FLJ20039** | 5.96581 | 1.70E-08 | -1.24714 | 0.21433 |
| **AK000703__AK000703** | 5.96252 | 1.73E-08 | -1.46802 | 0.144235 |
| **NM_016093__LOC51121** | 5.95978 | 1.75E-08 | 3.21825 | 0.001587 |
| **Contig41420_RC** | 5.9561 | 1.78E-08 | -2.2089 | 0.028729 |
| **Contig575_RC** | 5.95438 | 1.80E-08 | -2.07283 | 0.039934 |
| **NM_016587__HECH** | 5.95426 | 1.80E-08 | -1.87865 | 0.062272 |
| **Contig35294_RC** | 5.95038 | 1.83E-08 | 3.38811 | 0.000903 |
| **AF161434** | 5.93751 | 1.95E-08 | 1.79512 | 0.074689 |
| **NM_018387__FLJ11307** | 5.93029 | 2.03E-08 | -0.2587 | 0.796227 |
| **Contig53048_RC** | 5.92818 | 2.05E-08 | -0.47954 | 0.632269 |
| **Contig55103_RC__DKFZp761B2423** | 5.92781 | 2.05E-08 | -1.18885 | 0.236416 |
| **NM_018061__FLJ10330** | 5.92242 | 2.11E-08 | -0.26685 | 0.789959 |
| **AL049998** | 5.91482 | 2.19E-08 | -2.51054 | 0.013137 |
| **AB018257__ZNF294** | 5.90545 | 2.29E-08 | -1.42941 | 0.155008 |
| **Contig39872_RC** | 5.89954 | 2.36E-08 | 0.41901 | 0.67582 |
| **NM_002495__NDUFS4** | 5.88532 | 2.53E-08 | 1.80963 | 0.072396 |
| **NM_014167__HSPC128** | 5.87873 | 2.61E-08 | 1.62546 | 0.106207 |
| **AB033079__KIAA1253** | 5.87701 | 2.63E-08 | -2.17309 | 0.031375 |
| **NM_014875__KIAA0042** | 5.87609 | 2.64E-08 | 2.99885 | 0.003183 |
| **NM_006256__PRKCL2** | 5.87179 | 2.70E-08 | -0.82302 | 0.41183 |
| **Contig43586_RC** | 5.86879 | 2.74E-08 | 1.183 | 0.238719 |
| **NM_018353__FLJ11186** | 5.86786 | 2.75E-08 | -0.65732 | 0.512002 |
| **AL050018__DKFZP564B116** | 5.86469 | 2.80E-08 | -2.90833 | 0.004198 |
| **Contig33708_RC** | 5.85889 | 2.88E-08 | 0.136282 | 0.891785 |
| **NM_006304__DSS1** | 5.85871 | 2.88E-08 | 2.04981 | 0.04216 |
| **AL117418__DKFZp564G2263** | 5.85775 | 2.89E-08 | -2.81622 | 0.005527 |
| **AB020681__KIAA0874** | 5.85627 | 2.91E-08 | -3.10853 | 0.002258 |
| **NM_003400__XPO1** | 5.85626 | 2.91E-08 | -0.49977 | 0.617986 |
| **NM_007269__STXBP3** | 5.85512 | 2.93E-08 | -2.73327 | 0.007041 |
| **NM_018509__PRO1855** | 5.85081 | 2.99E-08 | 3.25227 | 0.00142 |
| **AK002177__WDR9** | 5.84615 | 3.06E-08 | -1.68209 | 0.094675 |
| **NM_006421__BIG1** | 5.84426 | 3.09E-08 | -1.28751 | 0.19994 |
| **NM_018204__CKAP2** | 5.83867 | 3.17E-08 | 1.36596 | 0.174038 |
| **NM_003908__EIF2S2** | 5.83556 | 3.22E-08 | 2.50318 | 0.013402 |
| **Contig57091_RC__ITM2B** | 5.83539 | 3.23E-08 | -4.11163 | 6.51E-05 |
| **NM_003292__TPR** | 5.82784 | 3.35E-08 | -1.96736 | 0.051024 |
| **Contig3164_RC** | 5.82727 | 3.36E-08 | -0.34795 | 0.728379 |
| **Contig43506_RC** | 5.8232 | 3.42E-08 | -0.7181 | 0.473833 |
| **NM_003510__H2AFD** | 5.82104 | 3.46E-08 | 3.19018 | 0.001738 |
| **Contig19770_RC** | 5.82101 | 3.46E-08 | -1.4236 | 0.156681 |
| **NM_003794__SNX4** | 5.819 | 3.49E-08 | 0.547571 | 0.584817 |
| **AL050199** | 5.81681 | 3.53E-08 | -1.52239 | 0.13006 |
| **Contig32846_RC** | 5.8164 | 3.54E-08 | 0.471249 | 0.638162 |
| **NM_018136__FLJ10549** | 5.80978 | 3.65E-08 | 2.23889 | 0.026665 |
| **Contig39591_RC** | 5.79517 | 3.92E-08 | -1.57097 | 0.11834 |
| **NM_002512__NME2** | 5.7835 | 4.15E-08 | 2.90305 | 0.004265 |
| **Contig33271_RC** | 5.77686 | 4.29E-08 | -2.81867 | 0.005487 |
| **NM_007217__PDCD10** | 5.77628 | 4.30E-08 | 0.823618 | 0.411491 |
| **Contig48919_RC__RAI** | 5.76044 | 4.64E-08 | 5.21714 | 6.07E-07 |
| **Contig51442_RC** | 5.75606 | 4.74E-08 | -2.29882 | 0.022924 |
| **NM_018167__FLJ10648** | 5.75293 | 4.82E-08 | -2.01968 | 0.045234 |
| **NM_005196__CENPF** | 5.74982 | 4.89E-08 | 3.17233 | 0.001841 |
| **AF084530__DMP1** | 5.74565 | 4.99E-08 | -3.43527 | 0.00077 |
| **NM_004735__LRRFIP1** | 5.74232 | 5.07E-08 | -0.38638 | 0.699775 |
| **AL137349__DKFZP434A0225** | 5.7401 | 5.12E-08 | -1.61 | 0.109543 |
| **Contig45011_RC** | 5.73838 | 5.17E-08 | -0.78931 | 0.431203 |
| **NM_003657__BCAS1** | 5.72947 | 5.39E-08 | 0.057429 | 0.954282 |
| **AK001838** | 5.72736 | 5.45E-08 | -0.72637 | 0.468769 |
| **NM_016395__HSPC121** | 5.72159 | 5.60E-08 | 1.11827 | 0.265276 |
| **Contig33343_RC** | 5.72071 | 5.63E-08 | -0.31419 | 0.75382 |
| **AF117236__MATR3** | 5.71768 | 5.71E-08 | -2.93276 | 0.003898 |
| **Contig57230_RC** | 5.7141 | 5.81E-08 | -2.06391 | 0.040785 |
| **NM_002822__PTK9** | 5.7098 | 5.93E-08 | -2.81724 | 0.00551 |
| **NM_014055__CDV-1** | 5.70829 | 5.98E-08 | -1.81951 | 0.070868 |
| **Contig45388_RC** | 5.70653 | 6.03E-08 | -3.05201 | 0.002698 |
| **NM_016359__LOC51203** | 5.70586 | 6.05E-08 | 3.39605 | 0.000879 |
| **Contig52623_RC** | 5.69907 | 6.25E-08 | 1.00275 | 0.317629 |
| **NM_016073__CGI-142** | 5.69901 | 6.25E-08 | -3.12319 | 0.002155 |
| **Contig57041_RC** | 5.69887 | 6.25E-08 | -1.98835 | 0.04863 |
| **AF052100** | 5.69769 | 6.29E-08 | -3.87487 | 0.00016 |
| **NM_006333__C1D** | 5.69405 | 6.40E-08 | -0.93542 | 0.351109 |
| **NM_003094__SNRPE** | 5.69168 | 6.47E-08 | 2.72338 | 0.007245 |
| **Contig34947_RC** | 5.68936 | 6.55E-08 | -1.99978 | 0.047367 |
| **Contig53962_RC** | 5.68237 | 6.77E-08 | -3.60337 | 0.000429 |
| **Contig44211_RC** | 5.67393 | 7.05E-08 | -1.90641 | 0.058549 |
| **Contig41392_RC** | 5.6711 | 7.15E-08 | -3.33054 | 0.001096 |
| **Contig26590_RC** | 5.66751 | 7.27E-08 | -1.50608 | 0.134192 |
| **NM_018844__BAP29** | 5.65006 | 7.90E-08 | -0.34552 | 0.730196 |
| **NM_005828__HAN11** | 5.64909 | 7.94E-08 | 5.50942 | 1.57E-07 |
| **Contig44289_RC** | 5.6486 | 7.96E-08 | 3.19537 | 0.001709 |
| **Contig39364_RC** | 5.63983 | 8.30E-08 | -1.25626 | 0.211015 |
| **AB014547__MTMR4** | 5.63535 | 8.48E-08 | 1.67208 | 0.096636 |
| **NM_004748__CPR8** | 5.6348 | 8.50E-08 | -3.04347 | 0.002771 |
| **Contig23751_RC** | 5.63428 | 8.52E-08 | -0.04881 | 0.96114 |
| **Contig47578_RC** | 5.63423 | 8.53E-08 | 1.00661 | 0.315776 |
| **NM_018123__FLJ10517** | 5.62722 | 8.82E-08 | 2.18777 | 0.030266 |
| **NM_016340__LOC51735** | 5.62477 | 8.92E-08 | -0.55986 | 0.576425 |
| **NM_005836__UK114** | 5.61871 | 9.18E-08 | -0.29194 | 0.770741 |
| **NM_018032__FLJ10231** | 5.61461 | 9.36E-08 | -0.61697 | 0.538211 |
| **AK001627__CPSF2** | 5.60449 | 9.83E-08 | 0.573246 | 0.567354 |
| **Contig48166_RC** | 5.60265 | 9.91E-08 | -0.57921 | 0.563337 |
| **NM_014264__STK18** | 5.60222 | 9.93E-08 | 2.52008 | 0.012801 |
| **Contig41194_RC** | 5.6004 | 1.00E-07 | 0.321061 | 0.74862 |
| **Contig42430_RC** | 5.5994 | 1.01E-07 | -2.8795 | 0.004578 |
| **AB011139__OPA1** | 5.58951 | 1.06E-07 | -1.25751 | 0.210563 |
| **Contig51105_RC** | 5.58646 | 1.07E-07 | -3.34465 | 0.001046 |
| **NM_012097__ARL5** | 5.58587 | 1.07E-07 | -0.66541 | 0.50683 |
| **Contig52932_RC** | 5.58469 | 1.08E-07 | -1.07061 | 0.2861 |
| **Contig51158_RC** | 5.57979 | 1.11E-07 | -0.59235 | 0.554524 |
| **Contig40997_RC** | 5.57975 | 1.11E-07 | 0.274793 | 0.783861 |
| **NM_017763__FLJ20315** | 5.57845 | 1.11E-07 | 1.01408 | 0.312211 |
| **NM_007146__ZNF161** | 5.57288 | 1.14E-07 | 0.465835 | 0.642023 |
| **Contig44251_RC** | 5.57172 | 1.15E-07 | -2.99151 | 0.003256 |
| **U28831** | 5.5686 | 1.17E-07 | -1.71327 | 0.088771 |
| **AB014578__KIAA0678** | 5.55729 | 1.23E-07 | 0.54637 | 0.58564 |
| **U17989__GS2NA** | 5.55474 | 1.24E-07 | -2.1907 | 0.030049 |
| **Contig38497_RC** | 5.55353 | 1.25E-07 | 1.55823 | 0.121329 |
| **AB029001__KIAA1078** | 5.55012 | 1.27E-07 | -2.41832 | 0.016816 |
| **NM_014288__ITGB3BP** | 5.54877 | 1.28E-07 | 0.249908 | 0.803007 |
| **D87684__KIAA0242** | 5.54869 | 1.28E-07 | -2.12966 | 0.034865 |
| **Contig56434_RC** | 5.54713 | 1.29E-07 | -2.87225 | 0.004679 |
| **Contig62588_RC** | 5.54703 | 1.29E-07 | -0.76694 | 0.444345 |
| **Contig53644_RC** | 5.54451 | 1.31E-07 | -1.87337 | 0.063003 |
| **AL137315** | 5.54236 | 1.32E-07 | -1.45437 | 0.147976 |
| **NM_006937__SMT3H2** | 5.54131 | 1.33E-07 | 1.96539 | 0.051254 |
| **NM_003187__TAF2G** | 5.53902 | 1.34E-07 | 0.73686 | 0.462382 |
| **NM_002157__HSPE1** | 5.53746 | 1.35E-07 | 1.31738 | 0.189761 |
| **AB037771__KIAA1350** | 5.53178 | 1.39E-07 | -3.36441 | 0.000979 |
| **NM_016265__GIOT-3** | 5.52924 | 1.40E-07 | -2.41587 | 0.016925 |
| **NM_002354__TACSTD1** | 5.52462 | 1.44E-07 | 2.51006 | 0.013154 |
| **Contig42686** | 5.52104 | 1.46E-07 | -3.31357 | 0.00116 |
| **NM_018569__PRO0971** | 5.51902 | 1.47E-07 | -2.05815 | 0.041342 |
| **AB002306__KIAA0308** | 5.5152 | 1.50E-07 | -1.34558 | 0.18051 |
| **NM_012124__CHP1** | 5.51516 | 1.50E-07 | 0.400246 | 0.689556 |
| **NM_018471__HT010** | 5.51359 | 1.51E-07 | -0.93258 | 0.352568 |
| **Contig51153_RC** | 5.50942 | 1.54E-07 | 0.851767 | 0.395729 |
| **AB007892__CDC5L** | 5.50667 | 1.56E-07 | -0.54396 | 0.587294 |
| **NM_013322__SNX10** | 5.49867 | 1.62E-07 | 0.778144 | 0.437735 |
| **Contig44521_RC** | 5.49805 | 1.63E-07 | -0.55532 | 0.579521 |
| **NM_002789__PSMA4** | 5.49305 | 1.67E-07 | 3.05565 | 0.002668 |
| **NM_005154__USP8** | 5.49133 | 1.68E-07 | -0.4921 | 0.623386 |
| **Contig54507_RC** | 5.48478 | 1.73E-07 | -2.87423 | 0.004651 |
| **AB032969__KIAA1143** | 5.48427 | 1.74E-07 | -2.38985 | 0.018123 |
| **NM_018469__HT008** | 5.47871 | 1.78E-07 | 2.1488 | 0.033288 |
| **Contig57726_RC** | 5.47657 | 1.80E-07 | -2.54745 | 0.011879 |
| **NM_018293__FLJ10997** | 5.47528 | 1.81E-07 | 0.644085 | 0.520524 |
| **Contig1061_RC** | 5.47441 | 1.82E-07 | -1.54899 | 0.123534 |
| **Contig46452_RC** | 5.47208 | 1.84E-07 | -2.62195 | 0.009663 |
| **NM_002358__MAD2L1** | 5.45706 | 1.97E-07 | 4.59826 | 9.11E-06 |
| **M73547__D5S346** | 5.45563 | 1.99E-07 | -3.70468 | 0.000299 |
| **NM_002137__HNRPA2B1** | 5.45273 | 2.01E-07 | 1.45845 | 0.14685 |
| **NM_017785__FLJ20364** | 5.45204 | 2.02E-07 | 2.66858 | 0.008473 |
| **NM_014829__KIAA0801** | 5.44597 | 2.08E-07 | -0.97876 | 0.329309 |
| **AL117599__CLCN3** | 5.44559 | 2.08E-07 | -0.54607 | 0.585847 |
| **AK000325__AK000325** | 5.44001 | 2.14E-07 | -0.36003 | 0.719341 |
| **AL137398** | 5.43663 | 2.17E-07 | -4.24517 | 3.85E-05 |
| **NM_004582__RABGGTB** | 5.4351 | 2.19E-07 | 0.048984 | 0.960998 |
| **Contig53629_RC** | 5.434 | 2.20E-07 | -3.18956 | 0.001742 |
| **Contig43746_RC** | 5.43283 | 2.21E-07 | -3.35879 | 0.000997 |
| **Contig39446_RC** | 5.42921 | 2.25E-07 | -1.44159 | 0.151545 |
| **NM_006544__SEC10L1** | 5.42913 | 2.25E-07 | 1.02383 | 0.307597 |
| **NM_002453__MTIF2** | 5.4264 | 2.28E-07 | 2.56609 | 0.011286 |
| **Contig42880_RC** | 5.42384 | 2.31E-07 | -1.23911 | 0.21728 |
| **Contig41590** | 5.41572 | 2.39E-07 | -3.62567 | 0.000397 |
| **Contig11415_RC** | 5.41107 | 2.45E-07 | -1.81871 | 0.070991 |
| **NM_007145__ZNF146** | 5.40929 | 2.47E-07 | -1.1696 | 0.244055 |
| **AF201947__MP1** | 5.4061 | 2.50E-07 | 0.16293 | 0.870797 |
| **Contig30357_RC** | 5.40542 | 2.51E-07 | -0.50084 | 0.617234 |
| **NM_016052__LOC51018** | 5.40365 | 2.53E-07 | 0.957389 | 0.339943 |
| **Contig32689_RC** | 5.40205 | 2.55E-07 | -2.27178 | 0.024551 |
| **Contig55971_RC** | 5.40084 | 2.57E-07 | -1.14499 | 0.254074 |
| **Contig54664_RC** | 5.39811 | 2.60E-07 | -0.65159 | 0.515685 |
| **NM_017845__FLJ20502** | 5.38977 | 2.70E-07 | -1.11935 | 0.264817 |
| **NM_016475__HSPC213** | 5.38824 | 2.72E-07 | 0.89412 | 0.372719 |
| **Contig50462_RC__SENP7** | 5.38215 | 2.80E-07 | -3.13416 | 0.002081 |
| **AL110163** | 5.38184 | 2.80E-07 | -2.11738 | 0.035911 |
| **Contig36900_RC** | 5.38164 | 2.81E-07 | -3.89423 | 0.000149 |
| **Contig33385_RC** | 5.37899 | 2.84E-07 | -1.62265 | 0.106807 |
| **NM_004757__SCYE1** | 5.37761 | 2.86E-07 | 1.39461 | 0.165237 |
| **NM_006444__CAP-E** | 5.37597 | 2.88E-07 | 0.658191 | 0.511445 |
| **NM_012115__CASP8AP2** | 5.37345 | 2.92E-07 | 0.907542 | 0.365606 |
| **NM_017743__FLJ20283** | 5.36519 | 3.03E-07 | -1.09672 | 0.274557 |
| **Contig32899_RC** | 5.36176 | 3.08E-07 | -1.89929 | 0.059485 |
| **Contig431_RC** | 5.36028 | 3.10E-07 | 1.76422 | 0.079772 |
| **Contig22386_RC** | 5.35428 | 3.19E-07 | -2.96271 | 0.003558 |
| **NM_006886__ATP5E** | 5.35386 | 3.19E-07 | 0.058281 | 0.953604 |
| **AF132362__HNRPH3** | 5.35013 | 3.25E-07 | -1.81717 | 0.071228 |
| **NM_015384__IDN3** | 5.34968 | 3.26E-07 | -1.4017 | 0.163113 |
| **NM_005999__TSNAX** | 5.3493 | 3.26E-07 | -1.82396 | 0.070189 |
| **AB040921__KIAA1488** | 5.34757 | 3.29E-07 | -0.22936 | 0.818909 |
| **Contig54609_RC** | 5.34263 | 3.36E-07 | -1.77418 | 0.078104 |
| **AW293856_RC** | 5.34147 | 3.38E-07 | 1.30831 | 0.19281 |
| **NM_007215__POLG2** | 5.34119 | 3.39E-07 | 2.74191 | 0.006867 |
| **AL080066__RPS6** | 5.34095 | 3.39E-07 | 1.55196 | 0.122822 |
| **AB002448** | 5.33944 | 3.41E-07 | -2.18208 | 0.030692 |
| **Contig39787_RC** | 5.33855 | 3.43E-07 | -0.33484 | 0.738226 |
| **Contig28888_RC** | 5.338 | 3.44E-07 | 3.95718 | 0.000118 |
| **AB002449** | 5.33705 | 3.45E-07 | -0.99289 | 0.322396 |
| **NM_006807__CBX1** | 5.3353 | 3.48E-07 | 1.55751 | 0.1215 |
| **Contig1351_RC** | 5.33369 | 3.51E-07 | -3.36544 | 0.000975 |
| **Contig37887_RC** | 5.33308 | 3.52E-07 | 2.56426 | 0.011343 |
| **Contig57610_RC** | 5.33122 | 3.55E-07 | -0.80881 | 0.419934 |
| **X76302__RY1** | 5.32899 | 3.58E-07 | 0.27538 | 0.783411 |
| **Contig50120_RC** | 5.32887 | 3.58E-07 | 2.88833 | 0.004459 |
| **NM_003077__SMARCD2** | 5.32865 | 3.59E-07 | 6.40278 | 1.93E-09 |
| **NM_003533__H3FF** | 5.32851 | 3.59E-07 | 3.47822 | 0.000664 |
| **NM_002703__PPAT** | 5.32653 | 3.62E-07 | 2.94662 | 0.003737 |
| **NM_018261__FLJ10893** | 5.32455 | 3.66E-07 | -2.14331 | 0.033734 |
| **AF160213__LOC56889** | 5.31591 | 3.81E-07 | -0.80307 | 0.42323 |
| **NM_002106__H2AFZ** | 5.31314 | 3.85E-07 | 5.01715 | 1.49E-06 |

**Table B. Pathways positively correlated to S6K1.**

| p-value | Term | Term ID | term description | Genes |
| --- | --- | --- | --- | --- |
|  |  |  |  |  |
| 4.86e-12 | GO:0044260 | BP | cellular macromolecule metabolic process | ATRX, BAZ1A, BAZ2B, BLZF1, C1D, CASP3, CASP8AP2, CBX1, CBX3, CCNE2, CDC5L, CENPE, CENPF, CHD1, CHP1, CKAP2, CLOCK, CLTC, CPSF2, DPM1, EIF2S2, FKBP3, H2AFZ, HAT1, HNRPDL, HSPE1, HSU53209, HTATSF1, ITGB3BP, ITM2B, LRRFIP1, MAD2L1, MNAT1, MPHOSPH10, MTIF2, MTMR4, MTX2, NDUFS4, NKTR, NP220, PDCD10, PIK3CA, PNN, POLR2K, PPM1D, PRKAR1A, PSMA3, PSMA4, PSMC5, PSMD12, RABGGTB, RAD51C, RFC1, RPL6, RPS6, RPS6KB1, SF3B1, SLU7, SMARCA5, SMARCD2, SNRPB2, SNRPE, SPOP, SRP54, SSB, STAG2, TAX1BP1, TBCA, TBK1, TLK2, TMF1, TOB1, TOM1L1, TOP2B, TPR, TRIP11, UK114, USP16, USP6, USP8, VBP1, WDR9, XPO1, ZNF146, ZNF254, ZNF91 |
| 2.38e-03 | GO:0046907 | BP | intracellular transport | APPBP2, ATP5E, BLZF1, CHP1, CLTC, CPSF2, GOLGA4, HOOK1, KIF3A, KIF5B, MTX2, RPL6, RPS6, SCP2, SLU7, SNX4, SRP54, TLOC1, TOB1, TOM1L1, TPR, TRIP11, XPO1 |
| 2.26e-02 | GO:0071013 | CC | catalytic step 2 spliceosome | CDC5L, PNN, SF3B1, SLU7, SNRPB2, SNRPE |
| 1.22e-14 | GO:0044424 | CC | intracellular part | ABCA5, APPBP2, ATP5E, ATRX, BAZ1A, BAZ2B, BCAS1, BLZF1, C1D, CASP3, CASP8AP2, CBX1, CBX3, CCNE2, CD2AP, CDC5L, CDS1, CENPC1, CENPE, CENPF, CETN3, CHD1, CHP1, CKAP2, CLCN3, CLOCK, CLTC, COIL, COX17, COX6A1, CPSF2, DMP1, DPM1, EIF2S2, FKBP3, GOLGA4, GOLGB1, H2AFZ, HAT1, HNRPDL, HOOK1, HSPE1, HSU53209, HTATSF1, ITGB3BP, ITM2B, KIF3A, KIF5B, KTN1, LRRFIP1, MAD2L1, MATR3, MNAT1, MPHOSPH10, MTIF2, MTMR4, MTX2, NDUFA5, NDUFS4, NEDD1, NP220, OXR1, PDCD10, PIK3CA, PNN, POLR2K, PPAT, PPM1D, PRKAR1A, PSMA3, PSMA4, PSMC5, PSMD12, RAD51C, RFC1, RPL6, RPS6, RPS6KB1, SCP2, SENP7, SF3B1, SHOC2, SLU7, SMARCA5, SMARCD2, SNRPB2, SNRPE, SNX10, SNX4, SPOP, SRP54, SSB, STAG2, STXBP3, TAX1BP1, TBCA, TBK1, TCTE1L, TLK2, TLOC1, TMF1, TOB1, TOM1L1, TOP2B, TPR, TRIP11, TSNAX, UK114, USP16, USP6, USP8, VBP1, WDR9, XPO1, ZNF146, ZNF254, ZNF91 |
| 1.36e-05 | GO:0005515 | MF | protein binding | APPBP2, ATRX, BAZ1A, BAZ2B, BLZF1, C1D, CASP3, CASP8AP2, CBX1, CBX3, CCNE2, CD2AP, CDC5L, CENPE, CENPF, CETN3, CHD1, CHP1, CLCN3, CLOCK, CLTC, COIL, CPSF2, DMP1, DPM1, EIF2S2, GOLGA4, GOLGB1, H2AFZ, HAT1, HOOK1, HSPE1, ITGB3BP, ITM2B, KIF3A, KIF5B, KTN1, LRRFIP1, MAD2L1, MATR3, MNAT1, MPHOSPH10, PDCD10, PIK3CA, PPM1D, PRKAR1A, PSMA3, PSMA4, PSMC5, RABGGTB, RAD51C, RFC1, RPS6, RPS6KB1, SCP2, SHOC2, SMARCA5, SNRPB2, SNRPE, SNX4, SPOP, STAG2, STXBP3, TAX1BP1, TBCA, TBK1, TCTE1L, TLK2, TMF1, TOB1, TOM1L1, TOP2B, TPR, TRIP11, USP16, USP6, USP8, VBP1, XPO1 |
| 2.33e-02 | GO:0003723 | MF | RNA binding | C1D, CDC5L, CPSF2, EIF2S2, HNRPDL, HSU53209, HTATSF1, LRRFIP1, MATR3, MTIF2, NP220, RPL6, SLU7, SNRPB2, SNRPE, SRP54, SSB, XPO1 |
| 4.95e-12 | BIOGRID:00000 | bi | BioGRID interaction data | APPBP2, ATP5E, ATRX, BAZ1A, BAZ2B, BCAS1, BLZF1, C1D, CASP3, CASP8AP2, CBX1, CBX3, CCNE2, CD2AP, CDC5L, CDS1, CENPC1, CENPE, CENPF, CETN3, CHD1, CHP1, CKAP2, CLCN3, CLOCK, CLTC, COIL, COX17, COX6A1, CPSF2 |
| 5.00e-02 | CORUM:94 | co | ATP-utilizing chromatin assembly and remodeling factor (hACF) complex | BAZ1A, SMARCA5 |
| 5.00e-02 | CORUM:925 | co | WCRF complex | BAZ1A, SMARCA5 |
| 4.29e-02 | KEGG:05016 | ke | Huntington's disease | ATP5E, CASP3, CLTC, COX6A1, NDUFA5, NDUFS4, POLR2K |
| 2.03e-02 | MI:hsa-miR-144 | mi | MI:hsa-miR-144 | BAZ1A, HAT1, HSU53209, ITGB3BP, MPHOSPH10, MTX2, OXR1, PSMA3, RABGGTB, RAD51C, SF3B1, SMARCA5, SNRPE, USP16, ZNF254 |
| 1.86e-02 | MI:hsa-miR-548d-3p | mi | MI:hsa-miR-548d-3p | BAZ1A, CBX3, CETN3, CKAP2, CLCN3, CLTC, COIL, H2AFZ, HSU53209, KTN1, MAD2L1, PPM1D, RABGGTB, SRP54, TBCA, TOP2B, USP16 |
| 9.41e-03 | MI:hsa-miR-340 | mi | MI:hsa-miR-340 | C1D, CENPC1, CETN3, HSPE1, MAD2L1, MPHOSPH10, MTIF2, MTX2, NP220, POLR2K, SSB, TOM1L1, USP16 |
| 2.42e-02 | MI:hsa-miR-513-3p | mi | MI:hsa-miR-513-3p | C1D, CASP3, CCNE2, FKBP3, H2AFZ, MATR3, MTX2, NP220, SMARCA5, SNRPB2, SNX4, SSB, STAG2, TOP2B |
| 2.46e-05 | MI:hsa-miR-590-3p | mi | MI:hsa-miR-590-3p | BAZ1A, CETN3, CKAP2, DPM1, FKBP3, ITGB3BP, MNAT1, MPHOSPH10, MTX2, NEDD1, NP220, OXR1, PSMA4, SCP2, SRP54, STAG2, TOB1, TPR, TSNAX |
| 9.77e-04 | MI:hsa-miR-548c-3p | mi | MI:hsa-miR-548c-3p | CBX3, CENPC1, COIL, DMP1, GOLGA4, GOLGB1, H2AFZ, MATR3, MTX2, NP220, PPM1D, SF3B1, SPOP, SRP54, STAG2, TOB1, TOP2B, USP16 |
| 6.44e-04 | MI:hsa-miR-656 | mi | MI:hsa-miR-656 | C1D, CENPC1, CENPF, CETN3, CKAP2, HAT1, HTATSF1, ITM2B, KTN1, MNAT1, MTX2, NEDD1, SSB, UK114 |
| 4.22e-02 | MI:mmu-miR-465b-5p | mi | MI:mmu-miR-465b-5p | C1D, CASP8AP2, CBX3, DPM1, MTX2, PNN, PSMA3, SF3B1, SMARCA5, SSB, STXBP3, TAX1BP1, USP16, USP8 |
| 3.54e-02 | MI:mmu-miR-465a-5p | mi | MI:mmu-miR-465a-5p | BAZ2B, C1D, CASP8AP2, CBX3, H2AFZ, MAD2L1, MTX2, PNN, PSMA3, SMARCA5, SSB, STXBP3, TRIP11, USP16 |
| 4.98e-02 | REAC:165726 | re | Phosphorylation of ribosomal protein S6 by activated S6K1 | RPS6, RPS6KB1 |
| 4.96e-04 | REAC:69278 | re | cell cycle, mitotic | CCNE2, CENPC1, CENPE, CENPF, ITGB3BP, MAD2L1, MNAT1, NEDD1, PSMA3, PSMA4, PSMC5, PSMD12, RFC1, STAG2, XPO1 |
| 2.21e-02 | TF:M00514_4 | tf | Factor: ATF4; motif: CVTGACGYMABG; match class: 4 | APPBP2, CD2AP, CDC5L, CDS1, CENPE, CLCN3, CLTC, HSU53209, ITGB3BP, NEDD1, RAD51C, RFC1, SMARCA5, STXBP3 |

**Table C. Genes inversely correlated to S6K1 and comparison to S6K2.**

| Gene name | S6K1  t-statistic | S6K1  p-value | S6K2  t-statistic | S6K2  p-value |
| --- | --- | --- | --- | --- |
| NM_018081__FLJ10385 | -5.31431 | 3.83E-07 | 1.99555 | 0.047831 |
| Contig1815_RC | -5.31677 | 3.79E-07 | -3.53326 | 0.000549 |
| NM_012237__SIR2L | -5.3209 | 3.72E-07 | 0.73906 | 0.461049 |
| NM_007326__DIA1 | -5.33195 | 3.53E-07 | -1.54497 | 0.124504 |
| NM_004454__ETV5 | -5.34051 | 3.40E-07 | 0.375005 | 0.708197 |
| NM_012127__CIZ1 | -5.34523 | 3.32E-07 | 0.569898 | 0.569617 |
| NM_002048__GAS1 | -5.34658 | 3.30E-07 | -4.47908 | 1.50E-05 |
| AF077051__M9 | -5.35778 | 3.14E-07 | 1.07351 | 0.284801 |
| NM_005766__FARP1 | -5.35892 | 3.12E-07 | -0.71828 | 0.473727 |
| AK000808 | -5.36986 | 2.96E-07 | 0.56503 | 0.572915 |
| Contig31757_RC | -5.37666 | 2.87E-07 | 3.51719 | 0.00058 |
| Contig4399_RC__MYH9 | -5.38536 | 2.76E-07 | -0.75529 | 0.451284 |
| AL137722__FLJ22215 | -5.39201 | 2.67E-07 | -0.41344 | 0.679885 |
| NM_000714__BZRP | -5.39298 | 2.66E-07 | 1.09708 | 0.2744 |
| NM_001667__ARL2 | -5.40769 | 2.49E-07 | 3.38323 | 0.000918 |
| NM_017933__FLJ20701 | -5.40985 | 2.46E-07 | -1.83593 | 0.068389 |
| NM_014164__FXYD5 | -5.41128 | 2.44E-07 | 1.92253 | 0.056473 |
| Contig55334_RC | -5.42085 | 2.34E-07 | -0.67306 | 0.501968 |
| AB037716__KIAA1295 | -5.42252 | 2.32E-07 | -2.82543 | 0.005379 |
| NM_003942__RPS6KA4 | -5.42317 | 2.31E-07 | 1.40814 | 0.161201 |
| NM_000014__A2M | -5.4304 | 2.24E-07 | -2.50098 | 0.013482 |
| NM_001436__FBL | -5.45741 | 1.97E-07 | 1.68819 | 0.093495 |
| NM_003139__SRPR | -5.45858 | 1.96E-07 | 0.28034 | 0.779611 |
| NM_003088__SNL | -5.46288 | 1.92E-07 | 1.27504 | 0.204307 |
| NM_001733__C1R | -5.46376 | 1.91E-07 | -2.31336 | 0.022089 |
| AK001638 | -5.48027 | 1.77E-07 | -1.29341 | 0.197898 |
| NM_006329__FBLN5 | -5.51951 | 1.47E-07 | -2.25478 | 0.025625 |
| AK000838__FLJ20831 | -5.53919 | 1.34E-07 | -3.29739 | 0.001224 |
| AL049957 | -5.54201 | 1.32E-07 | -2.73191 | 0.007069 |
| Contig58471_RC | -5.55375 | 1.25E-07 | -3.3932 | 0.000888 |
| AF209931__OR1I1 | -5.56643 | 1.18E-07 | -0.01131 | 0.990994 |
| NM_002743__PRKCSH | -5.56972 | 1.16E-07 | 2.79893 | 0.005816 |
| NM_014026__HSPC015 | -5.57397 | 1.14E-07 | 2.21969 | 0.027971 |
| NM_001996__FBLN1 | -5.57881 | 1.11E-07 | 0.396016 | 0.692667 |
| NM_014851__KIAA0469 | -5.58568 | 1.07E-07 | -2.79111 | 0.005951 |
| NM_001386__DPYSL2 | -5.58728 | 1.07E-07 | -3.61489 | 0.000412 |
| Contig46614_RC | -5.59513 | 1.03E-07 | -0.40126 | 0.68881 |
| Contig37385_RC | -5.6252 | 8.90E-08 | -4.55929 | 1.07E-05 |
| NM_006058__NAF1 | -5.63085 | 8.66E-08 | -0.41574 | 0.678205 |
| NM_012139__DELGEF | -5.63737 | 8.40E-08 | -0.59088 | 0.555511 |
| NM_003152__STAT5A | -5.66227 | 7.45E-08 | -2.97332 | 0.003444 |
| NM_000177__GSN | -5.67759 | 6.93E-08 | -4.94093 | 2.09E-06 |
| NM_000398__DIA1 | -5.68056 | 6.83E-08 | -1.34294 | 0.181361 |
| AB020649__KIAA0842 | -5.69042 | 6.51E-08 | -0.83901 | 0.402824 |
| NM_001456__FLNA | -5.69786 | 6.28E-08 | 0.851906 | 0.395652 |
| NM_000064__C3 | -5.7044 | 6.09E-08 | -2.64524 | 0.009051 |
| Contig40495_RC | -5.72105 | 5.62E-08 | -1.57945 | 0.116382 |
| NM_002101__GYPC | -5.7299 | 5.38E-08 | -2.59483 | 0.010423 |
| NM_014654__KIAA0468 | -5.74957 | 4.90E-08 | -0.33932 | 0.734856 |
| NM_003131__SRF | -5.77033 | 4.43E-08 | -0.99838 | 0.319737 |
| NM_017821__FLJ20435 | -5.77734 | 4.28E-08 | -0.72348 | 0.470534 |
| NM_001541__HSPB2 | -5.77972 | 4.23E-08 | -3.18829 | 0.001749 |
| Contig27701_RC | -5.79763 | 3.88E-08 | 1.04217 | 0.299044 |
| NM_018207__FLJ10759 | -5.8011 | 3.81E-08 | 2.29025 | 0.023429 |
| NM_017836__FLJ20473 | -5.80539 | 3.73E-08 | 0.005778 | 0.995397 |
| Contig54933_RC | -5.82668 | 3.37E-08 | -2.45754 | 0.015152 |
| Contig38130_RC | -5.83116 | 3.29E-08 | -2.76433 | 0.006435 |
| Contig56623_RC | -5.85674 | 2.91E-08 | 0.168001 | 0.866813 |
| AB014568__KIAA0668 | -5.87877 | 2.61E-08 | -0.08003 | 0.93632 |
| NM_016546__LOC51279 | -5.88546 | 2.53E-08 | -3.27771 | 0.001306 |
| NM_000557__GDF5 | -5.89365 | 2.43E-08 | 0.959744 | 0.33876 |
| NM_018458__BM042 | -5.89837 | 2.37E-08 | -2.93244 | 0.003902 |
| NM_007286__KIAA1029 | -5.89934 | 2.36E-08 | -2.43805 | 0.01596 |
| Contig46616_RC | -5.90853 | 2.25E-08 | -0.6459 | 0.519354 |
| Contig50101 | -5.92907 | 2.04E-08 | 0.791392 | 0.42999 |
| AB011173__KIAA0601 | -5.93846 | 1.95E-08 | 1.83418 | 0.068649 |
| Contig20926_RC | -5.9394 | 1.94E-08 | 0.365049 | 0.7156 |
| NM_006813__B4-2 | -5.97202 | 1.65E-08 | -0.487 | 0.626984 |
| NM_006509__RELB | -5.98736 | 1.53E-08 | 0.296026 | 0.767628 |
| NM_016735__LIMK1 | -6.0089 | 1.37E-08 | 0.10526 | 0.916313 |
| Contig54817_RC | -6.03045 | 1.23E-08 | 0.105938 | 0.915776 |
| NM_020650__LOC57333 | -6.03503 | 1.21E-08 | 0.685589 | 0.494052 |
| AL110218__DKFZP434A163 | -6.0413 | 1.17E-08 | 1.49618 | 0.136751 |
| NM_000366__TPM1 | -6.06682 | 1.03E-08 | -2.28298 | 0.023865 |
| Contig56261_RC | -6.06732 | 1.03E-08 | -4.58231 | 9.74E-06 |
| AF113694__LOC51219 | -6.07558 | 9.85E-09 | -0.21165 | 0.832677 |
| Contig52354_RC__FLJ22969 | -6.07614 | 9.83E-09 | 0.470599 | 0.638625 |
| Contig55538_RC__BA395L14.2 | -6.16318 | 6.35E-09 | 0.647065 | 0.518599 |
| Contig64794 | -6.16321 | 6.35E-09 | -0.07336 | 0.941617 |
| Contig49061_RC | -6.16436 | 6.31E-09 | -0.53265 | 0.595079 |
| Contig58325_RC | -6.16713 | 6.23E-09 | -0.77039 | 0.442306 |
| NM_006389__ORP150 | -6.17266 | 6.06E-09 | 1.07873 | 0.282475 |
| NM_000703__ATP1A3 | -6.18683 | 5.64E-09 | 2.78582 | 0.006043 |
| NM_005336__HDLBP | -6.19345 | 5.45E-09 | 0.779099 | 0.437174 |
| NM_017980__FLJ10044 | -6.25866 | 3.92E-09 | -3.15943 | 0.001919 |
| AL137743__DKFZP434G0310 | -6.29494 | 3.26E-09 | -1.92861 | 0.055707 |
| AK000715__PTRF | -6.30366 | 3.12E-09 | -1.77814 | 0.077448 |
| NM_004368__CNN2 | -6.31845 | 2.89E-09 | -3.45027 | 0.000731 |
| AB023216__KIAA0999 | -6.34713 | 2.50E-09 | -3.78843 | 0.00022 |
| NM_016091__HSPC025 | -6.34943 | 2.47E-09 | -1.34317 | 0.181287 |
| NM_003102__SOD3 | -6.37879 | 2.12E-09 | -1.146 | 0.253657 |
| NM_006487__FBLN1 | -6.39985 | 1.91E-09 | -1.8902 | 0.0607 |
| NM_006612__KIF1C | -6.41175 | 1.79E-09 | -1.6052 | 0.110596 |
| NM_000977__RPL13 | -6.43131 | 1.62E-09 | -0.18474 | 0.85369 |
| NM_014390__p100 | -6.45341 | 1.45E-09 | 1.68433 | 0.09424 |
| NM_004082__DCTN1 | -6.48275 | 1.24E-09 | -0.41638 | 0.677739 |
| NM_003651__CSDA | -6.48402 | 1.24E-09 | 0.461063 | 0.645435 |
| NM_001157__ANXA11 | -6.50433 | 1.11E-09 | -0.46435 | 0.643087 |
| NM_000701__ATP1A1 | -6.51073 | 1.08E-09 | -0.03028 | 0.975882 |
| NM_000528__MAN2B1 | -6.53586 | 9.45E-10 | 1.99778 | 0.047586 |
| AF055006__SEC6 | -6.53851 | 9.32E-10 | 0.886231 | 0.376941 |
| AB018324__KIAA0781 | -6.55418 | 8.59E-10 | -4.25259 | 3.74E-05 |
| NM_002444__MSN | -6.64188 | 5.43E-10 | 0.768051 | 0.443689 |
| AB033118__KIAA1292 | -6.65035 | 5.20E-10 | -1.61812 | 0.10778 |
| Contig51254_RC__FLJ00018 | -6.69415 | 4.13E-10 | -0.93975 | 0.348888 |
| NM_003169__SUPT5H | -6.73208 | 3.38E-10 | 2.53957 | 0.012138 |
| Contig54477_RC__LOC56963 | -6.79818 | 2.39E-10 | -2.48401 | 0.014114 |
| NM_005730__OS4 | -6.82918 | 2.02E-10 | -2.61608 | 0.009823 |
| NM_004723__ARHGEF2 | -6.89911 | 1.40E-10 | 0.947666 | 0.344855 |
| NM_015917__LOC51064 | -6.90554 | 1.35E-10 | -2.81408 | 0.005562 |
| NM_003754__EIF3S5 | -7.09654 | 4.84E-11 | 0.354187 | 0.723706 |
| AB037859__KIAA1438 | -7.15104 | 3.60E-11 | -2.37928 | 0.01863 |
| NM_003753__EIF3S7 | -7.16845 | 3.28E-11 | -0.94055 | 0.348479 |
| AB028950__KIAA1027 | -7.27624 | 1.82E-11 | -0.85494 | 0.393978 |
| NM_020360__PLSCR3 | -7.41743 | 8.39E-12 | -1.5614 | 0.12058 |
| AL161960__FLJ21324 | -7.49027 | 5.61E-12 | -1.9257 | 0.056072 |
| NM_016418__LOC51219 | -7.50569 | 5.15E-12 | -0.41498 | 0.67876 |
| NM_015343__HSA011916 | -7.82901 | 8.46E-13 | -2.4071 | 0.01732 |
| NM_018056__FLJ10315 | -8.2048 | 1.00E-13 | -0.82213 | 0.412337 |

**Table D. Pathways inversely correlated to S6K1.**

| p-value | Term | Term ID | Term description | Genes |
| --- | --- | --- | --- | --- |
|  |  |  |  |  |
| 7.60e-03 | GO:0050896 | BP | response to stimulus | A2M, ANXA11, ARHGEF2, ARL2, ATP1A3, C1R, C3, CNN2, CSDA, DCTN1, DPYSL2, ETV5, FARP1, FBLN5, FLJ20701, FLNA, GAS1, GDF5, GSN, MAN2B1, MYH9, PLSCR3, PRKCSH, RELB, RPS6KA4, SOD3, SRF, SRPR, STAT5A, SUPT5H, TPM1 |
| 4.68e-03 | GO:0051179 | BP | localization | A2M, ANXA11, ARHGEF2, ARL2, ATP1A3, C3, CIZ1, CNN2, DCTN1, DPYSL2, FBLN5, FLJ20701, FLNA, GAS1, GSN, HDLBP, KIF1C, MSN, MYH9, RPL13, SRF, SRPR, STAT5A, TPM1 |
| 2.14e-02 | GO:0048518 | BP | positive regulation of biological process | A2M, ARL2, C1R, C3, CIZ1, CNN2, CSDA, DCTN1, DPYSL2, ETV5, FLJ20701, FLNA, GAS1, GDF5, RELB, RPS6KA4, SRF, SRPR, STAT5A, SUPT5H, TPM1 |
| 6.56e-03 | GO:0005488 | MF | binding | A2M, ANXA11, ARHGEF2, ARL2, ATP1A3, C1R, C3, CIZ1, CNN2, CSDA, DCTN1, DPYSL2, ETV5, FARP1, FBL, FBLN1, FBLN5, FLNA, FXYD5, GAS1, GDF5, GSN, GYPC, HDLBP, KIF1C, LOC51064, MAN2B1, MSN, MYH9, PLSCR3, PRKCSH, PTRF, RELB, RPL13, RPS6KA4, SOD3, SRF, SRPR, STAT5A, SUPT5H, TPM1 |
| 2.24e-05 | BIOGRID:00000 | bi | BioGRID interaction data | A2M, ANXA11, ARHGEF2, ARL2, ATP1A3, C1R, C3, CIZ1 |
| 4.09e-03 | MI:hsa-miR-886-5p | mi | MI:hsa-miR-886-5p | ARL2, CNN2, DCTN1, FARP1, FLNA, FXYD5, HDLBP, MAN2B1, RPS6KA4 |
| 6.50e-03 | MI:hsa-miR-92b* | mi | MI:hsa-miR-92b* | ARL2, ATP1A3, CIZ1, DCTN1, FXYD5, MYH9, PRKCSH, RELB |
