# Supplementary material for: Revealing Different Roles of the mTOR-Targets S6K1 and S6K2 in Breast Cancer by Expression Profiling and Structural Analysis
Source: PLoS One. 2015 Dec 23;10(12):e0145013. doi: 10.1371/journal.pone.0145013 (PMC4689523; doi:10.1371/journal.pone.0145013)
Supplement: S3 Table — Genes positively correlated to S6K2 and comparison with S6K1 (Table A). Pathways positively correlated to S6K2 (Table B). Genes inversely correlated to S6K2 and comparison to S6K1 (Table C). Pathways inversely correlated to S6K2 (Table D). (DOCX) [file pone.0145013.s007.docx]

**Table A. Genes positively correlated to S6K2 and comparison with S6K1.**

| Genes positively correlated with S6K2 | S6K2  t-tatistic | S6K2  p-value | S6K1  t-statistic | S6K1  p-value |
| --- | --- | --- | --- | --- |
| NM_003952__RPS6KB2 | 17.418 | 1.45E-37 | 0.309444 | 0.757416 |
| NM_000485__APRT | 9.21653 | 2.97E-16 | 0.759599 | 0.448694 |
| NM_002461__MVD | 8.67202 | 7.23E-15 | -0.27565 | 0.783197 |
| AF070552 | 8.61565 | 1.00E-14 | -0.08088 | 0.93565 |
| r60_3__r60_3 | 7.66054 | 2.29E-12 | -2.19105 | 0.030002 |
| NM_005220__DLX3 | 7.42954 | 8.23E-12 | -2.55337 | 0.011674 |
| NM_020530__OSM | 7.35156 | 1.26E-11 | -1.51358 | 0.132251 |
| NM_002466__MYBL2 | 7.29461 | 1.72E-11 | 0.709361 | 0.479209 |
| Contig52872_RC__FLJ12517 | 7.25837 | 2.10E-11 | 1.53399 | 0.127153 |
| M96577__E2F1 | 7.23576 | 2.37E-11 | 2.02653 | 0.044494 |
| NM_014501__E2-EPF | 7.0664 | 5.92E-11 | 1.97121 | 0.050552 |
| NM_006076__RAB-R | 6.85657 | 1.82E-10 | -1.53812 | 0.12614 |
| NM_002708__PPP1CA | 6.81606 | 2.25E-10 | 0.415025 | 0.678721 |
| NM_003258__TK1 | 6.7349 | 3.45E-10 | 2.69637 | 0.007817 |
| NM_013289__KIR3DL1 | 6.72358 | 3.66E-10 | -0.7647 | 0.445658 |
| NM_005599__NHLH2 | 6.71864 | 3.76E-10 | -0.85487 | 0.393997 |
| NM_004219__PTTG1 | 6.68303 | 4.53E-10 | 1.91973 | 0.056803 |
| NM_003093__SNRPC | 6.60173 | 6.92E-10 | 1.79444 | 0.07477 |
| NM_006819__STIP1 | 6.59875 | 7.03E-10 | 0.992181 | 0.322718 |
| NM_015923__LOC51597 | 6.56056 | 8.57E-10 | -1.84539 | 0.066966 |
| NM_003158__STK6 | 6.45555 | 1.47E-09 | 2.90215 | 0.004269 |
| Contig42653_RC__LOC56961 | 6.43422 | 1.65E-09 | 1.22651 | 0.221942 |
| NM_003077__SMARCD2 | 6.40278 | 1.93E-09 | 5.32865 | 3.59E-07 |
| AL109730 | 6.36768 | 2.31E-09 | 1.42581 | 0.156015 |
| NM_019037__FLJ20591 | 6.32924 | 2.81E-09 | 0.783129 | 0.434794 |
| NM_003495__H4FM | 6.3043 | 3.20E-09 | 2.96184 | 0.00356 |
| NM_002720__PPP4C | 6.29821 | 3.30E-09 | -0.4325 | 0.666001 |
| NM_002653__PITX1 | 6.27994 | 3.62E-09 | 0.402628 | 0.687799 |
| Contig40184 | 6.27497 | 3.71E-09 | 0.56356 | 0.573901 |
| NM_021052__H2AFA | 6.26689 | 3.86E-09 | 2.24543 | 0.026212 |
| NM_005155__PPT2 | 6.26605 | 3.88E-09 | -0.04697 | 0.962598 |
| NM_001034__RRM2 | 6.25939 | 4.01E-09 | 1.48202 | 0.140447 |
| NM_003548__H4F2 | 6.23476 | 4.54E-09 | 3.1312 | 0.002096 |
| NM_005412__SHMT2 | 6.21694 | 4.97E-09 | -1.45779 | 0.147003 |
| Contig44882_RC | 6.18521 | 5.83E-09 | -1.73312 | 0.085143 |
| NM_004701__CCNB2 | 6.18279 | 5.90E-09 | 2.35291 | 0.019934 |
| NM_018683__ZNF313 | 6.16713 | 6.39E-09 | 0.136322 | 0.891751 |
| NM_003833__MATN4 | 6.15262 | 6.87E-09 | -1.16068 | 0.247628 |
| NM_020187__DC12 | 6.11843 | 8.15E-09 | -2.13267 | 0.034591 |
| NM_006396__SSSCA1 | 6.10314 | 8.80E-09 | -0.19443 | 0.846106 |
| NM_019056__FLJ20494 | 6.09433 | 9.20E-09 | -0.8446 | 0.399691 |
| Contig16298_RC | 6.04052 | 1.20E-08 | -0.66973 | 0.504068 |
| Contig31288_RC | 6.0344 | 1.24E-08 | 4.76597 | 4.43E-06 |
| L22005__CDC34 | 6.01448 | 1.37E-08 | 1.35374 | 0.177869 |
| NM_018052__FLJ10305 | 6.01227 | 1.38E-08 | -2.19186 | 0.029942 |
| Contig61254_RC | 6.00859 | 1.41E-08 | -0.16165 | 0.871804 |
| NM_001916__CYC1 | 5.91787 | 2.20E-08 | 0.102098 | 0.918816 |
| NM_004111__FEN1 | 5.89278 | 2.49E-08 | 2.84633 | 0.005047 |
| NM_003600__STK15 | 5.88633 | 2.57E-08 | 2.49872 | 0.01355 |
| NM_004217__STK12 | 5.8777 | 2.68E-08 | 0.711588 | 0.477833 |
| Contig48913_RC | 5.86624 | 2.84E-08 | 0.588401 | 0.557154 |
| M55914__MPB1 | 5.85711 | 2.96E-08 | -4.23667 | 3.96E-05 |
| NM_003765__STX10 | 5.83448 | 3.31E-08 | -3.3699 | 0.000958 |
| AB024704__C20ORF1 | 5.82957 | 3.39E-08 | 1.18282 | 0.238764 |
| NM_003504__CDC45L | 5.82649 | 3.44E-08 | 1.71406 | 0.088597 |
| NM_004461__FARSL | 5.81077 | 3.71E-08 | -3.45682 | 0.000713 |
| NM_001168__BIRC5 | 5.80518 | 3.82E-08 | 2.55639 | 0.011577 |
| NM_020675__AD024 | 5.80205 | 3.88E-08 | 4.35998 | 2.41E-05 |
| NM_017882__FLJ20561 | 5.79981 | 3.92E-08 | -1.67247 | 0.09653 |
| NM_006607__PTTG2 | 5.79591 | 3.99E-08 | 2.72856 | 0.007127 |
| NM_003544__H4FI | 5.79548 | 4.00E-08 | 1.29625 | 0.196894 |
| NM_004553__NDUFS6 | 5.79287 | 4.05E-08 | 1.68991 | 0.093136 |
| NM_002707__PPM1G | 5.7828 | 4.25E-08 | -2.00696 | 0.046563 |
| NM_014207__CD5 | 5.76844 | 4.56E-08 | -0.62477 | 0.533078 |
| NM_002212__ITGB4BP | 5.76561 | 4.62E-08 | -0.21511 | 0.829979 |
| Contig15639_RC | 5.76199 | 4.70E-08 | -0.16851 | 0.86641 |
| Contig56843_RC__CCNB1 | 5.75972 | 4.76E-08 | 1.6451 | 0.102057 |
| Contig56017_RC | 5.74445 | 5.12E-08 | -1.35247 | 0.178274 |
| Contig41787_RC | 5.74258 | 5.17E-08 | -1.4755 | 0.142188 |
| D43950__KIAA0098 | 5.73558 | 5.34E-08 | 1.32256 | 0.188008 |
| Contig56396_RC | 5.72261 | 5.69E-08 | -1.20285 | 0.230942 |
| NM_007103__NDUFV1 | 5.72202 | 5.70E-08 | -3.29994 | 0.00121 |
| NM_002131__HMGIY | 5.72072 | 5.74E-08 | -0.58909 | 0.556696 |
| Contig39603_RC__CNOT3 | 5.7092 | 6.07E-08 | 1.92884 | 0.055652 |
| AF000560 | 5.70824 | 6.10E-08 | 1.40531 | 0.162011 |
| NM_002799__PSMB7 | 5.69419 | 6.52E-08 | 1.621 | 0.107132 |
| NM_004320__ATP2A1 | 5.69412 | 6.52E-08 | -1.58932 | 0.114108 |
| NM_007274__HBACH | 5.69383 | 6.53E-08 | -0.24004 | 0.81063 |
| Contig38949_RC | 5.68948 | 6.67E-08 | -2.58915 | 0.010575 |
| NM_014272__ADAMTS7 | 5.67374 | 7.19E-08 | -2.76371 | 0.006436 |
| Contig1699_RC__FLJ12438 | 5.66975 | 7.33E-08 | -1.47371 | 0.142669 |
| NM_014182__HSPC160 | 5.65958 | 7.70E-08 | 3.4007 | 0.000863 |
| Contig29528 | 5.65418 | 7.90E-08 | 0.878652 | 0.381005 |
| AF161371 | 5.63977 | 8.46E-08 | 0.304152 | 0.761436 |
| NM_001809__CENPA | 5.63819 | 8.53E-08 | 2.71996 | 0.007305 |
| NM_002757__MAP2K5 | 5.6348 | 8.66E-08 | 1.37412 | 0.171468 |
| Contig51103_RC | 5.6323 | 8.77E-08 | -2.24643 | 0.026147 |
| NM_002918__RFX1 | 5.62075 | 9.27E-08 | 0.980242 | 0.328556 |
| NM_005082__ZNF147 | 5.61298 | 9.61E-08 | 3.93264 | 0.000128 |
| NM_005945__MPB1 | 5.60721 | 9.88E-08 | -4.53992 | 1.15E-05 |
| NM_016535__HSPC189 | 5.60617 | 9.93E-08 | -2.43568 | 0.016044 |
| U96131__TRIP13 | 5.60524 | 9.97E-08 | 0.466068 | 0.641847 |
| NM_013282__ICBP90 | 5.6034 | 1.01E-07 | 3.38316 | 0.000916 |
| NM_002883__RANGAP1 | 5.60321 | 1.01E-07 | -1.33759 | 0.183068 |
| Contig35661_RC | 5.59864 | 1.03E-07 | -1.56643 | 0.119369 |
| NM_005787__NOT56L | 5.59012 | 1.07E-07 | 0.603984 | 0.546773 |
| NM_005192__CDKN3 | 5.57684 | 1.14E-07 | 3.18513 | 0.001762 |
| NM_004203__PKMYT1 | 5.56565 | 1.20E-07 | 1.44718 | 0.149947 |
| NM_003198__TCEB3 | 5.55603 | 1.26E-07 | -2.01668 | 0.045525 |
| NM_016185__LOC51155 | 5.54771 | 1.31E-07 | 2.8825 | 0.004529 |
| NM_018455__BM039 | 5.53412 | 1.40E-07 | 1.62369 | 0.106556 |
| Contig52490_RC | 5.5313 | 1.42E-07 | 4.59058 | 9.32E-06 |
| Contig48114_RC | 5.52602 | 1.45E-07 | 0.966394 | 0.335413 |
| Contig55038_RC__MCOLN1 | 5.5164 | 1.52E-07 | 1.76422 | 0.079744 |
| NM_001255__CDC20 | 5.51544 | 1.53E-07 | -0.75033 | 0.454242 |
| Contig46634 | 5.51197 | 1.55E-07 | -2.83029 | 0.005293 |
| NM_005828__HAN11 | 5.50942 | 1.57E-07 | 5.64909 | 7.94E-08 |
| Contig41413_RC | 5.50334 | 1.62E-07 | 2.84502 | 0.005066 |
| NM_021000__PTTG3 | 5.48589 | 1.75E-07 | 1.08389 | 0.280164 |
| Contig9227 | 5.48091 | 1.79E-07 | -0.95582 | 0.340711 |
| NM_004358__CDC25B | 5.47973 | 1.80E-07 | 0.680377 | 0.497322 |
| NM_016209__LOC51693 | 5.47819 | 1.82E-07 | 0.855132 | 0.393851 |
| NM_004704__U3-55K | 5.4778 | 1.82E-07 | -2.98398 | 0.003326 |
| Contig55031_RC__NG7 | 5.47454 | 1.85E-07 | -0.475 | 0.635486 |
| NM_001428__ENO1 | 5.47186 | 1.87E-07 | -4.70524 | 5.74E-06 |
| NM_014275__MGAT4B | 5.46563 | 1.93E-07 | -1.14921 | 0.252311 |
| NM_018410__DKFZp762E1312 | 5.45339 | 2.04E-07 | 1.74419 | 0.083188 |
| NM_016326__LOC51192 | 5.45336 | 2.04E-07 | 0.330231 | 0.741689 |
| Contig55189_RC | 5.45206 | 2.05E-07 | 0.433762 | 0.665089 |
| NM_004596__SNRPA | 5.44179 | 2.16E-07 | -3.45194 | 0.000725 |
| NM_002875__RAD51 | 5.44175 | 2.16E-07 | 1.77088 | 0.078625 |
| D14678__KNSL2 | 5.42161 | 2.37E-07 | 0.417159 | 0.677163 |
| NM_005371__METTL1 | 5.41805 | 2.41E-07 | 0.495965 | 0.62065 |
| NM_005480__TROAP | 5.40803 | 2.52E-07 | 1.49573 | 0.136839 |
| NM_003491__ARD1 | 5.40569 | 2.55E-07 | -0.97224 | 0.332508 |
| Contig56217_RC | 5.37778 | 2.90E-07 | 0.768967 | 0.44313 |
| NM_005796__PP15 | 5.37637 | 2.92E-07 | -0.38562 | 0.70033 |
| Contig43741_RC | 5.37616 | 2.93E-07 | -2.19169 | 0.029954 |
| NM_002602__PDE6G | 5.36757 | 3.04E-07 | 0.916883 | 0.360686 |
| NM_002904__RDBP | 5.36654 | 3.06E-07 | 0.043601 | 0.965281 |
| AK000860__DKFZP762I166 | 5.36207 | 3.12E-07 | 0.126653 | 0.899386 |
| Contig51373_RC | 5.35877 | 3.17E-07 | -3.35352 | 0.001012 |
| Contig16026_RC | 5.35117 | 3.28E-07 | -0.04545 | 0.963812 |
| NM_013299__HSU79266 | 5.34555 | 3.37E-07 | -0.10944 | 0.913002 |
| NM_000454__SOD1 | 5.34309 | 3.41E-07 | 4.90553 | 2.41E-06 |
| Contig16250_RC | 5.32794 | 3.66E-07 | 0.604377 | 0.546512 |
| Contig28418_RC | 5.32591 | 3.69E-07 | -2.7668 | 0.006379 |
| NM_017713__FLJ20211 | 5.32502 | 3.71E-07 | -1.6679 | 0.097436 |
| NM_000402__G6PD | 5.32482 | 3.71E-07 | -0.66132 | 0.509427 |
| NM_014176__HSPC150 | 5.32418 | 3.72E-07 | 3.87028 | 0.000162 |
| AB040950__KIAA1517 | 5.32404 | 3.72E-07 | 0.344532 | 0.730932 |
| NM_021257__NGB | 5.31604 | 3.86E-07 | -0.60243 | 0.547804 |
| NM_000291__PGK1 | 5.31298 | 3.92E-07 | 0.2849 | 0.776116 |
| NM_006222__PIN1L | 5.31222 | 3.93E-07 | -0.46572 | 0.642099 |

**Table B. Pathways positively correlated to S6K2.**

| p-value | Term | Term ID | Term description | Genes |
| --- | --- | --- | --- | --- |
| 4.68e-02 | GO:0006259 | BP | DNA metabolic process | CCNB1, CDC34, CENPA, FEN1, PPP4C, PTTG1, PTTG2, RAD51, RRM2, SOD1, TK1, TRIP13 |
| 1.97e-03 | GO:0044237 | BP | cellular metabolic process | APRT, ATP2A1, BIRC5, CCNB1, CCNB2, CDC20, CDC25B, CDC34, CDKN3, CENPA, CYC1, E2F1, ENO1, FEN1, G6PD, MAP2K5, METTL1, MGAT4B, MVD, MYBL2, NDUFS6, NDUFV1, NHLH2, OSM, PDE6G, PGK1, PITX1, PKMYT1, PPM1G, PPP4C, PSMB7, PTTG1, PTTG2, RAD51, RANGAP1, RPS6KB2, RRM2, SHMT2, SMARCD2, SNRPA, SNRPC, SOD1, TCEB3, TK1, TRIP13 |
| 1.98e-02 | GO:0001556 | BP | oocyte maturation | CCNB1, CDC25B, TRIP13 |
| 1.81e-09 | GO:0022403 | BP | cell cycle phase | BIRC5, CCNB1, CCNB2, CDC20, CDC25B, CDC34, CDKN3, CENPA, E2F1, FEN1, MYBL2, OSM, PKMYT1, PSMB7, PTTG1, RAD51, RANGAP1, RRM2, SSSCA1, TRIP13 |
| 1.94e-02 | GO:0005654 | CC | nucleoplasm | CCNB1, CDC20, CDC25B, CENPA, E2F1, FEN1, NHLH2, PITX1, PKMYT1, PSMB7, RAD51, RPS6KB2, RRM2, SNRPA, SNRPC, TCEB3 |
| 1.42e-02 | GO:0005634 | CC | nucleus | APRT, BIRC5, CCNB1, CCNB2, CDC20, CDC25B, CDC34, CENPA, DLX3, E2F1, ENO1, FEN1, MAP2K5, METTL1, MYBL2, NHLH2, PITX1, PKMYT1, PPM1G, PPP4C, PSMB7, PTTG1, PTTG2, RAD51, RANGAP1, RFX1, RPS6KB2, RRM2, SMARCD2, SNRPA, SNRPC, SOD1, STIP1, TCEB3, TRIP13 |
| 3.11e-02 | GO:0005829 | CC | cytosol | APRT, BIRC5, CCNB1, CCNB2, CDC20, CDC25B, CENPA, ENO1, G6PD, MAP2K5, MVD, NGB, PDE6G, PGK1, PSMB7, PTTG1, RANGAP1, RRM2, SOD1, TK1 |
| 3.43e-04 | GO:0005488 | MF | binding | ADAMTS7, APRT, ATP2A1, BIRC5, CCNB1, CCNB2, CD5, CDC20, CDC25B, CDC34, CDKN3, CENPA, CYC1, DLX3, E2F1, ENO1, FEN1, G6PD, MAP2K5, MATN4, METTL1, MGAT4B, MVD, MYBL2, NDUFV1, NGB, NHLH2, OSM, PDE6G, PGK1, PITX1, PKMYT1, PPM1G, PPP4C, PSMB7, PTTG1, PTTG2, RAD51, RANGAP1, RFX1, RPS6KB2, RRM2, SHMT2, SNRPA, SNRPC, SOD1, SSSCA1, STIP1, STX10, TCEB3, TK1, TRIP13, TROAP, ZNF313 |
| 7.64e-10 | BIOGRID:00000 | bi | BioGRID interaction data | APRT, ATP2A1, BIRC5, CCNB1, CCNB2, CD5, CDC20, CDC25B, CDC34, CDKN3, CENPA, CYC1, E2F1, ENO1, FEN1, G6PD, MAP2K5, MATN4, MCOLN1 |
| 1.86e-03 | KEGG:04114 | ke | oocyte meiosis | CCNB1, CCNB2, CDC20, PKMYT1, PTTG1, PTTG2 |
| 1.91e-05 | KEGG:04110 | ke | cell cycle | CCNB1, CCNB2, CDC20, CDC25B, E2F1, PKMYT1, PTTG1, PTTG2 |
| 2.32e-02 | MI:rno-miR-336 | mi | MI:rno-miR-336 | MAP2K5, MCOLN1, MYBL2, NDUFS6, NGB, PPP4C, RPS6KB2, RRM2, SNRPC |
| 9.70e-04 | REAC:69273 | re | cyclin A/B1 associated events during G2/M transition | CCNB1, CCNB2, CDC25B, PKMYT1 |
| 8.45e-05 | REAC:69278 | re | cell cycle, mitotic | BIRC5, CCNB1, CCNB2, CDC20, CDC25B, CENPA, E2F1, FEN1, PKMYT1, PSMB7, PTTG1, RANGAP1, RRM2 |
| 3.19e-02 | TF:M004283 | tf | Factor: E2F-1; motif: NKTSSCGC; match class: 3 | APRT, CCNB1, CD5, CDC20, CDC25B, CDC34, CENPA, CYC1, E2F1, ENO1, FEN1, G6PD, MAP2K5, MCOLN1, METTL1, MGAT4B, MVD, MYBL2, NDUFS6, NDUFV1, PGK1, PITX1, PKMYT1, PPM1G, PPP4C, RANGAP1, RFX1, RPS6KB2, RRM2, SHMT2, SNRPA, SNRPC, SOD1, STIP1, TK1, TRIP13, TROAP, ZNF313 |

**Table C. Genes inversely correlated to S6K2 and comparison to S6K1.**

| Gene | S6K2  t-statistic | S6K2  p-value | S6K1  t-statistic | S6K1  p-value |
| --- | --- | --- | --- | --- |
| Contig53909_RC__LOC56849 | -7,6584 | 2,32E-12 | -0,898192 | 0,370532 |
| X57025__IGF1 | -7,6115 | 3,01E-12 | 0,269094 | 0,78823 |
| AF131817 | -7,23884 | 2,33E-11 | -1,27365 | 0,204771 |
| AL050148 | -7,15096 | 3,76E-11 | -2,44076 | 0,0158288 |
| NM_013231__FLRT2 | -7,08597 | 5,33E-11 | -3,4384 | 0,000758918 |
| Contig50670 | -6,8667 | 1,72E-10 | -0,505698 | 0,613816 |
| Contig31646_RC | -6,85788 | 1,80E-10 | -3,17166 | 0,0018407 |
| Contig54847_RC | -6,82658 | 2,13E-10 | -1,89788 | 0,059646 |
| Contig50367__FLJ21935 | -6,66337 | 5,02E-10 | -2,92791 | 0,00394856 |
| Contig38928_RC | -6,66217 | 5,05E-10 | -1,68694 | 0,0937074 |
| NM_005780__LHFP | -6,61677 | 6,40E-10 | 0,251158 | 0,802038 |
| Contig51393_RC__KIAA1628 | -6,53683 | 9,69E-10 | -2,44579 | 0,0156183 |
| NM_004684__SPARCL1 | -6,47591 | 1,33E-09 | 1,51354 | 0,132261 |
| NM_001920__DCN | -6,44519 | 1,56E-09 | -2,08491 | 0,0387834 |
| AL080218 | -6,4037 | 1,92E-09 | -3,93461 | 0,000127413 |
| Contig52305_RC | -6,30451 | 3,19E-09 | 1,83713 | 0,068183 |
| Contig50855_RC | -6,30369 | 3,21E-09 | -2,09648 | 0,0377295 |
| Contig55048_RC | -6,24446 | 4,33E-09 | -1,98443 | 0,0490445 |
| Contig58512_RC | -6,22924 | 4,67E-09 | -2,12488 | 0,0352465 |
| Contig2141_RC | -6,20244 | 5,35E-09 | 0,30156 | 0,763408 |
| Contig48249_RC | -6,19592 | 5,53E-09 | -0,795871 | 0,427373 |
| Contig49279_RC | -6,1796 | 6,00E-09 | 0,0333573 | 0,973434 |
| Contig55801_RC | -6,1689 | 6,33E-09 | -1,86755 | 0,0637891 |
| NM_000313__PROS1 | -6,14172 | 7,26E-09 | -0,148746 | 0,881955 |
| AL137332 | -6,14048 | 7,30E-09 | 1,82785 | 0,0695724 |
| Contig40105 | -6,07763 | 9,99E-09 | -0,87398 | 0,383536 |
| Contig26641 | -6,06195 | 1,08E-08 | 1,3842 | 0,168367 |
| AL050228 | -6,06192 | 1,08E-08 | -2,15385 | 0,0328601 |
| Contig51220_RC | -6,06111 | 1,09E-08 | -3,44423 | 0,000743929 |
| Contig1667_RC | -6,04715 | 1,16E-08 | 0,32004 | 0,749386 |
| NM_005261__GEM | -6,03322 | 1,25E-08 | -0,448659 | 0,654329 |
| D50406__RECK | -6,02551 | 1,29E-08 | -2,15813 | 0,0325197 |
| NM_007038__ADAMTS5 | -6,01252 | 1,38E-08 | 0,539929 | 0,590052 |
| NM_003014__SFRP4 | -6,00978 | 1,40E-08 | -2,36081 | 0,0195298 |
| Contig43759_RC | -5,99945 | 1,47E-08 | -4,27253 | 3,43E-05 |
| NM_004538__NAP1L3 | -5,98759 | 1,56E-08 | -0,615898 | 0,538902 |
| Contig56276_RC | -5,94612 | 1,92E-08 | 1,44615 | 0,150235 |
| NM_006472__VDUP1 | -5,93331 | 2,04E-08 | -1,22928 | 0,220905 |
| Contig3607_RC | -5,8627 | 2,88E-08 | -2,61721 | 0,00977935 |
| NM_000214__JAG1 | -5,8288 | 3,40E-08 | -1,48849 | 0,138735 |
| NM_005100__AKAP12 | -5,80489 | 3,82E-08 | -0,957631 | 0,3398 |
| Contig50360_RC | -5,78444 | 4,22E-08 | -1,19069 | 0,235669 |
| Contig52904_RC | -5,77485 | 4,42E-08 | -0,330261 | 0,741667 |
| NM_004787__SLIT2 | -5,76032 | 4,74E-08 | -2,71437 | 0,00742387 |
| NM_012429__SEC14L2 | -5,741 | 5,21E-08 | -3,37587 | 0,000938506 |
| AL133047 | -5,737 | 5,31E-08 | 2,28617 | 0,0236539 |
| Contig44503_RC | -5,72742 | 5,56E-08 | 1,35037 | 0,178945 |
| NM_020353__LOC57088 | -5,709 | 6,07E-08 | 0,631378 | 0,528761 |
| NM_001393__ECM2 | -5,70587 | 6,17E-08 | -0,438284 | 0,661815 |
| AB040971__KIAA1538 | -5,594 | 1,05E-07 | -1,74467 | 0,0831042 |
| Contig36714_RC | -5,56205 | 1,22E-07 | 0,668702 | 0,50472 |
| Contig53881_RC | -5,54498 | 1,33E-07 | -0,71723 | 0,474355 |
| AB011115__KIAA0543 | -5,53644 | 1,38E-07 | -2,69911 | 0,00775602 |
| NM_019018__FLJ11127 | -5,53039 | 1,42E-07 | -1,96892 | 0,0508174 |
| AL049949 | -5,52567 | 1,45E-07 | -2,80776 | 0,00565709 |
| NM_001753__CAV1 | -5,48333 | 1,77E-07 | -2,44105 | 0,0158166 |
| AI928427_RC | -5,44884 | 2,09E-07 | -4,75321 | 4,67E-06 |
| Contig14068_RC | -5,4422 | 2,15E-07 | 1,51938 | 0,130786 |
| Contig53953_RC | -5,42588 | 2,32E-07 | -0,375728 | 0,707654 |
| Contig54263_RC | -5,42226 | 2,36E-07 | 2,23676 | 0,0267869 |
| NM_014965__KIAA1042 | -5,41883 | 2,40E-07 | 0,813506 | 0,417226 |
| D80002__KIAA0180 | -5,40025 | 2,62E-07 | -3,85698 | 0,000170371 |
| Contig53742_RC | -5,39068 | 2,73E-07 | 1,56043 | 0,12078 |
| AL133605__PELI2 | -5,37833 | 2,90E-07 | -1,58399 | 0,115316 |
| Contig53661_RC | -5,3695 | 3,02E-07 | -1,92253 | 0,056447 |
| Contig778_RC | -5,36181 | 3,13E-07 | -1,12641 | 0,261803 |
| Contig45367_RC | -5,35359 | 3,25E-07 | -1,3541 | 0,177755 |
| Contig45859_RC | -5,32786 | 3,66E-07 | -0,844775 | 0,399591 |

**Table D. Pathways inversely correlated to S6K2.**

| p-value | Term | Term ID | Term description | Genes |
| --- | --- | --- | --- | --- |
| 6.30e-03 | GO:0022612 | BP | gland morphogenesis | CAV1, IGF1, SFRP4, SLIT2 |
| 1.06e-02 | GO:0048584 | BP | positive regulation of response to stimulus | AKAP12, CAV1, IGF1, JAG1, PELI2, PROS1, SFRP4, SLIT2 |
| 7.59e-03 | GO:0001944 | BP | vasculature development | CAV1, IGF1, JAG1, RECK, SFRP4, SLIT2 |
| 7.68e-03 | GO:0044421 | CC | extracellular region part | ADAMTS5, DCN, ECM2, FLRT2, IGF1, SFRP4, SLIT2, SPARCL1 |
| 4.97e-02 | CORUM:2462 | co | caveolin-1 homodimer complex | CAV1 |
