# Supplementary material for: Revealing Different Roles of the mTOR-Targets S6K1 and S6K2 in Breast Cancer by Expression Profiling and Structural Analysis
Source: PLoS One. 2015 Dec 23;10(12):e0145013. doi: 10.1371/journal.pone.0145013 (PMC4689523; doi:10.1371/journal.pone.0145013)
Supplement: S4 Table — Genes positively correlated to 4EBP1 (Table A). Pathways positively correlated to 4EBP1 (Table B). Genes inversely correlated to 4EBP1 (Table C). Pathways inversely correlated to 4EBP1 (Table D). (DOCX) [file pone.0145013.s008.docx]

**Table A. Genes positively correlated to 4EBP1.**

| Gene | t-statistic | p-value |
| --- | --- | --- |
| NM_004095__EIF4EBP1 | 20.9479 | 5.58E-46 |
| U96131__TRIP13 | 10.8601 | 1.53E-20 |
| NM_003258__TK1 | 9.42392 | 8.70E-17 |
| NM_005412__SHMT2 | 9.08436 | 6.48E-16 |
| Contig61254_RC | 9.05632 | 7.64E-16 |
| NM_000365__TPI1 | 8.98993 | 1.13E-15 |
| NM_001168__BIRC5 | 8.82295 | 3.00E-15 |
| NM_000291__PGK1 | 8.76379 | 4.24E-15 |
| NM_004701__CCNB2 | 8.72292 | 5.37E-15 |
| D43950__KIAA0098 | 8.61228 | 1.02E-14 |
| NM_016185__LOC51155 | 8.60324 | 1.08E-14 |
| NM_006067__NOC4 | 8.55628 | 1.41E-14 |
| NM_014342__MTCH2 | 8.40438 | 3.40E-14 |
| NM_020122__DKFZP434L1021 | 8.32504 | 5.36E-14 |
| NM_001255__CDC20 | 8.25009 | 8.23E-14 |
| L48692__LOC56902 | 8.18985 | 1.16E-13 |
| AF141882__APMCF1 | 8.16997 | 1.30E-13 |
| NM_007274__HBACH | 8.13663 | 1.57E-13 |
| NM_005796__PP15 | 8.12049 | 1.72E-13 |
| Contig66705_RC | 8.12034 | 1.72E-13 |
| NM_004255__COX5A | 8.0837 | 2.12E-13 |
| NM_018188__FLJ10709 | 8.06117 | 2.41E-13 |
| NM_001237__CCNA2 | 8.05789 | 2.46E-13 |
| NM_004846__EIF4EL3 | 8.05361 | 2.52E-13 |
| NM_021203__APMCF1 | 8.04478 | 2.65E-13 |
| NM_003681__PDXK | 8.03165 | 2.85E-13 |
| M96577__E2F1 | 8.0123 | 3.18E-13 |
| NM_001809__CENPA | 7.98575 | 3.70E-13 |
| NM_016491__MRPL37 | 7.96444 | 4.17E-13 |
| Contig57584_RC | 7.93823 | 4.84E-13 |
| Contig55189_RC | 7.91853 | 5.41E-13 |
| AF078866 | 7.90484 | 5.84E-13 |
| NM_018683__ZNF313 | 7.90202 | 5.93E-13 |
| NM_019013__FLJ10156 | 7.87805 | 6.79E-13 |
| NM_018840__LOC55969 | 7.84994 | 7.95E-13 |
| NM_001034__RRM2 | 7.84708 | 8.08E-13 |
| NM_014501__E2-EPF | 7.84533 | 8.16E-13 |
| NM_002707__PPM1G | 7.80302 | 1.03E-12 |
| D55716__MCM7 | 7.77973 | 1.18E-12 |
| NM_006114__D19S1177E | 7.76968 | 1.25E-12 |
| NM_006623__PHGDH | 7.76557 | 1.28E-12 |
| Contig38901_RC | 7.75756 | 1.33E-12 |
| NM_004217__STK12 | 7.7537 | 1.36E-12 |
| NM_003504__CDC45L | 7.74948 | 1.40E-12 |
| NM_001689__ATP5G3 | 7.74497 | 1.43E-12 |
| NM_003498__SNN | 7.73485 | 1.52E-12 |
| NM_002875__RAD51 | 7.72915 | 1.56E-12 |
| NM_002949__MRPL12 | 7.72685 | 1.58E-12 |
| AF155652__DKFZP434L1021 | 7.72406 | 1.61E-12 |
| NM_004203__PKMYT1 | 7.68384 | 2.01E-12 |
| NM_003600__STK15 | 7.63665 | 2.62E-12 |
| NM_004053__BYSL | 7.63592 | 2.63E-12 |
| NM_002626__PFKL | 7.61774 | 2.91E-12 |
| NM_003683__D21S2056E | 7.59909 | 3.23E-12 |
| NM_018410__DKFZp762E1312 | 7.58925 | 3.41E-12 |
| NM_002808__PSMD2 | 7.58583 | 3.47E-12 |
| NM_013277__ID-GAP | 7.54783 | 4.29E-12 |
| AF112213__LOC55969 | 7.54091 | 4.45E-12 |
| NM_002904__RDBP | 7.53141 | 4.69E-12 |
| NM_002914__RFC2 | 7.51514 | 5.13E-12 |
| NM_003875__GMPS | 7.5147 | 5.15E-12 |
| Contig48270_RC | 7.47889 | 6.27E-12 |
| NM_001274__CHEK1 | 7.47427 | 6.43E-12 |
| NM_001673__ASNS | 7.47393 | 6.44E-12 |
| D38553__KIAA0074 | 7.44741 | 7.46E-12 |
| NM_018389__FLJ11320 | 7.44312 | 7.64E-12 |
| NM_004596__SNRPA | 7.43488 | 7.99E-12 |
| AF070552 | 7.43231 | 8.10E-12 |
| NM_007019__UBCH10 | 7.42375 | 8.49E-12 |
| NM_001569__IRAK1 | 7.40655 | 9.33E-12 |
| AB024704__C20ORF1 | 7.39263 | 1.01E-11 |
| NM_018135__FLJ10548 | 7.38033 | 1.08E-11 |
| AF047002__ALY | 7.36907 | 1.15E-11 |
| X97074__AP2S1 | 7.35812 | 1.22E-11 |
| NM_003720__DSCR2 | 7.35281 | 1.25E-11 |
| NM_006009__TUBA3 | 7.34297 | 1.32E-11 |
| Contig49169_RC | 7.31263 | 1.56E-11 |
| NM_004493__HADH2 | 7.31129 | 1.57E-11 |
| NM_014321__ORC6L | 7.31051 | 1.58E-11 |
| NM_004111__FEN1 | 7.30239 | 1.65E-11 |
| AK000552__WDR5 | 7.28928 | 1.77E-11 |
| NM_002950__RPN1 | 7.28784 | 1.79E-11 |
| NM_020675__AD024 | 7.27538 | 1.91E-11 |
| Contig55997_RC | 7.27391 | 1.93E-11 |
| NM_013237__PX19 | 7.26132 | 2.06E-11 |
| NM_004804__CIAO1 | 7.24927 | 2.20E-11 |
| NM_006087__TUBB5 | 7.22578 | 2.50E-11 |
| Contig57097_RC | 7.22283 | 2.54E-11 |
| NM_014791__KIAA0175 | 7.22138 | 2.56E-11 |
| Contig55725_RC | 7.21938 | 2.59E-11 |
| NM_003132__SRM | 7.19883 | 2.90E-11 |
| NM_002168__IDH2 | 7.19598 | 2.94E-11 |
| NM_016405__HSU93243 | 7.18107 | 3.19E-11 |
| AF052155__SEC13L1 | 7.17761 | 3.25E-11 |
| NM_016072__LOC51026 | 7.15624 | 3.65E-11 |
| NM_002046__GAPD | 7.14052 | 3.97E-11 |
| Contig48913_RC | 7.13898 | 4.01E-11 |
| Contig53130 | 7.13451 | 4.10E-11 |
| NM_012247__SPS | 7.12503 | 4.32E-11 |
| S90469__POR | 7.12355 | 4.35E-11 |
| Contig31221_RC | 7.10871 | 4.72E-11 |
| NM_016495__LOC51256 | 7.10772 | 4.74E-11 |
| NM_002708__PPP1CA | 7.10693 | 4.76E-11 |
| NM_006845__KNSL6 | 7.09101 | 5.19E-11 |
| NM_018518__PRO2249 | 7.06749 | 5.89E-11 |
| AF121255__EIF2C2 | 7.05062 | 6.45E-11 |
| NM_005945__MPB1 | 7.0458 | 6.62E-11 |
| NM_006341__MAD2L2 | 7.04449 | 6.66E-11 |
| NM_017876__FLJ20552 | 7.0436 | 6.69E-11 |
| NM_005837__RPP20 | 7.0266 | 7.33E-11 |
| Contig34952 | 7.02071 | 7.57E-11 |
| AL117600__DKFZP564J0863 | 7.00328 | 8.31E-11 |
| Contig50880_RC | 6.99986 | 8.46E-11 |
| AF161383 | 6.99742 | 8.57E-11 |
| NM_006027__EXO1 | 6.99601 | 8.64E-11 |
| Contig41893 | 6.99598 | 8.64E-11 |
| M55914__MPB1 | 6.98895 | 8.97E-11 |
| NM_000485__APRT | 6.98692 | 9.07E-11 |
| NM_002466__MYBL2 | 6.98306 | 9.26E-11 |
| NM_004336__BUB1 | 6.9714 | 9.85E-11 |
| NM_001905__CTPS | 6.96046 | 1.04E-10 |
| NM_005507__CFL1 | 6.95958 | 1.05E-10 |
| NM_003158__STK6 | 6.95896 | 1.05E-10 |
| NM_016101__HSPC031 | 6.95814 | 1.06E-10 |
| NM_004674__ASH2L | 6.93724 | 1.18E-10 |
| Contig41413_RC | 6.92093 | 1.29E-10 |
| NM_016095__LOC51659 | 6.91946 | 1.30E-10 |
| NM_012474__UMPK | 6.90936 | 1.37E-10 |
| NM_003491__ARD1 | 6.90197 | 1.43E-10 |
| NM_013986__EWSR1 | 6.89559 | 1.48E-10 |
| Contig53180_RC | 6.88301 | 1.58E-10 |
| NM_018407__LC27 | 6.88255 | 1.58E-10 |
| NM_006351__TIM44 | 6.8811 | 1.59E-10 |
| Contig55258_RC | 6.87966 | 1.61E-10 |
| NM_014364__GAPDS | 6.84582 | 1.92E-10 |
| NM_014462__LSM1 | 6.83503 | 2.03E-10 |
| NM_005805__POH1 | 6.82949 | 2.10E-10 |
| NM_014754__PTDSS1 | 6.82948 | 2.10E-10 |
| AF067972__DNMT3A | 6.82328 | 2.17E-10 |
| NM_004096__EIF4EBP2 | 6.82009 | 2.20E-10 |
| NM_006082__K-ALPHA-1 | 6.81059 | 2.32E-10 |
| NM_004052__BNIP3 | 6.80491 | 2.39E-10 |
| Contig43741_RC | 6.79711 | 2.49E-10 |
| Contig40251_RC | 6.79684 | 2.49E-10 |
| NM_012341__NGB | 6.79477 | 2.52E-10 |
| NM_004184__WARS | 6.79441 | 2.52E-10 |
| NM_002346__LY6E | 6.78107 | 2.71E-10 |
| NM_004219__PTTG1 | 6.77583 | 2.78E-10 |
| NM_018101__FLJ10468 | 6.7757 | 2.78E-10 |
| NM_006739__MCM5 | 6.77522 | 2.79E-10 |
| NM_014064__AD-003 | 6.76567 | 2.93E-10 |
| NM_000268__NF2 | 6.76027 | 3.02E-10 |
| NM_005002__NDUFA9 | 6.75705 | 3.07E-10 |
| NM_020150__SAR1 | 6.75358 | 3.13E-10 |
| NM_003686__EXO1 | 6.75114 | 3.17E-10 |
| NM_004504__HRB | 6.74803 | 3.22E-10 |
| NM_004153__ORC1L | 6.74676 | 3.24E-10 |
| Contig25827_RC | 6.7457 | 3.26E-10 |
| Contig55188_RC | 6.74202 | 3.32E-10 |
| NM_018455__BM039 | 6.73836 | 3.39E-10 |
| NM_005371__METTL1 | 6.73322 | 3.48E-10 |
| NM_004027__INPP4A | 6.73219 | 3.50E-10 |
| NM_001360__DHCR7 | 6.71644 | 3.80E-10 |
| Contig1697_RC | 6.71615 | 3.81E-10 |
| NM_012310__KIF4A | 6.70672 | 4.00E-10 |
| NM_015922__H105E3 | 6.70541 | 4.03E-10 |
| Contig50841_RC | 6.69965 | 4.15E-10 |
| NM_003680__YARS | 6.69554 | 4.24E-10 |
| NM_002047__GARS | 6.69083 | 4.35E-10 |
| NM_005721__ACTR3 | 6.68582 | 4.46E-10 |
| NM_002394__SLC3A2 | 6.67916 | 4.62E-10 |
| Contig6498 | 6.6594 | 5.12E-10 |
| Contig57493_RC | 6.64559 | 5.51E-10 |
| AL160131 | 6.63967 | 5.68E-10 |
| NM_002794__PSMB2 | 6.63383 | 5.86E-10 |
| NM_006397__RNASEHI | 6.63162 | 5.92E-10 |
| NM_002957__RXRA | 6.6163 | 6.42E-10 |
| U81002 | 6.60134 | 6.94E-10 |
| AF025441__OIP5 | 6.59638 | 7.12E-10 |
| Contig59136_RC | 6.59338 | 7.23E-10 |
| NM_001916__CYC1 | 6.58363 | 7.61E-10 |
| D50914__KIAA0124 | 6.56722 | 8.28E-10 |
| NM_004231__ATP6S14 | 6.56635 | 8.32E-10 |
| NM_020313__LOC57019 | 6.56576 | 8.34E-10 |
| NM_002266__KPNA2 | 6.56162 | 8.53E-10 |
| NM_006023__D123 | 6.55861 | 8.66E-10 |
| NM_006429__CCT7 | 6.55783 | 8.70E-10 |
| NM_006579__EBP | 6.55704 | 8.73E-10 |
| Contig38288_RC | 6.55448 | 8.85E-10 |
| NM_019009__LOC54472 | 6.55384 | 8.88E-10 |
| NM_004526__MCM2 | 6.55117 | 9.00E-10 |
| NM_018310__FLJ11052 | 6.54923 | 9.09E-10 |
| Contig47519_RC__LOC51622 | 6.54252 | 9.41E-10 |
| NM_002936__RNASEH1 | 6.53706 | 9.68E-10 |
| Contig28947_RC | 6.52844 | 1.01E-09 |
| NM_013299__HSU79266 | 6.52689 | 1.02E-09 |
| Contig40573_RC | 6.52556 | 1.03E-09 |
| NM_005918__MDH2 | 6.52382 | 1.04E-09 |
| NM_006590__SAD1 | 6.5154 | 1.08E-09 |
| NM_004358__CDC25B | 6.51361 | 1.09E-09 |
| NM_006819__STIP1 | 6.50641 | 1.13E-09 |
| NM_012346__NUP62 | 6.50559 | 1.14E-09 |
| NM_002105__H2AFX | 6.49927 | 1.18E-09 |
| NM_004900__DJ742C19.2 | 6.49083 | 1.23E-09 |
| NM_001970__EIF5A | 6.48785 | 1.25E-09 |
| NM_004327__BCR | 6.47329 | 1.35E-09 |
| NM_018842__LOC55971 | 6.45893 | 1.45E-09 |
| NM_001605__AARS | 6.44205 | 1.58E-09 |
| Contig54187_RC | 6.44092 | 1.59E-09 |
| AF007157 | 6.43628 | 1.63E-09 |
| NM_003108__SOX11 | 6.43528 | 1.64E-09 |
| NM_004077__CS | 6.43345 | 1.65E-09 |
| NM_002358__MAD2L1 | 6.42768 | 1.70E-09 |
| Contig24907_RC | 6.417 | 1.80E-09 |
| Contig29727_RC | 6.41683 | 1.80E-09 |
| NM_015971__LOC51081 | 6.41316 | 1.83E-09 |
| NM_001188__BAK1 | 6.41187 | 1.85E-09 |
| NM_004642__DOC1 | 6.41106 | 1.85E-09 |
| NM_005744__ARIH1 | 6.40616 | 1.90E-09 |
| NM_003056__SLC19A1 | 6.39521 | 2.01E-09 |
| NM_002812__PSMD8 | 6.3936 | 2.03E-09 |
| NM_002689__POLA2 | 6.3841 | 2.13E-09 |
| AF151109 | 6.38405 | 2.13E-09 |
| NM_017613__DONSON | 6.37857 | 2.19E-09 |
| NM_020143__LOC56902 | 6.37607 | 2.22E-09 |
| NM_003579__RAD54L | 6.36806 | 2.31E-09 |
| NM_021000__PTTG3 | 6.3606 | 2.40E-09 |
| NM_018396__FLJ11350 | 6.35382 | 2.48E-09 |
| NM_014325__CORO1C | 6.35204 | 2.51E-09 |
| NM_004127__GPS1 | 6.34997 | 2.53E-09 |
| D14678__KNSL2 | 6.34887 | 2.55E-09 |
| NM_006070__TFG | 6.34745 | 2.57E-09 |
| NM_003276__TMPO | 6.34443 | 2.61E-09 |
| NM_004383__CSK | 6.3433 | 2.62E-09 |
| NM_013421__GGT1 | 6.33383 | 2.75E-09 |
| Contig40813_RC | 6.32888 | 2.82E-09 |
| NM_005483__CHAF1A | 6.32564 | 2.87E-09 |
| NM_003981__PRC1 | 6.32093 | 2.94E-09 |
| AF114818__SIAHBP1 | 6.31145 | 3.08E-09 |
| NM_006201__PCTK1 | 6.30576 | 3.17E-09 |
| NM_017522__LRP8 | 6.30396 | 3.20E-09 |
| NM_004154__P2RY6 | 6.2991 | 3.28E-09 |
| NM_000154__GALK1 | 6.29534 | 3.34E-09 |
| NM_012291__KIAA0165 | 6.2844 | 3.53E-09 |
| NM_005915__MCM6 | 6.28224 | 3.57E-09 |
| AK001166__FLJ11252 | 6.27657 | 3.68E-09 |
| NM_002882__RANBP1 | 6.25766 | 4.05E-09 |
| NM_016031__ELOVL1 | 6.257 | 4.06E-09 |
| NM_014175__HSPC145 | 6.25112 | 4.18E-09 |
| NM_019848__P3 | 6.23722 | 4.49E-09 |
| Contig52872_RC__FLJ12517 | 6.23458 | 4.55E-09 |
| NM_006693__CPSF4 | 6.2317 | 4.62E-09 |
| NM_018380__FLJ11282 | 6.2274 | 4.72E-09 |
| NM_003318__TTK | 6.22229 | 4.84E-09 |
| NM_004298__NUP155 | 6.22206 | 4.85E-09 |
| NM_014272__ADAMTS7 | 6.22052 | 4.88E-09 |
| NM_021039__S100A14 | 6.21887 | 4.92E-09 |
| NM_000175__GPI | 6.21493 | 5.02E-09 |
| NM_001686__ATP5B | 6.20967 | 5.16E-09 |
| NM_021095__SLC5A6 | 6.20865 | 5.18E-09 |
| Contig48842_RC | 6.20188 | 5.36E-09 |
| NM_001929__DGUOK | 6.19954 | 5.43E-09 |
| NM_001047__SRD5A1 | 6.19934 | 5.43E-09 |
| NM_004068__AP2M1 | 6.19671 | 5.51E-09 |
| NM_006442__DRAP1 | 6.19162 | 5.65E-09 |
| NM_005648__TCEB1 | 6.18883 | 5.73E-09 |
| NM_003662__PIR | 6.18651 | 5.80E-09 |
| NM_020187__DC12 | 6.18626 | 5.80E-09 |
| Contig42201_RC | 6.18411 | 5.87E-09 |
| NM_001806__CEBPG | 6.16196 | 6.56E-09 |
| NM_005439__MLF2 | 6.15844 | 6.67E-09 |
| Contig50004_RC | 6.15628 | 6.75E-09 |
| NM_019044__FLJ10996 | 6.15112 | 6.92E-09 |
| NM_003821__RIPK2 | 6.15079 | 6.93E-09 |
| Contig49270_RC__KIAA1553 | 6.14752 | 7.05E-09 |
| NM_004131__GZMB | 6.14716 | 7.06E-09 |
| NM_003035__SIL | 6.14043 | 7.30E-09 |
| NM_005733__RAB6KIFL | 6.13577 | 7.48E-09 |
| NM_015530__DKFZP434D156 | 6.13504 | 7.50E-09 |
| NM_005804__DDXL | 6.13397 | 7.54E-09 |
| NM_017702__FLJ20186 | 6.13125 | 7.65E-09 |
| NM_016306__LOC51726 | 6.13048 | 7.68E-09 |
| AL050374__DKFZP586C1619 | 6.12496 | 7.89E-09 |
| NM_004749__CPR2 | 6.11484 | 8.30E-09 |
| Contig66827_RC | 6.11296 | 8.38E-09 |
| AL137718 | 6.10828 | 8.58E-09 |
| NM_002916__RFC4 | 6.10619 | 8.67E-09 |
| Contig56843_RC__CCNB1 | 6.10288 | 8.81E-09 |
| NM_004499__HNRPAB | 6.09772 | 9.04E-09 |
| NM_012151__F8A | 6.09633 | 9.11E-09 |
| Contig42809_RC | 6.09364 | 9.23E-09 |
| NM_003548__H4F2 | 6.09064 | 9.37E-09 |
| NM_004553__NDUFS6 | 6.08801 | 9.49E-09 |
| NM_006406__AOE372 | 6.08275 | 9.74E-09 |
| Contig46218_RC | 6.08228 | 9.77E-09 |
| NM_018131__FLJ10540 | 6.06645 | 1.06E-08 |
| NM_006230__POLD2 | 6.05914 | 1.10E-08 |
| NM_001859__SLC31A1 | 6.05687 | 1.11E-08 |
| NM_005333__HCCS | 6.05095 | 1.14E-08 |
| NM_016426__GTSE1 | 6.03745 | 1.22E-08 |
| Contig56298_RC | 6.0322 | 1.25E-08 |
| NM_004559__NSEP1 | 6.03087 | 1.26E-08 |
| NM_018265__FLJ10901 | 6.03052 | 1.26E-08 |
| NM_005742__P5 | 6.02527 | 1.30E-08 |
| D86978__KIAA0225 | 6.02258 | 1.31E-08 |
| NM_005146__SART1 | 6.02166 | 1.32E-08 |
| NM_002635__SLC25A3 | 6.01794 | 1.34E-08 |
| NM_005563__LAP18 | 6.01542 | 1.36E-08 |
| NM_001211__BUB1B | 6.00814 | 1.41E-08 |
| NM_002106__H2AFZ | 6.00665 | 1.42E-08 |
| NM_016094__LOC51122 | 6.00549 | 1.43E-08 |
| NM_018622__PRO2207 | 5.99884 | 1.48E-08 |
| AF155120__UBE2V1 | 5.99868 | 1.48E-08 |
| NM_003213__TEAD4 | 5.99205 | 1.53E-08 |
| NM_001428__ENO1 | 5.99006 | 1.54E-08 |
| Contig28712_RC | 5.9877 | 1.56E-08 |
| NM_005620__S100A11 | 5.98586 | 1.58E-08 |
| NM_017447__YG81 | 5.98016 | 1.62E-08 |
| M94362__LMNB2 | 5.97296 | 1.68E-08 |
| NM_004222__DNJ3 | 5.97293 | 1.68E-08 |
| NM_020979__APS | 5.96579 | 1.74E-08 |
| AJ245416__LSM2 | 5.96298 | 1.76E-08 |
| NM_018846__SBBI26 | 5.95761 | 1.81E-08 |
| NM_004856__KNSL5 | 5.95037 | 1.88E-08 |
| NM_005192__CDKN3 | 5.94256 | 1.95E-08 |
| AJ271216__DPP3 | 5.93154 | 2.06E-08 |
| AF113132__PSA | 5.93012 | 2.07E-08 |
| NM_000918__P4HB | 5.92913 | 2.08E-08 |
| NM_002654__PKM2 | 5.92884 | 2.09E-08 |
| NM_020470__54TM | 5.92828 | 2.09E-08 |
| Contig48776_RC | 5.92687 | 2.11E-08 |
| NM_007051__FAF1 | 5.9199 | 2.18E-08 |
| NM_004049__BCL2A1 | 5.91708 | 2.21E-08 |
| NM_003481__USP5 | 5.91654 | 2.22E-08 |
| NM_004309__ARHGDIA | 5.91364 | 2.25E-08 |
| Y18643__METTL1 | 5.91264 | 2.26E-08 |
| NM_000100__CSTB | 5.91121 | 2.28E-08 |
| NM_003486__SLC7A5 | 5.90753 | 2.32E-08 |
| Contig38630_RC | 5.9062 | 2.33E-08 |
| NM_006383__KIP2 | 5.9053 | 2.34E-08 |
| NM_001320__CSNK2B | 5.90399 | 2.36E-08 |
| NM_003953__MPZL1 | 5.89656 | 2.44E-08 |
| NM_005005__NDUFB9 | 5.8953 | 2.46E-08 |
| NM_017803__FLJ20399 | 5.88802 | 2.55E-08 |
| AI091551_RC | 5.88292 | 2.61E-08 |
| NM_001536__HRMT1L2 | 5.87771 | 2.68E-08 |
| NM_003093__SNRPC | 5.87557 | 2.71E-08 |
| Contig55538_RC__BA395L14.2 | 5.87326 | 2.74E-08 |
| Contig42041_RC | 5.87209 | 2.76E-08 |
| NM_020467__LOC57228 | 5.86797 | 2.81E-08 |
| NM_002086__GRB2 | 5.86517 | 2.85E-08 |
| U74612__FOXM1 | 5.85946 | 2.93E-08 |
| NM_007057__ZWINT | 5.84739 | 3.11E-08 |
| NM_000593__ABCB2 | 5.84477 | 3.15E-08 |
| NM_002720__PPP4C | 5.84465 | 3.15E-08 |
| NM_002815__PSMD11 | 5.83682 | 3.27E-08 |
| NM_017882__FLJ20561 | 5.83396 | 3.32E-08 |
| NM_004147__DRG1 | 5.82643 | 3.44E-08 |
| NM_007103__NDUFV1 | 5.82036 | 3.55E-08 |
| NM_016732__RALY | 5.81434 | 3.65E-08 |
| NM_001861__COX4 | 5.81142 | 3.70E-08 |
| Contig31288_RC | 5.81116 | 3.71E-08 |
| AL162049__USP10 | 5.81016 | 3.73E-08 |
| NM_017414__USP18 | 5.80917 | 3.74E-08 |
| NM_013438__UBQLN1 | 5.80769 | 3.77E-08 |
| Contig51464_RC | 5.80646 | 3.79E-08 |
| NM_006302__GCS1 | 5.80494 | 3.82E-08 |
| Contig54563_RC__KNSL5 | 5.79985 | 3.92E-08 |
| AF129536__FBXO6 | 5.78276 | 4.25E-08 |
| D80007__KIAA0185 | 5.77581 | 4.40E-08 |
| X77588__ARD1 | 5.77475 | 4.42E-08 |
| NM_019037__FLJ20591 | 5.77416 | 4.44E-08 |
| NM_014251__SLC25A13 | 5.76979 | 4.53E-08 |
| NM_005548__KARS | 5.76768 | 4.58E-08 |
| Contig44909_RC | 5.76065 | 4.74E-08 |
| Contig45032_RC | 5.75676 | 4.83E-08 |
| NM_006826__YWHAQ | 5.74878 | 5.01E-08 |
| NM_018179__FLJ10688 | 5.74773 | 5.04E-08 |
| NM_002131__HMGIY | 5.74574 | 5.09E-08 |
| NM_002254__KIF3C | 5.74401 | 5.13E-08 |
| Contig55264_RC | 5.74363 | 5.14E-08 |
| NM_002079__GOT1 | 5.74024 | 5.23E-08 |
| NM_004990__MARS | 5.72846 | 5.53E-08 |
| NM_001071__TYMS | 5.72461 | 5.63E-08 |
| Contig21010_RC | 5.71496 | 5.90E-08 |
| NM_017779__FLJ20354 | 5.71103 | 6.01E-08 |
| NM_005651__TDO2 | 5.7092 | 6.07E-08 |
| NM_000688__ALAS1 | 5.70467 | 6.20E-08 |
| Contig50194_RC | 5.70375 | 6.23E-08 |
| NM_006086__TUBB4 | 5.70101 | 6.31E-08 |
| Contig44939_RC | 5.69693 | 6.44E-08 |
| NM_003449__ZNF173 | 5.69565 | 6.48E-08 |
| Contig28550_RC | 5.69561 | 6.48E-08 |
| NM_018685__ANLN | 5.68971 | 6.66E-08 |
| Contig52482_RC | 5.68591 | 6.79E-08 |
| NM_021259__M83 | 5.68255 | 6.90E-08 |
| NM_002768__PCOLN3 | 5.68205 | 6.91E-08 |
| NM_002801__PSMB10 | 5.68172 | 6.92E-08 |
| NM_014669__KIAA0095 | 5.67844 | 7.03E-08 |
| NM_005022__PFN1 | 5.67829 | 7.04E-08 |
| NM_001826__CKS1 | 5.6742 | 7.18E-08 |
| NM_004207__SLC16A3 | 5.67044 | 7.31E-08 |
| NM_001127__AP1B1 | 5.66654 | 7.45E-08 |
| Contig1022_RC | 5.66392 | 7.54E-08 |
| NM_017518__HSXQ28ORF | 5.6547 | 7.88E-08 |
| NM_001084__PLOD3 | 5.65378 | 7.91E-08 |
| NM_001363__DKC1 | 5.65213 | 7.98E-08 |
| NM_003345__UBE2I | 5.65156 | 8.00E-08 |
| AF052151__MTVR | 5.65141 | 8.00E-08 |
| NM_014390__p100 | 5.64963 | 8.07E-08 |
| NM_018052__FLJ10305 | 5.64948 | 8.08E-08 |
| NM_004456__EZH2 | 5.64922 | 8.09E-08 |
| NM_004579__MAP4K2 | 5.64616 | 8.21E-08 |
| X52882__TCP1 | 5.64408 | 8.29E-08 |
| NM_006607__PTTG2 | 5.63915 | 8.49E-08 |
| NM_005526__HSF1 | 5.63216 | 8.77E-08 |
| NM_002268__KPNA4 | 5.62968 | 8.88E-08 |
| AF238083__SPHK1 | 5.62556 | 9.06E-08 |
| AF090913__TMSB10 | 5.62048 | 9.28E-08 |
| NM_002691__POLD1 | 5.61983 | 9.31E-08 |
| NM_013260__HCNGP | 5.61945 | 9.32E-08 |
| NM_017669__FLJ20105 | 5.61888 | 9.35E-08 |
| Contig57482 | 5.61459 | 9.54E-08 |
| Contig51882_RC | 5.61401 | 9.57E-08 |
| NM_004671__PIASX-BETA | 5.61131 | 9.69E-08 |
| NM_005342__HMG4 | 5.61056 | 9.73E-08 |
| Contig45816_RC | 5.6048 | 1.00E-07 |
| Contig16101_RC | 5.60407 | 1.00E-07 |
| NM_004712__HGS | 5.59539 | 1.05E-07 |
| NM_020230__PPAN | 5.59495 | 1.05E-07 |
| NM_007198__PROSC | 5.58633 | 1.09E-07 |
| NM_003765__STX10 | 5.58591 | 1.09E-07 |
| Contig20217_RC | 5.58443 | 1.10E-07 |
| NM_002764__PRPS1 | 5.58295 | 1.11E-07 |
| NM_004461__FARSL | 5.5798 | 1.13E-07 |
| Contig36953_RC | 5.57864 | 1.13E-07 |
| Contig46516_RC | 5.57746 | 1.14E-07 |
| NM_018128__FLJ10534 | 5.57743 | 1.14E-07 |
| NM_005343__HRAS | 5.5719 | 1.17E-07 |
| R11316_RC | 5.57121 | 1.17E-07 |
| NM_014750__KIAA0008 | 5.57101 | 1.17E-07 |
| NM_006409__ARPC1A | 5.56616 | 1.20E-07 |
| NM_000342__SLC4A1 | 5.56593 | 1.20E-07 |
| NM_014302__SEC61G | 5.5593 | 1.24E-07 |
| AF100756__LOC51137 | 5.54986 | 1.30E-07 |
| NM_003564__TAGLN2 | 5.54694 | 1.32E-07 |
| NM_012417__RDGBB | 5.54199 | 1.35E-07 |
| NM_014452__DR6 | 5.53872 | 1.37E-07 |
| NM_016034__LOC51116 | 5.53748 | 1.38E-07 |
| AL050008__DKFZP564A063 | 5.52851 | 1.43E-07 |
| NM_000075__CDK4 | 5.52841 | 1.44E-07 |
| NM_004074__COX8 | 5.52351 | 1.47E-07 |
| NM_001419__ELAVL1 | 5.51897 | 1.50E-07 |
| NM_005745__DXS1357E | 5.51634 | 1.52E-07 |
| NM_020166__MCC-B | 5.51585 | 1.52E-07 |
| NM_018697__LOC55915 | 5.51441 | 1.53E-07 |
| NM_000819__GART | 5.51283 | 1.54E-07 |
| NM_013332__HIG2 | 5.50999 | 1.57E-07 |
| NM_006665__HPSE | 5.50954 | 1.57E-07 |
| Contig48806_RC | 5.50458 | 1.61E-07 |
| U37689__POLR2H | 5.50289 | 1.62E-07 |
| Contig37015_RC | 5.49916 | 1.65E-07 |
| NM_019056__FLJ20494 | 5.49748 | 1.66E-07 |
| NM_003544__H4FI | 5.49427 | 1.69E-07 |
| NM_006004__UQCRH | 5.49398 | 1.69E-07 |
| Contig43549_RC | 5.49366 | 1.69E-07 |
| NM_006012__CLPP | 5.49133 | 1.71E-07 |
| NM_004741__P130 | 5.49088 | 1.71E-07 |
| AL049943__DKFZP564F0522 | 5.48976 | 1.72E-07 |
| AF234532__MYO10 | 5.48812 | 1.73E-07 |
| NM_006303__JTV1 | 5.48526 | 1.76E-07 |
| NM_005662__VDAC3 | 5.47847 | 1.82E-07 |
| NM_005480__TROAP | 5.47645 | 1.83E-07 |
| NM_016062__LOC51647 | 5.47533 | 1.84E-07 |
| NM_017546__C40 | 5.47263 | 1.87E-07 |
| NM_015949__LOC51608 | 5.47203 | 1.87E-07 |
| U81599__HOXB13 | 5.47179 | 1.87E-07 |
| NM_004401__DFFA | 5.46828 | 1.90E-07 |
| NM_001694__ATP6L | 5.46724 | 1.91E-07 |
| NM_017870__FLJ20539 | 5.45647 | 2.01E-07 |
| Contig2099_RC | 5.45599 | 2.02E-07 |
| NM_018087__FLJ10407 | 5.45376 | 2.04E-07 |
| X75315__HSRNASEB | 5.4518 | 2.06E-07 |
| NM_014176__HSPC150 | 5.44522 | 2.12E-07 |
| AF054996 | 5.4439 | 2.13E-07 |
| NM_002610__PDK1 | 5.44286 | 2.14E-07 |
| Contig36879_RC | 5.43562 | 2.22E-07 |
| NM_021103__TMSB10 | 5.42237 | 2.36E-07 |
| NM_014317__TPT | 5.42043 | 2.38E-07 |
| NM_016352__LOC51200 | 5.41874 | 2.40E-07 |
| NM_002461__MVD | 5.41847 | 2.40E-07 |
| NM_005659__UFD1L | 5.41685 | 2.42E-07 |
| NM_000057__BLM | 5.41455 | 2.45E-07 |
| NM_006907__PYCR1 | 5.41311 | 2.46E-07 |
| NM_000636__SOD2 | 5.41185 | 2.48E-07 |
| Contig1295_RC__F25965 | 5.40378 | 2.57E-07 |
| NM_020365__EIF2B3 | 5.40374 | 2.57E-07 |
| NM_016577__RAB6B | 5.40029 | 2.62E-07 |
| NM_016183__LOC51154 | 5.39926 | 2.63E-07 |
| NM_006342__TACC3 | 5.39881 | 2.63E-07 |
| Contig55629 | 5.39877 | 2.63E-07 |
| NM_004269__CRSP8 | 5.39564 | 2.67E-07 |
| Contig51006_RC | 5.39467 | 2.68E-07 |
| NM_006833__MOV34-34KD | 5.38778 | 2.77E-07 |
| NM_006636__MTHFD2 | 5.38542 | 2.80E-07 |
| NM_015947__LOC51008 | 5.38375 | 2.82E-07 |
| NM_007367__RALY | 5.37955 | 2.88E-07 |
| NM_017760__FLJ20311 | 5.37728 | 2.91E-07 |
| NM_004515__ILF2 | 5.37495 | 2.94E-07 |
| NM_002790__PSMA5 | 5.37131 | 2.99E-07 |
| NM_015044__KIAA1080 | 5.36999 | 3.01E-07 |
| D25328__PFKP | 5.36859 | 3.03E-07 |
| NM_001152__SLC25A5 | 5.36452 | 3.09E-07 |
| NM_006170__NOL1 | 5.36404 | 3.09E-07 |
| NM_005174__ATP5C1 | 5.36241 | 3.12E-07 |
| NM_003191__TARS | 5.36096 | 3.14E-07 |
| NM_014050__PTD007 | 5.35845 | 3.18E-07 |
| NM_003678__PK1.3 | 5.35086 | 3.29E-07 |
| NM_004035__ACOX1 | 5.34931 | 3.31E-07 |
| NM_006854__KDELR2 | 5.3478 | 3.34E-07 |
| D21064__KIAA0123 | 5.34375 | 3.40E-07 |
| NM_004523__KNSL1 | 5.34209 | 3.43E-07 |
| NM_001101__ACTB | 5.3414 | 3.44E-07 |
| NM_013242__AF093680 | 5.33963 | 3.46E-07 |
| NM_001863__COX6B | 5.33297 | 3.57E-07 |
| AL157851__CGI-96 | 5.3299 | 3.62E-07 |
| NM_005880__HIRIP4 | 5.32917 | 3.64E-07 |
| Contig16298_RC | 5.32859 | 3.65E-07 |
| NM_002797__PSMB5 | 5.32689 | 3.67E-07 |
| Contig58329_RC | 5.32624 | 3.68E-07 |
| NM_000512__GALNS | 5.32581 | 3.69E-07 |
| NM_018332__FLJ11126 | 5.3255 | 3.70E-07 |
| AB006198__SART1 | 5.32155 | 3.77E-07 |
| NM_007006__CFIM25 | 5.3169 | 3.85E-07 |
| NM_001288__CLIC1 | 5.316 | 3.86E-07 |
| NM_003975__SH2D2A | 5.31131 | 3.95E-07 |
| NM_017660__FLJ20085 +428:445428:44742 + A3:C479428:906 | 5.30869 | 4.00E-07 |

**Table B. Pathways positively correlated to 4EBP1.**

| p-value | Term | Term ID | Term description | Genes |
| --- | --- | --- | --- | --- |
| 2.40e-02 | GO:0006094 | BP | gluconeogenesis | TPI1, PGK1, GAPD, GPI, ENO1, SLC25A13, GOT1 |
| 2.52e-02 | GO:0009165 | BP | nucleotide biosynthetic process | TK1, RRM2, ATP5G3, GMPS, APRT, GARS, ATP5B, DGUOK, PKM2, SLC25A13, KARS, TYMS, PRPS1, ATP5C1 |
| 1.11e-02 | GO:0016032 | BP | viral reproduction | CENPA, PSMD2, AP2S1, BUB1, BNIP3, PSMB2, RXRA, NUP62, PSMD8, RANBP1, CPSF4, NUP155, AP2M1, TCEB1, GRB2, PSMD11, KARS, TYMS, PSMB10, PFN1, AP1B1, UBE2I, POLR2H, PSMA5, SLC25A5, PSMB5 |
| 3.52e-19 | GO:0044710 | BP | single-organism metabolic process | EIF4EBP1, TRIP13, TK1, SHMT2, TPI1, BIRC5, PGK1, CCNB2, CDC20, COX5A, CCNA2, PDXK, E2F1, CENPA, MRPL37, RRM2, PPM1G, MCM7, PHGDH, ATP5G3, RAD51, MRPL12, PKMYT1, PFKL, PSMD2, GMPS, CHEK1, ASNS, SNRPA, IRAK1, HADH2, FEN1, WDR5, RPN1, CIAO1, SRM, IDH2, GAPD, EIF2C2, MAD2L2, EXO1, APRT, MYBL2, CFL1, ASH2L, EWSR1, LSM1, PTDSS1, DNMT3A, EIF4EBP2, BNIP3, WARS, LY6E, PTTG1, MCM5, NF2, NDUFA9, METTL1, INPP4A, DHCR7, YARS, GARS, SLC3A2, PSMB2, RXRA, OIP5, CYC1, CCT7, EBP, MCM2, RNASEH1, CDC25B, NUP62, H2AFX, EIF5A, BCR, AARS, SOX11, CS, MAD2L1, BAK1, ARIH1, SLC19A1, PSMD8, POLA2, RAD54L, GPS1, TMPO, CSK, CHAF1A, PCTK1, LRP8, GALK1, MCM6, ELOVL1, CPSF4, TTK, NUP155, ADAMTS7, GPI, ATP5B, SLC5A6, DGUOK, SRD5A1, DRAP1, TCEB1, PIR, CEBPG, RIPK2, GZMB, RFC4, CCNB1, NDUFS6, POLD2, HCCS, P5, SART1, BUB1B, H2AFZ, TEAD4, ENO1, S100A11, APS, CDKN3, DPP3, P4HB, PKM2, FAF1, USP5, SLC7A5, NDUFB9, SNRPC, GRB2, FOXM1, PPP4C, PSMD11, DRG1, NDUFV1, RALY, USP10, USP18, UBQLN1, FBXO6, SLC25A13, KARS, YWHAQ, GOT1, MARS, TYMS, ALAS1, PSMB10, PFN1, SLC16A3, PLOD3, DKC1, UBE2I, EZH2, MAP4K2, TCP1, PTTG2, SPHK1, POLD1, HGS, PPAN, PRPS1, SLC4A1, SEC61G, CDK4, COX8, ELAVL1, HPSE, POLR2H, UQCRH, CLPP, HOXB13, DFFA, PDK1, MVD, UFD1L, BLM, SOD2, EIF2B3, MTHFD2, ILF2, PSMA5, PFKP, SLC25A5, ATP5C1, TARS, ACOX1, PSMB5, GALNS |
| 2.56e-02 | GO:0021700 | BP | developmental maturation | TRIP13, CDC20, PSMD2, AP2S1, KIF4A, PSMB2, CDC25B, PSMD8, AP2M1, CCNB1, PSMD11, KIF3C, TYMS, PSMB10, AP1B1, SEC61G, HOXB13, PSMA5, PSMB5 |
| 4.87e-22 | GO:0044424 | CC | intracellular part | EIF4EBP1, TRIP13, TK1, SHMT2, TPI1, BIRC5, PGK1, CCNB2, MTCH2, CDC20, COX5A, CCNA2, PDXK, E2F1, CENPA, MRPL37, RRM2, PPM1G, MCM7, PHGDH, ATP5G3, SNN, RAD51, MRPL12, PKMYT1, BYSL, PFKL, PSMD2, GMPS, CHEK1, ASNS, SNRPA, IRAK1, AP2S1, HADH2, FEN1, WDR5, RPN1, CIAO1, SRM, IDH2, GAPD, EIF2C2, MAD2L2, EXO1, APRT, MYBL2, BUB1, CFL1, ASH2L, EWSR1, LSM1, PTDSS1, DNMT3A, BNIP3, NGB, WARS, PTTG1, MCM5, NF2, NDUFA9, METTL1, INPP4A, DHCR7, KIF4A, YARS, GARS, ACTR3, SLC3A2, PSMB2, RXRA, OIP5, CYC1, CCT7, EBP, MCM2, RNASEH1, CDC25B, STIP1, NUP62, H2AFX, EIF5A, BCR, AARS, SOX11, CS, MAD2L1, BAK1, ARIH1, PSMD8, POLA2, DONSON, RAD54L, CORO1C, GPS1, TFG, TMPO, CSK, CHAF1A, PRC1, PCTK1, GALK1, MCM6, RANBP1, ELOVL1, CPSF4, TTK, NUP155, S100A14, GPI, ATP5B, DGUOK, SRD5A1, AP2M1, DRAP1, TCEB1, PIR, CEBPG, MLF2, RIPK2, GZMB, RFC4, CCNB1, NDUFS6, POLD2, HCCS, GTSE1, P5, SART1, SLC25A3, BUB1B, H2AFZ, TEAD4, ENO1, S100A11, LMNB2, CDKN3, DPP3, P4HB, PKM2, FAF1, BCL2A1, USP5, ARHGDIA, CSTB, SLC7A5, NDUFB9, SNRPC, LOC57228, GRB2, FOXM1, ZWINT, PPP4C, PSMD11, DRG1, NDUFV1, RALY, USP10, USP18, UBQLN1, FBXO6, SLC25A13, KARS, YWHAQ, KIF3C, GOT1, MARS, TYMS, ALAS1, ANLN, PSMB10, PFN1, SLC16A3, AP1B1, PLOD3, DKC1, UBE2I, EZH2, MAP4K2, TCP1, PTTG2, KPNA4, SPHK1, TMSB10, POLD1, HGS, PPAN, PROSC, STX10, PRPS1, ARPC1A, SLC4A1, SEC61G, TAGLN2, CDK4, COX8, ELAVL1, HPSE, POLR2H, UQCRH, CLPP, MYO10, VDAC3, TROAP, HOXB13, DFFA, PDK1, MVD, UFD1L, BLM, SOD2, EIF2B3, TACC3, MTHFD2, ILF2, PSMA5, PFKP, SLC25A5, ATP5C1, TARS, ACOX1, KDELR2, PSMB5, GALNS, SH2D2A |
| 1.25e-02 | GO:0004812 | MF | aminoacyl-tRNA ligase activity | WARS, YARS, GARS, AARS, KARS, MARS, TARS |
| 4.02e-12 | GO:0005515 | MF | protein binding | EIF4EBP1, TRIP13, SHMT2, BIRC5, CCNB2, CDC20, CCNA2, PDXK, E2F1, CENPA, RRM2, PPM1G, MCM7, RAD51, MRPL12, PKMYT1, BYSL, PFKL, PSMD2, CHEK1, ASNS, SNRPA, IRAK1, HADH2, FEN1, WDR5, SRM, GAPD, EIF2C2, MAD2L2, EXO1, BUB1, CFL1, ASH2L, EWSR1, LSM1, DNMT3A, EIF4EBP2, BNIP3, WARS, PTTG1, MCM5, NF2, NDUFA9, METTL1, YARS, GARS, ACTR3, SLC3A2, RXRA, OIP5, CCT7, MCM2, RNASEH1, CDC25B, STIP1, NUP62, H2AFX, EIF5A, BCR, MAD2L1, BAK1, ARIH1, POLA2, RAD54L, CORO1C, TFG, TMPO, CSK, CHAF1A, PRC1, PCTK1, LRP8, MCM6, RANBP1, ELOVL1, S100A14, GPI, ATP5B, AP2M1, DRAP1, TCEB1, PIR, CEBPG, RIPK2, GZMB, RFC4, CCNB1, POLD2, P5, SART1, BUB1B, H2AFZ, TEAD4, ENO1, S100A11, APS, CDKN3, P4HB, PKM2, FAF1, BCL2A1, USP5, ARHGDIA, CSTB, MPZL1, SNRPC, GRB2, FOXM1, ZWINT, PPP4C, DRG1, USP10, UBQLN1, FBXO6, YWHAQ, ANLN, PFN1, AP1B1, PLOD3, DKC1, UBE2I, EZH2, MAP4K2, TCP1, PTTG2, SPHK1, TMSB10, POLD1, HGS, STX10, PRPS1, ARPC1A, SLC4A1, TAGLN2, CDK4, ELAVL1, HPSE, UQCRH, CLPP, MYO10, TROAP, PDK1, MVD, UFD1L, BLM, SOD2, EIF2B3, TACC3, ILF2, PSMA5, SLC25A5, TARS, ACOX1, PSMB5, SH2D2A |
| 1.04e-23 | BIOGRID:00000 | bi | BioGRID interaction data | EIF4EBP1, TRIP13, TK1, SHMT2, TPI1, BIRC5, PGK1, CCNB2, MTCH2, CDC20, COX5A, CCNA2, PDXK, E2F1, CENPA, MRPL37, ZNF313, RRM2, PPM1G, MCM7, PHGDH, ATP5G3, RAD51, MRPL12, PKMYT1, BYSL, PFKL, PSMD2, GMPS, CHEK1, ASNS, SNRPA, IRAK1, AP2S1, HADH2, FEN1, WDR5, RPN1, CIAO1, SRM, IDH2, GAPD, EIF2C2, MAD2L2, EXO1, APRT, MYBL2, BUB1, CFL1, ASH2L, EWSR1, LSM1, PTDSS1, DNMT3A, EIF4EBP2, BNIP3, NGB, WARS, PTTG1, MCM5, NF2, NDUFA9, METTL1, INPP4A, DHCR7, KIF4A, YARS, GARS, ACTR3, SLC3A2, PSMB2, RXRA, OIP5, CYC1, CCT7, EBP, MCM2, CDC25B, STIP1, NUP62, H2AFX, EIF5A, BCR, AARS, CS, MAD2L1, BAK1, ARIH1, SLC19A1, PSMD8, POLA2, DONSON, RAD54L, CORO1C, GPS1, TFG, TMPO, CSK, CHAF1A, PRC1, PCTK1, LRP8, GALK1, MCM6, RANBP1, ELOVL1, CPSF4, TTK, NUP155, S100A14, GPI, ATP5B, SLC5A6 |
| 5.00e-02 | CORUM:190 | co | mitotic checkpoint complex (MCC) | CDC20, MAD2L1, BUB1B |
| 1.28e-02 | CORUM:1452 | co | MCM2-MCM6-MCM7 complex | MCM7, MCM2, MCM6 |
| 6.34e-03 | CORUM:387 | co | MCM complex | MCM7, MCM5, MCM2, MCM6 |
| 5.00e-02 | CORUM:2792 | co | MCM2-MCM4-MCM6-MCM7 complex | MCM7, MCM2, MCM6 |
| 4.70e-02 | HP:0002936 | hp | distal sensory impairment | YARS, GARS, AARS, TFG, KARS, PRPS1 |
| 5.76e-11 | KEGG:04110 | ke | cell cycle | CCNB2, CDC20, CCNA2, E2F1, MCM7, PKMYT1, CHEK1, MAD2L2, BUB1, PTTG1, MCM5, MCM2, CDC25B, MAD2L1, MCM6, TTK, CCNB1, BUB1B, YWHAQ, PTTG2, CDK4 |
| 2.31e-04 | KEGG:05016 | ke | Huntington's disease | COX5A, ATP5G3, AP2S1, NDUFA9, CYC1, ATP5B, AP2M1, NDUFS6, NDUFB9, NDUFV1, COX8, POLR2H, UQCRH, VDAC3, SOD2, SLC25A5, ATP5C1 |
| 1.35e-02 | KEGG:00970 | ke | Aminoacyl-tRNA biosynthesis | WARS, YARS, GARS, AARS, KARS, MARS, TARS |
| 2.11e-02 | KEGG:04114 | ke | oocyte meiosis | CCNB2, CDC20, PKMYT1, MAD2L2, BUB1, PTTG1, MAD2L1, CCNB1, YWHAQ, PTTG2 |
| 3.13e-02 | KEGG:05010 | ke | Alzheimer's disease | COX5A, ATP5G3, HADH2, GAPD, NDUFA9, CYC1, ATP5B, NDUFS6, NDUFB9, NDUFV1, COX8, UQCRH, ATP5C1 |
| 3.63e-02 | KEGG:03440 | ke | homologous recombination | RAD51, RAD54L, POLD2, POLD1, BLM |
| 3.34e-02 | KEGG:03050 | ke | proteasome | PSMD2, PSMB2, PSMD8, PSMD11, PSMB10, PSMA5, PSMB5 |
| 5.44e-07 | KEGG:03030 | ke | DNA replication | MCM7, FEN1, MCM5, MCM2, RNASEH1, POLA2, MCM6, RFC4, POLD2, POLD1 |
| 1.11e-02 | KEGG:00010 | ke | glycolysis / gluconeogenesis | TPI1, PGK1, PFKL, GAPD, GPI, ENO1, PKM2, PFKP |
| 4.03e-04 | KEGG:01100 | ke | metabolic pathways | TK1, SHMT2, TPI1, PGK1, COX5A, PDXK, RRM2, PHGDH, ATP5G3, PFKL, GMPS, ASNS, HADH2, RPN1, SRM, IDH2, GAPD, APRT, PTDSS1, DNMT3A, NDUFA9, INPP4A, DHCR7, CYC1, EBP, CS, POLA2, GALK1, GPI, ATP5B, DGUOK, NDUFS6, POLD2, ENO1, PKM2, NDUFB9, NDUFV1, GOT1, TYMS, ALAS1, SPHK1, POLD1, PRPS1, COX8, HPSE, POLR2H, UQCRH, MVD, MTHFD2, PFKP, ATP5C1, ACOX1, GALNS |
| 1.79e-03 | KEGG:05012 | ke | Parkinson's disease | COX5A, ATP5G3, NDUFA9, CYC1, ATP5B, NDUFS6, NDUFB9, NDUFV1, COX8, UQCRH, VDAC3, SLC25A5, ATP5C1 |
| 8.68e-05 | MI:hsa-miR-423-5p | mi | MI:hsa-miR-423-5p | EIF4EBP1, PPM1G, PKMYT1, AP2S1, HADH2, RPN1, SRM, IDH2, MYBL2, MCM5, METTL1, SLC3A2, MCM2, P2RY6, GPI, ATP5B, DGUOK, AP2M1, DRAP1, PKM2, FAF1, USP5, FOXM1, PPP4C, HGS, PPAN, COX8 |
| 2.83e-02 | REAC:68954 | re | Mcm2-7 is phosphorylated by DDK | MCM7, MCM5, MCM2, MCM6 |
| 2.48e-02 | REAC:162909 | re | host Interactions of HIV factors | PSMD2, AP2S1, PSMB2, NUP62, PSMD8, RANBP1, NUP155, AP2M1, TCEB1, PSMD11, PSMB10, AP1B1, PSMA5, SLC25A5, PSMB5 |
| 9.68e-03 | REAC:379716 | re | cytosolic tRNA aminoacylation | WARS, YARS, GARS, AARS, KARS, MARS, TARS |
| 2.05e-07 | REAC:69278 | re | cell cycle, mitotic | BIRC5, CCNB2, CDC20, CCNA2, E2F1, CENPA, RRM2, MCM7, PKMYT1, PSMD2, FEN1, BUB1, PTTG1, MCM5, PSMB2, MCM2, CDC25B, MAD2L1, PSMD8, POLA2, MCM6, RFC4, CCNB1, POLD2, BUB1B, ZWINT, PSMD11, TYMS, PSMB10, POLD1, CDK4, PSMA5, PSMB5 |
| 1.35e-04 | REAC:265764 | re | glucose regulation of insulin secretion | TPI1, PGK1, COX5A, PFKL, GAPD, NDUFA9, CYC1, CS, GPI, ATP5B, NDUFS6, ENO1, PKM2, NDUFB9, NDUFV1, COX8, UQCRH, PFKP, SLC25A5, ATP5C1 |
| 6.39e-03 | REAC:169468 | re | MCM2-7 mediated fork unwinding | MCM7, MCM5, MCM2, MCM6 |
| 2.54e-05 | REAC:69620 | re | cell cycle checkpoints | CCNB2, CDC20, MCM7, PSMD2, CHEK1, MCM5, PSMB2, MCM2, MAD2L1, PSMD8, MCM6, RFC4, CCNB1, BUB1B, PSMD11, PSMB10, PSMA5, PSMB5 |
| 1.50e-02 | REAC:69273 | re | cyclin A/B1 associated events during G2/M transition | CCNB2, CCNA2, PKMYT1, CDC25B, CCNB1 |
| 4.97e-02 | REAC:176942 | re | multiple proteins are localized at replication fork | MCM7, MCM5, MCM2, MCM6 |
| 1.13e-02 | TF:M00931_2 | tf | Factor: Sp1; motif: GGGGCGGGGC; match class: 2 | EIF4EBP1, MTCH2, CDC20, CCNA2, PDXK, E2F1, ZNF313, MCM7, SNN, BYSL, PFKL, PSMD2, GMPS, CHEK1, ASNS, SNRPA, WDR5, CIAO1, SRM, IDH2, EIF2C2, MAD2L2, EXO1, APRT, MYBL2, EIF4EBP2, BNIP3, WARS, LY6E, NF2, INPP4A, DHCR7, KIF4A, YARS, GARS, SLC3A2, RXRA, CYC1, CCT7, EBP, RNASEH1, STIP1, NUP62, EIF5A, AARS, SOX11, ARIH1, SLC19A1, RAD54L, CORO1C, GPS1, TFG, PRC1, PCTK1, LRP8, GALK1, MCM6, ELOVL1, ADAMTS7, GPI, SLC5A6, DRAP1, MLF2, RFC4, CCNB1, NDUFS6, POLD2, P5, TEAD4, ENO1, S100A11, DPP3, PKM2, CSTB, SLC7A5, SNRPC, GRB2, DRG1, RALY, UBQLN1, KARS, KIF3C, ALAS1, ANLN, PSMB10, PFN1, SLC16A3, AP1B1, UBE2I, EZH2, MAP4K2, TCP1, KPNA4, SPHK1, TMSB10, POLD1, HGS, PPAN, TAGLN2, POLR2H, MYO10, MVD, BLM, SOD2, EIF2B3, TACC3, PFKP, SLC25A5, ATP5C1, KDELR2, GALNS |
| 2.64e-05 | TF:M00940_3 | tf | Factor: E2F-1; motif: NTTTCGCGCS; match class: 3 | SHMT2, PDXK, E2F1, CENPA, MRPL37, MCM7, MRPL12, BYSL, CHEK1, CIAO1, GAPD, MAD2L2, EXO1, PTDSS1, MCM5, NF2, GARS, OIP5, CDC25B, SOX11, POLA2, DONSON, MCM6, RANBP1, TTK, NUP155, SLC5A6, NDUFS6, SLC31A1, FOXM1, ZWINT, TCP1, SPHK1, VDAC3, TARS |
| 2.54e-03 | TF:M00050_0 | tf | Factor: E2F; motif: TTTSGCGC; match class: 0 | SHMT2, PDXK, E2F1, MRPL37, MCM7, MRPL12, EXO1, MCM5, GARS, OIP5, POLA2, DONSON, MCM6, NUP155, SLC5A6, NDUFS6, FOXM1, TCP1, SPHK1, COX8 |
| 3.67e-03 | TF:M00196_4 | tf | Factor: Sp1; motif: NGGGGGCGGGGYN; match class: 4 | EIF4EBP1, TRIP13, TPI1, MTCH2, CDC20, COX5A, CCNA2, PDXK, E2F1, ZNF313, PPM1G, MCM7, SNN, BYSL, PFKL, PSMD2, GMPS, CHEK1, ASNS, SNRPA, IRAK1, WDR5, CIAO1, SRM, IDH2, EIF2C2, MAD2L2, EXO1, APRT, MYBL2, LSM1, PTDSS1, EIF4EBP2, BNIP3, WARS, LY6E, NF2, INPP4A, DHCR7, KIF4A, YARS, GARS, SLC3A2, RXRA, CYC1, CCT7, EBP, RNASEH1, STIP1, NUP62, EIF5A, AARS, SOX11, CS, ARIH1, SLC19A1, DONSON, RAD54L, CORO1C, GPS1, TFG, TMPO, PRC1, PCTK1, LRP8, GALK1, MCM6, RANBP1, ELOVL1, ADAMTS7, GPI, SLC5A6, DRAP1, MLF2, RFC4, CCNB1, NDUFS6, POLD2, HCCS, P5, TEAD4, ENO1, S100A11, DPP3, P4HB, PKM2, CSTB, SLC7A5, SNRPC, LOC57228, GRB2, PSMD11, DRG1, RALY, UBQLN1, KARS, YWHAQ, KIF3C, GOT1, ALAS1, ANLN, PSMB10, PFN1, SLC16A3, AP1B1, UBE2I, EZH2, MAP4K2, TCP1, KPNA4, SPHK1, TMSB10, POLD1, HGS, PPAN, PRPS1, TAGLN2, POLR2H, MYO10, PDK1, MVD, BLM, SOD2, EIF2B3, TACC3, PFKP, SLC25A5, ATP5C1, ACOX1, KDELR2, AF093680, GALNS |
| 6.45e-07 | TF:M00428_4 | tf | Factor: E2F-1; motif: NKTSSCGC; match class: 4 | TRIP13, TK1, SHMT2, TPI1, PGK1, CDC20, COX5A, PDXK, E2F1, CENPA, MRPL37, ZNF313, RRM2, PPM1G, MCM7, ATP5G3, SNN, MRPL12, PKMYT1, BYSL, PFKL, PSMD2, GMPS, CHEK1, ASNS, SNRPA, AP2S1, FEN1, WDR5, CIAO1, SRM, IDH2, GAPD, EIF2C2, MAD2L2, EXO1, APRT, MYBL2, ASH2L, EWSR1, LSM1, PTDSS1, DNMT3A, BNIP3, WARS, LY6E, MCM5, NF2, NDUFA9, METTL1, INPP4A, DHCR7, KIF4A, YARS, GARS, SLC3A2, PSMB2, OIP5, CYC1, EBP, RNASEH1, CDC25B, STIP1, H2AFX, AARS, SOX11, CS, MAD2L1, BAK1, ARIH1, SLC19A1, POLA2, DONSON, RAD54L, CORO1C, GPS1, TMPO, CSK, CHAF1A, PRC1, PCTK1, P2RY6, GALK1, MCM6, RANBP1, TTK, NUP155, ADAMTS7, SLC5A6, SRD5A1, DRAP1, PIR, CEBPG, RIPK2, RFC4, CCNB1, NDUFS6, POLD2, SLC31A1, GTSE1, SART1, SLC25A3, BUB1B, H2AFZ, TEAD4, ENO1, S100A11, APS, DPP3, P4HB, PKM2, FAF1, USP5, CSTB, SLC7A5, NDUFB9, SNRPC, LOC57228, FOXM1, ZWINT, PPP4C, DRG1, NDUFV1, RALY, USP18, UBQLN1, KARS, GOT1, TYMS, ANLN, PFN1, PLOD3, DKC1, UBE2I, EZH2, TCP1, SPHK1, HGS, PPAN, PRPS1, ARPC1A, SEC61G, TAGLN2, COX8, ELAVL1, POLR2H, UQCRH, CLPP, MYO10, VDAC3, TROAP, DFFA, PDK1, MVD, UFD1L, BLM, SOD2, EIF2B3, TACC3, MTHFD2, ILF2, PSMA5, PFKP, ATP5C1, TARS, AF093680, GALNS |
| 4.34e-02 | TF:M00516_2 | tf | Factor: E2F; motif: TTTSGCGCGMNR; match class: 2 | E2F1, MCM7, EXO1, MCM5, MCM6, NUP155, SLC5A6, COX8 |
| 1.23e-06 | TF:M00803_0 | tf | Factor: E2F; motif: GGCGSG; match class: 0 | EIF4EBP1, TRIP13, TK1, BIRC5, PGK1, MTCH2, COX5A, PDXK, E2F1, ZNF313, RRM2, MCM7, ATP5G3, SNN, RAD51, MRPL12, PKMYT1, PFKL, PSMD2, CHEK1, ASNS, IRAK1, AP2S1, HADH2, WDR5, RPN1, CIAO1, SRM, IDH2, GAPD, EIF2C2, MAD2L2, EXO1, APRT, MYBL2, ASH2L, EWSR1, PTDSS1, DNMT3A, BNIP3, WARS, LY6E, MCM5, NF2, METTL1, INPP4A, DHCR7, KIF4A, GARS, PSMB2, RXRA, OIP5, CYC1, EBP, MCM2, RNASEH1, CDC25B, STIP1, NUP62, H2AFX, EIF5A, AARS, SOX11, CS, MAD2L1, BAK1, SLC19A1, POLA2, CORO1C, GPS1, TMPO, CSK, CHAF1A, PRC1, PCTK1, LRP8, GALK1, MCM6, RANBP1, CPSF4, NUP155, GPI, ATP5B, SLC5A6, SRD5A1, AP2M1, DRAP1, MLF2, RIPK2, RFC4, CCNB1, POLD2, GTSE1, SART1, SLC25A3, H2AFZ, TEAD4, ENO1, S100A11, LMNB2, CDKN3, DPP3, P4HB, FAF1, ARHGDIA, CSTB, SLC7A5, NDUFB9, SNRPC, LOC57228, ZWINT, PPP4C, PSMD11, DRG1, NDUFV1, RALY, USP10, USP18, UBQLN1, FBXO6, SLC25A13, KARS, YWHAQ, KIF3C, GOT1, MARS, TYMS, ALAS1, ANLN, PFN1, SLC16A3, PLOD3, DKC1, UBE2I, EZH2, MAP4K2, TCP1, KPNA4, SPHK1, TMSB10, POLD1, HGS, PROSC, PRPS1, ARPC1A, SEC61G, COX8, ELAVL1, POLR2H, UQCRH, CLPP, MYO10, VDAC3, TROAP, DFFA, PDK1, MVD, SOD2, EIF2B3, TACC3, MTHFD2, PFKP, SLC25A5, ATP5C1, ACOX1, KDELR2, AF093680, PSMB5 |
| 3.63e-05 | TF:M00427_1 | tf | Factor: E2F; motif: TTTSGCGS; match class: 1 | TK1, SHMT2, PDXK, E2F1, MRPL37, RRM2, MCM7, MRPL12, CHEK1, AP2S1, EXO1, ASH2L, PTDSS1, MCM5, DHCR7, GARS, OIP5, CDC25B, SOX11, POLA2, DONSON, CHAF1A, MCM6, RANBP1, NUP155, SLC5A6, NDUFS6, ENO1, FOXM1, TCP1, SPHK1, COX8, VDAC3, PDK1, UFD1L, BLM |
| 1.52e-03 | TF:M00187_4 | tf | Factor: USF; motif: GYCACGTGNC; match class: 4 | MTCH2, RRM2, MCM7, SNN, PFKL, SNRPA, SRM, EIF2C2, MYBL2, EWSR1, DNMT3A, BNIP3, DHCR7, RXRA, MCM2, STIP1, SOX11, SLC19A1, RAD54L, GPS1, TFG, CHAF1A, RANBP1, ELOVL1, GPI, SRD5A1, RFC4, NDUFS6, H2AFZ, APS, DPP3, ARHGDIA, SLC7A5, FOXM1, NDUFV1, SLC25A13, ANLN, PSMB10, KPNA4, SLC4A1, CDK4, MVD, BLM, MTHFD2, ACOX1, GALNS |
| 3.63e-05 | TF:M00426_2 | tf | Factor: E2F; motif: TTTSGCGS; match class: 2 | TK1, SHMT2, PDXK, E2F1, MRPL37, RRM2, MCM7, MRPL12, CHEK1, AP2S1, EXO1, ASH2L, PTDSS1, MCM5, DHCR7, GARS, OIP5, CDC25B, SOX11, POLA2, DONSON, CHAF1A, MCM6, RANBP1, NUP155, SLC5A6, NDUFS6, ENO1, FOXM1, TCP1, SPHK1, COX8, VDAC3, PDK1, UFD1L, BLM |
| 1.20e-03 | TF:M00516_4 | tf | Factor: E2F; motif: TTTSGCGCGMNR; match class: 4 | SHMT2, E2F1, MRPL37, MCM7, MRPL12, EXO1, MCM5, GARS, OIP5, DONSON, MCM6, NUP155, SLC5A6, TCP1, SPHK1, COX8 |
| 2.26e-02 | TF:M00931_0 | tf | Factor: Sp1; motif: GGGGCGGGGC; match class: 0 | EIF4EBP1, MTCH2, CCNA2, PDXK, E2F1, ZNF313, SNN, BYSL, PFKL, GMPS, CHEK1, ASNS, WDR5, SRM, IDH2, APRT, MYBL2, EIF4EBP2, BNIP3, WARS, INPP4A, SLC3A2, RXRA, CYC1, CCT7, EBP, RNASEH1, STIP1, NUP62, SOX11, ARIH1, RAD54L, CORO1C, GPS1, GALK1, MCM6, ADAMTS7, GPI, SLC5A6, DRAP1, MLF2, CCNB1, P5, TEAD4, ENO1, S100A11, CSTB, SLC7A5, SNRPC, GRB2, KARS, KIF3C, ALAS1, PFN1, SLC16A3, AP1B1, UBE2I, EZH2, MAP4K2, TMSB10, POLD1, PPAN, TAGLN2, MYO10, SOD2, EIF2B3, KDELR2, GALNS |
| 3.47e-06 | TF:M00431_4 | tf | Factor: E2F-1; motif: TTTSGCGS; match class: 4 | SHMT2, TPI1, PGK1, CDC20, PDXK, E2F1, CENPA, MRPL37, RRM2, PPM1G, MCM7, ATP5G3, MRPL12, BYSL, CHEK1, ASNS, FEN1, CIAO1, SRM, GAPD, MAD2L2, EXO1, PTDSS1, BNIP3, WARS, MCM5, NF2, METTL1, INPP4A, YARS, GARS, SLC3A2, OIP5, EBP, CDC25B, SOX11, CS, MAD2L1, POLA2, DONSON, TMPO, CSK, CHAF1A, PCTK1, P2RY6, MCM6, RANBP1, TTK, NUP155, SLC5A6, SRD5A1, DRAP1, CCNB1, NDUFS6, SLC31A1, SART1, BUB1B, H2AFZ, DPP3, SLC7A5, LOC57228, FOXM1, ZWINT, NDUFV1, UBQLN1, KARS, TYMS, PFN1, TCP1, SPHK1, HGS, PPAN, PRPS1, COX8, UQCRH, VDAC3, TROAP, DFFA, TACC3, MTHFD2, PFKP, ATP5C1, TARS, GALNS |
| 2.78e-04 | TF:M00939_3 | tf | Factor: E2F-1; motif: TTTSGCGSG; match class: 3 | SHMT2, E2F1, MRPL37, RRM2, MRPL12, CHEK1, AP2S1, EXO1, MCM5, OIP5, POLA2, DONSON, CHAF1A, MCM6, RANBP1, NUP155, SLC5A6, NDUFS6, SPHK1, COX8, UFD1L |
| 2.54e-03 | TF:M00740_0 | tf | Factor: Rb:E2F-1:DP-1; motif: TTTSGCGC; match class: 0 | SHMT2, PDXK, E2F1, MRPL37, MCM7, MRPL12, EXO1, MCM5, GARS, OIP5, POLA2, DONSON, MCM6, NUP155, SLC5A6, NDUFS6, FOXM1, TCP1, SPHK1, COX8 |
| 1.15e-09 | TF:M00008_4 | tf | Factor: Sp1; motif: GGGGCGGGGT; match class: 4 | EIF4EBP1, TRIP13, TK1, TPI1, BIRC5, PGK1, MTCH2, CDC20, CCNA2, PDXK, E2F1, MRPL37, ZNF313, RRM2, PPM1G, MCM7, ATP5G3, SNN, RAD51, MRPL12, BYSL, PFKL, PSMD2, GMPS, CHEK1, ASNS, SNRPA, IRAK1, WDR5, CIAO1, SRM, IDH2, GAPD, EIF2C2, MAD2L2, EXO1, APRT, MYBL2, BUB1, CFL1, LSM1, PTDSS1, DNMT3A, EIF4EBP2, BNIP3, WARS, LY6E, PTTG1, NF2, METTL1, INPP4A, DHCR7, KIF4A, YARS, GARS, ACTR3, SLC3A2, RXRA, OIP5, CYC1, CCT7, EBP, RNASEH1, CDC25B, STIP1, NUP62, EIF5A, BCR, AARS, SOX11, CS, MAD2L1, ARIH1, SLC19A1, PSMD8, DONSON, RAD54L, CORO1C, GPS1, TFG, TMPO, CSK, PRC1, PCTK1, LRP8, P2RY6, GALK1, MCM6, RANBP1, ELOVL1, CPSF4, TTK, NUP155, ADAMTS7, S100A14, GPI, SLC5A6, DGUOK, DRAP1, CEBPG, MLF2, RIPK2, RFC4, CCNB1, NDUFS6, POLD2, HCCS, GTSE1, P5, SART1, H2AFZ, TEAD4, ENO1, S100A11, CDKN3, DPP3, PKM2, FAF1, ARHGDIA, CSTB, SLC7A5, MPZL1, SNRPC, GRB2, FOXM1, PSMD11, DRG1, NDUFV1, RALY, UBQLN1, KARS, YWHAQ, KIF3C, MARS, TYMS, ALAS1, ANLN, PSMB10, PFN1, SLC16A3, AP1B1, DKC1, UBE2I, EZH2, MAP4K2, TCP1, KPNA4, SPHK1, TMSB10, POLD1, HGS, PPAN, PRPS1, SEC61G, TAGLN2, ELAVL1, POLR2H, CLPP, MYO10, TROAP, HOXB13, PDK1, MVD, UFD1L, BLM, SOD2, EIF2B3, TACC3, MTHFD2, PSMA5, PFKP, SLC25A5, ATP5C1, ACOX1, KDELR2, AF093680, GALNS, SH2D2A |
| 2.78e-04 | TF:M00918_2 | tf | Factor: E2F; motif: TTTSGCGSG; match class: 2 | SHMT2, E2F1, MRPL37, RRM2, MRPL12, CHEK1, AP2S1, EXO1, MCM5, OIP5, POLA2, DONSON, CHAF1A, MCM6, RANBP1, NUP155, SLC5A6, NDUFS6, SPHK1, COX8, UFD1L |
| 4.21e-03 | TF:M00932_4 | tf | Factor: Sp1; motif: NNGGGGCGGGGNN; match class: 4 | EIF4EBP1, TRIP13, TPI1, MTCH2, CDC20, COX5A, CCNA2, PDXK, E2F1, ZNF313, PPM1G, MCM7, SNN, BYSL, PFKL, PSMD2, GMPS, CHEK1, ASNS, SNRPA, IRAK1, AP2S1, WDR5, CIAO1, SRM, IDH2, GAPD, EIF2C2, MAD2L2, EXO1, APRT, MYBL2, CFL1, PTDSS1, DNMT3A, EIF4EBP2, BNIP3, WARS, LY6E, NF2, INPP4A, DHCR7, KIF4A, YARS, GARS, SLC3A2, RXRA, CYC1, CCT7, EBP, RNASEH1, STIP1, NUP62, EIF5A, AARS, SOX11, CS, ARIH1, SLC19A1, RAD54L, CORO1C, GPS1, TFG, TMPO, CSK, PRC1, PCTK1, LRP8, GALK1, MCM6, RANBP1, ELOVL1, ADAMTS7, GPI, SLC5A6, DRAP1, MLF2, RFC4, CCNB1, NDUFS6, POLD2, P5, TEAD4, ENO1, S100A11, DPP3, P4HB, PKM2, CSTB, SLC7A5, SNRPC, LOC57228, GRB2, PSMD11, DRG1, RALY, UBQLN1, KARS, KIF3C, ALAS1, ANLN, PSMB10, PFN1, SLC16A3, AP1B1, UBE2I, EZH2, MAP4K2, TCP1, KPNA4, SPHK1, TMSB10, POLD1, HGS, PPAN, PRPS1, TAGLN2, POLR2H, UQCRH, MYO10, HOXB13, PDK1, MVD, BLM, SOD2, EIF2B3, TACC3, PSMA5, PFKP, SLC25A5, ATP5C1, ACOX1, KDELR2, GALNS |
| 5.58e-04 | TF:M00919_4 | tf | Factor: E2F; motif: NCSCGCSAAAN; match class: 4 | SHMT2, E2F1, MRPL37, RRM2, MRPL12, CHEK1, AP2S1, EXO1, MCM5, OIP5, CDC25B, POLA2, DONSON, CHAF1A, MCM6, RANBP1, NUP155, SLC5A6, NDUFS6, SPHK1, COX8, UFD1L |
| 1.15e-02 | TF:M00931_4 | tf | Factor: Sp1; motif: GGGGCGGGGC; match class: 4 | EIF4EBP1, TRIP13, TPI1, MTCH2, CDC20, COX5A, CCNA2, PDXK, E2F1, ZNF313, PPM1G, MCM7, SNN, BYSL, PFKL, PSMD2, GMPS, CHEK1, ASNS, SNRPA, IRAK1, WDR5, CIAO1, SRM, IDH2, GAPD, EIF2C2, MAD2L2, EXO1, APRT, MYBL2, CFL1, PTDSS1, DNMT3A, EIF4EBP2, BNIP3, WARS, LY6E, NF2, INPP4A, DHCR7, KIF4A, YARS, GARS, SLC3A2, RXRA, CYC1, CCT7, EBP, RNASEH1, STIP1, NUP62, EIF5A, AARS, SOX11, ARIH1, SLC19A1, RAD54L, CORO1C, GPS1, TFG, TMPO, CSK, PRC1, PCTK1, LRP8, GALK1, MCM6, ELOVL1, ADAMTS7, GPI, SLC5A6, DRAP1, MLF2, RFC4, CCNB1, NDUFS6, POLD2, P5, TEAD4, ENO1, S100A11, DPP3, P4HB, PKM2, CSTB, SLC7A5, SNRPC, LOC57228, GRB2, PSMD11, DRG1, RALY, UBQLN1, KARS, KIF3C, ALAS1, ANLN, PSMB10, PFN1, SLC16A3, AP1B1, UBE2I, EZH2, MAP4K2, TCP1, KPNA4, SPHK1, TMSB10, POLD1, HGS, PPAN, PRPS1, TAGLN2, POLR2H, UQCRH, MYO10, MVD, BLM, SOD2, EIF2B3, TACC3, PFKP, SLC25A5, ATP5C1, KDELR2, GALNS |
| 9.65e-03 | TF:M00920_1 | tf | Factor: E2F; motif: NKCGCGCSAAAN; match class: 1 | E2F1, EXO1, MCM5, MCM6, NUP155, SLC5A6, SPHK1, COX8 |

**Table C. Genes inversely correlated to 4EBP1.**

| Gene | t-statistic | p-value |
| --- | --- | --- |
| NM_001380__DOCK1 | -8.86561 | 2.34E-15 |
| AL049423 | -8.83092 | 2.86E-15 |
| AL133047 | -8.70465 | 5.98E-15 |
| Contig44690_RC | -8.55264 | 1.44E-14 |
| AL049265 | -8.50974 | 1.85E-14 |
| Contig52305_RC | -8.39811 | 3.52E-14 |
| Contig34129_RC | -8.37088 | 4.12E-14 |
| NM_005824__P37NB | -8.26492 | 7.56E-14 |
| NM_015484__P29 | -8.2306 | 9.20E-14 |
| AF167706__CRIM1 | -8.16655 | 1.33E-13 |
| AF070617 | -8.15707 | 1.40E-13 |
| Contig50855_RC | -8.12036 | 1.72E-13 |
| AL050148 | -8.02692 | 2.93E-13 |
| NM_004684__SPARCL1 | -7.98482 | 3.72E-13 |
| Contig63683_RC__KIAA1548 | -7.96402 | 4.18E-13 |
| NM_016441__CRIM1 | -7.92728 | 5.15E-13 |
| Contig55883_RC | -7.9105 | 5.66E-13 |
| Contig38170_RC | -7.88538 | 6.52E-13 |
| Contig58301_RC | -7.86085 | 7.48E-13 |
| NM_004791__ITGBL1 | -7.83606 | 8.60E-13 |
| AL117502 | -7.826 | 9.10E-13 |
| NM_014880__KIAA0022 | -7.78255 | 1.16E-12 |
| Contig58129_RC | -7.70475 | 1.79E-12 |
| Contig56276_RC | -7.70453 | 1.79E-12 |
| Contig38398_RC | -7.67811 | 2.08E-12 |
| Contig44064_RC | -7.67541 | 2.11E-12 |
| NM_016603__LOC51306 | -7.61663 | 2.93E-12 |
| NM_006460__HIS1 | -7.61077 | 3.02E-12 |
| NM_001552__IGFBP4 | -7.60269 | 3.16E-12 |
| Contig2141_RC | -7.53926 | 4.49E-12 |
| AL117666__DKFZP586O1624 | -7.47729 | 6.33E-12 |
| AB020681__KIAA0874 | -7.4247 | 8.45E-12 |
| NM_017680__FLJ20129 | -7.42052 | 8.64E-12 |
| Contig54847_RC | -7.39565 | 9.91E-12 |
| AL137540__NTN4 | -7.39258 | 1.01E-11 |
| NM_020974__CEGP1 | -7.39173 | 1.01E-11 |
| Contig56689_RC | -7.38399 | 1.06E-11 |
| Contig760_RC | -7.38265 | 1.06E-11 |
| Contig46598_RC | -7.38179 | 1.07E-11 |
| AL161983 | -7.36417 | 1.18E-11 |
| AL049949 | -7.34533 | 1.31E-11 |
| NM_005935__MLLT2 | -7.32929 | 1.42E-11 |
| Contig36714_RC | -7.32794 | 1.44E-11 |
| AL049470__HYPB | -7.29787 | 1.69E-11 |
| Contig53023_RC | -7.28934 | 1.77E-11 |
| Contig56678_RC | -7.24731 | 2.23E-11 |
| NM_006197__PCM1 | -7.2446 | 2.26E-11 |
| Contig37141_RC | -7.24226 | 2.29E-11 |
| Contig50670 | -7.22515 | 2.51E-11 |
| Contig31646_RC | -7.19599 | 2.94E-11 |
| NM_016337__RNB6 | -7.14857 | 3.80E-11 |
| Contig66219_RC | -7.14425 | 3.89E-11 |
| NM_000332__SCA1 | -7.13253 | 4.15E-11 |
| Contig58512_RC | -7.11547 | 4.55E-11 |
| NM_001280__CIRBP | -7.10753 | 4.75E-11 |
| NM_019597__HNRPH2 | -7.09758 | 5.01E-11 |
| Contig40017_RC | -7.09259 | 5.14E-11 |
| NM_005778__RBM5 | -7.08923 | 5.24E-11 |
| AL117645 | -7.07062 | 5.79E-11 |
| Contig56390_RC | -7.0566 | 6.24E-11 |
| Contig3607_RC | -7.05173 | 6.41E-11 |
| Contig55114_RC | -7.03047 | 7.18E-11 |
| Contig53962_RC | -7.02466 | 7.41E-11 |
| Contig14284_RC | -7.01206 | 7.93E-11 |
| NM_014773__KIAA0141 | -7.00898 | 8.06E-11 |
| NM_020347__LZTFL1 | -6.99987 | 8.46E-11 |
| NM_018446__LOC55830 | -6.99655 | 8.61E-11 |
| Contig65663 | -6.98856 | 8.99E-11 |
| Contig55991_RC | -6.96902 | 9.98E-11 |
| Contig10150_RC | -6.92695 | 1.25E-10 |
| NM_003199__TCF4 | -6.91898 | 1.30E-10 |
| AL049309 | -6.90594 | 1.40E-10 |
| NM_014965__KIAA1042 | -6.90259 | 1.42E-10 |
| NM_018695__LOC55914 | -6.89834 | 1.45E-10 |
| NM_007195__POLI | -6.87326 | 1.66E-10 |
| Contig35629_RC | -6.86042 | 1.78E-10 |
| Contig49197_RC | -6.84966 | 1.88E-10 |
| Contig31449_RC | -6.84651 | 1.91E-10 |
| Contig29369_RC | -6.84644 | 1.92E-10 |
| Contig45455_RC | -6.84308 | 1.95E-10 |
| NM_012096__APPL | -6.79284 | 2.54E-10 |
| AL137566 | -6.78746 | 2.62E-10 |
| Contig57494_RC | -6.78691 | 2.62E-10 |
| Contig46044_RC | -6.78346 | 2.67E-10 |
| Contig34350_RC | -6.78114 | 2.70E-10 |
| AF131817 | -6.77369 | 2.81E-10 |
| NM_000125__ESR1 | -6.77066 | 2.86E-10 |
| NM_015032__KIAA0979 | -6.76785 | 2.90E-10 |
| AF052101 | -6.76006 | 3.02E-10 |
| Contig50360_RC | -6.75812 | 3.05E-10 |
| Contig39090_RC | -6.75133 | 3.16E-10 |
| Contig53112_RC | -6.7429 | 3.31E-10 |
| Contig57091_RC__ITM2B | -6.74125 | 3.34E-10 |
| Contig52684__LEPR | -6.73561 | 3.44E-10 |
| U79271__SDCCAG8 | -6.72679 | 3.60E-10 |
| NM_001920__DCN | -6.7203 | 3.73E-10 |
| Contig3659_RC | -6.71262 | 3.88E-10 |
| AB037749__KIAA1328 | -6.71082 | 3.92E-10 |
| NM_018167__FLJ10648 | -6.70987 | 3.93E-10 |
| Contig1040_RC | -6.7047 | 4.04E-10 |
| NM_005780__LHFP | -6.70006 | 4.14E-10 |
| NM_014635__KIAA0336 | -6.68682 | 4.44E-10 |
| AL359052__ITGBL1 | -6.67949 | 4.61E-10 |
| AB002448 | -6.67915 | 4.62E-10 |
| Contig53881_RC | -6.67634 | 4.69E-10 |
| NM_003239__TGFB3 | -6.67224 | 4.79E-10 |
| Contig26077_RC | -6.67109 | 4.82E-10 |
| NM_001393__ECM2 | -6.66723 | 4.92E-10 |
| AB007969__KIAA0500 | -6.66528 | 4.97E-10 |
| NM_003760__EIF4G3 | -6.65371 | 5.28E-10 |
| AB029032__KIAA1109 | -6.64942 | 5.40E-10 |
| Contig50367__FLJ21935 | -6.64486 | 5.53E-10 |
| Contig52430_RC | -6.63645 | 5.78E-10 |
| AB040971__KIAA1538 | -6.63276 | 5.89E-10 |
| Contig41828_RC | -6.62186 | 6.23E-10 |
| Contig52425_RC | -6.61884 | 6.33E-10 |
| NM_020353__LOC57088 | -6.60364 | 6.85E-10 |
| Contig61227_RC | -6.51503 | 1.09E-09 |
| NM_005899__M17S2 | -6.49529 | 1.20E-09 |
| U79293 | -6.49252 | 1.22E-09 |
| Contig21406_RC | -6.48507 | 1.27E-09 |
| Contig44503_RC | -6.46546 | 1.40E-09 |
| NM_007035__KERA | -6.45431 | 1.48E-09 |
| NM_014057__OGN | -6.45139 | 1.51E-09 |
| Contig51220_RC | -6.42938 | 1.69E-09 |
| NM_004787__SLIT2 | -6.41533 | 1.81E-09 |
| NM_016444__ZNF226 | -6.40967 | 1.87E-09 |
| Contig31398_RC | -6.40944 | 1.87E-09 |
| Contig53364_RC | -6.40783 | 1.88E-09 |
| Contig51117_RC | -6.38676 | 2.10E-09 |
| AL137698 | -6.38366 | 2.13E-09 |
| AB028998__KIAA1075 | -6.37694 | 2.21E-09 |
| NM_013231__FLRT2 | -6.37403 | 2.24E-09 |
| NM_003022__SH3BGRL | -6.37242 | 2.26E-09 |
| Contig48249_RC | -6.37168 | 2.27E-09 |
| AL117441__DKFZP434N126 | -6.3689 | 2.30E-09 |
| AL137707__LOC55901 | -6.36818 | 2.31E-09 |
| Contig53953_RC | -6.35891 | 2.42E-09 |
| NM_006475__OSF-2 | -6.35378 | 2.48E-09 |
| NM_006531__TG737 | -6.35239 | 2.50E-09 |
| AF007153 | -6.34977 | 2.54E-09 |
| Contig37873 | -6.3351 | 2.73E-09 |
| U56725__HSPA2 | -6.33038 | 2.80E-09 |
| Contig47308_RC | -6.32386 | 2.89E-09 |
| AJ224741__MATN3 | -6.30713 | 3.15E-09 |
| NM_002293__LAMC1 | -6.30554 | 3.18E-09 |
| NM_018194__FLJ10724 | -6.29265 | 3.39E-09 |
| Contig78_RC__SLAP | -6.29207 | 3.40E-09 |
| Contig3057_RC | -6.28865 | 3.46E-09 |
| AK001020 | -6.27661 | 3.68E-09 |
| Contig41751_RC | -6.26223 | 3.96E-09 |
| Contig778_RC | -6.24487 | 4.32E-09 |
| NM_020163__LOC56920 | -6.24274 | 4.36E-09 |
| Contig42686 | -6.24175 | 4.39E-09 |
| Contig8888_RC | -6.23858 | 4.46E-09 |
| NM_003862__FGF18 | -6.23389 | 4.56E-09 |
| NM_014827__KIAA0663 | -6.23303 | 4.58E-09 |
| Contig23404_RC | -6.23011 | 4.65E-09 |
| Contig39226_RC | -6.22233 | 4.84E-09 |
| Contig43613_RC | -6.22113 | 4.87E-09 |
| Contig54295_RC | -6.20204 | 5.36E-09 |
| NM_001656__ARFD1 | -6.19151 | 5.65E-09 |
| Contig37758_RC | -6.18766 | 5.76E-09 |
| Contig33477_RC | -6.17645 | 6.10E-09 |
| NM_014483__RBMS3 | -6.17272 | 6.21E-09 |
| AB007883__KIAA0423 | -6.17007 | 6.29E-09 |
| U50534__13CDNA73 | -6.16436 | 6.48E-09 |
| Contig46597_RC | -6.16266 | 6.53E-09 |
| NM_016348__C5ORF4 | -6.16076 | 6.60E-09 |
| Contig36810 | -6.15428 | 6.81E-09 |
| Contig42919_RC | -6.13516 | 7.50E-09 |
| AB037791__FLJ10980 | -6.13173 | 7.63E-09 |
| Contig31596_RC | -6.1263 | 7.84E-09 |
| Contig41086_RC | -6.12558 | 7.87E-09 |
| AF279865__KIF13B | -6.12305 | 7.97E-09 |
| AL157488 | -6.11976 | 8.10E-09 |
| NM_007373__SHOC2 | -6.11956 | 8.11E-09 |
| Contig30047_RC | -6.11442 | 8.32E-09 |
| Contig1239_RC | -6.10865 | 8.56E-09 |
| NM_018171__FLJ10659 | -6.10506 | 8.72E-09 |
| Contig56307 | -6.10065 | 8.91E-09 |
| Contig53909_RC__LOC56849 | -6.09761 | 9.05E-09 |
| Contig34872_RC | -6.09288 | 9.26E-09 |
| Contig42174 | -6.08731 | 9.52E-09 |
| M73547__D5S346 | -6.08436 | 9.67E-09 |
| NM_001202__BMP4 | -6.07887 | 9.93E-09 |
| Contig37198_RC | -6.07538 | 1.01E-08 |
| AB023163__KIAA0946 | -6.07369 | 1.02E-08 |
| NM_002222__ITPR1 | -6.07129 | 1.03E-08 |
| Contig27464_RC | -6.06765 | 1.05E-08 |
| AK000915__FLJ21940 | -6.06433 | 1.07E-08 |
| Contig8156_RC | -6.0576 | 1.10E-08 |
| NM_018676__LOC55901 | -6.04562 | 1.17E-08 |
| Contig51369_RC | -6.04529 | 1.17E-08 |
| NM_000633__BCL2 | -6.04236 | 1.19E-08 |
| Contig42854 | -6.03665 | 1.23E-08 |
| Contig46937_RC | -6.0343 | 1.24E-08 |
| Contig55834_RC | -6.02583 | 1.29E-08 |
| AL050227 | -6.01754 | 1.35E-08 |
| Contig57822_RC | -6.01728 | 1.35E-08 |
| NM_017810__FLJ20417 | -6.01407 | 1.37E-08 |
| NM_004487__GOLGB1 | -6.01118 | 1.39E-08 |
| AB011118__KIAA0546 | -6.00827 | 1.41E-08 |
| NM_006763__BTG2 | -6.00786 | 1.41E-08 |
| Contig43822_RC | -6.00568 | 1.43E-08 |
| Contig37878_RC | -6.00391 | 1.44E-08 |
| Contig49388_RC | -5.99842 | 1.48E-08 |
| NM_000662__NAT1 | -5.99426 | 1.51E-08 |
| Y07512__PRKG1 | -5.98574 | 1.58E-08 |
| NM_016831__PER3 | -5.98446 | 1.59E-08 |
| Contig47456_RC | -5.98374 | 1.59E-08 |
| NM_000313__PROS1 | -5.98045 | 1.62E-08 |
| Contig46934_RC | -5.97829 | 1.64E-08 |
| AL133574 | -5.97201 | 1.69E-08 |
| Contig51749_RC__RAI2 | -5.96009 | 1.79E-08 |
| AL137332 | -5.95859 | 1.80E-08 |
| NM_002023__FMOD | -5.9582 | 1.81E-08 |
| Contig65507_RC | -5.95273 | 1.86E-08 |
| Contig37874_RC | -5.94563 | 1.92E-08 |
| Contig31055_RC | -5.94334 | 1.94E-08 |
| NM_003014__SFRP4 | -5.94097 | 1.97E-08 |
| AL133617 | -5.94013 | 1.97E-08 |
| NM_014912__KIAA0940 | -5.93452 | 2.03E-08 |
| Contig21679_RC | -5.92424 | 2.13E-08 |
| AK000004 | -5.92285 | 2.15E-08 |
| Contig53307_RC | -5.92059 | 2.17E-08 |
| NM_017684__FLJ20136 | -5.91561 | 2.23E-08 |
| NM_003225__TFF1 | -5.90852 | 2.31E-08 |
| Contig40222_RC | -5.90802 | 2.31E-08 |
| NM_015417__DKFZP434I114 | -5.90141 | 2.39E-08 |
| NM_004460__FAP | -5.88259 | 2.62E-08 |
| NM_004126__GNG11 | -5.88183 | 2.63E-08 |
| AB011182__KIAA0610 | -5.87923 | 2.66E-08 |
| AB014558__CRY2 | -5.87891 | 2.66E-08 |
| Contig55040_RC | -5.87501 | 2.72E-08 |
| AB011115__KIAA0543 | -5.87313 | 2.74E-08 |
| NM_004349__CBFA2T1 | -5.86781 | 2.81E-08 |
| Contig37571_RC | -5.86562 | 2.84E-08 |
| Contig57644_RC | -5.86331 | 2.88E-08 |
| AL122049 | -5.85992 | 2.92E-08 |
| Contig1238_RC | -5.85606 | 2.98E-08 |
| NM_018474__HT013 | -5.8527 | 3.03E-08 |
| NM_006264__PTPN13 | -5.84279 | 3.18E-08 |
| AB011100__KIAA0528 | -5.84027 | 3.22E-08 |
| Contig55268_RC | -5.83699 | 3.27E-08 |
| AL049932__UBE2N | -5.83417 | 3.31E-08 |
| NM_016121__LOC51133 | -5.83345 | 3.33E-08 |
| Contig39795_RC | -5.83222 | 3.35E-08 |
| Contig48144_RC | -5.83168 | 3.36E-08 |
| AB011121__KIAA0549 | -5.83007 | 3.38E-08 |
| Contig55049_RC | -5.82637 | 3.44E-08 |
| NM_014904__KIAA0941 | -5.82412 | 3.48E-08 |
| Contig64502 | -5.79373 | 4.03E-08 |
| Contig6568_RC | -5.7932 | 4.05E-08 |
| Contig52743 | -5.78215 | 4.27E-08 |
| Contig13300_RC | -5.78185 | 4.27E-08 |
| AL080095 | -5.78056 | 4.30E-08 |
| Contig6164_RC | -5.76984 | 4.53E-08 |
| Contig42402_RC | -5.76517 | 4.63E-08 |
| Contig56007_RC__DDXBP1 | -5.76496 | 4.64E-08 |
| Contig53646_RC | -5.75977 | 4.76E-08 |
| NM_000426__LAMA2 | -5.75673 | 4.83E-08 |
| NM_018439__IMPACT | -5.75633 | 4.84E-08 |
| NM_003479__PTP4A2 | -5.75272 | 4.92E-08 |
| Contig49520_RC | -5.75224 | 4.93E-08 |
| Contig53870_RC | -5.74839 | 5.02E-08 |
| D87076__KIAA0239 | -5.74835 | 5.03E-08 |
| AF070582 | -5.74818 | 5.03E-08 |
| Contig53357_RC | -5.74416 | 5.13E-08 |
| Contig21421_RC | -5.74386 | 5.14E-08 |
| NM_005802__TP53BPL | -5.73905 | 5.26E-08 |
| Contig37826 | -5.73833 | 5.27E-08 |
| Contig54656_RC | -5.73311 | 5.41E-08 |
| Contig749_RC | -5.72854 | 5.53E-08 |
| Contig36836_RC | -5.72263 | 5.69E-08 |
| Contig29380_RC | -5.71798 | 5.82E-08 |
| AL080186__DKFZP564B0769 | -5.71458 | 5.91E-08 |
| Contig56944_RC | -5.71397 | 5.93E-08 |
| Contig2237_RC__SMOC2 | -5.7139 | 5.93E-08 |
| Contig50118_RC | -5.69922 | 6.37E-08 |
| NM_018443__LOC55828 | -5.69901 | 6.37E-08 |
| NM_016210__LOC51161 | -5.69831 | 6.39E-08 |
| Contig17074_RC | -5.69751 | 6.42E-08 |
| AL117418__DKFZp564G2263 | -5.69702 | 6.43E-08 |
| NM_005777__RBM6 | -5.69241 | 6.58E-08 |
| Contig53305_RC | -5.68338 | 6.87E-08 |
| NM_014675__KIAA0445 | -5.68132 | 6.94E-08 |
| AB020689__KIAA0882 | -5.67393 | 7.19E-08 |
| Contig30437_RC | -5.67121 | 7.28E-08 |
| AF073770__COT | -5.67119 | 7.28E-08 |
| NM_014454__PA26 | -5.66353 | 7.55E-08 |
| AL157464__FLJ12085 | -5.66177 | 7.62E-08 |
| U68494 | -5.65338 | 7.93E-08 |
| NM_016248__LOC51707 | -5.64963 | 8.07E-08 |
| Contig54915_RC | -5.64908 | 8.09E-08 |
| R41639_RC | -5.6465 | 8.19E-08 |
| AB018260__KIAA0717 | -5.64336 | 8.32E-08 |
| NM_000254__MTR | -5.64325 | 8.32E-08 |
| NM_018422__DKFZp761K1423 | -5.63705 | 8.57E-08 |
| NM_007168__ABCA8 | -5.63491 | 8.66E-08 |
| Contig40434_RC | -5.63426 | 8.69E-08 |
| AB037748__KIAA1327 | -5.63028 | 8.85E-08 |
| Contig35814_RC | -5.62 | 9.30E-08 |
| Contig48790_RC | -5.61923 | 9.33E-08 |
| Contig10429_RC | -5.61564 | 9.49E-08 |
| Contig50939_RC__MAP4K5 | -5.61342 | 9.59E-08 |
| Contig39556_RC | -5.61131 | 9.69E-08 |
| Contig51105_RC | -5.6035 | 1.01E-07 |
| Contig57825_RC | -5.59698 | 1.04E-07 |
| NM_014639__KIAA0372 | -5.58726 | 1.09E-07 |
| NM_007184__I-1 | -5.57669 | 1.14E-07 |
| D50406__RECK | -5.57472 | 1.15E-07 |
| AL137438__SEC15L | -5.57371 | 1.16E-07 |
| AB023210__KIAA0993 | -5.56824 | 1.19E-07 |
| AL157502 | -5.56644 | 1.20E-07 |
| Contig2226_RC__UTRN | -5.56594 | 1.20E-07 |
| Contig54511_RC | -5.56504 | 1.21E-07 |
| NM_002332__LRP1 | -5.56485 | 1.21E-07 |
| Contig50297_RC | -5.55255 | 1.28E-07 |
| Contig46777_RC | -5.55012 | 1.30E-07 |
| Contig58107_RC | -5.54967 | 1.30E-07 |
| Contig56026_RC | -5.54958 | 1.30E-07 |
| NM_002001__FCER1A | -5.5439 | 1.33E-07 |
| AF052100 | -5.53107 | 1.42E-07 |
| Contig51553_RC | -5.531 | 1.42E-07 |
| Contig37399_RC | -5.53015 | 1.42E-07 |
| NM_004538__NAP1L3 | -5.52997 | 1.42E-07 |
| AF007155__FLJ10257 | -5.52958 | 1.43E-07 |
| AB011136__KIAA0564 | -5.52933 | 1.43E-07 |
| AF180425__KIAA1105 | -5.52373 | 1.47E-07 |
| Contig6118_RC | -5.51744 | 1.51E-07 |
| Contig20697_RC | -5.5137 | 1.54E-07 |
| NM_001609__ACADSB | -5.51009 | 1.56E-07 |
| NM_016002__LOC51097 | -5.50972 | 1.57E-07 |
| Contig40500_RC | -5.50851 | 1.58E-07 |
| Contig52147_RC | -5.50695 | 1.59E-07 |
| Contig3228_RC | -5.50545 | 1.60E-07 |
| AL080114 | -5.50327 | 1.62E-07 |
| NM_014870__KIAA0478 | -5.50306 | 1.62E-07 |
| Contig62901_RC | -5.50111 | 1.63E-07 |
| NM_017661__FLJ20086 | -5.49656 | 1.67E-07 |
| Contig33062_RC | -5.49625 | 1.67E-07 |
| Contig45367_RC | -5.4909 | 1.71E-07 |
| NM_004594__SLC9A5 | -5.49001 | 1.72E-07 |
| AB037721__KIAA1300 | -5.48999 | 1.72E-07 |
| NM_016477__LOC51245 | -5.48959 | 1.72E-07 |
| D86985__KIAA0232 | -5.48771 | 1.74E-07 |
| NM_004354__CCNG2 | -5.48728 | 1.74E-07 |
| Contig32970_RC | -5.48637 | 1.75E-07 |
| D26070__ITPR1 | -5.48513 | 1.76E-07 |
| NM_005808__HYA22 | -5.48426 | 1.77E-07 |
| AL050228 | -5.48325 | 1.78E-07 |
| Contig52639_RC__RAB5C | -5.47914 | 1.81E-07 |
| NM_012429__SEC14L2 | -5.47164 | 1.87E-07 |
| Contig13550_RC | -5.47137 | 1.88E-07 |
| Contig10575_RC | -5.46449 | 1.94E-07 |
| AL049378 | -5.46244 | 1.96E-07 |
| S80864__CYCL | -5.46209 | 1.96E-07 |
| NM_018836__MOT8 | -5.46022 | 1.98E-07 |
| Contig35896_RC | -5.45662 | 2.01E-07 |
| Contig39935_RC | -5.45567 | 2.02E-07 |
| Contig55048_RC | -5.45409 | 2.03E-07 |
| AK000060__AK000060 | -5.45221 | 2.05E-07 |
| NM_015642__ZNF288 | -5.45178 | 2.06E-07 |
| Contig17490_RC | -5.44973 | 2.08E-07 |
| NM_016056__LOC51643 | -5.44797 | 2.09E-07 |
| Contig32236_RC | -5.44653 | 2.11E-07 |
| Contig40365_RC | -5.43797 | 2.19E-07 |
| NM_017844__FLJ20499 | -5.4372 | 2.20E-07 |
| NM_000060__BTD | -5.43652 | 2.21E-07 |
| Contig56152_RC | -5.43604 | 2.21E-07 |
| Contig57173_RC | -5.43256 | 2.25E-07 |
| Contig40727_RC | -5.42823 | 2.30E-07 |
| Contig57062_RC | -5.42812 | 2.30E-07 |
| AF227899__KIAA0117 | -5.42624 | 2.32E-07 |
| NM_016576__LOC51292 | -5.42322 | 2.35E-07 |
| AB040969__KIAA1536 | -5.4227 | 2.36E-07 |
| NM_014710__KIAA0443 | -5.4199 | 2.39E-07 |
| NM_004902__CC1.3 | -5.41953 | 2.39E-07 |
| Contig37140_RC | -5.41674 | 2.42E-07 |
| Contig178_RC | -5.41344 | 2.46E-07 |
| Contig784_RC | -5.41156 | 2.48E-07 |
| NM_003248__THBS4 | -5.41042 | 2.49E-07 |
| Contig54913_RC__SLC1A1 | -5.40646 | 2.54E-07 |
| Contig40105 | -5.4052 | 2.56E-07 |
| NM_002345__LUM | -5.40274 | 2.59E-07 |
| Contig31913_RC | -5.40007 | 2.62E-07 |
| Contig65785_RC | -5.39859 | 2.64E-07 |
| Contig24609_RC | -5.37186 | 2.98E-07 |
| X57025__IGF1 | -5.36967 | 3.01E-07 |
| NM_004385__CSPG2 | -5.3687 | 3.03E-07 |
| Contig50588_RC | -5.36848 | 3.03E-07 |
| Contig52205_RC__LOC51244 | -5.36626 | 3.06E-07 |
| AL049337 | -5.3662 | 3.06E-07 |
| Contig47539_RC | -5.36277 | 3.11E-07 |
| Contig51795_RC | -5.36142 | 3.13E-07 |
| Contig25290_RC | -5.35966 | 3.16E-07 |
| NM_000495__COL4A5 | -5.35776 | 3.19E-07 |
| NM_018036__FLJ10242 | -5.35224 | 3.27E-07 |
| Contig41448_RC | -5.35075 | 3.29E-07 |
| Contig57896_RC | -5.34769 | 3.34E-07 |
| NM_004866__SCAMP1 | -5.34532 | 3.37E-07 |
| X59405__MCP | -5.34387 | 3.40E-07 |
| Contig3359_RC | -5.3427 | 3.42E-07 |
| AK000884__LRRFIP1 | -5.34165 | 3.43E-07 |
| NM_005767__P2Y5 | -5.34128 | 3.44E-07 |
| NM_006561__CUGBP2 | -5.34034 | 3.45E-07 |
| Contig55327_RC | -5.33946 | 3.47E-07 |
| Contig263_RC | -5.33924 | 3.47E-07 |
| Contig3820_RC__LOC56898 | -5.33908 | 3.47E-07 |
| Contig23855_RC | -5.33435 | 3.55E-07 |
| NM_016089__SZF1 | -5.33042 | 3.61E-07 |
| Contig16740_RC | -5.32875 | 3.64E-07 |
| Contig42008_RC | -5.32799 | 3.66E-07 |
| NM_005544__IRS1 | -5.32591 | 3.69E-07 |
| Contig53585_RC__CG005 | -5.32583 | 3.69E-07 |
| NM_000165__GJA1 | -5.32432 | 3.72E-07 |
| NM_000414__HSD17B4 | -5.31905 | 3.81E-07 |
| AL049335 | -5.31889 | 3.81E-07 |
| NM_001286__CLCN6 | -5.31852 | 3.82E-07 |
| Contig44186_RC | -5.3182 | 3.82E-07 |
| Contig26641 | -5.31734 | 3.84E-07 |
| NM_000153__GALC | -5.31563 | 3.87E-07 |
| Contig54751_RC | -5.31291 | 3.92E-07 |
| Contig1034_RC | -5.30986 | 3.97E-07 |
| Contig36533_RC | -5.30818 | 4.00E-07 |

**Table D. Pathways inversely correlated to 4EBP1.**

| p-value | Term | Term ID | Term description | Genes |
| --- | --- | --- | --- | --- |
| 6.30e-03 | GO:0022612 | BP | gland morphogenesis | CAV1, IGF1, SFRP4, SLIT2 |
| 1.06e-02 | GO:0048584 | BP | positive regulation of response to stimulus | AKAP12, CAV1, IGF1, JAG1, PELI2, PROS1, SFRP4, SLIT2 |
| 7.59e-03 | GO:0001944 | BP | vasculature development | CAV1, IGF1, JAG1, RECK, SFRP4, SLIT2 |
| 7.68e-03 | GO:0044421 | CC | extracellular region part | ADAMTS5, DCN, ECM2, FLRT2, IGF1, SFRP4, SLIT2, SPARCL1 |
| 4.97e-02 | CORUM:2462 | co | caveolin-1 homodimer complex | CAV1 |
