# Supplementary material for: Revealing Different Roles of the mTOR-Targets S6K1 and S6K2 in Breast Cancer by Expression Profiling and Structural Analysis
Source: PLoS One. 2015 Dec 23;10(12):e0145013. doi: 10.1371/journal.pone.0145013 (PMC4689523; doi:10.1371/journal.pone.0145013)
Supplement: S5 Table — (DOCX) [file pone.0145013.s009.docx]

**S5 Table. Genes correlated to both S6K1 and 4EBP1 in the van de Vijver cohort.**

| Genes correlated to | S6K1  t-statistic | S6K1  p-value | S6K2  t-statistic | S6K2  p-value | 4EBP1  t-statistic | 4EBP1  p-value |
| --- | --- | --- | --- | --- | --- | --- |
| *S6K1 and 4EBP1* |  |  |  |  |  |  |
| NM_002106__H2AFZ | 5,31 | 3,85E-07 | 5,02 | 1,49E-06 | 6,01 | 1,42E-08 |
| NM_002358__MAD2L1 | 5,46 | 1,97E-07 | 4,60 | 9,11E-06 | 6,43 | 1,70E-09 |
|  |  |  |  |  |  |  |
| *4EBP1 positive, S6K1 inverse* |  |  |  |  |  |  |
| NM_014390__p100 | -6,45 | 1,45E-09 | 1,68 | 0,09424 | 5,65 | 8,07E-08 |
| NM_001084__PLOD3 | -5,27 | 4,72E-07 | 1,29 | 0,199419 | 5,65 | 7,91E-08 |
|  |  |  |  |  |  |  |
| *S6K1 positive, 4EBP1 inverse* |  |  |  |  |  |  |
| AB020681__KIAA0874 | 5,86 | 2,91E-08 | -3,11 | 0,00225799 | -7,42 | 8,45E-12 |
| Contig53962_RC | 5,68 | 6,77E-08 | -3,60 | 0,000429205 | -7,02 | 7,41E-11 |
| AL049309 | 6,64 | 5,60E-10 | -3,82 | 0,000195659 | -6,91 | 1,40E-10 |
| NM_018695__LOC55914 | 6,86 | 1,75E-10 | -3,14 | 0,00203901 | -6,90 | 1,45E-10 |
| Contig49197_RC | 6,75 | 3,10E-10 | -2,84 | 0,00518715 | -6,85 | 1,88E-10 |
| Contig57091_RC__ITM2B | 5,84 | 3,23E-08 | -4,11 | 6,51E-05 | -6,74 | 3,34E-10 |
| NM_018167__FLJ10648 | 5,75 | 4,82E-08 | -2,02 | 0,0452337 | -6,71 | 3,93E-10 |
| NM_014635__KIAA0336 | 6,00 | 1,41E-08 | -4,47 | 1,53E-05 | -6,69 | 4,44E-10 |
| AB002448 | 5,34 | 3,41E-07 | -2,18 | 0,0306919 | -6,68 | 4,62E-10 |
| Contig42686 | 5,52 | 1,46E-07 | -3,31 | 0,00115981 | -6,24 | 4,39E-09 |
| NM_014827__KIAA0663 | 6,19 | 5,45E-09 | -1,70 | 0,0919609 | -6,23 | 4,58E-09 |
| NM_001656__ARFD1 | 7,62 | 2,80E-12 | -3,92 | 0,000137865 | -6,19 | 5,65E-09 |
| NM_007373__SHOC2 | 6,41 | 1,78E-09 | -1,60 | 0,11183 | -6,12 | 8,11E-09 |
| Contig42174 | 6,19 | 5,55E-09 | -2,37 | 0,0189004 | -6,09 | 9,52E-09 |
| M73547__D5S346 | 5,46 | 1,99E-07 | -3,70 | 0,000298942 | -6,08 | 9,67E-09 |
| NM_004487__GOLGB1 | 7,32 | 1,46E-11 | -1,16 | 0,248242 | -6,01 | 1,39E-08 |
| AB011118__KIAA0546 | 6,22 | 4,70E-09 | -2,76 | 0,0064426 | -6,01 | 1,41E-08 |
| Contig37826 | 7,87 | 6,72E-13 | -2,79 | 0,0059352 | -5,74 | 5,27E-08 |
| AL117418__DKFZp564G2263 | 5,86 | 2,89E-08 | -2,82 | 0,0055269 | -5,70 | 6,43E-08 |
| Contig51105_RC | 5,59 | 1,07E-07 | -3,34 | 0,00104557 | -5,60 | 1,01E-07 |
| AF052100 | 5,70 | 6,29E-08 | -3,87 | 0,000160215 | -5,53 | 1,42E-07 |
| AF227899__KIAA0117 | 8,11 | 1,77E-13 | -2,71 | 0,00755505 | -5,43 | 2,32E-07 |
| NM_004902__CC1,3 | 6,10 | 8,94E-09 | -2,43 | 0,0161076 | -5,42 | 2,39E-07 |
