# Supplementary material for: Revealing Different Roles of the mTOR-Targets S6K1 and S6K2 in Breast Cancer by Expression Profiling and Structural Analysis
Source: PLoS One. 2015 Dec 23;10(12):e0145013. doi: 10.1371/journal.pone.0145013 (PMC4689523; doi:10.1371/journal.pone.0145013)
Supplement: S6 Table — Genes positively correlated to S6K2 only (Table A). Genes inversely correlated to S6K2 only (Table B). Pathways positively correlated to S6K2 only (Table C). Pathways inversely correlated to S6K2 only (Table D). (DOCX) [file pone.0145013.s010.docx]

**Table A. Genes positively correlated to S6K2 only.**

| Genes correlated with | S6K2  t-statistic | S6K2  p-value | S6K1  t-statistic | S6K1  p-value | 4EBP1  t-statistic | 4EBP1  p-value |
| --- | --- | --- | --- | --- | --- | --- |
| NM_003952__RPS6KB2 | 17,418 | 1,45E-37 | 0,309444 | 0,757416 | 3,02348 | 0,00294943 |
| NM_005220__DLX3 | 7,42954 | 8,23E-12 | -2,55337 | 0,0116736 | 3,78649 | 0,000222056 |
| NM_020530__OSM | 7,35156 | 1,26E-11 | -1,51358 | 0,132251 | 1,59325 | 0,113252 |
| NM_006076__RAB-R | 6,85657 | 1,82E-10 | -1,53812 | 0,12614 | 3,35568 | 0,00100762 |
| NM_013289__KIR3DL1 | 6,72358 | 3,66E-10 | -0,764702 | 0,445658 | 3,59181 | 0,000447082 |
| NM_005599__NHLH2 | 6,71864 | 3,76E-10 | -0,854867 | 0,393997 | 4,60662 | 8,80E-06 |
| NM_015923__LOC51597 | 6,56056 | 8,57E-10 | -1,84539 | 0,0669657 | 4,45352 | 1,66E-05 |
| NM_003077__SMARCD2 | 6,40278 | 1,93E-09 | 5,32865 | 3,59E-07 | 3,07549 | 0,00250653 |
| NM_003495__H4FM | 6,3043 | 3,20E-09 | 2,96184 | 0,00356018 | 5,0654 | 1,20E-06 |
| NM_002653__PITX1 | 6,27994 | 3,62E-09 | 0,402628 | 0,687799 | 4,081 | 7,33E-05 |
| NM_021052__H2AFA | 6,26689 | 3,86E-09 | 2,24543 | 0,0262123 | 2,56448 | 0,0113356 |
| NM_005155__PPT2 | 6,26605 | 3,88E-09 | -0,0469721 | 0,962598 | 2,00071 | 0,047265 |
| NM_003833__MATN4 | 6,15262 | 6,87E-09 | -1,16068 | 0,247628 | 2,0686 | 0,0403354 |
| NM_006396__SSSCA1 | 6,10314 | 8,80E-09 | -0,194427 | 0,846106 | 5,24394 | 5,38E-07 |
| L22005__CDC34 | 6,01448 | 1,37E-08 | 1,35374 | 0,177869 | 5,04608 | 1,31E-06 |
| NM_014207__CD5 | 5,76844 | 4,56E-08 | -0,624769 | 0,533078 | 1,49184 | 0,137884 |
| NM_002212__ITGB4BP | 5,76561 | 4,62E-08 | -0,215106 | 0,829979 | 3,83359 | 0,000186727 |
| Contig39603_RC__CNOT3 | 5,7092 | 6,07E-08 | 1,92884 | 0,0556518 | 2,44351 | 0,0157295 |
| NM_002799__PSMB7 | 5,69419 | 6,52E-08 | 1,621 | 0,107132 | 4,89115 | 2,60E-06 |
| NM_004320__ATP2A1 | 5,69412 | 6,52E-08 | -1,58932 | 0,114108 | 3,53833 | 0,000539314 |
| NM_014182__HSPC160 | 5,65958 | 7,70E-08 | 3,4007 | 0,000862893 | 4,35833 | 2,45E-05 |
| NM_002757__MAP2K5 | 5,6348 | 8,66E-08 | 1,37412 | 0,171468 | 4,18607 | 4,87E-05 |
| NM_002918__RFX1 | 5,62075 | 9,27E-08 | 0,980242 | 0,328556 | 2,91707 | 0,00408832 |
| NM_005082__ZNF147 | 5,61298 | 9,61E-08 | 3,93264 | 0,000128363 | 3,95848 | 0,000117073 |
| NM_016535__HSPC189 | 5,60617 | 9,93E-08 | -2,43568 | 0,0160439 | 3,13768 | 0,00205786 |
| NM_013282__ICBP90 | 5,6034 | 1,01E-07 | 3,38316 | 0,000915686 | 4,2572 | 3,67E-05 |
| NM_002883__RANGAP1 | 5,60321 | 1,01E-07 | -1,33759 | 0,183068 | 4,74604 | 4,87E-06 |
| NM_005787__NOT56L | 5,59012 | 1,07E-07 | 0,603984 | 0,546773 | 4,355 | 2,48E-05 |
| NM_003198__TCEB3 | 5,55603 | 1,26E-07 | -2,01668 | 0,0455253 | 4,76784 | 4,44E-06 |
| Contig55038_RC__MCOLN1 | 5,5164 | 1,52E-07 | 1,76422 | 0,0797442 | 1,53468 | 0,127012 |
| NM_005828__HAN11 | 5,50942 | 1,57E-07 | 5,64909 | 7,94E-08 | 3,62204 | 0,000401748 |
| NM_016209__LOC51693 | 5,47819 | 1,82E-07 | 0,855132 | 0,393851 | 3,87412 | 0,000160663 |
| NM_004704__U3-55K | 5,4778 | 1,82E-07 | -2,98398 | 0,00332602 | 2,60314 | 0,0101842 |
| Contig55031_RC__NG7 | 5,47454 | 1,85E-07 | -0,474995 | 0,635486 | 4,413 | 1,96E-05 |
| NM_014275__MGAT4B | 5,46563 | 1,93E-07 | -1,14921 | 0,252311 | 2,20529 | 0,028987 |
| NM_016326__LOC51192 | 5,45336 | 2,04E-07 | 0,330231 | 0,741689 | 2,90615 | 0,00422558 |
| NM_002602__PDE6G | 5,36757 | 3,04E-07 | 0,916883 | 0,360686 | 0,495384 | 0,621069 |
| NM_000454__SOD1 | 5,34309 | 3,41E-07 | 4,90553 | 2,41E-06 | 1,72374 | 0,0868572 |
| NM_017713__FLJ20211 | 5,32502 | 3,71E-07 | -1,6679 | 0,0974359 | 2,11978 | 0,0357044 |
| NM_000402__G6PD | 5,32482 | 3,71E-07 | -0,661323 | 0,509427 | 5,13493 | 8,81E-07 |
| NM_021257__NGB | 5,31604 | 3,86E-07 | -0,602429 | 0,547804 | 0,325638 | 0,745161 |
| NM_006222__PIN1L | 5,31222 | 3,93E-07 | -0,465716 | 0,642099 | 0,406702 | 0,684818 |

**Table B. Genes inversely correlated to S6K2 only.**

| Genes correlated to | S6K2  t-statistic | S6K2  p-value | S6K1  t-statistic | S6K1  p-value | 4EBP1  t-statistic | 4EBP1  p-value |
| --- | --- | --- | --- | --- | --- | --- |
| NM_005261__GEM | -6,03322 | 1,25E-08 | -0,44866 | 0,654329 | -2,96395 | 0,003544 |
| NM_007038__ADAMTS5 | -6,01252 | 1,38E-08 | 0,539929 | 0,590052 | -5,12901 | 9,05E-07 |
| NM_006472__VDUP1 | -5,93331 | 2,04E-08 | -1,22928 | 0,220905 | -4,57787 | 9,93E-06 |
| NM_000214__JAG1 | -5,8288 | 3,40E-08 | -1,48849 | 0,138735 | -3,39564 | 0,000881 |
| NM_005100__AKAP12 | -5,80489 | 3,82E-08 | -0,95763 | 0,3398 | -5,07448 | 1,16E-06 |
| NM_019018__FLJ11127 | -5,53039 | 1,42E-07 | -1,96892 | 0,050817 | -5,03224 | 1,40E-06 |
| NM_001753__CAV1 | -5,48333 | 1,77E-07 | -2,44105 | 0,015817 | -4,49202 | 1,42E-05 |
| AL133605__PELI2 | -5,37833 | 2,90E-07 | -1,58399 | 0,115316 | -4,94401 | 2,06E-06 |

**Table C. Pathways positively correlated to S6K2 only.**

| p-value | Term | Term ID | Term description | Genes |
| --- | --- | --- | --- | --- |
| 2.04e-02 | BIOGRID:00000 | bi | BioGRID interaction data | ATP2A1,CD5,CDC34,DLX3,MAP2K5 |
| 4.86e-02 | MI:mmu-miR-715 | mi | MI:mmu-miR-715 | ATP2A1,MAP2K5,OSM,PDE6G,RPS6KB2 |
| 1.77e-03 | MI:mmu-miR-702 | mi | MI:mmu-miR-702 | CD5,MAP2K5,MCOLN1,PDE6G,RPS6KB2,SMARCD2 |

**Table D. Pathways inversely correlated to S6K2 only.**

| p-value | Term | Term ID | Term description | Genes |
| --- | --- | --- | --- | --- |
| 5.00e-02 | CORUM:5714 | co | eNOS-CAV1 complex | CAV1 |
| 2.50e-02 | CORUM:2462 | co | caveolin-1 homodimer complex | CAV1 |
