# Supplementary material for: Revealing Different Roles of the mTOR-Targets S6K1 and S6K2 in Breast Cancer by Expression Profiling and Structural Analysis
Source: PLoS One. 2015 Dec 23;10(12):e0145013. doi: 10.1371/journal.pone.0145013 (PMC4689523; doi:10.1371/journal.pone.0145013)
Supplement: S7 Table — Genes positively correlated to 4EBP1 only (Table A). Pathways correlated positively to 4EBP1 only (Table B). Genes inversely correlated to 4EBP1 only (Table C). Pathways inversely correlated to 4EBP1 only (Table D). (DOCX) [file pone.0145013.s011.docx]

**Table A. Genes positively correlated to 4EBP1 only.**

| Gene | 4EBP1  t-statistic | 4EBP1  p-value | S6K1  t-statistic | S6K1  p-value | S6K2  t-statistic | S6K2  p-value |
| --- | --- | --- | --- | --- | --- | --- |
| NM_004095__EIF4EBP1 | 20,9479 | 5,58E-46 | -0,112358 | 0,910691 | 4,57234 | 1,02E-05 |
| NM_000365__TPI1 | 8,98993 | 1,13E-15 | -2,94284 | 0,0037731 | 5,00114 | 1,60E-06 |
| NM_006067__NOC4 | 8,55628 | 1,41E-14 | -1,16931 | 0,244146 | 4,48203 | 1,48E-05 |
| NM_014342__MTCH2 | 8,40438 | 3,40E-14 | -2,36357 | 0,0193903 | 4,44141 | 1,75E-05 |
| AF141882__APMCF1 | 8,16997 | 1,30E-13 | -1,42158 | 0,157238 | 3,32376 | 0,001121 |
| NM_004255__COX5A | 8,0837 | 2,12E-13 | -0,101081 | 0,919622 | 5,18645 | 6,98E-07 |
| NM_001237__CCNA2 | 8,05789 | 2,46E-13 | 0,719106 | 0,473202 | 4,58054 | 9,82E-06 |
| NM_004846__EIF4EL3 | 8,05361 | 2,52E-13 | 0,0831936 | 0,933809 | 4,54576 | 1,13E-05 |
| NM_021203__APMCF1 | 8,04478 | 2,65E-13 | -2,06259 | 0,0408879 | 3,03158 | 0,002876 |
| NM_003681__PDXK | 8,03165 | 2,85E-13 | -0,299572 | 0,764921 | 5,17639 | 7,31E-07 |
| NM_016491__MRPL37 | 7,96444 | 4,17E-13 | -1,73377 | 0,0850274 | 3,78396 | 0,000224 |
| D55716__MCM7 | 7,77973 | 1,18E-12 | -0,107322 | 0,914678 | 3,91093 | 0,00014 |
| NM_006114__D19S1177E | 7,76968 | 1,25E-12 | -0,513305 | 0,608498 | 4,5797 | 9,85E-06 |
| NM_006623__PHGDH | 7,76557 | 1,28E-12 | -1,30657 | 0,193372 | 2,98943 | 0,003277 |
| NM_001689__ATP5G3 | 7,74497 | 1,43E-12 | -0,431141 | 0,666989 | 4,24922 | 3,79E-05 |
| NM_003498__SNN | 7,73485 | 1,52E-12 | -0,080575 | 0,935888 | 4,41804 | 1,92E-05 |
| NM_002949__MRPL12 | 7,72685 | 1,58E-12 | 1,7592 | 0,0805961 | 5,04892 | 1,30E-06 |
| AF155652__DKFZP434L1021 | 7,72406 | 1,61E-12 | -0,60859 | 0,543723 | 3,20649 | 0,001649 |
| NM_004053__BYSL | 7,63592 | 2,63E-12 | -1,1415 | 0,255493 | 5,21316 | 6,19E-07 |
| NM_002626__PFKL | 7,61774 | 2,91E-12 | -2,68355 | 0,0081085 | 3,8176 | 0,000198 |
| NM_003683__D21S2056E | 7,59909 | 3,23E-12 | -2,06574 | 0,0405851 | 4,62135 | 8,27E-06 |
| NM_002808__PSMD2 | 7,58583 | 3,47E-12 | -1,39625 | 0,164716 | 5,09414 | 1,06E-06 |
| NM_013277__ID-GAP | 7,54783 | 4,29E-12 | 2,95469 | 0,003639 | 4,83449 | 3,33E-06 |
| AF112213__LOC55969 | 7,54091 | 4,45E-12 | 2,97836 | 0,0033841 | 4,32067 | 2,85E-05 |
| NM_002914__RFC2 | 7,51514 | 5,13E-12 | 1,31309 | 0,191171 | 4,37061 | 2,33E-05 |
| NM_003875__GMPS | 7,5147 | 5,15E-12 | 0,104059 | 0,917262 | 5,26177 | 4,96E-07 |
| NM_001274__CHEK1 | 7,47427 | 6,43E-12 | -0,403877 | 0,686882 | 3,16988 | 0,001856 |
| NM_001673__ASNS | 7,47393 | 6,44E-12 | 0,019023 | 0,984848 | 3,82523 | 0,000193 |
| NM_007019__UBCH10 | 7,42375 | 8,49E-12 | 1,77798 | 0,0774468 | 4,75639 | 4,66E-06 |
| NM_001569__IRAK1 | 7,40655 | 9,33E-12 | -4,51528 | 1,28E-05 | 2,93017 | 0,003929 |
| AF047002__ALY | 7,36907 | 1,15E-11 | 2,15622 | 0,0326712 | 5,09601 | 1,05E-06 |
| X97074__AP2S1 | 7,35812 | 1,22E-11 | -0,49585 | 0,620731 | 4,74086 | 4,98E-06 |
| NM_003720__DSCR2 | 7,35281 | 1,25E-11 | 0,0834425 | 0,933612 | 3,31844 | 0,001141 |
| NM_006009__TUBA3 | 7,34297 | 1,32E-11 | -0,806681 | 0,421136 | 4,13604 | 5,92E-05 |
| NM_004493__HADH2 | 7,31129 | 1,57E-11 | -1,55305 | 0,122533 | 4,74944 | 4,80E-06 |
| NM_014321__ORC6L | 7,31051 | 1,58E-11 | 4,20677 | 4,46E-05 | 4,91204 | 2,37E-06 |
| AK000552__WDR5 | 7,28928 | 1,77E-11 | -0,374426 | 0,70862 | 5,21733 | 6,07E-07 |
| NM_002950__RPN1 | 7,28784 | 1,79E-11 | -1,60123 | 0,111444 | 3,65462 | 0,000358 |
| NM_013237__PX19 | 7,26132 | 2,06E-11 | 2,12115 | 0,0355644 | 4,40621 | 2,02E-05 |
| NM_004804__CIAO1 | 7,24927 | 2,20E-11 | 1,25672 | 0,210822 | 4,36613 | 2,37E-05 |
| NM_006087__TUBB5 | 7,22578 | 2,50E-11 | -3,20905 | 0,0016309 | 4,57948 | 9,86E-06 |
| NM_003132__SRM | 7,19883 | 2,90E-11 | -2,31093 | 0,0222083 | 3,63682 | 0,000381 |
| NM_002168__IDH2 | 7,19598 | 2,94E-11 | -0,675375 | 0,500484 | 4,78828 | 4,06E-06 |
| NM_016405__HSU93243 | 7,18107 | 3,19E-11 | -2,87411 | 0,0046447 | 2,77109 | 0,006309 |
| AF052155__SEC13L1 | 7,17761 | 3,25E-11 | -0,598101 | 0,550681 | 4,53862 | 1,17E-05 |
| NM_002046__GAPD | 7,14052 | 3,97E-11 | -3,14314 | 0,0020173 | 5,02858 | 1,42E-06 |
| NM_012247__SPS | 7,12503 | 4,32E-11 | -3,03631 | 0,0028278 | 2,64697 | 0,009007 |
| S90469__POR | 7,12355 | 4,35E-11 | -0,583364 | 0,56053 | 1,57454 | 0,117512 |
| NM_006845__KNSL6 | 7,09101 | 5,19E-11 | 0,695245 | 0,487985 | 4,7359 | 5,09E-06 |
| NM_018518__PRO2249 | 7,06749 | 5,89E-11 | 0,81514 | 0,416293 | 4,50697 | 1,33E-05 |
| AF121255__EIF2C2 | 7,05062 | 6,45E-11 | 0,107394 | 0,914621 | 3,81854 | 0,000197 |
| NM_006341__MAD2L2 | 7,04449 | 6,66E-11 | -0,708084 | 0,479999 | 1,28483 | 0,200873 |
| NM_005837__RPP20 | 7,0266 | 7,33E-11 | 0,379561 | 0,704812 | 2,80613 | 0,005694 |
| NM_006027__EXO1 | 6,99601 | 8,64E-11 | 1,57241 | 0,117977 | 3,87263 | 0,000162 |
| NM_004336__BUB1 | 6,9714 | 9,85E-11 | 2,25499 | 0,0255912 | 4,94939 | 2,01E-06 |
| NM_001905__CTPS | 6,96046 | 1,04E-10 | -1,52661 | 0,128978 | 2,52534 | 0,012619 |
| NM_005507__CFL1 | 6,95958 | 1,05E-10 | -2,37572 | 0,0187866 | 4,38936 | 2,16E-05 |
| NM_016101__HSPC031 | 6,95814 | 1,06E-10 | 0,160694 | 0,872552 | 3,43605 | 0,000768 |
| NM_004674__ASH2L | 6,93724 | 1,18E-10 | 0,917314 | 0,360461 | 0,374586 | 0,708508 |
| NM_012474__UMPK | 6,90936 | 1,37E-10 | 0,303681 | 0,761795 | 4,23471 | 4,02E-05 |
| NM_013986__EWSR1 | 6,89559 | 1,48E-10 | 0,832074 | 0,406699 | 3,22755 | 0,00154 |
| NM_018407__LC27 | 6,88255 | 1,58E-10 | -0,104782 | 0,91669 | 1,89683 | 0,059812 |
| NM_006351__TIM44 | 6,8811 | 1,59E-10 | -1,04647 | 0,297039 | 3,83096 | 0,000189 |
| NM_014364__GAPDS | 6,84582 | 1,92E-10 | -2,33904 | 0,0206616 | 5,06458 | 1,21E-06 |
| NM_014462__LSM1 | 6,83503 | 2,03E-10 | 4,18687 | 4,82E-05 | 1,57985 | 0,116291 |
| NM_005805__POH1 | 6,82949 | 2,10E-10 | 2,91845 | 0,0040636 | 3,79142 | 0,000218 |
| NM_014754__PTDSS1 | 6,82948 | 2,10E-10 | -2,65719 | 0,0087388 | 2,07179 | 0,040032 |
| AF067972__DNMT3A | 6,82328 | 2,17E-10 | 1,55455 | 0,122175 | 3,2911 | 0,00125 |
| NM_004096__EIF4EBP2 | 6,82009 | 2,20E-10 | -4,52298 | 1,24E-05 | 0,478436 | 0,633051 |
| NM_006082__K-ALPHA-1 | 6,81059 | 2,32E-10 | -1,07994 | 0,281914 | 3,8588 | 0,00017 |
| NM_004052__BNIP3 | 6,80491 | 2,39E-10 | 1,21685 | 0,225585 | 3,74299 | 0,00026 |
| NM_012341__NGB | 6,79477 | 2,52E-10 | 2,06247 | 0,0408995 | 3,23399 | 0,001508 |
| NM_004184__WARS | 6,79441 | 2,52E-10 | -1,32059 | 0,188663 | 4,45257 | 1,67E-05 |
| NM_002346__LY6E | 6,78107 | 2,71E-10 | -1,05349 | 0,293822 | 2,7123 | 0,007479 |
| NM_006739__MCM5 | 6,77522 | 2,79E-10 | -3,65868 | 0,0003511 | 2,8297 | 0,005311 |
| NM_014064__AD-003 | 6,76567 | 2,93E-10 | -1,80405 | 0,0732434 | 5,15532 | 8,04E-07 |
| NM_000268__NF2 | 6,76027 | 3,02E-10 | -1,55906 | 0,121104 | 4,35569 | 2,47E-05 |
| NM_005002__NDUFA9 | 6,75705 | 3,07E-10 | -1,23881 | 0,217364 | 3,81042 | 0,000203 |
| NM_020150__SAR1 | 6,75358 | 3,13E-10 | 0,551579 | 0,582063 | 5,05532 | 1,26E-06 |
| NM_003686__EXO1 | 6,75114 | 3,17E-10 | 1,12455 | 0,262588 | 4,19655 | 4,67E-05 |
| NM_004504__HRB | 6,74803 | 3,22E-10 | -1,88051 | 0,0619903 | 2,28501 | 0,023743 |
| NM_004153__ORC1L | 6,74676 | 3,24E-10 | -0,946705 | 0,345322 | 4,87965 | 2,73E-06 |
| NM_004027__INPP4A | 6,73219 | 3,50E-10 | 1,44319 | 0,151066 | 2,90281 | 0,004268 |
| NM_001360__DHCR7 | 6,71644 | 3,80E-10 | -0,468922 | 0,639811 | 3,79826 | 0,000213 |
| NM_012310__KIF4A | 6,70672 | 4,00E-10 | 2,06577 | 0,0405822 | 5,18448 | 7,05E-07 |
| NM_015922__H105E3 | 6,70541 | 4,03E-10 | -1,24701 | 0,21435 | 2,15698 | 0,032633 |
| NM_003680__YARS | 6,69554 | 4,24E-10 | -1,76264 | 0,0800115 | 4,67392 | 6,62E-06 |
| NM_002047__GARS | 6,69083 | 4,35E-10 | -0,96307 | 0,337073 | 3,25814 | 0,001393 |
| NM_005721__ACTR3 | 6,68582 | 4,46E-10 | 0,915488 | 0,361415 | 3,68188 | 0,000324 |
| NM_002394__SLC3A2 | 6,67916 | 4,62E-10 | -0,088405 | 0,929674 | 4,29408 | 3,17E-05 |
| NM_002794__PSMB2 | 6,63383 | 5,86E-10 | -1,08617 | 0,279158 | 4,89961 | 2,51E-06 |
| NM_006397__RNASEHI | 6,63162 | 5,92E-10 | 0,470702 | 0,638542 | 5,30324 | 4,10E-07 |
| NM_002957__RXRA | 6,6163 | 6,42E-10 | -0,614551 | 0,539789 | 5,14945 | 8,26E-07 |
| AF025441__OIP5 | 6,59638 | 7,12E-10 | 1,60605 | 0,11038 | 3,22442 | 0,001555 |
| NM_004231__ATP6S14 | 6,56635 | 8,32E-10 | 1,75213 | 0,0818087 | 2,82277 | 0,005421 |
| NM_002266__KPNA2 | 6,56162 | 8,53E-10 | 4,96239 | 1,88E-06 | 4,77664 | 4,27E-06 |
| NM_006023__D123 | 6,55861 | 8,66E-10 | -1,05416 | 0,293516 | 3,12349 | 0,002153 |
| NM_006429__CCT7 | 6,55783 | 8,70E-10 | 1,01117 | 0,313574 | 4,58045 | 9,82E-06 |
| NM_006579__EBP | 6,55704 | 8,73E-10 | 1,35378 | 0,177856 | 4,33939 | 2,64E-05 |
| NM_004526__MCM2 | 6,55117 | 9,00E-10 | 0,0843294 | 0,932908 | 4,85955 | 2,98E-06 |
| NM_002936__RNASEH1 | 6,53706 | 9,68E-10 | -0,00889 | 0,992919 | 4,63889 | 7,68E-06 |
| NM_005918__MDH2 | 6,52382 | 1,04E-09 | -1,9677 | 0,0509592 | 3,28138 | 0,00129 |
| NM_006590__SAD1 | 6,5154 | 1,08E-09 | -0,372826 | 0,709808 | 2,87232 | 0,004678 |
| NM_012346__NUP62 | 6,50559 | 1,14E-09 | -0,172035 | 0,863643 | 5,07191 | 1,17E-06 |
| NM_002105__H2AFX | 6,49927 | 1,18E-09 | -1,52242 | 0,130023 | 4,43316 | 1,81E-05 |
| NM_004900__DJ742C19,2 | 6,49083 | 1,23E-09 | 1,15794 | 0,248741 | 2,27768 | 0,024188 |
| NM_001970__EIF5A | 6,48785 | 1,25E-09 | -2,28693 | 0,0236083 | 3,92266 | 0,000134 |
| NM_004327__BCR | 6,47329 | 1,35E-09 | -1,16323 | 0,246596 | 2,41743 | 0,016855 |
| NM_001605__AARS | 6,44205 | 1,58E-09 | -0,896779 | 0,371283 | 4,06703 | 7,74E-05 |
| NM_003108__SOX11 | 6,43528 | 1,64E-09 | -0,452827 | 0,651332 | 3,20538 | 0,001655 |
| NM_004077__CS | 6,43345 | 1,65E-09 | -0,270542 | 0,787118 | 2,71733 | 0,007372 |
| NM_002358__MAD2L1 | 6,42768 | 1,70E-09 | 5,45706 | 1,97E-07 | 4,59826 | 9,11E-06 |
| NM_001188__BAK1 | 6,41187 | 1,85E-09 | 1,61421 | 0,108598 | 5,25204 | 5,18E-07 |
| NM_004642__DOC1 | 6,41106 | 1,85E-09 | -1,4874 | 0,139022 | 2,03606 | 0,04354 |
| NM_005744__ARIH1 | 6,40616 | 1,90E-09 | 0,0835432 | 0,933532 | 1,97357 | 0,050306 |
| NM_003056__SLC19A1 | 6,39521 | 2,01E-09 | -0,814066 | 0,416906 | 3,44648 | 0,000741 |
| NM_002812__PSMD8 | 6,3936 | 2,03E-09 | 0,233332 | 0,815824 | 4,05989 | 7,95E-05 |
| NM_002689__POLA2 | 6,3841 | 2,13E-09 | -0,893669 | 0,37294 | 5,13712 | 8,73E-07 |
| NM_017613__DONSON | 6,37857 | 2,19E-09 | 1,39968 | 0,163688 | 1,62048 | 0,107272 |
| NM_003579__RAD54L | 6,36806 | 2,31E-09 | 0,774568 | 0,439822 | 4,03207 | 8,85E-05 |
| NM_014325__CORO1C | 6,35204 | 2,51E-09 | -0,348725 | 0,727788 | 3,58377 | 0,00046 |
| NM_004127__GPS1 | 6,34997 | 2,53E-09 | 0,981171 | 0,328099 | 5,25989 | 5,00E-07 |
| NM_006070__TFG | 6,34745 | 2,57E-09 | -2,74987 | 0,0067005 | 1,24252 | 0,216023 |
| NM_003276__TMPO | 6,34443 | 2,61E-09 | 2,47672 | 0,0143777 | 3,5898 | 0,00045 |
| NM_004383__CSK | 6,3433 | 2,62E-09 | -4,65307 | 7,17E-06 | 2,78563 | 0,006047 |
| NM_013421__GGT1 | 6,33383 | 2,75E-09 | -1,15186 | 0,251223 | 2,34105 | 0,020573 |
| NM_005483__CHAF1A | 6,32564 | 2,87E-09 | -0,392762 | 0,695057 | 3,41661 | 0,00082 |
| NM_003981__PRC1 | 6,32093 | 2,94E-09 | 2,97753 | 0,0033927 | 4,32384 | 2,81E-05 |
| AF114818__SIAHBP1 | 6,31145 | 3,08E-09 | -0,387931 | 0,698621 | 5,24064 | 5,46E-07 |
| NM_006201__PCTK1 | 6,30576 | 3,17E-09 | -1,43834 | 0,152435 | 4,16267 | 5,33E-05 |
| NM_017522__LRP8 | 6,30396 | 3,20E-09 | 1,38473 | 0,168205 | 4,31097 | 2,96E-05 |
| NM_004154__P2RY6 | 6,2991 | 3,28E-09 | -2,58938 | 0,0105682 | 3,68348 | 0,000323 |
| NM_000154__GALK1 | 6,29534 | 3,34E-09 | -0,710892 | 0,478262 | 4,11541 | 6,42E-05 |
| NM_012291__KIAA0165 | 6,2844 | 3,53E-09 | 2,74784 | 0,0067401 | 5,01381 | 1,52E-06 |
| NM_005915__MCM6 | 6,28224 | 3,57E-09 | 2,67663 | 0,0082698 | 3,3089 | 0,001178 |
| NM_002882__RANBP1 | 6,25766 | 4,05E-09 | -1,28276 | 0,201568 | 3,8219 | 0,000195 |
| NM_016031__ELOVL1 | 6,257 | 4,06E-09 | -4,44665 | 1,69E-05 | 1,7719 | 0,078483 |
| NM_014175__HSPC145 | 6,25112 | 4,18E-09 | 0,0113712 | 0,990943 | 2,48319 | 0,014145 |
| NM_019848__P3 | 6,23722 | 4,49E-09 | -3,38713 | 0,0009035 | 4,3966 | 2,10E-05 |
| NM_006693__CPSF4 | 6,2317 | 4,62E-09 | -0,393824 | 0,694274 | 1,45599 | 0,147528 |
| NM_003318__TTK | 6,22229 | 4,84E-09 | 1,72301 | 0,0869613 | 4,4626 | 1,60E-05 |
| NM_004298__NUP155 | 6,22206 | 4,85E-09 | -0,621087 | 0,535491 | 4,20505 | 4,52E-05 |
| NM_021039__S100A14 | 6,21887 | 4,92E-09 | -1,55303 | 0,122537 | 3,8844 | 0,000155 |
| NM_000175__GPI | 6,21493 | 5,02E-09 | -3,20794 | 0,0016368 | 3,99769 | 0,000101 |
| NM_001686__ATP5B | 6,20967 | 5,16E-09 | -1,09747 | 0,274206 | 3,8152 | 0,0002 |
| NM_021095__SLC5A6 | 6,20865 | 5,18E-09 | -1,71928 | 0,08764 | 2,42316 | 0,016602 |
| NM_001929__DGUOK | 6,19954 | 5,43E-09 | 0,748947 | 0,45507 | 2,98906 | 0,003281 |
| NM_001047__SRD5A1 | 6,19934 | 5,43E-09 | -1,89845 | 0,0595703 | 1,52822 | 0,128607 |
| NM_004068__AP2M1 | 6,19671 | 5,51E-09 | -1,15753 | 0,248908 | 3,56898 | 0,000484 |
| NM_006442__DRAP1 | 6,19162 | 5,65E-09 | -2,69212 | 0,0079126 | 5,21306 | 6,19E-07 |
| NM_005648__TCEB1 | 6,18883 | 5,73E-09 | 4,04867 | 8,25E-05 | 4,06775 | 7,71E-05 |
| NM_003662__PIR | 6,18651 | 5,80E-09 | 1,86502 | 0,0641453 | 3,67251 | 0,000336 |
| NM_001806__CEBPG | 6,16196 | 6,56E-09 | 0,964743 | 0,336237 | 3,09671 | 0,002344 |
| NM_005439__MLF2 | 6,15844 | 6,67E-09 | -2,30155 | 0,0227465 | 4,07168 | 7,60E-05 |
| NM_003821__RIPK2 | 6,15079 | 6,93E-09 | 1,23943 | 0,217135 | 2,99348 | 0,003237 |
| NM_004131__GZMB | 6,14716 | 7,06E-09 | -1,54833 | 0,123664 | 3,04678 | 0,002743 |
| NM_003035__SIL | 6,14043 | 7,30E-09 | 3,00191 | 0,0031469 | 3,44435 | 0,000746 |
| NM_005733__RAB6KIFL | 6,13577 | 7,48E-09 | 1,70883 | 0,0895646 | 4,69638 | 6,02E-06 |
| NM_005804__DDXL | 6,13397 | 7,54E-09 | 2,04112 | 0,0430037 | 4,6258 | 8,12E-06 |
| NM_004749__CPR2 | 6,11484 | 8,30E-09 | -0,423908 | 0,672244 | 5,03804 | 1,36E-06 |
| NM_002916__RFC4 | 6,10619 | 8,67E-09 | 1,49293 | 0,13757 | 3,6827 | 0,000324 |
| NM_004499__HNRPAB | 6,09772 | 9,04E-09 | 1,71757 | 0,0879526 | 4,54442 | 1,14E-05 |
| NM_012151__F8A | 6,09633 | 9,11E-09 | 0,445237 | 0,656795 | 4,66076 | 7,00E-06 |
| NM_006406__AOE372 | 6,08275 | 9,74E-09 | -0,195804 | 0,84503 | 3,45435 | 0,000721 |
| NM_006230__POLD2 | 6,05914 | 1,10E-08 | -0,47802 | 0,633337 | 4,60536 | 8,85E-06 |
| NM_001859__SLC31A1 | 6,05687 | 1,11E-08 | -0,297492 | 0,766505 | 3,21049 | 0,001628 |
| NM_005333__HCCS | 6,05095 | 1,14E-08 | 0,14383 | 0,885829 | 3,51136 | 0,000592 |
| NM_016426__GTSE1 | 6,03745 | 1,22E-08 | -0,315977 | 0,752462 | 3,18879 | 0,001746 |
| NM_004559__NSEP1 | 6,03087 | 1,26E-08 | -1,67435 | 0,0961596 | 1,65574 | 0,099908 |
| NM_005742__P5 | 6,02527 | 1,30E-08 | -1,22861 | 0,221155 | 2,21016 | 0,02864 |
| NM_005146__SART1 | 6,02166 | 1,32E-08 | -2,26566 | 0,0249131 | 2,95758 | 0,003614 |
| NM_002635__SLC25A3 | 6,01794 | 1,34E-08 | -3,86624 | 0,0001646 | 3,10561 | 0,002279 |
| NM_005563__LAP18 | 6,01542 | 1,36E-08 | 0,742709 | 0,458827 | 4,17518 | 5,08E-05 |
| NM_001211__BUB1B | 6,00814 | 1,41E-08 | 1,1544 | 0,250184 | 5,08924 | 1,08E-06 |
| NM_002106__H2AFZ | 6,00665 | 1,42E-08 | 5,31314 | 3,85E-07 | 5,01715 | 1,49E-06 |
| NM_018622__PRO2207 | 5,99884 | 1,48E-08 | -0,494739 | 0,621513 | 2,95852 | 0,003604 |
| AF155120__UBE2V1 | 5,99868 | 1,48E-08 | 0,441787 | 0,659284 | 1,74386 | 0,083274 |
| NM_003213__TEAD4 | 5,99205 | 1,53E-08 | -4,24788 | 3,79E-05 | 2,92077 | 0,004043 |
| NM_005620__S100A11 | 5,98586 | 1,58E-08 | -2,2046 | 0,0290155 | 3,53271 | 0,00055 |
| NM_017447__YG81 | 5,98016 | 1,62E-08 | -0,544648 | 0,58681 | 2,48065 | 0,014242 |
| M94362__LMNB2 | 5,97296 | 1,68E-08 | 0,73731 | 0,462093 | 3,61751 | 0,000408 |
| NM_004222__DNJ3 | 5,97293 | 1,68E-08 | 1,80331 | 0,0733601 | 3,54763 | 0,000522 |
| NM_020979__APS | 5,96579 | 1,74E-08 | -3,75735 | 0,0002459 | 1,71789 | 0,087922 |
| AJ245416__LSM2 | 5,96298 | 1,76E-08 | -2,32747 | 0,0212864 | 3,67698 | 0,00033 |
| NM_018846__SBBI26 | 5,95761 | 1,81E-08 | -1,00526 | 0,316401 | 1,16749 | 0,244903 |
| NM_004856__KNSL5 | 5,95037 | 1,88E-08 | 3,61101 | 0,0004161 | 4,34951 | 2,54E-05 |
| AJ271216__DPP3 | 5,93154 | 2,06E-08 | 1,93718 | 0,0546151 | 3,91151 | 0,00014 |
| AF113132__PSA | 5,93012 | 2,07E-08 | -3,00786 | 0,0030894 | 2,31338 | 0,022088 |
| NM_000918__P4HB | 5,92913 | 2,08E-08 | 1,02699 | 0,30609 | 4,20729 | 4,48E-05 |
| NM_002654__PKM2 | 5,92884 | 2,09E-08 | -2,76949 | 0,0063288 | 4,86511 | 2,91E-06 |
| NM_020470__54TM | 5,92828 | 2,09E-08 | -1,33752 | 0,183091 | 3,70473 | 0,000299 |
| NM_007051__FAF1 | 5,9199 | 2,18E-08 | -2,58226 | 0,010779 | 2,46355 | 0,014911 |
| NM_004049__BCL2A1 | 5,91708 | 2,21E-08 | 1,03317 | 0,303198 | 3,66149 | 0,000349 |
| NM_003481__USP5 | 5,91654 | 2,22E-08 | -2,52164 | 0,0127322 | 3,27844 | 0,001303 |
| NM_004309__ARHGDIA | 5,91364 | 2,25E-08 | 0,426722 | 0,670198 | 5,22464 | 5,87E-07 |
| Y18643__METTL1 | 5,91264 | 2,26E-08 | -0,07295 | 0,941944 | 5,15265 | 8,14E-07 |
| NM_000100__CSTB | 5,91121 | 2,28E-08 | -3,56564 | 0,0004883 | 2,93575 | 0,003863 |
| NM_003486__SLC7A5 | 5,90753 | 2,32E-08 | -2,12817 | 0,0349682 | 3,16008 | 0,001915 |
| NM_006383__KIP2 | 5,9053 | 2,34E-08 | -2,04104 | 0,0430118 | 1,19689 | 0,233276 |
| NM_001320__CSNK2B | 5,90399 | 2,36E-08 | -1,89944 | 0,0594391 | 2,84837 | 0,005025 |
| NM_003953__MPZL1 | 5,89656 | 2,44E-08 | -0,814435 | 0,416696 | 3,5724 | 0,000479 |
| NM_005005__NDUFB9 | 5,8953 | 2,46E-08 | -0,089629 | 0,928702 | 4,49456 | 1,40E-05 |
| NM_001536__HRMT1L2 | 5,87771 | 2,68E-08 | 0,453007 | 0,651203 | 4,2864 | 3,27E-05 |
| Contig55538_RC__BA395L14,2 | 5,87326 | 2,74E-08 | -6,16318 | 6,35E-09 | 0,647065 | 0,518599 |
| NM_002086__GRB2 | 5,86517 | 2,85E-08 | 3,40248 | 0,0008577 | 3,30199 | 0,001205 |
| U74612__FOXM1 | 5,85946 | 2,93E-08 | 1,21419 | 0,226596 | 4,36374 | 2,40E-05 |
| NM_007057__ZWINT | 5,84739 | 3,11E-08 | 3,3637 | 0,0009778 | 5,07918 | 1,13E-06 |
| NM_000593__ABCB2 | 5,84477 | 3,15E-08 | 0,219121 | 0,826856 | 3,55672 | 0,000506 |
| NM_002815__PSMD11 | 5,83682 | 3,27E-08 | 0,513246 | 0,608539 | 2,66826 | 0,008481 |
| NM_004147__DRG1 | 5,82643 | 3,44E-08 | -0,788307 | 0,43177 | 3,24686 | 0,001445 |
| NM_016732__RALY | 5,81434 | 3,65E-08 | 0,0819775 | 0,934775 | 3,15103 | 0,001972 |
| NM_001861__COX4 | 5,81142 | 3,70E-08 | -0,148179 | 0,882402 | 4,98203 | 1,74E-06 |
| AL162049__USP10 | 5,81016 | 3,73E-08 | -0,71655 | 0,474773 | 3,60148 | 0,000432 |
| NM_017414__USP18 | 5,80917 | 3,74E-08 | 1,54419 | 0,124664 | 1,99112 | 0,048321 |
| NM_013438__UBQLN1 | 5,80769 | 3,77E-08 | 1,06318 | 0,28942 | 3,8178 | 0,000198 |
| NM_006302__GCS1 | 5,80494 | 3,82E-08 | -2,9714 | 0,0034573 | 1,71049 | 0,089285 |
| Contig54563_RC__KNSL5 | 5,79985 | 3,92E-08 | 3,4835 | 0,00065 | 4,27485 | 3,42E-05 |
| AF129536__FBXO6 | 5,78276 | 4,25E-08 | 0,736607 | 0,46252 | 4,06014 | 7,94E-05 |
| X77588__ARD1 | 5,77475 | 4,42E-08 | -1,47325 | 0,142793 | 3,37972 | 0,000929 |
| NM_014251__SLC25A13 | 5,76979 | 4,53E-08 | 0,25459 | 0,799391 | 2,27428 | 0,024397 |
| NM_005548__KARS | 5,76768 | 4,58E-08 | -1,65587 | 0,0998527 | 3,8904 | 0,000151 |
| NM_006826__YWHAQ | 5,74878 | 5,01E-08 | 0,143356 | 0,886203 | 1,9839 | 0,049129 |
| NM_002254__KIF3C | 5,74401 | 5,13E-08 | -3,41282 | 0,0008281 | 2,10981 | 0,036569 |
| NM_002079__GOT1 | 5,74024 | 5,23E-08 | -1,44288 | 0,151153 | 4,38959 | 2,16E-05 |
| NM_004990__MARS | 5,72846 | 5,53E-08 | -1,05167 | 0,294654 | 3,6817 | 0,000325 |
| NM_001071__TYMS | 5,72461 | 5,63E-08 | 0,823624 | 0,41147 | 4,15279 | 5,55E-05 |
| NM_005651__TDO2 | 5,7092 | 6,07E-08 | 1,39882 | 0,163945 | 3,53133 | 0,000553 |
| NM_000688__ALAS1 | 5,70467 | 6,20E-08 | -3,11878 | 0,0021804 | 2,00525 | 0,046772 |
| NM_006086__TUBB4 | 5,70101 | 6,31E-08 | -3,40475 | 0,0008511 | 3,10291 | 0,002299 |
| NM_003449__ZNF173 | 5,69565 | 6,48E-08 | -2,13773 | 0,0341702 | 3,90423 | 0,000144 |
| NM_018685__ANLN | 5,68971 | 6,66E-08 | 4,10042 | 6,76E-05 | 3,66193 | 0,000349 |
| NM_021259__M83 | 5,68255 | 6,90E-08 | 1,57661 | 0,117006 | 4,09778 | 6,87E-05 |
| NM_002768__PCOLN3 | 5,68205 | 6,91E-08 | 0,191185 | 0,848641 | 3,95602 | 0,000118 |
| NM_002801__PSMB10 | 5,68172 | 6,92E-08 | -0,515129 | 0,607226 | 5,05924 | 1,24E-06 |
| NM_005022__PFN1 | 5,67829 | 7,04E-08 | -3,00607 | 0,0031066 | 3,64059 | 0,000376 |
| NM_001826__CKS1 | 5,6742 | 7,18E-08 | 1,97782 | 0,0497936 | 3,90834 | 0,000141 |
| NM_004207__SLC16A3 | 5,67044 | 7,31E-08 | -0,45153 | 0,652264 | 4,78347 | 4,15E-06 |
| NM_001127__AP1B1 | 5,66654 | 7,45E-08 | -4,13296 | 5,95E-05 | 2,72534 | 0,007204 |
| NM_017518__HSXQ28ORF | 5,6547 | 7,88E-08 | -1,09401 | 0,275716 | 3,52059 | 0,000574 |
| NM_001084__PLOD3 | 5,65378 | 7,91E-08 | -5,2691 | 4,72E-07 | 1,28901 | 0,199419 |
| NM_001363__DKC1 | 5,65213 | 7,98E-08 | -0,209901 | 0,834032 | 3,04797 | 0,002733 |
| NM_003345__UBE2I | 5,65156 | 8,00E-08 | 0,652325 | 0,515197 | 3,45085 | 0,00073 |
| AF052151__MTVR | 5,65141 | 8,00E-08 | -1,08208 | 0,280965 | 3,5429 | 0,000531 |
| NM_014390__p100 | 5,64963 | 8,07E-08 | -6,45341 | 1,45E-09 | 1,68433 | 0,09424 |
| NM_004456__EZH2 | 5,64922 | 8,09E-08 | 1,35331 | 0,178006 | 3,9334 | 0,000129 |
| NM_004579__MAP4K2 | 5,64616 | 8,21E-08 | -2,99565 | 0,0032084 | 2,70964 | 0,007536 |
| X52882__TCP1 | 5,64408 | 8,29E-08 | -1,84923 | 0,066406 | 3,17233 | 0,001841 |
| NM_005526__HSF1 | 5,63216 | 8,77E-08 | -1,56442 | 0,11984 | 3,57053 | 0,000482 |
| NM_002268__KPNA4 | 5,62968 | 8,88E-08 | 0,292713 | 0,770149 | 2,40757 | 0,017299 |
| AF238083__SPHK1 | 5,62556 | 9,06E-08 | -3,2866 | 0,0012647 | 3,66187 | 0,000349 |
| AF090913__TMSB10 | 5,62048 | 9,28E-08 | 1,35159 | 0,178555 | 3,69763 | 0,000307 |
| NM_002691__POLD1 | 5,61983 | 9,31E-08 | -1,70483 | 0,0903103 | 4,62562 | 8,12E-06 |
| NM_013260__HCNGP | 5,61945 | 9,32E-08 | 2,58368 | 0,0107366 | 3,09113 | 0,002386 |
| NM_004671__PIASX-BETA | 5,61131 | 9,69E-08 | 0,285736 | 0,775477 | 4,00182 | 9,93E-05 |
| NM_005342__HMG4 | 5,61056 | 9,73E-08 | 1,21463 | 0,226429 | 3,97969 | 0,000108 |
| NM_004712__HGS | 5,59539 | 1,05E-07 | 0,264289 | 0,791922 | 3,55872 | 0,000502 |
| NM_020230__PPAN | 5,59495 | 1,05E-07 | -2,15527 | 0,0327468 | 5,15722 | 7,97E-07 |
| NM_007198__PROSC | 5,58633 | 1,09E-07 | 1,94546 | 0,0536021 | 1,3337 | 0,184365 |
| NM_002764__PRPS1 | 5,58295 | 1,11E-07 | 0,221844 | 0,824739 | 4,4109 | 1,98E-05 |
| NM_005343__HRAS | 5,5719 | 1,17E-07 | 3,86081 | 0,000168 | 5,20067 | 6,55E-07 |
| NM_006409__ARPC1A | 5,56616 | 1,20E-07 | -1,42107 | 0,157386 | 2,49261 | 0,01379 |
| NM_000342__SLC4A1 | 5,56593 | 1,20E-07 | -3,31144 | 0,0011646 | 4,76539 | 4,48E-06 |
| NM_014302__SEC61G | 5,5593 | 1,24E-07 | 2,23848 | 0,026672 | 4,05478 | 8,11E-05 |
| AF100756__LOC51137 | 5,54986 | 1,30E-07 | -1,96151 | 0,0516835 | 3,12273 | 0,002158 |
| NM_003564__TAGLN2 | 5,54694 | 1,32E-07 | -2,15927 | 0,0324295 | 4,04586 | 8,39E-05 |
| NM_012417__RDGBB | 5,54199 | 1,35E-07 | 2,08293 | 0,0389662 | 3,80629 | 0,000206 |
| NM_014452__DR6 | 5,53872 | 1,37E-07 | -1,29762 | 0,196423 | 2,33253 | 0,02103 |
| NM_000075__CDK4 | 5,52841 | 1,44E-07 | -2,38737 | 0,0182233 | 3,41905 | 0,000813 |
| NM_004074__COX8 | 5,52351 | 1,47E-07 | 2,57199 | 0,0110897 | 4,4979 | 1,38E-05 |
| NM_001419__ELAVL1 | 5,51897 | 1,50E-07 | -2,0061 | 0,0466554 | 3,18486 | 0,001768 |
| NM_005745__DXS1357E | 5,51634 | 1,52E-07 | -0,075939 | 0,93957 | 4,82231 | 3,51E-06 |
| NM_020166__MCC-B | 5,51585 | 1,52E-07 | -1,31474 | 0,190617 | 3,42109 | 0,000808 |
| NM_000819__GART | 5,51283 | 1,54E-07 | -3,11387 | 0,0022148 | 2,87893 | 0,004586 |
| NM_013332__HIG2 | 5,50999 | 1,57E-07 | 0,285654 | 0,77554 | 3,70157 | 0,000302 |
| NM_006665__HPSE | 5,50954 | 1,57E-07 | -0,434967 | 0,664216 | 3,27003 | 0,00134 |
| U37689__POLR2H | 5,50289 | 1,62E-07 | 2,80214 | 0,0057515 | 5,20464 | 6,43E-07 |
| NM_006004__UQCRH | 5,49398 | 1,69E-07 | 0,88574 | 0,377185 | 3,58255 | 0,000462 |
| NM_006012__CLPP | 5,49133 | 1,71E-07 | 0,13067 | 0,896213 | 4,24036 | 3,93E-05 |
| NM_004741__P130 | 5,49088 | 1,71E-07 | -0,818058 | 0,41463 | 4,11933 | 6,32E-05 |
| AF234532__MYO10 | 5,48812 | 1,73E-07 | -1,01688 | 0,310859 | 0,944805 | 0,346309 |
| NM_006303__JTV1 | 5,48526 | 1,76E-07 | -1,64588 | 0,101896 | 3,15937 | 0,00192 |
| NM_005662__VDAC3 | 5,47847 | 1,82E-07 | 1,36915 | 0,173013 | 1,99859 | 0,047497 |
| NM_017546__C40 | 5,47263 | 1,87E-07 | -0,257743 | 0,796961 | 1,87132 | 0,063288 |
| U81599__HOXB13 | 5,47179 | 1,87E-07 | 0,0261172 | 0,979199 | 2,36451 | 0,019361 |
| NM_004401__DFFA | 5,46828 | 1,90E-07 | -1,94233 | 0,0539831 | 2,37163 | 0,019006 |
| NM_001694__ATP6L | 5,46724 | 1,91E-07 | -0,755886 | 0,450911 | 4,39675 | 2,10E-05 |
| X75315__HSRNASEB | 5,4518 | 2,06E-07 | -1,06858 | 0,286987 | 4,36738 | 2,36E-05 |
| NM_002610__PDK1 | 5,44286 | 2,14E-07 | -2,3087 | 0,0223352 | 2,78671 | 0,006028 |
| NM_021103__TMSB10 | 5,42237 | 2,36E-07 | 1,3505 | 0,178903 | 4,24452 | 3,86E-05 |
| NM_014317__TPT | 5,42043 | 2,38E-07 | 1,44654 | 0,150126 | 2,94086 | 0,003803 |
| NM_005659__UFD1L | 5,41685 | 2,42E-07 | 0,788923 | 0,431411 | 3,02949 | 0,002895 |
| NM_000057__BLM | 5,41455 | 2,45E-07 | 3,20324 | 0,001662 | 4,12835 | 6,10E-05 |
| NM_006907__PYCR1 | 5,41311 | 2,46E-07 | -0,306748 | 0,759463 | 4,29041 | 3,22E-05 |
| NM_000636__SOD2 | 5,41185 | 2,48E-07 | -2,11093 | 0,036448 | 3,56143 | 0,000497 |
| NM_020365__EIF2B3 | 5,40374 | 2,57E-07 | -1,37661 | 0,170698 | 2,98001 | 0,003374 |
| NM_016577__RAB6B | 5,40029 | 2,62E-07 | -2,36395 | 0,0193712 | 2,8862 | 0,004487 |
| NM_006342__TACC3 | 5,39881 | 2,63E-07 | 0,721236 | 0,471894 | 3,19735 | 0,001698 |
| NM_004269__CRSP8 | 5,39564 | 2,67E-07 | 1,19552 | 0,233783 | 3,21268 | 0,001616 |
| NM_006833__MOV34-34KD | 5,38778 | 2,77E-07 | -0,916001 | 0,361147 | 2,78158 | 0,006119 |
| NM_006636__MTHFD2 | 5,38542 | 2,80E-07 | 0,887551 | 0,376213 | 4,16875 | 5,21E-05 |
| NM_007367__RALY | 5,37955 | 2,88E-07 | -2,63097 | 0,0094093 | 4,08678 | 7,17E-05 |
| NM_004515__ILF2 | 5,37495 | 2,94E-07 | -0,827745 | 0,409139 | 3,43075 | 0,000782 |
| NM_002790__PSMA5 | 5,37131 | 2,99E-07 | 2,73119 | 0,0070728 | 4,71829 | 5,49E-06 |
| D25328__PFKP | 5,36859 | 3,03E-07 | -3,1392 | 0,0020429 | 3,25342 | 0,001415 |
| NM_001152__SLC25A5 | 5,36452 | 3,09E-07 | -0,608194 | 0,543985 | 4,05677 | 8,05E-05 |
| NM_006170__NOL1 | 5,36404 | 3,09E-07 | -2,27129 | 0,0245618 | 2,68858 | 0,008004 |
| NM_005174__ATP5C1 | 5,36241 | 3,12E-07 | 2,03531 | 0,0435921 | 3,71947 | 0,000283 |
| NM_003191__TARS | 5,36096 | 3,14E-07 | 1,96284 | 0,0515271 | 2,72222 | 0,007269 |
| NM_014050__PTD007 | 5,35845 | 3,18E-07 | 3,57983 | 0,0004645 | 3,10311 | 0,002297 |
| NM_003678__PK1,3 | 5,35086 | 3,29E-07 | -2,49702 | 0,0136119 | 1,93539 | 0,054862 |
| NM_004035__ACOX1 | 5,34931 | 3,31E-07 | 2,68668 | 0,0080364 | 3,22691 | 0,001543 |
| NM_006854__KDELR2 | 5,3478 | 3,34E-07 | 0,183906 | 0,854337 | 2,21588 | 0,028237 |
| NM_004523__KNSL1 | 5,34209 | 3,43E-07 | 2,67904 | 0,0082133 | 4,8787 | 2,75E-06 |
| NM_001101__ACTB | 5,3414 | 3,44E-07 | -4,76708 | 4,40E-06 | 1,51607 | 0,131649 |
| NM_013242__AF093680 | 5,33963 | 3,46E-07 | 1,04985 | 0,295487 | 2,78289 | 0,006096 |
| NM_001863__COX6B | 5,33297 | 3,57E-07 | 0,944297 | 0,346547 | 3,46047 | 0,000706 |
| NM_005880__HIRIP4 | 5,32917 | 3,64E-07 | -0,01744 | 0,986109 | 1,79583 | 0,074576 |
| NM_002797__PSMB5 | 5,32689 | 3,67E-07 | 3,03454 | 0,0028434 | 4,30978 | 2,98E-05 |
| NM_000512__GALNS | 5,32581 | 3,69E-07 | -2,30536 | 0,0225265 | 4,5992 | 9,08E-06 |
| AB006198__SART1 | 5,32155 | 3,77E-07 | -3,72526 | 0,0002763 | 3,06402 | 0,002599 |
| NM_007006__CFIM25 | 5,3169 | 3,85E-07 | 0,915822 | 0,36124 | 4,40517 | 2,02E-05 |
| NM_001288__CLIC1 | 5,316 | 3,86E-07 | 0,135373 | 0,8925 | 4,22405 | 4,19E-05 |
| NM_003975__SH2D2A | 5,31131 | 3,95E-07 | -1,83478 | 0,0685326 | 2,52323 | 0,012692 |

**Table B. Pathways correlated positively to 4EBP1 only.**

| p-value | Term | Term ID | Term description | Genes |
| --- | --- | --- | --- | --- |
| 3.18e-02 | GO:0002478 | BP | antigen processing and presentation of exogenous peptide antigen | AP1B1, AP2M1, AP2S1, KIF3C, KIF4A, PSMA5, PSMB10, PSMB2, PSMB5, PSMD11, PSMD2, PSMD8, SEC61G |
| 1.28e-03 | GO:0016032 | BP | viral process | AP1B1, AP2M1, AP2S1, BNIP3, BUB1, CPSF4, GRB2, KARS, NUP155, NUP62, PFN1, POLR2H, PSMA5, PSMB10, PSMB2, PSMB5, PSMD11, PSMD2, PSMD8, RANBP1, SLC25A5, TCEB1, TYMS, UBE2I |
| 4.02e-02 | GO:0045947 | BP | negative regulation of translational initiation | EIF2B3, EIF2C2, EIF4EBP1, EIF4EBP2 |
| 4.93e-13 | GO:0022402 | BP | cell cycle process | ACTR3, ANLN, BLM, BUB1, BUB1B, CCNA2, CDK4, CFL1, CHEK1, EIF4EBP1, EXO1, EZH2, FBXO6, FOXM1, GTSE1, H2AFX, MAD2L1, MAD2L2, MCM2, MCM5, MCM6, MCM7, NUP155, NUP62, OIP5, PHGDH, POLA2, POLD1, POLD2, PRC1, PSMA5, PSMB10, PSMB2, PSMB5, PSMD11, PSMD2, PSMD8, RAD54L, RANBP1, RFC4, SART1, SOX11, TACC3, TTK, TYMS, UBE2I, ZWINT |
| 3.51e-03 | GO:0045333 | BP | cellular respiration | ATP5B, ATP5C1, COX5A, COX8, CS, IDH2, NDUFA9, NDUFB9, SLC25A13, SOD2, UQCRH |
| 4.58e-02 | GO:0006312 | BP | mitotic recombination | BLM, POLA2, POLD1, POLD2, RFC4 |
| 1.30e-16 | GO:0044424 | CC | intracellular part | AARS, ACOX1, ACTB, ACTR3, AF093680, ALAS1, ANLN, AP1B1, AP2M1, AP2S1, ARHGDIA, ARIH1, ARPC1A, ASH2L, ASNS, ATP5B, ATP5C1, ATP5G3, BAK1, BCL2A1, BCR, BLM, BNIP3, BUB1, BUB1B, BYSL, CCNA2, CCT7, CDK4, CEBPG, CFL1, CHAF1A, CHEK1, CIAO1, CLPP, CORO1C, COX5A, COX8, CPSF4, CS, CSK, CSTB, DFFA, DGUOK, DHCR7, DNMT3A, DONSON, DPP3, DRAP1, DRG1, EIF2B3, EIF2C2, EIF4EBP1, EIF5A, ELAVL1, ELOVL1, EWSR1, EXO1, EZH2, FAF1, FBXO6, FOXM1, GALK1, GALNS, GAPD, GARS, GMPS, GOT1, GPI, GPS1, GRB2, GTSE1, GZMB, H2AFX, H2AFZ, HCCS, HGS, HOXB13, HPSE, IDH2, ILF2, INPP4A, KARS, KDELR2, KIF3C, KIF4A, KPNA4, LMNB2, LOC57228, LSM1, MAD2L1, MAD2L2, MAP4K2, MARS, MCM2, MCM5, MCM6, MCM7, METTL1, MLF2, MRPL12, MRPL37, MTCH2, MTHFD2, MYO10, NDUFA9, NDUFB9, NF2, NUP155, NUP62, OIP5, P4HB, PDK1, PDXK, PFKL, PFKP, PFN1, PHGDH, PIR, PKM2, PLOD3, POLA2, POLD1, POLD2, POLR2H, PRC1, PROSC, PRPS1, PSMA5, PSMB10, PSMB2, PSMB5, PSMD11, PSMD2, PSMD8, PTDSS1, PYCR1, RAD54L, RALY, RANBP1, RFC4, RIPK2, RNASEH1, RPN1, S100A11, S100A14, SART1, SEC61G, SH2D2A, SLC16A3, SLC25A13, SLC25A3, SLC25A5, SLC3A2, SLC4A1, SLC7A5, SNN, SOD2, SOX11, SPHK1, SRD5A1, SRM, TACC3, TARS, TCEB1, TCP1, TEAD4, TFG, TMPO, TMSB10, TTK, TYMS, UBE2I, UBQLN1, UFD1L, UQCRH, USP10, USP18, WARS, VDAC3, WDR5, YARS, YWHAQ, ZWINT |
| 4.55e-09 | GO:0005515 | MF | protein binding | ACOX1, ACTB, ACTR3, ANLN, AP1B1, AP2M1, APS, ARHGDIA, ARIH1, ARPC1A, ASH2L, ASNS, ATP5B, BAK1, BCL2A1, BCR, BLM, BNIP3, BUB1, BUB1B, BYSL, CCNA2, CCT7, CDK4, CEBPG, CFL1, CHAF1A, CHEK1, CLPP, CORO1C, CSK, CSTB, DNMT3A, DRAP1, DRG1, EIF2B3, EIF2C2, EIF4EBP1, EIF4EBP2, EIF5A, ELAVL1, ELOVL1, EWSR1, EXO1, EZH2, FAF1, FBXO6, FOXM1, GAPD, GARS, GPI, GRB2, GZMB, H2AFX, H2AFZ, HGS, HPSE, ILF2, KIF3C, KIF4A, LRP8, LSM1, MAD2L1, MAD2L2, MAP4K2, MCM2, MCM5, MCM6, MCM7, METTL1, MPZL1, MRPL12, MYO10, NDUFA9, NF2, NUP62, OIP5, P4HB, PDXK, PFKL, PFN1, PIR, PKM2, PLOD3, POLA2, POLD1, POLD2, PRC1, PRPS1, PSMA5, PSMB5, PSMD2, PYCR1, RAD54L, RANBP1, RFC4, RIPK2, S100A11, S100A14, SART1, SH2D2A, SLC25A5, SLC3A2, SLC4A1, SOD2, SPHK1, SRM, TACC3, TAGLN2, TARS, TCEB1, TCP1, TEAD4, TFG, TMPO, TMSB10, TTK, TYMS, UBE2I, UBQLN1, UFD1L, UQCRH, USP10, WARS, WDR5, YARS, YWHAQ, ZWINT |
| 3.46e-02 | GO:0000049 | MF | tRNA binding | AARS, KARS, MARS, METTL1, YARS |
| 1.99e-03 | GO:0016876 | MF | ligase activity, forming aminoacyl-tRNA and related compounds | AARS, GARS, KARS, MARS, TARS, WARS, YARS |
| 1.72e-24 | BIOGRID:00000 | bi | BioGRID interaction data | AARS, ACOX1, ACTB, ACTR3, AF093680, ALAS1, ANLN, AP1B1, AP2M1, AP2S1, APS, ARHGDIA, ARIH1, ARPC1A, ASH2L, ASNS, ATP5B, ATP5C1, ATP5G3, BAK1, BCL2A1, BCR, BLM, BNIP3, BUB1, BUB1B, BYSL, CCNA2, CCT7, CDK4, CEBPG, CFL1, CHAF1A, CHEK1, CIAO1, CLPP, CORO1C, COX5A, COX8, CPSF4, CS, CSK, CSTB, DFFA, DGUOK, DHCR7, DNMT3A, DONSON, DPP3, DRAP1, DRG1, EIF2B3, EIF2C2, EIF4EBP1, EIF4EBP2, EIF5A, ELAVL1, ELOVL1, EWSR1, EXO1, EZH2, FAF1, FBXO6, FOXM1, GALK1, GALNS, GAPD, GARS, GMPS, GOT1, GPI, GPS1, GRB2, GTSE1, GZMB, H2AFX, H2AFZ, HCCS, HGS, HOXB13, HPSE, IDH2, ILF2, INPP4A, KARS, KDELR2, KIF3C, KIF4A, KPNA4, LMNB2, LOC57228, LRP8, LSM1, LY6E, MAD2L1, MAD2L2, MAP4K2, MARS, MCM2, MCM5, MCM6, MCM7, METTL1, MLF2, MPZL1, MRPL12, MRPL37, MTCH2, MTHFD2, MYO10, NDUFA9, NDUFB9, NF2, NUP155, NUP62, OIP5, P2RY6, P4HB, PDK1, PDXK, PFKL, PFKP, PFN1, PHGDH, PIR, PKM2, PLOD3, POLA2, POLD1, POLD2, POLR2H, PRC1, PROSC, PRPS1 |
| 1.86e-03 | CORUM:387 | co | MCM complex | MCM2, MCM5, MCM6, MCM7 |
| 4.69e-03 | CORUM:1452 | co | MCM2-MCM6-MCM7 complex | MCM2, MCM6, MCM7 |
| 1.84e-02 | CORUM:2792 | co | MCM2-MCM4-MCM6-MCM7 complex | MCM2, MCM6, MCM7 |
| 4.04e-02 | HP:0002936 | hp | Distal sensory impairment | AARS, GARS, KARS, PRPS1, TFG, YARS |
| 1.55e-02 | KEGG:01100 | ke | Metabolic pathways | ACOX1, ALAS1, ASNS, ATP5B, ATP5C1, ATP5G3, COX5A, COX8, CS, DGUOK, DHCR7, DNMT3A, GALK1, GALNS, GAPD, GMPS, GOT1, GPI, HPSE, IDH2, INPP4A, MTHFD2, NDUFA9, NDUFB9, PDXK, PFKL, PFKP, PHGDH, PKM2, POLA2, POLD1, POLD2, POLR2H, PRPS1, PTDSS1, PYCR1, RPN1, SPHK1, SRM, TYMS, UQCRH |
| 4.76e-05 | KEGG:04110 | ke | Cell cycle | BUB1, BUB1B, CCNA2, CDK4, CHEK1, MAD2L1, MAD2L2, MCM2, MCM5, MCM6, MCM7, TTK, YWHAQ |
| 2.86e-02 | KEGG:05012 | ke | Parkinson's disease | ATP5B, ATP5C1, ATP5G3, COX5A, COX8, NDUFA9, NDUFB9, SLC25A5, UQCRH, VDAC3 |
| 8.43e-03 | KEGG:03050 | ke | Proteasome | PSMA5, PSMB10, PSMB2, PSMB5, PSMD11, PSMD2, PSMD8 |
| 1.20e-06 | KEGG:03030 | ke | DNA replication | MCM2, MCM5, MCM6, MCM7, POLA2, POLD1, POLD2, RFC4, RNASEH1 |
| 7.68e-05 | KEGG:01230 | ke | Biosynthesis of amino acids | CS, GAPD, GOT1, IDH2, PFKL, PFKP, PHGDH, PKM2, PRPS1, PYCR1 |
| 3.30e-03 | KEGG:00970 | ke | Aminoacyl-tRNA biosynthesis | AARS, GARS, KARS, MARS, TARS, WARS, YARS |
| 1.58e-03 | KEGG:05016 | ke | Huntington's disease | AP2M1, AP2S1, ATP5B, ATP5C1, ATP5G3, COX5A, COX8, NDUFA9, NDUFB9, POLR2H, SLC25A5, SOD2, UQCRH, VDAC3 |
| 8.02e-03 | KEGG:01200 | ke | Carbon metabolism | CS, GAPD, GPI, IDH2, PFKL, PFKP, PHGDH, PKM2, PRPS1 |
| 4.50e-02 | MI:hsa-miR-423-5p | mi | MI:hsa-miR-423-5p | AP2M1, AP2S1, ATP5B, COX8, DGUOK, DRAP1, EIF4EBP1, FOXM1, GPI, HGS, IDH2, MCM2, MCM5, METTL1, P2RY6, PKM2, RPN1, SLC3A2, SRM |
| 7.39e-06 | REAC:69242 | re | S Phase | CDK4, MCM2, MCM5, MCM6, MCM7, POLA2, POLD1, POLD2, PSMA5, PSMB10, PSMB2, PSMB5, PSMD11, PSMD2, PSMD8, RFC4 |
| 1.80e-03 | REAC:162909 | re | Host Interactions of HIV factors | AP1B1, AP2M1, AP2S1, NUP155, NUP62, PSMA5, PSMB10, PSMB2, PSMB5, PSMD11, PSMD2, PSMD8, RANBP1, SLC25A5, TCEB1 |
| 1.49e-03 | REAC:379716 | re | Cytosolic tRNA aminoacylation | AARS, GARS, KARS, MARS, TARS, WARS, YARS |
| 1.83e-04 | REAC:69620 | re | Cell Cycle Checkpoints | BUB1B, CHEK1, MAD2L1, MCM2, MCM5, MCM6, MCM7, PSMA5, PSMB10, PSMB2, PSMB5, PSMD11, PSMD2, PSMD8, RFC4 |
| 4.21e-02 | REAC:381150 | re | Diabetes pathways | APS, ATP5B, ATP5C1, COX5A, COX8, CS, GAPD, GPI, NDUFA9, NDUFB9, PFKL, PFKP, PKM2, RPN1, SEC61G, SLC25A5, UQCRH |
| 1.07e-02 | REAC:68954 | re | Mcm2-7 is phosphorylated by DDK | MCM2, MCM5, MCM6, MCM7 |
| 1.08e-02 | TF:M00187_4 | tf | Factor: USF; motif: GYCACGTGNC; match class: 4 | ACOX1, ANLN, APS, ARHGDIA, BLM, BNIP3, CDK4, CHAF1A, DHCR7, DNMT3A, DPP3, EIF2C2, ELOVL1, EWSR1, FOXM1, GALNS, GPI, GPS1, H2AFZ, KPNA4, MCM2, MCM7, MTCH2, MTHFD2, PFKL, PSMB10, RAD54L, RANBP1, RFC4, SLC19A1, SLC25A13, SLC4A1, SLC7A5, SNN, SOX11, SRD5A1, SRM, TFG |
| 6.33e-04 | TF:M00919_2 | tf | Factor: E2F; motif: NCSCGCSAAAN; match class: 2 | CHAF1A, CHEK1, COX8, EXO1, MCM5, MCM6, NUP155, RANBP1, SLC5A6, SPHK1, UFD1L |
| 4.78e-03 | TF:M00196_4 | tf | Factor: Sp1; motif: NGGGGGCGGGGYN; match class: 4 | AARS, ACOX1, ACTB, AF093680, ALAS1, ANLN, AP1B1, ARIH1, ASNS, ATP5C1, BLM, BNIP3, BYSL, CCNA2, CCT7, CHEK1, CIAO1, CORO1C, COX5A, CS, CSTB, DHCR7, DONSON, DPP3, DRAP1, DRG1, EIF2B3, EIF2C2, EIF4EBP1, EIF4EBP2, EIF5A, ELOVL1, EXO1, EZH2, GALK1, GALNS, GARS, GMPS, GOT1, GPI, GPS1, GRB2, HCCS, HGS, IDH2, INPP4A, KARS, KDELR2, KIF3C, KIF4A, KPNA4, LOC57228, LRP8, LSM1, LY6E, MAD2L2, MAP4K2, MCM6, MCM7, MLF2, MTCH2, MYO10, NF2, NUP62, P4HB, PDK1, PDXK, PFKL, PFKP, PFN1, PKM2, POLD1, POLD2, POLR2H, PRC1, PRPS1, PSMB10, PSMD11, PSMD2, PTDSS1, PYCR1, RAD54L, RALY, RANBP1, RFC4, RNASEH1, S100A11, SLC16A3, SLC19A1, SLC25A5, SLC3A2, SLC5A6, SLC7A5, SNN, SOD2, SOX11, SPHK1, SRM, TACC3, TAGLN2, TCP1, TEAD4, TFG, TMPO, TMSB10, UBE2I, UBQLN1, WARS, WDR5, YARS, YWHAQ |
| 2.90e-03 | TF:M00516_4 | tf | Factor: E2F; motif: TTTSGCGCGMNR; match class: 4 | COX8, DONSON, EXO1, GARS, MCM5, MCM6, MCM7, MRPL12, MRPL37, NUP155, OIP5, SLC5A6, SPHK1, TCP1 |
| 9.23e-03 | TF:M00050_0 | tf | Factor: E2F; motif: TTTSGCGC; match class: 0 | COX8, DONSON, EXO1, FOXM1, GARS, MCM5, MCM6, MCM7, MRPL12, MRPL37, NUP155, OIP5, PDXK, POLA2, SLC5A6, SPHK1, TCP1 |
| 1.12e-02 | TF:M00931_2 | tf | Factor: Sp1; motif: GGGGCGGGGC; match class: 2 | AARS, ACTB, ALAS1, ANLN, AP1B1, ARIH1, ASNS, ATP5C1, BLM, BNIP3, BYSL, CCNA2, CCT7, CHEK1, CIAO1, CORO1C, CSTB, DHCR7, DPP3, DRAP1, DRG1, EIF2B3, EIF2C2, EIF4EBP1, EIF4EBP2, EIF5A, ELOVL1, EXO1, EZH2, GALK1, GALNS, GARS, GMPS, GPI, GPS1, GRB2, HGS, IDH2, INPP4A, KARS, KDELR2, KIF3C, KIF4A, KPNA4, LRP8, LY6E, MAD2L2, MAP4K2, MCM6, MCM7, MLF2, MTCH2, MYO10, NF2, NUP62, PDXK, PFKL, PFKP, PFN1, PKM2, POLD1, POLD2, POLR2H, PRC1, PSMB10, PSMD2, PYCR1, RAD54L, RALY, RFC4, RNASEH1, S100A11, SLC16A3, SLC19A1, SLC25A5, SLC3A2, SLC5A6, SLC7A5, SNN, SOD2, SOX11, SPHK1, SRM, TACC3, TAGLN2, TCP1, TEAD4, TFG, TMSB10, UBE2I, UBQLN1, WARS, WDR5, YARS |
| 4.65e-03 | TF:M00932_4 | tf | Factor: Sp1; motif: NNGGGGCGGGGNN; match class: 4 | AARS, ACOX1, ACTB, ALAS1, ANLN, AP1B1, AP2S1, ARIH1, ASNS, ATP5C1, BLM, BNIP3, BYSL, CCNA2, CCT7, CFL1, CHEK1, CIAO1, CORO1C, COX5A, CS, CSK, CSTB, DHCR7, DNMT3A, DPP3, DRAP1, DRG1, EIF2B3, EIF2C2, EIF4EBP1, EIF4EBP2, EIF5A, ELOVL1, EXO1, EZH2, GALK1, GALNS, GAPD, GARS, GMPS, GPI, GPS1, GRB2, HGS, HOXB13, IDH2, INPP4A, KARS, KDELR2, KIF3C, KIF4A, KPNA4, LOC57228, LRP8, LY6E, MAD2L2, MAP4K2, MCM6, MCM7, MLF2, MTCH2, MYO10, NF2, NUP62, P4HB, PDK1, PDXK, PFKL, PFKP, PFN1, PKM2, POLD1, POLD2, POLR2H, PRC1, PRPS1, PSMA5, PSMB10, PSMD11, PSMD2, PTDSS1, PYCR1, RAD54L, RALY, RANBP1, RFC4, RNASEH1, S100A11, SLC16A3, SLC19A1, SLC25A5, SLC3A2, SLC5A6, SLC7A5, SNN, SOD2, SOX11, SPHK1, SRM, TACC3, TAGLN2, TCP1, TEAD4, TFG, TMPO, TMSB10, UBE2I, UBQLN1, UQCRH, WARS, WDR5, YARS |
| 1.78e-02 | TF:M00736_1 | tf | Factor: E2F-1:DP-1; motif: TTTCSCGC; match class: 1 | BNIP3, BYSL, CHEK1, CSK, EXO1, FOXM1, GAPD, GARS, MCM5, MCM6, MCM7, MTHFD2, NF2, PDXK, PFN1, POLA2, PRPS1, RANBP1, SLC31A1, SPHK1, TARS, TCP1, TTK, TYMS, UBQLN1 |
| 1.11e-04 | TF:M00431_2 | tf | Factor: E2F-1; motif: TTTSGCGS; match class: 2 | ATP5C1, ATP5G3, CHEK1, CIAO1, COX8, DFFA, DONSON, DRAP1, EXO1, FOXM1, GARS, H2AFZ, MAD2L1, MAD2L2, MCM5, MCM6, MCM7, METTL1, MRPL12, MRPL37, NUP155, OIP5, PDXK, POLA2, PTDSS1, SART1, SLC5A6, SOX11, SPHK1, SRD5A1, TACC3, TCP1, TMPO, UQCRH, WARS, VDAC3, ZWINT |
| 1.93e-07 | TF:M00008_4 | tf | Factor: Sp1; motif: GGGGCGGGGT; match class: 4 | AARS, ACOX1, ACTB, ACTR3, AF093680, ALAS1, ANLN, AP1B1, ARHGDIA, ARIH1, ASNS, ATP5C1, ATP5G3, BCR, BLM, BNIP3, BUB1, BYSL, CCNA2, CCT7, CEBPG, CFL1, CHEK1, CIAO1, CLPP, CORO1C, CPSF4, CS, CSK, CSTB, DGUOK, DHCR7, DNMT3A, DONSON, DPP3, DRAP1, DRG1, EIF2B3, EIF2C2, EIF4EBP1, EIF4EBP2, EIF5A, ELAVL1, ELOVL1, EXO1, EZH2, FAF1, FOXM1, GALK1, GALNS, GAPD, GARS, GMPS, GPI, GPS1, GRB2, GTSE1, H2AFZ, HCCS, HGS, HOXB13, IDH2, INPP4A, KARS, KDELR2, KIF3C, KIF4A, KPNA4, LRP8, LSM1, LY6E, MAD2L1, MAD2L2, MAP4K2, MARS, MCM6, MCM7, METTL1, MLF2, MPZL1, MRPL12, MRPL37, MTCH2, MTHFD2, MYO10, NF2, NUP155, NUP62, OIP5, P2RY6, PDK1, PDXK, PFKL, PFKP, PFN1, PKM2, POLD1, POLD2, POLR2H, PRC1, PRPS1, PSMA5, PSMB10, PSMD11, PSMD2, PSMD8, PTDSS1, PYCR1, RAD54L, RALY, RANBP1, RFC4, RIPK2, RNASEH1, S100A11, S100A14, SART1, SEC61G, SH2D2A, SLC16A3, SLC19A1, SLC25A5, SLC3A2, SLC5A6, SLC7A5, SNN, SOD2, SOX11, SPHK1, SRM, TACC3, TAGLN2, TCP1, TEAD4, TFG, TMPO, TMSB10, TTK, TYMS, UBE2I, UBQLN1, UFD1L, WARS, WDR5, YARS, YWHAQ |
| 9.23e-03 | TF:M00740_0 | tf | Factor: Rb:E2F-1:DP-1; motif: TTTSGCGC; match class: 0 | COX8, DONSON, EXO1, FOXM1, GARS, MCM5, MCM6, MCM7, MRPL12, MRPL37, NUP155, OIP5, PDXK, POLA2, SLC5A6, SPHK1, TCP1 |
| 1.34e-06 | TF:M00803_0 | tf | Factor: E2F; motif: GGCGSG; match class: 0 | AARS, ACOX1, ACTB, AF093680, ALAS1, ANLN, AP2M1, AP2S1, ARHGDIA, ARPC1A, ASH2L, ASNS, ATP5B, ATP5C1, ATP5G3, BAK1, BNIP3, CHAF1A, CHEK1, CIAO1, CLPP, CORO1C, COX5A, COX8, CPSF4, CS, CSK, CSTB, DFFA, DHCR7, DNMT3A, DPP3, DRAP1, DRG1, EIF2B3, EIF2C2, EIF4EBP1, EIF5A, ELAVL1, EWSR1, EXO1, EZH2, FAF1, FBXO6, GALK1, GAPD, GARS, GOT1, GPI, GPS1, GTSE1, H2AFX, H2AFZ, HGS, IDH2, INPP4A, KARS, KDELR2, KIF3C, KIF4A, KPNA4, LMNB2, LOC57228, LRP8, LY6E, MAD2L1, MAD2L2, MAP4K2, MARS, MCM2, MCM5, MCM6, MCM7, METTL1, MLF2, MRPL12, MTCH2, MTHFD2, MYO10, NDUFB9, NF2, NUP155, NUP62, OIP5, P4HB, PDK1, PDXK, PFKL, PFKP, PFN1, PLOD3, POLA2, POLD1, POLD2, POLR2H, PRC1, PROSC, PRPS1, PSMB2, PSMB5, PSMD11, PSMD2, PTDSS1, PYCR1, RALY, RANBP1, RFC4, RIPK2, RNASEH1, RPN1, S100A11, SART1, SEC61G, SLC16A3, SLC19A1, SLC25A13, SLC25A3, SLC25A5, SLC5A6, SLC7A5, SNN, SOD2, SOX11, SPHK1, SRD5A1, SRM, TACC3, TCP1, TEAD4, TMPO, TMSB10, TYMS, UBE2I, UBQLN1, UQCRH, USP10, USP18, WARS, VDAC3, WDR5, YWHAQ, ZWINT |
| 2.41e-02 | TF:M00920_1 | tf | Factor: E2F; motif: NKCGCGCSAAAN; match class: 1 | COX8, EXO1, MCM5, MCM6, NUP155, SLC5A6, SPHK1 |
| 1.71e-02 | TF:M00427_4 | tf | Factor: E2F; motif: TTTSGCGS; match class: 4 | ACTB, AP2S1, ASH2L, ATP5C1, ATP5G3, BLM, CCNA2, CEBPG, CFL1, CHAF1A, CHEK1, CIAO1, COX8, CS, DFFA, DHCR7, DONSON, DPP3, DRAP1, EIF2C2, EXO1, FOXM1, GARS, H2AFZ, MAD2L1, MAD2L2, MCM5, MCM6, MCM7, METTL1, MRPL12, MRPL37, NUP155, OIP5, PDK1, PDXK, POLA2, PTDSS1, RANBP1, SART1, SEC61G, SLC3A2, SLC5A6, SOX11, SPHK1, SRD5A1, TACC3, TARS, TCP1, TMPO, TMSB10, UFD1L, UQCRH, USP18, WARS, VDAC3, WDR5, YWHAQ, ZWINT |
| 1.12e-04 | TF:M00940_4 | tf | Factor: E2F-1; motif: NTTTCGCGCS; match class: 4 | ATP5G3, BYSL, CHEK1, CIAO1, COX8, DONSON, EXO1, FOXM1, GAPD, GARS, H2AFZ, MAD2L2, MCM5, MCM6, MCM7, MRPL12, MRPL37, NF2, NUP155, OIP5, PDXK, POLA2, PTDSS1, RANBP1, SART1, SLC31A1, SLC5A6, SOX11, SPHK1, TARS, TCP1, TTK, TYMS, VDAC3, ZWINT |
| 1.02e-03 | TF:M00426_2 | tf | Factor: E2F; motif: TTTSGCGS; match class: 2 | AP2S1, ASH2L, BLM, CHAF1A, CHEK1, COX8, DHCR7, DONSON, EXO1, FOXM1, GARS, MCM5, MCM6, MCM7, MRPL12, MRPL37, NUP155, OIP5, PDK1, PDXK, POLA2, PTDSS1, RANBP1, SLC5A6, SOX11, SPHK1, TCP1, UFD1L, VDAC3 |
| 3.96e-03 | TF:M00939_2 | tf | Factor: E2F-1; motif: TTTSGCGSG; match class: 2 | AP2S1, CHAF1A, CHEK1, COX8, EXO1, MCM5, MCM6, NUP155, POLA2, RANBP1, SLC5A6, SPHK1, UFD1L |
| 4.52e-03 | TF:M00918_2 | tf | Factor: E2F; motif: TTTSGCGSG; match class: 2 | AP2S1, CHAF1A, CHEK1, COX8, DONSON, EXO1, MCM5, MCM6, MRPL12, MRPL37, NUP155, OIP5, POLA2, RANBP1, SLC5A6, SPHK1, UFD1L |
| 2.78e-02 | TF:M00425_0 | tf | Factor: E2F; motif: TTTCGCGC; match class: 0 | EXO1, FOXM1, GARS, MCM5, MCM6, MCM7, PDXK, POLA2, SPHK1, TCP1 |
| 6.81e-05 | TF:M00428_4 | tf | Factor: E2F-1; motif: NKTSSCGC; match class: 4 | AARS, ACTB, AF093680, ANLN, AP2S1, APS, ARIH1, ARPC1A, ASH2L, ASNS, ATP5C1, ATP5G3, BAK1, BLM, BNIP3, BUB1B, BYSL, CEBPG, CHAF1A, CHEK1, CIAO1, CLPP, CORO1C, COX5A, COX8, CS, CSK, CSTB, DFFA, DHCR7, DNMT3A, DONSON, DPP3, DRAP1, DRG1, EIF2B3, EIF2C2, ELAVL1, EWSR1, EXO1, EZH2, FAF1, FOXM1, GALK1, GALNS, GAPD, GARS, GMPS, GOT1, GPS1, GTSE1, H2AFX, H2AFZ, HGS, IDH2, ILF2, INPP4A, KARS, KIF4A, LOC57228, LSM1, LY6E, MAD2L1, MAD2L2, MCM5, MCM6, MCM7, METTL1, MRPL12, MRPL37, MTHFD2, MYO10, NDUFA9, NDUFB9, NF2, NUP155, OIP5, P2RY6, P4HB, PDK1, PDXK, PFKL, PFKP, PFN1, PIR, PKM2, PLOD3, POLA2, POLD2, POLR2H, PRC1, PRPS1, PSMA5, PSMB2, PSMD2, PTDSS1, PYCR1, RAD54L, RALY, RANBP1, RFC4, RIPK2, RNASEH1, S100A11, SART1, SEC61G, SLC19A1, SLC25A3, SLC31A1, SLC3A2, SLC5A6, SLC7A5, SNN, SOD2, SOX11, SPHK1, SRD5A1, SRM, TACC3, TAGLN2, TARS, TCP1, TEAD4, TMPO, TTK, TYMS, UBE2I, UBQLN1, UFD1L, UQCRH, USP18, WARS, VDAC3, WDR5, YARS, ZWINT |
| 9.81e-03 | TF:M00920_3 | tf | Factor: E2F; motif: NKCGCGCSAAAN; match class: 3 | CHAF1A, CHEK1, COX8, EXO1, MCM5, MCM6, NUP155, RANBP1, SLC5A6, SPHK1, UFD1L |
| 2.78e-02 | TF:M00738_0 | tf | Factor: E2F-4:DP-1; motif: TTTSGCGC; match class: 0 | EXO1, FOXM1, GARS, MCM5, MCM6, MCM7, PDXK, POLA2, SPHK1, TCP1 |
| 5.27e-03 | TF:M00428_1 | tf | Factor: E2F-1; motif: NKTSSCGC; match class: 1 | ACTB, ARIH1, ATP5C1, ATP5G3, CHAF1A, CHEK1, CIAO1, COX8, CS, DFFA, DNMT3A, DONSON, DPP3, DRAP1, EXO1, FOXM1, GARS, GOT1, H2AFZ, HGS, INPP4A, LOC57228, MAD2L1, MAD2L2, MCM5, MCM6, MCM7, METTL1, MRPL12, MRPL37, NUP155, OIP5, P2RY6, PDXK, PFKP, POLA2, PSMD2, PYCR1, SART1, SEC61G, SLC3A2, SLC5A6, SPHK1, SRD5A1, TACC3, TCP1, TMPO, UQCRH, WARS, VDAC3, ZWINT |
| 1.02e-03 | TF:M00427_1 | tf | Factor: E2F; motif: TTTSGCGS; match class: 1 | AP2S1, ASH2L, BLM, CHAF1A, CHEK1, COX8, DHCR7, DONSON, EXO1, FOXM1, GARS, MCM5, MCM6, MCM7, MRPL12, MRPL37, NUP155, OIP5, PDK1, PDXK, POLA2, PTDSS1, RANBP1, SLC5A6, SOX11, SPHK1, TCP1, UFD1L, VDAC3 |
| 2.43e-02 | TF:M00931_4 | tf | Factor: Sp1; motif: GGGGCGGGGC; match class: 4 | AARS, ACTB, ALAS1, ANLN, AP1B1, ARIH1, ASNS, ATP5C1, BLM, BNIP3, BYSL, CCNA2, CCT7, CFL1, CHEK1, CIAO1, CORO1C, COX5A, CSK, CSTB, DHCR7, DNMT3A, DPP3, DRAP1, DRG1, EIF2B3, EIF2C2, EIF4EBP1, EIF4EBP2, EIF5A, ELOVL1, EXO1, EZH2, GALK1, GALNS, GAPD, GARS, GMPS, GPI, GPS1, GRB2, HGS, IDH2, INPP4A, KARS, KDELR2, KIF3C, KIF4A, KPNA4, LOC57228, LRP8, LY6E, MAD2L2, MAP4K2, MCM6, MCM7, MLF2, MTCH2, MYO10, NF2, NUP62, P4HB, PDXK, PFKL, PFKP, PFN1, PKM2, POLD1, POLD2, POLR2H, PRC1, PRPS1, PSMB10, PSMD11, PSMD2, PTDSS1, PYCR1, RAD54L, RALY, RFC4, RNASEH1, S100A11, SLC16A3, SLC19A1, SLC25A5, SLC3A2, SLC5A6, SLC7A5, SNN, SOD2, SOX11, SPHK1, SRM, TACC3, TAGLN2, TCP1, TEAD4, TFG, TMPO, TMSB10, UBE2I, UBQLN1, UQCRH, WARS, WDR5, YARS |

**Table C. Genes inversely correlated to 4EBP1 only.**

| Gene | 4EBP1  t-statistic | 4EBP1  p-value | S6K1  t-statistic | S6K1  p-value | S6K2  t-statistic | S6K2  p-value |
| --- | --- | --- | --- | --- | --- | --- |
| NM_000153__GALC | -5,31563 | 3,87E-07 | 1,7225 | 0,087054 | -3,36047 | 0,000992 |
| NM_001286__CLCN6 | -5,31852 | 3,82E-07 | -1,92028 | 0,056733 | -5,06006 | 1,23E-06 |
| NM_000414__HSD17B4 | -5,31905 | 3,81E-07 | -0,25469 | 0,799317 | -3,63145 | 0,000389 |
| NM_000165__GJA1 | -5,32432 | 3,72E-07 | -2,45126 | 0,015392 | -3,49087 | 0,000636 |
| NM_005544__IRS1 | -5,32591 | 3,69E-07 | -0,83211 | 0,406681 | -3,59246 | 0,000446 |
| NM_016089__SZF1 | -5,33042 | 3,61E-07 | 0,010576 | 0,991576 | -1,69562 | 0,092075 |
| NM_006561__CUGBP2 | -5,34034 | 3,45E-07 | -2,91827 | 0,004066 | -2,47428 | 0,014488 |
| NM_005767__P2Y5 | -5,34128 | 3,44E-07 | 3,55924 | 0,000499 | -5,17095 | 7,49E-07 |
| AK000884__LRRFIP1 | -5,34165 | 3,43E-07 | 2,29414 | 0,02318 | -4,55264 | 1,10E-05 |
| X59405__MCP | -5,34387 | 3,40E-07 | 4,79844 | 3,85E-06 | -1,76873 | 0,079013 |
| NM_004866__SCAMP1 | -5,34532 | 3,37E-07 | 5,21649 | 6,00E-07 | -2,47272 | 0,014549 |
| NM_000495__COL4A5 | -5,35776 | 3,19E-07 | -1,05104 | 0,294942 | -3,95038 | 0,000121 |
| NM_004385__CSPG2 | -5,3687 | 3,03E-07 | -0,23472 | 0,814749 | -3,8269 | 0,000191 |
| NM_002345__LUM | -5,40274 | 2,59E-07 | 2,77654 | 0,0062 | -4,70194 | 5,88E-06 |
| Contig54913_RC__SLC1A1 | -5,40646 | 2,54E-07 | -0,59522 | 0,552599 | -3,35147 | 0,001022 |
| NM_003248__THBS4 | -5,41042 | 2,49E-07 | -0,1356 | 0,892324 | -4,25538 | 3,70E-05 |
| NM_004902__CC1,3 | -5,41953 | 2,39E-07 | 6,09503 | 8,94E-09 | -2,43457 | 0,016108 |
| AF227899__KIAA0117 | -5,42624 | 2,32E-07 | 8,10524 | 1,77E-13 | -2,70878 | 0,007555 |
| NM_000060__BTD | -5,43652 | 2,21E-07 | -2,29513 | 0,023122 | -3,97753 | 0,000109 |
| NM_015642__ZNF288 | -5,45178 | 2,06E-07 | -1,20507 | 0,230087 | -3,29874 | 0,001218 |
| NM_018836__MOT8 | -5,46022 | 1,98E-07 | 0,648103 | 0,517916 | -0,27105 | 0,786734 |
| S80864__CYCL | -5,46209 | 1,96E-07 | 0,370892 | 0,711245 | -3,00973 | 0,003078 |
| Contig52639_RC__RAB5C | -5,47914 | 1,81E-07 | 1,65431 | 0,10017 | -1,47678 | 0,141874 |
| NM_005808__HYA22 | -5,48426 | 1,77E-07 | -1,72692 | 0,086254 | -2,49941 | 0,013539 |
| D26070__ITPR1 | -5,48513 | 1,76E-07 | 1,54118 | 0,125394 | -3,47916 | 0,000662 |
| NM_004354__CCNG2 | -5,48728 | 1,74E-07 | 1,7821 | 0,07677 | -3,45107 | 0,000729 |
| NM_004594__SLC9A5 | -5,49001 | 1,72E-07 | 2,5244 | 0,012637 | -1,75182 | 0,08189 |
| NM_001609__ACADSB | -5,51009 | 1,56E-07 | 2,73611 | 0,006973 | -3,00174 | 0,003155 |
| AF180425__KIAA1105 | -5,52373 | 1,47E-07 | 2,25506 | 0,025587 | -3,86272 | 0,000168 |
| NM_002001__FCER1A | -5,5439 | 1,33E-07 | -1,97263 | 0,050389 | -4,72599 | 5,31E-06 |
| NM_002332__LRP1 | -5,56485 | 1,21E-07 | -3,52881 | 0,000555 | -4,96596 | 1,87E-06 |
| Contig2226_RC__UTRN | -5,56594 | 1,20E-07 | 1,16358 | 0,246454 | -4,23127 | 4,07E-05 |
| AL137438__SEC15L | -5,57371 | 1,16E-07 | 4,6466 | 7,36E-06 | -1,48553 | 0,139545 |
| NM_007184__I-1 | -5,57669 | 1,14E-07 | -4,22131 | 4,21E-05 | -4,01019 | 9,62E-05 |
| Contig50939_RC__MAP4K5 | -5,61342 | 9,59E-08 | 4,36863 | 2,33E-05 | -3,94083 | 0,000125 |
| NM_007168__ABCA8 | -5,63491 | 8,66E-08 | -1,2154 | 0,226136 | -4,22424 | 4,19E-05 |
| NM_018422__DKFZp761K1423 | -5,63705 | 8,57E-08 | -0,1352 | 0,892637 | -4,25615 | 3,69E-05 |
| NM_000254__MTR | -5,64325 | 8,32E-08 | 1,58584 | 0,114896 | -3,82382 | 0,000194 |
| NM_014454__PA26 | -5,66353 | 7,55E-08 | 0,211127 | 0,833077 | -4,39391 | 2,12E-05 |
| AF073770__COT | -5,67119 | 7,28E-08 | -0,00687 | 0,99453 | -4,32203 | 2,83E-05 |
| NM_005777__RBM6 | -5,69241 | 6,58E-08 | -0,08488 | 0,932469 | -2,84849 | 0,005023 |
| Contig2237_RC__SMOC2 | -5,7139 | 5,93E-08 | -1,91835 | 0,056979 | -3,84764 | 0,000177 |
| NM_005802__TP53BPL | -5,73905 | 5,26E-08 | 1,89454 | 0,060091 | -2,0325 | 0,043903 |
| NM_003479__PTP4A2 | -5,75272 | 4,92E-08 | 1,48507 | 0,139638 | -1,81436 | 0,071661 |
| NM_018439__IMPACT | -5,75633 | 4,84E-08 | 2,43537 | 0,016057 | -3,62405 | 0,000399 |
| NM_000426__LAMA2 | -5,75673 | 4,83E-08 | 0,099318 | 0,92102 | -1,3942 | 0,165361 |
| Contig56007_RC__DDXBP1 | -5,76496 | 4,64E-08 | 4,38627 | 2,17E-05 | -3,33991 | 0,001062 |
| AL049932__UBE2N | -5,83417 | 3,31E-08 | 0,189644 | 0,849846 | -2,16606 | 0,031919 |
| NM_006264__PTPN13 | -5,84279 | 3,18E-08 | 2,43816 | 0,015939 | -3,1522 | 0,001964 |
| NM_018474__HT013 | -5,8527 | 3,03E-08 | -0,48084 | 0,631333 | -1,5466 | 0,12411 |
| NM_004349__CBFA2T1 | -5,86781 | 2,81E-08 | -1,71369 | 0,088665 | -4,21253 | 4,38E-05 |
| AB014558__CRY2 | -5,87891 | 2,66E-08 | -4,33434 | 2,68E-05 | -4,5466 | 1,13E-05 |
| NM_004126__GNG11 | -5,88183 | 2,63E-08 | -0,4618 | 0,6449 | -4,64694 | 7,43E-06 |
| NM_004460__FAP | -5,88259 | 2,62E-08 | 0,807114 | 0,420887 | -4,49078 | 1,43E-05 |
| NM_003225__TFF1 | -5,90852 | 2,31E-08 | 1,6575 | 0,099522 | -2,23662 | 0,026817 |
| NM_002023__FMOD | -5,9582 | 1,81E-08 | -2,83581 | 0,005207 | -4,40774 | 2,00E-05 |
| Contig51749_RC__RAI2 | -5,96009 | 1,79E-08 | -2,5993 | 0,010281 | -3,849 | 0,000176 |
| NM_016831__PER3 | -5,98446 | 1,59E-08 | 0,037524 | 0,970117 | -3,31039 | 0,001172 |
| Y07512__PRKG1 | -5,98574 | 1,58E-08 | 3,27854 | 0,001299 | -3,23146 | 0,00152 |
| NM_000662__NAT1 | -5,99426 | 1,51E-08 | -0,25129 | 0,801935 | -3,3242 | 0,001119 |
| NM_006763__BTG2 | -6,00786 | 1,41E-08 | -0,77398 | 0,440168 | -3,76136 | 0,000243 |
| NM_004487__GOLGB1 | -6,01118 | 1,39E-08 | 7,31644 | 1,46E-11 | -1,15923 | 0,248242 |
| NM_000633__BCL2 | -6,04236 | 1,19E-08 | 0,838817 | 0,402916 | -4,68995 | 6,19E-06 |
| NM_002222__ITPR1 | -6,07129 | 1,03E-08 | 0,634944 | 0,526439 | -3,81311 | 0,000201 |
| NM_001202__BMP4 | -6,07887 | 9,93E-09 | 1,32892 | 0,185906 | -2,25884 | 0,025365 |
| NM_007373__SHOC2 | -6,11956 | 8,11E-09 | 6,41367 | 1,78E-09 | -1,59962 | 0,11183 |
| AF279865__KIF13B | -6,12305 | 7,97E-09 | -2,23247 | 0,027075 | -3,64603 | 0,000369 |
| NM_016348__C5ORF4 | -6,16076 | 6,60E-09 | -1,35603 | 0,177141 | -3,67701 | 0,00033 |
| U50534__13CDNA73 | -6,16436 | 6,48E-09 | -0,61135 | 0,541901 | -4,50232 | 1,36E-05 |
| NM_014483__RBMS3 | -6,17272 | 6,21E-09 | -1,84264 | 0,067369 | -4,86005 | 2,98E-06 |
| NM_001656__ARFD1 | -6,19151 | 5,65E-09 | 7,61577 | 2,80E-12 | -3,91507 | 0,000138 |
| NM_003862__FGF18 | -6,23389 | 4,56E-09 | -2,73686 | 0,006958 | -4,56414 | 1,05E-05 |
| Contig78_RC__SLAP | -6,29207 | 3,40E-09 | 4,05534 | 8,04E-05 | -3,23693 | 0,001493 |
| NM_002293__LAMC1 | -6,30554 | 3,18E-09 | -1,78893 | 0,075658 | -3,29369 | 0,001239 |
| AJ224741__MATN3 | -6,30713 | 3,15E-09 | 0,336175 | 0,737212 | -4,91162 | 2,38E-06 |
| U56725__HSPA2 | -6,33038 | 2,80E-09 | -1,27947 | 0,20272 | -2,69402 | 0,007881 |
| NM_006531__TG737 | -6,35239 | 2,50E-09 | 1,60297 | 0,111059 | -3,33265 | 0,001088 |
| NM_006475__OSF-2 | -6,35378 | 2,48E-09 | 2,06686 | 0,040478 | -4,36848 | 2,35E-05 |
| NM_003022__SH3BGRL | -6,37242 | 2,26E-09 | 1,24683 | 0,214416 | -2,88773 | 0,004467 |
| NM_016444__ZNF226 | -6,40967 | 1,87E-09 | 4,97532 | 1,77E-06 | -3,38032 | 0,000927 |
| NM_014057__OGN | -6,45139 | 1,51E-09 | 1,48237 | 0,140354 | -4,24923 | 3,79E-05 |
| NM_007035__KERA | -6,45431 | 1,48E-09 | 0,623954 | 0,533612 | -2,01912 | 0,045293 |
| NM_005899__M17S2 | -6,49529 | 1,20E-09 | 2,28352 | 0,023813 | -3,03631 | 0,002834 |
| NM_003760__EIF4G3 | -6,65371 | 5,28E-10 | 2,94151 | 0,003788 | -2,31938 | 0,021752 |
| NM_003239__TGFB3 | -6,67224 | 4,79E-10 | -1,84624 | 0,066842 | -4,61234 | 8,59E-06 |
| AL359052__ITGBL1 | -6,67949 | 4,61E-10 | -0,81078 | 0,418785 | -3,87339 | 0,000161 |
| U79271__SDCCAG8 | -6,72679 | 3,60E-10 | -1,25446 | 0,211639 | -4,45488 | 1,65E-05 |
| Contig52684__LEPR | -6,73561 | 3,44E-10 | -0,41425 | 0,679287 | -4,50545 | 1,34E-05 |
| Contig57091_RC__ITM2B | -6,74125 | 3,34E-10 | 5,83539 | 3,23E-08 | -4,11163 | 6,51E-05 |
| NM_000125__ESR1 | -6,77066 | 2,86E-10 | 1,98353 | 0,049146 | -3,17176 | 0,001845 |
| NM_012096__APPL | -6,79284 | 2,54E-10 | 2,5631 | 0,011365 | -3,66503 | 0,000345 |
| NM_007195__POLI | -6,87326 | 1,66E-10 | 2,59927 | 0,010282 | -4,89828 | 2,52E-06 |
| NM_003199__TCF4 | -6,91898 | 1,30E-10 | 0,092572 | 0,926368 | -5,03169 | 1,40E-06 |
| NM_020347__LZTFL1 | -6,99987 | 8,46E-11 | 2,34782 | 0,020198 | -3,2316 | 0,001519 |
| NM_005778__RBM5 | -7,08923 | 5,24E-11 | 0,705883 | 0,481363 | -4,36284 | 2,40E-05 |
| NM_019597__HNRPH2 | -7,09758 | 5,01E-11 | 3,02387 | 0,00294 | -4,43042 | 1,83E-05 |
| NM_001280__CIRBP | -7,10753 | 4,75E-11 | 0,208726 | 0,834947 | -3,8403 | 0,000182 |
| NM_000332__SCA1 | -7,13253 | 4,15E-11 | 0,959967 | 0,338627 | -4,57738 | 9,95E-06 |
| NM_016337__RNB6 | -7,14857 | 3,80E-11 | -0,91074 | 0,363905 | -2,8592 | 0,004865 |
| NM_006197__PCM1 | -7,2446 | 2,26E-11 | 4,34143 | 2,60E-05 | -2,94904 | 0,00371 |
| AL049470__HYPB | -7,29787 | 1,69E-11 | 3,48544 | 0,000646 | -4,53284 | 1,20E-05 |
| NM_005935__MLLT2 | -7,32929 | 1,42E-11 | 1,33121 | 0,185153 | -4,30025 | 3,09E-05 |
| NM_020974__CEGP1 | -7,39173 | 1,01E-11 | -0,44489 | 0,657046 | -4,38126 | 2,23E-05 |
| AL137540__NTN4 | -7,39258 | 1,01E-11 | -1,1265 | 0,261765 | -3,53387 | 0,000548 |
| NM_001552__IGFBP4 | -7,60269 | 3,16E-12 | -3,6596 | 0,00035 | -4,34675 | 2,57E-05 |
| NM_006460__HIS1 | -7,61077 | 3,02E-12 | 1,19186 | 0,235211 | -3,76762 | 0,000238 |
| NM_004791__ITGBL1 | -7,83606 | 8,60E-13 | 0,46679 | 0,641332 | -5,0538 | 1,27E-06 |
| NM_016441__CRIM1 | -7,92728 | 5,15E-13 | -1,2752 | 0,204223 | -4,75235 | 4,74E-06 |
| AF167706__CRIM1 | -8,16655 | 1,33E-13 | -1,1671 | 0,245034 | -4,78226 | 4,17E-06 |
| NM_015484__P29 | -8,2306 | 9,20E-14 | 1,86299 | 0,064432 | -2,76164 | 0,006485 |
| NM_005824__P37NB | -8,26492 | 7,56E-14 | 0,44533 | 0,656728 | -4,69783 | 5,98E-06 |
| NM_001380__DOCK1 | -8,86561 | 2,34E-15 | -0,54965 | 0,58338 | -4,09526 | 6,94E-05 |

**Table D. Pathways inversely correlated to 4EBP1 only.**

| p-value | Term | Term ID | Term description | Genes |
| --- | --- | --- | --- | --- |
| 1.47e-04 | GO:0030198 | BP | extracellular matrix organization | BMP4, COL4A5, FAP, FMOD, LAMA2, LAMC1, LUM, MATN3, NTN4, SMOC2, TGFB3 |
| 3.09e-03 | GO:0048468 | BP | cell development | BCL2, BMP4, BTG2, COL4A5, DOCK1, ESR1, FGF18, GJA1, HSD17B4, HSPA2, KIF13B, LAMA2, LAMC1, NTN4, PCM1, PRKG1, SLIT2, TCF4, TGFB3, UTRN |
| 4.62e-02 | GO:0048699 | BP | generation of neurons | BCL2, BMP4, BTG2, COL4A5, DOCK1, GJA1, KIF13B, LAMA2, LAMC1, NTN4, PCM1, PRKG1, SLIT2, TCF4, UTRN |
| 1.72e-02 | GO:0022612 | BP | gland morphogenesis | BCL2, BMP4, ESR1, NTN4, SLIT2, TGFB3 |
| 1.54e-04 | GO:0042340 | BP | keratan sulfate catabolic process | FMOD, KERA, LUM, OGN |
| 4.99e-02 | GO:0045844 | BP | positive regulation of striated muscle tissue development | BCL2, BMP4, GJA1 |
| 2.99e-02 | GO:0043202 | CC | lysosomal lumen | FMOD, GALC, KERA, LUM, OGN |
| 1.06e-06 | GO:0031012 | CC | extracellular matrix | BMP4, COL4A5, FMOD, KERA, LAMA2, LAMC1, LUM, MATN3, NTN4, OGN, SMOC2, SPARCL1, TGFB3, THBS4 |
| 1.99e-02 | KEGG:05200 | ke | Pathways in cancer | BCL2, BMP4, COL4A5, FGF18, LAMA2, LAMC1, CBFA2T1, TGFB3 |
| 3.50e-02 | KEGG:04151 | ke | PI3K-Akt signaling pathway | BCL2, COL4A5, FGF18, GNG11, IRS1, LAMA2, LAMC1, THBS4 |
