# Supplementary material for: Revealing Different Roles of the mTOR-Targets S6K1 and S6K2 in Breast Cancer by Expression Profiling and Structural Analysis
Source: PLoS One. 2015 Dec 23;10(12):e0145013. doi: 10.1371/journal.pone.0145013 (PMC4689523; doi:10.1371/journal.pone.0145013)
Supplement: S8 Table — Genes upregulated in response to both S6K1 and S6K2 siRNA (Table A). Pathways upregulated in response to both S6K1 and S6K2 siRNA (Table B). Genes downregulated in response to both S6K1 and S6K2 siRNA (Table C). (DOCX) [file pone.0145013.s012.docx]

**Table A. Genes upregulated in response to both S6K1 and S6K2 siRNA.**

| Transcripts Cluster ID | Gene symbol | Gene description | S6K1 siRNA  Fold change | S6K1 siRNA  p-value | S6K2 siRNA  Fold change | S6K2 siRNA  p-value |
| --- | --- | --- | --- | --- | --- | --- |
| 17109714 | SCARNA9L | small Cajal body-specific RNA 9-like | 0,80 | 0,0024 | 0,41 | 0,0212 |
| 16830577 | CD68 |  | 0,66 | 0,0032 | 0,34 | 0,0089 |
| 16868443 | ZNF562 | zinc finger protein 562 | 0,57 | 0,0464 | 0,5 | 0,0158 |
| 16967875 | PARM1 | prostate androgen-regulated mucin-like protein 1 | 0,51 | 0,0286 | 0,25 | 0,0252 |
| 17096728 | ABCA1 | ATP-binding cassette, sub-family A (ABC1), member 1 | 0,51 | 0,0039 | 0,34 | 0,0044 |
| 16777309 | ZDHHC20 | zinc finger, DHHC-type containing 20 | 0,48 | 0,0366 | 0,6 | 0,0181 |
| 17066065 | SLC7A2 | solute carrier family 7 (cationic amino acid transporter, y+ system), member 2 | 0,48 | 0,0125 | 0,33 | 0,0337 |
| 16937855 | FBLN2 | fibulin 2 | 0,47 | 0,0293 | 0,36 | 0,0409 |
| 16723175 | ARL14EP | ADP-ribosylation factor-like 14 effector protein | 0,47 | 0,0062 | 0,37 | 0,0042 |
| 16974968 | SEL1L3 | sel-1 suppressor of lin-12-like 3 (C. elegans) | 0,46 | 0,0086 | 0,45 | 0,0130 |
| 16873049 | CADM4 | cell adhesion molecule 4 | 0,44 | 0,0468 | 0,49 | 0,0492 |
| 16735557 | SCUBE2 | signal peptide, CUB domain, EGF-like 2 | 0,44 | 0,0092 | 0,39 | 0,0164 |
| 16725619 | SDHAF2 | succinate dehydrogenase complex assembly factor 2 | 0,43 | 0,0228 | 0,45 | 0,0204 |
| 16875274 | TFPT | TCF3 (E2A) fusion partner (in childhood Leukemia) | 0,43 | 0,0482 | 0,34 | 0,0303 |
| 16954576 | VPRBP | Vpr (HIV-1) binding protein | 0,41 | 0,0490 | 0,46 | 0,0211 |
| 17001100 | NR3C1 | nuclear receptor subfamily 3, group C, member 1 (glucocorticoid receptor) | 0,41 | 0,0428 | 0,29 | 0,0472 |
| 16662123 | ZBTB8A | zinc finger and BTB domain containing 8A | 0,40 | 0,0455 | 0,4 | 0,0328 |
| 17110237 | CASK | calcium/calmodulin-dependent serine protein kinase (MAGUK family) | 0,40 | 0,0296 | 0,5 | 0,0247 |
| 16887993 | MTX2 | metaxin 2 | 0,39 | 0,0069 | 0,33 | 0,0079 |
| 16926029 | TFF3 | trefoil factor 3 (intestinal) | 0,39 | 0,0126 | 0,3 | 0,0099 |
| 16999259 | CEP120 | centrosomal protein 120kDa | 0,38 | 0,0243 | 0,28 | 0,0358 |
| 16841542 | COX10-AS1 | COX10 antisense RNA 1 (non-protein coding) | 0,38 | 0,0383 | 0,34 | 0,0271 |
| 16836457 | RAD51C | RAD51 homolog C (S. cerevisiae) | 0,37 | 0,0046 | 0,15 | 0,0249 |
| 17061048 | UPK3BL\|POLR2J3 | uroplakin 3B-like \| polymerase (RNA) II (DNA directed) polypeptide J3 | 0,37 | 0,0418 | 0,62 | 0,0441 |
| 16723614 | CD44 |  | 0,37 | 0,0249 | 0,26 | 0,0250 |
| 17014562 | QKI |  | 0,36 | 0,0229 | 0,42 | 0,0004 |
| 16698556 | SLC41A1 | solute carrier family 41, member 1 | 0,36 | 0,0247 | 0,23 | 0,0230 |
| 16860678 | LSM14A |  | 0,36 | 0,0374 | 0,39 | 0,0006 |
| 16687418 | TMEM48 | transmembrane protein 48 | 0,36 | 0,0010 | 0,42 | 0,0125 |
| 16989054 | RAD50 |  | 0,35 | 0,0361 | 0,28 | 0,0191 |
| 16771962 | SBNO1 | strawberry notch homolog 1 (Drosophila) | 0,35 | 0,0108 | 0,37 | 0,0217 |
| 16943548 | ALCAM | activated leukocyte cell adhesion molecule | 0,35 | 0,0076 | 0,38 | 0,0145 |
| 16692724 | ANP32E | acidic (leucine-rich) nuclear phosphoprotein 32 family, member E | 0,35 | 0,0346 | 0,38 | 0,0099 |
| 16700370 | PGBD5 | piggyBac transposable element derived 5 | 0,34 | 0,0093 | 0,4 | 0,0111 |
| 16671457 | IL6R | interleukin 6 receptor | 0,34 | 0,0353 | 0,21 | 0,0220 |
| 16940110 | SACM1L | SAC1 suppressor of actin mutations 1-like (yeast) | 0,34 | 0,0329 | 0,28 | 0,0201 |
| 16844356 | TNS4 | tensin 4 | 0,34 | 0,0129 | 0,49 | 0,0131 |
| 16918996 | SOGA1 | suppressor of glucose, autophagy associated 1 | 0,33 | 0,0361 | 0,28 | 0,0253 |
| 16929509 | HMGXB4 | HMG box domain containing 4 | 0,33 | 0,0243 | 0,31 | 0,0065 |
| 16730340 | CEP57 | centrosomal protein 57kDa | 0,33 | 0,0167 | 0,19 | 0,0148 |
| 16922292 | SON |  | 0,33 | 0,0116 | 0,39 | 0,0483 |
| 17093762 | TLN1 | talin 1 | 0,32 | 0,0107 | 0,41 | 0,0231 |
| 17000079 | SAR1B | SAR1 homolog B (S. cerevisiae) | 0,32 | 0,0426 | 0,34 | 0,0096 |
| 16822868 | MSRB1 | methionine sulfoxide reductase B1 | 0,32 | 0,0061 | 0,3 | 0,0489 |
| 16951601 | TOP2B | topoisomerase (DNA) II beta 180kDa | 0,32 | 0,0165 | 0,33 | 0,0017 |
| 16757831 | CCDC64 | coiled-coil domain containing 64 | 0,32 | 0,0184 | 0,35 | 0,0268 |
| 16906534 | STAT1 | signal transducer and activator of transcription 1, 91kDa | 0,31 | 0,0456 | 0,3 | 0,0346 |
| 16853234 | ATP9B | ATPase, class II, type 9B | 0,31 | 0,0037 | 0,33 | 0,0200 |
| 16766318 | PRIM1 | primase, DNA, polypeptide 1 (49kDa) | 0,31 | 0,0106 | 0,36 | 0,0008 |
| 16917173 | MKKS | McKusick-Kaufman syndrome | 0,31 | 0,0189 | 0,29 | 0,0325 |
| 16822356 | LUC7L | LUC7-like (S. cerevisiae) | 0,31 | 0,0388 | 0,32 | 0,0125 |
| 16964731 | LOC93622 | Morf4 family associated protein 1-like 1 pseudogene | 0,31 | 0,0292 | 0,54 | 0,0161 |
| 16670391 | BOLA1 | bolA homolog 1 (E. coli) | 0,31 | 0,0264 | 0,26 | 0,0196 |
| 17084936 | ZCCHC7 | zinc finger, CCHC domain containing 7 | 0,31 | 0,0393 | 0,22 | 0,0296 |
| 16754177 | TMEM19 | transmembrane protein 19 | 0,30 | 0,0008 | 0,27 | 0,0082 |
| 16906440 | OSGEPL1 | O-sialoglycoprotein endopeptidase-like 1 | 0,30 | 0,0067 | 0,27 | 0,0411 |
| 17112269 | ATRX | alpha thalassemia/mental retardation syndrome X-linked | 0,30 | 0,0355 | 0,36 | 0,0389 |
| 16926011 | ZNF295 | zinc finger protein 295 | 0,30 | 0,0196 | 0,34 | 0,0446 |
| 16698425 | RBBP5 | retinoblastoma binding protein 5 | 0,30 | 0,0337 | 0,28 | 0,0059 |
| 16972396 | GALNT7 | UDP-N-acetyl-alpha-D-galactosamine:polypeptide N-acetylgalactosaminyltransferase 7 (GalNAc-T7) | 0,30 | 0,0129 | 0,17 | 0,0350 |
| 16675354 | CDC73 | cell division cycle 73, Paf1/RNA polymerase II complex component, homolog (S. cerevisiae) | 0,30 | 0,0417 | 0,26 | 0,0054 |
| 17088576 | LOC100288842 | UDP-GlcNAc:betaGal beta-1,3-N-acetylglucosaminyltransferase 5 pseudogene | 0,30 | 0,0077 | 0,48 | 0,0004 |
| 16667608 | HIAT1 | hippocampus abundant transcript 1 | 0,30 | 0,0331 | 0,33 | 0,0478 |
| 17010991 | CASP8AP2 | caspase 8 associated protein 2 | 0,29 | 0,0147 | 0,32 | 0,0296 |
| 16786243 | C14orf169 | chromosome 14 open reading frame 169 | 0,29 | 0,0303 | 0,24 | 0,0460 |
| 16708468 | BTRC | beta-transducin repeat containing E3 ubiquitin protein ligase | 0,29 | 0,0345 | 0,32 | 0,0118 |
| 16714798 | JMJD1C | jumonji domain containing 1C | 0,29 | 0,0048 | 0,31 | 0,0070 |
| 17055937 | OSBPL3 | oxysterol binding protein-like 3 | 0,29 | 0,0043 | 0,21 | 0,0161 |
| 16789258 | KLC1 | kinesin light chain 1 | 0,29 | 0,0219 | 0,18 | 0,0390 |
| 17024578 | PPIL4 | peptidylprolyl isomerase (cyclophilin)-like 4 | 0,28 | 0,0220 | 0,25 | 0,0170 |
| 16667037 | CDC7 | cell division cycle 7 homolog (S. cerevisiae) | 0,28 | 0,0145 | 0,32 | 0,0199 |
| 16857315 | SAFB | scaffold attachment factor B | 0,28 | 0,0372 | 0,36 | 0,0000 |
| 16821614 | COX4I1 | cytochrome c oxidase subunit IV isoform 1 | 0,28 | 0,0135 | 0,2 | 0,0063 |
| 16887702 | ITGA6 | integrin, alpha 6 | 0,28 | 0,0225 | 0,2 | 0,0005 |
| 16772004 | RILPL1 | Rab interacting lysosomal protein-like 1 | 0,28 | 0,0047 | 0,4 | 0,0119 |
| 16732856 | TBRG1 | transforming growth factor beta regulator 1 | 0,28 | 0,0018 | 0,21 | 0,0074 |
| 16914469 | SLC2A10 | solute carrier family 2 (facilitated glucose transporter), member 10 | 0,27 | 0,0108 | 0,25 | 0,0041 |
| 16740797 | MRPL11 | mitochondrial ribosomal protein L11 | 0,27 | 0,0020 | 0,43 | 0,0173 |
| 16781248 | DCUN1D2 | DCN1, defective in cullin neddylation 1, domain containing 2 (S. cerevisiae) | 0,27 | 0,0271 | 0,33 | 0,0287 |
| 16890424 | SPAG16 | sperm associated antigen 16 | 0,27 | 0,0335 | 0,29 | 0,0178 |
| 16683061 | USP48 | ubiquitin specific peptidase 48 | 0,27 | 0,0188 | 0,38 | 0,0290 |
| 16909638 | USP40 | ubiquitin specific peptidase 40 | 0,26 | 0,0287 | 0,38 | 0,0348 |
| 16979389 | MAD2L1 | MAD2 mitotic arrest deficient-like 1 (yeast) | 0,26 | 0,0027 | 0,21 | 0,0196 |
| 16902570 | WDR33\|SFT2D3 | WD repeat domain 33 \| SFT2 domain containing 3 | 0,26 | 0,0154 | 0,3 | 0,0108 |
| 16824690 | ERI2 | ERI1 exoribonuclease family member 2 | 0,26 | 0,0268 | 0,28 | 0,0130 |
| 16969009 | PDLIM5 | PDZ and LIM domain 5 | 0,25 | 0,0263 | 0,26 | 0,0098 |
| 16668891 | LRIG2 | leucine-rich repeats and immunoglobulin-like domains 2 | 0,25 | 0,0023 | 0,42 | 0,0104 |
| 16685482 | YRDC | yrdC domain containing (E. coli) | 0,25 | 0,0202 | 0,36 | 0,0386 |
| 16816479 | CCP110 | centriolar coiled coil protein 110kDa | 0,25 | 0,0289 | 0,25 | 0,0157 |
| 16842766 | SDF2 | stromal cell-derived factor 2 | 0,25 | 0,0162 | 0,22 | 0,0498 |
| 17098642 | ST6GALNAC6 | ST6 (alpha-N-acetyl-neuraminyl-2,3-beta-galactosyl-1,3)-N-acetylgalactosaminide alpha-2,6-sialyltransferase 6 | 0,25 | 0,0312 | 0,19 | 0,0021 |
| 16838698 | RPTOR | regulatory associated protein of MTOR, complex 1 | 0,25 | 0,0051 | 0,28 | 0,0348 |
| 16964764 | TBC1D14 | TBC1 domain family, member 14 | 0,25 | 0,0278 | 0,23 | 0,0063 |
| 16970673 | PHF17 | PHD finger protein 17 | 0,25 | 0,0127 | 0,4 | 0,0188 |
| 16854904 | PSTPIP2 | proline-serine-threonine phosphatase interacting protein 2 | 0,24 | 0,0117 | 0,29 | 0,0073 |
| 16917655 | CRNKL1 | crooked neck pre-mRNA splicing factor-like 1 (Drosophila) | 0,24 | 0,0426 | 0,25 | 0,0060 |
| 17086784 | CENPP | centromere protein P | 0,24 | 0,0310 | 0,34 | 0,0209 |
| 16824143 | RRN3 |  | 0,24 | 0,0152 | 0,28 | 0,0016 |
| 16697438 | UCHL5 | ubiquitin carboxyl-terminal hydrolase L5 | 0,23 | 0,0397 | 0,21 | 0,0346 |
| 16861647 | KCNK6 | potassium channel, subfamily K, member 6 | 0,23 | 0,0186 | 0,23 | 0,0176 |
| 16974779 | GPR125 | G protein-coupled receptor 125 | 0,23 | 0,0206 | 0,19 | 0,0134 |
| 16923491 | PWP2 |  | 0,23 | 0,0036 | 0,19 | 0,0183 |
| 16918832 | NFS1 |  | 0,23 | 0,0311 | 0,38 | 0,0154 |
| 16937563 | BRK1\|LOC100653323 | BRICK1, SCAR/WAVE actin-nucleating complex subunit \| uncharacterized LOC100653323 | 0,23 | 0,0405 | 0,15 | 0,0168 |
| 16778433 | ELF1 | E74-like factor 1 (ets domain transcription factor) | 0,23 | 0,0141 | 0,15 | 0,0333 |
| 16852206 | HAUS1 | HAUS augmin-like complex, subunit 1 | 0,23 | 0,0350 | 0,35 | 0,0422 |
| 16686140 | MED8 | mediator complex subunit 8 | 0,23 | 0,0284 | 0,29 | 0,0140 |
| 17021385 | SYNCRIP | synaptotagmin binding, cytoplasmic RNA interacting protein | 0,23 | 0,0117 | 0,25 | 0,0084 |
| 17023592 | MED23 | mediator complex subunit 23 | 0,23 | 0,0443 | 0,21 | 0,0115 |
| 16820289 | NUTF2\|LOC128322 | nuclear transport factor 2 \| nuclear transport factor 2-like | 0,22 | 0,0077 | 0,38 | 0,0008 |
| 16706586 | C10orf57 | chromosome 10 open reading frame 57 | 0,22 | 0,0262 | 0,32 | 0,0132 |
| 16770733 | MED13L | mediator complex subunit 13-like | 0,22 | 0,0263 | 0,2 | 0,0341 |
| 17015434 | MUTED\|EEF1E1-MUTED\|MUTED-TXNDC5 | muted homolog (mouse) \| EEF1E1-MUTED readthrough \| MUTED-TXNDC5 readthrough (non-protein coding) \| thioredoxin domain containing 5 (endoplasmic reticulum) \| eukaryotic translation elongation factor 1 epsilon 1 | 0,22 | 0,0185 | 0,33 | 0,0002 |
| 16667011 | ZNF326 | zinc finger protein 326 | 0,22 | 0,0373 | 0,38 | 0,0191 |
| 17062915 | TSGA13\|COPG2 | testis specific, 13 \| coatomer protein complex, subunit gamma 2 | 0,22 | 0,0401 | 0,23 | 0,0284 |
| 17053019 | CUL1 | cullin 1 | 0,22 | 0,0026 | 0,21 | 0,0070 |
| 17061374 | ORC5 | origin recognition complex, subunit 5 | 0,21 | 0,0360 | 0,24 | 0,0451 |
| 16818672 | LONP2 | lon peptidase 2, peroxisomal | 0,21 | 0,0452 | 0,23 | 0,0310 |
| 16886105 | HNMT | histamine N-methyltransferase | 0,21 | 0,0481 | 0,2 | 0,0167 |
| 16886818 | TANC1 | tetratricopeptide repeat, ankyrin repeat and coiled-coil containing 1 | 0,21 | 0,0427 | 0,29 | 0,0065 |
| 16789509 | SIVA1 |  | 0,21 | 0,0077 | 0,25 | 0,0084 |
| 16715031 | SLC25A16 | solute carrier family 25 (mitochondrial carrier; Graves disease autoantigen), member 16 | 0,21 | 0,0024 | 0,46 | 0,0310 |
| 16915804 | C20orf11 | chromosome 20 open reading frame 11 | 0,21 | 0,0011 | 0,24 | 0,0137 |
| 16847996 | HELZ | helicase with zinc finger | 0,20 | 0,0311 | 0,19 | 0,0373 |
| 16681323 | RERE | arginine-glutamic acid dipeptide (RE) repeats | 0,20 | 0,0023 | 0,28 | 0,0409 |
| 16817090 | RBBP6 | retinoblastoma binding protein 6 | 0,20 | 0,0413 | 0,26 | 0,0256 |
| 16802795 | NEO1 | neogenin 1 | 0,20 | 0,0460 | 0,32 | 0,0189 |
| 17023308 | HDDC2 | HD domain containing 2 | 0,20 | 0,0110 | 0,28 | 0,0182 |
| 16753498 | TBK1 | TANK-binding kinase 1 | 0,20 | 0,0375 | 0,35 | 0,0179 |
| 16732067 | CCDC84 | coiled-coil domain containing 84 | 0,20 | 0,0226 | 0,25 | 0,0231 |
| 16780509 | DOCK9 | dedicator of cytokinesis 9 | 0,19 | 0,0080 | 0,27 | 0,0273 |
| 16669963 | GPR89C\|GPR89A\|GPR89B | G protein-coupled receptor 89C \| G protein-coupled receptor 89A \| G protein-coupled receptor 89B | 0,19 | 0,0013 | 0,25 | 0,0019 |
| 16868564 | EIF3G | eukaryotic translation initiation factor 3, subunit G | 0,19 | 0,0354 | 0,22 | 0,0178 |
| 17102668 | ATP6AP2 | ATPase, H+ transporting, lysosomal accessory protein 2 | 0,19 | 0,0434 | 0,21 | 0,0092 |
| 17003180 | RAB24\|MXD3 | RAB24, member RAS oncogene family \| MAX dimerization protein 3 | 0,19 | 0,0052 | 0,28 | 0,0380 |
| 16906509 | TMEM194B | transmembrane protein 194B | 0,19 | 0,0464 | 0,24 | 0,0106 |
| 17012404 | RNF146 | ring finger protein 146 | 0,19 | 0,0404 | 0,2 | 0,0407 |
| 16949611 | IL1RAP | interleukin 1 receptor accessory protein | 0,19 | 0,0499 | 0,2 | 0,0292 |
| 16773046 | SAP18 | Sin3A-associated protein, 18kDa | 0,18 | 0,0346 | 0,14 | 0,0373 |
| 16753712 | CAND1 | cullin-associated and neddylation-dissociated 1 | 0,18 | 0,0174 | 0,26 | 0,0068 |
| 17005922 | ZNF193 | zinc finger protein 193 | 0,18 | 0,0276 | 0,09 | 0,0200 |
| 17066036 | VPS37A | vacuolar protein sorting 37 homolog A (S. cerevisiae) | 0,18 | 0,0413 | 0,21 | 0,0076 |
| 17063448 | LUC7L2\|LOC100129148 | LUC7-like 2 (S. cerevisiae) \| uncharacterized LOC100129148 | 0,18 | 0,0193 | 0,17 | 0,0295 |
| 16989867 | PAIP2 | poly(A) binding protein interacting protein 2 | 0,18 | 0,0000 | 0,24 | 0,0236 |
| 16889054 | CCDC150 | coiled-coil domain containing 150 | 0,18 | 0,0119 | 0,12 | 0,0015 |
| 17066381 | XPO7 | exportin 7 | 0,18 | 0,0073 | 0,22 | 0,0007 |
| 16973375 | GAK | cyclin G associated kinase | 0,18 | 0,0142 | 0,21 | 0,0193 |
| 16771507 | KDM2B | lysine (K)-specific demethylase 2B | 0,17 | 0,0400 | 0,2 | 0,0139 |
| 17076372 | WHSC1L1 | Wolf-Hirschhorn syndrome candidate 1-like 1 | 0,17 | 0,0376 | 0,26 | 0,0039 |
| 17078342 | TCEB1 | transcription elongation factor B (SIII), polypeptide 1 (15kDa, elongin C) | 0,17 | 0,0178 | 0,18 | 0,0385 |
| 16808322 | PPIP5K1 | diphosphoinositol pentakisphosphate kinase 1 | 0,17 | 0,0112 | 0,27 | 0,0316 |
| 16804294 | PDE8A | phosphodiesterase 8A | 0,17 | 0,0037 | 0,19 | 0,0183 |
| 16920454 | ZNF217 | zinc finger protein 217 | 0,17 | 0,0479 | 0,31 | 0,0075 |
| 16729611 | ANKRD42 | ankyrin repeat domain 42 | 0,17 | 0,0096 | 0,16 | 0,0111 |
| 16919138 | TTI1 | TELO2 interacting protein 1 | 0,17 | 0,0230 | 0,2 | 0,0195 |
| 16943284 | TBC1D23 | TBC1 domain family, member 23 | 0,17 | 0,0409 | 0,16 | 0,0414 |
| 16837451 | C17orf80 | chromosome 17 open reading frame 80 | 0,16 | 0,0205 | 0,2 | 0,0065 |
| 16878800 | BIRC6 | baculoviral IAP repeat containing 6 | 0,16 | 0,0094 | 0,2 | 0,0200 |
| 16966008 | KLF3 | Kruppel-like factor 3 (basic) | 0,16 | 0,0144 | 0,19 | 0,0034 |
| 17064724 | PAXIP1 | PAX interacting (with transcription-activation domain) protein 1 | 0,16 | 0,0252 | 0,24 | 0,0447 |
| 16916261 | PCMTD2 | protein-L-isoaspartate (D-aspartate) O-methyltransferase domain containing 2 | 0,15 | 0,0376 | 0,21 | 0,0247 |
| 16861012 | FFAR3 | free fatty acid receptor 3 | 0,15 | 0,0087 | 0,39 | 0,0281 |
| 16975928 | USP46 | ubiquitin specific peptidase 46 | 0,15 | 0,0494 | 0,2 | 0,0156 |
| 16841982 | TOM1L2 | target of myb1-like 2 (chicken) | 0,15 | 0,0228 | 0,14 | 0,0437 |
| 16799084 | PGBD4 | piggyBac transposable element derived 4 | 0,15 | 0,0144 | 0,07 | 0,0370 |
| 16977970 | GPRIN3 | GPRIN family member 3 | 0,15 | 0,0325 | 0,3 | 0,0244 |
| 16873632 | SLC1A5 | solute carrier family 1 (neutral amino acid transporter), member 5 | 0,15 | 0,0075 | 0,21 | 0,0092 |
| 16808094 | LCMT2 | leucine carboxyl methyltransferase 2 | 0,15 | 0,0289 | 0,19 | 0,0035 |
| 16880277 | CCDC85A | coiled-coil domain containing 85A | 0,15 | 0,0459 | 0,09 | 0,0484 |
| 16994002 | LPCAT1 | lysophosphatidylcholine acyltransferase 1 | 0,14 | 0,0489 | 0,18 | 0,0154 |
| 16759977 | DCP1B | DCP1 decapping enzyme homolog B (S. cerevisiae) | 0,14 | 0,0043 | 0,12 | 0,0499 |
| 16697686 | ZNF281 | zinc finger protein 281 | 0,14 | 0,0294 | 0,26 | 0,0375 |
| 16855684 | KDSR | 3-ketodihydrosphingosine reductase | 0,14 | 0,0108 | 0,28 | 0,0261 |
| 16669121 | ATP1A1 | ATPase, Na+/K+ transporting, alpha 1 polypeptide | 0,14 | 0,0292 | 0,19 | 0,0261 |
| 16828357 | FA2H | fatty acid 2-hydroxylase | 0,14 | 0,0214 | 0,18 | 0,0389 |
| 16827353 | ZDHHC1 | zinc finger, DHHC-type containing 1 | 0,13 | 0,0246 | 0,15 | 0,0171 |
| 16731084 | SIK2 | salt-inducible kinase 2 | 0,13 | 0,0300 | 0,21 | 0,0302 |
| 16676150 | BTG2 | BTG family, member 2 | 0,13 | 0,0308 | 0,09 | 0,0187 |
| 16915220 | RAB22A |  | 0,13 | 0,0119 | 0,3 | 0,0336 |
| 16670141 | GPR89B\|GPR89A\|GPR89C | G protein-coupled receptor 89B \| G protein-coupled receptor 89A \| G protein-coupled receptor 89C | 0,13 | 0,0260 | 0,24 | 0,0009 |
| 16906921 | SF3B1 | splicing factor 3b, subunit 1, 155kDa | 0,13 | 0,0004 | 0,18 | 0,0374 |
| 16825923 | ZNF668 | zinc finger protein 668 | 0,12 | 0,0255 | 0,11 | 0,0207 |
| 16855318 | MEX3C | mex-3 homolog C (C. elegans) | 0,12 | 0,0106 | 0,2 | 0,0291 |
| 16696548 | RC3H1 | ring finger and CCCH-type domains 1 | 0,12 | 0,0065 | 0,18 | 0,0352 |
| 17074490 | PPP1R3B | protein phosphatase 1, regulatory subunit 3B | 0,12 | 0,0180 | 0,19 | 0,0183 |
| 16824400 | PKD1P1\|NPIP\|LOC399491\|LOC100288332\|LOC642799\|LOC642778\|LOC100506193\|NPIPP1 | polycystic kidney disease 1 (autosomal dominant) pseudogene 1 \| nuclear pore complex interacting protein \| GPS, PLAT and transmembrane domain-containing protein \| nuclear pore complex-interacting protein-like 1-like \| nuclear pore complex interacting protein pseudogene 1 | 0,11 | 0,0147 | 0,17 | 0,0340 |
| 16726336 | PLCB3 | phospholipase C, beta 3 (phosphatidylinositol-specific) | 0,11 | 0,0182 | 0,07 | 0,0005 |
| 16971048 | SMARCA5 | SWI/SNF related, matrix associated, actin dependent regulator of chromatin, subfamily a, member 5 | 0,11 | 0,0206 | 0,18 | 0,0316 |
| 16958812 | RUVBL1 | RuvB-like 1 (E. coli) | 0,11 | 0,0247 | 0,2 | 0,0134 |
| 16776658 | ATP11A | ATPase, class VI, type 11A | 0,10 | 0,0001 | 0,24 | 0,0218 |
| 16899079 | DGUOK-AS1 | DGUOK antisense RNA 1 (non-protein coding) | 0,10 | 0,0342 | 0,09 | 0,0109 |
| 16760603 | MLF2 | myeloid leukemia factor 2 | 0,10 | 0,0087 | 0,14 | 0,0006 |
| 16801035 | AP4E1 | adaptor-related protein complex 4, epsilon 1 subunit | 0,09 | 0,0483 | 0,28 | 0,0298 |
| 16847117 | MTMR4 | myotubularin related protein 4 | 0,09 | 0,0103 | 0,09 | 0,0198 |
| 16924007 | DIP2A | DIP2 disco-interacting protein 2 homolog A (Drosophila) | 0,09 | 0,0388 | 0,18 | 0,0064 |
| 16890490 | ATIC | 5-aminoimidazole-4-carboxamide ribonucleotide formyltransferase/IMP cyclohydrolase | 0,08 | 0,0145 | 0,12 | 0,0042 |
| 16676130 | ADORA1 | adenosine A1 receptor | 0,08 | 0,0073 | 0,14 | 0,0143 |
| 16692787 | ENSA | endosulfine alpha | 0,07 | 0,0491 | 0,15 | 0,0255 |
| 16964050 | WHSC1 | Wolf-Hirschhorn syndrome candidate 1 | 0,07 | 0,0285 | 0,2 | 0,0247 |
| 16824429 | NOMO2\|NOMO1\|NOMO3 | NODAL modulator 2 \| NODAL modulator 1 \| NODAL modulator 3 | 0,07 | 0,0358 | 0,09 | 0,0401 |
| 16907488 | RAPH1 | Ras association (RalGDS/AF-6) and pleckstrin homology domains 1 | 0,06 | 0,0497 | 0,31 | 0,0019 |
| 16849194 | PRPSAP1 | phosphoribosyl pyrophosphate synthetase-associated protein 1 | 0,06 | 0,0323 | 0,12 | 0,0205 |
| 16756310 | TCP11L2 | t-complex 11 (mouse)-like 2 | 0,06 | 0,0261 | 0,19 | 0,0036 |
| 17065109 | ERICH1-AS1 | ERICH1 antisense RNA 1 (non-protein coding) | 0,06 | 0,0314 | 0,06 | 0,0146 |
| 16713187 | NRP1 | neuropilin 1 | 0,06 | 0,0057 | 0,27 | 0,0249 |
| 16802605 | LRRC49 | leucine rich repeat containing 49 | 0,05 | 0,0374 | 0,21 | 0,0120 |
| 16677789 | BROX | BRO1 domain and CAAX motif containing | 0,04 | 0,0383 | 0,08 | 0,0321 |
| 16820486 | CDH1 | cadherin 1, type 1, E-cadherin (epithelial) | 0,03 | 0,0380 | 0,09 | 0,0078 |

**Table B. Pathways upregulated in response to both S6K1 and S6K2 siRNA.**

| p-value | Term | Term ID | Term description | Genes |
| --- | --- | --- | --- | --- |
| 2.69e-03 | GO:0044260 | BP | cellular macromolecule metabolic process | CDH1, NRP1, WHSC1, ADORA1, MTMR4, RUVBL1, SMARCA5, PPP1R3B, RC3H1, SF3B1, BTG2, SIK2, ZDHHC1, ZNF281, DCP1B, LCMT2, USP46, PAXIP1, KLF3, BIRC6, ANKRD42, ZNF217, PDE8A, TCEB1, WHSC1L1, KDM2B, PAIP2, VPS37A, CAND1, SAP18, RNF146, ATP6AP2, EIF3G, TBK1, NEO1, RBBP6, RERE, SIVA1, LONP2, ORC5, CUL1, ZNF326, MED23, SYNCRIP, MED8, ELF1, NFS1, UCHL5, RRN3, CENPP, CRNKL1, PHF17, RPTOR, ST6GALNAC6, SDF2, MAD2L1, USP40, USP48, MRPL11, TBRG1, ITGA6, SAFB, CDC7, PPIL4, JMJD1C, BTRC, CASP8AP2, CDC73, GALNT7, RBBP5, ZNF295, OSGEPL1, MKKS, PRIM1, STAT1, TOP2B, MSRB1, SAR1B, TLN1, SON, IL6R, SBNO1, RAD50, QKI, CD44, RAD51C, MTX2, CASK, ZBTB8A, NR3C1, SDHAF2, ABCA1, ZNF562 |
| 1.10e-02 | GO:0031981 | CC | nuclear lumen | WHSC1, ENSA, RUVBL1, SMARCA5, SF3B1, PAXIP1, ZNF217, TCEB1, KDM2B, SAP18, RBBP6, RERE, SIVA1, ORC5, CUL1, ZNF326, MED13L, MED23, SYNCRIP, MED8, PWP2, UCHL5, RRN3, CENPP, CRNKL1, PHF17, CDC7, JMJD1C, CASP8AP2, CDC73, RBBP5, ZCCHC7, PRIM1, STAT1, TOP2B, SON, RAD50, RAD51C, CASK, NR3C1, SDHAF2 |
| 6.39e-03 | BIOGRID:00000 | bi | BioGRID interaction data | CDH1, BROX, LRRC49, NRP1, TCP11L2, PRPSAP1, RAPH1, WHSC1, ENSA, ADORA1, ATIC, DIP2A, MTMR4, AP4E1, MLF2, ATP11A, RUVBL1, SMARCA5, PLCB3, PPP1R3B, RC3H1, MEX3C, SF3B1, RAB22A, BTG2, SIK2, ZDHHC1, FA2H, KDSR, ZNF281, DCP1B, LPCAT1, CCDC85A, LCMT2, SLC1A5, TOM1L2, USP46, PCMTD2, PAXIP1, KLF3, BIRC6, TBC1D23, TTI1, ANKRD42, ZNF217, PDE8A, PPIP5K1, TCEB1, WHSC1L1, KDM2B |

**Table C. Genes downregulated in response to both S6K1 and S6K2 siRNA.**

| Transcripts cluster ID | Gene symbol | Gene description | S6K1 siRNA  Fold change | S6K1 siRNA  p-value^1^ | S6K2 siRNA  Fold change | S6K2 siRNA  p-value^1^ |  |  |
| --- | --- | --- | --- | --- | --- | --- | --- | --- |
| 17048563 | PEG10 | paternally expressed 10 | -0,63 | 0,0133 | -0,33 | 0,0318 |  |  |
| 17101292 | STS | steroid sulfatase (microsomal), isozyme S | -0,53 | 0,0471 | -0,34 | 0,0100 |  |  |
| 16859205 | UCA1 | urothelial cancer associated 1 (non-protein coding) | -0,50 | 0,0256 | -0,44 | 0,0119 |  |  |
| 16860123 | ZNF486 | zinc finger protein 486 | -0,48 | 0,0138 | -0,32 | 0,0417 |  |  |
| 16920475 | BCAS1 | breast carcinoma amplified sequence 1 | -0,44 | 0,0204 | -0,16 | 0,0497 |  |  |
| 16954761 | GLYCTK-AS1 | GLYCTK antisense RNA 1 (non-protein coding) | -0,42 | 0,0112 | -0,45 | 0,0113 |  |  |
| 16701023 | CHRM3-AS1 | CHRM3 antisense RNA 1 (non-protein coding) | -0,39 | 0,0359 | -0,37 | 0,0117 |  |  |
| 16762655 | KLHDC5 | kelch domain containing 5 | -0,37 | 0,0218 | -0,37 | 0,0190 |  |  |
| 16738333 | TRIM51HP | tripartite motif-containing 51H, pseudogene | -0,36 | 0,0068 | -0,35 | 0,0394 |  |  |
| 16761820 | MGP | matrix Gla protein | -0,36 | 0,0076 | -0,28 | 0,0330 |  |  |
| 16918294 | TSPY26P | testis specific protein, Y-linked 26, pseudogene | -0,33 | 0,0381 | -0,12 | 0,0453 |  |  |
| 16829718 | OR1A2 | olfactory receptor, family 1, subfamily A, member 2 | -0,32 | 0,0082 | -0,28 | 0,0028 |  |  |
| 17029847 | HLA-DPA1 | major histocompatibility complex, class II, DP alpha 1 | -0,28 | 0,0471 | -0,42 | 0,0344 |  |  |
| 17116583 | TTTY4\| TTTY4B\| TTTY4C | testis-specific transcript, Y-linked 4 (non-protein coding) \| testis-specific transcript, Y-linked 4B (non-protein coding) \| testis-specific transcript, Y-linked 4C (non-protein coding) | -0,27 | 0,0436 | -0,19 | 0,0386 |  |  |
| 16681468 | C1orf200 | chromosome 1 open reading frame 200 | -0,27 | 0,0447 | -0,25 | 0,0392 |  |  |
| 16754717 | MYF5 | myogenic factor 5 | -0,27 | 0,0039 | -0,31 | 0,0360 |  |  |
| 17104688 | ITGB1BP2 | integrin beta 1 binding protein (melusin) 2 | -0,26 | 0,0038 | -0,19 | 0,0438 |  |  |
| 16916396 | TMEM74B | transmembrane protein 74B | -0,25 | 0,0213 | -0,26 | 0,0491 |  |  |
| 16960771 | KCNAB1-AS1 | KCNAB1 antisense RNA 1 (non-protein coding) | -0,25 | 0,0198 | -0,09 | 0,0406 |  |  |
| 17006649 | LTA | lymphotoxin alpha (TNF superfamily, member 1) | -0,23 | 0,0295 | -0,26 | 0,0162 |  |  |
| 16703563 | BAMBI | BMP and activin membrane-bound inhibitor homolog (Xenopus laevis) | -0,23 | 0,0154 | -0,17 | 0,0008 |  |  |
| 17033327 | LTA | lymphotoxin alpha (TNF superfamily, member 1) | -0,23 | 0,0352 | -0,27 | 0,0172 |  |  |
| 16815918 | TNFRSF17 | tumor necrosis factor receptor superfamily, member 17 | -0,22 | 0,0245 | -0,15 | 0,0220 |  |  |
| 17043355 | AIMP2 | aminoacyl tRNA synthetase complex-interacting multifunctional protein 2 | -0,22 | 0,0267 | -0,12 | 0,0158 |  |  |
| 16960844 | VEPH1 | ventricular zone expressed PH domain homolog 1 (zebrafish) | -0,22 | 0,0457 | -0,15 | 0,0346 |  |  |
| 16841295 | MYH1 | myosin, heavy chain 1, skeletal muscle, adult | -0,21 | 0,0257 | -0,22 | 0,0460 |  |  |
| 17075712 | CHRNA2 | cholinergic receptor, nicotinic, alpha 2 (neuronal) | -0,21 | 0,0438 | -0,26 | 0,0358 |  |  |
| 16800642 | HMGN2P46 | high mobility group nucleosomal binding domain 2 pseudogene 46 | -0,20 | 0,0291 | -0,21 | 0,0406 |  |  |
| 16829880 | GLTPD2 | glycolipid transfer protein domain containing 2 | -0,20 | 0,0206 | -0,21 | 0,0301 |  |  |
| 16881021 | GKN1 | gastrokine 1 | -0,19 | 0,0035 | -0,26 | 0,0194 |  |  |
| 17035408 | LTA | lymphotoxin alpha (TNF superfamily, member 1) | -0,19 | 0,0353 | -0,21 | 0,0121 |  |  |
| 16775626 | POU4F1-AS1 | POU4F1 antisense RNA 1 (non-protein coding) | -0,19 | 0,0117 | -0,17 | 0,0366 |  |  |
| 16946835 | WWTR1-AS1 | WWTR1 antisense RNA 1 (non-protein coding) | -0,19 | 0,0255 | -0,32 | 0,0101 |  |  |
| 16790138 | METTL17 | methyltransferase like 17 | -0,19 | 0,0161 | -0,25 | 0,0180 |  |  |
| 16923675 | KRTAP10-12 | keratin associated protein 10-12 | -0,19 | 0,0364 | -0,21 | 0,0175 |  |  |
| 16874878 | SIGLEC12 | sialic acid binding Ig-like lectin 12 (gene/pseudogene) | -0,18 | 0,0225 | -0,22 | 0,0186 |  |  |
| 17070713 | NECAB1 | N-terminal EF-hand calcium binding protein 1 | -0,18 | 0,0066 | -0,35 | 0,0408 |  |  |
| 17118019 | ATP1B3\| LOC100507375 | ATPase, Na+/K+ transporting, beta 3 polypeptide \| uncharacterized LOC100507375 | -0,18 | 0,0208 | -0,36 | 0,0004 |  |  |
| 17035225 | MDC1\| MDC1-AS1 | mediator of DNA-damage checkpoint 1 \| MDC1 antisense RNA 1 (non-protein coding) | -0,17 | 0,0426 | -0,24 | 0,0455 |  |  |
| 17113677 | RHOXF2B | Rhox homeobox family, member 2B | -0,17 | 0,0092 | -0,18 | 0,0314 |  |  |
| 16887463 | SP5 | Sp5 transcription factor | -0,17 | 0,0352 | -0,17 | 0,0138 |  |  |
| 16751420 | GRASP | GRP1 (general receptor for phosphoinositides 1)-associated scaffold protein | -0,17 | 0,0283 | -0,14 | 0,0162 |  |  |
| 17026419 | PSMB9 | proteasome (prosome, macropain) subunit, beta type, 9 (large multifunctional peptidase 2) | -0,17 | 0,0049 | -0,18 | 0,0257 |  |  |
| 16957807 | POPDC2\|COX17 | popeye domain containing 2 \| COX17 cytochrome c oxidase assembly homolog (S. cerevisiae) | -0,17 | 0,0365 | -0,16 | 0,0250 |  |  |
| 16835013 | SPPL2C | signal peptide peptidase like 2C | -0,15 | 0,0043 | -0,16 | 0,0307 |  |  |
| 16935994 | SMC1B | structural maintenance of chromosomes 1B | -0,15 | 0,0440 | -0,17 | 0,0378 |  |  |
| 16766283 | PTGES3\| LOC100506732\| PTGES3P3 | prostaglandin E synthase 3 (cytosolic) \| uncharacterized LOC100506732 \| prostaglandin E synthase 3 (cytosolic) pseudogene 3 | -0,14 | 0,0432 | -0,07 | 0,0329 |  |  |
| 17000180 | IL9 | interleukin 9 | -0,13 | 0,0312 | -0,09 | 0,0257 |  |  |
| 16724190 | CRY2 | cryptochrome 2 (photolyase-like) | -0,13 | 0,0034 | -0,12 | 0,0031 |  |  |
| 16904514 | SCN3A | sodium channel, voltage-gated, type III, alpha subunit | -0,12 | 0,0272 | -0,11 | 0,0428 |  |  |
| 16916462 | SIRPB2 | signal-regulatory protein beta 2 | -0,12 | 0,0155 | -0,14 | 0,0275 |  |  |
| 16857766 | CLEC4GP1 | C-type lectin domain family 4, member G pseudogene 1 | -0,10 | 0,0169 | -0,1 | 0,0339 |  |  |
| 16800117 | STARD9 | StAR-related lipid transfer (START) domain containing 9 | -0,10 | 0,0208 | -0,11 | 0,0442 |  |  |
| 16954068 | AMT | aminomethyltransferase | -0,09 | 0,0252 | -0,04 | 0,0051 |  |  |
| 17054312 | C7orf50 | chromosome 7 open reading frame 50 | -0,09 | 0,0295 | -0,09 | 0,0268 |  |  |
| 16939755 | ZNF662\| KRBOX1 | zinc finger protein 662 \| KRAB box domain containing 1 | -0,09 | 0,0309 | -0,14 | 0,0311 |  |  |
| 16981219 | DDX60 | DEAD (Asp-Glu-Ala-Asp) box polypeptide 60 | -0,08 | 0,0384 | -0,17 | 0,0208 |  |  |
| 16752168 | OR6C65 | olfactory receptor, family 6, subfamily C, member 65 | -0,08 | 0,0075 | -0,17 | 0,0256 |  |  |
| 17020799 | MTO1 | mitochondrial translation optimization 1 homolog (S. cerevisiae) | -0,06 | 0,0005 | -0,05 | 0,0227 |  |  |
| 16881353 | DYSF | dysferlin, limb girdle muscular dystrophy 2B (autosomal recessive) | -0,05 | 0,0237 | -0,04 | 0,0421 |  |  |

^1^From Student’s t-test.
