# Supplementary material for: Revealing Different Roles of the mTOR-Targets S6K1 and S6K2 in Breast Cancer by Expression Profiling and Structural Analysis
Source: PLoS One. 2015 Dec 23;10(12):e0145013. doi: 10.1371/journal.pone.0145013 (PMC4689523; doi:10.1371/journal.pone.0145013)
Supplement: S9 Table — Genes upregulated in response to double S6K1/S6K2 siRNA, but not to single siRNA (Table A). Pathways upregulated in response to S6K1/S6K2 siRNA, but not to single siRNA (Table B). Genes downregulated in response to double S6K1/S6K2 siRNA, but not to single siRNA (Table C). Pathways downregulated in response to double S6K1/S6K2 siRNA, but not to single siRNA (Table D). (DOCX) [file pone.0145013.s013.docx]

**Table A. Genes upregulated in response to double S6K1/S6K2 siRNA, but not to single siRNA.**

| Transcripts cluster ID | Gene description | Gene symbol | S6K1 siRNA  and  S6K2 siRNA  Fold change | S6K1 siRNA  and  S6K2 siRNA  p-value^1^ | S6K1 siRNA  Fold change | S6K1 siRNA  p-value^1^ | S6K2 siRNA  Fold change | S6K2 siRNA  p-value^1^ |
| --- | --- | --- | --- | --- | --- | --- | --- | --- |
| 16734163 | chitinase domain containing 1 | CHID1 | 0,02 | 0,0256 | 0,04 | 0,7027 | 0,03 | 0,3974 |
| 17093570 | valosin containing protein | VCP | 0,03 | 0,0348 | 0,03 | 0,5045 | 0,06 | 0,5465 |
| 16866867 | MOB kinase activator 3A | MOB3A | 0,03 | 0,0197 | -0,10 | 0,1893 | -0,03 | 0,6996 |
| 16935290 | megakaryoblastic leukemia (translocation) 1 | MKL1 | 0,03 | 0,0096 | -0,03 | 0,8054 | 0 | 0,9662 |
| 17101732 | carbonic anhydrase VB pseudogene 1 | CA5BP1 | 0,04 | 0,0116 | 0,07 | 0,0718 | 0,18 | 0,0682 |
| 16818461 | HECT and RLD domain containing E3 ubiquitin protein ligase 2 pseudogene \| hect domain and RLD 2 pseudogene 4 \| uncharacterized LOC100653257 | LOC100289574\| HERC2P4\| LOC100653257\| LOC100652752 | 0,04 | 0,0424 | -0,05 | 0,7017 | 0,01 | 0,9505 |
| 16818374 | solute carrier family 5 (sodium/glucose cotransporter), member 2 | SLC5A2 | 0,04 | 0,0103 | -0,07 | 0,6492 | 0,19 | 0,1838 |
| 17009032 | polymerase (RNA) I polypeptide C, 30kDa | POLR1C | 0,04 | 0,0059 | 0,08 | 0,4172 | 0,01 | 0,9423 |
| 17057035 | proteasome (prosome, macropain) subunit, alpha type, 2 | PSMA2 | 0,04 | 0,0233 | 0,03 | 0,7309 | 0,07 | 0,1679 |
| 17094998 | chromosome 9 open reading frame 40 | C9orf40 | 0,05 | 0,0434 | -0,13 | 0,2052 | -0,02 | 0,8217 |
| 16922327 | intersectin 1 (SH3 domain protein) | ITSN1 | 0,05 | 0,0072 | -0,04 | 0,3238 | 0,09 | 0,1081 |
| 16738615 | olfactory receptor, family 5, subfamily B, member 2 | OR5B2 | 0,05 | 0,0297 | 0,06 | 0,4718 | -0,04 | 0,7634 |
| 16753202 | Ts translation elongation factor, mitochondrial | TSFM | 0,05 | 0,0437 | 0,05 | 0,0772 | 0,06 | 0,2337 |
| 16671217 | integrator complex subunit 3 | INTS3 | 0,05 | 0,0497 | -0,05 | 0,6483 | 0,07 | 0,3572 |
| 16866974 | guanine nucleotide binding protein (G protein), gamma 7 | GNG7 | 0,05 | 0,0068 | 0,03 | 0,8257 | -0,21 | 0,1656 |
| 17048724 | basic helix-loop-helix family, member a15 | BHLHA15 | 0,05 | 0,0395 | -0,02 | 0,3317 | 0,12 | 0,3178 |
| 16761631 | dual specificity phosphatase 16 | DUSP16 | 0,05 | 0,0145 | -0,19 | 0,1460 | 0,02 | 0,8342 |
| 16849098 | exocyst complex component 7 | EXOC7 | 0,05 | 0,0395 | -0,12 | 0,4488 | -0,06 | 0,6437 |
| 16767911 | PRKC, apoptosis, WT1, regulator | PAWR | 0,05 | 0,0115 | -0,04 | 0,7643 | 0,12 | 0,0720 |
| 16812685 | homer homolog 2 (Drosophila) | HOMER2 | 0,06 | 0,0228 | -0,05 | 0,5902 | 0,01 | 0,9549 |
| 16844099 | calcium channel, voltage-dependent, beta 1 subunit | CACNB1 | 0,06 | 0,0191 | -0,07 | 0,0993 | 0,16 | 0,1115 |
| 17094766 | transient receptor potential cation channel, subfamily M, member 3 | TRPM3 | 0,06 | 0,0447 | 0,11 | 0,1495 | -0,02 | 0,8189 |
| 16839294 | active BCR-related | ABR | 0,06 | 0,0258 | -0,12 | 0,3239 | -0,05 | 0,6647 |
| 16874486 | zinc finger protein 420-like | LOC100287477 | 0,06 | 0,0043 | 0,13 | 0,5539 | -0,04 | 0,6310 |
| 16813112 | Rh family, C glycoprotein | RHCG | 0,06 | 0,0141 | 0,00 | 0,9856 | -0,01 | 0,9295 |
| 16869034 | heterogeneous nuclear ribonucleoprotein A1 pseudogene 10 | HNRNPA1P10 | 0,06 | 0,0012 | -0,05 | 0,5239 | 0,1 | 0,4018 |
| 16707927 | ribosomal protein L13a pseudogene 5 \| small nucleolar RNA, C/D box 32A \| small nucleolar RNA, C/D box 33 \| small nucleolar RNA, C/D box 34 \| small nucleolar RNA, C/D box 35A \| ribosomal protein L13a pseudogene 20 | RPL13AP5\| SNORD32A\| SNORD33\| SNORD34\| SNORD35A \|RPL13AP20 | 0,06 | 0,0471 | 0,10 | 0,1109 | 0,01 | 0,7618 |
| 16863629 | NAPA antisense RNA 1 (non-protein coding) | NAPA-AS1 | 0,06 | 0,0212 | 0,02 | 0,6919 | -0,04 | 0,3889 |
| 16960775 | signal sequence receptor, gamma (translocon-associated protein gamma) | SSR3 | 0,06 | 0,0220 | 0,03 | 0,4155 | -0,04 | 0,7263 |
| 16679517 | chromosome 1 open reading frame 101 | C1orf101 | 0,06 | 0,0283 | 0,04 | 0,4301 | 0,04 | 0,6273 |
| 16774405 | DnaJ (Hsp40) homolog, subfamily C, member 15 | DNAJC15 | 0,07 | 0,0195 | 0,05 | 0,2171 | 0,06 | 0,6129 |
| 16733278 | decapping enzyme, scavenger | DCPS | 0,07 | 0,0410 | -0,11 | 0,3008 | 0,06 | 0,7223 |
| 16926725 | chromosome 21 open reading frame 58 | C21orf58 | 0,07 | 0,0244 | 0,11 | 0,3927 | 0,05 | 0,6449 |
| 17011992 | karyopherin alpha 5 (importin alpha 6) | KPNA5 | 0,07 | 0,0006 | 0,10 | 0,1045 | 0,12 | 0,3352 |
| 16958897 | KIAA1257 \| uncharacterized LOC100132731 | KIAA1257\| LOC100132731 | 0,07 | 0,0463 | 0,05 | 0,7074 | -0,01 | 0,7364 |
| 16843456 | schlafen family member 14 | SLFN14 | 0,07 | 0,0159 | 0,02 | 0,7522 | -0,09 | 0,5068 |
| 16805594 | myocyte enhancer factor 2A | MEF2A | 0,07 | 0,0386 | -0,01 | 0,9071 | -0,04 | 0,7972 |
| 17100317 | suppressor APC domain containing 2 | SAPCD2 | 0,07 | 0,0458 | 0,08 | 0,6623 | 0,08 | 0,0924 |
| 16684136 | EYA3 intronic transcript 1 (non-protein coding) | EYA3-IT1 | 0,07 | 0,0107 | 0,21 | 0,0643 | 0,09 | 0,5019 |
| 17061972 | chromosome 7 open reading frame 60 | C7orf60 | 0,07 | 0,0216 | 0,06 | 0,3535 | 0,14 | 0,1924 |
| 16696295 | kinesin-associated protein 3 | KIFAP3 | 0,07 | 0,0137 | 0,08 | 0,1032 | 0,02 | 0,1566 |
| 16664156 | microtubule associated serine/threonine kinase 2 | MAST2 | 0,07 | 0,0083 | -0,01 | 0,9397 | 0,04 | 0,0603 |
| 17043822 | basic leucine zipper and W2 domains 2 | BZW2 | 0,07 | 0,0341 | 0,06 | 0,1884 | 0,02 | 0,6280 |
| 16671540 | lens epithelial protein | LENEP | 0,07 | 0,0358 | 0,15 | 0,2034 | 0,03 | 0,4749 |
| 16857842 | chemokine (C-C motif) ligand 25 | CCL25 | 0,07 | 0,0381 | 0,05 | 0,6786 | 0,04 | 0,2752 |
| 16969591 | hydroxyacyl-CoA dehydrogenase | HADH | 0,07 | 0,0232 | 0,10 | 0,1460 | 0,02 | 0,6814 |
| 17038967 | tripartite motif containing 31 | TRIM31 | 0,07 | 0,0025 | 0,24 | 0,2186 | 0,12 | 0,1877 |
| 17057579 |  | HUS1 | 0,07 | 0,0437 | -0,07 | 0,3736 | 0,07 | 0,4475 |
| 16733045 | etoposide induced 2.4 mRNA | EI24 | 0,08 | 0,0133 | 0,07 | 0,1394 | -0,04 | 0,4025 |
| 16792486 | ribosomal protein S29 | RPS29 | 0,08 | 0,0418 | 0,13 | 0,3111 | 0,01 | 0,4239 |
| 17039202 | surfactant associated 2 | SFTA2 | 0,08 | 0,0496 | -0,03 | 0,4658 | 0,03 | 0,6771 |
| 16699112 |  | NSL1 | 0,08 | 0,0149 | -0,03 | 0,7111 | 0 | 0,9883 |
| 16944386 | translocase of inner mitochondrial membrane domain containing 1 | TIMMDC1 | 0,08 | 0,0324 | 0,03 | 0,6073 | 0,07 | 0,5061 |
| 17077866 | transcription factor 24 | TCF24 | 0,08 | 0,0431 | 0,03 | 0,6218 | -0,01 | 0,8843 |
| 17079744 | zinc finger protein 706 | ZNF706 | 0,08 | 0,0239 | 0,15 | 0,0720 | 0,09 | 0,3089 |
| 17010175 | opioid growth factor receptor-like 1 | OGFRL1 | 0,08 | 0,0024 | -0,03 | 0,7936 | -0,06 | 0,1223 |
| 17079711 | tyrosine 3-monooxygenase/tryptophan 5-monooxygenase activation protein, zeta polypeptide \| tyrosine 3-monooxygenase/tryptophan 5-monooxygenase activation protein, zeta pseudogene 3 \| tyrosine 3-monooxygenase/tryptophan 5-monooxygenase activation protein, zeta pseudogene 2 | YWHAZ\| YWHAZP3\| YWHAZP2 | 0,08 | 0,0046 | -0,02 | 0,8113 | 0,03 | 0,1898 |
| 16847352 | adaptor-related protein complex 1, sigma 2 subunit pseudogene | LOC653653 | 0,08 | 0,0003 | 0,08 | 0,5467 | 0,04 | 0,7821 |
| 16930144 | apolipoprotein B mRNA editing enzyme, catalytic polypeptide-like 3A \| APOBEC3A and APOBEC3B deletion hybrid \| apolipoprotein B mRNA editing enzyme, catalytic polypeptide-like 3B | APOBEC3A\| APOBEC3A_B\| APOBEC3B | 0,08 | 0,0044 | -0,06 | 0,6711 | 0,04 | 0,7979 |
| 16730308 | endonuclease domain containing 1 | ENDOD1 | 0,08 | 0,0305 | 0,04 | 0,6921 | -0,11 | 0,5170 |
| 16709268 | acyl-CoA synthetase long-chain family member 5 | ACSL5 | 0,08 | 0,0249 | 0,09 | 0,4492 | 0,18 | 0,3900 |
| 16939558 | catenin (cadherin-associated protein), beta 1, 88kDa | CTNNB1 | 0,08 | 0,0042 | 0,00 | 0,9833 | 0,12 | 0,0557 |
| 17046228 | glioblastoma amplified sequence | GBAS | 0,08 | 0,0122 | -0,02 | 0,8419 | 0,16 | 0,1494 |
| 16664912 | leucine rich repeat containing 42 | LRRC42 | 0,09 | 0,0455 | 0,17 | 0,3198 | 0,24 | 0,1063 |
| 16987986 | EPB41L4A antisense RNA 1 (non-protein coding) | EPB41L4A-AS1 | 0,09 | 0,0080 | -0,04 | 0,7576 | 0,01 | 0,9352 |
| 17049939 | peptidase (mitochondrial processing) beta | PMPCB | 0,09 | 0,0481 | 0,02 | 0,6849 | 0,08 | 0,0585 |
| 16931068 | parvin, gamma | PARVG | 0,09 | 0,0004 | -0,01 | 0,8181 | 0,06 | 0,4687 |
| 16884594 | interleukin 36, gamma | IL36G | 0,09 | 0,0462 | 0,26 | 0,0718 | 0,24 | 0,3700 |
| 16721773 | importin 7 | IPO7 | 0,09 | 0,0374 | -0,04 | 0,6155 | 0,1 | 0,2108 |
| 16936095 | uncharacterized LOC150384 | CN5H6.4 | 0,09 | 0,0418 | -0,11 | 0,6023 | 0,03 | 0,7513 |
| 17117817 | putative uncharacterized protein FLJ38264-like | LOC100652943 | 0,09 | 0,0322 | -0,19 | 0,1261 | -0,05 | 0,5653 |
| 16850616 | myosin, light chain 12B, regulatory | MYL12B | 0,09 | 0,0016 | 0,15 | 0,0687 | 0,09 | 0,3829 |
| 16898326 | pellino E3 ubiquitin protein ligase 1 | PELI1 | 0,09 | 0,0123 | 0,13 | 0,3048 | 0,15 | 0,3790 |
| 16967801 | methylenetetrahydrofolate dehydrogenase (NADP+ dependent) 2-like | MTHFD2L | 0,09 | 0,0013 | 0,05 | 0,8092 | 0,04 | 0,6832 |
| 17075883 | integrator complex subunit 9 | INTS9 | 0,09 | 0,0377 | 0,01 | 0,8568 | 0,05 | 0,6671 |
| 16771216 | ribosomal protein, large, P0 | RPLP0 | 0,09 | 0,0140 | 0,02 | 0,9072 | 0,05 | 0,6712 |
| 16976868 | betacellulin | BTC | 0,09 | 0,0496 | 0,20 | 0,1260 | 0,23 | 0,1725 |
| 17096715 | olfactory receptor, family 13, subfamily C, member 3 | OR13C3 | 0,09 | 0,0228 | 0,30 | 0,1414 | 0,09 | 0,4032 |
| 17067537 | leptin receptor overlapping transcript-like 1 | LEPROTL1 | 0,09 | 0,0141 | 0,02 | 0,8399 | 0,06 | 0,3848 |
| 16955511 | pyruvate dehydrogenase (lipoamide) beta | PDHB | 0,09 | 0,0407 | 0,13 | 0,2394 | 0,17 | 0,0677 |
| 16777930 | FRY antisense RNA 1 (non-protein coding) | FRY-AS1 | 0,09 | 0,0400 | 0,05 | 0,2830 | 0,07 | 0,2275 |
| 16959871 | transcription factor Dp-2 (E2F dimerization partner 2) | TFDP2 | 0,09 | 0,0457 | -0,08 | 0,5624 | 0,13 | 0,3061 |
| 16924979 |  | URB1 | 0,09 | 0,0055 | 0,08 | 0,4684 | 0,06 | 0,7579 |
| 16834749 | RUN domain containing 3A | RUNDC3A | 0,10 | 0,0218 | -0,02 | 0,8604 | 0,11 | 0,3571 |
| 17001258 | protein phosphatase 2, regulatory subunit B, beta | PPP2R2B | 0,10 | 0,0311 | 0,20 | 0,4237 | 0,18 | 0,3974 |
| 16894894 | lysosomal protein transmembrane 4 alpha | LAPTM4A | 0,10 | 0,0398 | -0,01 | 0,8803 | 0,08 | 0,0636 |
| 17065173 | Rho guanine nucleotide exchange factor (GEF) 10 | ARHGEF10 | 0,10 | 0,0156 | -0,07 | 0,2145 | 0,06 | 0,5023 |
| 16703340 | myosin IIIA | MYO3A | 0,10 | 0,0417 | 0,16 | 0,2414 | 0,06 | 0,2391 |
| 16668150 | seryl-tRNA synthetase | SARS | 0,10 | 0,0004 | 0,06 | 0,5645 | 0,03 | 0,7617 |
| 17057946 | phosphoserine phosphatase | PSPH | 0,10 | 0,0442 | 0,01 | 0,9464 | 0,16 | 0,1969 |
| 16845246 | high mobility group nucleosomal binding domain 2 pseudogene 42 | HMGN2P42 | 0,10 | 0,0241 | 0,12 | 0,1479 | 0,01 | 0,9240 |
| 17110527 | zinc finger protein 630 | ZNF630 | 0,10 | 0,0250 | 0,12 | 0,5324 | 0,11 | 0,3492 |
| 16915262 | STX16-NPEPL1 readthrough (non-protein coding) \| syntaxin 16 \| aminopeptidase-like 1 \| uncharacterized LOC100652930 | STX16-NPEPL1\| LOC100652930 | 0,10 | 0,0319 | 0,03 | 0,7221 | 0,09 | 0,3127 |
| 16660828 | serine/arginine repetitive matrix 1 | SRRM1 | 0,10 | 0,0172 | -0,05 | 0,4544 | 0,07 | 0,1379 |
| 16866252 | zinc finger protein 8 | ZNF8 | 0,10 | 0,0047 | -0,01 | 0,8962 | 0,1 | 0,1247 |
| 17103451 | histone deacetylase 6 | HDAC6 | 0,10 | 0,0480 | -0,05 | 0,7060 | 0,01 | 0,9600 |
| 16740848 | coiled-coil domain containing 87 | CCDC87 | 0,10 | 0,0221 | -0,14 | 0,6541 | 0,22 | 0,1618 |
| 17098424 | mitogen-activated protein kinase associated protein 1 | MAPKAP1 | 0,10 | 0,0153 | 0,07 | 0,3263 | 0,08 | 0,5061 |
| 16959623 | alpha-1,4-N-acetylglucosaminyltransferase | A4GNT | 0,10 | 0,0073 | 0,10 | 0,0927 | -0,02 | 0,4101 |
| 17068541 | voltage-dependent anion channel 3 | VDAC3 | 0,11 | 0,0154 | 0,12 | 0,1067 | 0,04 | 0,3273 |
| 16812942 | neurotrophic tyrosine kinase, receptor, type 3 | NTRK3 | 0,11 | 0,0330 | 0,00 | 0,9698 | 0,02 | 0,8349 |
| 16834921 | hexamethylene bis-acetamide inducible 1 | HEXIM1 | 0,11 | 0,0412 | 0,04 | 0,6585 | 0,09 | 0,3515 |
| 16671125 | late cornified envelope-like proline-rich 1 | LELP1 | 0,11 | 0,0145 | 0,07 | 0,6947 | 0,05 | 0,8347 |
| 16968468 | CDP-diacylglycerol synthase (phosphatidate cytidylyltransferase) 1 | CDS1 | 0,11 | 0,0106 | -0,02 | 0,8173 | 0,1 | 0,2054 |
| 17096931 | transmembrane protein 245 | TMEM245 | 0,11 | 0,0489 | 0,07 | 0,3808 | 0,09 | 0,5844 |
| 17104943 | zinc finger, CCHC domain containing 13 | ZCCHC13 | 0,11 | 0,0027 | 0,16 | 0,1758 | 0,13 | 0,1064 |
| 16783368 |  | KIAA0391 | 0,11 | 0,0441 | 0,08 | 0,5635 | 0,14 | 0,2330 |
| 17088148 | sorting nexin family member 30 | SNX30 | 0,11 | 0,0159 | -0,01 | 0,9685 | 0,02 | 0,8363 |
| 16889209 | chromosome 2 open reading frame 47 | C2orf47 | 0,11 | 0,0437 | 0,10 | 0,2568 | 0,17 | 0,2653 |
| 17092208 | endoplasmic reticulum metallopeptidase 1 | ERMP1 | 0,11 | 0,0254 | -0,05 | 0,2433 | 0,09 | 0,3767 |
| 17092767 | myeloid/lymphoid or mixed-lineage leukemia (trithorax homolog, Drosophila); translocated to, 3 | MLLT3 | 0,11 | 0,0084 | 0,22 | 0,1039 | 0,03 | 0,6885 |
| 16760162 | chromosome 12 open reading frame 4 | C12orf4 | 0,11 | 0,0397 | 0,11 | 0,5146 | 0,12 | 0,1332 |
| 17102538 | cytochrome b-245, beta polypeptide | CYBB | 0,11 | 0,0039 | 0,12 | 0,5300 | -0,07 | 0,7620 |
| 16767794 | oxysterol binding protein-like 8 | OSBPL8 | 0,11 | 0,0032 | 0,05 | 0,4016 | 0,04 | 0,6397 |
| 16691668 | notch 2 \| uncharacterized LOC100506528 | NOTCH2\| LOC100506528 | 0,11 | 0,0146 | 0,11 | 0,0583 | 0,17 | 0,0931 |
| 16909537 | phosphodiesterase 6D, cGMP-specific, rod, delta | PDE6D | 0,11 | 0,0389 | -0,01 | 0,9560 | 0 | 0,9762 |
| 16700034 | lin-9 homolog (C. elegans) | LIN9 | 0,11 | 0,0497 | 0,11 | 0,3083 | 0,21 | 0,1064 |
| 16846901 | chromosome 17 open reading frame 67 | C17orf67 | 0,11 | 0,0241 | -0,04 | 0,5190 | 0,16 | 0,2478 |
| 16916233 | myelin transcription factor 1 | MYT1 | 0,11 | 0,0290 | 0,17 | 0,0975 | 0,09 | 0,5445 |
| 16677082 | hedgehog acyltransferase | HHAT | 0,11 | 0,0247 | -0,01 | 0,8956 | 0,02 | 0,7775 |
| 16661459 | syntaxin 12 | STX12 | 0,11 | 0,0427 | 0,08 | 0,4349 | 0,05 | 0,0635 |
| 16754060 | CCR4-NOT transcription complex, subunit 2 | CNOT2 | 0,11 | 0,0231 | -0,03 | 0,7938 | 0,09 | 0,3188 |
| 16736942 | metallophosphoesterase domain containing 2 | MPPED2 | 0,11 | 0,0206 | -0,03 | 0,8366 | -0,1 | 0,1702 |
| 17072439 | fer-1-like 6 (C. elegans) | FER1L6 | 0,11 | 0,0190 | 0,04 | 0,0501 | 0,06 | 0,3752 |
| 17014630 | FGFR1 oncogene partner | FGFR1OP | 0,11 | 0,0499 | 0,14 | 0,1797 | 0,17 | 0,1125 |
| 16826539 | AKT interacting protein \| AKT interacting protein pseudogene | AKTIP\| LOC100130746 | 0,11 | 0,0019 | -0,08 | 0,4394 | 0,14 | 0,1339 |
| 16981893 | claudin 24 | CLDN24 | 0,12 | 0,0202 | 0,09 | 0,4466 | 0,05 | 0,2325 |
| 17092615 | PC4 and SFRS1 interacting protein 1 | PSIP1 | 0,12 | 0,0044 | -0,07 | 0,6269 | 0,1 | 0,1278 |
| 16957929 | RAB, member of RAS oncogene family-like 3 | RABL3 | 0,12 | 0,0271 | 0,02 | 0,8427 | 0,19 | 0,1383 |
| 17108500 | dyskeratosis congenita 1, dyskerin \| small nucleolar RNA, H/ACA box 56 | DKC1\| SNORA56 | 0,12 | 0,0499 | 0,16 | 0,0876 | 0,1 | 0,2351 |
| 16793277 | TATA box binding protein like 2 | TBPL2 | 0,12 | 0,0451 | 0,07 | 0,5250 | -0,07 | 0,5005 |
| 16990294 | protocadherin beta 9 \| protocadherin beta 10 | PCDHB9\| PCDHB10 | 0,12 | 0,0464 | 0,06 | 0,4722 | 0,02 | 0,9141 |
| 16815316 | THO complex 6 homolog (Drosophila) | THOC6 | 0,12 | 0,0197 | 0,13 | 0,1457 | 0,12 | 0,1450 |
| 16938583 | STT3, subunit of the oligosaccharyltransferase complex, homolog B (S. cerevisiae) | STT3B | 0,12 | 0,0397 | 0,07 | 0,1641 | 0,1 | 0,2426 |
| 16945189 | Sec61 alpha 1 subunit (S. cerevisiae) | SEC61A1 | 0,12 | 0,0035 | 0,01 | 0,4231 | 0,05 | 0,3387 |
| 16809506 | cAMP-regulated phosphoprotein, 19kDa | ARPP19 | 0,12 | 0,0073 | -0,15 | 0,0837 | 0,13 | 0,2108 |
| 16826690 | uncharacterized LOC26077 | DKFZP434H168 | 0,12 | 0,0164 | 0,07 | 0,0554 | 0,08 | 0,5188 |
| 16782334 | neuroguidin, EIF4E binding protein | NGDN | 0,12 | 0,0027 | 0,08 | 0,4087 | 0,13 | 0,4482 |
| 16957372 | autophagy related 3 | ATG3 | 0,12 | 0,0370 | 0,09 | 0,3424 | 0,1 | 0,2127 |
| 16839019 | solute carrier family 16, member 3 (monocarboxylic acid transporter 4) | SLC16A3 | 0,12 | 0,0008 | 0,34 | 0,1258 | 0,18 | 0,1158 |
| 16973693 | HAUS augmin-like complex, subunit 3 \| polymerase (DNA directed) nu | HAUS3\|POLN | 0,12 | 0,0135 | 0,19 | 0,0697 | 0,12 | 0,4196 |
| 17006787 | lymphocyte antigen 6 complex, locus G6F \| lymphocyte antigen 6 complex, locus G6D | LY6G6F\|LY6G6D | 0,12 | 0,0001 | 0,02 | 0,8941 | -0,06 | 0,0978 |
| 16794256 | enhancer of rudimentary homolog (Drosophila) | ERH | 0,12 | 0,0158 | 0,13 | 0,3593 | 0,18 | 0,1774 |
| 16822125 | transcription factor 25 (basic helix-loop-helix) | TCF25 | 0,12 | 0,0151 | 0,04 | 0,2758 | 0,08 | 0,4203 |
| 16791991 | nuclear factor of kappa light polypeptide gene enhancer in B-cells inhibitor, alpha | NFKBIA | 0,12 | 0,0357 | 0,02 | 0,6496 | 0,26 | 0,0919 |
| 16728095 | protein phosphatase 6, regulatory subunit 3 | PPP6R3 | 0,12 | 0,0010 | 0,04 | 0,7645 | 0,14 | 0,0644 |
| 16956055 | ubiquitin-like modifier activating enzyme 3 | UBA3 | 0,12 | 0,0283 | 0,13 | 0,2972 | 0,15 | 0,2034 |
| 16979698 | progesterone receptor membrane component 2 \| uncharacterized LOC100653110 \| uncharacterized LOC100652849 | PGRMC2\| LOC100653110\| LOC100652849 | 0,12 | 0,0301 | 0,21 | 0,0864 | 0,02 | 0,7238 |
| 16847159 | chromosome 17 open reading frame 47 | C17orf47 | 0,12 | 0,0234 | 0,07 | 0,3832 | 0,09 | 0,2811 |
| 16744667 |  | BUD13 | 0,12 | 0,0272 | 0,16 | 0,2759 | -0,11 | 0,5723 |
| 16978568 | centromere protein E, 312kDa | CENPE | 0,12 | 0,0233 | 0,06 | 0,7220 | 0,09 | 0,2329 |
| 16984365 | growth hormone receptor | GHR | 0,12 | 0,0369 | -0,06 | 0,1946 | -0,09 | 0,3786 |
| 16793901 | MYC associated factor X | MAX | 0,13 | 0,0034 | 0,09 | 0,2004 | 0,15 | 0,1067 |
| 16792798 | salvador homolog 1 (Drosophila) | SAV1 | 0,13 | 0,0381 | -0,15 | 0,4466 | 0,07 | 0,5372 |
| 16950072 | forty-two-three domain containing 1 | FYTTD1 | 0,13 | 0,0080 | 0,03 | 0,6612 | 0,11 | 0,2823 |
| 17074815 | LON peptidase N-terminal domain and ring finger 1 | LONRF1 | 0,13 | 0,0434 | 0,02 | 0,5977 | 0,1 | 0,4599 |
| 16856453 | glutathione peroxidase 4 | GPX4 | 0,13 | 0,0251 | 0,04 | 0,6181 | 0,06 | 0,0774 |
| 16696903 | acyl-CoA binding domain containing 6 | ACBD6 | 0,13 | 0,0274 | 0,02 | 0,7895 | 0,08 | 0,3450 |
| 16705159 | cyclin-dependent kinase 1 | CDK1 | 0,13 | 0,0458 | 0,13 | 0,4986 | 0,11 | 0,1653 |
| 16985208 | leucine rich repeat containing 70 \| importin 11 | LRRC70\|IPO11 | 0,13 | 0,0035 | 0,13 | 0,1496 | 0,04 | 0,5207 |
| 16876881 | cleavage and polyadenylation specific factor 3, 73kDa | CPSF3 | 0,13 | 0,0165 | 0,06 | 0,2880 | 0,13 | 0,0627 |
| 16681661 | mechanistic target of rapamycin (serine/threonine kinase) | MTOR | 0,13 | 0,0184 | 0,01 | 0,8521 | 0,06 | 0,5158 |
| 17065453 | defensin, beta 103A \| defensin, beta 103B | DEFB103A\| DEFB103B | 0,13 | 0,0119 | 0,17 | 0,1043 | 0,2 | 0,0846 |
| 16701691 | zinc finger, MYND-type containing 11 | ZMYND11 | 0,13 | 0,0399 | 0,00 | 0,9312 | 0,08 | 0,1053 |
| 16937035 | ADP-ribosylation factor-like 8B | ARL8B | 0,13 | 0,0417 | 0,12 | 0,4917 | 0,05 | 0,6912 |
| 16996582 | excision repair cross-complementing rodent repair deficiency, complementation group 8 | ERCC8 | 0,13 | 0,0295 | 0,17 | 0,1194 | 0,08 | 0,4752 |
| 16848079 | WD repeat domain, phosphoinositide interacting 1 | WIPI1 | 0,13 | 0,0423 | 0,05 | 0,3403 | 0,08 | 0,3023 |
| 16832768 | carboxypeptidase D | CPD | 0,13 | 0,0380 | 0,04 | 0,2938 | 0,04 | 0,1979 |
| 16797051 | protein phosphatase 1, regulatory subunit 13B | PPP1R13B | 0,13 | 0,0383 | 0,07 | 0,2384 | 0,04 | 0,4893 |
| 16842673 | sperm associated antigen 5 \| uncharacterized serine/threonine-protein kinase SgK494 | SPAG5\| SGK494 | 0,13 | 0,0039 | -0,01 | 0,8655 | 0,14 | 0,3121 |
| 17089119 | pre-B-cell leukemia homeobox 3 | PBX3 | 0,13 | 0,0332 | 0,06 | 0,4930 | 0,12 | 0,2296 |
| 16732880 | sperm autoantigenic protein 17 | SPA17 | 0,13 | 0,0286 | 0,02 | 0,6679 | 0,1 | 0,0655 |
| 17057354 | transmembrane emp24 protein transport domain containing 4 | TMED4 | 0,13 | 0,0126 | 0,02 | 0,8228 | 0,1 | 0,3738 |
| 16698443 | dual serine/threonine and tyrosine protein kinase | DSTYK | 0,13 | 0,0240 | -0,02 | 0,7840 | 0,04 | 0,6353 |
| 16962359 | transformer 2 beta homolog (Drosophila) | TRA2B | 0,13 | 0,0249 | 0,09 | 0,3625 | 0,18 | 0,0540 |
| 16690493 | WD repeat domain 47 | WDR47 | 0,13 | 0,0361 | -0,04 | 0,7717 | 0,12 | 0,1276 |
| 16936816 | contactin 6 | CNTN6 | 0,13 | 0,0244 | 0,00 | 0,9696 | -0,05 | 0,4883 |
| 16893992 | acireductone dioxygenase 1 | ADI1 | 0,13 | 0,0287 | 0,14 | 0,2836 | 0,1 | 0,2744 |
| 16739552 | nuclear RNA export factor 1 | NXF1 | 0,13 | 0,0344 | 0,13 | 0,1458 | 0,08 | 0,1356 |
| 17104927 |  | JPX | 0,13 | 0,0401 | 0,00 | 0,9837 | 0,14 | 0,3273 |
| 16658991 | patched domain containing 2 | PTCHD2 | 0,13 | 0,0407 | 0,18 | 0,0694 | 0,04 | 0,8148 |
| 16726652 | chromosome 11 open reading frame 2 | C11orf2 | 0,13 | 0,0103 | 0,14 | 0,3507 | 0,13 | 0,1574 |
| 16763882 | ankyrin repeat and SOCS box containing 8 | ASB8 | 0,13 | 0,0163 | 0,09 | 0,0830 | 0,15 | 0,1134 |
| 16861393 | zinc finger protein 146 | ZNF146 | 0,13 | 0,0337 | 0,14 | 0,1629 | 0,07 | 0,5747 |
| 16785606 | membrane protein, palmitoylated 5 (MAGUK p55 subfamily member 5) | MPP5 | 0,13 | 0,0482 | 0,13 | 0,2943 | 0,07 | 0,4995 |
| 17061827 | THAP domain containing 5 \| patatin-like phospholipase domain containing 8 | THAP5\| PNPLA8 | 0,13 | 0,0049 | 0,01 | 0,9555 | 0,02 | 0,8198 |
| 17011893 | triosephosphate isomerase 1 pseudogene 3 | TPI1P3 | 0,13 | 0,0210 | 0,16 | 0,0891 | 0,1 | 0,2203 |
| 16859990 |  | MAU2 | 0,14 | 0,0371 | 0,01 | 0,8766 | 0,12 | 0,5329 |
| 16873751 | N-ethylmaleimide-sensitive factor attachment protein, alpha | NAPA | 0,14 | 0,0081 | -0,01 | 0,8359 | 0,07 | 0,1055 |
| 16897797 | polyribonucleotide nucleotidyltransferase 1 | PNPT1 | 0,14 | 0,0008 | 0,10 | 0,3882 | 0,19 | 0,0611 |
| 17081483 | zinc finger and AT hook domain containing | ZFAT | 0,14 | 0,0352 | 0,02 | 0,6448 | 0 | 0,9953 |
| 16739208 | ferritin, heavy polypeptide 1 | FTH1 | 0,14 | 0,0449 | -0,11 | 0,7003 | 0,15 | 0,1486 |
| 16938271 | K(lysine) acetyltransferase 2B | KAT2B | 0,14 | 0,0207 | -0,01 | 0,9224 | 0,07 | 0,2805 |
| 16825427 | rabaptin, RAB GTPase binding effector protein 2 | RABEP2 | 0,14 | 0,0047 | 0,06 | 0,5751 | 0,09 | 0,5449 |
| 16681064 | ribosomal protein L22 | RPL22 | 0,14 | 0,0071 | 0,04 | 0,6228 | 0,11 | 0,2028 |
| 17061467 | pseudouridylate synthase 7 homolog (S. cerevisiae) | PUS7 | 0,14 | 0,0205 | 0,11 | 0,2368 | 0,25 | 0,0712 |
| 16873800 | sulfotransferase family, cytosolic, 2A, dehydroepiandrosterone (DHEA)-preferring, member 1 | SULT2A1 | 0,14 | 0,0277 | 0,12 | 0,1497 | 0,05 | 0,3263 |
| 16663231 | phosphopantothenoylcysteine synthetase | PPCS | 0,14 | 0,0279 | 0,02 | 0,8809 | 0,01 | 0,8745 |
| 16818733 | HEAT repeat containing 3 | HEATR3 | 0,14 | 0,0093 | 0,13 | 0,1415 | 0,3 | 0,1410 |
| 17084184 | aconitase 1, soluble | ACO1 | 0,14 | 0,0485 | 0,08 | 0,2088 | 0,06 | 0,4770 |
| 16902221 | chromosome 2 open reading frame 76 | C2orf76 | 0,14 | 0,0263 | 0,15 | 0,3514 | 0,17 | 0,1410 |
| 17068398 | 1-acylglycerol-3-phosphate O-acyltransferase 6 (lysophosphatidic acid acyltransferase, zeta) | AGPAT6 | 0,14 | 0,0351 | 0,12 | 0,0522 | 0,12 | 0,2870 |
| 17066897 | solute carrier family 25 (mitochondrial iron transporter), member 37 | SLC25A37 | 0,14 | 0,0045 | 0,04 | 0,7567 | 0,13 | 0,4328 |
| 16937834 | histone deacetylase 11 | HDAC11 | 0,14 | 0,0277 | -0,03 | 0,8643 | 0,01 | 0,9123 |
| 16881770 | ladybird homeobox 2 \| LBX2 antisense RNA 1 (non-protein coding) | LBX2\| LBX2-AS1 | 0,14 | 0,0070 | 0,01 | 0,9173 | -0,14 | 0,2365 |
| 17003898 | mitogen-activated protein kinase 9 | MAPK9 | 0,14 | 0,0388 | 0,02 | 0,8329 | 0,01 | 0,9631 |
| 16854828 | synaptotagmin IV | SYT4 | 0,14 | 0,0446 | 0,07 | 0,1544 | 0,03 | 0,8197 |
| 16821949 | zinc finger protein 778 | ZNF778 | 0,14 | 0,0315 | 0,06 | 0,4361 | 0,14 | 0,3275 |
| 17001134 | Yip1 domain family, member 5 | YIPF5 | 0,14 | 0,0271 | 0,06 | 0,1138 | 0,1 | 0,1711 |
| 16986249 | 3-hydroxy-3-methylglutaryl-CoA reductase | HMGCR | 0,14 | 0,0175 | 0,04 | 0,4009 | 0,05 | 0,5255 |
| 16812383 | mesoderm development candidate 2 | MESDC2 | 0,14 | 0,0086 | 0,13 | 0,1679 | 0,16 | 0,3828 |
| 16683852 | zinc finger protein 683 | ZNF683 | 0,14 | 0,0317 | 0,21 | 0,4357 | 0,2 | 0,4475 |
| 16815090 | cyclin F | CCNF | 0,15 | 0,0316 | 0,03 | 0,6089 | 0,09 | 0,3174 |
| 16923031 | myxovirus (influenza virus) resistance 1, interferon-inducible protein p78 (mouse) | MX1 | 0,15 | 0,0089 | 0,14 | 0,0554 | 0,13 | 0,3451 |
| 16854064 | protein tyrosine phosphatase, non-receptor type 2 | PTPN2 | 0,15 | 0,0375 | 0,19 | 0,2549 | 0,21 | 0,0534 |
| 16849501 | cytohesin 1 | CYTH1 | 0,15 | 0,0409 | 0,06 | 0,2055 | 0,1 | 0,5444 |
| 16836697 | breast carcinoma amplified sequence 3 | BCAS3 | 0,15 | 0,0035 | 0,04 | 0,7670 | 0,03 | 0,7770 |
| 16807848 | vacuolar protein sorting 39 homolog (S. cerevisiae) | VPS39 | 0,15 | 0,0261 | -0,01 | 0,9404 | 0,08 | 0,4605 |
| 16998551 | solute carrier organic anion transporter family, member 4C1 | SLCO4C1 | 0,15 | 0,0039 | 0,18 | 0,1864 | 0,38 | 0,0981 |
| 17098911 | zer-1 homolog (C. elegans) | ZER1 | 0,15 | 0,0233 | -0,01 | 0,9581 | 0,08 | 0,5500 |
| 16994588 | zinc finger protein 622 | ZNF622 | 0,15 | 0,0151 | -0,05 | 0,1911 | 0,12 | 0,3712 |
| 16851565 | tetratricopeptide repeat domain 39C | TTC39C | 0,15 | 0,0420 | 0,15 | 0,2098 | -0,07 | 0,2508 |
| 16731654 | transgelin | TAGLN | 0,15 | 0,0087 | 0,03 | 0,7561 | 0,06 | 0,4754 |
| 16905371 |  | KIAA1715 | 0,15 | 0,0255 | -0,02 | 0,8470 | 0,07 | 0,4362 |
| 16959061 | transmembrane and coiled-coil domain family 1 | TMCC1 | 0,15 | 0,0036 | -0,01 | 0,9668 | 0,11 | 0,1253 |
| 16793067 | fermitin family member 2 | FERMT2 | 0,15 | 0,0225 | 0,22 | 0,1724 | 0,19 | 0,2559 |
| 17022562 | solute carrier family 22 (organic cation/carnitine transporter), member 16 | SLC22A16 | 0,15 | 0,0395 | 0,23 | 0,0900 | 0,15 | 0,3126 |
| 16838107 | methyltransferase like 23 | METTL23 | 0,15 | 0,0493 | 0,09 | 0,4465 | 0,24 | 0,1792 |
| 16814336 |  | RAB40C | 0,15 | 0,0248 | 0,09 | 0,3598 | 0,15 | 0,2654 |
| 17089027 | actin related protein 2/3 complex, subunit 5-like | ARPC5L | 0,15 | 0,0369 | 0,08 | 0,4777 | 0,11 | 0,4793 |
| 16944363 | protein O-glucosyltransferase 1 | POGLUT1 | 0,15 | 0,0198 | 0,12 | 0,1043 | 0,1 | 0,0737 |
| 16866093 | zinc finger protein 134 \| zinc finger protein 211 | ZNF134\| ZNF211 | 0,15 | 0,0014 | -0,02 | 0,8726 | 0,11 | 0,4598 |
| 16846993 | serine/arginine-rich splicing factor 1 | SRSF1 | 0,15 | 0,0167 | 0,04 | 0,1096 | 0,1 | 0,2403 |
| 16920299 | potassium voltage-gated channel, subfamily G, member 1 | KCNG1 | 0,15 | 0,0337 | 0,03 | 0,6116 | -0,05 | 0,5831 |
| 17009918 | protein tyrosine phosphatase type IVA, member 1 | PTP4A1 | 0,15 | 0,0174 | 0,14 | 0,2429 | 0 | 0,9676 |
| 16976177 | HOP homeobox | HOPX | 0,15 | 0,0367 | 0,05 | 0,7325 | -0,05 | 0,7584 |
| 16939592 | trafficking protein, kinesin binding 1 | TRAK1 | 0,15 | 0,0057 | 0,10 | 0,1646 | 0,1 | 0,0909 |
| 16688164 | Janus kinase 1 | JAK1 | 0,15 | 0,0109 | 0,00 | 0,9985 | 0,07 | 0,3593 |
| 16860587 | G patch domain containing 1 | GPATCH1 | 0,15 | 0,0476 | 0,12 | 0,1650 | 0,15 | 0,1381 |
| 16664460 | forkhead box D2 | FOXD2 | 0,15 | 0,0459 | -0,11 | 0,4240 | -0,01 | 0,9451 |
| 16717452 | ER lipid raft associated 1 | ERLIN1 | 0,15 | 0,0362 | 0,09 | 0,2787 | 0,08 | 0,3920 |
| 17074366 | defensin, beta 103A \| defensin, beta 103B | DEFB103A\| DEFB103B | 0,15 | 0,0446 | 0,19 | 0,1417 | 0,22 | 0,1082 |
| 17019218 | transcriptional regulating factor 1 | TRERF1 | 0,15 | 0,0412 | 0,14 | 0,1704 | 0,12 | 0,3952 |
| 16938899 | mutL homolog 1, colon cancer, nonpolyposis type 2 (E. coli) | MLH1 | 0,15 | 0,0094 | -0,07 | 0,5761 | 0,14 | 0,3455 |
| 16914509 | nuclear receptor coactivator 3 | NCOA3 | 0,15 | 0,0349 | 0,05 | 0,1336 | 0,16 | 0,0544 |
| 16953775 | ariadne homolog 2 (Drosophila) | ARIH2 | 0,15 | 0,0028 | 0,20 | 0,3281 | 0,34 | 0,1456 |
| 16681192 | nucleolar protein 9 | NOL9 | 0,16 | 0,0230 | -0,07 | 0,6647 | 0,13 | 0,1366 |
| 16921527 | RNA binding motif protein 11 | RBM11 | 0,16 | 0,0207 | 0,05 | 0,4924 | 0,31 | 0,1224 |
| 16970937 | ELMO/CED-12 domain containing 2 | ELMOD2 | 0,16 | 0,0025 | 0,14 | 0,1538 | 0,12 | 0,4506 |
| 17076046 | general transcription factor IIE, polypeptide 2, beta 34kDa | GTF2E2 | 0,16 | 0,0159 | 0,01 | 0,9497 | 0,11 | 0,3404 |
| 16882975 | non-SMC condensin I complex, subunit H | NCAPH | 0,16 | 0,0410 | 0,14 | 0,3592 | 0,15 | 0,1354 |
| 16911432 | chromosome 20 open reading frame 94 | C20orf94 | 0,16 | 0,0428 | -0,01 | 0,7863 | -0,03 | 0,8569 |
| 16796048 | NADH dehydrogenase (ubiquinone) 1 beta subcomplex, 1, 7kDa | NDUFB1 | 0,16 | 0,0328 | 0,19 | 0,1569 | 0,05 | 0,5292 |
| 16967892 | THAP domain containing 6 | THAP6 | 0,16 | 0,0203 | 0,09 | 0,1508 | 0,12 | 0,1650 |
| 16953778 | DALR anticodon binding domain containing 3 | DALRD3 | 0,16 | 0,0301 | 0,13 | 0,4850 | 0,05 | 0,6781 |
| 16849861 | chromosome 17 open reading frame 70 | C17orf70 | 0,16 | 0,0119 | 0,10 | 0,5155 | 0,03 | 0,9225 |
| 17023887 | HBS1-like (S. cerevisiae) | HBS1L | 0,16 | 0,0081 | 0,15 | 0,2398 | 0,11 | 0,2983 |
| 16897401 | luteinizing hormone/choriogonadotropin receptor | LHCGR | 0,16 | 0,0374 | 0,13 | 0,4749 | 0,22 | 0,3355 |
| 16898518 | protein phosphatase 3, regulatory subunit B, alpha | PPP3R1 | 0,16 | 0,0051 | 0,12 | 0,1442 | 0,07 | 0,4461 |
| 16986895 | X-ray repair complementing defective repair in Chinese hamster cells 4 | XRCC4 | 0,16 | 0,0079 | 0,26 | 0,1302 | 0,2 | 0,1085 |
| 16851397 | retinoblastoma binding protein 8 | RBBP8 | 0,16 | 0,0457 | 0,10 | 0,2903 | 0,07 | 0,4204 |
| 16902945 | NCK-associated protein 5 | NCKAP5 | 0,16 | 0,0276 | 0,19 | 0,2182 | 0,11 | 0,5161 |
| 16758601 | small nuclear ribonucleoprotein 35kDa (U11/U12) \| Rab interacting lysosomal protein-like 1 | SNRNP35\| RILPL1 | 0,16 | 0,0047 | -0,01 | 0,8539 | 0,17 | 0,0539 |
| 16749524 | kelch domain containing 5 | KLHDC5 | 0,16 | 0,0334 | 0,12 | 0,1082 | 0,18 | 0,1494 |
| 16774789 | SET domain, bifurcated 2 | SETDB2 | 0,16 | 0,0500 | 0,04 | 0,0886 | 0,12 | 0,4566 |
| 16848453 | solute carrier family 39 (metal ion transporter), member 11 | SLC39A11 | 0,16 | 0,0184 | 0,14 | 0,1944 | 0 | 0,9803 |
| 17078916 | nibrin | NBN | 0,16 | 0,0300 | 0,08 | 0,1343 | 0,08 | 0,5074 |
| 16854145 | Rho-associated, coiled-coil containing protein kinase 1 | ROCK1 | 0,16 | 0,0456 | 0,15 | 0,2422 | 0,12 | 0,1032 |
| 16796938 | CDC42 binding protein kinase beta (DMPK-like) | CDC42BPB | 0,16 | 0,0471 | -0,01 | 0,8556 | 0,13 | 0,0631 |
| 16703858 | cAMP responsive element modulator | CREM | 0,16 | 0,0244 | 0,03 | 0,5337 | 0,21 | 0,1576 |
| 16711866 | selenophosphate synthetase 1 | SEPHS1 | 0,16 | 0,0454 | 0,07 | 0,1730 | 0,13 | 0,2429 |
| 16858386 | low density lipoprotein receptor | LDLR | 0,16 | 0,0160 | 0,06 | 0,3737 | 0,18 | 0,1615 |
| 16756431 | chromosome 12 open reading frame 23 | C12orf23 | 0,16 | 0,0035 | -0,01 | 0,9442 | 0,13 | 0,3236 |
| 17012888 | peroxisomal biogenesis factor 7 | PEX7 | 0,16 | 0,0210 | 0,07 | 0,3972 | 0,15 | 0,4616 |
| 17033451 | lymphocyte antigen 6 complex, locus G6F \| lymphocyte antigen 6 complex, locus G6D | LY6G6F\| LY6G6D | 0,16 | 0,0211 | 0,04 | 0,6920 | -0,04 | 0,5129 |
| 17097813 | CDK5 regulatory subunit associated protein 2 | CDK5RAP2 | 0,16 | 0,0498 | 0,01 | 0,8125 | 0,08 | 0,3630 |
| 17087753 | olfactory receptor, family 13, subfamily C, member 8 | OR13C8 | 0,16 | 0,0361 | -0,06 | 0,8037 | 0,06 | 0,6795 |
| 16724593 | protein tyrosine phosphatase, mitochondrial 1 \| NADH dehydrogenase (ubiquinone) Fe-S protein 3, 30kDa (NADH-coenzyme Q reductase) | PTPMT1\| NDUFS3 | 0,16 | 0,0217 | 0,06 | 0,4922 | 0,16 | 0,3422 |
| 16772625 | polymerase (DNA directed), epsilon, catalytic subunit | POLE | 0,16 | 0,0221 | 0,05 | 0,4780 | 0,19 | 0,0790 |
| 16888822 | major facilitator superfamily domain containing 6 | MFSD6 | 0,16 | 0,0121 | 0,09 | 0,1614 | 0,08 | 0,1344 |
| 16658005 |  | RER1 | 0,16 | 0,0285 | 0,06 | 0,7363 | 0,15 | 0,0923 |
| 16706822 | GRID1 antisense RNA 1 (non-protein coding) | GRID1-AS1 | 0,17 | 0,0411 | -0,03 | 0,7847 | 0,11 | 0,5188 |
| 16817630 | quinolinate phosphoribosyltransferase | QPRT | 0,17 | 0,0185 | 0,21 | 0,1376 | 0,06 | 0,7550 |
| 16927040 | mitochondrial ribosomal protein L40 | MRPL40 | 0,17 | 0,0331 | 0,10 | 0,4652 | 0,19 | 0,1275 |
| 17109367 | CTP synthase 2 | CTPS2 | 0,17 | 0,0265 | 0,10 | 0,2724 | 0,12 | 0,1176 |
| 16921948 | ubiquitin specific peptidase 16 | USP16 | 0,17 | 0,0384 | 0,12 | 0,2608 | 0,1 | 0,5227 |
| 17046284 | sulfatase modifying factor 2 | SUMF2 | 0,17 | 0,0029 | 0,09 | 0,2939 | 0,03 | 0,6940 |
| 16678954 | geranylgeranyl diphosphate synthase 1 | GGPS1 | 0,17 | 0,0350 | 0,15 | 0,0886 | 0,12 | 0,2058 |
| 16678579 | UDP-N-acetyl-alpha-D-galactosamine:polypeptide N-acetylgalactosaminyltransferase 2 (GalNAc-T2) | GALNT2 | 0,17 | 0,0475 | 0,15 | 0,1200 | 0,05 | 0,5608 |
| 17030746 | lymphocyte antigen 6 complex, locus G6F \| lymphocyte antigen 6 complex, locus G6D | LY6G6F\| LY6G6D | 0,17 | 0,0275 | 0,05 | 0,6020 | -0,03 | 0,6047 |
| 16699983 | pyrroline-5-carboxylate reductase family, member 2 | PYCR2 | 0,17 | 0,0084 | 0,04 | 0,7010 | 0,15 | 0,3786 |
| 16788296 | echinoderm microtubule associated protein like 1 | EML1 | 0,17 | 0,0019 | 0,14 | 0,1861 | 0,05 | 0,4807 |
| 17071208 | lysosomal protein transmembrane 4 beta | LAPTM4B | 0,17 | 0,0261 | 0,18 | 0,0565 | 0,09 | 0,1327 |
| 16867047 | amino-terminal enhancer of split | AES | 0,17 | 0,0281 | 0,09 | 0,4244 | 0,08 | 0,5650 |
| 17113047 | solute carrier family 25, member 53 | SLC25A53 | 0,17 | 0,0307 | 0,07 | 0,5096 | 0,04 | 0,7141 |
| 17009316 | ectonucleotide pyrophosphatase/phosphodiesterase 4 (putative) | ENPP4 | 0,17 | 0,0241 | -0,02 | 0,8323 | 0,09 | 0,6723 |
| 17068003 | BCL2-associated athanogene 4 | BAG4 | 0,17 | 0,0335 | 0,03 | 0,3535 | 0,18 | 0,3601 |
| 16862866 | zinc finger protein 221 | ZNF221 | 0,17 | 0,0296 | 0,23 | 0,1553 | 0,14 | 0,4993 |
| 16879145 | chromosome 2 open reading frame 56 | C2orf56 | 0,17 | 0,0330 | 0,24 | 0,1356 | 0,14 | 0,0947 |
| 16731945 | archain 1 | ARCN1 | 0,17 | 0,0347 | 0,12 | 0,1000 | 0,1 | 0,1155 |
| 17090526 | proline-rich coiled-coil 2B | PRRC2B | 0,17 | 0,0125 | 0,01 | 0,9225 | 0,09 | 0,4947 |
| 16834045 | WAS/WASL interacting protein family, member 2 | WIPF2 | 0,17 | 0,0279 | 0,06 | 0,2325 | 0,04 | 0,6584 |
| 16799170 | chromosome 15 open reading frame 41 | C15orf41 | 0,17 | 0,0383 | 0,15 | 0,0832 | 0,12 | 0,4869 |
| 17079477 | serine/threonine kinase 3 | STK3 | 0,17 | 0,0450 | 0,05 | 0,3318 | 0,12 | 0,3190 |
| 16978113 | tetraspanin 5 | TSPAN5 | 0,17 | 0,0462 | 0,11 | 0,2426 | 0,14 | 0,1901 |
| 16933960 | dual specificity phosphatase 18 | DUSP18 | 0,17 | 0,0140 | 0,04 | 0,7044 | 0,11 | 0,5303 |
| 16795987 | thyroid hormone receptor interactor 11 | TRIP11 | 0,17 | 0,0198 | 0,09 | 0,4200 | 0,02 | 0,8069 |
| 17009054 | polymerase (DNA directed), eta | POLH | 0,17 | 0,0074 | 0,01 | 0,5709 | 0 | 0,9825 |
| 16833082 | ras homolog family member T1 | RHOT1 | 0,17 | 0,0005 | 0,09 | 0,3198 | 0,16 | 0,2879 |
| 16987720 | diphosphoinositol pentakisphosphate kinase 2 | PPIP5K2 | 0,17 | 0,0016 | 0,05 | 0,5722 | 0,06 | 0,4918 |
| 16688269 | solute carrier family 35 (UDP-glucuronic acid/UDP-N-acetylgalactosamine dual transporter), member D1 | SLC35D1 | 0,17 | 0,0041 | 0,14 | 0,1209 | 0,11 | 0,4363 |
| 16683317 | ArfGAP with SH3 domain, ankyrin repeat and PH domain 3 | ASAP3 | 0,17 | 0,0230 | 0,16 | 0,2608 | 0,13 | 0,2329 |
| 16697166 | ER degradation enhancer, mannosidase alpha-like 3 | EDEM3 | 0,17 | 0,0036 | -0,04 | 0,2663 | 0,01 | 0,9052 |
| 16718800 | RAB11 family interacting protein 2 (class I) | RAB11FIP2 | 0,17 | 0,0261 | 0,18 | 0,1180 | 0,08 | 0,5657 |
| 16865214 | CCR4-NOT transcription complex, subunit 3 | CNOT3 | 0,17 | 0,0004 | 0,04 | 0,4600 | 0,09 | 0,1325 |
| 16921171 | highly accelerated region 1B (non-protein coding) \| highly accelerated region 1A (non-protein coding) | HAR1B\| HAR1A | 0,18 | 0,0311 | 0,00 | 0,9462 | 0,09 | 0,3497 |
| 16828292 | golgi glycoprotein 1 | GLG1 | 0,18 | 0,0441 | 0,07 | 0,2428 | 0,14 | 0,2168 |
| 16932928 | preferentially expressed antigen in melanoma | PRAME | 0,18 | 0,0345 | 0,10 | 0,4279 | -0,03 | 0,6617 |
| 17060186 | SMAD specific E3 ubiquitin protein ligase 1 | SMURF1 | 0,18 | 0,0227 | 0,06 | 0,5031 | 0,17 | 0,1588 |
| 16779792 | TBC1 domain family, member 4 | TBC1D4 | 0,18 | 0,0296 | 0,15 | 0,1598 | 0,14 | 0,2623 |
| 16770284 | transmembrane protein 116 | TMEM116 | 0,18 | 0,0044 | 0,05 | 0,4743 | 0,2 | 0,0829 |
| 16699706 | tumor protein p53 binding protein, 2 | TP53BP2 | 0,18 | 0,0327 | 0,02 | 0,7368 | 0,1 | 0,3752 |
| 17076867 | protein kinase, DNA-activated, catalytic polypeptide | PRKDC | 0,18 | 0,0384 | 0,07 | 0,6238 | 0,18 | 0,0999 |
| 16840550 | dishevelled, dsh homolog 2 (Drosophila) | DVL2 | 0,18 | 0,0336 | 0,22 | 0,2889 | 0,13 | 0,3308 |
| 17076829 | ring finger protein 170 \| microRNA 4469 | RNF170\|MIR4469 | 0,18 | 0,0031 | -0,05 | 0,2650 | 0,07 | 0,4974 |
| 16855637 | phosphatidylinositol glycan anchor biosynthesis, class N | PIGN | 0,18 | 0,0480 | 0,00 | 0,9810 | 0,14 | 0,3487 |
| 17075426 | tumor necrosis factor receptor superfamily, member 10b | TNFRSF10B | 0,18 | 0,0200 | 0,10 | 0,5546 | 0,14 | 0,2577 |
| 17093474 | ribonuclease P/MRP 25kDa subunit-like | RPP25L | 0,18 | 0,0016 | 0,24 | 0,1238 | 0,1 | 0,2464 |
| 17091239 | peptidase (mitochondrial processing) alpha | PMPCA | 0,18 | 0,0081 | 0,03 | 0,6416 | 0,08 | 0,4812 |
| 16877888 | ethanolaminephosphotransferase 1 (CDP-ethanolamine-specific) | EPT1 | 0,18 | 0,0402 | 0,10 | 0,2891 | 0,17 | 0,0555 |
| 16746341 | uncharacterized LOC283174 \| microRNA 4697 | LOC283174\| MIR4697 | 0,18 | 0,0285 | 0,19 | 0,3627 | 0,15 | 0,2430 |
| 16968735 | HECT and RLD domain containing E3 ubiquitin protein ligase family member 6 | HERC6 | 0,18 | 0,0461 | 0,08 | 0,4050 | 0,19 | 0,1447 |
| 16790381 | methyltransferase like 3 | METTL3 | 0,18 | 0,0492 | 0,10 | 0,4748 | 0,09 | 0,1275 |
| 16902646 | heparan sulfate 6-O-sulfotransferase 1 | HS6ST1 | 0,18 | 0,0485 | 0,02 | 0,8793 | -0,01 | 0,9386 |
| 16710948 | mitochondrial GTPase 1 homolog (S. cerevisiae) | MTG1 | 0,18 | 0,0483 | 0,02 | 0,5290 | 0,14 | 0,0527 |
| 16694913 | Fc receptor-like 4 | FCRL4 | 0,18 | 0,0427 | 0,26 | 0,1685 | 0,1 | 0,2664 |
| 16889202 | chromosome 2 open reading frame 69 | C2orf69 | 0,18 | 0,0421 | 0,05 | 0,5292 | 0,1 | 0,3373 |
| 16673268 | uncharacterized LOC100505828 \| microsomal glutathione S-transferase 3 | LOC100505828\|MGST3 | 0,18 | 0,0048 | 0,13 | 0,0602 | 0,01 | 0,8608 |
| 17015862 | ataxin 1 | ATXN1 | 0,18 | 0,0174 | 0,13 | 0,4888 | 0,13 | 0,1837 |
| 16782876 | KH and NYN domain containing | KHNYN | 0,18 | 0,0356 | 0,03 | 0,3300 | 0,13 | 0,1827 |
| 16883088 | cyclin M3 | CNNM3 | 0,18 | 0,0074 | 0,09 | 0,3764 | 0,12 | 0,1286 |
| 16774623 | leucine-rich repeats and calponin homology (CH) domain containing 1 | LRCH1 | 0,18 | 0,0295 | 0,12 | 0,4936 | 0,19 | 0,0969 |
| 16830146 |  | MIS12 | 0,18 | 0,0285 | 0,13 | 0,1635 | 0,26 | 0,1480 |
| 16944120 | GRAM domain containing 1C | GRAMD1C | 0,18 | 0,0211 | 0,06 | 0,7247 | 0,02 | 0,7188 |
| 16889636 | bone morphogenetic protein receptor, type II (serine/threonine kinase) | BMPR2 | 0,18 | 0,0483 | -0,03 | 0,7115 | 0,1 | 0,3009 |
| 16896256 | mediator of cell motility 1 \| dpy-30 homolog (C. elegans) \| mediator of cell motility 1 pseudogene 1 | MEMO1\| DPY30\| MEMO1P1 | 0,18 | 0,0493 | 0,09 | 0,5005 | 0,12 | 0,3946 |
| 16922887 | immunoglobulin superfamily, member 5 | IGSF5 | 0,18 | 0,0129 | 0,15 | 0,1432 | 0,11 | 0,4255 |
| 17086987 | protein tyrosine phosphatase domain containing 1 | PTPDC1 | 0,18 | 0,0346 | 0,06 | 0,4315 | 0,05 | 0,6297 |
| 16778652 | TSC22 domain family, member 1 | TSC22D1 | 0,18 | 0,0310 | 0,06 | 0,3541 | 0,18 | 0,1488 |
| 17025937 | PHD finger protein 10 | PHF10 | 0,19 | 0,0003 | 0,03 | 0,7706 | 0,17 | 0,1850 |
| 17020480 | glucuronidase, beta pseudogene 4 | GUSBP4 | 0,19 | 0,0429 | 0,30 | 0,0624 | 0,15 | 0,0952 |
| 16877172 | growth regulation by estrogen in breast cancer 1 | GREB1 | 0,19 | 0,0111 | 0,19 | 0,1568 | 0,05 | 0,6725 |
| 16700009 |  | SDE2 | 0,19 | 0,0271 | 0,13 | 0,0894 | 0,15 | 0,2494 |
| 17112198 | ring finger protein, LIM domain interacting | RLIM | 0,19 | 0,0305 | 0,04 | 0,8358 | 0,15 | 0,2317 |
| 16887589 | cytochrome b reductase 1 | CYBRD1 | 0,19 | 0,0177 | 0,23 | 0,0573 | 0,2 | 0,0714 |
| 16692701 | OTU domain containing 7B | OTUD7B | 0,19 | 0,0012 | -0,06 | 0,6342 | 0,04 | 0,7319 |
| 16870131 | HAUS augmin-like complex, subunit 8 | HAUS8 | 0,19 | 0,0386 | -0,04 | 0,8412 | 0,23 | 0,1283 |
| 17014031 | AT rich interactive domain 1B (SWI1-like) | ARID1B | 0,19 | 0,0471 | 0,10 | 0,2916 | 0,03 | 0,7388 |
| 16680935 | chromosome 1 open reading frame 174 | C1orf174 | 0,19 | 0,0467 | 0,15 | 0,1534 | 0,14 | 0,1790 |
| 16850337 | N-ethylmaleimide-sensitive factor pseudogene 1 \| vesicle-fusing ATPase-like | NSFP1\| LOC100507699 | 0,19 | 0,0099 | 0,03 | 0,5795 | 0,1 | 0,0745 |
| 17075506 | ectonucleoside triphosphate diphosphohydrolase 4 | ENTPD4 | 0,19 | 0,0386 | 0,06 | 0,3507 | 0,07 | 0,2997 |
| 16822438 | transmembrane protein 8A | TMEM8A | 0,19 | 0,0403 | 0,01 | 0,8232 | 0,09 | 0,2749 |
| 16929283 | DEP domain containing 5 | DEPDC5 | 0,19 | 0,0248 | 0,05 | 0,5912 | 0,17 | 0,1737 |
| 16816214 | ATP-binding cassette, sub-family C (CFTR/MRP), member 1 | ABCC1 | 0,19 | 0,0107 | 0,15 | 0,0552 | 0,09 | 0,2467 |
| 16884335 | BCL2-like 11 (apoptosis facilitator) | BCL2L11 | 0,19 | 0,0217 | 0,09 | 0,4176 | 0,14 | 0,3430 |
| 16971139 | ATP-binding cassette, sub-family E (OABP), member 1 | ABCE1 | 0,19 | 0,0074 | 0,16 | 0,1214 | 0,12 | 0,2107 |
| 16835064 | N-ethylmaleimide-sensitive factor pseudogene 1 \| vesicle-fusing ATPase-like | NSFP1\| LOC100507699 | 0,19 | 0,0106 | 0,03 | 0,5884 | 0,09 | 0,0759 |
| 16669796 | thioredoxin interacting protein | TXNIP | 0,19 | 0,0312 | 0,08 | 0,4103 | 0 | 0,9947 |
| 16859788 | pyroglutamyl-peptidase I \| FK506 binding protein 8, 38kDa | PGPEP1\|FKBP8 | 0,19 | 0,0226 | 0,08 | 0,5557 | 0,18 | 0,0791 |
| 16971505 | ADP-ribosylation factor interacting protein 1 | ARFIP1 | 0,19 | 0,0071 | 0,05 | 0,4571 | -0,02 | 0,7636 |
| 16705260 | receptor accessory protein 3 | REEP3 | 0,19 | 0,0249 | 0,08 | 0,3318 | 0,01 | 0,9411 |
| 16908415 | zinc finger protein 142 | ZNF142 | 0,19 | 0,0050 | 0,09 | 0,2686 | 0,13 | 0,1709 |
| 16667183 | metal response element binding transcription factor 2 | MTF2 | 0,19 | 0,0353 | 0,11 | 0,3148 | 0,29 | 0,0583 |
| 17024980 | F-box protein 5 | FBXO5 | 0,19 | 0,0406 | 0,12 | 0,1877 | 0,1 | 0,4593 |
| 16741223 | suppressor of variegation 4-20 homolog 1 (Drosophila) | SUV420H1 | 0,19 | 0,0141 | 0,09 | 0,3086 | 0,16 | 0,0755 |
| 16920885 | TAF4 RNA polymerase II, TATA box binding protein (TBP)-associated factor, 135kDa \| microRNA 1257 | TAF4\| MIR1257 | 0,19 | 0,0413 | 0,18 | 0,1468 | 0,12 | 0,1624 |
| 16678710 | glyceronephosphate O-acyltransferase | GNPAT | 0,19 | 0,0172 | 0,04 | 0,6376 | 0,08 | 0,4718 |
| 16830789 | chromodomain helicase DNA binding protein 3 | CHD3 | 0,19 | 0,0190 | 0,07 | 0,4773 | 0,11 | 0,0890 |
| 16851121 | SEH1-like (S. cerevisiae) | SEH1L | 0,19 | 0,0147 | 0,16 | 0,0578 | 0,11 | 0,1057 |
| 16826320 | ATP-binding cassette, sub-family C (CFTR/MRP), member 11 | ABCC11 | 0,19 | 0,0090 | -0,14 | 0,3828 | 0,29 | 0,0838 |
| 16924928 | SR-related CTD-associated factor 4 | SCAF4 | 0,20 | 0,0257 | 0,08 | 0,2513 | 0,14 | 0,2776 |
| 17001718 | dynactin 4 (p62) | DCTN4 | 0,20 | 0,0100 | -0,01 | 0,8672 | 0,14 | 0,2425 |
| 16695508 | thiosulfate sulfurtransferase (rhodanese)-like domain containing 1 \| F11 receptor | TSTD1\| F11R | 0,20 | 0,0127 | 0,27 | 0,1451 | 0,11 | 0,3616 |
| 17074571 | PIN2/TERF1 interacting, telomerase inhibitor 1 \| SRY (sex determining region Y)-box 7 | PINX1\| SOX7 | 0,20 | 0,0430 | 0,13 | 0,2550 | 0,21 | 0,1796 |
| 16935130 | Josephin domain containing 1 | JOSD1 | 0,20 | 0,0102 | 0,02 | 0,4494 | 0,15 | 0,1398 |
| 16864427 | myosin, heavy chain 14, non-muscle | MYH14 | 0,20 | 0,0321 | -0,05 | 0,7411 | 0,17 | 0,4389 |
| 16852445 | chromosome 18 open reading frame 54 | C18orf54 | 0,20 | 0,0188 | 0,03 | 0,8549 | 0,23 | 0,1020 |
| 17104908 | cysteine-rich hydrophobic domain 1 | CHIC1 | 0,20 | 0,0441 | 0,01 | 0,7811 | 0,1 | 0,4355 |
| 16723832 | apoptosis inhibitor 5 | API5 | 0,20 | 0,0252 | 0,12 | 0,3467 | 0,15 | 0,1061 |
| 17092688 | HAUS augmin-like complex, subunit 6 | HAUS6 | 0,20 | 0,0293 | 0,19 | 0,1185 | 0,36 | 0,2087 |
| 16865917 | zinc finger protein 471 | ZNF471 | 0,20 | 0,0148 | 0,00 | 0,9716 | 0,02 | 0,8764 |
| 17079808 | ribonucleotide reductase M2 B (TP53 inducible) | RRM2B | 0,20 | 0,0019 | 0,11 | 0,3630 | 0,21 | 0,3640 |
| 16730471 |  | KIAA1377 | 0,20 | 0,0293 | 0,15 | 0,1043 | 0,26 | 0,0508 |
| 16758336 | kinetochore associated 1 | KNTC1 | 0,20 | 0,0428 | 0,11 | 0,1673 | 0,17 | 0,1034 |
| 16754324 | ataxin 7-like 3B | ATXN7L3B | 0,20 | 0,0269 | 0,05 | 0,4905 | 0,17 | 0,0776 |
| 16939960 | kinesin family member 15 | KIF15 | 0,20 | 0,0133 | 0,02 | 0,6565 | 0,22 | 0,0582 |
| 16717986 | 5'-nucleotidase, cytosolic II | NT5C2 | 0,20 | 0,0034 | 0,07 | 0,4816 | 0,29 | 0,0653 |
| 16991886 | methionine adenosyltransferase II, beta | MAT2B | 0,20 | 0,0371 | 0,25 | 0,0531 | 0,18 | 0,2993 |
| 16664765 | zyg-11 homolog B (C. elegans) | ZYG11B | 0,20 | 0,0116 | 0,00 | 0,9755 | 0,11 | 0,3820 |
| 16900022 | lysine-rich coiled-coil 1 | KRCC1 | 0,20 | 0,0455 | 0,01 | 0,9432 | 0,21 | 0,0705 |
| 16910994 | attractin | ATRN | 0,20 | 0,0429 | -0,04 | 0,6202 | 0,13 | 0,2824 |
| 16681542 | castor zinc finger 1 | CASZ1 | 0,20 | 0,0185 | 0,08 | 0,4794 | 0,09 | 0,1019 |
| 17007475 | PHD finger protein 1 | PHF1 | 0,20 | 0,0128 | 0,07 | 0,3919 | 0,15 | 0,1825 |
| 17046886 | general transcription factor IIi, pseudogene 1 \| general transcription factor IIi \| general transcription factor II, i, pseudogene | GTF2IP1\| LOC100093631 | 0,20 | 0,0448 | 0,06 | 0,5366 | 0,13 | 0,1324 |
| 16952639 | HIG1 hypoxia inducible domain family, member 1A | HIGD1A | 0,20 | 0,0172 | 0,19 | 0,1334 | 0,08 | 0,3494 |
| 16959465 | anaphase promoting complex subunit 13 | ANAPC13 | 0,20 | 0,0269 | 0,24 | 0,2362 | 0,1 | 0,0616 |
| 16944763 | SEC22 vesicle trafficking protein homolog A (S. cerevisiae) \| protein disulfide isomerase family A, member 5 | SEC22A\|PDIA5 | 0,20 | 0,0245 | 0,10 | 0,3122 | 0,13 | 0,1024 |
| 16898872 | testis expressed 261 | TEX261 | 0,20 | 0,0386 | 0,06 | 0,1347 | 0,1 | 0,0557 |
| 16926679 | minichromosome maintenance complex component 3 associated protein | MCM3AP | 0,20 | 0,0007 | -0,01 | 0,9527 | 0,31 | 0,0568 |
| 17069212 | syndecan binding protein (syntenin) | SDCBP | 0,21 | 0,0476 | 0,12 | 0,0563 | 0,14 | 0,0798 |
| 16751209 | LETM1 domain containing 1 | LETMD1 | 0,21 | 0,0386 | 0,14 | 0,1216 | 0,23 | 0,0775 |
| 16842856 | PHD finger protein 12 | PHF12 | 0,21 | 0,0311 | 0,07 | 0,1499 | 0,14 | 0,2332 |
| 16702608 | PRP18 pre-mRNA processing factor 18 homolog (S. cerevisiae) | PRPF18 | 0,21 | 0,0466 | 0,12 | 0,1325 | 0,17 | 0,1189 |
| 17099001 | carnitine O-acetyltransferase | CRAT | 0,21 | 0,0042 | 0,17 | 0,1427 | 0,25 | 0,1199 |
| 16912742 | BPI fold containing family A, member 2 | BPIFA2 | 0,21 | 0,0007 | -0,06 | 0,3955 | 0,1 | 0,1917 |
| 16678105 | signal recognition particle 9kDa | SRP9 | 0,21 | 0,0146 | 0,14 | 0,0975 | 0,16 | 0,0940 |
| 16914352 | cathepsin A | CTSA | 0,21 | 0,0255 | 0,06 | 0,2430 | 0,06 | 0,4269 |
| 16948493 | ATPase, class VI, type 11B | ATP11B | 0,21 | 0,0433 | 0,10 | 0,0773 | 0,09 | 0,2634 |
| 17015477 | solute carrier family 35, member B3 | SLC35B3 | 0,21 | 0,0363 | 0,11 | 0,2329 | 0,17 | 0,0938 |
| 16865590 | EPS8-like 1 | EPS8L1 | 0,21 | 0,0146 | 0,21 | 0,1039 | 0,1 | 0,4635 |
| 16673315 | pogo transposable element with KRAB domain | POGK | 0,21 | 0,0057 | 0,11 | 0,1291 | 0,2 | 0,0519 |
| 16667247 | down-regulator of transcription 1, TBP-binding (negative cofactor 2) | DR1 | 0,21 | 0,0082 | 0,10 | 0,3040 | 0,1 | 0,3240 |
| 16880538 | EH domain binding protein 1 | EHBP1 | 0,21 | 0,0245 | 0,17 | 0,0742 | 0,18 | 0,2928 |
| 16815855 | C-type lectin domain family 16, member A | CLEC16A | 0,21 | 0,0037 | 0,08 | 0,4718 | 0,1 | 0,3970 |
| 16730503 | Yes-associated protein 1 | YAP1 | 0,21 | 0,0445 | -0,06 | 0,6314 | 0,15 | 0,2117 |
| 17043214 | RB-associated KRAB zinc finger \| uncharacterized LOC389458 \| RBAK-LOC389458 readthrough | RBAK\| LOC389458\| RBAK-LOC389458 | 0,21 | 0,0008 | 0,30 | 0,1056 | 0,21 | 0,0810 |
| 17068636 | protein kinase-like protein SgK196 | SGK196 | 0,21 | 0,0369 | 0,09 | 0,2987 | 0,19 | 0,1187 |
| 17067459 | homeobox containing 1 | HMBOX1 | 0,21 | 0,0107 | 0,10 | 0,2351 | 0,16 | 0,2472 |
| 16918569 | gamma-glutamyltransferase 7 | GGT7 | 0,21 | 0,0250 | 0,17 | 0,3726 | 0,15 | 0,3637 |
| 16995551 | RPTOR independent companion of MTOR, complex 2 | RICTOR | 0,21 | 0,0318 | 0,15 | 0,0640 | 0,17 | 0,0772 |
| 16714998 | DNA replication helicase 2 homolog (yeast) | DNA2 | 0,21 | 0,0014 | 0,05 | 0,1500 | 0,07 | 0,5967 |
| 16871873 | uncharacterized 100631378 | LOC100631378 | 0,21 | 0,0130 | 0,15 | 0,3151 | 0,17 | 0,0543 |
| 17093920 | glucosamine (UDP-N-acetyl)-2-epimerase/N-acetylmannosamine kinase | GNE | 0,21 | 0,0151 | 0,11 | 0,2270 | 0,15 | 0,1186 |
| 16772811 | zinc finger protein 605 | ZNF605 | 0,21 | 0,0079 | 0,00 | 0,9925 | 0,02 | 0,9298 |
| 17095098 | guanine nucleotide binding protein (G protein), alpha 14 | GNA14 | 0,21 | 0,0464 | 0,13 | 0,2469 | 0,37 | 0,1491 |
| 16699458 | RAB3 GTPase activating protein subunit 2 (non-catalytic) \| aurora kinase A pseudogene 1 | RAB3GAP2\| AURKAPS1 | 0,21 | 0,0187 | 0,04 | 0,4912 | 0,14 | 0,1561 |
| 16668333 | glutathione S-transferase mu 4 | GSTM4 | 0,21 | 0,0299 | 0,25 | 0,4442 | 0,34 | 0,1401 |
| 16879408 | echinoderm microtubule associated protein like 4 | EML4 | 0,21 | 0,0309 | 0,07 | 0,3797 | 0,14 | 0,2298 |
| 17020930 | transmembrane protein 30A | TMEM30A | 0,21 | 0,0030 | 0,15 | 0,4116 | 0,18 | 0,0933 |
| 16972082 | translation machinery associated 16 homolog (S. cerevisiae) | TMA16 | 0,21 | 0,0119 | 0,17 | 0,2435 | 0,2 | 0,2085 |
| 16946393 | RAS p21 protein activator 2 | RASA2 | 0,21 | 0,0321 | 0,16 | 0,2285 | 0,19 | 0,1110 |
| 16929006 | solute carrier family 35, member E4 | SLC35E4 | 0,22 | 0,0211 | 0,10 | 0,3663 | 0,15 | 0,1426 |
| 16676520 | solute carrier family 41, member 1 | SLC41A1 | 0,22 | 0,0408 | 0,10 | 0,4869 | 0,02 | 0,8600 |
| 16804631 | chromosome 15 open reading frame 42 | C15orf42 | 0,22 | 0,0329 | 0,20 | 0,0513 | 0,2 | 0,0843 |
| 17089549 | dynamin 1 | DNM1 | 0,22 | 0,0246 | 0,04 | 0,6435 | 0,23 | 0,0928 |
| 16756757 | ubiquitin protein ligase E3B | UBE3B | 0,22 | 0,0192 | 0,00 | 0,9851 | 0,09 | 0,4953 |
| 16991329 | microfibrillar-associated protein 3 | MFAP3 | 0,22 | 0,0463 | 0,15 | 0,0922 | 0,13 | 0,1434 |
| 17009620 | transmembrane protein 14A | TMEM14A | 0,22 | 0,0146 | 0,25 | 0,0807 | 0,16 | 0,2079 |
| 16963340 | UBX domain protein 7 | UBXN7 | 0,22 | 0,0367 | 0,15 | 0,0625 | 0,23 | 0,1030 |
| 17081737 | PTK2 protein tyrosine kinase 2 \| uncharacterized LOC100653146 \| uncharacterized LOC100653024 | PTK2\| LOC100653146\| LOC100653024 | 0,22 | 0,0489 | 0,00 | 0,9964 | 0,18 | 0,2888 |
| 16974498 | F-box and leucine-rich repeat protein 5 | FBXL5 | 0,22 | 0,0454 | 0,12 | 0,1057 | 0,08 | 0,5783 |
| 16807879 | transmembrane protein 87A | TMEM87A | 0,22 | 0,0036 | 0,08 | 0,5892 | 0,16 | 0,1831 |
| 17102017 | membrane-bound transcription factor peptidase, site 2 | MBTPS2 | 0,22 | 0,0088 | 0,06 | 0,6672 | 0,16 | 0,1458 |
| 16993349 | RUN and FYVE domain containing 1 | RUFY1 | 0,22 | 0,0221 | 0,12 | 0,1514 | 0,24 | 0,1839 |
| 16835071 | leucine rich repeat containing 37, member A2 \| leucine rich repeat containing 37A \| leucine rich repeat containing 37, member A3 | LRRC37A2\|LRRC37A3 | 0,22 | 0,0013 | 0,13 | 0,1746 | 0,06 | 0,5456 |
| 16680901 | centrosomal protein 104kDa | CEP104 | 0,22 | 0,0484 | 0,10 | 0,6120 | 0,14 | 0,0561 |
| 17005904 | zinc finger protein 192 | ZNF192 | 0,22 | 0,0009 | 0,23 | 0,0905 | 0,19 | 0,1162 |
| 16735321 | olfactory receptor, family 5, subfamily P, member 3 | OR5P3 | 0,22 | 0,0263 | 0,24 | 0,2867 | 0,14 | 0,1068 |
| 16885913 | cyclin T2 | CCNT2 | 0,22 | 0,0221 | -0,02 | 0,8205 | 0,21 | 0,1236 |
| 17096188 | solute carrier family 35, member D2 | SLC35D2 | 0,22 | 0,0438 | -0,01 | 0,9282 | 0,16 | 0,3631 |
| 16795582 | protein tyrosine phosphatase, non-receptor type 21 | PTPN21 | 0,22 | 0,0275 | 0,09 | 0,6534 | 0,07 | 0,3290 |
| 17021004 | pleckstrin homology domain interacting protein | PHIP | 0,22 | 0,0411 | 0,13 | 0,0986 | 0,24 | 0,1314 |
| 16876440 | acid phosphatase 1, soluble | ACP1 | 0,22 | 0,0336 | 0,04 | 0,2921 | 0,15 | 0,1494 |
| 16687028 | epidermal growth factor receptor pathway substrate 15 | EPS15 | 0,22 | 0,0199 | 0,06 | 0,5085 | 0,2 | 0,3115 |
| 16998059 | arrestin domain containing 3 | ARRDC3 | 0,22 | 0,0379 | 0,11 | 0,7000 | 0,28 | 0,1232 |
| 16700554 | pecanex-like 2 (Drosophila) | PCNXL2 | 0,22 | 0,0103 | 0,10 | 0,3246 | 0,14 | 0,1640 |
| 16866025 | zinc finger protein 548 | ZNF548 | 0,22 | 0,0459 | 0,17 | 0,0797 | 0,16 | 0,1226 |
| 16911943 | 5'-3' exoribonuclease 2 | XRN2 | 0,23 | 0,0108 | 0,12 | 0,3700 | 0,28 | 0,1817 |
| 16978142 | eukaryotic translation initiation factor 4E | EIF4E | 0,23 | 0,0044 | 0,28 | 0,1124 | 0,26 | 0,0911 |
| 17025215 | radial spoke 3 homolog (Chlamydomonas) | RSPH3 | 0,23 | 0,0442 | 0,15 | 0,1830 | 0,09 | 0,1422 |
| 16919295 | chromodomain helicase DNA binding protein 6 | CHD6 | 0,23 | 0,0115 | 0,13 | 0,0610 | 0,26 | 0,0510 |
| 16816813 | eukaryotic elongation factor-2 kinase | EEF2K | 0,23 | 0,0243 | 0,08 | 0,6084 | 0,14 | 0,0586 |
| 16961704 | transducin (beta)-like 1 X-linked receptor 1 | TBL1XR1 | 0,23 | 0,0343 | 0,12 | 0,3364 | 0,23 | 0,0944 |
| 17028680 | ring finger protein 39 | RNF39 | 0,23 | 0,0380 | 0,10 | 0,6086 | 0,17 | 0,1069 |
| 16751570 | eukaryotic translation initiation factor 4B \| uncharacterized LOC100653227 \| eukaryotic translation initiation factor 4B pseudogene 3 \| eukaryotic translation initiation factor 4B pseudogene 6 \| eukaryotic translation initiation factor 4B pseudogene 7 | EIF4B\|LOC100653227\|EIF4BP3\|EIF4BP6\|EIF4BP7 | 0,23 | 0,0306 | 0,17 | 0,2563 | 0,19 | 0,2000 |
| 16990190 | zinc finger, matrin-type 2 | ZMAT2 | 0,23 | 0,0299 | 0,19 | 0,2581 | 0,1 | 0,0920 |
| 16872447 | SERTA domain containing 3 | SERTAD3 | 0,23 | 0,0247 | 0,06 | 0,5771 | 0,26 | 0,0913 |
| 16879118 | coiled-coil domain containing 75 | CCDC75 | 0,23 | 0,0493 | 0,18 | 0,0563 | 0,15 | 0,3180 |
| 16683250 | leucine zipper protein 1 | LUZP1 | 0,23 | 0,0092 | 0,08 | 0,0503 | 0,26 | 0,0539 |
| 16839732 | transient receptor potential cation channel, subfamily V, member 1 \| sedoheptulokinase | TRPV1\|SHPK | 0,23 | 0,0268 | 0,09 | 0,5791 | 0,21 | 0,1001 |
| 16705074 | transcription factor A, mitochondrial | TFAM | 0,23 | 0,0188 | -0,01 | 0,9576 | 0,17 | 0,1785 |
| 16695888 | aldehyde dehydrogenase 9 family, member A1 | ALDH9A1 | 0,23 | 0,0408 | 0,17 | 0,1042 | 0,13 | 0,1890 |
| 16686538 | intracisternal A particle-promoted polypeptide | IPP | 0,23 | 0,0112 | 0,07 | 0,6765 | -0,05 | 0,8624 |
| 16786738 | chromosome 14 open reading frame 118 | C14orf118 | 0,23 | 0,0269 | 0,08 | 0,5141 | 0,07 | 0,5367 |
| 16895434 | additional sex combs like 2 (Drosophila) | ASXL2 | 0,23 | 0,0231 | 0,15 | 0,1680 | 0,1 | 0,4307 |
| 17109716 | ribosomal protein S6 kinase, 90kDa, polypeptide 3 | RPS6KA3 | 0,24 | 0,0309 | 0,30 | 0,0607 | 0,17 | 0,2231 |
| 16756136 | host cell factor C2 | HCFC2 | 0,24 | 0,0409 | 0,12 | 0,3851 | 0,16 | 0,3164 |
| 16685374 | Smad nuclear interacting protein 1 | SNIP1 | 0,24 | 0,0113 | 0,04 | 0,6736 | 0,2 | 0,0554 |
| 16902296 | cytoplasmic linker associated protein 1 | CLASP1 | 0,24 | 0,0081 | 0,12 | 0,4186 | 0,19 | 0,1975 |
| 16886503 | RAP1 interacting factor homolog (yeast) | RIF1 | 0,24 | 0,0483 | 0,25 | 0,2017 | 0,26 | 0,1709 |
| 17080723 | ZHX1-C8ORF76 readthrough \| chromosome 8 open reading frame 76 \| zinc fingers and homeoboxes 1 | ZHX1-C8ORF76\|C8orf76 | 0,24 | 0,0153 | 0,10 | 0,4757 | 0,12 | 0,4753 |
| 16732296 | Cbl proto-oncogene, E3 ubiquitin protein ligase | CBL | 0,24 | 0,0483 | 0,15 | 0,2122 | 0,23 | 0,0846 |
| 16975803 | FRY-like | FRYL | 0,24 | 0,0097 | 0,16 | 0,0577 | 0,2 | 0,1094 |
| 16804942 | furin (paired basic amino acid cleaving enzyme) | FURIN | 0,24 | 0,0328 | 0,15 | 0,2708 | 0,02 | 0,8134 |
| 16786167 | presenilin 1 | PSEN1 | 0,24 | 0,0030 | 0,21 | 0,2469 | 0,23 | 0,0527 |
| 16907843 | LanC lantibiotic synthetase component C-like 1 (bacterial) | LANCL1 | 0,24 | 0,0422 | 0,06 | 0,7507 | 0,12 | 0,5413 |
| 16740969 | cardiotrophin-like cytokine factor 1 | CLCF1 | 0,24 | 0,0491 | 0,10 | 0,1879 | 0,24 | 0,0583 |
| 16974082 | Morf4 family associated protein 1-like 1 | MRFAP1L1 | 0,24 | 0,0240 | 0,22 | 0,1220 | 0,14 | 0,0671 |
| 16810686 | ClpX caseinolytic peptidase X homolog (E. coli) | CLPX | 0,24 | 0,0335 | 0,15 | 0,1309 | 0,05 | 0,6068 |
| 16925057 | synaptojanin 1 | SYNJ1 | 0,24 | 0,0168 | 0,24 | 0,1394 | 0,05 | 0,5399 |
| 17117613 | ankyrin repeat domain 10 \| ANKRD10 intronic transcript 1 (non-protein coding) | ANKRD10\|ANKRD10-IT1 | 0,24 | 0,0192 | 0,32 | 0,1494 | 0,3 | 0,1011 |
| 16819952 | core-binding factor, beta subunit | CBFB | 0,24 | 0,0100 | 0,17 | 0,0897 | 0,19 | 0,0598 |
| 17068642 | heparan-alpha-glucosaminide N-acetyltransferase | HGSNAT | 0,24 | 0,0236 | 0,03 | 0,7320 | 0,15 | 0,0569 |
| 16818630 | phosphorylase kinase, beta | PHKB | 0,24 | 0,0070 | 0,07 | 0,3251 | 0,12 | 0,3112 |
| 16782754 | guanosine monophosphate reductase 2 | GMPR2 | 0,24 | 0,0427 | 0,22 | 0,1056 | 0,25 | 0,0733 |
| 16660527 | zinc finger and BTB domain containing 40 | ZBTB40 | 0,24 | 0,0475 | 0,06 | 0,5624 | 0,14 | 0,1753 |
| 16820556 | syntrophin, beta 2 (dystrophin-associated protein A1, 59kDa, basic component 2) | SNTB2 | 0,24 | 0,0019 | 0,09 | 0,0895 | 0,15 | 0,0591 |
| 16730591 | dynein, cytoplasmic 2, heavy chain 1 | DYNC2H1 | 0,24 | 0,0348 | 0,13 | 0,2210 | 0,17 | 0,0755 |
| 16694506 | chromosome 1 open reading frame 85 | C1orf85 | 0,24 | 0,0048 | 0,11 | 0,3997 | 0,12 | 0,0987 |
| 16696824 | v-abl Abelson murine leukemia viral oncogene homolog 2 | ABL2 | 0,24 | 0,0406 | 0,07 | 0,4261 | 0,11 | 0,3946 |
| 16936408 | mitogen-activated protein kinase 12 | MAPK12 | 0,24 | 0,0496 | 0,23 | 0,0689 | 0,32 | 0,0624 |
| 16900183 | lymphocyte-specific protein 1 pseudogene | LOC654342 | 0,24 | 0,0218 | 0,21 | 0,4745 | 0,17 | 0,2771 |
| 17110193 | mediator complex subunit 14 | MED14 | 0,24 | 0,0289 | 0,06 | 0,1198 | 0,16 | 0,0667 |
| 17068064 | leucine zipper-EF-hand containing transmembrane protein 2 | LETM2 | 0,24 | 0,0098 | 0,13 | 0,4385 | 0,23 | 0,1316 |
| 17048368 | RNA binding motif protein 48 | RBM48 | 0,24 | 0,0230 | 0,20 | 0,3144 | 0,13 | 0,1050 |
| 16711797 | nudix (nucleoside diphosphate linked moiety X)-type motif 5 | NUDT5 | 0,25 | 0,0039 | 0,18 | 0,0970 | 0,18 | 0,1192 |
| 16725846 | secretoglobin, family 2A, member 2 | SCGB2A2 | 0,25 | 0,0404 | 0,23 | 0,0684 | 0,08 | 0,3075 |
| 16704351 | zinc finger protein 22 (KOX 15) | ZNF22 | 0,25 | 0,0243 | 0,12 | 0,5198 | 0,09 | 0,0643 |
| 16950848 | v-raf-1 murine leukemia viral oncogene homolog 1 | RAF1 | 0,25 | 0,0080 | 0,01 | 0,9129 | 0,16 | 0,1492 |
| 17093117 | topoisomerase I binding, arginine/serine-rich, E3 ubiquitin protein ligase | TOPORS | 0,25 | 0,0025 | 0,19 | 0,0882 | 0,24 | 0,1662 |
| 16747184 | CD9 molecule \| uncharacterized LOC100653288 \| uncharacterized LOC100652804 | CD9\| LOC100653288\| LOC100652804 | 0,25 | 0,0286 | 0,14 | 0,1298 | 0,21 | 0,0933 |
| 16896620 | heterogeneous nuclear ribonucleoprotein L-like | HNRPLL | 0,25 | 0,0466 | 0,00 | 0,9644 | 0,19 | 0,1756 |
| 16802720 | ariadne homolog, ubiquitin-conjugating enzyme E2 binding protein, 1 (Drosophila) | ARIH1 | 0,25 | 0,0299 | 0,18 | 0,1737 | 0,16 | 0,0802 |
| 17061881 | dedicator of cytokinesis 4 | DOCK4 | 0,25 | 0,0020 | 0,19 | 0,1794 | 0,21 | 0,0766 |
| 16985720 | general transcription factor IIH, polypeptide 2C \| general transcription factor IIH, polypeptide 2D | GTF2H2C\|GTF2H2D | 0,25 | 0,0177 | 0,28 | 0,1467 | 0,07 | 0,7643 |
| 16984689 | integrin, alpha 2 (CD49B, alpha 2 subunit of VLA-2 receptor) | ITGA2 | 0,25 | 0,0195 | 0,04 | 0,7290 | 0,24 | 0,1239 |
| 16933502 | checkpoint kinase 2 | CHEK2 | 0,25 | 0,0339 | 0,06 | 0,7912 | 0,11 | 0,2440 |
| 16844000 | phosphatidylinositol-5-phosphate 4-kinase, type II, beta | PIP4K2B | 0,25 | 0,0250 | 0,19 | 0,1045 | 0,19 | 0,0656 |
| 16798250 | small nuclear ribonucleoprotein polypeptide N \| uncharacterized LOC100506948 \| small nucleolar RNA, C/D box 116-28 \| small nucleolar RNA, C/D box 115-26 \| small nucleolar RNA, C/D box 115-13 \| small nucleolar RNA, C/D box 115-7 \| small nucleolar RNA, C/D box 107 | SNRPN\| LOC100506948\| SNORD116-28\| SNORD115-26\| SNORD115-13\| SNORD115-7\| SNORD107 | 0,25 | 0,0007 | 0,22 | 0,0698 | 0,08 | 0,2263 |
| 16957525 | spindle and centriole associated protein 1 | SPICE1 | 0,25 | 0,0085 | 0,14 | 0,4371 | 0,12 | 0,4397 |
| 17018425 |  | TAF11 | 0,25 | 0,0045 | 0,20 | 0,2930 | 0,2 | 0,1167 |
| 16795128 | transmembrane emp24 protein transport domain containing 8 | TMED8 | 0,25 | 0,0235 | 0,16 | 0,2527 | 0,17 | 0,4141 |
| 16851486 | laminin, alpha 3 | LAMA3 | 0,25 | 0,0204 | 0,14 | 0,0635 | 0,12 | 0,1734 |
| 16661192 | high mobility group nucleosomal binding domain 2 | HMGN2 | 0,25 | 0,0361 | 0,02 | 0,9006 | 0,12 | 0,5980 |
| 16808101 | zinc finger and SCAN domain containing 29 | ZSCAN29 | 0,25 | 0,0360 | 0,14 | 0,1401 | 0,15 | 0,4703 |
| 17015919 | kinesin family member 13A | KIF13A | 0,25 | 0,0175 | 0,19 | 0,0564 | 0,11 | 0,3555 |
| 17002753 | F-box and WD repeat domain containing 11 | FBXW11 | 0,26 | 0,0052 | 0,13 | 0,1490 | 0,23 | 0,0843 |
| 16665512 | asparagine-linked glycosylation 6, alpha-1,3-glucosyltransferase homolog (S. cerevisiae) | ALG6 | 0,26 | 0,0347 | 0,09 | 0,6251 | 0,24 | 0,1862 |
| 17047707 | putative homeodomain transcription factor 2 | PHTF2 | 0,26 | 0,0091 | 0,19 | 0,1294 | 0,17 | 0,2994 |
| 17042805 | golgi to ER traffic protein 4 homolog (S. cerevisiae) \| Sad1 and UNC84 domain containing 1 | GET4\|SUN1 | 0,26 | 0,0265 | 0,08 | 0,6705 | 0,17 | 0,1723 |
| 16819640 | chromosome 16 open reading frame 57 | C16orf57 | 0,26 | 0,0032 | 0,04 | 0,8292 | 0,14 | 0,2825 |
| 16983200 | membrane-associated ring finger (C3HC4) 6, E3 ubiquitin protein ligase | MARCH6 | 0,26 | 0,0103 | 0,19 | 0,0626 | 0,17 | 0,0816 |
| 16777143 | paraspeckle component 1 \| PSPC1 overlapping transcript 1 (non-protein coding) | PSPC1\| PSPC1-OT1 | 0,26 | 0,0168 | 0,15 | 0,0992 | 0,1 | 0,1708 |
| 16821162 | nudix (nucleoside diphosphate linked moiety X)-type motif 7 | NUDT7 | 0,26 | 0,0092 | 0,00 | 0,9948 | 0,22 | 0,2072 |
| 16695944 | transcriptional adaptor 1 | TADA1 | 0,26 | 0,0096 | 0,21 | 0,1090 | 0,11 | 0,3430 |
| 16919408 | chromosome 20 open reading frame 111 | C20orf111 | 0,26 | 0,0153 | 0,22 | 0,0988 | 0,15 | 0,0783 |
| 16828762 | hydroxysteroid dehydrogenase like 1 | HSDL1 | 0,26 | 0,0340 | 0,20 | 0,1919 | 0,19 | 0,1685 |
| 17007930 | bromodomain and PHD finger containing, 3 | BRPF3 | 0,26 | 0,0077 | 0,08 | 0,5152 | 0,14 | 0,3828 |
| 16906065 |  | CWC22 | 0,26 | 0,0403 | 0,14 | 0,3532 | 0,22 | 0,3082 |
| 16964723 | Morf4 family associated protein 1 | MRFAP1 | 0,26 | 0,0323 | 0,15 | 0,1874 | 0,2 | 0,1854 |
| 16813974 | TM2 domain containing 3 | TM2D3 | 0,26 | 0,0313 | 0,19 | 0,1862 | 0,19 | 0,1709 |
| 16723546 | catalase | CAT | 0,26 | 0,0028 | 0,17 | 0,0806 | 0,06 | 0,1597 |
| 17064603 | myeloid/lymphoid or mixed-lineage leukemia 3 | MLL3 | 0,26 | 0,0390 | 0,15 | 0,0861 | 0,24 | 0,0631 |
| 16781059 | ankyrin repeat domain 10 \| ANKRD10 intronic transcript 1 (non-protein coding) | ANKRD10\| ANKRD10-IT1 | 0,26 | 0,0320 | 0,12 | 0,2204 | 0,23 | 0,0666 |
| 16787994 | T-cell leukemia/lymphoma 1B | TCL1B | 0,26 | 0,0128 | 0,20 | 0,2401 | 0,14 | 0,4211 |
| 16911675 | chromosome 20 open reading frame 72 | C20orf72 | 0,26 | 0,0122 | 0,21 | 0,0727 | 0,24 | 0,0682 |
| 17114368 | motile sperm domain containing 1 | MOSPD1 | 0,26 | 0,0141 | 0,14 | 0,1382 | 0,18 | 0,1261 |
| 16986417 | coagulation factor II (thrombin) receptor-like 1 | F2RL1 | 0,26 | 0,0346 | 0,31 | 0,0581 | 0,13 | 0,1946 |
| 16977340 | heterogeneous nuclear ribonucleoprotein D (AU-rich element RNA binding protein 1, 37kDa) | HNRNPD | 0,26 | 0,0179 | 0,17 | 0,0563 | 0,21 | 0,0959 |
| 17078061 | nuclear receptor coactivator 2 | NCOA2 | 0,26 | 0,0333 | 0,13 | 0,1787 | 0,18 | 0,1342 |
| 16762798 | importin 8 | IPO8 | 0,26 | 0,0115 | 0,03 | 0,7881 | 0,18 | 0,0772 |
| 16998621 | nudix (nucleoside diphosphate linked moiety X)-type motif 12 | NUDT12 | 0,26 | 0,0182 | -0,08 | 0,6576 | 0,2 | 0,1546 |
| 17088511 | tripartite motif containing 32 | TRIM32 | 0,26 | 0,0231 | 0,15 | 0,1078 | 0,14 | 0,5268 |
| 16845574 | ataxin 7-like 3 | ATXN7L3 | 0,26 | 0,0169 | 0,02 | 0,8950 | 0,18 | 0,1152 |
| 16945702 | DnaJ (Hsp40) homolog, subfamily C, member 13 | DNAJC13 | 0,27 | 0,0269 | 0,13 | 0,2348 | 0,14 | 0,0730 |
| 16791669 | HECT domain containing E3 ubiquitin protein ligase 1 | HECTD1 | 0,27 | 0,0180 | 0,08 | 0,5278 | 0,25 | 0,0618 |
| 16954295 | macrophage stimulating 1 receptor (c-met-related tyrosine kinase) | MST1R | 0,27 | 0,0428 | 0,18 | 0,1516 | 0,19 | 0,1173 |
| 16946841 | ring finger protein 13 | RNF13 | 0,27 | 0,0318 | 0,23 | 0,1353 | 0,2 | 0,0825 |
| 16715849 | discs, large homolog 5 (Drosophila) | DLG5 | 0,27 | 0,0150 | 0,10 | 0,3905 | 0,07 | 0,1474 |
| 17004500 | phenylalanyl-tRNA synthetase 2, mitochondrial | FARS2 | 0,27 | 0,0141 | 0,13 | 0,3516 | 0,2 | 0,3753 |
| 16855049 | transcription elongation factor B polypeptide 3C (elongin A3) \| transcription elongation factor B polypeptide 3C-like | TCEB3C\| TCEB3CL\| LOC100506888 | 0,27 | 0,0279 | 0,05 | 0,6566 | 0,06 | 0,5923 |
| 16879923 | mutS homolog 6 (E. coli) | MSH6 | 0,27 | 0,0302 | 0,17 | 0,1181 | 0,25 | 0,0531 |
| 16699435 | 3'(2'), 5'-bisphosphate nucleotidase 1 | BPNT1 | 0,27 | 0,0152 | 0,24 | 0,1598 | 0,09 | 0,3145 |
| 16792381 | MIS18 binding protein 1 | MIS18BP1 | 0,27 | 0,0436 | 0,21 | 0,1751 | 0,21 | 0,1860 |
| 16877987 | microtubule-associated protein, RP/EB family, member 3 | MAPRE3 | 0,27 | 0,0274 | 0,01 | 0,9242 | 0,08 | 0,7666 |
| 16837298 | archaelysin family metallopeptidase 2 | AMZ2 | 0,27 | 0,0254 | 0,19 | 0,0768 | 0,18 | 0,0589 |
| 16756995 | tectonic family member 1 | TCTN1 | 0,27 | 0,0169 | 0,08 | 0,5880 | 0,12 | 0,4225 |
| 16776335 | abhydrolase domain containing 13 | ABHD13 | 0,27 | 0,0087 | 0,01 | 0,8784 | 0,14 | 0,1062 |
| 16676183 | ATPase, Ca++ transporting, plasma membrane 4 | ATP2B4 | 0,27 | 0,0420 | 0,19 | 0,3129 | 0,1 | 0,4567 |
| 17004612 | desmoplakin | DSP | 0,27 | 0,0275 | 0,21 | 0,1034 | 0,08 | 0,6325 |
| 16989534 | SMAD family member 5 | SMAD5 | 0,27 | 0,0454 | 0,20 | 0,0703 | 0,23 | 0,1668 |
| 16903461 | methylmalonic aciduria (cobalamin deficiency) cblD type, with homocystinuria | MMADHC | 0,27 | 0,0011 | 0,15 | 0,4743 | 0,13 | 0,0915 |
| 16819052 | iroquois homeobox 6 | IRX6 | 0,27 | 0,0173 | 0,16 | 0,3919 | 0,29 | 0,0767 |
| 17072489 | tRNA methyltransferase 12 homolog (S. cerevisiae) | TRMT12 | 0,27 | 0,0451 | 0,26 | 0,1093 | 0,17 | 0,5372 |
| 16780859 | ephrin-B2 | EFNB2 | 0,27 | 0,0280 | 0,09 | 0,4484 | 0,13 | 0,0995 |
| 16677748 | melanoma inhibitory activity family, member 3 | MIA3 | 0,27 | 0,0233 | 0,08 | 0,5987 | 0,11 | 0,1797 |
| 17013920 | SR-related CTD-associated factor 8 \| uncharacterized LOC100505519 \| T-cell lymphoma invasion and metastasis 2 | SCAF8\| LOC100505519\| TIAM2 | 0,27 | 0,0270 | 0,13 | 0,2173 | 0,12 | 0,2786 |
| 16915748 | chromosome 20 open reading frame 20 | C20orf20 | 0,27 | 0,0254 | 0,08 | 0,5286 | 0,18 | 0,0729 |
| 16797574 | immunoglobulin heavy variable 4-59 | IGHV4-59 | 0,27 | 0,0297 | 0,12 | 0,5364 | 0,36 | 0,1475 |
| 16710245 | acyl-CoA dehydrogenase, short/branched chain | ACADSB | 0,27 | 0,0120 | 0,34 | 0,2170 | 0,23 | 0,1603 |
| 16853962 | metallophosphoesterase 1 | MPPE1 | 0,27 | 0,0318 | 0,09 | 0,2018 | 0,18 | 0,1802 |
| 16944173 | queuine tRNA-ribosyltransferase domain containing 1 | QTRTD1 | 0,27 | 0,0394 | 0,15 | 0,0634 | 0,2 | 0,0580 |
| 16802251 | SMAD family member 3 | SMAD3 | 0,27 | 0,0238 | 0,06 | 0,4146 | 0,37 | 0,0818 |
| 17099799 | UBA domain containing 1 | UBAC1 | 0,27 | 0,0396 | 0,19 | 0,0871 | 0,19 | 0,0878 |
| 17067613 | UBX domain protein 8 | UBXN8 | 0,27 | 0,0498 | 0,25 | 0,3605 | 0,17 | 0,3783 |
| 16691177 | tripartite motif containing 33 | TRIM33 | 0,27 | 0,0242 | 0,09 | 0,5266 | 0,2 | 0,1102 |
| 17113744 | cullin 4B | CUL4B | 0,27 | 0,0278 | 0,19 | 0,2374 | 0,14 | 0,2111 |
| 16705247 | nuclear receptor binding factor 2 | NRBF2 | 0,27 | 0,0163 | -0,06 | 0,8110 | 0,16 | 0,4061 |
| 16847398 | amyloid beta precursor protein (cytoplasmic tail) binding protein 2 | APPBP2 | 0,27 | 0,0337 | 0,05 | 0,4410 | 0,21 | 0,2733 |
| 17058152 | endogenous retrovirus group 3, member 1 \| zinc finger protein 117 | ERV3-1\| ZNF117 | 0,28 | 0,0092 | 0,31 | 0,0976 | 0,22 | 0,0613 |
| 16885874 | mannosyl (alpha-1,6-)-glycoprotein beta-1,6-N-acetyl-glucosaminyltransferase | MGAT5 | 0,28 | 0,0032 | 0,24 | 0,0872 | 0,13 | 0,5561 |
| 16765513 | chromobox homolog 5 | CBX5 | 0,28 | 0,0278 | 0,22 | 0,0868 | 0,21 | 0,1874 |
| 16812738 | hepatoma-derived growth factor, related protein 3 | HDGFRP3 | 0,28 | 0,0312 | 0,01 | 0,8703 | 0,36 | 0,0665 |
| 16936432 | mitogen-activated protein kinase 11 | MAPK11 | 0,28 | 0,0446 | 0,09 | 0,5459 | 0,18 | 0,2438 |
| 16839913 | zinc finger, ZZ-type with EF-hand domain 1 | ZZEF1 | 0,28 | 0,0410 | -0,03 | 0,7767 | 0,18 | 0,1050 |
| 17092252 | RAN binding protein 6 | RANBP6 | 0,28 | 0,0263 | 0,20 | 0,0822 | 0,17 | 0,3740 |
| 16715529 | ubiquitin specific peptidase 54 | USP54 | 0,28 | 0,0203 | 0,13 | 0,4100 | 0,18 | 0,1468 |
| 16888367 | DnaJ (Hsp40) homolog, subfamily C, member 10 | DNAJC10 | 0,28 | 0,0226 | 0,20 | 0,1317 | 0,1 | 0,2889 |
| 17005871 | histone cluster 1, H2bn | HIST1H2BN | 0,28 | 0,0342 | 0,22 | 0,0656 | 0,24 | 0,1681 |
| 16761495 | taste receptor, type 2, member 10 | TAS2R10 | 0,28 | 0,0020 | 0,29 | 0,2004 | 0,2 | 0,2389 |
| 16793532 | reticulon 1 | RTN1 | 0,29 | 0,0001 | 0,05 | 0,7094 | 0,22 | 0,0696 |
| 16971758 | tryptophan 2,3-dioxygenase | TDO2 | 0,29 | 0,0219 | 0,08 | 0,4514 | 0,26 | 0,0502 |
| 16885625 | uncharacterized LOC100216479 \| putative fatty acyl-CoA reductase 2-like protein FLJ43933-like | LOC100216479\| LOC100288897 | 0,29 | 0,0241 | 0,25 | 0,6744 | -0,02 | 0,9428 |
| 16774727 | fibronectin type III domain containing 3A | FNDC3A | 0,29 | 0,0090 | 0,07 | 0,6014 | 0,27 | 0,1054 |
| 16877007 | Kruppel-like factor 11 | KLF11 | 0,29 | 0,0280 | 0,28 | 0,0664 | 0,18 | 0,0718 |
| 16792754 | mitogen-activated protein kinase kinase kinase kinase 5 | MAP4K5 | 0,29 | 0,0234 | 0,17 | 0,3616 | 0,22 | 0,1093 |
| 16819666 | coiled-coil domain containing 113 | CCDC113 | 0,29 | 0,0082 | 0,03 | 0,7991 | 0,22 | 0,0921 |
| 16801283 | phosphatidylinositol glycan anchor biosynthesis, class B | PIGB | 0,29 | 0,0082 | -0,03 | 0,8373 | 0,14 | 0,5138 |
| 16772704 | ankyrin repeat and LEM domain containing 2 | ANKLE2 | 0,29 | 0,0177 | 0,13 | 0,2490 | 0,19 | 0,3267 |
| 17080342 | trichorhinophalangeal syndrome I | TRPS1 | 0,29 | 0,0339 | 0,19 | 0,1302 | 0,23 | 0,0974 |
| 16687638 | ubiquitin specific peptidase 24 | USP24 | 0,29 | 0,0149 | 0,17 | 0,2038 | 0,26 | 0,0531 |
| 17019254 | chromosome 6 open reading frame 226 | C6orf226 | 0,29 | 0,0389 | 0,13 | 0,3438 | 0,1 | 0,6555 |
| 16671115 | small proline-rich protein 1B | SPRR1B | 0,29 | 0,0174 | 0,06 | 0,7261 | 0,07 | 0,7187 |
| 17047176 | general transcription factor IIi | GTF2I | 0,29 | 0,0025 | 0,19 | 0,0765 | 0,29 | 0,1594 |
| 16977912 | phosphatidylinositol glycan anchor biosynthesis, class Y | PIGY | 0,29 | 0,0250 | 0,34 | 0,2927 | 0,23 | 0,1725 |
| 16763257 | YY1 associated factor 2 | YAF2 | 0,29 | 0,0287 | 0,25 | 0,1158 | 0,21 | 0,1092 |
| 16911338 | phospholipase C, beta 4 | PLCB4 | 0,29 | 0,0138 | 0,19 | 0,0852 | 0,2 | 0,1396 |
| 16789526 | zinc finger and BTB domain containing 42 | ZBTB42 | 0,29 | 0,0131 | 0,05 | 0,8431 | 0,14 | 0,4247 |
| 16843429 | schlafen family member 13 | SLFN13 | 0,30 | 0,0196 | 0,20 | 0,1443 | 0,16 | 0,1059 |
| 17009930 | PHD finger protein 3 | PHF3 | 0,30 | 0,0242 | 0,27 | 0,0915 | 0,24 | 0,1029 |
| 17058176 | glucuronidase, beta | GUSB | 0,30 | 0,0252 | 0,22 | 0,0770 | 0,21 | 0,0892 |
| 16983286 | triple functional domain (PTPRF interacting) | TRIO | 0,30 | 0,0097 | 0,09 | 0,3792 | 0,22 | 0,1052 |
| 16708802 |  | TAF5 | 0,30 | 0,0072 | 0,22 | 0,0876 | 0,36 | 0,0987 |
| 16659251 | vacuolar protein sorting 13 homolog D (S. cerevisiae) | VPS13D | 0,30 | 0,0413 | 0,13 | 0,5006 | 0,24 | 0,1628 |
| 16974900 | coiled-coil domain containing 149 | CCDC149 | 0,30 | 0,0311 | 0,04 | 0,7204 | 0,11 | 0,2158 |
| 17005790 | histone cluster 2, H4b \| histone cluster 4, H4 \| histone cluster 2, H4a \| histone cluster 1, H4l \| histone cluster 1, H4e \| histone cluster 1, H4b \| histone cluster 1, H4h \| histone cluster 1, H4c \| histone cluster 1, H4j \| histone cluster 1, H4k \| histone cluster 1, H4f \| histone cluster 1, H4d \| histone cluster 1, H4a \| histone cluster 1, H4i | HIST2H4B\| HIST4H4\| HIST2H4A\| HIST1H4L\| HIST1H4E\| HIST1H4B\| HIST1H4H\| HIST1H4C\| HIST1H4J\| HIST1H4K\| HIST1H4F\| HIST1H4D\| HIST1H4A\| HIST1H4I | 0,30 | 0,0265 | 0,12 | 0,2285 | 0,05 | 0,6372 |
| 16854558 | trafficking protein particle complex 8 | TRAPPC8 | 0,30 | 0,0478 | 0,13 | 0,4308 | 0,2 | 0,0980 |
| 16688441 | ankyrin repeat domain 13C | ANKRD13C | 0,30 | 0,0247 | 0,17 | 0,2451 | 0,27 | 0,1875 |
| 16957554 |  | KIAA2018 | 0,30 | 0,0291 | 0,11 | 0,2530 | 0,19 | 0,1018 |
| 16830016 | kinesin family member 1C | KIF1C | 0,30 | 0,0004 | 0,11 | 0,2803 | 0,21 | 0,1474 |
| 16904917 | FAST kinase domains 1 | FASTKD1 | 0,30 | 0,0458 | 0,20 | 0,3869 | 0,18 | 0,2287 |
| 16780358 | UDP-glucose glycoprotein glucosyltransferase 2 | UGGT2 | 0,30 | 0,0177 | 0,22 | 0,1232 | 0,23 | 0,1110 |
| 16677361 | ribosomal protein S6 kinase, 52kDa, polypeptide 1 | RPS6KC1 | 0,30 | 0,0333 | 0,12 | 0,5214 | 0,19 | 0,0786 |
| 16924151 | myeloid/lymphoid or mixed-lineage leukemia 3 \| transmembrane phosphatase with tensin homology | MLL3\|TPTE | 0,30 | 0,0302 | 0,19 | 0,1819 | 0,28 | 0,1477 |
| 16970096 | methyltransferase like 14 | METTL14 | 0,30 | 0,0178 | 0,24 | 0,0645 | 0,24 | 0,1558 |
| 16785821 | solute carrier family 39 (zinc transporter), member 9 | SLC39A9 | 0,30 | 0,0388 | 0,12 | 0,3352 | 0,09 | 0,4181 |
| 16832658 | TAO kinase 1 | TAOK1 | 0,30 | 0,0294 | 0,19 | 0,1722 | 0,13 | 0,1134 |
| 17068435 | adaptor-related protein complex 3, mu 2 subunit | AP3M2 | 0,31 | 0,0193 | 0,24 | 0,1289 | 0,22 | 0,2080 |
| 16795304 | centrosomal protein 128kDa | CEP128 | 0,31 | 0,0297 | 0,15 | 0,1653 | 0,26 | 0,2185 |
| 16808940 | SECIS binding protein 2-like | SECISBP2L | 0,31 | 0,0487 | 0,13 | 0,5107 | 0,27 | 0,0987 |
| 16752244 | diacylglycerol kinase, alpha 80kDa | DGKA | 0,31 | 0,0237 | 0,17 | 0,3052 | 0,08 | 0,2445 |
| 16741496 | SH3 and multiple ankyrin repeat domains 2 | SHANK2 | 0,31 | 0,0041 | 0,04 | 0,5960 | 0,28 | 0,0601 |
| 16834196 | FK506 binding protein 10, 65 kDa | FKBP10 | 0,31 | 0,0244 | 0,22 | 0,0687 | 0,15 | 0,2148 |
| 16700058 | inositol-trisphosphate 3-kinase B | ITPKB | 0,31 | 0,0421 | -0,04 | 0,7655 | 0,16 | 0,1764 |
| 16669087 | solute carrier family 22, member 15 | SLC22A15 | 0,31 | 0,0292 | 0,18 | 0,0896 | 0,19 | 0,0870 |
| 16866232 | zinc finger protein 544 | ZNF544 | 0,31 | 0,0009 | 0,01 | 0,9574 | 0,22 | 0,3298 |
| 16832255 | dehydrogenase/reductase (SDR family) member 7B | DHRS7B | 0,31 | 0,0068 | 0,18 | 0,1238 | 0,18 | 0,3052 |
| 16934140 | proline rich 14-like | PRR14L | 0,31 | 0,0085 | -0,10 | 0,6218 | 0,19 | 0,0715 |
| 16789209 | MAP/microtubule affinity-regulating kinase 3 | MARK3 | 0,31 | 0,0118 | 0,23 | 0,1204 | 0,21 | 0,0833 |
| 16744991 | myelin protein zero-like 3 | MPZL3 | 0,31 | 0,0182 | 0,06 | 0,4674 | 0,12 | 0,4866 |
| 16846448 | speckle-type POZ protein | SPOP | 0,31 | 0,0050 | 0,25 | 0,1792 | 0,22 | 0,1807 |
| 16705439 | storkhead box 1 | STOX1 | 0,31 | 0,0154 | 0,18 | 0,4382 | 0,19 | 0,0639 |
| 16678247 | zinc finger protein 678 | ZNF678 | 0,31 | 0,0233 | 0,24 | 0,2894 | 0,32 | 0,2461 |
| 16863922 | BCL2-associated X protein | BAX | 0,31 | 0,0161 | 0,16 | 0,1551 | 0,13 | 0,2377 |
| 16855026 | protein inhibitor of activated STAT, 2 | PIAS2 | 0,32 | 0,0342 | 0,28 | 0,0968 | 0,24 | 0,0827 |
| 17079530 | cytochrome c oxidase subunit VIc | COX6C | 0,32 | 0,0046 | 0,30 | 0,1150 | 0,2 | 0,1383 |
| 16819252 | metallothionein 1F | MT1F | 0,32 | 0,0371 | 0,13 | 0,1083 | 0,21 | 0,1858 |
| 16766093 | signal transducer and activator of transcription 2, 113kDa | STAT2 | 0,32 | 0,0411 | 0,06 | 0,5916 | 0,24 | 0,1121 |
| 16939863 | chromosome 3 open reading frame 23 | C3orf23 | 0,32 | 0,0320 | 0,23 | 0,3314 | 0,19 | 0,0501 |
| 16802653 | thrombospondin, type I, domain containing 4 | THSD4 | 0,32 | 0,0014 | 0,07 | 0,3479 | 0,16 | 0,1776 |
| 16870174 | anoctamin 8 | ANO8 | 0,32 | 0,0277 | 0,18 | 0,2915 | 0,21 | 0,2109 |
| 16836799 | tousled-like kinase 2 | TLK2 | 0,32 | 0,0180 | 0,20 | 0,1567 | 0,25 | 0,1353 |
| 16925158 | transmembrane protein 50B | TMEM50B | 0,32 | 0,0006 | 0,18 | 0,1093 | 0,21 | 0,0757 |
| 16988576 | casein kinase 1, gamma 3 | CSNK1G3 | 0,32 | 0,0097 | 0,17 | 0,0629 | 0,19 | 0,2170 |
| 17044302 | coiled-coil domain containing 126 | CCDC126 | 0,32 | 0,0170 | 0,05 | 0,7952 | 0,16 | 0,4130 |
| 16971019 | GRB2-associated binding protein 1 | GAB1 | 0,32 | 0,0314 | 0,19 | 0,2969 | 0,3 | 0,1236 |
| 16990199 | vault RNA 1-1 | VTRNA1-1 | 0,32 | 0,0395 | 0,04 | 0,9262 | 0,13 | 0,6522 |
| 16972912 | solute carrier family 25 (mitochondrial carrier; adenine nucleotide translocator), member 4 | SLC25A4 | 0,32 | 0,0420 | 0,12 | 0,2048 | 0,1 | 0,4227 |
| 16730792 | cullin 5 | CUL5 | 0,33 | 0,0348 | 0,16 | 0,3449 | 0,2 | 0,1897 |
| 17026012 | programmed cell death 2 | PDCD2 | 0,33 | 0,0239 | 0,16 | 0,2953 | 0,29 | 0,1028 |
| 16738736 | protein associated with topoisomerase II homolog 1 (yeast) | PATL1 | 0,33 | 0,0296 | 0,18 | 0,0671 | 0,29 | 0,0864 |
| 16717522 | biogenesis of lysosomal organelles complex-1, subunit 2 | BLOC1S2 | 0,33 | 0,0111 | 0,20 | 0,1333 | 0,27 | 0,2327 |
| 16985162 | importin 11 \| kinesin heavy chain member 2A | IPO11\|KIF2A | 0,33 | 0,0160 | 0,23 | 0,0517 | 0,31 | 0,1005 |
| 16811994 | NKF3 kinase family member | PEAK1 | 0,33 | 0,0226 | 0,17 | 0,1479 | 0,21 | 0,1888 |
| 17114829 | iduronate 2-sulfatase | IDS | 0,33 | 0,0208 | 0,12 | 0,2698 | 0,21 | 0,2052 |
| 16680420 | solute carrier family 35, member E2B | SLC35E2B | 0,33 | 0,0262 | 0,15 | 0,0811 | 0,22 | 0,1843 |
| 16870925 | zinc finger protein 724, pseudogene | ZNF724P | 0,33 | 0,0136 | 0,32 | 0,2245 | 0,27 | 0,5271 |
| 16935703 | ADP-ribosylation factor GTPase activating protein 3 | ARFGAP3 | 0,33 | 0,0218 | 0,26 | 0,1758 | 0,32 | 0,1149 |
| 16729585 |  | PCF11 | 0,33 | 0,0406 | 0,25 | 0,0907 | 0,29 | 0,0612 |
| 16688487 | zinc finger, RAN-binding domain containing 2 | ZRANB2 | 0,33 | 0,0209 | 0,31 | 0,0918 | 0,2 | 0,0765 |
| 16721861 | SWAP switching B-cell complex 70kDa subunit | SWAP70 | 0,34 | 0,0420 | 0,23 | 0,2723 | 0,27 | 0,0527 |
| 16967557 |  | UTP3 | 0,34 | 0,0476 | 0,45 | 0,0531 | 0,47 | 0,1194 |
| 16666835 | SH3-domain GRB2-like endophilin B1 | SH3GLB1 | 0,34 | 0,0134 | 0,18 | 0,0985 | 0,26 | 0,0965 |
| 16939796 | SNF related kinase | SNRK | 0,34 | 0,0212 | 0,22 | 0,1563 | 0,29 | 0,0721 |
| 16792287 | F-box protein 33 | FBXO33 | 0,34 | 0,0238 | 0,14 | 0,0619 | 0,18 | 0,3495 |
| 16863593 | G protein-coupled receptor 77 | GPR77 | 0,34 | 0,0495 | 0,40 | 0,1630 | 0 | 0,9908 |
| 17085055 | DDB1 and CUL4 associated factor 10 | DCAF10 | 0,34 | 0,0028 | 0,24 | 0,1246 | 0,3 | 0,1167 |
| 16836404 | dynein, light chain, LC8-type 2 | DYNLL2 | 0,34 | 0,0294 | 0,14 | 0,0534 | 0,22 | 0,1444 |
| 16850865 | ankyrin repeat domain 12 | ANKRD12 | 0,34 | 0,0400 | 0,25 | 0,0786 | 0,25 | 0,1005 |
| 16853625 | zinc finger protein 161 homolog (mouse) | ZFP161 | 0,34 | 0,0167 | 0,05 | 0,6582 | 0,11 | 0,4077 |
| 16710481 | dedicator of cytokinesis 1 | DOCK1 | 0,35 | 0,0315 | 0,15 | 0,3763 | 0,27 | 0,0943 |
| 17011635 | cell division cycle 40 homolog (S. cerevisiae) | CDC40 | 0,35 | 0,0325 | 0,27 | 0,1105 | 0,23 | 0,1438 |
| 17058460 | bromodomain adjacent to zinc finger domain, 1B | BAZ1B | 0,35 | 0,0304 | 0,20 | 0,0766 | 0,28 | 0,0832 |
| 17010522 | interleukin-1 receptor-associated kinase 1 binding protein 1 | IRAK1BP1 | 0,35 | 0,0198 | 0,24 | 0,2186 | 0,3 | 0,0641 |
| 16896346 | fasciculation and elongation protein zeta 2 (zygin II) | FEZ2 | 0,35 | 0,0184 | 0,12 | 0,1355 | 0,32 | 0,0664 |
| 16952797 | solute carrier family 6 (proline IMINO transporter), member 20 | SLC6A20 | 0,35 | 0,0001 | 0,17 | 0,0501 | 0,23 | 0,0592 |
| 16729188 | UV radiation resistance associated gene | UVRAG | 0,35 | 0,0167 | 0,17 | 0,3545 | 0,27 | 0,1424 |
| 16854185 | establishment of cohesion 1 homolog 1 (S. cerevisiae) | ESCO1 | 0,35 | 0,0191 | 0,23 | 0,0818 | 0,21 | 0,1369 |
| 17110289 | monoamine oxidase B | MAOB | 0,35 | 0,0270 | 0,34 | 0,0835 | 0,19 | 0,2585 |
| 16800991 | ubiquitin specific peptidase 8 | USP8 | 0,36 | 0,0117 | 0,13 | 0,1234 | 0,26 | 0,1123 |
| 16907173 | origin recognition complex, subunit 2 | ORC2 | 0,36 | 0,0180 | 0,28 | 0,1249 | 0,24 | 0,2422 |
| 16735054 | hemopexin | HPX | 0,36 | 0,0062 | 0,25 | 0,0691 | 0,19 | 0,0789 |
| 16708719 | tripartite motif containing 8 | TRIM8 | 0,36 | 0,0009 | -0,03 | 0,8293 | 0,2 | 0,0751 |
| 16884956 | STEAP family member 3, metalloreductase | STEAP3 | 0,36 | 0,0219 | 0,41 | 0,0544 | 0,29 | 0,1034 |
| 16848784 | MIF4G domain containing | MIF4GD | 0,36 | 0,0459 | 0,07 | 0,6381 | 0,25 | 0,1959 |
| 16689352 | guanylate binding protein 2, interferon-inducible | GBP2 | 0,36 | 0,0269 | 0,31 | 0,0884 | 0,21 | 0,0899 |
| 16664802 | sterol carrier protein 2 | SCP2 | 0,36 | 0,0248 | 0,23 | 0,0687 | 0,18 | 0,0743 |
| 16856834 | zinc finger protein 555 | ZNF555 | 0,36 | 0,0159 | -0,03 | 0,9098 | 0,18 | 0,4841 |
| 17023150 | mannosidase, alpha, class 1A, member 1 | MAN1A1 | 0,36 | 0,0120 | 0,28 | 0,0971 | 0,24 | 0,2783 |
| 16761518 | taste receptor, type 2, member 31 \| taste receptor, type 2, member 45 | TAS2R31\| TAS2R45 | 0,36 | 0,0240 | 0,39 | 0,1982 | 0,21 | 0,1686 |
| 16741864 | FCH and double SH3 domains 2 | FCHSD2 | 0,36 | 0,0459 | 0,24 | 0,1632 | 0,31 | 0,1095 |
| 16870802 | zinc finger protein 708 | ZNF708 | 0,37 | 0,0227 | 0,12 | 0,6723 | 0,08 | 0,6684 |
| 16761583 | low density lipoprotein receptor-related protein 6 | LRP6 | 0,37 | 0,0390 | 0,24 | 0,2080 | 0,23 | 0,1437 |
| 16723858 | tetratricopeptide repeat domain 17 | TTC17 | 0,37 | 0,0037 | 0,12 | 0,1919 | 0,23 | 0,1141 |
| 17112918 | brain expressed, X-linked 1 | BEX1 | 0,37 | 0,0406 | 0,21 | 0,0844 | -0,06 | 0,7279 |
| 16898002 | ubiquitin specific peptidase 34 | USP34 | 0,37 | 0,0205 | 0,24 | 0,1047 | 0,3 | 0,0691 |
| 16694188 | ash1 (absent, small, or homeotic)-like (Drosophila) \| microRNA 555 | ASH1L\|MIR555 | 0,37 | 0,0471 | 0,21 | 0,0602 | 0,23 | 0,1242 |
| 17101677 | motile sperm domain containing 2 | MOSPD2 | 0,38 | 0,0399 | 0,18 | 0,3246 | 0,28 | 0,1554 |
| 16687829 | OMA1 zinc metallopeptidase homolog (S. cerevisiae) \| disabled homolog 1 (Drosophila) | OMA1\|DAB1 | 0,38 | 0,0340 | 0,12 | 0,3192 | 0,1 | 0,4735 |
| 16884441 | zinc finger CCCH-type containing 6 | ZC3H6 | 0,38 | 0,0012 | 0,26 | 0,0533 | 0,18 | 0,1654 |
| 16820601 | nuclear factor of activated T-cells 5, tonicity-responsive | NFAT5 | 0,38 | 0,0251 | 0,17 | 0,5585 | 0,27 | 0,1214 |
| 16902147 | coiled-coil domain containing 93 | CCDC93 | 0,38 | 0,0066 | 0,33 | 0,2066 | 0,3 | 0,0986 |
| 16861282 | amyloid beta (A4) precursor-like protein 1 | APLP1 | 0,39 | 0,0136 | 0,21 | 0,3339 | 0,31 | 0,1209 |
| 16740630 | FOS-like antigen 1 | FOSL1 | 0,39 | 0,0123 | 0,18 | 0,3193 | 0,24 | 0,2520 |
| 16961475 | eukaryotic translation initiation factor 5A2 | EIF5A2 | 0,39 | 0,0096 | 0,13 | 0,6021 | 0,16 | 0,1290 |
| 16956027 | TATA element modulatory factor 1 | TMF1 | 0,39 | 0,0217 | 0,11 | 0,3108 | 0,28 | 0,2315 |
| 17083975 | methylthioadenosine phosphorylase | MTAP | 0,39 | 0,0360 | 0,18 | 0,1622 | 0,38 | 0,0684 |
| 17071625 | frizzled family receptor 6 | FZD6 | 0,39 | 0,0422 | 0,25 | 0,0643 | 0,2 | 0,1997 |
| 16722217 | aryl hydrocarbon receptor nuclear translocator-like | ARNTL | 0,40 | 0,0150 | 0,29 | 0,0820 | 0,36 | 0,0670 |
| 16815304 | uncharacterized LOC100507378 \| long intergenic non-protein coding RNA 514 | LOC100507378\| LINC00514 | 0,40 | 0,0022 | 0,37 | 0,1748 | 0,23 | 0,1889 |
| 17013597 | chromosome 6 open reading frame 72 | C6orf72 | 0,40 | 0,0208 | 0,22 | 0,0849 | 0,33 | 0,1230 |
| 16786304 | prostaglandin reductase 2 | PTGR2 | 0,40 | 0,0358 | 0,18 | 0,1319 | 0,23 | 0,1280 |
| 17087836 | fukutin | FKTN | 0,40 | 0,0176 | 0,14 | 0,2246 | 0,31 | 0,0543 |
| 16714381 | cleavage stimulation factor, 3' pre-RNA, subunit 2, 64kDa, tau variant | CSTF2T | 0,40 | 0,0475 | 0,31 | 0,1222 | 0,31 | 0,0605 |
| 16796128 | BTB (POZ) domain containing 7 | BTBD7 | 0,41 | 0,0463 | 0,03 | 0,8951 | 0,17 | 0,2064 |
| 16793052 | glucosamine-phosphate N-acetyltransferase 1 | GNPNAT1 | 0,41 | 0,0346 | 0,34 | 0,1081 | 0,25 | 0,1034 |
| 16875599 | troponin T type 1 (skeletal, slow) | TNNT1 | 0,41 | 0,0028 | 0,18 | 0,1318 | 0,27 | 0,2046 |
| 16985304 | chromosome 5 open reading frame 44 | C5orf44 | 0,41 | 0,0486 | 0,24 | 0,3004 | 0,31 | 0,1351 |
| 16946120 | armadillo repeat containing 8 | ARMC8 | 0,41 | 0,0221 | 0,12 | 0,2676 | 0,24 | 0,0871 |
| 16986539 | secretory carrier membrane protein 1 | SCAMP1 | 0,41 | 0,0389 | 0,21 | 0,4165 | 0,24 | 0,0838 |
| 16907303 | transmembrane protein 237 | TMEM237 | 0,41 | 0,0428 | 0,10 | 0,7005 | 0,13 | 0,0579 |
| 17005077 | myosin regulatory light chain interacting protein | MYLIP | 0,42 | 0,0434 | 0,15 | 0,1779 | 0,21 | 0,0791 |
| 16942060 | sarcolemma associated protein \| uncharacterized LOC100287789 | SLMAP\| LOC100287789 | 0,43 | 0,0176 | 0,32 | 0,2630 | 0,32 | 0,0830 |
| 16798240 | small nuclear ribonucleoprotein polypeptide N \| uncharacterized LOC100506948 \| small nucleolar RNA, C/D box 116-28 \| small nucleolar RNA, C/D box 115-26 \| small nucleolar RNA, C/D box 115-13 \| small nucleolar RNA, C/D box 115-7 \| small nucleolar RNA, C/D box 107 | SNRPN\| LOC100506948\| SNORD116-28\| SNORD115-26\| SNORD115-13\| SNORD115-7\| SNORD107 | 0,43 | 0,0046 | 0,28 | 0,1174 | 0,2 | 0,0852 |
| 16907546 | glycine cleavage system protein H (aminomethyl carrier) pseudogene 3 | GCSHP3 | 0,44 | 0,0324 | 0,14 | 0,2673 | 0,29 | 0,2353 |
| 16876043 | zinc finger protein 417 | ZNF417 | 0,44 | 0,0071 | 0,14 | 0,3158 | 0,25 | 0,0640 |
| 16917689 | Ral GTPase activating protein, alpha subunit 2 (catalytic) | RALGAPA2 | 0,44 | 0,0230 | 0,44 | 0,0552 | 0,28 | 0,1238 |
| 16811930 |  | TYRO3P | 0,44 | 0,0347 | 0,32 | 0,0502 | 0,37 | 0,4238 |
| 16952672 | anoctamin 10 | ANO10 | 0,45 | 0,0232 | 0,21 | 0,0555 | 0,26 | 0,0971 |
| 16668079 | G-protein signaling modulator 2 | GPSM2 | 0,45 | 0,0067 | 0,22 | 0,2817 | 0,26 | 0,0948 |
| 17112253 | tetratricopeptide repeat domain 3 pseudogene 1 | TTC3P1 | 0,46 | 0,0134 | 0,31 | 0,2149 | 0,38 | 0,0645 |
| 16851353 | mindbomb E3 ubiquitin protein ligase 1 | MIB1 | 0,47 | 0,0141 | 0,21 | 0,1208 | 0,33 | 0,0958 |
| 16984492 | nicotinamide nucleotide transhydrogenase | NNT | 0,47 | 0,0148 | 0,24 | 0,1724 | 0,34 | 0,0549 |
| 16780015 | RNA binding motif protein 26 | RBM26 | 0,48 | 0,0458 | 0,22 | 0,2774 | 0,42 | 0,1415 |
| 17066183 | N-acetyltransferase 1 (arylamine N-acetyltransferase) | NAT1 | 0,48 | 0,0486 | 0,41 | 0,0811 | 0,2 | 0,2924 |
| 16820439 | zinc finger protein 90 homolog (mouse) | ZFP90 | 0,48 | 0,0322 | 0,08 | 0,7576 | 0,35 | 0,1263 |
| 16863684 | selenoprotein W, 1 | SEPW1 | 0,49 | 0,0290 | 0,36 | 0,0650 | 0,32 | 0,2250 |
| 17094224 | zinc finger protein 658B, pseudogene \| zinc finger protein 658 \| zinc finger protein 658 pseudogene \| zinc finger protein 658-like | ZNF658B\| LOC653501\| LOC100653002 | 0,49 | 0,0109 | 0,28 | 0,0611 | 0,42 | 0,0757 |
| 16961399 | leucine rich repeat containing 31 | LRRC31 | 0,49 | 0,0113 | 0,40 | 0,2614 | 0,17 | 0,2658 |
| 16818927 | chromodomain helicase DNA binding protein 9 | CHD9 | 0,50 | 0,0085 | 0,23 | 0,1131 | 0,42 | 0,0503 |
| 17107118 | zinc finger protein 449 | ZNF449 | 0,51 | 0,0054 | 0,27 | 0,4008 | 0,36 | 0,0543 |
| 17112376 | bromodomain and WD repeat domain containing 3 | BRWD3 | 0,51 | 0,0136 | 0,32 | 0,0952 | 0,34 | 0,0603 |
| 16956448 | glucan (1,4-alpha-), branching enzyme 1 | GBE1 | 0,52 | 0,0357 | 0,46 | 0,0651 | 0,36 | 0,0761 |
| 16682259 | ciliary rootlet coiled-coil, rootletin pseudogene 2 | CROCCP2 | 0,52 | 0,0371 | 0,14 | 0,4088 | 0,4 | 0,1729 |
| 16965162 | cytoplasmic polyadenylation element binding protein 2 | CPEB2 | 0,53 | 0,0071 | 0,25 | 0,1855 | 0,32 | 0,0651 |
| 16861500 | zinc finger protein 420 | ZNF420 | 0,54 | 0,0268 | 0,41 | 0,1636 | 0,33 | 0,1229 |
| 16851230 | ankyrin repeat domain 20 family, member A5, pseudogene | ANKRD20A5P | 0,54 | 0,0479 | 0,32 | 0,2253 | 0,07 | 0,7690 |
| 16689474 | zinc finger protein 644 | ZNF644 | 0,54 | 0,0227 | 0,38 | 0,1119 | 0,38 | 0,0785 |
| 16873645 | small ILF3/NF90-associated RNA B1 \| small ILF3/NF90-associated RNA B2 \| small ILF3/NF90-associated RNA C1 \| small ILF3/NF90-associated RNA C2 \| small ILF3/NF90-associated RNA C3 \| small ILF3/NF90-associated RNA C4 \| small ILF3/NF90-associated RNA C5 \| small ILF3/NF90-associated RNA E \| melanoma cell adhesion molecule | SNAR-B1\| SNAR-B2\| SNAR-C1\| SNAR-C2 \|SNAR-C3\| SNAR-C4\| SNAR-C5\| SNAR-E\| MCAM | 0,55 | 0,0339 | 0,54 | 0,1281 | 0,42 | 0,0744 |
| 17107919 | zinc finger protein 185 (LIM domain) | ZNF185 | 0,55 | 0,0075 | 0,52 | 0,0681 | 0,19 | 0,2156 |
| 17046470 | zinc finger protein 107 | ZNF107 | 0,56 | 0,0468 | 0,37 | 0,2340 | 0,34 | 0,0547 |
| 16970176 | uncharacterized LOC645513 \| uncharacterized LOC100507322 | LOC645513\| LOC100507322 | 0,62 | 0,0068 | 0,27 | 0,0910 | 0,44 | 0,0901 |
| 16684391 | Na+/K+ transporting ATPase interacting 1 | NKAIN1 | 0,65 | 0,0483 | 0,45 | 0,1155 | 0,45 | 0,0892 |
| 16830235 | arachidonate 12-lipoxygenase pseudogene 2 | ALOX12P2 | 0,81 | 0,0431 | 0,45 | 0,1049 | 0,69 | 0,0653 |

^1^From Student’s t-test

**Table B. Pathways upregulated in response to S6K1/S6K2 siRNA, but not to single siRNA.**

| p-value | Term | Term ID | Term description | Genes |
| --- | --- | --- | --- | --- |
| 3.39e-08 | GO:0044237 | BP | cellular metabolic process | VCP, MKL1, POLR1C, TSFM, GNG7, BHLHA15, DUSP16, EXOC7, PAWR, SSR3, DCPS, MAST2, BZW2, HADH, HUS1, EI24, RPS29, TCF24, ACSL5, CTNNB1, GBAS, PMPCB, PELI1, MTHFD2L, INTS9, RPLP0, PDHB, TFDP2, PPP2R2B, ARHGEF10, MYO3A, SARS, PSPH, SRRM1, ZNF8, A4GNT, NTRK3, HEXIM1, CDS1, MLLT3, PDE6D, LIN9, MYT1, HHAT, CNOT2, FGFR1OP, PSIP1, TBPL2, THOC6, STT3B, ARPP19, NGDN, ATG3, SLC16A3, ERH, TCF25, NFKBIA, PPP6R3, UBA3, BUD13, CENPE, GHR, MAX, GPX4, CDK1, CPSF3, MTOR, ARL8B, ERCC8, WIPI1, PBX3, TRA2B, ADI1, ASB8, ZNF146, PNPT1, KAT2B, RPL22, PUS7, SULT2A1, PPCS, ACO1, AGPAT6, HDAC11, MAPK9, ZNF778, HMGCR, MESDC2, ZNF683, CCNF, PTPN2, ZER1, ZNF622, RAB40C, POGLUT1, SRSF1, PTP4A1, HOPX, TRAK1, JAK1, GPATCH1, FOXD2, ERLIN1, TRERF1, MLH1, NCOA3, ARIH2, NOL9, RBM11, ELMOD2, GTF2E2, NCAPH, NDUFB1, DALRD3, C17ORF70, HBS1L, LHCGR, XRCC4, RBBP8, KLHDC5, SETDB2, NBN, ROCK1, CDC42BPB, CREM, SEPHS1, PEX7, CDK5RAP2, POLE, QPRT, CTPS2, USP16, SUMF2, GGPS1, GALNT2, PYCR2, AES, BAG4, ZNF221, STK3, DUSP18, TRIP11, POLH, SLC35D1, ASAP3, EDEM3, PRAME, SMURF1, PRKDC, DVL2, PIGN, TNFRSF10B, PMPCA, EPT1, HERC6, METTL3, HS6ST1, BMPR2, TSC22D1, PHF10, RLIM, CYBRD1, ARID1B, ENTPD4, ABCC1, ABCE1, ZNF142, MTF2, FBXO5, SUV420H1, GNPAT, CHD3, ZNF471, RRM2B, NT5C2, MAT2B, CASZ1, HIGD1A, ANAPC13, MCM3AP, SDCBP, PHF12, PRPF18, CRAT, SRP9, CTSA, SLC35B3, POGK, DR1, YAP1, HMBOX1, GGT7, RICTOR, DNA2, GNE, ZNF605, GSTM4, RASA2, DNM1, UBE3B, FBXL5, MBTPS2, CCNT2, SLC35D2, PTPN21, PHIP, XRN2, EIF4E, CHD6, EEF2K, TBL1XR1, SERTAD3, TFAM, ALDH9A1, ASXL2, RPS6KA3, HCFC2, SNIP1, RIF1, CBL, FRYL, FURIN, PSEN1, CLCF1, CLPX, SYNJ1, CBFB, PHKB, GMPR2, ZBTB40, C1ORF85, ABL2, MAPK12, MED14, NUDT5, ZNF22, RAF1, TOPORS, HNRPLL, ARIH1, DOCK4, CHEK2, PIP4K2B, TAF11, HMGN2, ZSCAN29, FBXW11, ALG6, PHTF2, MARCH6, NUDT7, TADA1, BRPF3, CWC22, CAT, MLL3, MOSPD1, F2RL1, HNRNPD, NCOA2, NUDT12, TRIM32, ATXN7L3, HECTD1, MST1R, RNF13, FARS2, MSH6, BPNT1, MIS18BP1, MAPRE3, DSP, SMAD5, MMADHC, TRMT12, ACADSB, MPPE1, QTRTD1, SMAD3, UBAC1, TRIM33, CUL4B, NRBF2, MGAT5, CBX5, MAPK11, USP54, DNAJC10, HIST1H2BN, KLF11, MAP4K5, PIGB, ANKLE2, TRPS1, USP24, SPRR1B, YAF2, PLCB4, ZBTB42, PHF3, TAF5, ANKRD13C, FASTKD1, UGGT2, METTL14, TAOK1, ITPKB, ZNF544, SPOP, ZNF678, BAX, PIAS2, COX6C, STAT2, TLK2, CSNK1G3, GAB1, SLC25A4, CUL5, PATL1, BLOC1S2, PEAK1, ZNF724P, ARFGAP3, PCF11, ZRANB2, SWAP70, SH3GLB1, SNRK, FBXO33, DCAF10, DOCK1, CDC40, IRAK1BP1, UVRAG, ESCO1, MAOB, USP8, ORC2, HPX, TRIM8, MIF4GD, SCP2, ZNF555, MAN1A1, ZNF708, LRP6, BEX1, USP34, NFAT5, APLP1, FOSL1, EIF5A2, TMF1, MTAP, FZD6, ARNTL, PTGR2, FKTN, CSTF2T, GNPNAT1, MYLIP, RALGAPA2, NNT, RBM26, NAT1, ZFP90, CHD9, GBE1, CPEB2, ZNF420, ZNF644, ZNF107 |
| 2.77e-02 | GO:0046907 | BP | intracellular transport | VCP, BHLHA15, DUSP16, CACNB1, SSR3, KPNA5, KIFAP3, RPS29, PMPCB, IPO7, RPLP0, PPP2R2B, SRRM1, STX12, THOC6, SEC61A1, ATG3, NFKBIA, BUD13, FYTTD1, CDK1, CPSF3, MTOR, WIPI1, NXF1, NAPA, PNPT1, FTH1, RPL22, SLC25A37, MAPK9, YIPF5, SRSF1, TRAK1, PPP3R1, PEX7, RER1, TRIP11, RHOT1, SMURF1, PMPCA, BCL2L11, ARFIP1, MCM3AP, SDCBP, SRP9, CTSA, TMEM30A, PHIP, EPS15, EIF4E, PSEN1, HGSNAT, KIF13A, FBXW11, IPO8, MPPE1, SMAD3, APPBP2, TRPS1, TRAPPC8, KIF1C, AP3M2, BAX, BLOC1S2, ARFGAP3, SH3GLB1, DYNLL2, CDC40, SCP2, LRP6, ARNTL, SCAMP1 |
| 3.10e-03 | GO:0048285 | BP | organelle fission | NSL1, PPP2R2B, ARHGEF10, ARPP19, ATG3, CENPE, CDK1, MAU2, CCNF, NCAPH, RBBP8, KLHDC5, SETDB2, USP16, MIS12, HAUS8, FBXO5, SEH1L, HAUS6, KNTC1, KIF15, ANAPC13, EML4, PHIP, CLASP1, CHEK2, SPICE1, MIS18BP1, MAPRE3, ANKLE2, BAX |
| 2.23e-10 | GO:0005622 | CC | intracellular | CHID1, VCP, MOB3A, MKL1, CA5BP1, POLR1C, ITSN1, TSFM, GNG7, BHLHA15, DUSP16, EXOC7, PAWR, HOMER2, CACNB1, ABR, RHCG, SSR3, DNAJC15, DCPS, KPNA5, SAPCD2, KIFAP3, MAST2, HADH, HUS1, EI24, RPS29, NSL1, TIMMDC1, TCF24, ACSL5, CTNNB1, GBAS, PMPCB, PARVG, IPO7, MYL12B, PELI1, MTHFD2L, INTS9, RPLP0, PDHB, TFDP2, URB1, PPP2R2B, LAPTM4A, ARHGEF10, MYO3A, SARS, PSPH, SRRM1, ZNF8, MAPKAP1, A4GNT, VDAC3, NTRK3, HEXIM1, CDS1, SNX30, C2ORF47, ERMP1, MLLT3, LIN9, MYT1, HHAT, STX12, CNOT2, FGFR1OP, PSIP1, RABL3, TBPL2, THOC6, STT3B, SEC61A1, ARPP19, NGDN, ATG3, SLC16A3, TCF25, NFKBIA, PPP6R3, UBA3, BUD13, CENPE, GHR, MAX, SAV1, FYTTD1, GPX4, ACBD6, CDK1, CPSF3, MTOR, ARL8B, ERCC8, WIPI1, PPP1R13B, PBX3, SPA17, TMED4, DSTYK, TRA2B, WDR47, ADI1, NXF1, PTCHD2, ASB8, ZNF146, MPP5, MAU2, NAPA, PNPT1, ZFAT, FTH1, KAT2B, RABEP2, RPL22, SULT2A1, PPCS, ACO1, AGPAT6, SLC25A37, HDAC11, MAPK9, SYT4, ZNF778, YIPF5, HMGCR, MESDC2, ZNF683, CCNF, MX1, PTPN2, CYTH1, BCAS3, VPS39, ZER1, ZNF622, TAGLN, FERMT2, RAB40C, ARPC5L, POGLUT1, SRSF1, KCNG1, PTP4A1, HOPX, TRAK1, JAK1, GPATCH1, FOXD2, ERLIN1, TRERF1, MLH1, NCOA3, ARIH2, NOL9, RBM11, ELMOD2, GTF2E2, NCAPH, NDUFB1, THAP6, C17ORF70, LHCGR, PPP3R1, XRCC4, RBBP8, KLHDC5, SETDB2, NBN, ROCK1, CDC42BPB, CREM, SEPHS1, PEX7, CDK5RAP2, POLE, RER1, QPRT, MRPL40, CTPS2, USP16, SUMF2, GGPS1, GALNT2, PYCR2, EML1, AES, BAG4, ZNF221, WIPF2, STK3, DUSP18, TRIP11, POLH, RHOT1, SLC35D1, ASAP3, EDEM3, RAB11FIP2, PRAME, SMURF1, TBC1D4, TP53BP2, PRKDC, DVL2, PIGN, RPP25L, PMPCA, EPT1, HERC6, METTL3, HS6ST1, MIS12, PTPDC1, TSC22D1, PHF10, RLIM, HAUS8, ARID1B, C1ORF174, ENTPD4, DEPDC5, ABCC1, BCL2L11, ABCE1, ARFIP1, ZNF142, MTF2, FBXO5, SUV420H1, GNPAT, CHD3, SEH1L, SCAF4, DCTN4, MYH14, CHIC1, API5, HAUS6, ZNF471, RRM2B, KNTC1, KIF15, NT5C2, MAT2B, ATRN, CASZ1, HIGD1A, ANAPC13, MCM3AP, SDCBP, LETMD1, PHF12, PRPF18, CRAT, SRP9, CTSA, ATP11B, SLC35B3, POGK, DR1, EHBP1, YAP1, HMBOX1, RICTOR, DNA2, GNE, ZNF605, GNA14, GSTM4, EML4, TMEM30A, RASA2, DNM1, UBE3B, UBXN7, FBXL5, MBTPS2, RUFY1, CEP104, CCNT2, SLC35D2, PTPN21, PHIP, ACP1, EPS15, ARRDC3, XRN2, EIF4E, CHD6, EEF2K, TBL1XR1, ZMAT2, SERTAD3, LUZP1, TFAM, ALDH9A1, IPP, ASXL2, RPS6KA3, HCFC2, SNIP1, CLASP1, RIF1, CBL, FURIN, PSEN1, LANCL1, CLPX, SYNJ1, CBFB, HGSNAT, PHKB, GMPR2, ZBTB40, DYNC2H1, C1ORF85, ABL2, MAPK12, MED14, LETM2, RBM48, NUDT5, ZNF22, RAF1, TOPORS, HNRPLL, ARIH1, DOCK4, CHEK2, PIP4K2B, SPICE1, TAF11, HMGN2, ZSCAN29, KIF13A, FBXW11, ALG6, PHTF2, MARCH6, NUDT7, TADA1, HSDL1, BRPF3, CWC22, MRFAP1, CAT, MLL3, MOSPD1, F2RL1, HNRNPD, NCOA2, IPO8, NUDT12, TRIM32, ATXN7L3, HECTD1, MST1R, RNF13, DLG5, FARS2, MSH6, BPNT1, MIS18BP1, MAPRE3, TCTN1, DSP, SMAD5, MMADHC, IRX6, MIA3, ACADSB, MPPE1, QTRTD1, SMAD3, UBAC1, TRIM33, CUL4B, NRBF2, APPBP2, MGAT5, CBX5, HDGFRP3, MAPK11, RANBP6, DNAJC10, HIST1H2BN, RTN1, FNDC3A, KLF11, MAP4K5, CCDC113, PIGB, ANKLE2, TRPS1, SPRR1B, YAF2, PLCB4, ZBTB42, SLFN13, PHF3, GUSB, TRIO, TAF5, TRAPPC8, ANKRD13C, KIAA2018, KIF1C, FASTKD1, UGGT2, RPS6KC1, METTL14, TAOK1, AP3M2, CEP128, DGKA, ITPKB, ZNF544, DHRS7B, SPOP, STOX1, ZNF678, BAX, PIAS2, COX6C, MT1F, STAT2, ANO8, TLK2, CSNK1G3, GAB1, SLC25A4, CUL5, PDCD2, PATL1, BLOC1S2, PEAK1, ZNF724P, ARFGAP3, PCF11, ZRANB2, SWAP70, UTP3, SH3GLB1, SNRK, DCAF10, DYNLL2, ANKRD12, DOCK1, CDC40, IRAK1BP1, UVRAG, ESCO1, MAOB, USP8, ORC2, HPX, TRIM8, STEAP3, MIF4GD, GBP2, SCP2, ZNF555, MAN1A1, ZNF708, LRP6, BEX1, NFAT5, APLP1, FOSL1, EIF5A2, TMF1, MTAP, FZD6, ARNTL, PTGR2, FKTN, CSTF2T, GNPNAT1, TNNT1, SCAMP1, TMEM237, MYLIP, RALGAPA2, ANO10, GPSM2, MIB1, NNT, NAT1, ZFP90, SEPW1, CHD9, GBE1, CPEB2, ZNF420, ZNF644, ZNF107 |
| 1.30e-08 | BIOGRID:00000 | bi | BioGRID interaction data | CHID1, VCP, MOB3A, MKL1, POLR1C, C9ORF40, ITSN1, TSFM, GNG7, BHLHA15, DUSP16, EXOC7, PAWR, HOMER2, CACNB1, ABR, SSR3, DNAJC15, DCPS, C21ORF58, KPNA5, KIFAP3, MAST2, BZW2, CCL25, HADH, HUS1, EI24, RPS29, NSL1, TIMMDC1, TCF24, ZNF706, ACSL5, CTNNB1, GBAS, LRRC42, PMPCB, PARVG, IPO7, MYL12B, PELI1, INTS9, RPLP0, LEPROTL1, PDHB, TFDP2, URB1, RUNDC3A, PPP2R2B, LAPTM4A, ARHGEF10, MYO3A, SARS, PSPH, SRRM1, ZNF8, CCDC87, MAPKAP1, VDAC3, NTRK3, HEXIM1, CDS1, TMEM245, ZCCHC13, SNX30, C2ORF47, ERMP1, MLLT3, C12ORF4, OSBPL8, PDE6D, LIN9, MYT1, HHAT, STX12, CNOT2, MPPED2, FER1L6, FGFR1OP, PSIP1, RABL3, TBPL2, THOC6, STT3B, SEC61A1, ARPP19, NGDN, ATG3, SLC16A3, ERH, TCF25, NFKBIA, PPP6R3, UBA3, BUD13, CENPE, GHR, MAX, SAV1, FYTTD1, LONRF1, GPX4, ACBD6, CDK1, CPSF3, MTOR, ARL8B, ERCC8, WIPI1, CPD, PPP1R13B, PBX3, SPA17, TMED4, DSTYK, TRA2B, WDR47, ADI1, NXF1, PTCHD2, ASB8, ZNF146, MPP5, MAU2, NAPA, PNPT1, ZFAT, FTH1, KAT2B, RABEP2, RPL22, PUS7, SULT2A1, PPCS, HEATR3, ACO1, C2ORF76, AGPAT6, SLC25A37, HDAC11, MAPK9, SYT4, ZNF778, YIPF5, HMGCR, MESDC2, CCNF, MX1, PTPN2, CYTH1, BCAS3, VPS39, ZER1, ZNF622, TTC39C, TAGLN, KIAA1715, TMCC1, FERMT2, SLC22A16, METTL23, RAB40C, ARPC5L, POGLUT1, SRSF1, KCNG1, PTP4A1, HOPX, TRAK1, JAK1, GPATCH1, ERLIN1, TRERF1, MLH1, NCOA3, ARIH2, NOL9, RBM11, ELMOD2, GTF2E2, NCAPH, NDUFB1, THAP6, DALRD3, C17ORF70, HBS1L, LHCGR, PPP3R1, XRCC4, RBBP8, NCKAP5, KLHDC5, SETDB2, SLC39A11, NBN, ROCK1, CDC42BPB, CREM, SEPHS1, C12ORF23, PEX7, CDK5RAP2, POLE, RER1, QPRT, MRPL40, CTPS2, USP16, SUMF2, GGPS1, GALNT2, PYCR2, EML1, LAPTM4B, AES, BAG4, ZNF221, PRRC2B, WIPF2, C15ORF41, STK3, TSPAN5, TRIP11, POLH, RHOT1, SLC35D1, ASAP3, EDEM3, RAB11FIP2, PRAME |
| 5.00e-02 | CORUM:3979 | co | mTORC2 complex (mTOR/FRAP1, LST8, mAVO3/RICTOR, SIN1) | MAPKAP1, MTOR, RICTOR |
| 3.42e-04 | TF:M00803_0 | tf | Factor: E2F; motif: GGCGSG; match class: 0 | CHID1, VCP, MOB3A, MKL1, C9ORF40, TSFM, GNG7, DUSP16, PAWR, HOMER2, CACNB1, ABR, SSR3, C1ORF101, DNAJC15, DCPS, C21ORF58, KPNA5, SAPCD2, MAST2, HADH, RPS29, NSL1, ZNF706, OGFRL1, ACSL5, CTNNB1, GBAS, LRRC42, IPO7, MYL12B, RPLP0, LEPROTL1, PDHB, PPP2R2B, ARHGEF10, MYO3A, SARS, PSPH, SRRM1, MAPKAP1, VDAC3, NTRK3, CDS1, TMEM245, ZCCHC13, C2ORF47, ERMP1, HHAT, STX12, FGFR1OP, PSIP1, THOC6, SEC61A1, ATG3, SLC16A3, TCF25, NFKBIA, GHR, MAX, SAV1, FYTTD1, LONRF1, GPX4, ACBD6, CPSF3, MTOR, ARL8B, ERCC8, WIPI1, PPP1R13B, PBX3, SPA17, TMED4, TRA2B, WDR47, ADI1, NXF1, ASB8, MPP5, NAPA, ZFAT, FTH1, PUS7, SULT2A1, PPCS, HEATR3, C2ORF76, AGPAT6, SLC25A37, HDAC11, ZNF778, CCNF, MX1, PTPN2, CYTH1, BCAS3, SLCO4C1, ZER1, ZNF622, KIAA1715, FERMT2, SLC22A16, RAB40C, ARPC5L, GPATCH1, ERLIN1, TRERF1, ARIH2, NOL9, ELMOD2, GTF2E2, NCAPH, NDUFB1, DALRD3, C17ORF70, PPP3R1, KLHDC5, SETDB2, SLC39A11, CDC42BPB, CREM, SEPHS1, C12ORF23, POLE, RER1, MRPL40, CTPS2, USP16, PYCR2, EML1, LAPTM4B, AES, ENPP4, ZNF221, STK3, TSPAN5, DUSP18, POLH, SLC35D1, ASAP3, EDEM3, RAB11FIP2, SMURF1, TBC1D4, TMEM116, TP53BP2, PRKDC, TNFRSF10B, PMPCA, METTL3, HS6ST1, KHNYN, CNNM3, LRCH1, MIS12, GRAMD1C, PTPDC1, TSC22D1, PHF10, HAUS8, ARID1B, C1ORF174, ENTPD4, TMEM8A, DEPDC5, ABCC1, BCL2L11, REEP3, MTF2, FBXO5, SUV420H1, SEH1L, SCAF4, JOSD1, MYH14, C18ORF54, CHIC1, KIAA1377, KNTC1, NT5C2, MAT2B, ZYG11B, KRCC1, ATRN, CASZ1, HIGD1A, ANAPC13, TEX261, MCM3AP, LETMD1, PRPF18, CRAT, CTSA, ATP11B, POGK, DR1, EHBP1, YAP1, RICTOR, GNE, ZNF605, GNA14, EML4, TMA16, SLC35E4, SLC41A1, FBXL5, RUFY1, CEP104, CCNT2, SLC35D2, PTPN21, PHIP, ACP1, EPS15, ARRDC3, CHD6, EEF2K, TBL1XR1, SERTAD3, TFAM, ALDH9A1, ASXL2, SNIP1, CLASP1, RIF1, CBL, PSEN1, MRFAP1L1, CLPX, CBFB, PHKB, ZBTB40, ABL2, MAPK12, MED14, LETM2, RBM48, HNRPLL, ITGA2, PIP4K2B, SPICE1, TAF11, LAMA3, HMGN2, KIF13A, ALG6, MARCH6, TADA1, C20ORF111, HSDL1, BRPF3, CWC22, MRFAP1, TM2D3, MLL3, F2RL1, HNRNPD, NCOA2, NUDT12, TRIM32, DNAJC13, HECTD1, MST1R, RNF13, DLG5, FARS2, MSH6, BPNT1, MIS18BP1, MAPRE3, TCTN1, ATP2B4, DSP, SMAD5, IRX6, EFNB2, ACADSB, MPPE1, QTRTD1, SMAD3, UBAC1, TRIM33, APPBP2, MGAT5, CBX5, HDGFRP3, MAPK11, ZZEF1, RANBP6, DNAJC10, FNDC3A, KLF11, MAP4K5, CCDC113, PIGB, TRPS1, C6ORF226, YAF2, SLFN13, GUSB, TRIO, VPS13D, TRAPPC8, KIF1C, UGGT2, METTL14, SLC39A9, TAOK1, AP3M2, DGKA, ITPKB, ZNF544, MARK3, SPOP, BAX, PIAS2, STAT2, TLK2, CSNK1G3, CCDC126, GAB1, SLC25A4, ARFGAP3, ZRANB2, SWAP70, UTP3, SH3GLB1, SNRK, FBXO33, GPR77, DCAF10, DYNLL2, ANKRD12, DOCK1, CDC40, IRAK1BP1, FEZ2, SLC6A20, ESCO1, TRIM8, STEAP3, MIF4GD, GBP2, SCP2, MAN1A1, FCHSD2, ZNF708, LRP6, NFAT5, CCDC93, FOSL1, EIF5A2, PTGR2, TMEM237, ANO10, GPSM2, NNT, RBM26, ZFP90, SEPW1, CPEB2, ZNF644, ZNF107 |
| 1.71e-02 | TF:M00931_4 | tf | Factor: Sp1; motif: GGGGCGGGGC; match class: 4 | CHID1, MOB3A, MKL1, POLR1C, ITSN1, GNG7, DUSP16, PAWR, HOMER2, CACNB1, TRPM3, ABR, RHCG, C21ORF58, SAPCD2, MAST2, BZW2, HADH, NSL1, TIMMDC1, ZNF706, CTNNB1, GBAS, LRRC42, LEPROTL1, RUNDC3A, PPP2R2B, LAPTM4A, ARHGEF10, PSPH, SRRM1, MAPKAP1, NTRK3, CDS1, MLLT3, OSBPL8, STX12, CNOT2, FGFR1OP, PSIP1, ARPP19, ATG3, SLC16A3, TCF25, NFKBIA, PPP6R3, UBA3, BUD13, GHR, MAX, LONRF1, ACBD6, CPSF3, MTOR, WIPI1, CPD, PPP1R13B, PBX3, SPA17, TMED4, DSTYK, TRA2B, WDR47, NXF1, MPP5, NAPA, FTH1, RABEP2, PUS7, HEATR3, C2ORF76, AGPAT6, HDAC11, MESDC2, CCNF, MX1, PTPN2, CYTH1, TAGLN, FERMT2, RAB40C, ARPC5L, TRAK1, JAK1, ERLIN1, TRERF1, RBM11, ELMOD2, DALRD3, C17ORF70, LHCGR, PPP3R1, RBBP8, KLHDC5, SETDB2, SLC39A11, CDC42BPB, CREM, SEPHS1, POLE, RER1, MRPL40, CTPS2, PYCR2, EML1, AES, BAG4, STK3, TSPAN5, DUSP18, POLH, SLC35D1, ASAP3, RAB11FIP2, SMURF1, TBC1D4, TMEM116, TP53BP2, PIGN, RPP25L, EPT1, HS6ST1, CNNM3, MIS12, BMPR2, TSC22D1, PHF10, GREB1, SDE2, HAUS8, ARID1B, ENTPD4, TMEM8A, DEPDC5, ABCC1, REEP3, SUV420H1, SEH1L, SCAF4, JOSD1, MYH14, API5, KNTC1, KIF15, NT5C2, MAT2B, ATRN, CASZ1, MCM3AP, CRAT, ATP11B, POGK, EHBP1, GGT7, RICTOR, GSTM4, EML4, TMA16, SLC35E4, DNM1, UBE3B, FBXL5, CEP104, CCNT2, PTPN21, PHIP, ACP1, EPS15, CHD6, EEF2K, TBL1XR1, SERTAD3, LUZP1, TFAM, ASXL2, HCFC2, RIF1, CBL, FURIN, PSEN1, CLCF1, SYNJ1, CBFB, PHKB, GMPR2, ZBTB40, ABL2, MAPK12, MED14, RBM48, RAF1, ARIH1, ITGA2, HMGN2, KIF13A, MARCH6, C20ORF111, BRPF3, TM2D3, CAT, MLL3, TCL1B, MOSPD1, F2RL1, HNRNPD, DNAJC13, MST1R, FARS2, MSH6, MAPRE3, ATP2B4, DSP, EFNB2, ACADSB, MPPE1, QTRTD1, TRIM33, APPBP2, HDGFRP3, MAPK11, RANBP6, DNAJC10, FNDC3A, KLF11, TRPS1, YAF2, GUSB, TRIO, TAF5, TRAPPC8, KIF1C, TAOK1, AP3M2, DGKA, ITPKB, SLC22A15, ZNF544, DHRS7B, PIAS2, MT1F, ANO8, TLK2, GAB1, PDCD2, BLOC1S2, ARFGAP3, PCF11, SWAP70, SNRK, FBXO33, DCAF10, DYNLL2, ANKRD12, CDC40, FEZ2, SLC6A20, UVRAG, MAOB, USP8, ORC2, STEAP3, SCP2, ZNF555, MAN1A1, FCHSD2, NFAT5, CCDC93, APLP1, FOSL1, EIF5A2, ARNTL, PTGR2, FKTN, BTBD7, GNPNAT1, TNNT1, ARMC8, MYLIP, ANO10, GPSM2, NNT, RBM26, NAT1, BRWD3, CPEB2 |
| 8.65e-03 | TF:M00196_4 | tf | Factor: Sp1; motif: NGGGGGCGGGGYN; match class: 4 | CHID1, MOB3A, MKL1, POLR1C, ITSN1, GNG7, DUSP16, EXOC7, PAWR, HOMER2, CACNB1, TRPM3, ABR, RHCG, C21ORF58, SAPCD2, MAST2, BZW2, HADH, TIMMDC1, ZNF706, CTNNB1, GBAS, LRRC42, LEPROTL1, RUNDC3A, PPP2R2B, LAPTM4A, ARHGEF10, SRRM1, MAPKAP1, NTRK3, CDS1, MLLT3, OSBPL8, HHAT, STX12, CNOT2, FGFR1OP, PSIP1, ATG3, SLC16A3, TCF25, NFKBIA, PPP6R3, UBA3, BUD13, GHR, MAX, LONRF1, GPX4, ACBD6, CPSF3, MTOR, WIPI1, CPD, PPP1R13B, PBX3, SPA17, TMED4, DSTYK, TRA2B, WDR47, NXF1, MPP5, NAPA, FTH1, RABEP2, PUS7, HEATR3, ACO1, C2ORF76, AGPAT6, HDAC11, MESDC2, CCNF, MX1, PTPN2, CYTH1, VPS39, TAGLN, FERMT2, RAB40C, ARPC5L, TRAK1, JAK1, ERLIN1, TRERF1, RBM11, ELMOD2, DALRD3, C17ORF70, LHCGR, PPP3R1, RBBP8, SETDB2, SLC39A11, CDC42BPB, CREM, SEPHS1, C12ORF23, POLE, RER1, MRPL40, CTPS2, PYCR2, EML1, AES, BAG4, STK3, TSPAN5, DUSP18, POLH, ASAP3, RAB11FIP2, SMURF1, TMEM116, TP53BP2, PIGN, RPP25L, HS6ST1, CNNM3, MIS12, BMPR2, TSC22D1, PHF10, GREB1, SDE2, HAUS8, ARID1B, ENTPD4, TMEM8A, DEPDC5, ABCC1, REEP3, FBXO5, SUV420H1, SEH1L, SCAF4, MYH14, API5, KIAA1377, KNTC1, KIF15, NT5C2, MAT2B, ZYG11B, ATRN, CASZ1, MCM3AP, CRAT, ATP11B, POGK, EHBP1, GGT7, RICTOR, GSTM4, EML4, TMA16, SLC35E4, SLC41A1, DNM1, UBE3B, FBXL5, CEP104, CCNT2, SLC35D2, PTPN21, PHIP, ACP1, EPS15, CHD6, EEF2K, TBL1XR1, SERTAD3, LUZP1, TFAM, ASXL2, HCFC2, RIF1, CBL, FURIN, PSEN1, LANCL1, CLCF1, SYNJ1, CBFB, PHKB, GMPR2, ZBTB40, ABL2, MAPK12, MED14, RBM48, RAF1, ARIH1, ITGA2, CHEK2, HMGN2, KIF13A, ALG6, MARCH6, TADA1, C20ORF111, HSDL1, TM2D3, CAT, MLL3, TCL1B, MOSPD1, F2RL1, HNRNPD, DNAJC13, MST1R, FARS2, MSH6, MAPRE3, DSP, EFNB2, ACADSB, QTRTD1, TRIM33, NRBF2, APPBP2, HDGFRP3, MAPK11, ZZEF1, RANBP6, DNAJC10, FNDC3A, KLF11, TRPS1, YAF2, GUSB, TRIO, TAF5, TRAPPC8, KIF1C, TAOK1, AP3M2, DGKA, ITPKB, SLC22A15, ZNF544, DHRS7B, PIAS2, MT1F, STAT2, ANO8, TLK2, GAB1, PDCD2, BLOC1S2, ARFGAP3, PCF11, SWAP70, SNRK, FBXO33, DCAF10, DYNLL2, ANKRD12, DOCK1, CDC40, FEZ2, UVRAG, MAOB, USP8, ORC2, SCP2, ZNF555, MAN1A1, FCHSD2, MOSPD2, NFAT5, APLP1, FOSL1, EIF5A2, ARNTL, PTGR2, FKTN, BTBD7, GNPNAT1, TNNT1, ARMC8, TMEM237, MYLIP, ANO10, GPSM2, NNT, RBM26, NAT1, BRWD3, CPEB2, ZNF644 |
| 7.96e-03 | TF:M01035_4 | tf | Factor: YY1; motif: NYNKCCATNTT; match class: 4 | EXOC7, TRPM3, DCPS, C21ORF58, HUS1, CTNNB1, MYL12B, PELI1, INTS9, RPLP0, LAPTM4A, MAPKAP1, LELP1, THOC6, ERH, CENPE, CPD, DSTYK, ASB8, MAU2, NAPA, FTH1, RABEP2, PPCS, MESDC2, CYTH1, ZNF622, POGLUT1, SRSF1, TRAK1, JAK1, GPATCH1, FOXD2, NDUFB1, THAP6, DALRD3, SETDB2, NBN, CREM, SEPHS1, CDK5RAP2, MFSD6, RER1, QPRT, GGPS1, LAPTM4B, BAG4, WIPF2, PIGN, METTL3, KHNYN, MIS12, TSC22D1, GREB1, RLIM, CYBRD1, C1ORF174, ABCE1, MTF2, CHD3, ABCC11, C18ORF54, KNTC1, KIF15, PHF12, CRAT, SRP9, HMBOX1, FBXL5, RUFY1, SERTAD3, LUZP1, SNIP1, RIF1, FURIN, SYNJ1, ZBTB40, ABL2, RAF1, PIP4K2B, SPICE1, LAMA3, KIF13A, ALG6, CWC22, MOSPD1, DNAJC13, FARS2, MIS18BP1, TRMT12, ACADSB, NRBF2, CBX5, ZZEF1, RANBP6, RTN1, GUSB, TRIO, TAF5, KIAA2018, METTL14, SLC39A9, TAOK1, DHRS7B, MARK3, SPOP, BAX, PIAS2, MT1F, STAT2, GAB1, PATL1, ZRANB2, SH3GLB1, ESCO1, SCP2, TTC17, BEX1, NFAT5, APLP1, FOSL1, CSTF2T, RBM26, SEPW1, ZNF644 |
| 4.29e-02 | TF:M00196_1 | tf | Factor: Sp1; motif: NGGGGGCGGGGYN; match class: 1 | CHID1, MOB3A, ITSN1, GNG7, PAWR, CACNB1, ABR, RHCG, C21ORF58, SAPCD2, MAST2, HADH, TIMMDC1, CTNNB1, LRRC42, RUNDC3A, LAPTM4A, MAPKAP1, NTRK3, CDS1, CNOT2, FGFR1OP, PSIP1, ATG3, SLC16A3, PPP6R3, UBA3, BUD13, GHR, MAX, LONRF1, ACBD6, WIPI1, CPD, PPP1R13B, PBX3, SPA17, TMED4, WDR47, NXF1, NAPA, RABEP2, PUS7, HEATR3, AGPAT6, HDAC11, MESDC2, MX1, PTPN2, CYTH1, TAGLN, FERMT2, RAB40C, ARPC5L, RBM11, ELMOD2, DALRD3, C17ORF70, PPP3R1, SLC39A11, CDC42BPB, CREM, SEPHS1, PYCR2, EML1, AES, STK3, DUSP18, ASAP3, RAB11FIP2, SMURF1, TMEM116, PIGN, RPP25L, HS6ST1, BMPR2, PHF10, SDE2, HAUS8, ARID1B, TMEM8A, DEPDC5, ABCC1, SUV420H1, SEH1L, SCAF4, MYH14, MAT2B, CRAT, ATP11B, GSTM4, EML4, TMA16, SLC35E4, FBXL5, PTPN21, PHIP, ACP1, EPS15, CHD6, EEF2K, TBL1XR1, SERTAD3, LUZP1, TFAM, HCFC2, CBL, PSEN1, SYNJ1, CBFB, ZBTB40, ABL2, RBM48, RAF1, ARIH1, ITGA2, KIF13A, TM2D3, TCL1B, MOSPD1, MST1R, FARS2, MSH6, MAPRE3, DSP, EFNB2, APPBP2, MAPK11, DNAJC10, FNDC3A, KLF11, GUSB, TRIO, TRAPPC8, AP3M2, DGKA, SLC22A15, PIAS2, MT1F, PDCD2, PCF11, SWAP70, SNRK, FBXO33, DCAF10, DYNLL2, ANKRD12, CDC40, FEZ2, USP8, SCP2, ZNF555, MAN1A1, FCHSD2, NFAT5, FOSL1, EIF5A2, GNPNAT1, TNNT1, MYLIP, ANO10, GPSM2, RBM26, NAT1, CPEB2 |
| 1.20e-02 | TF:M00932_4 | tf | Factor: Sp1; motif: NNGGGGCGGGGNN; match class: 4 | CHID1, MOB3A, MKL1, POLR1C, ITSN1, GNG7, DUSP16, PAWR, HOMER2, CACNB1, TRPM3, ABR, RHCG, C21ORF58, SAPCD2, MAST2, BZW2, HADH, NSL1, TIMMDC1, ZNF706, CTNNB1, GBAS, LRRC42, INTS9, LEPROTL1, RUNDC3A, PPP2R2B, LAPTM4A, ARHGEF10, PSPH, SRRM1, MAPKAP1, NTRK3, CDS1, MLLT3, OSBPL8, STX12, CNOT2, FGFR1OP, PSIP1, ARPP19, ATG3, SLC16A3, TCF25, NFKBIA, PPP6R3, UBA3, BUD13, GHR, MAX, SAV1, LONRF1, GPX4, ACBD6, CPSF3, MTOR, WIPI1, CPD, PPP1R13B, PBX3, SPA17, TMED4, DSTYK, TRA2B, WDR47, NXF1, MPP5, NAPA, FTH1, RABEP2, PUS7, HEATR3, C2ORF76, AGPAT6, HDAC11, MESDC2, CCNF, MX1, PTPN2, CYTH1, VPS39, TAGLN, FERMT2, RAB40C, ARPC5L, TRAK1, JAK1, ERLIN1, TRERF1, RBM11, ELMOD2, DALRD3, C17ORF70, LHCGR, PPP3R1, RBBP8, SETDB2, SLC39A11, CDC42BPB, CREM, SEPHS1, POLE, RER1, MRPL40, CTPS2, PYCR2, EML1, AES, BAG4, STK3, TSPAN5, DUSP18, POLH, SLC35D1, ASAP3, RAB11FIP2, SMURF1, TBC1D4, TMEM116, TP53BP2, PIGN, RPP25L, EPT1, HS6ST1, CNNM3, MIS12, BMPR2, TSC22D1, PHF10, GREB1, SDE2, CYBRD1, HAUS8, ARID1B, C1ORF174, TMEM8A, DEPDC5, ABCC1, REEP3, FBXO5, SUV420H1, SEH1L, SCAF4, JOSD1, MYH14, CHIC1, API5, KNTC1, KIF15, NT5C2, MAT2B, ZYG11B, ATRN, CASZ1, MCM3AP, CRAT, ATP11B, POGK, EHBP1, HMBOX1, GGT7, RICTOR, GSTM4, EML4, TMA16, SLC35E4, DNM1, UBE3B, FBXL5, MBTPS2, CEP104, CCNT2, PTPN21, PHIP, ACP1, EPS15, CHD6, EEF2K, TBL1XR1, SERTAD3, LUZP1, TFAM, ASXL2, HCFC2, RIF1, CBL, FURIN, PSEN1, CLCF1, SYNJ1, CBFB, PHKB, GMPR2, ZBTB40, ABL2, MAPK12, MED14, RBM48, RAF1, ARIH1, ITGA2, LAMA3, HMGN2, KIF13A, MARCH6, C20ORF111, BRPF3, TM2D3, CAT, MLL3, TCL1B, MOSPD1, F2RL1, HNRNPD, DNAJC13, MST1R, FARS2, MSH6, MAPRE3, ATP2B4, DSP, EFNB2, ACADSB, MPPE1, QTRTD1, TRIM33, NRBF2, APPBP2, HDGFRP3, MAPK11, ZZEF1, RANBP6, DNAJC10, FNDC3A, KLF11, TRPS1, YAF2, GUSB, TRIO, TAF5, TRAPPC8, KIF1C, TAOK1, AP3M2, DGKA, ITPKB, SLC22A15, ZNF544, DHRS7B, BAX, PIAS2, MT1F, ANO8, TLK2, GAB1, PDCD2, BLOC1S2, ARFGAP3, PCF11, SWAP70, SNRK, FBXO33, DCAF10, DYNLL2, ANKRD12, CDC40, FEZ2, UVRAG, MAOB, USP8, ORC2, STEAP3, SCP2, ZNF555, MAN1A1, FCHSD2, MOSPD2, NFAT5, CCDC93, APLP1, FOSL1, EIF5A2, ARNTL, PTGR2, FKTN, BTBD7, GNPNAT1, TNNT1, ARMC8, TMEM237, MYLIP, ANO10, GPSM2, NNT, RBM26, NAT1, BRWD3, CPEB2 |
| 3.77e-04 | TF:M00695_0 | tf | Factor: ETF; motif: GVGGMGG; match class: 0 | CHID1, MOB3A, C9ORF40, TSFM, GNG7, PAWR, CACNB1, ABR, C21ORF58, KPNA5, BZW2, EI24, ZNF706, OGFRL1, CTNNB1, GBAS, LRRC42, MYL12B, RPLP0, LEPROTL1, PSPH, NTRK3, CDS1, ERMP1, C12ORF4, PSIP1, SEC61A1, ERH, TCF25, NFKBIA, PPP6R3, GHR, MAX, SAV1, ARL8B, WIPI1, CPD, PPP1R13B, PBX3, SPA17, WDR47, NXF1, MPP5, PPCS, ACO1, C2ORF76, SLC25A37, CCNF, PTPN2, SLCO4C1, ZER1, ZNF622, KIAA1715, FERMT2, SLC22A16, RAB40C, ARPC5L, POGLUT1, ERLIN1, TRERF1, NCOA3, NDUFB1, DALRD3, HBS1L, RBBP8, KLHDC5, CDC42BPB, CREM, C12ORF23, POLE, RER1, MRPL40, GGPS1, EML1, AES, BAG4, WIPF2, STK3, TSPAN5, DUSP18, POLH, RHOT1, SLC35D1, ASAP3, RAB11FIP2, SMURF1, TBC1D4, TP53BP2, PRKDC, RPP25L, PMPCA, HS6ST1, KHNYN, CNNM3, LRCH1, MIS12, BMPR2, PTPDC1, TSC22D1, PHF10, SDE2, RLIM, CYBRD1, HAUS8, ARID1B, C1ORF174, ENTPD4, TMEM8A, ABCC1, BCL2L11, ABCE1, REEP3, MTF2, SUV420H1, CHD3, SEH1L, SCAF4, JOSD1, C18ORF54, KIAA1377, NT5C2, KRCC1, ATRN, CASZ1, HIGD1A, TEX261, CRAT, CTSA, ATP11B, SLC35B3, EHBP1, CLEC16A, YAP1, SGK196, GGT7, RICTOR, GNE, GNA14, TMA16, SLC35E4, MFAP3, FBXL5, TMEM87A, PTPN21, PHIP, ACP1, XRN2, RSPH3, EEF2K, TBL1XR1, SERTAD3, TFAM, CBL, PSEN1, CLCF1, SYNJ1, CBFB, PHKB, GMPR2, ABL2, MAPK12, RBM48, NUDT5, CHEK2, LAMA3, KIF13A, ALG6, C20ORF111, HSDL1, BRPF3, MRFAP1, MLL3, HNRNPD, NCOA2, DNAJC13, HECTD1, MST1R, FARS2, MAPRE3, DSP, EFNB2, ACADSB, MPPE1, SMAD3, UBAC1, HDGFRP3, MAPK11, ZZEF1, DNAJC10, RTN1, FNDC3A, KLF11, TRPS1, YAF2, SLFN13, TRIO, UGGT2, SLC39A9, TAOK1, AP3M2, DGKA, ITPKB, SLC22A15, ZNF544, PIAS2, STAT2, TLK2, CSNK1G3, CCDC126, GAB1, ARFGAP3, PCF11, SWAP70, SH3GLB1, DYNLL2, ANKRD12, DOCK1, CDC40, FEZ2, SLC6A20, ESCO1, TRIM8, STEAP3, MIF4GD, MAN1A1, FCHSD2, LRP6, MOSPD2, NFAT5, CCDC93, MTAP, FZD6, ARNTL, FKTN, ANO10, GPSM2, NNT, RBM26, GBE1, CPEB2, ZNF644 |
| 1.07e-02 | TF:M00793_1 | tf | Factor: YY1; motif: GCCATNTTN; match class: 1 | EXOC7, CACNB1, TRPM3, DCPS, C21ORF58, HUS1, CTNNB1, PMPCB, MYL12B, PELI1, INTS9, RPLP0, LAPTM4A, HEXIM1, LELP1, RABL3, THOC6, ERH, CENPE, CPD, MAU2, NAPA, FTH1, RABEP2, PPCS, HMGCR, MESDC2, CYTH1, ZNF622, SLC22A16, POGLUT1, SRSF1, PTP4A1, JAK1, GPATCH1, FOXD2, NDUFB1, THAP6, DALRD3, KLHDC5, SETDB2, NBN, CREM, SEPHS1, CDK5RAP2, MFSD6, RER1, QPRT, GGPS1, LAPTM4B, WIPF2, PIGN, METTL3, KHNYN, MIS12, TSC22D1, GREB1, RLIM, ARID1B, C1ORF174, ABCE1, MTF2, CHD3, ABCC11, C18ORF54, KNTC1, KIF15, PHF12, CRAT, SRP9, HMBOX1, GNE, MFAP3, FBXL5, RUFY1, SERTAD3, LUZP1, SNIP1, FURIN, SYNJ1, ZBTB40, ABL2, LETM2, RAF1, PIP4K2B, SPICE1, LAMA3, KIF13A, ALG6, CWC22, MOSPD1, DNAJC13, FARS2, TRMT12, ACADSB, MPPE1, NRBF2, CBX5, ZZEF1, RANBP6, RTN1, GUSB, TAF5, KIAA2018, METTL14, SLC39A9, TAOK1, DHRS7B, MARK3, SPOP, BAX, PIAS2, MT1F, STAT2, CSNK1G3, GAB1, PATL1, ZRANB2, SWAP70, SH3GLB1, UVRAG, ESCO1, SCP2, TTC17, BEX1, NFAT5, APLP1, FOSL1, CSTF2T, RBM26, ZNF644 |

**Table C. Genes downregulated in response to double S6K1/S6K2 siRNA, but not to single siRNA.**

| Transcripts Cluster ID | Gene description | Gene symbol | S6K1 siRNA  and  S6K2 siRNA  Fold change | S6K1 siRNA  and  S6K2 siRNA  p-value^1^ | S6K1 siRNA  Fold change | S6K1 siRNA  p-value^1^ | S6K2 siRNA  Fold change | S6K2 siRNA  p-value^1^ |
| --- | --- | --- | --- | --- | --- | --- | --- | --- |
| 16679981 | tubulin, beta 8 class VIII | TUBB8 | -0,65 | 0,0117 | -0,79 | 0,1323 | -0,51 | 0,3086 |
| 16884285 | two pore channel 3 pseudogene \| LIMS3-LOC440895 readthrough | LOC440895\| LOC100507334\| LIMS3-LOC440895 | -0,46 | 0,0426 | -0,22 | 0,1599 | -0,32 | 0,2823 |
| 16856060 | myelin basic protein | MBP | -0,46 | 0,0060 | -0,39 | 0,0531 | -0,26 | 0,1859 |
| 16786906 | neuroglobin \| microRNA 1260a | NGB\| MIR1260A | -0,46 | 0,0045 | -0,19 | 0,4370 | -0,18 | 0,4637 |
| 16797224 | G protein-coupled receptor 132 | GPR132 | -0,45 | 0,0080 | -0,08 | 0,1862 | -0,15 | 0,2499 |
| 16740211 | glycoprotein hormone alpha 2 | GPHA2 | -0,45 | 0,0438 | -0,31 | 0,2116 | -0,3 | 0,1848 |
| 16916196 | transcription elongation factor A (SII), 2 | TCEA2 | -0,44 | 0,0412 | -0,36 | 0,1228 | -0,19 | 0,1368 |
| 16872966 | pregnancy specific beta-1-glycoprotein 9 | PSG9 | -0,44 | 0,0288 | -0,38 | 0,3613 | -0,35 | 0,1031 |
| 16862643 | Rho guanine nucleotide exchange factor (GEF) 1 | ARHGEF1 | -0,43 | 0,0426 | -0,35 | 0,2009 | -0,42 | 0,0546 |
| 16876520 | syntrophin, gamma 2 | SNTG2 | -0,43 | 0,0460 | -0,31 | 0,1698 | -0,27 | 0,3431 |
| 17000208 | vault RNA 2-1 | VTRNA2-1 | -0,43 | 0,0207 | -0,42 | 0,0859 | -0,35 | 0,2780 |
| 16808363 | small EDRK-rich factor 2 \| microRNA 1282 | SERF2\| MIR1282 | -0,42 | 0,0467 | -0,27 | 0,3518 | -0,5 | 0,0882 |
| 17057381 | myosin IG | MYO1G | -0,42 | 0,0224 | -0,31 | 0,2588 | -0,43 | 0,1488 |
| 16672349 | olfactory receptor, family 10, subfamily K, member 1 | OR10K1 | -0,41 | 0,0130 | -0,23 | 0,4643 | -0,14 | 0,3636 |
| 16781908 | T cell receptor alpha variable 18 | TRAV18 | -0,40 | 0,0361 | -0,21 | 0,3555 | -0,34 | 0,0661 |
| 16747098 | UDP-N-acetyl-alpha-D-galactosamine:polypeptide N-acetylgalactosaminyltransferase 8 (GalNAc-T8) | GALNT8 | -0,40 | 0,0322 | -0,19 | 0,5805 | -0,28 | 0,3541 |
| 17116533 | testis-specific transcript, Y-linked 4 (non-protein coding) \| testis-specific transcript, Y-linked 4B (non-protein coding) \| testis-specific transcript, Y-linked 4C (non-protein coding) | TTTY4\| TTTY4B\| TTTY4C | -0,39 | 0,0098 | -0,30 | 0,0527 | -0,19 | 0,0639 |
| 16834151 | keratin associated protein 9-8 | KRTAP9-8 | -0,38 | 0,0221 | -0,42 | 0,1030 | -0,27 | 0,1787 |
| 16775785 | glypican 5 | GPC5 | -0,38 | 0,0022 | -0,23 | 0,0506 | -0,24 | 0,0521 |
| 17108740 | purinergic receptor P2Y, G-protein coupled, 8 | P2RY8 | -0,38 | 0,0366 | -0,09 | 0,2702 | -0,25 | 0,0703 |
| 16690001 | DPYD intronic transcript 1 (non-protein coding) | DPYD-IT1 | -0,37 | 0,0342 | -0,34 | 0,1213 | -0,33 | 0,1918 |
| 16886174 | kynureninase | KYNU | -0,37 | 0,0404 | -0,24 | 0,2048 | -0,07 | 0,5366 |
| 16695463 | intelectin 1 (galactofuranose binding) | ITLN1 | -0,37 | 0,0429 | -0,28 | 0,1676 | -0,14 | 0,2846 |
| 16901497 | ST6 beta-galactosamide alpha-2,6-sialyltranferase 2 | ST6GAL2 | -0,37 | 0,0084 | -0,22 | 0,2690 | -0,3 | 0,1248 |
| 16961487 | solute carrier family 2 (facilitated glucose transporter), member 2 | SLC2A2 | -0,36 | 0,0158 | -0,44 | 0,2110 | -0,27 | 0,2465 |
| 16936271 | interleukin 17 receptor E-like | IL17REL | -0,35 | 0,0410 | -0,46 | 0,1782 | -0,26 | 0,1688 |
| 17004337 | tubulin, beta 2A class IIa | TUBB2A | -0,35 | 0,0204 | -0,40 | 0,1377 | -0,26 | 0,3411 |
| 17004333 | tubulin, beta 2A class IIa | TUBB2A | -0,35 | 0,0215 | -0,40 | 0,1398 | -0,26 | 0,3430 |
| 16969384 | CXXC finger protein 4 | CXXC4 | -0,35 | 0,0376 | -0,40 | 0,3316 | -0,25 | 0,0648 |
| 16662018 | doublecortin domain containing 2B | DCDC2B | -0,34 | 0,0141 | -0,17 | 0,2893 | -0,22 | 0,1196 |
| 16913619 | adipogenin | ADIG | -0,34 | 0,0495 | -0,24 | 0,1240 | -0,23 | 0,0607 |
| 16709951 | chromosome 10 open reading frame 85 | C10orf85 | -0,34 | 0,0427 | -0,14 | 0,1166 | -0,26 | 0,1002 |
| 16761843 | Rho GDP dissociation inhibitor (GDI) beta | ARHGDIB | -0,34 | 0,0399 | -0,49 | 0,0917 | -0,15 | 0,1724 |
| 16693414 | S100 calcium binding protein A8 | S100A8 | -0,33 | 0,0191 | -0,13 | 0,0803 | -0,05 | 0,1632 |
| 16854137 | ankyrin repeat domain 30B pseudogene | LOC644669 | -0,33 | 0,0285 | -0,16 | 0,4155 | 0,03 | 0,8896 |
| 16864849 | zinc finger protein 528 | ZNF528 | -0,33 | 0,0243 | -0,13 | 0,0715 | -0,28 | 0,0854 |
| 17111549 | P antigen family, member 3 (prostate associated) | PAGE3 | -0,32 | 0,0236 | -0,15 | 0,2898 | -0,36 | 0,0591 |
| 16817641 | zymogen granule protein 16 homolog (rat) | ZG16 | -0,32 | 0,0269 | -0,21 | 0,2264 | -0,28 | 0,3117 |
| 16830152 | uncharacterized LOC339166 \| WSC domain containing 1 | LOC339166\| WSCD1 | -0,31 | 0,0048 | -0,20 | 0,2628 | -0,26 | 0,1412 |
| 16968638 | dentin sialophosphoprotein | DSPP | -0,31 | 0,0178 | -0,09 | 0,1275 | -0,12 | 0,2169 |
| 17070249 | stathmin-like 2 | STMN2 | -0,31 | 0,0114 | -0,33 | 0,1418 | -0,36 | 0,0569 |
| 16672838 | Fc fragment of IgE, high affinity I, receptor for; gamma polypeptide | FCER1G | -0,31 | 0,0130 | -0,19 | 0,3256 | -0,29 | 0,1863 |
| 17093743 | tropomyosin 2 (beta) | TPM2 | -0,30 | 0,0061 | -0,06 | 0,4270 | -0,17 | 0,2562 |
| 17046601 | argininosuccinate lyase | ASL | -0,30 | 0,0415 | -0,13 | 0,4304 | -0,21 | 0,2464 |
| 16867905 | methyl-CpG binding domain protein 3-like 4 \| methyl-CpG binding domain protein 3-like 3 \| methyl-CpG binding domain protein 3-like 5 \| methyl-CpG binding domain protein 3-like 2 | MBD3L4\| MBD3L3\| MBD3L5\| MBD3L2 | -0,30 | 0,0448 | -0,21 | 0,1791 | -0,12 | 0,4805 |
| 16722844 | NEL-like 1 (chicken) | NELL1 | -0,30 | 0,0342 | -0,09 | 0,6352 | 0,04 | 0,9049 |
| 16836476 | protein phosphatase, Mg2+/Mn2+ dependent, 1E | PPM1E | -0,30 | 0,0068 | -0,15 | 0,4673 | -0,21 | 0,2305 |
| 16662329 | gap junction protein, alpha 4, 37kDa | GJA4 | -0,30 | 0,0063 | -0,11 | 0,5880 | -0,14 | 0,5019 |
| 16840772 | potassium voltage-gated channel, shaker-related subfamily, beta member 3 | KCNAB3 | -0,30 | 0,0461 | -0,16 | 0,2204 | -0,23 | 0,2885 |
| 16843078 | solute carrier family 6 (neurotransmitter transporter, serotonin), member 4 | SLC6A4 | -0,30 | 0,0262 | -0,19 | 0,3436 | -0,21 | 0,4263 |
| 16960560 | purinergic receptor P2Y, G-protein coupled, 14 | P2RY14 | -0,29 | 0,0138 | -0,01 | 0,9129 | -0,08 | 0,3445 |
| 16669549 | phosphodiesterase 4D interacting protein pseudogene | LOC100130000 | -0,29 | 0,0438 | -0,27 | 0,1821 | -0,27 | 0,2971 |
| 16844477 | keratin 10 | KRT10 | -0,29 | 0,0144 | 0,04 | 0,8828 | -0,16 | 0,0655 |
| 16807511 | protein phosphatase 1, regulatory (inhibitor) subunit 14D | PPP1R14D | -0,29 | 0,0290 | -0,16 | 0,0759 | -0,17 | 0,2225 |
| 16669960 | neuroblastoma breakpoint family, member 10 | NBPF10 | -0,29 | 0,0235 | -0,23 | 0,0749 | -0,25 | 0,0728 |
| 16805859 | golgin A6 family-like 6 | GOLGA6L6 | -0,29 | 0,0450 | -0,24 | 0,0845 | -0,3 | 0,3164 |
| 16719953 | secretoglobin, family 1C, member 1 \| secretoglobin, family 1C, member 1-like | SCGB1C1\| LOC653486 | -0,29 | 0,0262 | -0,08 | 0,2936 | -0,12 | 0,3883 |
| 16664581 | chromosome 1 open reading frame 185 | C1orf185 | -0,29 | 0,0319 | -0,27 | 0,2511 | -0,29 | 0,0549 |
| 16657656 | tubulin tyrosine ligase-like family, member 10 | TTLL10 | -0,28 | 0,0203 | -0,18 | 0,0943 | -0,29 | 0,1070 |
| 16729431 | aquaporin 11 | AQP11 | -0,28 | 0,0177 | -0,15 | 0,2423 | -0,21 | 0,1271 |
| 17007446 | prefoldin subunit 6 | PFDN6 | -0,28 | 0,0258 | -0,20 | 0,2564 | -0,28 | 0,1427 |
| 17041253 | HLA-DQB1 antisense RNA 1 (non-protein coding) | HLA-DQB1-AS1 | -0,28 | 0,0094 | 0,02 | 0,9472 | -0,17 | 0,3032 |
| 17005880 | histone cluster 1, H2bo | HIST1H2BO | -0,28 | 0,0272 | -0,29 | 0,1285 | -0,24 | 0,0871 |
| 16838085 | progressive rod-cone degeneration | PRCD | -0,28 | 0,0482 | -0,23 | 0,1007 | -0,24 | 0,3277 |
| 16744370 | crystallin, alpha B | CRYAB | -0,28 | 0,0139 | -0,04 | 0,4883 | -0,05 | 0,0516 |
| 17115763 | coagulation factor VIII-associated 1 \| coagulation factor VIII-associated 2 \| coagulation factor VIII-associated 3 \| microRNA 1184-1 \| microRNA 1184-2 \| microRNA 1184-3 | F8A1\| F8A2\| F8A3\| MIR1184-1\| MIR1184-2\| MIR1184-3 | -0,28 | 0,0428 | -0,47 | 0,1385 | -0,25 | 0,3052 |
| 16849992 | phosphate cytidylyltransferase 2, ethanolamine | PCYT2 | -0,28 | 0,0343 | -0,15 | 0,3880 | -0,1 | 0,4380 |
| 16944231 | zinc finger and BTB domain containing 20 | ZBTB20 | -0,28 | 0,0242 | -0,24 | 0,1851 | -0,16 | 0,3079 |
| 17115796 | coagulation factor VIII-associated 1 \| coagulation factor VIII-associated 2 \| coagulation factor VIII-associated 3 \| microRNA 1184-1 \| microRNA 1184-2 \| microRNA 1184-3 | F8A1\| F8A2\| F8A3\| MIR1184-1\| MIR1184-2\| MIR1184-3 | -0,28 | 0,0446 | -0,47 | 0,1381 | -0,24 | 0,3143 |
| 16748111 | A2M antisense RNA 1 (non-protein coding) | A2M-AS1 | -0,27 | 0,0403 | -0,05 | 0,8004 | -0,19 | 0,0831 |
| 16869293 | chromosome 19 open reading frame 43 | C19orf43 | -0,27 | 0,0355 | -0,02 | 0,8967 | -0,1 | 0,4566 |
| 16900441 | dual specificity phosphatase 2 | DUSP2 | -0,27 | 0,0045 | -0,10 | 0,1496 | -0,27 | 0,1012 |
| 17108585 | coagulation factor VIII-associated 1 \| coagulation factor VIII-associated 2 \| coagulation factor VIII-associated 3 \| microRNA 1184-1 \| microRNA 1184-2 \| microRNA 1184-3 | F8A1\| F8A2\| F8A3\| MIR1184-1\| MIR1184-2\| MIR1184-3 | -0,27 | 0,0443 | -0,47 | 0,1327 | -0,24 | 0,3105 |
| 16874202 | chorionic gonadotropin, beta polypeptide 7 | CGB7 | -0,27 | 0,0201 | -0,17 | 0,2508 | -0,16 | 0,4174 |
| 17070492 | ATPase, H+ transporting, lysosomal 38kDa, V0 subunit d2 | ATP6V0D2 | -0,27 | 0,0466 | -0,24 | 0,2057 | -0,3 | 0,0856 |
| 16862118 | Charcot-Leyden crystal protein pseudogene | LGALS17A | -0,27 | 0,0049 | -0,12 | 0,5145 | -0,17 | 0,1738 |
| 16732799 | olfactory receptor, family 10, subfamily G, member 9 | OR10G9 | -0,27 | 0,0439 | -0,08 | 0,7791 | 0,08 | 0,1498 |
| 16910948 | gonadotropin-releasing hormone 2 | GNRH2 | -0,27 | 0,0035 | -0,08 | 0,5800 | -0,05 | 0,1858 |
| 16781841 | T cell receptor alpha variable 6 | TRAV6 | -0,27 | 0,0286 | -0,10 | 0,5697 | -0,15 | 0,2300 |
| 16912370 | cytochrome c oxidase subunit IV isoform 2 (lung) | COX4I2 | -0,27 | 0,0197 | -0,36 | 0,0578 | -0,24 | 0,3131 |
| 17039511 | lymphocyte antigen 6 complex, locus G6E (pseudogene) | LY6G6E | -0,26 | 0,0042 | -0,27 | 0,2722 | -0,24 | 0,0668 |
| 16923314 | crystallin, alpha A | CRYAA | -0,26 | 0,0452 | -0,13 | 0,1677 | -0,02 | 0,6790 |
| 17086845 | sushi domain containing 3 | SUSD3 | -0,26 | 0,0375 | -0,20 | 0,3633 | -0,11 | 0,3064 |
| 16934660 | testis expressed 33 | TEX33 | -0,26 | 0,0276 | -0,26 | 0,0959 | -0,22 | 0,0974 |
| 16810298 |  | MGC15885 | -0,26 | 0,0336 | -0,12 | 0,3084 | -0,13 | 0,3051 |
| 16923580 | chromosome 21 open reading frame 2 | C21orf2 | -0,26 | 0,0054 | -0,11 | 0,1568 | -0,19 | 0,1360 |
| 17082507 | block of proliferation 1 | BOP1 | -0,26 | 0,0333 | -0,08 | 0,3697 | -0,09 | 0,4906 |
| 16945841 | beaded filament structural protein 2, phakinin | BFSP2 | -0,26 | 0,0445 | -0,29 | 0,3657 | -0,27 | 0,2343 |
| 16903897 | nuclear receptor subfamily 4, group A, member 2 | NR4A2 | -0,26 | 0,0368 | -0,27 | 0,1419 | -0,02 | 0,8399 |
| 16955064 | transketolase | TKT | -0,25 | 0,0360 | -0,16 | 0,1811 | -0,06 | 0,4691 |
| 16995494 | EGF-like, fibronectin type III and laminin G domains | EGFLAM | -0,25 | 0,0114 | -0,04 | 0,6503 | -0,14 | 0,1573 |
| 16724818 | purinergic receptor P2X, ligand-gated ion channel, 3 | P2RX3 | -0,25 | 0,0096 | -0,13 | 0,6130 | -0,02 | 0,9042 |
| 16697971 | leiomodin 1 (smooth muscle) | LMOD1 | -0,25 | 0,0087 | 0,03 | 0,8905 | -0,14 | 0,3906 |
| 17057983 | coiled-coil-helix-coiled-coil-helix domain containing 2 | CHCHD2 | -0,25 | 0,0252 | -0,19 | 0,2113 | -0,18 | 0,0765 |
| 17083352 | insulin-like 4 (placenta) | INSL4 | -0,25 | 0,0403 | -0,19 | 0,3883 | -0,15 | 0,2571 |
| 17041994 | lymphocyte antigen 6 complex, locus G6E (pseudogene) | LY6G6E | -0,25 | 0,0085 | -0,26 | 0,2884 | -0,22 | 0,0884 |
| 16686922 | AGBL4 intronic transcript 1 (non-protein coding) | AGBL4-IT1 | -0,25 | 0,0404 | -0,22 | 0,0644 | -0,22 | 0,1152 |
| 16864713 | carcinoembryonic antigen-related cell adhesion molecule 18 | CEACAM18 | -0,25 | 0,0044 | -0,13 | 0,4473 | -0,15 | 0,2355 |
| 16803427 | SH2 domain containing 7 | SH2D7 | -0,24 | 0,0478 | -0,19 | 0,2312 | -0,12 | 0,4373 |
| 16820927 | dihydroorotate dehydrogenase (quinone) | DHODH | -0,24 | 0,0358 | 0,05 | 0,6622 | -0,11 | 0,7330 |
| 16852457 | chromosome 18 open reading frame 26 | C18orf26 | -0,24 | 0,0008 | -0,21 | 0,2130 | -0,25 | 0,3036 |
| 17038609 | major histocompatibility complex, class II, DQ alpha 1 \| HLA class II histocompatibility antigen, DQ alpha 1 chain-like | HLA-DQA1\| LOC100509457\| LOC100507718 | -0,24 | 0,0498 | -0,15 | 0,2538 | -0,17 | 0,1014 |
| 17036058 | prefoldin subunit 6 | PFDN6 | -0,24 | 0,0067 | -0,10 | 0,3473 | -0,19 | 0,2213 |
| 16708990 | sortilin-related VPS10 domain containing receptor 3 | SORCS3 | -0,24 | 0,0187 | -0,21 | 0,0802 | -0,13 | 0,1058 |
| 16830778 | transmembrane protein 88 | TMEM88 | -0,24 | 0,0168 | 0,08 | 0,5988 | -0,09 | 0,4697 |
| 17067011 | neurofilament, medium polypeptide | NEFM | -0,24 | 0,0197 | -0,06 | 0,7027 | -0,05 | 0,6574 |
| 17001617 | calcium/calmodulin-dependent protein kinase II alpha | CAMK2A | -0,24 | 0,0465 | -0,04 | 0,8165 | -0,05 | 0,7416 |
| 17057433 | transforming growth factor beta regulator 4 \| small nucleolar RNA, H/ACA box 5B | TBRG4\| SNORA5B | -0,23 | 0,0222 | -0,19 | 0,3092 | -0,09 | 0,4679 |
| 16693383 | peptidoglycan recognition protein 3 | PGLYRP3 | -0,23 | 0,0178 | 0,16 | 0,3633 | -0,09 | 0,2842 |
| 17038779 | prefoldin subunit 6 | PFDN6 | -0,23 | 0,0068 | -0,10 | 0,3550 | -0,19 | 0,2267 |
| 16861895 | nuclear factor of kappa light polypeptide gene enhancer in B-cells inhibitor, beta | NFKBIB | -0,23 | 0,0050 | -0,11 | 0,3464 | -0,08 | 0,1854 |
| 16758299 | leucine rich repeat containing 43 | LRRC43 | -0,23 | 0,0230 | -0,09 | 0,3053 | -0,12 | 0,1870 |
| 16732956 | hepatocellular carcinoma, down-regulated 1 \| hepatic and glial cell adhesion molecule | HEPN1\| HEPACAM | -0,23 | 0,0072 | -0,16 | 0,5266 | -0,18 | 0,0626 |
| 17031309 | prefoldin subunit 6 | PFDN6 | -0,23 | 0,0040 | -0,09 | 0,3578 | -0,18 | 0,2280 |
| 16671090 | sperm mitochondria-associated cysteine-rich protein | SMCP | -0,23 | 0,0371 | -0,08 | 0,3265 | -0,01 | 0,7792 |
| 16852683 | phorbol-12-myristate-13-acetate-induced protein 1 | PMAIP1 | -0,23 | 0,0473 | -0,06 | 0,7097 | -0,01 | 0,9510 |
| 16747137 | potassium voltage-gated channel, shaker-related subfamily, member 5 | KCNA5 | -0,23 | 0,0334 | -0,23 | 0,2345 | -0,06 | 0,4020 |
| 16941939 | WNT5A antisense RNA 1 (non-protein coding) | WNT5A-AS1 | -0,23 | 0,0277 | -0,08 | 0,5229 | -0,1 | 0,1163 |
| 17028495 | prefoldin subunit 6 | PFDN6 | -0,23 | 0,0073 | -0,09 | 0,3567 | -0,18 | 0,2212 |
| 16916852 | centromere protein B, 80kDa | CENPB | -0,23 | 0,0399 | -0,37 | 0,0636 | -0,2 | 0,1949 |
| 16827546 | dipeptidase 2 | DPEP2 | -0,23 | 0,0014 | -0,07 | 0,5578 | -0,08 | 0,6211 |
| 16859101 | olfactory receptor, family 1, subfamily I, member 1 | OR1I1 | -0,23 | 0,0111 | -0,10 | 0,1189 | -0,26 | 0,0626 |
| 16686401 | mutY homolog (E. coli) | MUTYH | -0,22 | 0,0293 | -0,09 | 0,3011 | -0,05 | 0,4039 |
| 16754241 | tryptophan hydroxylase 2 | TPH2 | -0,22 | 0,0096 | -0,04 | 0,7401 | -0,18 | 0,2238 |
| 16817083 | calcium channel, voltage-dependent, gamma subunit 3 | CACNG3 | -0,22 | 0,0209 | -0,26 | 0,4861 | -0,11 | 0,2544 |
| 16960344 | transmembrane 4 L six family member 18 | TM4SF18 | -0,22 | 0,0052 | -0,28 | 0,2320 | -0,07 | 0,5356 |
| 16931838 | acrosin | ACR | -0,22 | 0,0234 | -0,03 | 0,6968 | -0,04 | 0,4236 |
| 16853994 | tubulin, beta 6 class V | TUBB6 | -0,22 | 0,0172 | -0,22 | 0,2417 | -0,06 | 0,7632 |
| 16923659 | keratin associated protein 10-9 | KRTAP10-9 | -0,22 | 0,0257 | -0,25 | 0,2275 | 0 | 0,9573 |
| 16932008 | BH3 interacting domain death agonist | BID | -0,22 | 0,0184 | -0,10 | 0,1445 | -0,11 | 0,1574 |
| 16958335 | rhophilin associated tail protein 1 | ROPN1 | -0,22 | 0,0169 | -0,16 | 0,4028 | -0,14 | 0,2901 |
| 17105130 | cylicin, basic protein of sperm head cytoskeleton 1 | CYLC1 | -0,22 | 0,0420 | -0,10 | 0,0848 | -0,23 | 0,0594 |
| 17008341 | dishevelled associated activator of morphogenesis 2 | DAAM2 | -0,22 | 0,0129 | -0,03 | 0,5474 | -0,06 | 0,4404 |
| 16911261 | bone morphogenetic protein 2 | BMP2 | -0,22 | 0,0121 | -0,02 | 0,8367 | -0,18 | 0,1263 |
| 16892012 | G protein-coupled receptor 55 | GPR55 | -0,22 | 0,0356 | -0,13 | 0,4424 | -0,17 | 0,1037 |
| 16873268 | excision repair cross-complementing rodent repair deficiency, complementation group 2 | ERCC2 | -0,22 | 0,0048 | -0,37 | 0,1107 | -0,12 | 0,0798 |
| 16752639 | canopy 2 homolog (zebrafish) | CNPY2 | -0,22 | 0,0122 | -0,21 | 0,4009 | -0,06 | 0,6514 |
| 16736658 | NAV2 antisense RNA 5 (non-protein coding) | NAV2-AS5 | -0,21 | 0,0395 | -0,27 | 0,2909 | -0,12 | 0,2977 |
| 16849238 | rhomboid 5 homolog 2 (Drosophila) | RHBDF2 | -0,21 | 0,0296 | 0,02 | 0,7908 | 0,02 | 0,8983 |
| 16918664 | eukaryotic translation initiation factor 6 | EIF6 | -0,21 | 0,0222 | -0,05 | 0,6699 | -0,11 | 0,2240 |
| 16804165 | golgin A6 family-like 4 \| golgin A6 family-like 3 | GOLGA6L4\| GOLGA6L3 | -0,21 | 0,0262 | -0,01 | 0,9701 | -0,18 | 0,2873 |
| 16864244 | protein arginine methyltransferase 1 | PRMT1 | -0,21 | 0,0417 | -0,02 | 0,8033 | -0,06 | 0,2565 |
| 16995914 | chemokine (C-C motif) ligand 28 | CCL28 | -0,21 | 0,0426 | -0,18 | 0,2269 | -0,19 | 0,1084 |
| 16848568 |  | MGC16275 | -0,21 | 0,0462 | -0,18 | 0,1841 | -0,25 | 0,1119 |
| 16954817 | troponin C type 1 (slow) | TNNC1 | -0,21 | 0,0188 | -0,20 | 0,2410 | -0,19 | 0,1079 |
| 17077359 | proenkephalin | PENK | -0,21 | 0,0249 | -0,13 | 0,0650 | -0,18 | 0,2308 |
| 17034468 | lymphocyte antigen 6 complex, locus G6E (pseudogene) | LY6G6E | -0,21 | 0,0343 | -0,21 | 0,2875 | -0,17 | 0,1895 |
| 17060380 | alpha-2-glycoprotein 1, zinc-binding | AZGP1 | -0,21 | 0,0480 | -0,16 | 0,1270 | -0,08 | 0,1089 |
| 17071722 | dendrocyte expressed seven transmembrane protein | DCSTAMP | -0,21 | 0,0172 | 0,06 | 0,6872 | -0,1 | 0,2037 |
| 16798587 | WAS protein homolog associated with actin, golgi membranes and microtubules pseudogene 2 | WHAMMP2 | -0,21 | 0,0448 | -0,06 | 0,7464 | -0,14 | 0,4365 |
| 17100697 | NADH dehydrogenase, subunit 6 (complex I) | ND6 | -0,21 | 0,0365 | 0,17 | 0,3074 | -0,25 | 0,1113 |
| 17040242 | zinc finger and BTB domain containing 22 | ZBTB22 | -0,21 | 0,0330 | -0,18 | 0,1719 | -0,17 | 0,0712 |
| 16986913 | versican | VCAN | -0,21 | 0,0379 | -0,24 | 0,0837 | -0,15 | 0,1937 |
| 16966801 | GS homeobox 2 | GSX2 | -0,21 | 0,0208 | -0,13 | 0,2218 | -0,2 | 0,0934 |
| 16807342 | phospholipase C, beta 2 | PLCB2 | -0,21 | 0,0405 | -0,04 | 0,1034 | -0,1 | 0,2867 |
| 16957221 | MORC family CW-type zinc finger 1 | MORC1 | -0,21 | 0,0021 | -0,23 | 0,0738 | -0,36 | 0,0794 |
| 16981631 | glycine receptor, alpha 3 | GLRA3 | -0,21 | 0,0139 | -0,09 | 0,4628 | -0,2 | 0,0639 |
| 16711023 | tubulin, beta 8 class VIII | TUBB8 | -0,21 | 0,0206 | -0,04 | 0,4450 | -0,14 | 0,4531 |
| 16716127 | leucine-rich repeat, immunoglobulin-like and transmembrane domains 2 | LRIT2 | -0,21 | 0,0443 | -0,03 | 0,5173 | -0,05 | 0,5248 |
| 17081945 | secreted LY6/PLAUR domain containing 1 | SLURP1 | -0,20 | 0,0463 | 0,09 | 0,6744 | -0,1 | 0,5516 |
| 16805874 | neurobeachin pseudogene 1 | NBEAP1 | -0,20 | 0,0458 | 0,01 | 0,9448 | -0,15 | 0,4110 |
| 16872517 | cytochrome P450, family 2, subfamily A, polypeptide 6 | CYP2A6 | -0,20 | 0,0092 | 0,01 | 0,9356 | 0,02 | 0,8547 |
| 17029213 | lymphocyte antigen 6 complex, locus G6E (pseudogene) | LY6G6E | -0,20 | 0,0158 | -0,21 | 0,3178 | -0,18 | 0,1503 |
| 17073213 | prostate stem cell antigen | PSCA | -0,20 | 0,0323 | -0,14 | 0,1393 | -0,06 | 0,1069 |
| 17031998 | lymphocyte antigen 6 complex, locus G6E (pseudogene) | LY6G6E | -0,20 | 0,0279 | -0,21 | 0,3078 | -0,17 | 0,1909 |
| 17115098 | paraneoplastic Ma antigen family member 6D \| paraneoplastic Ma antigen family member 6C \| paraneoplastic Ma antigen family member 6A | PNMA6D\| PNMA6C\| PNMA6A | -0,20 | 0,0428 | -0,07 | 0,6603 | -0,3 | 0,1552 |
| 16904586 | uncharacterized LOC100506124 \| tetratricopeptide repeat domain 21B | LOC100506124\| TTC21B | -0,20 | 0,0436 | -0,08 | 0,4120 | -0,24 | 0,0744 |
| 16874672 | kallikrein-related peptidase 4 | KLK4 | -0,20 | 0,0464 | -0,17 | 0,2054 | -0,18 | 0,3245 |
| 16887840 | cell division cycle associated 7 | CDCA7 | -0,20 | 0,0067 | -0,08 | 0,3982 | 0 | 0,9183 |
| 16890566 | transmembrane protein 169 | TMEM169 | -0,20 | 0,0411 | -0,07 | 0,3767 | 0 | 0,9193 |
| 16693365 | small proline-rich protein 2C (pseudogene) | SPRR2C | -0,20 | 0,0203 | 0,12 | 0,3593 | -0,04 | 0,6333 |
| 17118254 | uncharacterized LOC100507042 \| chromosome 7 open reading frame 73 pseudogene | LOC100507042\| LOC100289196 | -0,20 | 0,0396 | -0,31 | 0,1692 | -0,12 | 0,5714 |
| 16871444 | IGF-like family receptor 1 | IGFLR1 | -0,20 | 0,0130 | -0,17 | 0,1525 | -0,12 | 0,3002 |
| 16764398 | formin-like 3 | FMNL3 | -0,20 | 0,0243 | -0,08 | 0,2514 | -0,1 | 0,5364 |
| 16841768 | centromere protein V | CENPV | -0,20 | 0,0372 | -0,12 | 0,4536 | -0,14 | 0,1782 |
| 16897276 | multiple coagulation factor deficiency 2 | MCFD2 | -0,20 | 0,0281 | -0,01 | 0,9229 | -0,1 | 0,3937 |
| 16807820 | phospholipase A2, group IVF | PLA2G4F | -0,20 | 0,0094 | -0,16 | 0,3085 | -0,12 | 0,3140 |
| 16833080 | SH3-domain GRB2-like 1 pseudogene 1 | SH3GL1P1 | -0,20 | 0,0495 | -0,14 | 0,4064 | -0,06 | 0,3938 |
| 16837634 | solute carrier family 9, subfamily A (NHE3, cation proton antiporter 3), member 3 regulator 1 \| microRNA 3615 | SLC9A3R1\| MIR3615 | -0,20 | 0,0074 | -0,07 | 0,3064 | -0,16 | 0,0540 |
| 16812761 | DNM1 pseudogene 41 | DNM1P41 | -0,20 | 0,0500 | -0,09 | 0,4262 | -0,11 | 0,1970 |
| 17056564 | NPSR1 antisense RNA 1 (non-protein coding) | NPSR1-AS1 | -0,20 | 0,0378 | -0,17 | 0,4495 | -0,12 | 0,4372 |
| 17036716 | lymphocyte antigen 6 complex, locus G6E (pseudogene) | LY6G6E | -0,19 | 0,0379 | -0,20 | 0,3297 | -0,16 | 0,2193 |
| 16917912 | cystatin 11 | CST11 | -0,19 | 0,0319 | -0,05 | 0,6288 | -0,18 | 0,1009 |
| 17073519 | sphingomyelin phosphodiesterase 5 | SMPD5 | -0,19 | 0,0464 | -0,13 | 0,0730 | -0,1 | 0,1528 |
| 16777773 | MTUS2 antisense RNA 1 (non-protein coding) | MTUS2-AS1 | -0,19 | 0,0000 | -0,13 | 0,3690 | -0,11 | 0,3829 |
| 16663514 | cell division cycle 20 homolog (S. cerevisiae) | CDC20 | -0,19 | 0,0144 | -0,03 | 0,3980 | -0,03 | 0,6532 |
| 16898567 | gastrokine 2 | GKN2 | -0,19 | 0,0097 | -0,15 | 0,3665 | -0,02 | 0,9020 |
| 16884602 | interleukin 36, alpha | IL36A | -0,19 | 0,0323 | 0,02 | 0,7797 | -0,03 | 0,8078 |
| 16921048 | chromosome 20 open reading frame 151 | C20orf151 | -0,19 | 0,0001 | -0,18 | 0,0847 | -0,14 | 0,2469 |
| 16659200 | migration and invasion inhibitory protein | MIIP | -0,19 | 0,0095 | 0,04 | 0,4563 | -0,12 | 0,0746 |
| 16819630 | testis, prostate and placenta expressed | TEPP | -0,19 | 0,0323 | 0,09 | 0,2260 | -0,12 | 0,1826 |
| 16705641 | chromosome 10 open reading frame 35 | C10orf35 | -0,19 | 0,0430 | -0,18 | 0,2372 | -0,19 | 0,3660 |
| 16880942 | pleckstrin | PLEK | -0,19 | 0,0175 | -0,08 | 0,3474 | -0,08 | 0,3759 |
| 16773547 | pancreatic and duodenal homeobox 1 | PDX1 | -0,19 | 0,0494 | -0,13 | 0,1600 | -0,15 | 0,1506 |
| 17006633 | DDX39B antisense RNA 1 (non-protein coding) | DDX39B-AS1 | -0,19 | 0,0176 | -0,16 | 0,2824 | -0,17 | 0,1167 |
| 16895673 | solute carrier family 5 (sodium-dependent vitamin transporter), member 6 | SLC5A6 | -0,19 | 0,0481 | -0,08 | 0,3868 | -0,07 | 0,1226 |
| 16975310 | UDP-glucose 6-dehydrogenase | UGDH | -0,19 | 0,0237 | -0,13 | 0,4192 | 0 | 0,9944 |
| 16679805 | olfactory receptor, family 2, subfamily T, member 2 | OR2T2 | -0,19 | 0,0140 | -0,11 | 0,7013 | -0,03 | 0,8010 |
| 16919524 | potassium voltage-gated channel, delayed-rectifier, subfamily S, member 1 | KCNS1 | -0,19 | 0,0402 | -0,01 | 0,8897 | -0,26 | 0,0979 |
| 16935176 | Sad1 and UNC84 domain containing 2 | SUN2 | -0,19 | 0,0458 | -0,07 | 0,4498 | -0,1 | 0,3859 |
| 16792501 | ribosomal protein L36a-like | RPL36AL | -0,19 | 0,0223 | -0,14 | 0,0906 | -0,05 | 0,4536 |
| 17088815 | olfactory receptor, family 1, subfamily L, member 3 | OR1L3 | -0,19 | 0,0438 | -0,07 | 0,4640 | -0,05 | 0,6181 |
| 16778632 | chromosome 13 open reading frame 44 | C13orf44 | -0,18 | 0,0148 | -0,06 | 0,7635 | -0,07 | 0,4624 |
| 16831525 | coiled-coil domain containing 144A | CCDC144A | -0,18 | 0,0073 | 0,03 | 0,8349 | 0 | 0,9926 |
| 16659371 | PRAME family member 1 | PRAMEF1 | -0,18 | 0,0164 | 0,20 | 0,0551 | -0,12 | 0,6159 |
| 16967323 | ferritin, light polypeptide pseudogene 10 | FTLP10 | -0,18 | 0,0098 | -0,15 | 0,1028 | -0,23 | 0,1017 |
| 17035519 | casein kinase 2, beta polypeptide | CSNK2B | -0,18 | 0,0320 | -0,12 | 0,1164 | -0,15 | 0,1209 |
| 17044877 | growth hormone releasing hormone receptor | GHRHR | -0,18 | 0,0201 | -0,12 | 0,5279 | -0,04 | 0,1544 |
| 17112108 | DMRT-like family C1B \| DMRT-like family C1 \| family with sequence similarity 226, member B (non-protein coding) | DMRTC1B\| FAM226B | -0,18 | 0,0322 | -0,23 | 0,1271 | -0,15 | 0,2872 |
| 16862145 | proteasome (prosome, macropain) 26S subunit, ATPase, 4 | PSMC4 | -0,18 | 0,0398 | -0,12 | 0,1196 | -0,09 | 0,1887 |
| 16927856 | immunoglobulin lambda joining 3 | IGLJ3 | -0,18 | 0,0263 | -0,13 | 0,5721 | -0,23 | 0,3833 |
| 16980905 | acid-sensing (proton-gated) ion channel family member 5 | ASIC5 | -0,18 | 0,0036 | -0,12 | 0,1022 | -0,16 | 0,0954 |
| 16896170 | calpain 14 | CAPN14 | -0,18 | 0,0105 | 0,00 | 0,9935 | -0,2 | 0,1421 |
| 17045258 | G protein-coupled receptor 141 | GPR141 | -0,18 | 0,0205 | -0,09 | 0,3009 | -0,1 | 0,3705 |
| 17113629 | ribosomal protein L39 \| uncharacterized LOC100652821 | RPL39\| LOC100652821 | -0,18 | 0,0088 | -0,03 | 0,6273 | 0 | 0,9607 |
| 17059323 | sema domain, immunoglobulin domain (Ig), short basic domain, secreted, (semaphorin) 3A | SEMA3A | -0,18 | 0,0095 | -0,02 | 0,5094 | -0,18 | 0,1467 |
| 16873015 | X-ray repair complementing defective repair in Chinese hamster cells 1 | XRCC1 | -0,18 | 0,0381 | -0,18 | 0,0696 | -0,11 | 0,1185 |
| 16856812 | thimet oligopeptidase 1 | THOP1 | -0,18 | 0,0171 | -0,16 | 0,0824 | -0,13 | 0,1394 |
| 16833297 | fibronectin type III domain containing 8 | FNDC8 | -0,18 | 0,0378 | -0,01 | 0,9062 | -0,11 | 0,1791 |
| 16663498 | myeloproliferative leukemia virus oncogene | MPL | -0,18 | 0,0321 | -0,04 | 0,3232 | -0,12 | 0,2320 |
| 17040686 | DDX39B antisense RNA 1 (non-protein coding) | DDX39B-AS1 | -0,18 | 0,0312 | -0,14 | 0,2920 | -0,15 | 0,1881 |
| 16867476 | scaffold attachment factor B2 | SAFB2 | -0,18 | 0,0214 | -0,08 | 0,6102 | -0,22 | 0,1674 |
| 16667130 | chromosome 1 open reading frame 146 | C1orf146 | -0,17 | 0,0144 | -0,09 | 0,4507 | -0,13 | 0,3082 |
| 17044596 | JAZF1 antisense RNA 1 (non-protein coding) | JAZF1-AS1 | -0,17 | 0,0048 | -0,05 | 0,6579 | -0,05 | 0,7034 |
| 16887492 | glutamate decarboxylase 1 (brain, 67kDa) | GAD1 | -0,17 | 0,0265 | -0,21 | 0,1178 | -0,21 | 0,2452 |
| 17094674 | chromosome 9 open reading frame 71 | C9orf71 | -0,17 | 0,0186 | -0,01 | 0,9402 | -0,13 | 0,3505 |
| 16986965 | neuroblastoma breakpoint family, member 22, pseudogene | NBPF22P | -0,17 | 0,0224 | -0,01 | 0,9490 | -0,06 | 0,2275 |
| 16691333 | calsequestrin 2 (cardiac muscle) | CASQ2 | -0,17 | 0,0482 | -0,15 | 0,1103 | -0,08 | 0,2523 |
| 17082023 | lymphocyte antigen 6 complex, locus H | LY6H | -0,17 | 0,0428 | -0,05 | 0,3369 | -0,15 | 0,0520 |
| 17097052 | thioredoxin | TXN | -0,17 | 0,0310 | -0,16 | 0,0910 | -0,01 | 0,8609 |
| 17086072 | forkhead box B2 | FOXB2 | -0,17 | 0,0360 | -0,06 | 0,3041 | -0,07 | 0,5380 |
| 16794846 | placental growth factor | PGF | -0,17 | 0,0182 | -0,03 | 0,7558 | -0,14 | 0,2117 |
| 16865910 | uncharacterized LOC100128252 \| uncharacterized LOC100288114 | LOC100128252\| LOC100288114 | -0,17 | 0,0245 | -0,13 | 0,1303 | 0,12 | 0,5440 |
| 16959797 | retinol binding protein 2, cellular | RBP2 | -0,17 | 0,0150 | -0,05 | 0,4949 | -0,14 | 0,2564 |
| 16977781 | uncharacterized LOC100506746 \| chromosome 4 open reading frame 36 | LOC100506746\| C4orf36 | -0,17 | 0,0465 | -0,11 | 0,6094 | -0,19 | 0,3346 |
| 17027898 | casein kinase 2, beta polypeptide | CSNK2B | -0,17 | 0,0331 | -0,09 | 0,1886 | -0,12 | 0,2455 |
| 17059788 | HEPACAM family member 2 | HEPACAM2 | -0,17 | 0,0231 | 0,02 | 0,6988 | -0,01 | 0,9382 |
| 16803642 |  | KIAA1024 | -0,17 | 0,0309 | -0,02 | 0,8883 | -0,08 | 0,3567 |
| 16914042 | hepatocyte nuclear factor 4, alpha | HNF4A | -0,17 | 0,0443 | 0,02 | 0,9221 | -0,02 | 0,8591 |
| 16748061 | polyhomeotic homolog 1 (Drosophila) | PHC1 | -0,17 | 0,0156 | -0,21 | 0,1786 | -0,07 | 0,6166 |
| 16917335 | sel-1 suppressor of lin-12-like 2 (C. elegans) | SEL1L2 | -0,17 | 0,0264 | -0,10 | 0,5189 | -0,02 | 0,7421 |
| 16872580 | ATP5S-like | ATP5SL | -0,17 | 0,0161 | -0,09 | 0,3885 | -0,19 | 0,1338 |
| 16710216 | chromosome 10 open reading frame 88 pseudogene | LOC399815 | -0,17 | 0,0070 | -0,04 | 0,7732 | -0,12 | 0,2464 |
| 16905401 | HOXD cluster antisense RNA 2 (non-protein coding) | HOXD-AS2 | -0,17 | 0,0371 | -0,16 | 0,3807 | -0,1 | 0,4817 |
| 16979553 | transient receptor potential cation channel, subfamily C, member 3 | TRPC3 | -0,17 | 0,0166 | -0,06 | 0,4681 | -0,01 | 0,3040 |
| 16913077 | matrix metallopeptidase 24 (membrane-inserted) | MMP24 | -0,17 | 0,0089 | -0,11 | 0,1950 | -0,17 | 0,1521 |
| 16952990 | protease, serine, 50 \| protease, serine, 45 | PRSS50\| PRSS45 | -0,17 | 0,0480 | -0,17 | 0,1854 | -0,15 | 0,2124 |
| 16742202 | arrestin, beta 1 | ARRB1 | -0,17 | 0,0455 | -0,23 | 0,0785 | -0,14 | 0,1088 |
| 17061156 | F-box and leucine-rich repeat protein 13 | FBXL13 | -0,17 | 0,0327 | -0,08 | 0,3355 | -0,1 | 0,4299 |
| 16980031 | mastermind-like 3 (Drosophila) | MAML3 | -0,16 | 0,0312 | -0,08 | 0,4872 | -0,2 | 0,2333 |
| 16981024 | chromosome 4 open reading frame 45 | C4orf45 | -0,16 | 0,0060 | -0,13 | 0,2008 | -0,18 | 0,0533 |
| 16874401 | polynucleotide kinase 3'-phosphatase | PNKP | -0,16 | 0,0016 | -0,17 | 0,2473 | -0,03 | 0,6933 |
| 16773165 | tumor necrosis factor receptor superfamily, member 19 | TNFRSF19 | -0,16 | 0,0188 | -0,06 | 0,6135 | -0,05 | 0,6579 |
| 17039885 | chromosome 6 open reading frame 10 | C6orf10 | -0,16 | 0,0175 | -0,17 | 0,2224 | -0,22 | 0,1228 |
| 17033428 | casein kinase 2, beta polypeptide | CSNK2B | -0,16 | 0,0337 | -0,10 | 0,1630 | -0,13 | 0,2052 |
| 16676795 | complement component (3d/Epstein Barr virus) receptor 2 | CR2 | -0,16 | 0,0442 | -0,03 | 0,3319 | -0,04 | 0,6303 |
| 17008404 | apolipoprotein B mRNA editing enzyme, catalytic polypeptide-like 2 | APOBEC2 | -0,16 | 0,0403 | -0,05 | 0,7495 | -0,11 | 0,2712 |
| 16747373 | COP9 constitutive photomorphogenic homolog subunit 7A (Arabidopsis) | COPS7A | -0,16 | 0,0097 | -0,07 | 0,4178 | -0,08 | 0,2392 |
| 16803103 | mannose phosphate isomerase | MPI | -0,16 | 0,0031 | -0,17 | 0,2883 | -0,23 | 0,1333 |
| 16794719 | latent transforming growth factor beta binding protein 2 | LTBP2 | -0,16 | 0,0072 | -0,08 | 0,2178 | -0,16 | 0,0556 |
| 17097560 | AT-hook transcription factor | AKNA | -0,16 | 0,0408 | 0,02 | 0,8074 | 0 | 0,9912 |
| 17083370 | programmed cell death 1 ligand 2 | PDCD1LG2 | -0,16 | 0,0322 | 0,04 | 0,4634 | -0,09 | 0,1800 |
| 16730693 | DNA-damage inducible 1 homolog 1 (S. cerevisiae) | DDI1 | -0,16 | 0,0164 | -0,11 | 0,4255 | -0,11 | 0,1909 |
| 16960084 | solute carrier family 9, subfamily A (NHE9, cation proton antiporter 9), member 9 | SLC9A9 | -0,16 | 0,0148 | 0,02 | 0,7795 | -0,05 | 0,7360 |
| 16935069 |  | DMC1 | -0,15 | 0,0248 | 0,00 | 0,9552 | -0,11 | 0,2460 |
| 16955565 | family with sequence similarity 107, member A \| uncharacterized LOC100506924 | FAM107A\| LOC100506924 | -0,15 | 0,0120 | -0,19 | 0,0991 | -0,1 | 0,4637 |
| 16968307 | gene differentially expressed in prostate | GDEP | -0,15 | 0,0415 | 0,08 | 0,6354 | -0,13 | 0,3418 |
| 17062137 | ankyrin repeat, SAM and basic leucine zipper domain containing 1 | ASZ1 | -0,15 | 0,0096 | -0,08 | 0,0740 | -0,14 | 0,1798 |
| 16818394 | alpha hemoglobin stabilizing protein | AHSP | -0,15 | 0,0420 | -0,15 | 0,1528 | -0,11 | 0,2495 |
| 16989977 | cysteine-rich transmembrane module containing 1 | CYSTM1 | -0,15 | 0,0141 | -0,05 | 0,4466 | 0 | 0,8087 |
| 16724789 | olfactory receptor, family 8, subfamily J, member 1 | OR8J1 | -0,15 | 0,0413 | -0,14 | 0,2202 | -0,17 | 0,0713 |
| 16942689 | PDZRN3 antisense RNA 1 (non-protein coding) | PDZRN3-AS1 | -0,15 | 0,0085 | -0,07 | 0,4318 | -0,17 | 0,2521 |
| 16882142 | thymosin beta 10 | TMSB10 | -0,15 | 0,0258 | -0,14 | 0,0528 | -0,16 | 0,1516 |
| 16833426 | chemokine (C-C motif) ligand 4-like 2 \| chemokine (C-C motif) ligand 4-like 1 \| chemokine (C-C motif) ligand 4 | CCL4L2\| CCL4L1 | -0,15 | 0,0252 | 0,02 | 0,8669 | -0,17 | 0,2223 |
| 17114682 | chromosome X open reading frame 66 | CXorf66 | -0,15 | 0,0474 | -0,12 | 0,4111 | -0,18 | 0,1216 |
| 16859446 | occludin/ELL domain containing 1 | OCEL1 | -0,15 | 0,0016 | -0,13 | 0,3524 | -0,14 | 0,1689 |
| 16764870 | keratin 82 | KRT82 | -0,15 | 0,0362 | -0,11 | 0,2321 | -0,17 | 0,1660 |
| 16994030 | succinate dehydrogenase complex, subunit A, flavoprotein pseudogene 3 | SDHAP3 | -0,15 | 0,0386 | 0,01 | 0,9591 | -0,17 | 0,2838 |
| 16872796 | chemokine (C-X-C motif) ligand 17 | CXCL17 | -0,15 | 0,0164 | -0,17 | 0,2780 | 0 | 0,9940 |
| 16708728 | sideroflexin 2 | SFXN2 | -0,15 | 0,0124 | -0,10 | 0,2422 | -0,22 | 0,0884 |
| 17085829 | guanine deaminase | GDA | -0,15 | 0,0357 | -0,01 | 0,9292 | -0,33 | 0,0767 |
| 16757990 | acyl-CoA dehydrogenase, C-2 to C-3 short chain | ACADS | -0,15 | 0,0028 | -0,08 | 0,1645 | -0,08 | 0,4188 |
| 16658752 | retinol binding protein 7, cellular | RBP7 | -0,15 | 0,0438 | -0,02 | 0,7404 | -0,08 | 0,2654 |
| 16921679 | chondrolectin | CHODL | -0,15 | 0,0332 | -0,02 | 0,6244 | -0,04 | 0,7029 |
| 17109873 | SMEK homolog 3, suppressor of mek1 (Dictyostelium) pseudogene | SMEK3P | -0,15 | 0,0364 | -0,16 | 0,0638 | -0,15 | 0,2611 |
| 17113147 | TSC22 domain family, member 3 | TSC22D3 | -0,15 | 0,0347 | -0,22 | 0,2575 | 0,02 | 0,7569 |
| 16989554 | TRPC7 antisense RNA 2 (non-protein coding) | TRPC7-AS2 | -0,14 | 0,0253 | -0,02 | 0,8685 | -0,18 | 0,1082 |
| 16678886 | solute carrier family 35, member F3 | SLC35F3 | -0,14 | 0,0014 | -0,07 | 0,4882 | -0,12 | 0,2636 |
| 16761745 | guanylate cyclase 2C (heat stable enterotoxin receptor) | GUCY2C | -0,14 | 0,0184 | -0,09 | 0,4535 | -0,04 | 0,6687 |
| 16910478 | BOK antisense RNA 1 (non-protein coding) | BOK-AS1 | -0,14 | 0,0368 | -0,25 | 0,0593 | -0,25 | 0,1331 |
| 16768413 | decorin | DCN | -0,14 | 0,0178 | 0,01 | 0,8916 | -0,09 | 0,0606 |
| 16826016 | zinc finger protein 843 | ZNF843 | -0,14 | 0,0317 | 0,09 | 0,4751 | 0,14 | 0,4414 |
| 16949839 | mucin 20, cell surface associated | MUC20 | -0,14 | 0,0340 | -0,15 | 0,3121 | -0,21 | 0,2470 |
| 16708122 | cyclin M1 | CNNM1 | -0,14 | 0,0233 | -0,12 | 0,1637 | -0,02 | 0,8969 |
| 16881242 | olfactory receptor, family 7, subfamily E, member 91 pseudogene | OR7E91P | -0,14 | 0,0270 | 0,13 | 0,3998 | 0,07 | 0,7129 |
| 17090748 | surfeit 2 | SURF2 | -0,14 | 0,0281 | 0,05 | 0,4400 | 0,08 | 0,3196 |
| 17115850 | colony stimulating factor 2 receptor, alpha, low-affinity (granulocyte-macrophage) | CSF2RA | -0,14 | 0,0244 | 0,06 | 0,3401 | -0,06 | 0,4228 |
| 16863597 | DEAH (Asp-Glu-Ala-His) box polypeptide 34 | DHX34 | -0,14 | 0,0108 | -0,10 | 0,4183 | -0,05 | 0,5225 |
| 17006636 | nuclear factor of kappa light polypeptide gene enhancer in B-cells inhibitor-like 1 | NFKBIL1 | -0,14 | 0,0476 | -0,05 | 0,5980 | -0,16 | 0,2810 |
| 16836492 | proline rich 11 | PRR11 | -0,14 | 0,0344 | -0,08 | 0,1618 | -0,08 | 0,2840 |
| 17072577 | KIAA0196 antisense RNA 1 (non-protein coding) | KIAA0196-AS1 | -0,14 | 0,0127 | 0,00 | 0,9864 | -0,06 | 0,5105 |
| 16926111 | cystathionine-beta-synthase | CBS | -0,14 | 0,0355 | -0,07 | 0,3253 | -0,08 | 0,0510 |
| 16936287 | tubulin tyrosine ligase-like family, member 8 | TTLL8 | -0,14 | 0,0167 | -0,11 | 0,3128 | -0,03 | 0,7937 |
| 16671053 | late cornified envelope 4A | LCE4A | -0,14 | 0,0371 | -0,04 | 0,6472 | -0,14 | 0,0799 |
| 16781573 | angiogenin, ribonuclease, RNase A family, 5 \| ribonuclease, RNase A family, 4 | ANG\| RNASE4 | -0,14 | 0,0095 | -0,30 | 0,1999 | -0,04 | 0,7594 |
| 16780997 |  | RAB20 | -0,14 | 0,0470 | -0,16 | 0,1349 | -0,06 | 0,4863 |
| 16690535 | proline/serine-rich coiled-coil 1 | PSRC1 | -0,14 | 0,0492 | -0,10 | 0,5824 | -0,16 | 0,2598 |
| 17052734 | olfactory receptor, family 6, subfamily V, member 1 | OR6V1 | -0,14 | 0,0232 | -0,19 | 0,1254 | -0,08 | 0,5787 |
| 16695147 | olfactory receptor, family 10, subfamily J, member 5 | OR10J5 | -0,14 | 0,0045 | -0,03 | 0,5301 | 0,08 | 0,5869 |
| 16825800 | selenophosphate synthetase 2 | SEPHS2 | -0,14 | 0,0136 | -0,02 | 0,7307 | 0 | 0,9886 |
| 16940260 | neurobeachin-like 2 | NBEAL2 | -0,13 | 0,0270 | -0,17 | 0,2956 | -0,09 | 0,3704 |
| 16871418 | ATPase, H+/K+ exchanging, alpha polypeptide | ATP4A | -0,13 | 0,0476 | -0,12 | 0,0602 | -0,15 | 0,2948 |
| 16955489 | deoxyribonuclease I-like 3 | DNASE1L3 | -0,13 | 0,0095 | 0,08 | 0,4479 | -0,04 | 0,1986 |
| 16669850 | integrin, alpha 10 | ITGA10 | -0,13 | 0,0323 | -0,05 | 0,5945 | -0,09 | 0,1832 |
| 16826038 | cold shock domain protein A pseudogene 1 | CSDAP1 | -0,13 | 0,0328 | -0,15 | 0,3350 | -0,06 | 0,5192 |
| 16657485 | MT-ND2 pseudogene 28 \| NADH-ubiquinone oxidoreductase chain 2-like | MTND2P28\| LOC100653240 | -0,13 | 0,0268 | -0,02 | 0,9085 | -0,29 | 0,1391 |
| 16734469 | KCNQ1 antisense RNA 1 (non-protein coding) | KCNQ1-AS1 | -0,13 | 0,0494 | -0,08 | 0,2231 | -0,02 | 0,2966 |
| 17117762 |  | DKFZp761P0212 | -0,13 | 0,0160 | -0,12 | 0,1935 | -0,1 | 0,2529 |
| 16928011 | chromosome 22 open reading frame 15 | C22orf15 | -0,13 | 0,0226 | -0,03 | 0,6587 | -0,1 | 0,4371 |
| 17066203 | nervous system abundant protein 11 | NSAP11 | -0,13 | 0,0480 | -0,28 | 0,1178 | -0,13 | 0,3099 |
| 16905238 | WAS/WASL interacting protein family, member 1 | WIPF1 | -0,13 | 0,0109 | 0,10 | 0,1518 | -0,07 | 0,1625 |
| 16761628 | loss of heterozygosity, 12, chromosomal region 2 (non-protein coding) | LOH12CR2 | -0,13 | 0,0496 | -0,13 | 0,3915 | -0,17 | 0,1730 |
| 16744358 | ferredoxin-fold anticodon binding domain containing 1 \| asparagine-linked glycosylation 9, alpha-1,2-mannosyltransferase homolog (S. cerevisiae) | FDXACB1\| ALG9 | -0,13 | 0,0026 | -0,11 | 0,1978 | 0,02 | 0,9080 |
| 16857110 | ankyrin repeat domain 24 | ANKRD24 | -0,13 | 0,0004 | -0,33 | 0,3334 | 0,12 | 0,3395 |
| 16729924 | N-acetylated alpha-linked acidic dipeptidase 2 | NAALAD2 | -0,13 | 0,0314 | -0,10 | 0,1614 | -0,1 | 0,4169 |
| 16778274 | transient receptor potential cation channel, subfamily C, member 4 | TRPC4 | -0,13 | 0,0047 | -0,04 | 0,3225 | -0,12 | 0,2357 |
| 17084650 | ATPase, class I, type 8B, member 5, pseudogene | ATP8B5P | -0,13 | 0,0234 | 0,02 | 0,7634 | -0,08 | 0,4946 |
| 17027695 | HLA complex group 22 (non-protein coding) | HCG22 | -0,13 | 0,0295 | -0,05 | 0,7451 | -0,11 | 0,6183 |
| 16934171 | chromosome 22 open reading frame 42 | C22orf42 | -0,13 | 0,0064 | -0,07 | 0,7483 | -0,11 | 0,1702 |
| 17061415 | LHFPL3 antisense RNA 2 (non-protein coding) | LHFPL3-AS2 | -0,13 | 0,0487 | 0,02 | 0,8462 | -0,1 | 0,1919 |
| 16970363 | adenosine deaminase domain containing 1 (testis-specific) | ADAD1 | -0,13 | 0,0085 | -0,04 | 0,5654 | -0,07 | 0,4029 |
| 16727650 | ras homolog family member D | RHOD | -0,13 | 0,0157 | -0,06 | 0,5332 | -0,12 | 0,0652 |
| 17008463 | natural cytotoxicity triggering receptor 2 | NCR2 | -0,12 | 0,0162 | -0,04 | 0,6624 | -0,21 | 0,1007 |
| 17074361 | defensin, beta 4B \| defensin, beta 4A | DEFB4B\| DEFB4A | -0,12 | 0,0215 | 0,01 | 0,9324 | -0,02 | 0,7504 |
| 17019912 | defensin, beta 114 | DEFB114 | -0,12 | 0,0340 | 0,02 | 0,8976 | 0,13 | 0,4264 |
| 16961374 | actin-related protein T3 | ACTRT3 | -0,12 | 0,0275 | 0,15 | 0,5191 | -0,07 | 0,2974 |
| 16797559 | immunoglobulin heavy variable 3-49 | IGHV3-49 | -0,12 | 0,0236 | -0,02 | 0,8006 | 0,05 | 0,7986 |
| 16998974 | TRIM36 intronic transcript 1 (non-protein coding) | TRIM36-IT1 | -0,12 | 0,0271 | -0,04 | 0,6876 | -0,04 | 0,7723 |
| 16755223 | keratin 19 pseudogene 2 \| microRNA 492 | KRT19P2\| MIR492 | -0,12 | 0,0323 | -0,02 | 0,8216 | 0 | 0,9931 |
| 17098293 | nuclear receptor subfamily 5, group A, member 1 | NR5A1 | -0,12 | 0,0116 | 0,00 | 0,9911 | -0,18 | 0,3080 |
| 16693173 | mitochondrial ribosomal protein L9 | MRPL9 | -0,12 | 0,0222 | -0,04 | 0,2294 | -0,07 | 0,2859 |
| 16849827 |  | DKFZp761P0212 | -0,12 | 0,0184 | -0,11 | 0,0963 | -0,09 | 0,2179 |
| 16702068 | calmodulin-like 3 | CALML3 | -0,12 | 0,0361 | -0,02 | 0,8995 | -0,17 | 0,2476 |
| 17087610 | muscle-related coiled-coil protein | MURC | -0,12 | 0,0028 | -0,06 | 0,2877 | -0,04 | 0,5891 |
| 17032367 | chromosome 6 open reading frame 10 | C6orf10 | -0,12 | 0,0069 | -0,11 | 0,3386 | -0,18 | 0,1321 |
| 16728731 | autophagy related 16-like 2 (S. cerevisiae) | ATG16L2 | -0,12 | 0,0380 | -0,10 | 0,4176 | -0,03 | 0,8226 |
| 17095440 | chromosome 9 open reading frame 153 | C9orf153 | -0,12 | 0,0384 | 0,00 | 0,9920 | -0,08 | 0,0895 |
| 17012144 | sialic acid binding Ig-like lectin, pseudogene 3 | SIGLECP3 | -0,12 | 0,0009 | -0,16 | 0,2029 | -0,12 | 0,4320 |
| 16897450 | neurexin 1 | NRXN1 | -0,12 | 0,0164 | -0,09 | 0,3670 | -0,2 | 0,0946 |
| 16764232 | desert hedgehog | DHH | -0,12 | 0,0377 | -0,08 | 0,3538 | 0,09 | 0,2903 |
| 17017748 | 1-acylglycerol-3-phosphate O-acyltransferase 1 (lysophosphatidic acid acyltransferase, alpha) \| PPT2-EGFL8 readthrough | AGPAT1\| PPT2-EGFL8 | -0,12 | 0,0167 | -0,20 | 0,0551 | -0,26 | 0,2153 |
| 17058582 | abhydrolase domain containing 11 | ABHD11 | -0,12 | 0,0343 | -0,17 | 0,1076 | -0,04 | 0,5668 |
| 17055786 | interleukin 6 (interferon, beta 2) \| uncharacterized LOC541472 | IL6\| LOC541472 | -0,12 | 0,0472 | 0,06 | 0,5297 | 0,01 | 0,7952 |
| 16767364 | lysozyme | LYZ | -0,12 | 0,0388 | -0,14 | 0,1387 | -0,14 | 0,1315 |
| 17024317 | neuromedin B receptor | NMBR | -0,12 | 0,0181 | -0,01 | 0,9431 | -0,11 | 0,0726 |
| 17038622 | major histocompatibility complex, class II, DQ beta 1 \| HLA class II histocompatibility antigen, DQ beta 1 chain-like | HLA-DQB1\| LOC100293977 | -0,11 | 0,0176 | 0,05 | 0,1092 | -0,12 | 0,1741 |
| 16817824 | coronin, actin binding protein, 1A | CORO1A | -0,11 | 0,0046 | -0,14 | 0,1217 | 0,08 | 0,6909 |
| 16838855 | hepatocyte growth factor-regulated tyrosine kinase substrate | HGS | -0,11 | 0,0391 | -0,16 | 0,1403 | -0,06 | 0,5717 |
| 16847296 |  | TBC1D3P1-DHX40P1 | -0,11 | 0,0459 | -0,15 | 0,0910 | -0,04 | 0,6553 |
| 16975247 | kelch-like 5 (Drosophila) | KLHL5 | -0,11 | 0,0392 | -0,08 | 0,2667 | -0,1 | 0,0794 |
| 16708229 | dynamin binding protein \| DNMBP antisense RNA 1 (non-protein coding) | DNMBP\| DNMBP-AS1 | -0,11 | 0,0259 | -0,15 | 0,1010 | -0,12 | 0,1644 |
| 16920617 | CCCTC-binding factor (zinc finger protein)-like | CTCFL | -0,11 | 0,0157 | -0,01 | 0,9585 | -0,06 | 0,7749 |
| 16666819 | chloride channel accessory 3, pseudogene | CLCA3P | -0,11 | 0,0272 | 0,04 | 0,3207 | -0,05 | 0,1221 |
| 16797841 | putative golgin subfamily A member 6-like protein 6-like | LOC100134285 | -0,11 | 0,0387 | 0,07 | 0,7401 | 0,03 | 0,8092 |
| 16692311 | flavin containing monooxygenase 5 | FMO5 | -0,11 | 0,0104 | -0,04 | 0,7566 | -0,05 | 0,5981 |
| 17064512 | WD repeat domain 86 \| uncharacterized LOC100507673 | WDR86\| LOC100507673 | -0,11 | 0,0332 | -0,07 | 0,0636 | 0,01 | 0,8256 |
| 16704040 | cyclin Y-like pseudogene | LOC100129055 | -0,10 | 0,0020 | 0,07 | 0,5857 | -0,16 | 0,2307 |
| 16862849 | serine/arginine repetitive matrix 5 \| zinc finger protein 428 | SRRM5\| ZNF428 | -0,10 | 0,0223 | -0,08 | 0,4831 | 0,07 | 0,4574 |
| 16994038 | programmed cell death 6 pseudogene | LOC728613 | -0,10 | 0,0449 | -0,14 | 0,1297 | -0,22 | 0,3056 |
| 17034777 | proteasome (prosome, macropain) subunit, beta type, 8 (large multifunctional peptidase 7) | PSMB8 | -0,10 | 0,0040 | -0,10 | 0,1855 | -0,02 | 0,0558 |
| 16926610 | formiminotransferase cyclodeaminase | FTCD | -0,10 | 0,0096 | -0,02 | 0,8285 | 0,01 | 0,8340 |
| 16711695 | surfactant associated 1, pseudogene | SFTA1P | -0,10 | 0,0450 | 0,02 | 0,8194 | -0,07 | 0,5801 |
| 16726020 | cholinergic receptor, muscarinic 1 | CHRM1 | -0,10 | 0,0253 | -0,07 | 0,5387 | -0,16 | 0,2748 |
| 16729444 | chromosome 11 open reading frame 67 | C11orf67 | -0,10 | 0,0421 | 0,01 | 0,7347 | -0,02 | 0,9146 |
| 16743926 | caspase recruitment domain family, member 18 | CARD18 | -0,10 | 0,0465 | 0,00 | 0,9975 | -0,02 | 0,7337 |
| 17037257 | proteasome (prosome, macropain) subunit, beta type, 8 (large multifunctional peptidase 7) | PSMB8 | -0,10 | 0,0041 | -0,10 | 0,2479 | 0,01 | 0,3876 |
| 16781448 | olfactory receptor, family 4, subfamily K, member 5 | OR4K5 | -0,10 | 0,0084 | 0,00 | 0,9946 | 0,02 | 0,8429 |
| 16803144 | golgin A6 family, member A \| golgin A6 family, member B \| golgin A6 family, member D \| golgin A6 family, member C \| golgin A6 family, member A pseudogene | GOLGA6A\| GOLGA6B\| GOLGA6D\| GOLGA6C\| LOC645752 | -0,10 | 0,0429 | 0,01 | 0,9315 | -0,01 | 0,9511 |
| 16968938 | glutamate receptor, ionotropic, delta 2 | GRID2 | -0,10 | 0,0187 | -0,02 | 0,7064 | -0,14 | 0,2281 |
| 16984905 | interleukin 31 receptor A | IL31RA | -0,10 | 0,0291 | -0,05 | 0,1461 | -0,1 | 0,2283 |
| 16976706 | ADAM metallopeptidase with thrombospondin type 1 motif, 3 | ADAMTS3 | -0,10 | 0,0230 | 0,04 | 0,7063 | -0,1 | 0,4133 |
| 16955368 | dynein, axonemal, heavy chain 12 | DNAH12 | -0,10 | 0,0008 | -0,12 | 0,0531 | -0,12 | 0,2375 |
| 16874237 | histidine rich calcium binding protein | HRC | -0,10 | 0,0108 | -0,04 | 0,6552 | -0,25 | 0,1370 |
| 17091905 | WAS protein family homolog 1 \| WAS protein family homolog 2 pseudogene \| WAS protein family homolog 7 pseudogene \| WAS protein family homolog 5 pseudogene \| WAS protein family homolog 3 pseudogene \| WAS protein family homolog 1 pseudogene \| WAS protein family homolog 4 pseudogene \| WAS protein family homolog 6 pseudogene | WASH1\| WASH2P\| WASH7P\| WASH5P\| WASH3P\| LOC100288778\| WASH4P\| WASH6P | -0,10 | 0,0451 | -0,14 | 0,2592 | -0,14 | 0,0801 |
| 16935274 | ENTH domain containing 1 | ENTHD1 | -0,10 | 0,0004 | 0,02 | 0,8192 | -0,1 | 0,3361 |
| 16982161 | PDZ and LIM domain 3 | PDLIM3 | -0,09 | 0,0115 | -0,13 | 0,1799 | -0,23 | 0,1828 |
| 16871880 | WD repeat domain 87 | WDR87 | -0,09 | 0,0085 | -0,08 | 0,2904 | -0,07 | 0,2981 |
| 16729095 | trophoblast glycoprotein-like | TPBGL | -0,09 | 0,0006 | -0,17 | 0,2521 | -0,14 | 0,0660 |
| 17076285 | RAB11 family interacting protein 1 (class I) | RAB11FIP1 | -0,09 | 0,0045 | -0,15 | 0,3020 | -0,07 | 0,6283 |
| 16998293 | glutaredoxin (thioltransferase) | GLRX | -0,09 | 0,0090 | -0,04 | 0,5238 | -0,04 | 0,3111 |
| 17042473 | proteasome (prosome, macropain) subunit, beta type, 8 (large multifunctional peptidase 7) | PSMB8 | -0,09 | 0,0042 | -0,10 | 0,1459 | -0,02 | 0,4279 |
| 17033639 | HLA class II histocompatibility antigen, DQ beta 1 chain-like \| major histocompatibility complex, class II, DQ beta 1 \| HLA-DQB1 antisense RNA 1 (non-protein coding) | LOC100293977\| HLA-DQB1\| HLA-DQB1-AS1 | -0,09 | 0,0379 | -0,07 | 0,2588 | -0,16 | 0,3167 |
| 17067890 | zinc finger protein 703 | ZNF703 | -0,09 | 0,0155 | -0,15 | 0,0870 | -0,12 | 0,2308 |
| 16799273 | chromosome 15 open reading frame 53 | C15orf53 | -0,09 | 0,0185 | -0,03 | 0,7834 | 0,03 | 0,8826 |
| 16865276 | tweety homolog 1 (Drosophila) | TTYH1 | -0,09 | 0,0404 | -0,16 | 0,1477 | -0,07 | 0,0923 |
| 17101554 | unknown transcript | LOC100093698 | -0,09 | 0,0302 | 0,01 | 0,7267 | -0,05 | 0,3655 |
| 16664218 | tetraspanin 1 | TSPAN1 | -0,09 | 0,0246 | -0,23 | 0,1825 | 0,02 | 0,2085 |
| 17097464 | haloacid dehalogenase-like hydrolase domain containing 3 | HDHD3 | -0,09 | 0,0257 | -0,10 | 0,5918 | -0,1 | 0,4087 |
| 17029774 | proteasome (prosome, macropain) subunit, beta type, 8 (large multifunctional peptidase 7) | PSMB8 | -0,09 | 0,0068 | -0,09 | 0,1519 | -0,01 | 0,4670 |
| 17107159 | cancer/testis antigen family 45, member A1 | CT45A1 | -0,09 | 0,0224 | -0,09 | 0,1766 | -0,14 | 0,2474 |
| 16988798 | hCG1981531 | LOC728586 | -0,09 | 0,0232 | -0,08 | 0,1702 | -0,09 | 0,3677 |
| 16680510 | chromosome 1 open reading frame 222 \| KIAA1751 | C1orf222\| KIAA1751 | -0,09 | 0,0478 | -0,10 | 0,3359 | -0,09 | 0,3601 |
| 16962085 | kelch-like 6 (Drosophila) | KLHL6 | -0,09 | 0,0392 | 0,02 | 0,8277 | 0,01 | 0,9409 |
| 16684686 | MARCKS-like 1 | MARCKSL1 | -0,09 | 0,0208 | -0,02 | 0,8145 | -0,06 | 0,5174 |
| 17098115 | olfactory receptor, family 1, subfamily J, member 1 | OR1J1 | -0,09 | 0,0062 | -0,03 | 0,8637 | -0,04 | 0,7692 |
| 16931990 | ATPase, H+ transporting, lysosomal 31kDa, V1 subunit E1 | ATP6V1E1 | -0,09 | 0,0233 | 0,00 | 0,9531 | -0,06 | 0,2783 |
| 16685769 | v-myc myelocytomatosis viral oncogene homolog 1, lung carcinoma derived (avian) | MYCL1 | -0,09 | 0,0377 | -0,08 | 0,3328 | -0,07 | 0,5070 |
| 16695974 | glycoprotein A33 (transmembrane) | GPA33 | -0,08 | 0,0406 | 0,14 | 0,3586 | 0 | 0,8962 |
| 16976833 | chemokine (C-X-C motif) ligand 3 | CXCL3 | -0,08 | 0,0496 | -0,03 | 0,8873 | -0,06 | 0,5089 |
| 17106933 |  | RAB33A | -0,08 | 0,0457 | 0,04 | 0,1231 | 0,02 | 0,7322 |
| 17089640 | cerebral endothelial cell adhesion molecule \| uncharacterized LOC100506065 | CERCAM\| LOC100506065 | -0,08 | 0,0341 | -0,03 | 0,6982 | -0,09 | 0,1124 |
| 16839331 | tyrosine 3-monooxygenase/tryptophan 5-monooxygenase activation protein, epsilon polypeptide | YWHAE | -0,08 | 0,0064 | -0,08 | 0,2866 | -0,14 | 0,3038 |
| 17039963 | proteasome (prosome, macropain) subunit, beta type, 8 (large multifunctional peptidase 7) | PSMB8 | -0,08 | 0,0091 | -0,08 | 0,1850 | 0 | 0,8690 |
| 17009461 | opsin 5 | OPN5 | -0,08 | 0,0200 | 0,04 | 0,7892 | 0,06 | 0,6935 |
| 16670547 | threonyl-tRNA synthetase 2, mitochondrial (putative) | TARS2 | -0,08 | 0,0069 | -0,01 | 0,8886 | -0,08 | 0,5824 |
| 17034570 | 1-acylglycerol-3-phosphate O-acyltransferase 1 (lysophosphatidic acid acyltransferase, alpha) | AGPAT1 | -0,08 | 0,0433 | -0,17 | 0,0530 | -0,26 | 0,2616 |
| 16838367 | transmembrane protein 235 | TMEM235 | -0,08 | 0,0050 | -0,11 | 0,1230 | -0,08 | 0,1775 |
| 16923656 | keratin associated protein 10-8 | KRTAP10-8 | -0,08 | 0,0190 | 0,03 | 0,7673 | -0,16 | 0,4144 |
| 16759539 | fibrosin-like 1 | FBRSL1 | -0,08 | 0,0188 | -0,17 | 0,4339 | -0,08 | 0,3458 |
| 16947816 | sterile alpha motif domain containing 7 | SAMD7 | -0,08 | 0,0092 | 0,04 | 0,7498 | -0,05 | 0,4115 |
| 16831644 | leucine rich repeat containing 48 | LRRC48 | -0,08 | 0,0428 | -0,12 | 0,4316 | -0,05 | 0,5053 |
| 16871943 | RAS guanyl releasing protein 4 | RASGRP4 | -0,08 | 0,0390 | -0,09 | 0,3757 | -0,03 | 0,0561 |
| 16965983 | pituitary tumor-transforming 2 | PTTG2 | -0,07 | 0,0478 | -0,03 | 0,7194 | 0,02 | 0,8238 |
| 16770915 | WD repeat and SOCS box containing 2 | WSB2 | -0,07 | 0,0442 | -0,07 | 0,2137 | -0,09 | 0,2225 |
| 16756627 | uracil-DNA glycosylase | UNG | -0,07 | 0,0276 | -0,07 | 0,5662 | -0,03 | 0,5478 |
| 17099484 | mediator complex subunit 22 | MED22 | -0,07 | 0,0329 | -0,04 | 0,6554 | -0,01 | 0,6408 |
| 17047760 | MAGI2 antisense RNA 3 (non-protein coding) | MAGI2-AS3 | -0,07 | 0,0123 | 0,09 | 0,6029 | -0,02 | 0,8991 |
| 16856510 | chromosome 19 open reading frame 24 | C19orf24 | -0,07 | 0,0248 | -0,06 | 0,7125 | -0,06 | 0,6541 |
| 16707551 | centrosomal protein 55kDa | CEP55 | -0,07 | 0,0473 | -0,11 | 0,1708 | 0,01 | 0,8415 |
| 16803794 | chromosome 15 open reading frame 26 | C15orf26 | -0,07 | 0,0356 | 0,07 | 0,2943 | 0,01 | 0,8928 |
| 16843158 | oligodendrocyte myelin glycoprotein | OMG | -0,07 | 0,0368 | -0,01 | 0,8148 | -0,07 | 0,2791 |
| 16981913 | RWD domain containing 4 \| RWD domain containing 4 pseudogene 2 \| RWD domain containing 4 pseudogene 1 | RWDD4\| RWDD4P2\| RWDD4P1 | -0,07 | 0,0328 | -0,13 | 0,4671 | -0,11 | 0,2198 |
| 17056827 | T cell receptor gamma variable 11 (non-functional) | TRGV11 | -0,07 | 0,0497 | -0,21 | 0,3612 | -0,08 | 0,5663 |
| 16964948 | G protein-coupled receptor 78 | GPR78 | -0,07 | 0,0493 | 0,05 | 0,6759 | -0,03 | 0,7289 |
| 16710927 | polyamine oxidase (exo-N4-amino) \| mitochondrial GTPase 1 homolog (S. cerevisiae) | PAOX\| MTG1 | -0,07 | 0,0452 | -0,01 | 0,9438 | -0,09 | 0,5595 |
| 17020838 |  | CD109 | -0,06 | 0,0084 | 0,08 | 0,2441 | 0,05 | 0,2539 |
| 16804694 | zinc finger protein 710 | ZNF710 | -0,06 | 0,0340 | -0,03 | 0,2374 | 0 | 0,9051 |
| 16716507 | protein phosphatase 1, regulatory subunit 3C | PPP1R3C | -0,06 | 0,0319 | 0,01 | 0,9226 | -0,12 | 0,2690 |
| 17044491 | HOXA cluster antisense RNA 2 (non-protein coding) | HOXA-AS2 | -0,06 | 0,0488 | -0,04 | 0,4174 | -0,1 | 0,1610 |
| 16993027 | MAX dimerization protein 3 \| PRELI domain containing 1 | MXD3\| PRELID1 | -0,06 | 0,0112 | -0,01 | 0,8661 | -0,03 | 0,4818 |
| 16752834 | serine hydroxymethyltransferase 2 (mitochondrial) | SHMT2 | -0,06 | 0,0385 | -0,10 | 0,4446 | -0,1 | 0,1840 |
| 16967523 | ameloblastin (enamel matrix protein) | AMBN | -0,06 | 0,0089 | -0,05 | 0,7868 | 0,04 | 0,8157 |
| 16825956 | vitamin K epoxide reductase complex, subunit 1 | VKORC1 | -0,06 | 0,0306 | -0,09 | 0,4926 | -0,06 | 0,1657 |
| 16996917 |  | TAF9 | -0,06 | 0,0293 | -0,02 | 0,7820 | -0,02 | 0,8560 |
| 17112550 | PABPC5 antisense RNA 1 (non-protein coding) | PABPC5-AS1 | -0,06 | 0,0099 | -0,06 | 0,4711 | -0,13 | 0,1994 |
| 17068254 | ADAM metallopeptidase domain 5, pseudogene | ADAM5P | -0,05 | 0,0464 | -0,01 | 0,4434 | -0,03 | 0,0575 |
| 16897637 | reticulon 4 | RTN4 | -0,05 | 0,0326 | -0,03 | 0,3848 | -0,03 | 0,4816 |
| 17037746 |  | TRIM39-RPP21 | -0,05 | 0,0293 | -0,10 | 0,2494 | -0,03 | 0,5435 |
| 16910154 | uncharacterized LOC100130449 | PP14571 | -0,05 | 0,0216 | 0,01 | 0,6364 | -0,01 | 0,8282 |
| 16926922 | peroxisomal biogenesis factor 26 | PEX26 | -0,05 | 0,0119 | -0,06 | 0,3127 | -0,07 | 0,3298 |
| 17074673 | cathepsin B | CTSB | -0,04 | 0,0077 | -0,14 | 0,2341 | -0,07 | 0,2640 |
| 17105321 | tenomodulin | TNMD | -0,04 | 0,0184 | 0,14 | 0,2171 | -0,06 | 0,5403 |
| 16945474 | collagen, type VI, alpha 4 pseudogene 2 | COL6A4P2 | -0,04 | 0,0359 | -0,01 | 0,9171 | 0,04 | 0,1071 |
| 16665757 | phosphodiesterase 4B, cAMP-specific | PDE4B | -0,04 | 0,0361 | 0,17 | 0,0931 | 0,05 | 0,4173 |
| 16914414 | solute carrier family 12 (potassium/chloride transporter), member 5 | SLC12A5 | -0,04 | 0,0129 | -0,02 | 0,8514 | -0,06 | 0,4079 |
| 16714433 | protocadherin-related 15 | PCDH15 | -0,03 | 0,0250 | -0,01 | 0,9256 | -0,1 | 0,2701 |
| 16857630 | patatin-like phospholipase domain containing 6 | PNPLA6 | -0,03 | 0,0461 | -0,16 | 0,2061 | -0,02 | 0,6583 |
| 16864778 | protein phosphatase 2, regulatory subunit A, alpha | PPP2R1A | -0,03 | 0,0179 | 0,02 | 0,4707 | -0,02 | 0,5896 |
| 16690090 | leucine rich repeat containing 39 | LRRC39 | -0,02 | 0,0255 | 0,06 | 0,6133 | -0,04 | 0,2762 |

^1^From Student’s t-test

**Table D. Pathways downregulated in response to double S6K1/S6K2 siRNA, but not to single siRNA.**

| p-value | Term | Term ID | Term description | Genes |
| --- | --- | --- | --- | --- |
| 6.28e-03 | BIOGRID:00000 | bi | BioGRID interaction data | MBP, TCEA2, PSG9, ARHGEF1, MYO1G, GPC5, P2RY8, KYNU, SLC2A2, TUBB2A, CXXC4, ARHGDIB, S100A8, ZNF528, ZG16, STMN2, FCER1G, TPM2, ASL, NELL1, PPM1E, KCNAB3, SLC6A4, LOC100130000, KRT10, AQP11, HIST1H2BO, PCYT2, ZBTB20, C19ORF43, DUSP2, ATP6V0D2, GNRH2, COX4I2, CRYAA, SUSD3, C21ORF2, NR4A2 |
| 2.38e-02 | CORUM:527 | co | TRPC3-TRPC4 channel complex, redox-sensitive | TRPC3, TRPC4 |
