# Supplementary material for: Revealing Different Roles of the mTOR-Targets S6K1 and S6K2 in Breast Cancer by Expression Profiling and Structural Analysis
Source: PLoS One. 2015 Dec 23;10(12):e0145013. doi: 10.1371/journal.pone.0145013 (PMC4689523; doi:10.1371/journal.pone.0145013)
Supplement: S10 Table — Genes upregulated in response to S6K1 siRNA, but not to S6K2 siRNA (Table A). Pathways upregulated in response to S6K1 siRNA, but not to S6K2 siRNA (Table B). Genes downregulated in response to S6K1 siRNA, but not to S6K2 siRNA (Table C). Pathways significantly downregulated in response to S6K1 siRNA, but not to S6K2 siRNA (Table D). (DOCX) [file pone.0145013.s014.docx]

**Table A. Genes upregulated in response to S6K1 siRNA, but not to S6K2 siRNA.**

| Transcripts Cluster ID | Gene description | Gene symbol | S6K1 siRNA  Fold change | S6K1 siRNA  p-value^1^ | S6K2 siRNA  Fold change | S6K2 siRNA  p-value^1^ |
| --- | --- | --- | --- | --- | --- | --- |
| 16965606 | solute carrier family 34 (sodium phosphate), member 2 | SLC34A2 | 0,59 | 0,00 | -0,07 | 0,70 |
| 16721053 | RNA, U7 small nuclear 50 pseudogene | RNU7-50P | 0,54 | 0,04 | 0,21 | 0,60 |
| 16698234 | fibromodulin | FMOD | 0,51 | 0,02 | 0,28 | 0,19 |
| 16674845 | laminin, gamma 2 | LAMC2 | 0,49 | 0,01 | 0,25 | 0,12 |
| 16682402 | peptidyl arginine deiminase, type II | PADI2 | 0,48 | 0,02 | 0,15 | 0,14 |
| 17045354 | Yae1 domain containing 1 | YAE1D1 | 0,47 | 0,04 | 0,34 | 0,09 |
| 16888047 | alkylglycerone phosphate synthase | AGPS | 0,46 | 0,03 | 0,19 | 0,06 |
| 16723400 | homeodomain interacting protein kinase 3 | HIPK3 | 0,46 | 0,00 | 0,50 | 0,05 |
| 17106102 | collagen, type IV, alpha 5 | COL4A5 | 0,45 | 0,04 | 0,15 | 0,10 |
| 16978376 | EMCN intronic transcript 3 (non-protein coding) | EMCN-IT3 | 0,44 | 0,01 | 0,06 | 0,64 |
| 17011185 | thiosulfate sulfurtransferase (rhodanese)-like domain containing 3 | TSTD3 | 0,43 | 0,01 | 0,17 | 0,29 |
| 16880527 | UDP-GlcNAc:betaGal beta-1,3-N-acetylglucosaminyltransferase 2 | B3GNT2 | 0,42 | 0,03 | 0,22 | 0,25 |
| 16845537 | histone deacetylase 5 | HDAC5 | 0,42 | 0,02 | 0,27 | 0,09 |
| 16725101 | RNA, U7 small nuclear 58 pseudogene | RNU7-58P | 0,42 | 0,04 | -0,03 | 0,73 |
| 16850923 |  | RAB31 | 0,41 | 0,04 | 0,06 | 0,19 |
| 16920585 | bone morphogenetic protein 7 | BMP7 | 0,40 | 0,04 | 0,26 | 0,16 |
| 16708192 | ATP-binding cassette, sub-family C (CFTR/MRP), member 2 | ABCC2 | 0,40 | 0,00 | 0,30 | 0,12 |
| 16777996 |  | CG030 | 0,40 | 0,01 | 0,22 | 0,16 |
| 17031378 | gamma-aminobutyric acid (GABA) B receptor, 1 | GABBR1 | 0,40 | 0,02 | 0,18 | 0,10 |
| 16898548 | F-box protein 48 | FBXO48 | 0,39 | 0,04 | 0,36 | 0,08 |
| 17052306 | aarF domain containing kinase 2 | ADCK2 | 0,39 | 0,01 | 0,23 | 0,23 |
| 17014459 | plasminogen | PLG | 0,39 | 0,04 | 0,11 | 0,75 |
| 16800888 | galactokinase 2 | GALK2 | 0,38 | 0,04 | 0,10 | 0,68 |
| 16882861 | prominin 2 | PROM2 | 0,38 | 0,05 | 0,37 | 0,14 |
| 16878443 | FOS-like antigen 2 | FOSL2 | 0,38 | 0,02 | 0,13 | 0,13 |
| 17051553 | carboxypeptidase A4 | CPA4 | 0,38 | 0,02 | 0,15 | 0,24 |
| 17104484 | kinesin family member 4A | KIF4A | 0,38 | 0,01 | 0,12 | 0,19 |
| 16984141 | WD repeat domain 70 | WDR70 | 0,38 | 0,01 | 0,38 | 0,06 |
| 17050645 | suppression of tumorigenicity 7 \| ST7 overlapping transcript 3 (non-protein coding) | ST7\| ST7-OT3 | 0,37 | 0,04 | 0,36 | 0,10 |
| 17064299 | potassium voltage-gated channel, subfamily H (eag-related), member 2 | KCNH2 | 0,36 | 0,03 | 0,32 | 0,13 |
| 17085040 | tRNA methyltransferase 10 homolog B (S. cerevisiae) | TRMT10B | 0,36 | 0,02 | 0,22 | 0,19 |
| 17117458 | uncharacterized LOC100130249 | PP2672 | 0,36 | 0,05 | 0,14 | 0,13 |
| 16871305 | zinc finger protein 792 | ZNF792 | 0,36 | 0,02 | 0,33 | 0,11 |
| 16746546 | beta-1,4-N-acetyl-galactosaminyl transferase 3 | B4GALNT3 | 0,35 | 0,03 | 0,36 | 0,15 |
| 16754536 | synaptotagmin I | SYT1 | 0,35 | 0,03 | 0,08 | 0,58 |
| 16777401 | mitochondrial intermediate peptidase | MIPEP | 0,35 | 0,03 | 0,26 | 0,07 |
| 16695268 | immunoglobulin superfamily, member 8 | IGSF8 | 0,35 | 0,04 | 0,14 | 0,46 |
| 16903427 | origin recognition complex, subunit 4 | ORC4 | 0,35 | 0,04 | 0,39 | 0,09 |
| 17085901 | annexin A1 | ANXA1 | 0,34 | 0,03 | -0,02 | 0,81 |
| 16942935 | zinc finger protein 654 | ZNF654 | 0,34 | 0,04 | 0,27 | 0,06 |
| 16819229 | metallothionein 1J, pseudogene | MT1JP | 0,34 | 0,02 | 0,51 | 0,06 |
| 17076273 | chromosome X open reading frame 56 pseudogene | LOC728024 | 0,34 | 0,02 | 0,38 | 0,09 |
| 16666851 | heparan sulfate 2-O-sulfotransferase 1 \| uncharacterized LOC339524 | HS2ST1\| LOC339524 | 0,34 | 0,02 | 0,23 | 0,06 |
| 16866008 | zinc finger protein 304 | ZNF304 | 0,33 | 0,04 | 0,19 | 0,09 |
| 16889115 | coenzyme Q10 homolog B (S. cerevisiae) | COQ10B | 0,33 | 0,05 | 0,26 | 0,06 |
| 16976908 | ring finger and CHY zinc finger domain containing 1, E3 ubiquitin protein ligase | RCHY1 | 0,33 | 0,04 | 0,37 | 0,07 |
| 16827735 | pyruvate dehydrogenase phosphatase regulatory subunit \| uncharacterized LOC400541 | PDPR\| LOC400541 | 0,33 | 0,00 | 0,08 | 0,66 |
| 16929855 | lectin, galactoside-binding, soluble, 1 | LGALS1 | 0,33 | 0,03 | 0,10 | 0,44 |
| 16866686 | ADAMTS-like 5 | ADAMTSL5 | 0,33 | 0,01 | 0,19 | 0,10 |
| 16730845 | ataxia telangiectasia mutated \| nuclear protein, ataxia-telangiectasia locus | ATM\| NPAT | 0,33 | 0,01 | 0,29 | 0,06 |
| 16917291 | taspase, threonine aspartase, 1 | TASP1 | 0,33 | 0,03 | 0,32 | 0,07 |
| 16855786 | thioredoxin-related transmembrane protein 3 | TMX3 | 0,32 | 0,04 | 0,24 | 0,08 |
| 17025177 | dynein, light chain, Tctex-type 1 | DYNLT1 | 0,32 | 0,02 | 0,15 | 0,37 |
| 16847393 | small Cajal body-specific RNA 20 | SCARNA20 | 0,32 | 0,01 | 0,24 | 0,37 |
| 17117893 | eukaryotic translation initiation factor 4E family member 2 | EIF4E2 | 0,32 | 0,01 | 0,16 | 0,52 |
| 16698407 | leucine rich repeat neuronal 2 | LRRN2 | 0,32 | 0,04 | 0,31 | 0,18 |
| 17051409 | adenosylhomocysteinase-like 2 | AHCYL2 | 0,32 | 0,00 | 0,15 | 0,07 |
| 16670492 | mitochondrial ribosomal protein S21 | MRPS21 | 0,31 | 0,04 | 0,08 | 0,52 |
| 16728141 | galanin prepropeptide | GAL | 0,31 | 0,04 | 0,41 | 0,10 |
| 16719217 | carbohydrate (N-acetylgalactosamine 4-sulfate 6-O) sulfotransferase 15 | CHST15 | 0,31 | 0,01 | 0,34 | 0,10 |
| 17060923 | RAB, member RAS oncogene family-like 5 | RABL5 | 0,31 | 0,03 | 0,29 | 0,18 |
| 16755542 | apoptotic peptidase activating factor 1 | APAF1 | 0,31 | 0,04 | 0,29 | 0,07 |
| 17106913 | BCL6 corepressor-like 1 | BCORL1 | 0,31 | 0,01 | -0,02 | 0,89 |
| 16736379 | secretion regulating guanine nucleotide exchange factor | SERGEF | 0,31 | 0,03 | 0,31 | 0,29 |
| 16993878 | solute carrier family 12 (potassium/chloride transporters), member 7 | SLC12A7 | 0,31 | 0,01 | 0,29 | 0,10 |
| 16661255 | AT rich interactive domain 1A (SWI-like) | ARID1A | 0,30 | 0,04 | 0,29 | 0,07 |
| 17050002 | myeloid/lymphoid or mixed-lineage leukemia 5 (trithorax homolog, Drosophila) | MLL5 | 0,30 | 0,02 | 0,31 | 0,16 |
| 17079633 | ankyrin repeat domain 46 \| glyceraldehyde 3 phosphate dehydrogenase pseudogene 62 | ANKRD46\| GAPDHP62 | 0,30 | 0,03 | 0,29 | 0,10 |
| 16753299 | ubiquitin specific peptidase 15 | USP15 | 0,30 | 0,01 | 0,31 | 0,10 |
| 17052413 | taste receptor, type 2, member 3 | TAS2R3 | 0,30 | 0,04 | 0,19 | 0,05 |
| 16747394 | parathymosin | PTMS | 0,30 | 0,05 | 0,25 | 0,23 |
| 16850797 |  | RAB12 | 0,30 | 0,00 | 0,25 | 0,05 |
| 17056476 | dpy-19-like 1 pseudogene 1 (C. elegans) \| AVL9 homolog (S. cerevisiase) | DPY19L1P1\| AVL9 | 0,30 | 0,01 | 0,11 | 0,68 |
| 16702967 | ADP-ribosylation factor-like 5B | ARL5B | 0,30 | 0,03 | 0,20 | 0,06 |
| 16853996 | AFG3 ATPase family gene 3-like 2 (S. cerevisiae) | AFG3L2 | 0,29 | 0,02 | 0,26 | 0,08 |
| 16674292 | Ral GEF with PH domain and SH3 binding motif 2 | RALGPS2 | 0,29 | 0,02 | 0,24 | 0,06 |
| 16847429 | nascent polypeptide-associated complex alpha subunit 2 | NACA2 | 0,29 | 0,03 | 0,11 | 0,68 |
| 16877297 | tribbles homolog 2 (Drosophila) | TRIB2 | 0,29 | 0,00 | 0,44 | 0,07 |
| 16837661 | otopetrin 2 | OTOP2 | 0,28 | 0,04 | 0,07 | 0,61 |
| 17114856 | uncharacterized LOC100131434 \| iduronate 2-sulfatase | LOC100131434\| IDS | 0,28 | 0,01 | 0,16 | 0,26 |
| 16852433 | polymerase (DNA directed) iota | POLI | 0,28 | 0,05 | 0,30 | 0,09 |
| 17029328 |  | LSM2 | 0,28 | 0,03 | 0,17 | 0,16 |
| 16744078 | nuclear protein, ataxia-telangiectasia locus | NPAT | 0,28 | 0,05 | 0,50 | 0,07 |
| 16849913 | ADP-ribosylation factor-like 16 | ARL16 | 0,28 | 0,01 | 0,26 | 0,17 |
| 17056515 | 5'-nucleotidase, cytosolic III | NT5C3 | 0,28 | 0,04 | 0,19 | 0,20 |
| 16856604 | receptor accessory protein 6 | REEP6 | 0,28 | 0,03 | 0,03 | 0,37 |
| 16963674 | zinc finger protein 141 | ZNF141 | 0,28 | 0,04 | 0,28 | 0,13 |
| 16988021 | adenomatous polyposis coli | APC | 0,28 | 0,00 | 0,17 | 0,15 |
| 16983907 | BRX1, biogenesis of ribosomes, homolog (S. cerevisiae) | BRIX1 | 0,28 | 0,04 | 0,11 | 0,08 |
| 16975578 | glucosamine-6-phosphate deaminase 2 | GNPDA2 | 0,28 | 0,02 | 0,27 | 0,12 |
| 17036832 |  | LSM2 | 0,28 | 0,02 | 0,15 | 0,20 |
| 16897184 | S1 RNA binding domain 1 | SRBD1 | 0,28 | 0,05 | 0,38 | 0,06 |
| 16916644 | U-box domain containing 5 \| FAST kinase domains 5 | UBOX5\| FASTKD5 | 0,28 | 0,03 | 0,17 | 0,33 |
| 17026855 |  | LSM2 | 0,28 | 0,03 | 0,16 | 0,16 |
| 16841525 | heparan sulfate (glucosamine) 3-O-sulfotransferase 3A1 | HS3ST3A1 | 0,28 | 0,04 | 0,32 | 0,15 |
| 17033974 | HLA complex group 18 (non-protein coding) | HCG18 | 0,28 | 0,02 | 0,12 | 0,40 |
| 17061759 | neuronal cell adhesion molecule | NRCAM | 0,28 | 0,01 | 0,23 | 0,18 |
| 17115428 | interleukin-1 receptor-associated kinase 1 | IRAK1 | 0,28 | 0,00 | 0,27 | 0,20 |
| 16928098 | macrophage migration inhibitory factor (glycosylation-inhibiting factor) \| uncharacterized LOC284889 | MIF\| LOC284889 | 0,27 | 0,02 | 0,19 | 0,18 |
| 16674805 | laminin, gamma 1 (formerly LAMB2) | LAMC1 | 0,27 | 0,02 | 0,30 | 0,06 |
| 16814693 | N-acetylglucosamine-1-phosphate transferase, gamma subunit | GNPTG | 0,27 | 0,00 | 0,18 | 0,08 |
| 16930066 | KDEL (Lys-Asp-Glu-Leu) endoplasmic reticulum protein retention receptor 3 | KDELR3 | 0,27 | 0,05 | 0,17 | 0,07 |
| 17098173 | zinc finger and BTB domain containing 6 | ZBTB6 | 0,27 | 0,02 | 0,39 | 0,09 |
| 16898394 |  | RAB1A | 0,27 | 0,05 | 0,17 | 0,06 |
| 16769585 | cytoskeleton-associated protein 4 | CKAP4 | 0,27 | 0,04 | 0,09 | 0,63 |
| 17077888 | COP9 constitutive photomorphogenic homolog subunit 5 (Arabidopsis) | COPS5 | 0,27 | 0,02 | 0,21 | 0,26 |
| 16998421 | RIO kinase 2 (yeast) | RIOK2 | 0,27 | 0,04 | 0,21 | 0,20 |
| 17032114 |  | LSM2 | 0,27 | 0,03 | 0,16 | 0,13 |
| 17085975 | osteoclast stimulating factor 1 | OSTF1 | 0,27 | 0,02 | 0,26 | 0,07 |
| 16673822 | methyltransferase like 13 | METTL13 | 0,27 | 0,02 | 0,13 | 0,40 |
| 16706875 | bone morphogenetic protein receptor, type IA | BMPR1A | 0,27 | 0,01 | 0,25 | 0,05 |
| 16885516 | sphingomyelin phosphodiesterase 4, neutral membrane (neutral sphingomyelinase-3) \| mitotic spindle organizing protein 2B \| mitotic spindle organizing protein 2A | SMPD4\| MZT2B\| MZT2A | 0,26 | 0,01 | 0,05 | 0,60 |
| 16739483 | heterogeneous nuclear ribonucleoprotein U-like 2 \| Berardinelli-Seip congenital lipodystrophy 2 (seipin) \| HNRNPUL2-BSCL2 readthrough | HNRNPUL2\| BSCL2\| HNRNPUL2-BSCL2 | 0,26 | 0,00 | 0,22 | 0,17 |
| 16907044 | SATB homeobox 2 | SATB2 | 0,26 | 0,05 | 0,25 | 0,07 |
| 16763375 | pseudouridylate synthase 7 homolog (S. cerevisiae)-like | PUS7L | 0,26 | 0,01 | 0,20 | 0,27 |
| 16684222 | serine/arginine-rich splicing factor 4 | SRSF4 | 0,26 | 0,03 | 0,31 | 0,06 |
| 16690473 | chloride channel CLIC-like 1 | CLCC1 | 0,26 | 0,03 | 0,27 | 0,09 |
| 17108658 | GTP binding protein 6 (putative) | GTPBP6 | 0,26 | 0,03 | 0,26 | 0,24 |
| 16662993 | rearranged L-myc fusion | RLF | 0,26 | 0,03 | 0,33 | 0,15 |
| 16661687 | erythrocyte membrane protein band 4.1 (elliptocytosis 1, RH-linked) | EPB41 | 0,26 | 0,00 | 0,20 | 0,46 |
| 16884403 | transmembrane protein 87B | TMEM87B | 0,26 | 0,04 | 0,23 | 0,07 |
| 17039565 |  | LSM2 | 0,26 | 0,02 | 0,16 | 0,17 |
| 16857402 | calcyphosine | CAPS | 0,26 | 0,03 | 0,15 | 0,22 |
| 17017476 |  | LSM2 | 0,26 | 0,02 | 0,16 | 0,16 |
| 16927772 | immunoglobulin lambda variable 2-33 (non-functional) | IGLV2-33 | 0,26 | 0,02 | 0,04 | 0,83 |
| 17025417 | 1-acylglycerol-3-phosphate O-acyltransferase 4 (lysophosphatidic acid acyltransferase, delta) | AGPAT4 | 0,26 | 0,00 | 0,01 | 0,95 |
| 17055480 | ankyrin repeat and MYND domain containing 2 | ANKMY2 | 0,26 | 0,01 | 0,15 | 0,09 |
| 16824481 | smg-1 homolog, phosphatidylinositol 3-kinase-related kinase (C. elegans) \| uncharacterized LOC100506830 \| SMG1 homolog, phosphatidylinositol 3-kinase-related kinase (C. elegans) pseudogene | SMG1\| LOC100506830\| LOC100506060 | 0,25 | 0,03 | 0,18 | 0,15 |
| 16747865 | Nanog homeobox pseudogene 1 | NANOGP1 | 0,25 | 0,00 | 0,03 | 0,69 |
| 16931569 | cysteine-rich with EGF-like domains 2 | CRELD2 | 0,25 | 0,00 | 0,05 | 0,65 |
| 16684110 | eyes absent homolog 3 (Drosophila) | EYA3 | 0,25 | 0,00 | 0,29 | 0,09 |
| 16976746 | ankyrin repeat domain 17 | ANKRD17 | 0,25 | 0,01 | 0,22 | 0,08 |
| 16749303 | LYR motif containing 5 | LYRM5 | 0,25 | 0,02 | 0,25 | 0,25 |
| 16812053 | TBC1 domain family, member 2B | TBC1D2B | 0,25 | 0,03 | 0,33 | 0,09 |
| 17096965 | erythrocyte membrane protein band 4.1 like 4B | EPB41L4B | 0,25 | 0,04 | 0,22 | 0,05 |
| 16912685 | BPI fold containing family B, member 6 | BPIFB6 | 0,25 | 0,03 | 0,20 | 0,09 |
| 16953993 | coiled-coil domain containing 71 | CCDC71 | 0,25 | 0,04 | 0,22 | 0,13 |
| 16847267 | tubulin, delta 1 | TUBD1 | 0,25 | 0,01 | 0,22 | 0,07 |
| 16998981 | protein geranylgeranyltransferase type I, beta subunit | PGGT1B | 0,25 | 0,01 | 0,17 | 0,11 |
| 16969051 | bone morphogenetic protein receptor, type IB | BMPR1B | 0,25 | 0,02 | 0,14 | 0,37 |
| 16919107 | bladder cancer associated protein | BLCAP | 0,25 | 0,02 | 0,15 | 0,28 |
| 16896481 | CCAAT/enhancer binding protein (C/EBP), zeta | CEBPZ | 0,24 | 0,00 | 0,16 | 0,19 |
| 16740814 | zinc finger, DHHC-type containing 24 | ZDHHC24 | 0,24 | 0,00 | 0,03 | 0,78 |
| 17014501 | mitogen-activated protein kinase kinase kinase 4 | MAP3K4 | 0,24 | 0,02 | 0,17 | 0,17 |
| 16817119 | trinucleotide repeat containing 6A | TNRC6A | 0,24 | 0,03 | 0,35 | 0,05 |
| 16668572 | chymosin pseudogene | CYMP | 0,24 | 0,02 | 0,23 | 0,31 |
| 16895268 | peptidyl-tRNA hydrolase domain containing 1 | PTRHD1 | 0,24 | 0,01 | 0,14 | 0,50 |
| 17084359 | T cell receptor beta variable 20/OR9-2 (non-functional) | TRBV20OR9-2 | 0,24 | 0,00 | 0,23 | 0,11 |
| 17005782 | transfer RNA isoleucine 2 (anticodon UAU) | TRNAI2 | 0,24 | 0,04 | 0,09 | 0,65 |
| 16825897 | B-cell CLL/lymphoma 7C | BCL7C | 0,24 | 0,00 | 0,18 | 0,41 |
| 16707695 | helicase, lymphoid-specific | HELLS | 0,24 | 0,01 | 0,13 | 0,34 |
| 16864721 | zinc finger protein 175 | ZNF175 | 0,24 | 0,04 | 0,28 | 0,34 |
| 16827566 | DEAD (Asp-Glu-Ala-Asp) box polypeptide 28 | DDX28 | 0,24 | 0,04 | 0,14 | 0,16 |
| 16980882 | microtubule-associated protein 9 | MAP9 | 0,24 | 0,05 | 0,19 | 0,10 |
| 16784947 | menage a trois homolog 1, cyclin H assembly factor (Xenopus laevis) | MNAT1 | 0,24 | 0,04 | 0,21 | 0,08 |
| 17000713 | prefoldin subunit 1 | PFDN1 | 0,23 | 0,03 | 0,19 | 0,18 |
| 17064135 | protein disulfide isomerase family A, member 4 | PDIA4 | 0,23 | 0,02 | 0,18 | 0,15 |
| 16936137 | ceramide kinase | CERK | 0,23 | 0,01 | 0,24 | 0,12 |
| 16747570 | enolase 2 (gamma, neuronal) | ENO2 | 0,23 | 0,02 | 0,09 | 0,15 |
| 17020103 | glutathione S-transferase alpha 4 | GSTA4 | 0,23 | 0,03 | -0,15 | 0,48 |
| 16989636 | kinesin family member 20A | KIF20A | 0,23 | 0,01 | 0,23 | 0,07 |
| 16835483 | ATP synthase, H+ transporting, mitochondrial Fo complex, subunit C1 (subunit 9) | ATP5G1 | 0,23 | 0,05 | 0,11 | 0,20 |
| 16999379 | aldehyde dehydrogenase 7 family, member A1 \| aldehyde dehydrogenase 7 family, member A1 pseudogene 1 | ALDH7A1\| ALDH7A1P1 | 0,23 | 0,02 | 0,08 | 0,10 |
| 17118444 | 60S ribosomal protein L23a-like \| 28S ribosomal protein S18c, mitochondrial-like \| mitochondrial ribosomal protein S18C | LOC100287195\| LOC100509751\| MRPS18C | 0,23 | 0,05 | 0,11 | 0,35 |
| 16853120 | teashirt zinc finger homeobox 1 | TSHZ1 | 0,23 | 0,05 | 0,31 | 0,29 |
| 17111251 | hydroxysteroid (17-beta) dehydrogenase 10 | HSD17B10 | 0,23 | 0,01 | 0,10 | 0,47 |
| 16855163 | ribosomal protein L17 \| RPL17-C18orf32 readthrough \| chromosome 18 open reading frame 32 | RPL17\| RPL17-C18ORF32\| C18orf32 | 0,23 | 0,01 | 0,18 | 0,14 |
| 16808386 | microfibrillar-associated protein 1 | MFAP1 | 0,23 | 0,01 | 0,26 | 0,12 |
| 16870640 | pre-B-cell leukemia homeobox 4 | PBX4 | 0,23 | 0,00 | 0,29 | 0,14 |
| 16791401 | short chain dehydrogenase/reductase family 39U, member 1 | SDR39U1 | 0,23 | 0,03 | 0,17 | 0,16 |
| 16743874 | caspase 5, apoptosis-related cysteine peptidase | CASP5 | 0,22 | 0,01 | 0,05 | 0,67 |
| 16777794 | ubiquitin-like 3 | UBL3 | 0,22 | 0,04 | 0,17 | 0,09 |
| 16671264 | solute carrier family 27 (fatty acid transporter), member 3 | SLC27A3 | 0,22 | 0,03 | 0,07 | 0,26 |
| 16664421 | cytochrome P450, family 4, subfamily A, polypeptide 22 | CYP4A22 | 0,22 | 0,04 | 0,05 | 0,73 |
| 16852982 | docking protein 6 | DOK6 | 0,22 | 0,01 | 0,33 | 0,22 |
| 17012100 | phospholamban | PLN | 0,22 | 0,04 | 0,14 | 0,07 |
| 16943047 | EPH receptor A6 | EPHA6 | 0,22 | 0,02 | 0,17 | 0,23 |
| 16967925 |  | USO1 | 0,22 | 0,04 | 0,14 | 0,10 |
| 16801143 | tropomodulin 3 (ubiquitous) | TMOD3 | 0,22 | 0,02 | 0,19 | 0,17 |
| 16768341 | ATPase, Ca++ transporting, plasma membrane 1 | ATP2B1 | 0,22 | 0,02 | 0,16 | 0,07 |
| 17064002 | olfactory receptor, family 2, subfamily A, member 20 pseudogene \| olfactory receptor, family 2, subfamily A, member 9 pseudogene | OR2A20P\| OR2A9P | 0,22 | 0,04 | -0,18 | 0,10 |
| 17080834 | FER1L6 antisense RNA 1 (non-protein coding) | FER1L6-AS1 | 0,22 | 0,03 | 0,16 | 0,67 |
| 17117611 | nuclear pore associated protein 1 pseudogene \| putative UPF0607 protein FLJ37424-like | LOC392364\| LOC100505502 | 0,22 | 0,05 | 0,01 | 0,87 |
| 16898665 | aspartic peptidase, retroviral-like 1 \| PCBP1 antisense RNA 1 (non-protein coding) | ASPRV1\| PCBP1-AS1 | 0,22 | 0,02 | 0,17 | 0,19 |
| 16721809 | zinc finger protein 143 | ZNF143 | 0,22 | 0,05 | 0,14 | 0,07 |
| 16761874 | RERG intronic transcript 1 (non-protein coding) | RERG-IT1 | 0,22 | 0,01 | 0,33 | 0,13 |
| 16951646 | N-glycanase 1 | NGLY1 | 0,22 | 0,04 | 0,06 | 0,45 |
| 16744524 | ubiquitin specific peptidase 28 | USP28 | 0,21 | 0,04 | 0,14 | 0,26 |
| 16746290 | opioid binding protein/cell adhesion molecule-like \| uncharacterized LOC100653275 | OPCML\| LOC100653275 | 0,21 | 0,02 | 0,15 | 0,48 |
| 17092710 | small Cajal body-specific RNA 8 | SCARNA8 | 0,21 | 0,01 | 0,10 | 0,05 |
| 16771560 | MORN repeat containing 3 | MORN3 | 0,21 | 0,01 | 0,14 | 0,32 |
| 16794441 | zinc finger, FYVE domain containing 1 | ZFYVE1 | 0,21 | 0,01 | 0,16 | 0,07 |
| 16904425 | growth factor receptor-bound protein 14 | GRB14 | 0,21 | 0,01 | -0,03 | 0,80 |
| 17009262 | CDC5 cell division cycle 5-like (S. pombe) | CDC5L | 0,21 | 0,04 | 0,26 | 0,07 |
| 16879883 | mutS homolog 2, colon cancer, nonpolyposis type 1 (E. coli) | MSH2 | 0,21 | 0,02 | 0,30 | 0,11 |
| 17060061 | asparagine synthetase (glutamine-hydrolyzing) | ASNS | 0,21 | 0,02 | 0,09 | 0,40 |
| 16944738 | protein disulfide isomerase family A, member 5 | PDIA5 | 0,21 | 0,01 | 0,07 | 0,57 |
| 17035090 | tripartite motif containing 40 | TRIM40 | 0,21 | 0,00 | 0,01 | 0,95 |
| 16872575 | UDP-GlcNAc:betaGal beta-1,3-N-acetylglucosaminyltransferase 8 | B3GNT8 | 0,21 | 0,03 | 0,04 | 0,73 |
| 16864901 | zinc finger protein 137, pseudogene | ZNF137P | 0,21 | 0,04 | 0,12 | 0,41 |
| 16673251 | microsomal glutathione S-transferase 3 \| uncharacterized LOC100505828 | MGST3\| LOC100505828 | 0,21 | 0,04 | 0,13 | 0,09 |
| 17086314 | idnK, gluconokinase homolog (E. coli) | IDNK | 0,21 | 0,05 | 0,08 | 0,17 |
| 16666091 | ZRANB2 antisense RNA 2 (non-protein coding) | ZRANB2-AS2 | 0,21 | 0,01 | -0,01 | 0,73 |
| 16751067 |  | COX14 | 0,21 | 0,03 | 0,15 | 0,20 |
| 16924668 | listerin E3 ubiquitin protein ligase 1 | LTN1 | 0,20 | 0,02 | 0,17 | 0,21 |
| 16795013 | vasohibin 1 \| uncharacterized LOC100506624 | VASH1\| LOC100506624 | 0,20 | 0,03 | 0,13 | 0,15 |
| 16725632 | protein phosphatase 1, regulatory subunit 32 | PPP1R32 | 0,20 | 0,02 | 0,08 | 0,31 |
| 16925911 | receptor-interacting serine-threonine kinase 4 | RIPK4 | 0,20 | 0,01 | 0,16 | 0,31 |
| 16830488 | polymerase (RNA) II (DNA directed) polypeptide A, 220kDa | POLR2A | 0,20 | 0,01 | 0,13 | 0,14 |
| 17078633 | zinc finger, AN1-type domain 1 | ZFAND1 | 0,20 | 0,03 | 0,24 | 0,18 |
| 16787667 | ITPK1 antisense RNA 1 (non-protein coding) | ITPK1-AS1 | 0,20 | 0,03 | -0,02 | 0,78 |
| 17023051 | golgi-associated PDZ and coiled-coil motif containing | GOPC | 0,20 | 0,00 | 0,29 | 0,17 |
| 16839184 | ribosomal protein L23a pseudogene | FLJ43681 | 0,20 | 0,02 | -0,04 | 0,74 |
| 16727074 | ovo-like 1(Drosophila) | OVOL1 | 0,20 | 0,03 | 0,09 | 0,45 |
| 16930678 | X-ray repair complementing defective repair in Chinese hamster cells 6 | XRCC6 | 0,20 | 0,03 | 0,10 | 0,13 |
| 16896279 | dpy-30 homolog (C. elegans) | DPY30 | 0,20 | 0,02 | 0,18 | 0,22 |
| 16800484 | tripartite motif containing 69 | TRIM69 | 0,20 | 0,04 | -0,07 | 0,82 |
| 17096631 | mitochondrial ribosomal protein L50 | MRPL50 | 0,20 | 0,02 | 0,09 | 0,26 |
| 16985491 | MAST4 intronic transcript 1 (non-protein coding) | MAST4-IT1 | 0,20 | 0,03 | 0,27 | 0,10 |
| 16823087 | enoyl-CoA delta isomerase 1 | ECI1 | 0,20 | 0,04 | 0,24 | 0,05 |
| 16989325 | calcium modulating ligand | CAMLG | 0,20 | 0,05 | 0,15 | 0,41 |
| 16844644 | keratin 33A | KRT33A | 0,20 | 0,02 | 0,03 | 0,84 |
| 17053925 | RNA binding motif protein 33 | RBM33 | 0,20 | 0,04 | 0,25 | 0,11 |
| 16813900 | small nuclear ribonucleoprotein polypeptide A' | SNRPA1 | 0,20 | 0,01 | 0,37 | 0,07 |
| 16814847 | fumarylacetoacetate hydrolase domain containing 1 | FAHD1 | 0,20 | 0,02 | 0,15 | 0,12 |
| 17080450 | exostosin 1 | EXT1 | 0,20 | 0,01 | 0,11 | 0,46 |
| 16990288 | protocadherin beta 16 | PCDHB16 | 0,20 | 0,04 | 0,21 | 0,23 |
| 17049003 | transmembrane protein 225-like \| zinc finger protein 655 | LOC100289187\| ZNF655 | 0,20 | 0,03 | 0,17 | 0,20 |
| 16757474 | phospholipase B domain containing 2 | PLBD2 | 0,19 | 0,05 | 0,21 | 0,05 |
| 16894979 | HCLS1 binding protein 3 | HS1BP3 | 0,19 | 0,01 | 0,04 | 0,77 |
| 16669288 | WD repeat domain 3 | WDR3 | 0,19 | 0,04 | 0,10 | 0,53 |
| 17097914 | PHD finger protein 19 | PHF19 | 0,19 | 0,03 | 0,23 | 0,08 |
| 16896650 | serine/arginine-rich splicing factor 7 | SRSF7 | 0,19 | 0,05 | 0,28 | 0,21 |
| 17093829 | glucosidase, beta (bile acid) 2 | GBA2 | 0,19 | 0,04 | 0,14 | 0,34 |
| 16988651 | GRAM domain containing 3 | GRAMD3 | 0,19 | 0,04 | 0,22 | 0,16 |
| 17050778 | inhibitor of growth family, member 3 | ING3 | 0,19 | 0,01 | 0,11 | 0,16 |
| 16792998 | thioredoxin domain containing 16 | TXNDC16 | 0,19 | 0,04 | 0,25 | 0,16 |
| 16831434 | ubiquitin B | UBB | 0,19 | 0,01 | 0,02 | 0,19 |
| 16776048 |  | FKSG29 | 0,19 | 0,05 | 0,20 | 0,13 |
| 16952383 | golgi reassembly stacking protein 1, 65kDa | GORASP1 | 0,19 | 0,02 | 0,14 | 0,32 |
| 16808974 | COP9 constitutive photomorphogenic homolog subunit 2 (Arabidopsis) | COPS2 | 0,19 | 0,04 | 0,07 | 0,23 |
| 16706849 | LIM domain binding 3 | LDB3 | 0,19 | 0,01 | -0,03 | 0,14 |
| 16900737 | ankyrin repeat domain 36B | ANKRD36B | 0,19 | 0,04 | 0,10 | 0,34 |
| 16743955 | kelch repeat and BTB (POZ) domain containing 3 | KBTBD3 | 0,19 | 0,03 | 0,14 | 0,18 |
| 16967614 | deoxycytidine kinase \| MOB kinase activator 1B | DCK\| MOB1B | 0,19 | 0,01 | 0,04 | 0,41 |
| 16700074 | CDC42 binding protein kinase alpha (DMPK-like) | CDC42BPA | 0,19 | 0,04 | 0,20 | 0,12 |
| 17049443 | guanine nucleotide binding protein (G protein), beta polypeptide 2 | GNB2 | 0,19 | 0,00 | 0,13 | 0,25 |
| 17021901 | failed axon connections homolog (Drosophila) | FAXC | 0,19 | 0,04 | 0,09 | 0,44 |
| 16706894 | RNA, U1 small nuclear 19, pseudogene | RNU1-19P | 0,19 | 0,02 | -0,09 | 0,62 |
| 17064888 | motor neuron and pancreas homeobox 1 | MNX1 | 0,18 | 0,00 | 0,23 | 0,11 |
| 16693864 | phosphomevalonate kinase | PMVK | 0,18 | 0,03 | 0,20 | 0,10 |
| 16732520 | tubulin folding cofactor E-like | TBCEL | 0,18 | 0,04 | 0,15 | 0,22 |
| 16885199 | translin | TSN | 0,18 | 0,03 | 0,19 | 0,08 |
| 17046249 | mitochondrial ribosomal protein S17 | MRPS17 | 0,18 | 0,04 | 0,16 | 0,15 |
| 16838561 | solute carrier family 26, member 11 | SLC26A11 | 0,18 | 0,01 | 0,27 | 0,08 |
| 16836735 | T-box 2 | TBX2 | 0,18 | 0,03 | 0,13 | 0,06 |
| 17095587 | sema domain, immunoglobulin domain (Ig), transmembrane domain (TM) and short cytoplasmic domain, (semaphorin) 4D | SEMA4D | 0,18 | 0,00 | 0,11 | 0,55 |
| 16819600 | katanin p80 (WD repeat containing) subunit B 1 | KATNB1 | 0,18 | 0,02 | 0,09 | 0,26 |
| 16893392 | protein phosphatase 1, regulatory subunit 7 | PPP1R7 | 0,18 | 0,00 | 0,01 | 0,84 |
| 17066815 | tumor necrosis factor receptor superfamily, member 10c, decoy without an intracellular domain \| uncharacterized LOC254896 | TNFRSF10C\| LOC254896 | 0,18 | 0,05 | 0,09 | 0,65 |
| 16821882 | trafficking protein particle complex 2-like | TRAPPC2L | 0,18 | 0,02 | 0,00 | 0,97 |
| 16997662 | dihydrofolate reductase | DHFR | 0,18 | 0,01 | 0,13 | 0,09 |
| 16666448 | GIPC PDZ domain containing family, member 2 | GIPC2 | 0,18 | 0,05 | -0,11 | 0,57 |
| 16962407 | diacylglycerol kinase, gamma 90kDa | DGKG | 0,18 | 0,02 | -0,01 | 0,93 |
| 16889602 |  | NOP58 | 0,18 | 0,01 | 0,16 | 0,11 |
| 17113596 | NFKB repressing factor | NKRF | 0,18 | 0,02 | 0,13 | 0,56 |
| 16821621 | interferon regulatory factor 8 | IRF8 | 0,18 | 0,00 | 0,01 | 0,93 |
| 16822809 | mitochondrial ribosomal protein S34 | MRPS34 | 0,18 | 0,03 | 0,25 | 0,24 |
| 16818207 | fused in sarcoma | FUS | 0,18 | 0,01 | 0,21 | 0,12 |
| 16659765 | spen homolog, transcriptional regulator (Drosophila) | SPEN | 0,18 | 0,00 | 0,27 | 0,08 |
| 16815128 | TBC1 domain family, member 24 | TBC1D24 | 0,18 | 0,03 | 0,11 | 0,32 |
| 16984244 | oncostatin M receptor | OSMR | 0,18 | 0,04 | 0,38 | 0,07 |
| 16998130 | POU domain class 5, transcription factor 2 | POU5F2 | 0,18 | 0,02 | 0,04 | 0,59 |
| 17103340 | TBC1 domain family, member 25 | TBC1D25 | 0,18 | 0,03 | 0,06 | 0,59 |
| 16967100 | polymerase (RNA) II (DNA directed) polypeptide B, 140kDa | POLR2B | 0,18 | 0,03 | 0,11 | 0,13 |
| 16961308 | golgi integral membrane protein 4 | GOLIM4 | 0,18 | 0,00 | 0,03 | 0,80 |
| 16685076 | splicing factor proline/glutamine-rich | SFPQ | 0,18 | 0,02 | 0,13 | 0,36 |
| 16870394 | leucine rich repeat containing 25 | LRRC25 | 0,18 | 0,01 | -0,04 | 0,74 |
| 16668785 | wingless-type MMTV integration site family, member 2B | WNT2B | 0,18 | 0,02 | 0,08 | 0,16 |
| 16709489 | von Willebrand factor A domain containing 2 | VWA2 | 0,18 | 0,01 | 0,31 | 0,21 |
| 16887635 | histone acetyltransferase 1 | HAT1 | 0,17 | 0,03 | 0,06 | 0,32 |
| 16874583 | leucine rich repeat containing 4B | LRRC4B | 0,17 | 0,04 | 0,17 | 0,15 |
| 16678838 | nucleoside-triphosphatase, cancer-related | NTPCR | 0,17 | 0,04 | 0,24 | 0,10 |
| 16695572 | prefoldin subunit 2 | PFDN2 | 0,17 | 0,04 | 0,07 | 0,47 |
| 16947974 | fibronectin type III domain containing 3B | FNDC3B | 0,17 | 0,02 | 0,12 | 0,23 |
| 16717562 | SEC31 homolog B (S. cerevisiae) | SEC31B | 0,17 | 0,03 | 0,17 | 0,14 |
| 16667155 | ribosomal protein L5 \| small nucleolar RNA, C/D box 21 | RPL5\| SNORD21 | 0,17 | 0,04 | 0,10 | 0,14 |
| 16799590 | RNA pseudouridylate synthase domain containing 2 | RPUSD2 | 0,17 | 0,04 | 0,19 | 0,15 |
| 16870581 | nuclear receptor 2C2-associated protein | NR2C2AP | 0,17 | 0,01 | 0,19 | 0,20 |
| 16823756 | ubiquitin specific peptidase 7 (herpes virus-associated) | USP7 | 0,17 | 0,05 | 0,09 | 0,09 |
| 16711987 | cerebral dopamine neurotrophic factor | CDNF | 0,17 | 0,03 | 0,07 | 0,54 |
| 16804687 | mesoderm posterior 2 homolog (mouse) | MESP2 | 0,17 | 0,01 | 0,18 | 0,16 |
| 16777549 | myotubularin related protein 6 | MTMR6 | 0,17 | 0,01 | 0,24 | 0,15 |
| 16722890 | anoctamin 5 | ANO5 | 0,17 | 0,02 | 0,19 | 0,38 |
| 16698923 | interferon regulatory factor 6 | IRF6 | 0,17 | 0,03 | 0,17 | 0,10 |
| 16741501 | 7-dehydrocholesterol reductase | DHCR7 | 0,17 | 0,01 | 0,20 | 0,15 |
| 17025666 | ribonuclease T2 | RNASET2 | 0,17 | 0,04 | 0,21 | 0,11 |
| 16735102 | ADP-ribosylation factor interacting protein 2 | ARFIP2 | 0,17 | 0,00 | 0,14 | 0,11 |
| 16769983 | G protein-coupled receptor kinase interacting ArfGAP 2 | GIT2 | 0,17 | 0,04 | 0,21 | 0,16 |
| 16715133 | pyrophosphatase (inorganic) 1 | PPA1 | 0,17 | 0,01 | 0,09 | 0,15 |
| 17021888 | F-box and leucine-rich repeat protein 4 | FBXL4 | 0,17 | 0,00 | 0,22 | 0,16 |
| 16943410 | PEST proteolytic signal containing nuclear protein | PCNP | 0,17 | 0,02 | 0,07 | 0,08 |
| 16847810 | polymerase (DNA directed), gamma 2, accessory subunit | POLG2 | 0,17 | 0,04 | 0,07 | 0,70 |
| 17082081 | RHPN1 antisense RNA 1 (non-protein coding) | RHPN1-AS1 | 0,17 | 0,01 | 0,23 | 0,12 |
| 16706600 | placenta-specific 9 | PLAC9 | 0,17 | 0,04 | 0,04 | 0,37 |
| 17011895 | 5'-nucleotidase domain containing 1 | NT5DC1 | 0,17 | 0,00 | 0,19 | 0,29 |
| 16840919 | CTS telomere maintenance complex component 1 | CTC1 | 0,17 | 0,01 | 0,07 | 0,45 |
| 17077826 | v-myb myeloblastosis viral oncogene homolog (avian)-like 1 \| uncharacterized LOC645895 | MYBL1\| LOC645895 | 0,17 | 0,04 | 0,19 | 0,12 |
| 16778468 | mitochondrial translational release factor 1 | MTRF1 | 0,16 | 0,04 | 0,00 | 0,95 |
| 16686000 | claudin 19 | CLDN19 | 0,16 | 0,03 | 0,07 | 0,31 |
| 17020918 | cytochrome c oxidase subunit VIIa polypeptide 2 (liver) | COX7A2 | 0,16 | 0,02 | 0,02 | 0,65 |
| 16674025 | RAB GTPase activating protein 1-like | RABGAP1L | 0,16 | 0,04 | 0,13 | 0,26 |
| 17012350 | tRNA methyltransferase 11 homolog (S. cerevisiae) | TRMT11 | 0,16 | 0,04 | 0,29 | 0,06 |
| 16670383 | histone cluster 2, H2aa4 \| histone cluster 2, H2aa3 | HIST2H2AA4\| HIST2H2AA3 | 0,16 | 0,04 | 0,14 | 0,09 |
| 17044862 | aquaporin 1 (Colton blood group) | AQP1 | 0,16 | 0,01 | 0,01 | 0,96 |
| 16753228 | XRCC6 binding protein 1 | XRCC6BP1 | 0,16 | 0,03 | 0,13 | 0,13 |
| 16909049 | cullin 3 | CUL3 | 0,16 | 0,03 | 0,13 | 0,11 |
| 16860709 | glucose-6-phosphate isomerase | GPI | 0,16 | 0,03 | 0,05 | 0,37 |
| 17076415 | fibroblast growth factor receptor 1 | FGFR1 | 0,16 | 0,03 | 0,18 | 0,19 |
| 17045528 | HECW1 intronic transcript 1 (non-protein coding) | HECW1-IT1 | 0,16 | 0,05 | -0,01 | 0,93 |
| 16753182 | membrane-associated ring finger (C3HC4) 9 | MARCH9 | 0,16 | 0,03 | 0,10 | 0,21 |
| 17113079 | MORC family CW-type zinc finger 4 | MORC4 | 0,16 | 0,04 | 0,19 | 0,12 |
| 16753964 | chaperonin containing TCP1, subunit 2 (beta) | CCT2 | 0,16 | 0,01 | 0,08 | 0,07 |
| 16866081 | zinc finger protein interacting with K protein 1 homolog (mouse) | ZIK1 | 0,16 | 0,02 | 0,04 | 0,83 |
| 16980744 | ring finger protein 175 | RNF175 | 0,16 | 0,04 | 0,03 | 0,80 |
| 16971167 | SMAD family member 1 | SMAD1 | 0,16 | 0,04 | 0,12 | 0,14 |
| 16803269 | F-box protein 22 | FBXO22 | 0,16 | 0,00 | 0,11 | 0,15 |
| 16997784 | transmembrane protein 167A | TMEM167A | 0,16 | 0,01 | 0,12 | 0,21 |
| 16700778 | beta-1,3-N-acetylgalactosaminyltransferase 2 | B3GALNT2 | 0,16 | 0,03 | 0,06 | 0,75 |
| 16952218 | phospholipase C, delta 1 | PLCD1 | 0,16 | 0,00 | 0,14 | 0,39 |
| 16750002 | mucin 19, oligomeric | MUC19 | 0,16 | 0,02 | 0,06 | 0,38 |
| 17118166 | zinc finger protein 451 | ZNF451 | 0,16 | 0,00 | 0,16 | 0,35 |
| 16763626 | RNA polymerase II associated protein 3 | RPAP3 | 0,16 | 0,04 | 0,10 | 0,07 |
| 17067941 | G protein-coupled receptor 124 | GPR124 | 0,15 | 0,01 | 0,13 | 0,26 |
| 16990119 | IK cytokine, down-regulator of HLA II \| microRNA 3655 | IK\| MIR3655 | 0,15 | 0,04 | 0,07 | 0,11 |
| 17019425 | exportin 5 | XPO5 | 0,15 | 0,05 | 0,24 | 0,10 |
| 16761778 | histone cluster 2, H4b \| histone cluster 4, H4 \| histone cluster 2, H4a \| histone cluster 1, H4l \| histone cluster 1, H4e \| histone cluster 1, H4b \| histone cluster 1, H4h \| histone cluster 1, H4c \| histone cluster 1, H4j \| histone cluster 1, H4k \| histone cluster 1, H4f \| histone cluster 1, H4d \| histone cluster 1, H4a \| histone cluster 1, H4i | HIST2H4B\| HIST4H4\| HIST2H4A\| HIST1H4L\| HIST1H4E\| HIST1H4B\| HIST1H4H\| HIST1H4C\| HIST1H4J\| HIST1H4K\| HIST1H4F\| HIST1H4D\| HIST1H4A\| HIST1H4I | 0,15 | 0,03 | 0,22 | 0,12 |
| 17098027 | stomatin | STOM | 0,15 | 0,05 | 0,02 | 0,86 |
| 16851309 | growth regulation by estrogen in breast cancer-like | GREB1L | 0,15 | 0,01 | 0,13 | 0,12 |
| 16760082 | EF-hand calcium binding domain 4B | EFCAB4B | 0,15 | 0,03 | 0,11 | 0,35 |
| 16875549 | glycoprotein VI (platelet) | GP6 | 0,15 | 0,05 | -0,03 | 0,65 |
| 16685330 | glutamate receptor, ionotropic, kainate 3 | GRIK3 | 0,15 | 0,02 | 0,14 | 0,10 |
| 16857449 | crumbs homolog 3 (Drosophila) | CRB3 | 0,15 | 0,03 | 0,04 | 0,74 |
| 16753125 | osteosarcoma amplified 9, endoplasmic reticulum lectin | OS9 | 0,15 | 0,01 | 0,05 | 0,57 |
| 16707454 | membrane-associated ring finger (C3HC4) 5 | MARCH5 | 0,15 | 0,03 | 0,16 | 0,07 |
| 17042925 | extracellular leucine-rich repeat and fibronectin type III domain containing 1 | ELFN1 | 0,15 | 0,05 | 0,17 | 0,39 |
| 16767142 | transmembrane BAX inhibitor motif containing 4 | TMBIM4 | 0,15 | 0,01 | 0,08 | 0,06 |
| 16683397 | UDP-galactose-4-epimerase | GALE | 0,15 | 0,01 | 0,11 | 0,08 |
| 16772559 | E1A binding protein p400 | EP400 | 0,15 | 0,04 | -0,20 | 0,09 |
| 16738713 | oxysterol binding protein | OSBP | 0,15 | 0,00 | 0,22 | 0,18 |
| 17068510 | polymerase (DNA directed), beta | POLB | 0,15 | 0,03 | 0,10 | 0,06 |
| 16880682 | aftiphilin | AFTPH | 0,15 | 0,03 | 0,17 | 0,06 |
| 16933630 | THO complex 5 | THOC5 | 0,15 | 0,02 | 0,17 | 0,08 |
| 16866981 | DIRAS family, GTP-binding RAS-like 1 | DIRAS1 | 0,15 | 0,01 | -0,04 | 0,29 |
| 16763939 | KAT8 regulatory NSL complex subunit 2 | KANSL2 | 0,15 | 0,00 | 0,13 | 0,29 |
| 16876310 | serine/arginine-rich splicing factor 10 | SRSF10 | 0,15 | 0,00 | 0,05 | 0,47 |
| 16810612 | spastic paraplegia 21 (autosomal recessive, Mast syndrome) | SPG21 | 0,15 | 0,03 | 0,15 | 0,07 |
| 16773874 | PDS5, regulator of cohesion maintenance, homolog B (S. cerevisiae) | PDS5B | 0,15 | 0,03 | 0,17 | 0,17 |
| 17054040 | ubiquitin protein ligase E3C | UBE3C | 0,15 | 0,01 | 0,08 | 0,42 |
| 16697930 | pleckstrin homology-like domain, family A, member 3 | PHLDA3 | 0,15 | 0,00 | 0,06 | 0,51 |
| 16873487 | Myb-related transcription factor, partner of profilin | MYPOP | 0,15 | 0,03 | 0,14 | 0,25 |
| 16786133 | RNA binding motif protein 25 | RBM25 | 0,14 | 0,05 | 0,14 | 0,16 |
| 16852997 | suppressor of cytokine signaling 6 | SOCS6 | 0,14 | 0,00 | 0,31 | 0,07 |
| 16879679 | protein kinase C, epsilon | PRKCE | 0,14 | 0,00 | 0,13 | 0,31 |
| 16732248 | NLR family member X1 | NLRX1 | 0,14 | 0,00 | 0,16 | 0,24 |
| 16773946 | replication factor C (activator 1) 3, 38kDa | RFC3 | 0,14 | 0,01 | 0,12 | 0,14 |
| 16696796 | RASAL2 antisense RNA 1 (non-protein coding) | RASAL2-AS1 | 0,14 | 0,04 | 0,02 | 0,75 |
| 16972155 | methylsterol monooxygenase 1 | MSMO1 | 0,14 | 0,03 | 0,08 | 0,42 |
| 16770590 | serine dehydratase | SDS | 0,14 | 0,02 | 0,10 | 0,58 |
| 17043843 | tetraspanin 13 | TSPAN13 | 0,14 | 0,00 | -0,03 | 0,31 |
| 17116720 | solute carrier family 25 (mitochondrial carrier; adenine nucleotide translocator), member 6 | SLC25A6 | 0,14 | 0,03 | 0,11 | 0,09 |
| 16797487 | immunoglobulin heavy variable 1-18 | IGHV1-18 | 0,14 | 0,03 | -0,01 | 0,70 |
| 17033956 | tripartite motif containing 26 | TRIM26 | 0,14 | 0,02 | 0,15 | 0,14 |
| 17021557 | gamma-aminobutyric acid (GABA) A receptor, rho 1 | GABRR1 | 0,14 | 0,01 | 0,01 | 0,81 |
| 16842382 | A kinase (PRKA) anchor protein 10 | AKAP10 | 0,14 | 0,03 | 0,13 | 0,10 |
| 16693433 | S100 calcium binding protein A6 | S100A6 | 0,14 | 0,04 | -0,05 | 0,82 |
| 16967031 | phosphoribosylaminoimidazole carboxylase, phosphoribosylaminoimidazole succinocarboxamide synthetase | PAICS | 0,14 | 0,00 | 0,08 | 0,26 |
| 16869769 | notch 3 | NOTCH3 | 0,14 | 0,05 | 0,09 | 0,37 |
| 16784829 | JNK1/MAPK8-associated membrane protein | JKAMP | 0,14 | 0,03 | 0,07 | 0,19 |
| 16787869 | serpin peptidase inhibitor, clade A (alpha-1 antiproteinase, antitrypsin), member 4 | SERPINA4 | 0,14 | 0,00 | 0,01 | 0,77 |
| 16926570 | solute carrier family 19 (folate transporter), member 1 \| uncharacterized LOC100130597 | SLC19A1\| LOC100130597 | 0,14 | 0,01 | 0,17 | 0,20 |
| 16815276 | kringle containing transmembrane protein 2 | KREMEN2 | 0,14 | 0,03 | 0,08 | 0,27 |
| 17055472 | sclerostin domain containing 1 | SOSTDC1 | 0,14 | 0,02 | 0,07 | 0,77 |
| 16868356 | olfactory receptor, family 7, subfamily G, member 3 | OR7G3 | 0,14 | 0,01 | -0,08 | 0,53 |
| 16997503 | lipoma HMGIC fusion partner-like 2 | LHFPL2 | 0,14 | 0,03 | 0,16 | 0,16 |
| 16833119 | zinc finger protein 207 \| microRNA 632 | ZNF207\| MIR632 | 0,14 | 0,02 | 0,18 | 0,17 |
| 17089174 | LIM homeobox transcription factor 1, beta | LMX1B | 0,14 | 0,00 | 0,10 | 0,19 |
| 16893709 | SH3 domain containing, Ysc84-like 1 (S. cerevisiae) | SH3YL1 | 0,14 | 0,04 | 0,03 | 0,67 |
| 16796020 | ataxin 3 | ATXN3 | 0,14 | 0,04 | 0,09 | 0,39 |
| 17056143 | homeobox A6 | HOXA6 | 0,14 | 0,05 | 0,14 | 0,23 |
| 16715409 | DnaJ (Hsp40) homolog, subfamily C, member 9 \| uncharacterized LOC100653136 \| uncharacterized LOC100652993 \| mitochondrial ribosomal protein S16 | DNAJC9\| LOC100653136\| LOC100652993\| MRPS16 | 0,14 | 0,03 | 0,21 | 0,25 |
| 16789955 | ribonuclease P RNA component H1 | RPPH1 | 0,14 | 0,04 | -0,09 | 0,43 |
| 16952925 | ALS2 C-terminal like | ALS2CL | 0,14 | 0,03 | 0,10 | 0,34 |
| 16915868 | baculoviral IAP repeat containing 7 | BIRC7 | 0,14 | 0,01 | 0,12 | 0,60 |
| 16850154 | coiled-coil domain containing 57 | CCDC57 | 0,14 | 0,04 | 0,08 | 0,11 |
| 16836333 | serine carboxypeptidase 1 | SCPEP1 | 0,14 | 0,03 | 0,21 | 0,08 |
| 16796207 | DEAD (Asp-Glu-Ala-Asp) box polypeptide 24 | DDX24 | 0,14 | 0,02 | 0,13 | 0,16 |
| 16827502 | solute carrier family 12 (potassium/chloride transporters), member 4 | SLC12A4 | 0,14 | 0,03 | 0,10 | 0,08 |
| 16886864 | membrane-associated ring finger (C3HC4) 7, E3 ubiquitin protein ligase | MARCH7 | 0,13 | 0,04 | 0,17 | 0,16 |
| 16966733 | RAS-like, family 11, member B | RASL11B | 0,13 | 0,02 | 0,09 | 0,29 |
| 16951485 | shugoshin-like 1 (S. pombe) | SGOL1 | 0,13 | 0,03 | 0,03 | 0,76 |
| 16996896 | coiled-coil domain containing 125 | CCDC125 | 0,13 | 0,04 | 0,07 | 0,55 |
| 16670920 | sorting nexin family member 27 | SNX27 | 0,13 | 0,05 | 0,23 | 0,07 |
| 16861704 | proteasome (prosome, macropain) 26S subunit, non-ATPase, 8 | PSMD8 | 0,13 | 0,01 | 0,12 | 0,07 |
| 17001578 | platelet-derived growth factor receptor, beta polypeptide | PDGFRB | 0,13 | 0,03 | 0,03 | 0,71 |
| 16804867 | CREB regulated transcription coactivator 3 | CRTC3 | 0,13 | 0,02 | 0,11 | 0,05 |
| 17056248 | carboxypeptidase, vitellogenic-like | CPVL | 0,13 | 0,04 | -0,01 | 0,91 |
| 17113463 | leucine-rich repeats and calponin homology (CH) domain containing 2 | LRCH2 | 0,13 | 0,00 | -0,01 | 0,82 |
| 16776856 | lysosomal-associated membrane protein 1 | LAMP1 | 0,13 | 0,04 | 0,25 | 0,07 |
| 16946341 | acid phosphatase-like 2 | ACPL2 | 0,13 | 0,04 | 0,10 | 0,29 |
| 16951997 | upstream binding protein 1 (LBP-1a) | UBP1 | 0,13 | 0,01 | 0,10 | 0,20 |
| 16861010 | free fatty acid receptor 1 | FFAR1 | 0,13 | 0,03 | 0,01 | 0,83 |
| 16687273 | enoyl CoA hydratase domain containing 2 | ECHDC2 | 0,13 | 0,00 | 0,25 | 0,19 |
| 16697245 | influenza virus NS1A binding protein | IVNS1ABP | 0,13 | 0,02 | 0,09 | 0,49 |
| 17045198 | anillin, actin binding protein | ANLN | 0,13 | 0,02 | 0,11 | 0,05 |
| 16976942 | GTPase activating protein (SH3 domain) binding protein 2 | G3BP2 | 0,13 | 0,05 | 0,17 | 0,11 |
| 16757894 | glutamyl-tRNA(Gln) amidotransferase, subunit C homolog (bacterial) | GATC | 0,13 | 0,02 | 0,07 | 0,09 |
| 16692209 | neuroblastoma breakpoint family, member 11 \| neuroblastoma breakpoint family, member 24 \| neuroblastoma breakpoint family, member 15 \| neuroblastoma breakpoint family, member 16 \| neuroblastoma breakpoint family, member 8 \| neuroblastoma breakpoint family, member 9 \| neuroblastoma breakpoint family, member 14 \| neuroblastoma breakpoint family, member 10 \| neuroblastoma breakpoint family, member 12 \| neuroblastoma breakpoint family member 21-like \| neuroblastoma breakpoint family, member 7 \| neuroblastoma breakpoint family, member 1 | NBPF11\| NBPF24\| NBPF15\| NBPF16\| NBPF8\| NBPF9\| NBPF14\| NBPF10\| NBPF12\| LOC100506032\| NBPF7 | 0,13 | 0,01 | 0,11 | 0,15 |
| 16794705 | Niemann-Pick disease, type C2 \| microRNA 4709 | NPC2\| MIR4709 | 0,13 | 0,05 | 0,08 | 0,19 |
| 16869719 | olfactory receptor, family 7, subfamily A, member 2 pseudogene | OR7A2P | 0,13 | 0,03 | -0,04 | 0,66 |
| 16985629 | mitochondrial ribosomal protein S36 | MRPS36 | 0,13 | 0,01 | 0,08 | 0,46 |
| 16880511 | copper metabolism (Murr1) domain containing 1 | COMMD1 | 0,12 | 0,03 | 0,12 | 0,20 |
| 17001506 | phosphodiesterase 6A, cGMP-specific, rod, alpha | PDE6A | 0,12 | 0,03 | 0,05 | 0,70 |
| 16986138 | 190 kDa guanine nucleotide exchange factor | RGNEF | 0,12 | 0,00 | 0,08 | 0,31 |
| 16745563 | CXADR-like membrane protein | CLMP | 0,12 | 0,01 | 0,09 | 0,37 |
| 16786652 | tubulin tyrosine ligase-like family, member 5 | TTLL5 | 0,12 | 0,01 | 0,11 | 0,13 |
| 16769362 | 5'-nucleotidase domain containing 3 | NT5DC3 | 0,12 | 0,02 | 0,04 | 0,66 |
| 16849970 | Rho GDP dissociation inhibitor (GDI) alpha | ARHGDIA | 0,12 | 0,01 | 0,03 | 0,72 |
| 16768628 | NADH dehydrogenase (ubiquinone) 1 alpha subcomplex, 12 | NDUFA12 | 0,12 | 0,05 | 0,01 | 0,95 |
| 17018292 | mitochondrial nucleoid factor 1 | MNF1 | 0,12 | 0,03 | 0,10 | 0,21 |
| 16938429 | 3-oxoacyl-ACP synthase, mitochondrial | OXSM | 0,12 | 0,04 | 0,09 | 0,45 |
| 16756370 | regulatory factor X, 4 (influences HLA class II expression) | RFX4 | 0,12 | 0,05 | 0,06 | 0,15 |
| 17008105 | TBC1 domain family, member 22B | TBC1D22B | 0,12 | 0,03 | 0,23 | 0,15 |
| 16723593 | pyruvate dehydrogenase complex, component X | PDHX | 0,12 | 0,02 | 0,02 | 0,78 |
| 16812831 | SEC11 homolog A (S. cerevisiae) | SEC11A | 0,12 | 0,00 | 0,16 | 0,25 |
| 16752652 | SPRY domain containing 4 | SPRYD4 | 0,12 | 0,03 | 0,13 | 0,10 |
| 16828499 | adenosine deaminase, tRNA-specific 1 | ADAT1 | 0,12 | 0,03 | 0,31 | 0,08 |
| 17046254 | chaperonin containing TCP1, subunit 6A (zeta 1) | CCT6A | 0,12 | 0,04 | 0,06 | 0,59 |
| 16969658 | SEC24 family, member B (S. cerevisiae) | SEC24B | 0,12 | 0,02 | 0,17 | 0,20 |
| 16893349 | sushi, nidogen and EGF-like domains 1 | SNED1 | 0,12 | 0,04 | 0,06 | 0,16 |
| 16953303 | NME/NM23 nucleoside diphosphate kinase 6 | NME6 | 0,12 | 0,02 | 0,06 | 0,41 |
| 16754166 | THAP domain containing, apoptosis associated protein 2 | THAP2 | 0,12 | 0,01 | 0,23 | 0,09 |
| 16973331 | major facilitator superfamily domain containing 7 | MFSD7 | 0,12 | 0,05 | -0,02 | 0,82 |
| 16871359 | dermokine | DMKN | 0,12 | 0,03 | 0,09 | 0,20 |
| 16932612 | phosphatidylinositol 4-kinase, catalytic, alpha | PI4KA | 0,11 | 0,03 | 0,12 | 0,07 |
| 17117867 | endothelial PAS domain protein 1 \| uncharacterized LOC100652809 | EPAS1\| LOC100652809 | 0,11 | 0,01 | 0,14 | 0,22 |
| 17080486 | tumor necrosis factor receptor superfamily, member 11b | TNFRSF11B | 0,11 | 0,01 | 0,00 | 1,00 |
| 16728261 | cyclin D1 | CCND1 | 0,11 | 0,03 | 0,09 | 0,19 |
| 16665656 | ribonucleoprotein, PTB-binding 2 | RAVER2 | 0,11 | 0,04 | 0,30 | 0,12 |
| 16807525 |  | INO80 | 0,11 | 0,01 | 0,15 | 0,10 |
| 17016832 | tripartite motif containing 26 | TRIM26 | 0,11 | 0,01 | 0,15 | 0,13 |
| 16809326 | LysM, putative peptidoglycan-binding, domain containing 2 | LYSMD2 | 0,11 | 0,04 | 0,01 | 0,93 |
| 16830220 | arachidonate 12-lipoxygenase pseudogene 2 | ALOX12P2 | 0,11 | 0,02 | 0,20 | 0,22 |
| 16886066 | thrombospondin, type I, domain containing 7B | THSD7B | 0,11 | 0,03 | 0,12 | 0,29 |
| 16920370 | sal-like 4 (Drosophila) | SALL4 | 0,11 | 0,04 | -0,08 | 0,70 |
| 17089999 | protein phosphatase 2A activator, regulatory subunit 4 | PPP2R4 | 0,11 | 0,03 | 0,09 | 0,33 |
| 16787951 | glutaredoxin 5 | GLRX5 | 0,11 | 0,03 | 0,05 | 0,31 |
| 16685042 | zinc finger, MYM-type 6 | ZMYM6 | 0,11 | 0,04 | 0,14 | 0,18 |
| 17077594 | aspartate beta-hydroxylase | ASPH | 0,11 | 0,03 | 0,05 | 0,39 |
| 16970611 | La ribonucleoprotein domain family, member 1B | LARP1B | 0,11 | 0,02 | 0,16 | 0,23 |
| 16660038 | peptidyl arginine deiminase, type III | PADI3 | 0,11 | 0,04 | 0,34 | 0,08 |
| 16945933 | centrosomal protein 63kDa | CEP63 | 0,11 | 0,04 | 0,03 | 0,75 |
| 17117786 | family with sequence similarity 104, member B pseudogene | LOC100287188\| FAM104B | 0,11 | 0,02 | 0,07 | 0,23 |
| 16864331 | mediator complex subunit 25 | MED25 | 0,11 | 0,02 | 0,26 | 0,10 |
| 16712168 | cubilin (intrinsic factor-cobalamin receptor) | CUBN | 0,11 | 0,01 | 0,02 | 0,86 |
| 16745961 | kin of IRRE like 3 (Drosophila) | KIRREL3 | 0,11 | 0,01 | 0,18 | 0,16 |
| 16797784 | cytoplasmic FMR1 interacting protein 1 | CYFIP1 | 0,11 | 0,02 | 0,17 | 0,08 |
| 16845850 | hexamethylene bis-acetamide inducible 1 | HEXIM1 | 0,11 | 0,04 | 0,10 | 0,11 |
| 16894848 | WD repeat domain 35 | WDR35 | 0,10 | 0,02 | 0,09 | 0,09 |
| 16728153 | immunoglobulin mu binding protein 2 | IGHMBP2 | 0,10 | 0,03 | 0,26 | 0,07 |
| 17082366 | plectin | PLEC | 0,10 | 0,04 | 0,18 | 0,09 |
| 16816692 | methyltransferase like 9 | METTL9 | 0,10 | 0,02 | 0,27 | 0,11 |
| 17049557 | thyroid hormone receptor interactor 6 | TRIP6 | 0,10 | 0,01 | 0,02 | 0,70 |
| 16897311 | calmodulin 3 (phosphorylase kinase, delta) \| calmodulin 2 (phosphorylase kinase, delta) \| calmodulin 1 (phosphorylase kinase, delta) | CALM3\| CALM2\| CALM1 | 0,10 | 0,03 | -0,01 | 0,80 |
| 16819689 | NDRG family member 4 | NDRG4 | 0,10 | 0,02 | 0,12 | 0,07 |
| 16930262 | TGF-beta activated kinase 1/MAP3K7 binding protein 1 \| uncharacterized LOC100506472 | TAB1\| LOC100506472 | 0,10 | 0,01 | 0,00 | 1,00 |
| 17112588 | X-ray repair complementing defective repair pseudogene | LOC442459 | 0,10 | 0,01 | 0,04 | 0,71 |
| 16866337 | tripartite motif containing 28 | TRIM28 | 0,10 | 0,04 | 0,06 | 0,69 |
| 16914844 | protein tyrosine phosphatase, non-receptor type 1 | PTPN1 | 0,10 | 0,04 | 0,07 | 0,41 |
| 16973914 | STX18 intronic transcript 1 (non-protein coding) | STX18-IT1 | 0,10 | 0,00 | 0,00 | 0,98 |
| 16871846 | zinc finger protein 607 | ZNF607 | 0,10 | 0,01 | 0,19 | 0,09 |
| 16957106 | intraflagellar transport 57 homolog (Chlamydomonas) | IFT57 | 0,10 | 0,01 | 0,13 | 0,28 |
| 16875467 | leukocyte immunoglobulin-like receptor, subfamily A (with TM domain), member 4 | LILRA4 | 0,10 | 0,02 | 0,02 | 0,47 |
| 16988376 | hydroxysteroid (17-beta) dehydrogenase 4 | HSD17B4 | 0,10 | 0,04 | 0,07 | 0,35 |
| 17001654 |  | CD74 | 0,10 | 0,02 | -0,04 | 0,57 |
| 17035971 | major histocompatibility complex, class II, DP beta 1 | HLA-DPB1 | 0,10 | 0,03 | 0,11 | 0,35 |
| 17089233 | GTPase activating Rap/RanGAP domain-like 3 | GARNL3 | 0,10 | 0,02 | -0,04 | 0,73 |
| 16770142 | protein phosphatase 1, catalytic subunit, gamma isozyme | PPP1CC | 0,10 | 0,02 | 0,06 | 0,47 |
| 17072799 | PHD finger protein 20-like 1 | PHF20L1 | 0,10 | 0,02 | 0,10 | 0,14 |
| 16942681 | endogenous Bornavirus-like nucleoprotein 2 | EBLN2 | 0,10 | 0,02 | 0,19 | 0,24 |
| 16731605 | SID1 transmembrane family, member 2 | SIDT2 | 0,09 | 0,04 | -0,05 | 0,16 |
| 16894930 | pumilio homolog 2 (Drosophila) | PUM2 | 0,09 | 0,05 | 0,19 | 0,07 |
| 16952594 | hedgehog acyltransferase-like | HHATL | 0,09 | 0,02 | 0,02 | 0,21 |
| 17078943 | calbindin 1, 28kDa | CALB1 | 0,09 | 0,03 | 0,04 | 0,56 |
| 17027506 | mitochondrial ribosomal protein S18B | MRPS18B | 0,09 | 0,04 | 0,09 | 0,29 |
| 16834302 | ATPase, H+ transporting, lysosomal V0 subunit a1 | ATP6V0A1 | 0,09 | 0,01 | 0,10 | 0,52 |
| 16867541 | dihydrouridine synthase 3-like (S. cerevisiae) | DUS3L | 0,09 | 0,00 | 0,17 | 0,20 |
| 16913095 | centrosomal protein 250kDa | CEP250 | 0,09 | 0,03 | 0,05 | 0,11 |
| 17047918 | carnitine O-octanoyltransferase | CROT | 0,09 | 0,04 | 0,13 | 0,27 |
| 16955442 | ADP-ribosylation factor 4 | ARF4 | 0,09 | 0,03 | -0,01 | 0,92 |
| 16831562 | myosin phosphatase Rho interacting protein | MPRIP | 0,09 | 0,03 | 0,12 | 0,11 |
| 16816739 | ubiquinol-cytochrome c reductase core protein II | UQCRC2 | 0,09 | 0,03 | 0,02 | 0,35 |
| 17080869 | TatD DNase domain containing 1 | TATDN1 | 0,09 | 0,00 | 0,09 | 0,14 |
| 16955155 | selenoprotein K | SELK | 0,09 | 0,01 | 0,14 | 0,30 |
| 17104021 | melanoma antigen family D, 2 | MAGED2 | 0,09 | 0,05 | -0,03 | 0,87 |
| 16927907 | breakpoint cluster region | BCR | 0,09 | 0,00 | 0,09 | 0,12 |
| 16821021 | proteasome (prosome, macropain) 26S subunit, non-ATPase, 7 | PSMD7 | 0,09 | 0,04 | 0,11 | 0,12 |
| 16857676 | calmodulin regulated spectrin-associated protein family, member 3 | CAMSAP3 | 0,09 | 0,02 | 0,16 | 0,06 |
| 16662052 | lymphocyte-specific protein tyrosine kinase | LCK | 0,09 | 0,01 | -0,11 | 0,27 |
| 16721905 | adrenomedullin | ADM | 0,09 | 0,01 | 0,20 | 0,11 |
| 16819217 | metallothionein 1E | MT1E | 0,08 | 0,01 | 0,03 | 0,78 |
| 16781452 | olfactory receptor, family 4, subfamily K, member 1 | OR4K1 | 0,08 | 0,03 | 0,05 | 0,48 |
| 16775623 | POU4F1 antisense RNA 1 (non-protein coding) | POU4F1-AS1 | 0,08 | 0,02 | -0,11 | 0,38 |
| 16739295 | terminal uridylyl transferase 1, U6 snRNA-specific \| microRNA 3654 | TUT1\| MIR3654 | 0,08 | 0,03 | 0,04 | 0,57 |
| 16858263 | queuine tRNA-ribosyltransferase 1 | QTRT1 | 0,08 | 0,02 | 0,04 | 0,75 |
| 16825661 | mitogen-activated protein kinase 3 | MAPK3 | 0,08 | 0,02 | 0,19 | 0,13 |
| 16688753 | far upstream element (FUSE) binding protein 1 | FUBP1 | 0,08 | 0,00 | 0,07 | 0,28 |
| 16933865 | SEC14-like 3 (S. cerevisiae) | SEC14L3 | 0,08 | 0,03 | -0,06 | 0,44 |
| 17007029 | serine/threonine kinase 19 \| serine/threonine kinase 19 pseudogene | STK19\| STK19P | 0,08 | 0,03 | 0,13 | 0,14 |
| 17101129 | ASMTL antisense RNA 1 (non-protein coding) | ASMTL-AS1 | 0,08 | 0,04 | 0,06 | 0,44 |
| 16662968 | CAP, adenylate cyclase-associated protein 1 (yeast) | CAP1 | 0,08 | 0,03 | 0,04 | 0,57 |
| 16991492 | protein phosphatase 1, regulatory (inhibitor) subunit 2 pseudogene 3 | PPP1R2P3 | 0,08 | 0,01 | -0,03 | 0,84 |
| 16775968 | FERM, RhoGEF (ARHGEF) and pleckstrin domain protein 1 (chondrocyte-derived) | FARP1 | 0,08 | 0,02 | 0,11 | 0,12 |
| 16804106 | ADAMTS-like 3 | ADAMTSL3 | 0,08 | 0,03 | 0,08 | 0,32 |
| 16777198 | crystallin, lambda 1 | CRYL1 | 0,07 | 0,04 | 0,31 | 0,17 |
| 16773191 | spermatogenesis associated 13 | SPATA13 | 0,07 | 0,02 | 0,06 | 0,68 |
| 16779280 | WDFY2 antisense RNA 1 (non-protein coding) | WDFY2-AS1 | 0,07 | 0,01 | -0,02 | 0,73 |
| 16996670 | 5-hydroxytryptamine (serotonin) receptor 1A, G protein-coupled | HTR1A | 0,07 | 0,03 | 0,07 | 0,54 |
| 16830103 | rabaptin, RAB GTPase binding effector protein 1 | RABEP1 | 0,07 | 0,01 | 0,10 | 0,20 |
| 16916546 | small nuclear ribonucleoprotein polypeptides B and B1 | SNRPB | 0,07 | 0,00 | 0,16 | 0,18 |
| 16798050 | paternally expressed transcript PAR-SN \| small nuclear ribonucleoprotein polypeptide N \| small nucleolar RNA, C/D box 107 \| SNRPN upstream reading frame \| uncharacterized LOC100506948 \| small nucleolar RNA, C/D box 116-28 \| small nucleolar RNA, C/D box 115-26 \| small nucleolar RNA, C/D box 115-13 \| small nucleolar RNA, C/D box 115-7 | PAR-SN\| SNRPN\| SNORD107\| SNURF\| LOC100506948\| SNORD116-28\| SNORD115-26\| SNORD115-13\| SNORD115-7 | 0,07 | 0,02 | -0,10 | 0,26 |
| 17023066 | centrosomal protein 85kDa-like | CEP85L | 0,07 | 0,04 | 0,03 | 0,57 |
| 16806467 | tight junction protein 1 (zona occludens 1) | TJP1 | 0,07 | 0,00 | 0,15 | 0,24 |
| 16681907 | PRAME family member 13 \| PRAME family member 14 \| PRAME family member 1 \| PRAME family member 2 | PRAMEF13\| PRAMEF14\| PRAMEF2 | 0,07 | 0,03 | 0,04 | 0,39 |
| 16766260 | ATP synthase, H+ transporting, mitochondrial F1 complex, beta polypeptide | ATP5B | 0,07 | 0,04 | 0,02 | 0,43 |
| 16861510 | zinc finger protein 383 | ZNF383 | 0,07 | 0,04 | -0,03 | 0,82 |
| 16730366 | mastermind-like 2 (Drosophila) \| microRNA 1260b | MAML2\| MIR1260B | 0,07 | 0,03 | 0,01 | 0,76 |
| 17115782 | chloride intracellular channel 2 | CLIC2 | 0,07 | 0,01 | 0,08 | 0,13 |
| 16872783 | lipase, hormone-sensitive | LIPE | 0,07 | 0,05 | 0,01 | 0,78 |
| 16755103 | nudix (nucleoside diphosphate linked moiety X)-type motif 4 | NUDT4 | 0,07 | 0,00 | 0,13 | 0,21 |
| 16846084 | mitochondrial ribosomal protein L45 pseudogene 2 | MRPL45P2 | 0,07 | 0,01 | -0,10 | 0,23 |
| 17022163 | prolyl endopeptidase | PREP | 0,07 | 0,02 | 0,03 | 0,82 |
| 17090827 | calcium channel flower domain containing 1 | CACFD1 | 0,06 | 0,01 | 0,11 | 0,29 |
| 16927639 | peptidylprolyl isomerase (cyclophilin)-like 2 | PPIL2 | 0,06 | 0,03 | 0,08 | 0,34 |
| 16689227 | 15 kDa selenoprotein | 41532 | 0,06 | 0,04 | 0,04 | 0,20 |
| 17099816 | NACC family member 2, BEN and BTB (POZ) domain containing | NACC2 | 0,06 | 0,04 | 0,14 | 0,36 |
| 17095450 | iron-sulfur cluster assembly 1 homolog (S. cerevisiae) | ISCA1 | 0,06 | 0,01 | -0,02 | 0,87 |
| 16924537 | ATP synthase, H+ transporting, mitochondrial Fo complex, subunit F6 | ATP5J | 0,06 | 0,04 | 0,00 | 0,99 |
| 17067902 | ER lipid raft associated 2 | ERLIN2 | 0,06 | 0,05 | 0,07 | 0,16 |
| 16819979 | F-box and leucine-rich repeat protein 8 | FBXL8 | 0,06 | 0,03 | 0,05 | 0,61 |
| 16709223 | soc-2 suppressor of clear homolog (C. elegans) | SHOC2 | 0,05 | 0,03 | 0,12 | 0,06 |
| 16980918 | cathepsin O | CTSO | 0,05 | 0,01 | 0,27 | 0,25 |
| 16914867 | par-6 partitioning defective 6 homolog beta (C. elegans) | PARD6B | 0,05 | 0,05 | 0,07 | 0,05 |
| 16694779 | insulin receptor-related receptor | INSRR | 0,05 | 0,02 | -0,03 | 0,88 |
| 17101799 | synapse associated protein 1 | SYAP1 | 0,05 | 0,00 | -0,09 | 0,20 |
| 16884918 | insulin induced gene 2 | INSIG2 | 0,04 | 0,02 | 0,11 | 0,68 |
| 16986450 | angiogenic factor with G patch and FHA domains 1 | AGGF1 | 0,04 | 0,03 | 0,07 | 0,45 |
| 16899686 | gamma-glutamyl carboxylase | GGCX | 0,04 | 0,05 | 0,05 | 0,14 |
| 16841696 | zinc finger, SWIM-type containing 7 | ZSWIM7 | 0,04 | 0,00 | 0,15 | 0,27 |
| 16794542 | acyl-CoA thioesterase 6 | ACOT6 | 0,04 | 0,04 | -0,11 | 0,14 |
| 16867663 | general transcription factor IIF, polypeptide 1, 74kDa | GTF2F1 | 0,04 | 0,05 | 0,08 | 0,53 |
| 16826175 | vacuolar protein sorting 35 homolog (S. cerevisiae) | VPS35 | 0,04 | 0,03 | 0,11 | 0,31 |
| 17087343 | nuclear cap binding protein subunit 1, 80kDa | NCBP1 | 0,04 | 0,03 | 0,02 | 0,64 |
| 16974117 | prosaposin-like 1 (gene/pseudogene) | PSAPL1 | 0,04 | 0,04 | 0,06 | 0,05 |
| 16863404 | uncharacterized LOC93429 | DKFZp434J0226 | 0,04 | 0,02 | -0,02 | 0,80 |
| 17114017 | zinc finger, DHHC-type containing 9 | ZDHHC9 | 0,03 | 0,03 | 0,03 | 0,55 |
| 16687602 | tetratricopeptide repeat domain 22 | TTC22 | 0,03 | 0,04 | -0,02 | 0,89 |
| 16916362 | scratch homolog 2, zinc finger protein (Drosophila) \| sulfiredoxin 1 | SCRT2\| SRXN1 | 0,03 | 0,03 | -0,07 | 0,20 |
| 17015388 | signal sequence receptor, alpha | SSR1 | 0,03 | 0,04 | 0,02 | 0,57 |
| 16760257 | von Willebrand factor | VWF | 0,03 | 0,02 | 0,01 | 0,64 |
| 16889935 | NADH dehydrogenase (ubiquinone) Fe-S protein 1, 75kDa (NADH-coenzyme Q reductase) | NDUFS1 | 0,02 | 0,03 | 0,03 | 0,13 |
| 16712028 | acyl-CoA binding domain containing 7 | ACBD7 | 0,02 | 0,03 | 0,06 | 0,33 |
| 16869350 | deoxyribonuclease II, lysosomal | DNASE2 | 0,02 | 0,02 | -0,02 | 0,69 |
| 16770848 | nitric oxide synthase 1 (neuronal) | NOS1 | 0,01 | 0,01 | -0,05 | 0,44 |

^1^From Student’s t-test.

**Table B. Pathways upregulated in response to S6K1 siRNA, but not to S6K2 siRNA.**

| p-value | Term | Term ID | Term description | Genes |
| --- | --- | --- | --- | --- |
| 1.95e-03 | GO:0043227 | CC | membrane-bounded organelle | NOS1, DNASE2, NDUFS1, VWF, SSR1, ZDHHC9, PSAPL1, NCBP1, VPS35, GTF2F1, ZSWIM7, GGCX, INSIG2, SYAP1, PARD6B, SHOC2, ERLIN2, ATP5J, ISCA1, NACC2, PPIL2, PREP, CLIC2, ZNF383, ATP5B, TJP1, SNRPB, RABEP1, SPATA13, FUBP1, MAPK3, QTRT1, MT1E, LCK, PSMD7, SELK, TATDN1, UQCRC2, ARF4, CROT, ATP6V0A1, CALB1, HHATL, SIDT2, PPP1CC, CD74, HSD17B4, IFT57, ZNF607, PTPN1, TRIM28, TRIP6, IGHMBP2, WDR35, HEXIM1, CUBN, MED25, PADI3, ASPH, ZMYM6, GLRX5, PPP2R4, SALL4, INO80, RAVER2, CCND1, PI4KA, THAP2, NME6, SEC24B, SPRYD4, SEC11A, PDHX, RFX4, OXSM, MNF1, NDUFA12, NT5DC3, TTLL5, COMMD1, ANLN, IVNS1ABP, ECHDC2, UBP1, LAMP1, CRTC3, PDGFRB, PSMD8, SNX27, SGOL1, DDX24, BIRC7, ALS2CL, HOXA6, LMX1B, JKAMP, NOTCH3, S100A6, AKAP10, SLC25A6, SDS, MSMO1, RFC3, NLRX1, PRKCE, RBM25, MYPOP, PHLDA3, UBE3C, PDS5B, SPG21, KANSL2, THOC5, AFTPH, POLB, OSBP, EP400, MARCH5, OS9, EFCAB4B, STOM, XPO5, ZNF451, MUC19, B3GALNT2, TMEM167A, FBXO22, SMAD1, ZIK1, CCT2, MARCH9, FGFR1, GPI, CUL3, RABGAP1L, COX7A2, CLDN19, MTRF1, CTC1, PCNP, FBXL4, GIT2, RNASET2, DHCR7, IRF6, ANO5, MTMR6, MESP2, USP7, NR2C2AP, SEC31B, FNDC3B, PFDN2, HAT1, SFPQ, GOLIM4, POLR2B, TBC1D25, POU5F2, SPEN, FUS, MRPS34, IRF8, NKRF, NOP58, DHFR, TRAPPC2L, PPP1R7, TBX2, SLC26A11, TSN, PMVK, MNX1, COPS2, GORASP1, UBB, ING3, GBA2, SRSF7, PHF19, WDR3, PLBD2, EXT1, FAHD1, SNRPA1, CAMLG, ECI1, MRPL50, TRIM69, DPY30, XRCC6, OVOL1, GOPC, POLR2A, RIPK4, COX14, ZNF137P, B3GNT8, PDIA5, MSH2, CDC5L, GRB14, ZFYVE1, USP28, ZNF143, USO1, PLN, CYP4A22, PBX4, HSD17B10, TSHZ1, ATP5G1, KIF20A, CERK, PDIA4, MNAT1, MAP9, DDX28, ZNF175, HELLS, CEBPZ, TUBD1, LYRM5, ANKRD17, EYA3, CRELD2, NANOGP1, AGPAT4, CAPS, EPB41, RLF, CLCC1, SRSF4, SATB2, COPS5, CKAP4, RAB1A, ZBTB6, KDELR3, GNPTG, IRAK1, HS3ST3A1, BRIX1, APC, ZNF141, NT5C3, NPAT, POLI, TRIB2, NACA2, AFG3L2, RAB12, PTMS, USP15, MLL5, ARID1A, SERGEF, BCORL1, APAF1, CHST15, GAL, DYNLT1, TMX3, LGALS1, RCHY1, COQ10B, ZNF304, ZNF654, ANXA1, ORC4, MIPEP, SYT1, B4GALNT3, ZNF792, KCNH2, KIF4A, FOSL2, PROM2, PLG, ADCK2, RAB31, HDAC5, B3GNT2, COL4A5, HIPK3, AGPS, FMOD |
| 6.21e-07 | BIOGRID:00000 | bi | BioGRID interaction data | NOS1, DNASE2, NDUFS1, VWF, SSR1, ZDHHC9, NCBP1, VPS35, GTF2F1, ZSWIM7, GGCX, AGGF1, INSIG2, SYAP1, INSRR, PARD6B, SHOC2, ERLIN2, ATP5J, ISCA1, NACC2, PPIL2, PREP, LIPE, CLIC2, ZNF383, ATP5B, TJP1, CEP85L, SNRPB, RABEP1, HTR1A, CRYL1, FARP1, CAP1, FUBP1, MAPK3, QTRT1, LCK, CAMSAP3, PSMD7, BCR, MAGED2, TATDN1, UQCRC2, MPRIP, ARF4, CEP250, DUS3L, ATP6V0A1, CALB1, HHATL, PUM2, EBLN2, PHF20L1, PPP1CC, CD74, HSD17B4, IFT57, ZNF607, PTPN1, TRIM28, TRIP6, METTL9, IGHMBP2, WDR35, HEXIM1, CYFIP1, CUBN, MED25, CEP63, LARP1B, ASPH, ZMYM6, PPP2R4, SALL4, THSD7B, INO80, CCND1, TNFRSF11B, PI4KA, THAP2, SEC24B, CCT6A, ADAT1, SEC11A, PDHX, RFX4, NDUFA12, ARHGDIA, TTLL5, RGNEF, COMMD1, G3BP2, ANLN |
| 2.19e-02 | MI:hsa-miR-144 | mi | MI:hsa-miR-144 | ATP5B, TJP1, SNRPB, ADM, TRIP6, CEP63, OXSM, CRTC3, SGOL1, MARCH7, JKAMP, B3GALNT2, SMAD1, CCT2, RABGAP1L, CDNF, HAT1, POLR2B, TBX2, MNX1, COPS2, WDR3, RBM33, XRCC6, ZFAND1, ASNS, MSH2, PGGT1B, RLF, SRSF4, BMPR1A, NPAT, MLL5, GAL, DYNLT1, HIPK3 |
| 2.69e-02 | TF:M00122_2 | tf | Factor: USF; motif: NNRNCACGTGNYNN; match class: 2 | NDUFS1, SSR1, ZDHHC9, NCBP1, TJP1, MAPK3, QTRT1, UQCRC2, ARF4, PHF20L1, NDRG4, TRIP6, CYFIP1, CEP63, ASPH, ZMYM6, GLRX5, CCND1, PI4KA, DMKN, NME6, CCT6A, SPRYD4, TBC1D22B, NT5DC3, CLMP, ANLN, IVNS1ABP, ECHDC2, LAMP1, SGOL1, SCPEP1, CCDC57, SOSTDC1, KREMEN2, JKAMP, PAICS, S100A6, AKAP10, SDS, THOC5, POLB, OSBP, CRB3, B3GALNT2, SMAD1, ZIK1, GPI, NT5DC1, PPA1, DHCR7, GOLIM4, TRAPPC2L, SLC26A11, TSN, TBCEL, MNX1, GNB2, KBTBD3, SRSF7, PLBD2, FAHD1, PDIA5, GRB14, USP28, NGLY1, USO1, PBX4, TSHZ1, PDIA4, MAP9, BCL7C, CCDC71, EPB41L4B, AGPAT4, EPB41, RLF, OSTF1, RIOK2, COPS5, KDELR3, GNPTG, BRIX1, APC, SLC12A7, AHCYL2, ORC4, MIPEP, SYT1, GALK2, RAB31, HDAC5, AGPS |
| 1.20e-02 | TF:M00104_2 | tf | Factor: CDP CR1; motif: NATCGATCGS; match class: 2 | VWF, GGCX, INSIG2, SYAP1, PARD6B, ERLIN2, SELK, GARNL3, METTL9, PPP2R4, NME6, COMMD1, LRCH2, MARCH7, CRB3, PLCD1, TMEM167A, MARCH9, GIT2, ANO5, NOP58, KBTBD3, CDC5L, CYP4A22, GSTA4, OSTF1, COPS5, NT5C3, ARL5B, ADCK2, HDAC5 |
| 1.51e-02 | TF:M00187_0 | tf | Factor: USF; motif: GYCACGTGNC; match class: 0 | SSR1, MAPK3, QTRT1, UQCRC2, PHF20L1, TRIP6, ANLN, LAMP1, SGOL1, S100A6, CRB3, TRAPPC2L, PLBD2, RLF, GNPTG, SLC12A7, RAB31, HDAC5 |

**Table C. Genes downregulated in response to S6K1 siRNA, but not to S6K2 siRNA.**

| Transcripts Cluster ID | Gene description | Gene symbol | S6K1 siRNA  Fold change | S6K1 siRNA  p-value^1^ | S6K2 siRNA  Fold change | S6K2 siRNA  p-value^1^ |
| --- | --- | --- | --- | --- | --- | --- |
| 16670681 | annexin A9 | ANXA9 | -1,11 | 0,04 | -0,69 | 0,13 |
| 16821186 | C-type lectin domain family 3, member A | CLEC3A | -0,86 | 0,02 | -0,21 | 0,08 |
| 16748529 | G protein-coupled receptor, family C, group 5, member A | GPRC5A | -0,80 | 0,00 | -0,22 | 0,15 |
| 16912790 | BPI fold containing family B, member 1 | BPIFB1 | -0,76 | 0,01 | -0,34 | 0,10 |
| 16879721 | endothelial PAS domain protein 1 \| uncharacterized LOC100652809 | EPAS1\| LOC100652809 | -0,69 | 0,02 | -0,17 | 0,09 |
| 16844735 | keratin 13 | KRT13 | -0,67 | 0,01 | -0,61 | 0,05 |
| 16786587 | FBJ murine osteosarcoma viral oncogene homolog | FOS | -0,62 | 0,00 | 0,10 | 0,16 |
| 17118323 | protein kinase, DNA-activated, catalytic polypeptide \| uncharacterized LOC100653330 | PRKDC\| LOC100653330 | -0,59 | 0,04 | -0,50 | 0,15 |
| 16765080 | keratin 4 | KRT4 | -0,59 | 0,01 | -0,31 | 0,14 |
| 16863939 | ferritin, light polypeptide | FTL | -0,58 | 0,01 | -0,10 | 0,22 |
| 16664406 | cytochrome P450, family 4, subfamily Z, polypeptide 1 | CYP4Z1 | -0,56 | 0,01 | -0,23 | 0,05 |
| 16914096 | potassium channel, subfamily K, member 15 \| regulating synaptic membrane exocytosis 4 | KCNK15\| RIMS4 | -0,54 | 0,01 | 0,00 | 1,00 |
| 16681827 | dehydrogenase/reductase (SDR family) member 3 | DHRS3 | -0,53 | 0,02 | -0,09 | 0,52 |
| 16969495 | nephronectin | NPNT | -0,49 | 0,00 | -0,12 | 0,39 |
| 17019877 | cysteine-rich secretory protein 3 | CRISP3 | -0,48 | 0,03 | 0,04 | 0,13 |
| 16863287 | FBJ murine osteosarcoma viral oncogene homolog B | FOSB | -0,47 | 0,01 | -0,02 | 0,62 |
| 16677278 | activating transcription factor 3 | ATF3 | -0,46 | 0,03 | -0,15 | 0,21 |
| 17088164 | solute carrier family 31 (copper transporters), member 2 | SLC31A2 | -0,42 | 0,03 | -0,05 | 0,62 |
| 17020846 | collagen, type XII, alpha 1 | COL12A1 | -0,42 | 0,03 | -0,04 | 0,80 |
| 17100711 | ankyrin repeat domain-containing protein 20B-like | LOC644339 | -0,41 | 0,00 | -0,18 | 0,17 |
| 16903396 | ZEB2 antisense RNA 1 (non-protein coding) | ZEB2-AS1 | -0,41 | 0,02 | -0,17 | 0,35 |
| 16707009 | 3'-phosphoadenosine 5'-phosphosulfate synthase 2 | PAPSS2 | -0,41 | 0,02 | -0,03 | 0,24 |
| 16841561 | peripheral myelin protein 22 | PMP22 | -0,40 | 0,05 | -0,12 | 0,39 |
| 17010703 | protease, serine, 35 | PRSS35 | -0,40 | 0,02 | -0,18 | 0,47 |
| 16774130 | FRAS1 related extracellular matrix protein 2 | FREM2 | -0,40 | 0,02 | -0,13 | 0,15 |
| 16857608 | zinc finger protein 358 | ZNF358 | -0,38 | 0,00 | -0,22 | 0,18 |
| 16803790 | mesoderm development candidate 1 | MESDC1 | -0,38 | 0,04 | 0,02 | 0,79 |
| 16725112 | syntaxin 3 | STX3 | -0,38 | 0,03 | 0,03 | 0,68 |
| 16823928 | lipopolysaccharide-induced TNF factor | LITAF | -0,38 | 0,00 | 0,06 | 0,18 |
| 16805870 | neurobeachin pseudogene 1 | NBEAP1 | -0,38 | 0,04 | -0,18 | 0,62 |
| 17016043 | membrane bound O-acyltransferase domain containing 1 | MBOAT1 | -0,37 | 0,05 | -0,04 | 0,69 |
| 16827679 | NAD(P)H dehydrogenase, quinone 1 | NQO1 | -0,37 | 0,00 | -0,17 | 0,10 |
| 16979917 | solute carrier family 7 (anionic amino acid transporter light chain, xc- system), member 11 | SLC7A11 | -0,36 | 0,02 | -0,08 | 0,68 |
| 16687618 | 24-dehydrocholesterol reductase | DHCR24 | -0,36 | 0,04 | -0,15 | 0,07 |
| 16671139 | S100 calcium binding protein A9 | S100A9 | -0,36 | 0,04 | 0,12 | 0,28 |
| 17116795 | testis-specific transcript, Y-linked 2 (non-protein coding) \| testis-specific transcript, Y-linked 2B (non-protein coding) | TTTY2\| TTTY2B | -0,35 | 0,04 | -0,06 | 0,55 |
| 16692371 | PDZ domain containing 1 pseudogene 1 \| PDZ domain containing 1 pseudogene 2 | PDZK1P1\| PDZK1P2 | -0,35 | 0,01 | -0,26 | 0,07 |
| 16692197 | PDZ domain containing 1 pseudogene 1 \| PDZ domain containing 1 pseudogene 2 | PDZK1P1\| PDZK1P2 | -0,35 | 0,01 | -0,26 | 0,07 |
| 16836021 | ATP-binding cassette, sub-family C (CFTR/MRP), member 3 | ABCC3 | -0,34 | 0,01 | 0,11 | 0,44 |
| 16738599 | olfactory receptor, family 1, subfamily S, member 2 | OR1S2 | -0,33 | 0,02 | -0,27 | 0,42 |
| 16859213 | olfactory receptor, family 10, subfamily H, member 4 | OR10H4 | -0,33 | 0,00 | 0,13 | 0,21 |
| 16798353 | small nuclear ribonucleoprotein polypeptide N \| uncharacterized LOC100506948 \| small nucleolar RNA, C/D box 116-28 \| small nucleolar RNA, C/D box 115-26 \| small nucleolar RNA, C/D box 115-13 \| small nucleolar RNA, C/D box 115-7 \| small nucleolar RNA, C/D box 107 | SNRPN\| LOC100506948\| SNORD116-28\| SNORD115-26\| SNORD115-13\| SNORD115-7\| SNORD107 | -0,33 | 0,04 | -0,22 | 0,10 |
| 16688162 | RNA, U7 small nuclear 62 pseudogene | RNU7-62P | -0,33 | 0,05 | -0,09 | 0,80 |
| 16862408 | cytochrome P450, family 2, subfamily F, polypeptide 1 | CYP2F1 | -0,33 | 0,03 | -0,19 | 0,35 |
| 17025005 | regulator of G-protein signaling 17 | RGS17 | -0,33 | 0,04 | -0,30 | 0,14 |
| 16690789 | DENN/MADD domain containing 2D | DENND2D | -0,32 | 0,01 | -0,03 | 0,85 |
| 16801925 | F-box and leucine-rich repeat protein 22 | FBXL22 | -0,32 | 0,02 | -0,21 | 0,27 |
| 16733040 | uncharacterized LOC403312 | MGC39545 | -0,32 | 0,01 | -0,23 | 0,18 |
| 16919574 | TP53 target 5 | TP53TG5 | -0,31 | 0,05 | -0,14 | 0,29 |
| 17009482 | centromere protein Q | CENPQ | -0,31 | 0,03 | 0,18 | 0,51 |
| 16781846 | T cell receptor alpha variable 7 | TRAV7 | -0,31 | 0,05 | -0,24 | 0,25 |
| 17103254 | sperm acrosome associated 5 \| sperm acrosome associated 5B | SPACA5\| SPACA5B | -0,31 | 0,02 | -0,20 | 0,08 |
| 16779839 | potassium channel tetramerisation domain containing 12 | KCTD12 | -0,31 | 0,03 | 0,00 | 0,99 |
| 16721011 | asparagine-linked glycosylation 1-like pseudogene | LOC650368 | -0,30 | 0,05 | -0,13 | 0,40 |
| 16751438 | nuclear receptor subfamily 4, group A, member 1 | NR4A1 | -0,30 | 0,04 | -0,04 | 0,73 |
| 16716782 | PDZ and LIM domain 1 | PDLIM1 | -0,30 | 0,03 | -0,14 | 0,07 |
| 16806521 | CHRNA7 (cholinergic receptor, nicotinic, alpha 7, exons 5-10) and FAM7A (family with sequence similarity 7A, exons A-E) fusion \| unc-51-like kinase 4 (C. elegans) pseudogene 2 \| unc-51-like kinase 4 (C. elegans) pseudogene 1 | CHRFAM7A\| ULK4P2\| ULK4P1 | -0,30 | 0,02 | -0,44 | 0,23 |
| 17022333 | osteopetrosis associated transmembrane protein 1 | OSTM1 | -0,30 | 0,02 | -0,09 | 0,27 |
| 16944344 | Rho GTPase activating protein 31 | ARHGAP31 | -0,29 | 0,02 | -0,15 | 0,20 |
| 17103141 | zinc finger protein 157 | ZNF157 | -0,29 | 0,00 | -0,23 | 0,16 |
| 16944048 | WDR52 antisense RNA 1 (non-protein coding) | WDR52-AS1 | -0,29 | 0,05 | -0,09 | 0,33 |
| 16743816 | platelet derived growth factor D | PDGFD | -0,29 | 0,01 | 0,05 | 0,72 |
| 16915740 | neurotensin receptor 1 (high affinity) | NTSR1 | -0,29 | 0,04 | 0,02 | 0,76 |
| 17098004 |  | RAB14 | -0,29 | 0,01 | -0,02 | 0,36 |
| 17091502 | prostaglandin D2 synthase 21kDa (brain) | PTGDS | -0,29 | 0,04 | -0,12 | 0,05 |
| 17008515 | translocase of outer mitochondrial membrane 6 homolog (yeast) \| prickle homolog 4 (Drosophila) | TOMM6\| PRICKLE4 | -0,29 | 0,01 | -0,06 | 0,78 |
| 17000210 | SMAD5 antisense RNA 1 (non-protein coding) | SMAD5-AS1 | -0,29 | 0,02 | -0,08 | 0,68 |
| 16757969 | malectin | MLEC | -0,28 | 0,02 | -0,09 | 0,37 |
| 17081067 | transmembrane protein 75 | TMEM75 | -0,28 | 0,02 | -0,08 | 0,40 |
| 16804760 | tubulin tyrosine ligase-like family, member 13 | TTLL13 | -0,28 | 0,03 | -0,28 | 0,09 |
| 16721320 | olfactory receptor, family 56, subfamily A, member 3 | OR56A3 | -0,28 | 0,04 | 0,14 | 0,51 |
| 16764941 | keratin 5 | KRT5 | -0,28 | 0,05 | -0,25 | 0,32 |
| 17112855 | brain expressed, X-linked 5 | BEX5 | -0,28 | 0,04 | -0,21 | 0,10 |
| 16916863 | ring finger protein 24 | RNF24 | -0,28 | 0,04 | -0,04 | 0,78 |
| 16959644 | NME/NM23 family member 9 | NME9 | -0,28 | 0,04 | -0,14 | 0,28 |
| 17040037 | major histocompatibility complex, class II, DP alpha 1 | HLA-DPA1 | -0,28 | 0,00 | -0,33 | 0,15 |
| 16718766 | solute carrier family 18 (vesicular monoamine), member 2 | SLC18A2 | -0,28 | 0,05 | -0,25 | 0,08 |
| 16786104 | DDB1 and CUL4 associated factor 4 | DCAF4 | -0,27 | 0,03 | -0,12 | 0,08 |
| 16761858 | RAS-like, estrogen-regulated, growth inhibitor | RERG | -0,27 | 0,05 | 0,15 | 0,06 |
| 17039217 | corneodesmosin \| psoriasis susceptibility 1 candidate 1 | CDSN\| PSORS1C1 | -0,27 | 0,00 | -0,05 | 0,68 |
| 16874072 | fucosyltransferase 1 (galactoside 2-alpha-L-fucosyltransferase, H blood group) | FUT1 | -0,27 | 0,00 | -0,18 | 0,37 |
| 16839038 | urotensin 2 receptor | UTS2R | -0,27 | 0,02 | -0,01 | 0,55 |
| 16926362 | thrombospondin-type laminin G domain and EAR repeats | TSPEAR | -0,27 | 0,02 | -0,11 | 0,31 |
| 16748304 | GABA(A) receptor-associated protein like 1 | GABARAPL1 | -0,26 | 0,04 | -0,05 | 0,60 |
| 16943596 | coiled-coil domain containing 54 | CCDC54 | -0,26 | 0,03 | -0,11 | 0,31 |
| 16675924 | E74-like factor 3 (ets domain transcription factor, epithelial-specific ) | ELF3 | -0,26 | 0,04 | -0,07 | 0,41 |
| 16967853 | amphiregulin | AREG | -0,26 | 0,01 | 0,26 | 0,18 |
| 17080082 | angiopoietin 1 | ANGPT1 | -0,26 | 0,04 | -0,28 | 0,17 |
| 16672265 | cytochrome c, somatic pseudogene 52 | CYCSP52 | -0,26 | 0,03 | -0,35 | 0,31 |
| 16690388 | solute carrier family 25 (mitochondrial carrier; phosphate carrier), member 24 | SLC25A24 | -0,26 | 0,04 | 0,06 | 0,66 |
| 16927690 | immunoglobulin lambda variable 4-69 | IGLV4-69 | -0,26 | 0,02 | -0,15 | 0,23 |
| 16866041 | zinc finger protein 419 | ZNF419 | -0,26 | 0,01 | -0,18 | 0,26 |
| 16673763 | topoisomerase (DNA) I pseudogene 1 | TOP1P1 | -0,26 | 0,05 | -0,24 | 0,10 |
| 16996433 | polo-like kinase 2 | PLK2 | -0,25 | 0,04 | -0,05 | 0,50 |
| 16731754 | interleukin 10 receptor, alpha | IL10RA | -0,25 | 0,03 | -0,05 | 0,45 |
| 16855510 | ATPase, aminophospholipid transporter, class I, type 8B, member 1 | ATP8B1 | -0,25 | 0,00 | -0,02 | 0,43 |
| 17042275 | 1-acylglycerol-3-phosphate O-acyltransferase 1 (lysophosphatidic acid acyltransferase, alpha) | AGPAT1 | -0,25 | 0,03 | -0,31 | 0,12 |
| 16998368 | endoplasmic reticulum aminopeptidase 1 | ERAP1 | -0,25 | 0,01 | -0,04 | 0,42 |
| 16960371 | WW domain containing transcription regulator 1 | WWTR1 | -0,25 | 0,02 | -0,17 | 0,07 |
| 16784698 | translocase of outer mitochondrial membrane 20 homolog (yeast)-like | TOMM20L | -0,24 | 0,02 | -0,14 | 0,36 |
| 17008325 | glucagon-like peptide 1 receptor | GLP1R | -0,24 | 0,03 | -0,10 | 0,35 |
| 16761238 |  | FLJ46363 | -0,24 | 0,01 | 0,03 | 0,80 |
| 17117841 | AHPA9419 | LOC100131131 | -0,24 | 0,04 | -0,16 | 0,05 |
| 16943879 | transgelin 3 | TAGLN3 | -0,24 | 0,04 | -0,18 | 0,21 |
| 16777380 | RNA, Ro-associated Y3 pseudogene 4 | RNY3P4 | -0,24 | 0,03 | -0,49 | 0,15 |
| 16781092 | ARHGEF7 antisense RNA 1 (non-protein coding) | ARHGEF7-AS1 | -0,24 | 0,00 | -0,22 | 0,30 |
| 16674705 | regulator of G-protein signaling like 1 | RGSL1 | -0,24 | 0,03 | -0,06 | 0,73 |
| 16792938 | FRMD6 antisense RNA 1 (non-protein coding) | FRMD6-AS1 | -0,24 | 0,01 | -0,32 | 0,06 |
| 17039779 | 1-acylglycerol-3-phosphate O-acyltransferase 1 (lysophosphatidic acid acyltransferase, alpha) | AGPAT1 | -0,24 | 0,04 | -0,30 | 0,11 |
| 16679684 | vomeronasal 1 receptor 5 (gene/pseudogene) | VN1R5 | -0,24 | 0,04 | -0,42 | 0,05 |
| 16866667 | proprotein convertase subtilisin/kexin type 4 | PCSK4 | -0,24 | 0,02 | -0,11 | 0,21 |
| 16947809 | leucine-rich repeats and IQ motif containing 4 | LRRIQ4 | -0,24 | 0,02 | -0,14 | 0,14 |
| 16935607 | NFAT activating protein with ITAM motif 1 | NFAM1 | -0,24 | 0,04 | -0,19 | 0,25 |
| 17118432 | retinoblastoma-specific gene 2 | RBSG2 | -0,23 | 0,03 | -0,17 | 0,19 |
| 16945025 | nucleoporin 210kDa pseudogene 1 | NUP210P1 | -0,23 | 0,05 | -0,12 | 0,18 |
| 17107949 | paraneoplastic Ma antigen 3 | PNMA3 | -0,23 | 0,03 | -0,17 | 0,27 |
| 16989955 | IgA-inducing protein homolog (Bos taurus) | IGIP | -0,23 | 0,01 | -0,18 | 0,36 |
| 16740914 | pyruvate carboxylase | PC | -0,23 | 0,03 | -0,21 | 0,11 |
| 16782385 | dehydrogenase/reductase (SDR family) member 2 | DHRS2 | -0,23 | 0,03 | 0,03 | 0,80 |
| 16804778 | neugrin, neurite outgrowth associated | NGRN | -0,23 | 0,01 | 0,02 | 0,91 |
| 16709333 | transcription factor 7-like 2 (T-cell specific, HMG-box) | TCF7L2 | -0,23 | 0,03 | 0,02 | 0,85 |
| 16728991 | potassium voltage-gated channel, Isk-related family, member 3 | KCNE3 | -0,23 | 0,03 | -0,09 | 0,34 |
| 16917949 | cystatin S | CST4 | -0,23 | 0,03 | -0,04 | 0,72 |
| 16899970 | anaphase promoting complex subunit 1 pseudogene | LOC285074 | -0,23 | 0,03 | -0,13 | 0,13 |
| 16734291 | keratin associated protein 5-4 | KRTAP5-4 | -0,23 | 0,05 | 0,03 | 0,95 |
| 16887972 | homeobox D4 \| homeobox D3 | HOXD4\| HOXD3 | -0,23 | 0,03 | -0,13 | 0,30 |
| 16724732 | olfactory receptor, family 4, subfamily P, member 4 | OR4P4 | -0,23 | 0,03 | -0,31 | 0,08 |
| 17063420 | zinc finger CCCH-type, antiviral 1-like | ZC3HAV1L | -0,23 | 0,01 | -0,16 | 0,54 |
| 16988062 |  | DCP2 | -0,22 | 0,01 | -0,01 | 0,92 |
| 17093949 | ring finger protein 38 | RNF38 | -0,22 | 0,03 | 0,01 | 0,94 |
| 17114266 | transcription factor Dp family, member 3 | TFDP3 | -0,22 | 0,00 | -0,22 | 0,24 |
| 17093227 | aquaporin 7 \| aquaporin-7-like | AQP7\| LOC100509620 | -0,22 | 0,00 | -0,20 | 0,39 |
| 17113340 | guanine nucleotide binding protein (G protein), gamma 5 pseudogene 2 | GNG5P2 | -0,22 | 0,04 | -0,09 | 0,45 |
| 16757324 | 2'-5'-oligoadenylate synthetase 1, 40/46kDa | OAS1 | -0,22 | 0,02 | -0,02 | 0,82 |
| 17082982 | dedicator of cytokinesis 8 | DOCK8 | -0,22 | 0,02 | -0,01 | 0,92 |
| 16930166 | apolipoprotein B mRNA editing enzyme, catalytic polypeptide-like 3C | APOBEC3C | -0,22 | 0,01 | -0,02 | 0,93 |
| 16671082 | late cornified envelope 1A | LCE1A | -0,22 | 0,03 | -0,24 | 0,17 |
| 16934681 | transmembrane protease, serine 6 | TMPRSS6 | -0,22 | 0,01 | 0,00 | 0,98 |
| 16799839 | inositol-trisphosphate 3-kinase A | ITPKA | -0,22 | 0,01 | -0,07 | 0,59 |
| 16926445 | pituitary tumor-transforming 1 interacting protein | PTTG1IP | -0,22 | 0,04 | 0,03 | 0,72 |
| 16885619 | cytochrome P450, family 4, subfamily F, polypeptide 30, pseudogene | CYP4F30P | -0,22 | 0,02 | -0,17 | 0,05 |
| 16821193 | WW domain containing oxidoreductase | WWOX | -0,22 | 0,01 | -0,11 | 0,11 |
| 17067963 | eukaryotic translation initiation factor 4E binding protein 1 | EIF4EBP1 | -0,22 | 0,04 | 0,03 | 0,78 |
| 16732734 | SUMO1 activating enzyme subunit 1 pseudogene | LOC341056 | -0,21 | 0,05 | -0,12 | 0,30 |
| 17074371 | sperm associated antigen 11B | SPAG11B | -0,21 | 0,03 | 0,00 | 0,99 |
| 17076773 | solute carrier family 20 (phosphate transporter), member 2 | SLC20A2 | -0,21 | 0,04 | -0,08 | 0,20 |
| 16786607 | Jun dimerization protein 2 | JDP2 | -0,21 | 0,03 | -0,11 | 0,31 |
| 16952534 | cholecystokinin | CCK | -0,21 | 0,04 | -0,17 | 0,09 |
| 16823984 | breast cancer anti-estrogen resistance 4 (non-protein coding) | BCAR4 | -0,21 | 0,01 | -0,04 | 0,81 |
| 17094893 | aldehyde dehydrogenase 1 family, member A1 | ALDH1A1 | -0,21 | 0,03 | -0,08 | 0,11 |
| 17012721 | eyes absent homolog 4 (Drosophila) | EYA4 | -0,21 | 0,03 | -0,03 | 0,32 |
| 16733421 | transmembrane protein 45B | TMEM45B | -0,21 | 0,03 | 0,20 | 0,06 |
| 17004066 | tripartite motif containing 7 | TRIM7 | -0,21 | 0,05 | -0,01 | 0,92 |
| 16819244 | metallothionein 1C, pseudogene | MT1CP | -0,21 | 0,01 | 0,09 | 0,33 |
| 16898764 | small nuclear ribonucleoprotein polypeptide G | SNRPG | -0,21 | 0,02 | -0,28 | 0,15 |
| 16989988 | solute carrier family 4, sodium bicarbonate cotransporter, member 9 | SLC4A9 | -0,20 | 0,02 | -0,16 | 0,25 |
| 16763465 | Rac GTPase activating protein 1 pseudogene | RACGAP1P | -0,20 | 0,02 | -0,22 | 0,20 |
| 17026611 | tripartite motif containing 10 | TRIM10 | -0,20 | 0,01 | -0,09 | 0,15 |
| 17055390 | diacylglycerol kinase, beta 90kDa | DGKB | -0,20 | 0,03 | -0,11 | 0,15 |
| 17043072 | archaelysin family metallopeptidase 1 | AMZ1 | -0,20 | 0,03 | -0,04 | 0,74 |
| 16991151 | synaptopodin | SYNPO | -0,20 | 0,02 | -0,11 | 0,47 |
| 17036227 | tripartite motif containing 10 | TRIM10 | -0,20 | 0,04 | -0,09 | 0,28 |
| 17070480 | REX1, RNA exonuclease 1 homolog (S. cerevisiae)-like 2 (pseudogene) \| REX1, RNA exonuclease 1 homolog (S. cerevisiae)-like 1 \| exonuclease GOR-like | REXO1L2P\| REXO1L1\| LOC100288562 | -0,20 | 0,03 | -0,15 | 0,55 |
| 16862563 | carcinoembryonic antigen-related cell adhesion molecule 6 (non-specific cross reacting antigen) | CEACAM6 | -0,20 | 0,02 | 0,07 | 0,52 |
| 16720049 | ATH1, acid trehalase-like 1 (yeast) | ATHL1 | -0,20 | 0,04 | -0,02 | 0,82 |
| 16988473 | zinc finger protein 474-like \| zinc finger protein 474 | LOC100505841\| ZNF474 | -0,20 | 0,02 | -0,15 | 0,20 |
| 16721247 | olfactory receptor, family 52, subfamily B, member 6 | OR52B6 | -0,20 | 0,03 | -0,28 | 0,08 |
| 16927624 | coiled-coil domain containing 116 | CCDC116 | -0,20 | 0,02 | -0,08 | 0,33 |
| 16832880 | ArfGAP with dual PH domains 2 | ADAP2 | -0,20 | 0,03 | -0,02 | 0,53 |
| 16870594 | hyaluronan and proteoglycan link protein 4 | HAPLN4 | -0,19 | 0,04 | -0,15 | 0,26 |
| 17012281 | tumor protein D52-like 1 | TPD52L1 | -0,19 | 0,04 | -0,06 | 0,42 |
| 17108354 | ATPase, H+ transporting, lysosomal accessory protein 1 | ATP6AP1 | -0,19 | 0,03 | 0,07 | 0,37 |
| 17115677 | GRB2-associated binding protein 3 | GAB3 | -0,19 | 0,01 | -0,16 | 0,11 |
| 17105515 | armadillo repeat containing, X-linked 1 | ARMCX1 | -0,19 | 0,03 | -0,24 | 0,05 |
| 17115669 | cancer/testis antigen 2 | CTAG2 | -0,19 | 0,02 | -0,19 | 0,45 |
| 16993321 | glutamate receptor, metabotropic 6 | GRM6 | -0,19 | 0,03 | 0,05 | 0,34 |
| 16746696 | adiponectin receptor 2 | ADIPOR2 | -0,19 | 0,01 | 0,12 | 0,29 |
| 16932821 | yippee-like 1 (Drosophila) | YPEL1 | -0,19 | 0,04 | -0,14 | 0,42 |
| 16946513 | transient receptor potential cation channel, subfamily C, member 1 | TRPC1 | -0,19 | 0,02 | 0,04 | 0,75 |
| 16840113 | chemokine (C-X-C motif) ligand 16 | CXCL16 | -0,19 | 0,00 | 0,11 | 0,55 |
| 16815828 | class II, major histocompatibility complex, transactivator | CIITA | -0,19 | 0,03 | -0,13 | 0,28 |
| 17108605 | vesicle-associated membrane protein 7 | VAMP7 | -0,19 | 0,03 | -0,11 | 0,33 |
| 16869725 | solute carrier family 1 (high affinity aspartate/glutamate transporter), member 6 | SLC1A6 | -0,18 | 0,03 | 0,00 | 0,96 |
| 17070737 | OTU domain containing 6B | OTUD6B | -0,18 | 0,05 | 0,07 | 0,58 |
| 17024506 | chromosome 15 open reading frame 29 pseudogene | LOC729176 | -0,18 | 0,01 | 0,00 | 0,99 |
| 17116684 | vesicle-associated membrane protein 7 | VAMP7 | -0,18 | 0,03 | -0,12 | 0,25 |
| 16882778 | immunoglobulin kappa constant \| immunoglobulin kappa variable 1D-13 | IGKC\| IGKV1D-13 | -0,18 | 0,04 | -0,13 | 0,38 |
| 16844572 | keratin associated protein 1-1 | KRTAP1-1 | -0,18 | 0,01 | -0,20 | 0,45 |
| 16822478 | jumonji domain containing 8 | JMJD8 | -0,18 | 0,00 | -0,01 | 0,75 |
| 17113518 | chromosome X open reading frame 61 pseudogene | LOC100506955 | -0,18 | 0,03 | -0,06 | 0,60 |
| 17031488 | tripartite motif containing 10 | TRIM10 | -0,18 | 0,04 | -0,08 | 0,19 |
| 16927535 | breakpoint cluster region pseudogene 2 | BCRP2 | -0,18 | 0,01 | -0,12 | 0,41 |
| 16785932 | ADAM metallopeptidase domain 21 | ADAM21 | -0,18 | 0,01 | -0,09 | 0,22 |
| 17028709 | tripartite motif containing 10 | TRIM10 | -0,18 | 0,04 | -0,08 | 0,25 |
| 16842637 | phosphatidylinositol glycan anchor biosynthesis, class S | PIGS | -0,18 | 0,00 | -0,03 | 0,38 |
| 16673126 | regulator of G-protein signaling 4 | RGS4 | -0,18 | 0,05 | -0,33 | 0,10 |
| 17033944 | tripartite motif containing 10 | TRIM10 | -0,18 | 0,04 | -0,08 | 0,22 |
| 16844600 | keratin associated protein 4-11 | KRTAP4-11 | -0,18 | 0,01 | -0,05 | 0,83 |
| 17097150 | olfactory receptor, family 2, subfamily K, member 2 | OR2K2 | -0,18 | 0,01 | -0,12 | 0,61 |
| 16769770 | selectin P ligand | SELPLG | -0,18 | 0,00 | -0,09 | 0,59 |
| 17059756 | cyclin-dependent kinase 6 | CDK6 | -0,17 | 0,00 | 0,09 | 0,15 |
| 17069816 | sulfatase 1 | SULF1 | -0,17 | 0,04 | -0,03 | 0,76 |
| 16725393 | transmembrane protein 109 | TMEM109 | -0,17 | 0,04 | 0,00 | 0,99 |
| 16931237 | fibulin 1 | FBLN1 | -0,17 | 0,02 | -0,06 | 0,23 |
| 17106003 | keratin 18 pseudogene 49 | KRT18P49 | -0,17 | 0,01 | -0,11 | 0,11 |
| 16679650 | saccharopine dehydrogenase (putative) | SCCPDH | -0,17 | 0,03 | 0,06 | 0,20 |
| 16740659 | cation channel, sperm associated 1 | CATSPER1 | -0,17 | 0,03 | -0,22 | 0,11 |
| 17055501 | anterior gradient 2 homolog (Xenopus laevis) | AGR2 | -0,17 | 0,05 | -0,09 | 0,27 |
| 16695730 | Fc fragment of IgG, low affinity IIb, receptor (CD32) | FCGR2B | -0,17 | 0,05 | -0,14 | 0,07 |
| 16874656 | kallikrein-related peptidase 15 | KLK15 | -0,17 | 0,01 | -0,20 | 0,29 |
| 16721549 | NLR family, pyrin domain containing 14 | NLRP14 | -0,17 | 0,02 | -0,09 | 0,16 |
| 16908483 |  | FEV | -0,17 | 0,04 | -0,07 | 0,41 |
| 17016461 | POM121 transmembrane nucleoporin-like 2 | POM121L2 | -0,17 | 0,02 | -0,25 | 0,10 |
| 16664978 | acyl-CoA thioesterase 11 | ACOT11 | -0,17 | 0,04 | -0,06 | 0,62 |
| 17040221 | TAP binding protein (tapasin) | TAPBP | -0,17 | 0,01 | 0,10 | 0,20 |
| 17057879 | human papillomavirus (type 18) E5 central sequence-like 1 | HPVC1 | -0,16 | 0,03 | -0,09 | 0,26 |
| 16900148 | immunoglobulin kappa variable 2-24 | IGKV2-24 | -0,16 | 0,02 | -0,23 | 0,09 |
| 17067566 | RNA binding protein with multiple splicing | RBPMS | -0,16 | 0,01 | 0,02 | 0,70 |
| 16724779 | olfactory receptor, family 5, subfamily T, member 1 | OR5T1 | -0,16 | 0,04 | -0,06 | 0,70 |
| 17118026 | H6 family homeobox 1 | HMX1 | -0,16 | 0,01 | -0,06 | 0,20 |
| 16833345 | adaptor-related protein complex 2, beta 1 subunit | AP2B1 | -0,16 | 0,01 | -0,01 | 0,95 |
| 16891629 | neuronal tyrosine-phosphorylated phosphoinositide-3-kinase adaptor 2 | NYAP2 | -0,16 | 0,05 | -0,04 | 0,71 |
| 16782615 | proteasome (prosome, macropain) activator subunit 1 (PA28 alpha) | PSME1 | -0,16 | 0,01 | -0,04 | 0,21 |
| 16845794 | kinesin family member 18B | KIF18B | -0,16 | 0,02 | 0,03 | 0,80 |
| 16906267 | zinc finger, SWIM-type containing 2 | ZSWIM2 | -0,16 | 0,03 | -0,13 | 0,14 |
| 16801573 | lactate dehydrogenase A-like 6B | LDHAL6B | -0,16 | 0,03 | -0,33 | 0,25 |
| 17086193 | phosphoserine aminotransferase 1 | PSAT1 | -0,16 | 0,01 | 0,00 | 0,98 |
| 17005569 | histone cluster 1, H1e | HIST1H1E | -0,16 | 0,03 | 0,05 | 0,55 |
| 16929562 | heme oxygenase (decycling) 1 | HMOX1 | -0,16 | 0,02 | -0,23 | 0,10 |
| 17113104 | ripply1 homolog (zebrafish) | RIPPLY1 | -0,16 | 0,02 | -0,45 | 0,11 |
| 17004198 | forkhead box F2 | FOXF2 | -0,16 | 0,02 | 0,00 | 0,95 |
| 17104106 | P antigen family, member 5 (prostate associated) | PAGE5 | -0,16 | 0,04 | 0,03 | 0,86 |
| 17028632 | zinc finger protein 57 homolog (mouse) | ZFP57 | -0,16 | 0,00 | -0,03 | 0,71 |
| 16863091 | poliovirus receptor-related 2 (herpesvirus entry mediator B) | PVRL2 | -0,16 | 0,04 | 0,02 | 0,81 |
| 16910006 |  | FLJ43879 | -0,16 | 0,01 | -0,01 | 0,79 |
| 17095423 | golgi membrane protein 1 | GOLM1 | -0,16 | 0,02 | 0,04 | 0,43 |
| 16962185 | asparagine-linked glycosylation 3, alpha-1,3- mannosyltransferase homolog (S. cerevisiae) | ALG3 | -0,15 | 0,01 | -0,21 | 0,11 |
| 17004518 | lymphocyte antigen 86 | LY86 | -0,15 | 0,05 | -0,06 | 0,44 |
| 17116641 | chondroitin sulfate proteoglycan 4 pseudogene 1, Y-linked \| DNM1 pseudogene 24 \| chondroitin sulfate proteoglycan 4 pseudogene 3, Y-linked \| DNM1 pseudogene 48 | CSPG4P1Y\| DNM1P24\| CSPG4P3Y\| DNM1P48 | -0,15 | 0,00 | -0,13 | 0,09 |
| 16754471 | neuron navigator 3 | NAV3 | -0,15 | 0,04 | -0,13 | 0,35 |
| 16994960 | golgi phosphoprotein 3 (coat-protein) | GOLPH3 | -0,15 | 0,02 | -0,04 | 0,51 |
| 16763512 | solute carrier family 38, member 1 | SLC38A1 | -0,15 | 0,04 | 0,07 | 0,31 |
| 16782637 | ER membrane protein complex subunit 9 | EMC9 | -0,15 | 0,03 | -0,14 | 0,28 |
| 16962632 | leprecan-like 1 | LEPREL1 | -0,15 | 0,00 | -0,02 | 0,44 |
| 16933265 | low density lipoprotein receptor-related protein 5-like | LRP5L | -0,15 | 0,03 | 0,18 | 0,05 |
| 16902623 | Sin3A-associated protein, 130kDa | SAP130 | -0,15 | 0,01 | -0,08 | 0,24 |
| 17019134 | ubiquitin specific peptidase 49 | USP49 | -0,15 | 0,02 | -0,06 | 0,44 |
| 17030643 | allograft inflammatory factor 1 | AIF1 | -0,15 | 0,03 | -0,11 | 0,07 |
| 16991991 | WW and C2 domain containing 1 | WWC1 | -0,15 | 0,04 | 0,06 | 0,62 |
| 16991192 | glutathione peroxidase 3 (plasma) | GPX3 | -0,15 | 0,00 | 0,04 | 0,43 |
| 17018615 | ets variant 7 | ETV7 | -0,15 | 0,02 | -0,13 | 0,10 |
| 16659238 | tumor necrosis factor receptor superfamily, member 1B \| microRNA 4632 | TNFRSF1B\| MIR4632 | -0,15 | 0,00 | -0,03 | 0,06 |
| 16851680 | CHST9 antisense RNA 1 (non-protein coding) | CHST9-AS1 | -0,15 | 0,03 | -0,02 | 0,91 |
| 16977868 | ATP-binding cassette, sub-family G (WHITE), member 2 | ABCG2 | -0,15 | 0,03 | -0,10 | 0,37 |
| 17117414 | INM04 | LOC100128751 | -0,15 | 0,04 | 0,00 | 1,00 |
| 17072047 | RAD21 antisense RNA 1 (non-protein coding) \| RAD21 homolog (S. pombe) | RAD21-AS1 | -0,15 | 0,02 | -0,20 | 0,14 |
| 17059828 | tissue factor pathway inhibitor 2 | TFPI2 | -0,15 | 0,00 | 0,22 | 0,07 |
| 16857897 |  | RAB11B | -0,15 | 0,02 | -0,16 | 0,31 |
| 17109885 | nuclear receptor subfamily 0, group B, member 1 | NR0B1 | -0,15 | 0,00 | -0,27 | 0,18 |
| 16801850 |  | RAB8B | -0,14 | 0,01 | 0,08 | 0,46 |
| 16699209 | Usher syndrome 2A (autosomal recessive, mild) | USH2A | -0,14 | 0,04 | -0,06 | 0,56 |
| 16795075 | neuroglobin | NGB | -0,14 | 0,03 | -0,06 | 0,70 |
| 16922114 | hormonally up-regulated Neu-associated kinase | HUNK | -0,14 | 0,04 | 0,00 | 0,95 |
| 16690618 | G protein-coupled receptor 61 | GPR61 | -0,14 | 0,03 | -0,09 | 0,35 |
| 17117337 | chondroitin sulfate proteoglycan 4 pseudogene 1, Y-linked \| DNM1 pseudogene 24 \| chondroitin sulfate proteoglycan 4 pseudogene 3, Y-linked \| DNM1 pseudogene 48 | CSPG4P1Y\| DNM1P24\| CSPG4P3Y\| DNM1P48 | -0,14 | 0,01 | -0,12 | 0,09 |
| 16979875 | protocadherin 18 | PCDH18 | -0,14 | 0,01 | -0,20 | 0,22 |
| 16908502 | coiled-coil domain containing 108 | CCDC108 | -0,14 | 0,05 | -0,08 | 0,40 |
| 16912068 | cystatin-like 1 | CSTL1 | -0,14 | 0,05 | -0,06 | 0,66 |
| 16691929 | uncharacterized LOC728855 \| uncharacterized LOC728875 | LOC728855\| LOC728875 | -0,14 | 0,03 | -0,07 | 0,42 |
| 16874453 | sialic acid binding Ig-like lectin 11 | SIGLEC11 | -0,14 | 0,01 | -0,14 | 0,21 |
| 16785838 | pleckstrin homology domain containing, family D (with coiled-coil domains) member 1 | PLEKHD1 | -0,14 | 0,05 | -0,08 | 0,42 |
| 16752147 | neuronal differentiation 4 | NEUROD4 | -0,14 | 0,02 | -0,13 | 0,13 |
| 16698779 | complement component (3b/4b) receptor 1 (Knops blood group) | CR1 | -0,14 | 0,02 | -0,16 | 0,27 |
| 17117567 | sodium channel, voltage gated, type VIII, alpha subunit | SCN8A | -0,13 | 0,01 | -0,11 | 0,28 |
| 16883325 | inositol polyphosphate-4-phosphatase, type I, 107kDa | INPP4A | -0,13 | 0,02 | 0,03 | 0,81 |
| 17068014 | DDHD domain containing 2 | DDHD2 | -0,13 | 0,03 | 0,01 | 0,85 |
| 16963949 | UV-stimulated scaffold protein A | UVSSA | -0,13 | 0,02 | 0,14 | 0,40 |
| 17058226 | RAB guanine nucleotide exchange factor (GEF) 1 pseudogene | LOC493754 | -0,13 | 0,00 | 0,08 | 0,42 |
| 16831702 | myosin XVA | MYO15A | -0,13 | 0,03 | -0,06 | 0,14 |
| 16779119 | karyopherin alpha 3 (importin alpha 4) | KPNA3 | -0,13 | 0,04 | 0,06 | 0,37 |
| 17088730 | MORN repeat containing 5 | MORN5 | -0,13 | 0,03 | -0,06 | 0,11 |
| 16870854 | zinc finger protein 676 | ZNF676 | -0,13 | 0,04 | 0,00 | 0,94 |
| 16783473 | paired box 9 | PAX9 | -0,13 | 0,05 | 0,00 | 0,96 |
| 16965942 | TBC1 (tre-2/USP6, BUB2, cdc16) domain family, member 1 | TBC1D1 | -0,13 | 0,04 | -0,01 | 0,86 |
| 16820849 | calbindin 2 | CALB2 | -0,13 | 0,03 | -0,02 | 0,60 |
| 17050959 | hyaluronoglucosaminidase 4 | HYAL4 | -0,13 | 0,04 | -0,16 | 0,19 |
| 16986423 | S100 calcium binding protein Z | S100Z | -0,13 | 0,01 | -0,11 | 0,23 |
| 16897159 | SIX homeobox 2 | SIX2 | -0,13 | 0,01 | -0,12 | 0,32 |
| 17117229 | PTPN13-like, Y-linked \| PTPN13-like, Y-linked 2 \| PTPN13-like protein, Y-linked-like | PRY\| PRY2\| LOC100509646 | -0,13 | 0,04 | -0,16 | 0,12 |
| 16821437 | dynein, axonemal, assembly factor 1 | DNAAF1 | -0,13 | 0,01 | -0,16 | 0,22 |
| 16987795 | uncharacterized LOC100652853 | HP07349 | -0,13 | 0,00 | -0,06 | 0,05 |
| 16810341 | carbonic anhydrase XII | CA12 | -0,13 | 0,00 | 0,11 | 0,26 |
| 16714409 | mannose-binding lectin (protein C) 2, soluble | MBL2 | -0,13 | 0,04 | 0,11 | 0,19 |
| 17014421 | calcium binding protein P22 pseudogene | LOC729603 | -0,12 | 0,05 | -0,17 | 0,17 |
| 17113144 | FERM and PDZ domain containing 3 | FRMPD3 | -0,12 | 0,03 | -0,29 | 0,09 |
| 16834218 | kelch-like 10 (Drosophila) | KLHL10 | -0,12 | 0,00 | -0,13 | 0,15 |
| 16946886 | TSC22 domain family, member 2 | TSC22D2 | -0,12 | 0,00 | 0,12 | 0,48 |
| 16871935 | gametogenetin | GGN | -0,12 | 0,01 | -0,14 | 0,22 |
| 16930831 | WBP2 N-terminal like | WBP2NL | -0,12 | 0,01 | -0,01 | 0,92 |
| 17019654 | chloride intracellular channel 5 | CLIC5 | -0,12 | 0,05 | -0,12 | 0,23 |
| 16830402 | transmembrane protein 95 | TMEM95 | -0,12 | 0,02 | -0,09 | 0,53 |
| 16920744 | GNAS antisense RNA 1 (non-protein coding) | GNAS-AS1 | -0,12 | 0,02 | 0,01 | 0,88 |
| 16957200 | resistin like beta | RETNLB | -0,12 | 0,02 | 0,15 | 0,46 |
| 16661934 | transmembrane protein 39B | TMEM39B | -0,12 | 0,01 | -0,06 | 0,13 |
| 17100277 | fucosyltransferase 7 (alpha (1,3) fucosyltransferase) | FUT7 | -0,12 | 0,00 | -0,06 | 0,26 |
| 16835497 | ubiquitin-conjugating enzyme E2Z | UBE2Z | -0,12 | 0,01 | -0,05 | 0,42 |
| 17085760 | MAM domain containing 2 | MAMDC2 | -0,12 | 0,03 | -0,11 | 0,20 |
| 16844653 | keratin 33B | KRT33B | -0,12 | 0,01 | -0,01 | 0,97 |
| 17094946 | transient receptor potential cation channel, subfamily M, member 6 | TRPM6 | -0,11 | 0,02 | -0,34 | 0,05 |
| 16667948 | VAV3 antisense RNA 1 (non-protein coding) | VAV3-AS1 | -0,11 | 0,04 | -0,04 | 0,60 |
| 17109849 | aristaless related homeobox | ARX | -0,11 | 0,02 | -0,04 | 0,43 |
| 16774598 | leucine rich repeat containing 63 | LRRC63 | -0,11 | 0,03 | -0,15 | 0,25 |
| 16881951 | regenerating islet-derived 3 gamma | REG3G | -0,11 | 0,01 | -0,06 | 0,51 |
| 16716590 | myoferlin | MYOF | -0,11 | 0,05 | 0,05 | 0,18 |
| 16844712 | keratin 35 | KRT35 | -0,11 | 0,00 | 0,00 | 0,99 |
| 16759855 | ninjurin 2 | NINJ2 | -0,11 | 0,03 | -0,02 | 0,87 |
| 16819202 | metallothionein 3 | MT3 | -0,11 | 0,05 | -0,14 | 0,12 |
| 16702026 | aldo-keto reductase family 1, member C4 (chlordecone reductase; 3-alpha hydroxysteroid dehydrogenase, type I; dihydrodiol dehydrogenase 4) | AKR1C4 | -0,11 | 0,01 | 0,07 | 0,65 |
| 16844853 | huntingtin-associated protein 1 | HAP1 | -0,11 | 0,01 | -0,10 | 0,42 |
| 16941693 | signal peptidase complex subunit 1 homolog (S. cerevisiae) | SPCS1 | -0,11 | 0,05 | -0,12 | 0,24 |
| 16777448 | SPATA13 antisense RNA 1 (non-protein coding) | SPATA13-AS1 | -0,11 | 0,04 | 0,15 | 0,37 |
| 16933350 | tuftelin interacting protein 11 | TFIP11 | -0,11 | 0,02 | -0,10 | 0,53 |
| 16765648 | dermcidin | DCD | -0,11 | 0,04 | -0,06 | 0,49 |
| 16762657 | kelch domain containing 5 | KLHDC5 | -0,11 | 0,00 | 0,00 | 0,99 |
| 16769761 | transmembrane protein 119 | TMEM119 | -0,11 | 0,03 | -0,09 | 0,15 |
| 16920497 | cytochrome P450, family 24, subfamily A, polypeptide 1 | CYP24A1 | -0,11 | 0,02 | 0,04 | 0,73 |
| 16780203 | GPC6 antisense RNA 1 (non-protein coding) | GPC6-AS1 | -0,11 | 0,03 | -0,03 | 0,74 |
| 16827381 | agouti related protein homolog (mouse) | AGRP | -0,11 | 0,02 | -0,15 | 0,20 |
| 16707983 | zinc finger, DHHC-type containing 16 | ZDHHC16 | -0,11 | 0,00 | 0,11 | 0,12 |
| 17014442 | solute carrier family 22 (extraneuronal monoamine transporter), member 3 | SLC22A3 | -0,11 | 0,02 | -0,08 | 0,34 |
| 16873495 | neuro-oncological ventral antigen 2 | NOVA2 | -0,11 | 0,00 | -0,12 | 0,48 |
| 17018025 | major histocompatibility complex, class II, DO alpha | HLA-DOA | -0,11 | 0,01 | 0,02 | 0,77 |
| 17048538 | CAS1 domain containing 1 | CASD1 | -0,11 | 0,03 | 0,02 | 0,79 |
| 17000294 | heterogeneous nuclear ribonucleoprotein A0 | HNRNPA0 | -0,11 | 0,04 | 0,14 | 0,05 |
| 17058083 | zinc finger protein 479 pseudogene | LOC643955 | -0,11 | 0,04 | -0,25 | 0,27 |
| 16805422 | nuclear receptor subfamily 2, group F, member 2 | NR2F2 | -0,10 | 0,03 | -0,08 | 0,59 |
| 17025775 | FERM domain containing 1 | FRMD1 | -0,10 | 0,02 | -0,24 | 0,08 |
| 16900030 | fatty acid binding protein 1, liver | FABP1 | -0,10 | 0,03 | -0,17 | 0,09 |
| 16783411 | nuclear factor of kappa light polypeptide gene enhancer in B-cells inhibitor, alpha \| uncharacterized LOC100289251 | NFKBIA\| LOC100289251 | -0,10 | 0,01 | -0,04 | 0,71 |
| 16746714 | leucine-rich repeats and transmembrane domains 2 | LRTM2 | -0,10 | 0,02 | -0,16 | 0,31 |
| 16780133 | SLIT and NTRK-like family, member 6 | SLITRK6 | -0,10 | 0,03 | -0,19 | 0,28 |
| 16863148 | cleft lip and palate associated transmembrane protein 1 | CLPTM1 | -0,10 | 0,02 | -0,02 | 0,05 |
| 17037532 | zinc finger and BTB domain containing 22 | ZBTB22 | -0,10 | 0,05 | -0,14 | 0,11 |
| 16885526 |  | IMP4 | -0,10 | 0,05 | -0,02 | 0,74 |
| 17020964 | interphotoreceptor matrix proteoglycan 1 | IMPG1 | -0,10 | 0,02 | -0,08 | 0,14 |
| 16751518 | keratin 86 | KRT86 | -0,10 | 0,01 | 0,05 | 0,63 |
| 16822762 | intraflagellar transport 140 homolog (Chlamydomonas) | IFT140 | -0,10 | 0,00 | 0,03 | 0,65 |
| 16832570 | NIMA (never in mitosis gene a)- related kinase 8 | NEK8 | -0,10 | 0,01 | -0,05 | 0,18 |
| 16951455 | EF-hand domain family, member B | EFHB | -0,10 | 0,02 | -0,17 | 0,11 |
| 16760668 | lysophosphatidylcholine acyltransferase 3 | LPCAT3 | -0,10 | 0,03 | 0,05 | 0,28 |
| 17006616 | MHC class I polypeptide-related sequence B | MICB | -0,10 | 0,04 | -0,02 | 0,80 |
| 16999799 | interleukin 5 (colony-stimulating factor, eosinophil) | IL5 | -0,10 | 0,02 | -0,17 | 0,16 |
| 17116550 | deleted in azoospermia 2 \| deleted in azoospermia 4 \| deleted in azoospermia 3 \| deleted in azoospermia 1 | DAZ2\| DAZ4\| DAZ3\| DAZ1 | -0,09 | 0,01 | -0,11 | 0,44 |
| 16818165 | branched chain ketoacid dehydrogenase kinase | BCKDK | -0,09 | 0,02 | -0,05 | 0,49 |
| 17104223 | spindlin family, member 4 | SPIN4 | -0,09 | 0,02 | -0,07 | 0,26 |
| 16707567 | omega-3 fatty acid receptor 1 | O3FAR1 | -0,09 | 0,04 | -0,08 | 0,21 |
| 16877097 | ATPase, H+ transporting, lysosomal 42kDa, V1 subunit C2 | ATP6V1C2 | -0,09 | 0,03 | -0,14 | 0,53 |
| 16679375 | WD repeat domain 64 | WDR64 | -0,09 | 0,00 | 0,03 | 0,73 |
| 17034090 | mediator of DNA-damage checkpoint 1 | MDC1 | -0,09 | 0,02 | 0,04 | 0,61 |
| 16840155 | profilin 1 | PFN1 | -0,09 | 0,01 | -0,12 | 0,20 |
| 16975634 | cytochrome c oxidase subunit VIIb2 | COX7B2 | -0,09 | 0,04 | -0,15 | 0,40 |
| 16845681 | integrin, alpha 2b (platelet glycoprotein IIb of IIb/IIIa complex, antigen CD41) | ITGA2B | -0,09 | 0,02 | -0,13 | 0,09 |
| 16876303 | proline-rich nuclear receptor coactivator 2 | PNRC2 | -0,09 | 0,01 | -0,16 | 0,22 |
| 16752368 | IKAROS family zinc finger 4 (Eos) | IKZF4 | -0,09 | 0,01 | 0,05 | 0,07 |
| 17059911 | paraoxonase 3 \| paraoxonase 1 | PON3\| PON1 | -0,09 | 0,01 | -0,18 | 0,05 |
| 16814001 | threonyl-tRNA synthetase-like 2 | TARSL2 | -0,09 | 0,00 | -0,16 | 0,07 |
| 17066330 | LZTS1 antisense RNA 1 (non-protein coding) | LZTS1-AS1 | -0,09 | 0,04 | -0,01 | 0,87 |
| 16873404 | SIX homeobox 5 | SIX5 | -0,09 | 0,03 | -0,15 | 0,16 |
| 17040670 | MHC class I polypeptide-related sequence B | MICB | -0,09 | 0,05 | -0,03 | 0,73 |
| 16804453 | NTRK3 antisense RNA 1 (non-protein coding) | NTRK3-AS1 | -0,09 | 0,05 | -0,13 | 0,41 |
| 17010782 | 5-hydroxytryptamine (serotonin) receptor 1E, G protein-coupled | HTR1E | -0,09 | 0,03 | -0,03 | 0,78 |
| 16962661 | claudin 1 | CLDN1 | -0,08 | 0,04 | 0,03 | 0,45 |
| 16798938 | secretogranin V (7B2 protein) | SCG5 | -0,08 | 0,01 | -0,01 | 0,84 |
| 16830257 | RNASEK-C17orf49 readthrough \| ribonuclease, RNase K \| chromosome 17 open reading frame 49 | RNASEK-C17ORF49\| C17orf49 | -0,08 | 0,03 | -0,14 | 0,41 |
| 16789723 | cysteine-rich protein 2 | CRIP2 | -0,08 | 0,01 | -0,06 | 0,08 |
| 17078452 | hairy/enhancer-of-split related with YRPW motif 1 | HEY1 | -0,08 | 0,00 | -0,15 | 0,44 |
| 16862344 | MIA-RAB4B readthrough \| RAB4B-EGLN2 readthrough \| melanoma inhibitory activity \| egl nine homolog 2 (C. elegans) \| RAB4B, member RAS oncogene family | MIA-RAB4B\| RAB4B-EGLN2 | -0,08 | 0,04 | -0,05 | 0,42 |
| 17064054 | thiamin pyrophosphokinase 1 | TPK1 | -0,08 | 0,03 | 0,08 | 0,46 |
| 16835374 | nuclear factor (erythroid-derived 2)-like 1 | NFE2L1 | -0,08 | 0,00 | -0,02 | 0,62 |
| 16663133 | potassium voltage-gated channel, KQT-like subfamily, member 4 | KCNQ4 | -0,08 | 0,03 | 0,13 | 0,53 |
| 16729155 | monoacylglycerol O-acyltransferase 2 | MOGAT2 | -0,08 | 0,04 | 0,09 | 0,46 |
| 17058484 | B-cell CLL/lymphoma 7B | BCL7B | -0,08 | 0,02 | 0,00 | 0,91 |
| 16757616 | microtubule-associated protein 1 light chain 3 beta 2 | MAP1LC3B2 | -0,08 | 0,00 | 0,11 | 0,65 |
| 16956316 | contactin 3 (plasmacytoma associated) | CNTN3 | -0,07 | 0,02 | 0,06 | 0,47 |
| 17046920 | FK506 binding protein 6, 36kDa | FKBP6 | -0,07 | 0,01 | -0,15 | 0,31 |
| 16840876 | vesicle-associated membrane protein 2 (synaptobrevin 2) | VAMP2 | -0,07 | 0,04 | 0,01 | 0,89 |
| 16813148 | kinesin family member 7 | KIF7 | -0,07 | 0,02 | -0,05 | 0,60 |
| 17087413 | UDP-N-acetyl-alpha-D-galactosamine:polypeptide N-acetylgalactosaminyltransferase 12 (GalNAc-T12) | GALNT12 | -0,07 | 0,04 | 0,21 | 0,28 |
| 16937252 | myotubularin related protein 14 | MTMR14 | -0,07 | 0,04 | 0,01 | 0,71 |
| 16799776 | OIP5 antisense RNA 1 (non-protein coding) | OIP5-AS1 | -0,07 | 0,04 | 0,01 | 0,91 |
| 16986351 | IQ motif containing GTPase activating protein 2 | IQGAP2 | -0,07 | 0,02 | -0,08 | 0,11 |
| 16774614 | RNA, U6 small nuclear 68 | RNU6-68 | -0,07 | 0,02 | -0,08 | 0,28 |
| 17068158 | HtrA serine peptidase 4 | HTRA4 | -0,07 | 0,04 | -0,08 | 0,10 |
| 16735801 | eukaryotic translation initiation factor 4 gamma, 2 | EIF4G2 | -0,07 | 0,03 | -0,11 | 0,15 |
| 16767422 | protein tyrosine phosphatase, receptor type, B | PTPRB | -0,07 | 0,02 | -0,03 | 0,69 |
| 17020733 | KCNQ5 antisense RNA 2 (non-protein coding) | KCNQ5-AS2 | -0,07 | 0,02 | -0,13 | 0,16 |
| 16952782 | transmembrane protein 158 (gene/pseudogene) | TMEM158 | -0,06 | 0,04 | -0,08 | 0,07 |
| 16724795 | olfactory receptor, family 5, subfamily AR, member 1 | OR5AR1 | -0,06 | 0,01 | -0,10 | 0,46 |
| 17009731 | MLIP intronic transcript 1 (non-protein coding) | MLIP-IT1 | -0,06 | 0,02 | -0,07 | 0,57 |
| 16672847 | translocase of outer mitochondrial membrane 40 homolog (yeast)-like | TOMM40L | -0,06 | 0,03 | 0,10 | 0,06 |
| 17085868 | transmembrane channel-like 1 | TMC1 | -0,06 | 0,02 | 0,09 | 0,69 |
| 16874432 | interleukin 4 induced 1 \| nucleoporin 62kDa | IL4I1\| NUP62 | -0,06 | 0,00 | 0,00 | 0,71 |
| 16761523 | taste receptor, type 2, member 43 | TAS2R43 | -0,06 | 0,00 | -0,09 | 0,36 |
| 16702007 | aldo-keto reductase family 1, member C3 (3-alpha hydroxysteroid dehydrogenase, type II) | AKR1C3 | -0,06 | 0,01 | 0,08 | 0,44 |
| 16705295 | leucine rich repeat transmembrane neuronal 3 | LRRTM3 | -0,06 | 0,03 | -0,03 | 0,80 |
| 16825057 | golgi-associated, gamma adaptin ear containing, ARF binding protein 2 | GGA2 | -0,06 | 0,04 | 0,04 | 0,42 |
| 17093300 | suppressor of G2 allele of SKP1 (S. cerevisiae) pseudogene 1 | SUGT1P1 | -0,06 | 0,02 | -0,13 | 0,24 |
| 16991797 | gamma-aminobutyric acid (GABA) A receptor, alpha 1 | GABRA1 | -0,05 | 0,02 | -0,02 | 0,83 |
| 17007824 | Fanconi anemia, complementation group E | FANCE | -0,05 | 0,04 | 0,03 | 0,81 |
| 17029003 | major histocompatibility complex, class I, C | HLA-C | -0,05 | 0,05 | 0,09 | 0,35 |
| 16972824 | storkhead box 2 | STOX2 | -0,05 | 0,02 | 0,08 | 0,14 |
| 16917533 | ovo-like 2 (Drosophila) | OVOL2 | -0,05 | 0,04 | 0,18 | 0,46 |
| 17085561 | COBW domain containing 7 \| COBW domain containing 3 \| COBW domain containing 5 \| COBW domain containing 1 \| COBW domain containing 6 \| COBW domain containing 2 \| uncharacterized LOC100653334 | CBWD7\| CBWD3\| CBWD5\| CBWD1\| CBWD6\| CBWD2\| LOC100653334 | -0,05 | 0,03 | 0,02 | 0,64 |
| 16823169 | protease, serine 27 | PRSS27 | -0,05 | 0,03 | 0,00 | 0,93 |
| 17016457 | histone cluster 1, H2bk | HIST1H2BK | -0,05 | 0,02 | 0,00 | 0,96 |
| 16725969 | transmembrane protein 179B | TMEM179B | -0,04 | 0,03 | 0,04 | 0,72 |
| 16827606 | sphingomyelin phosphodiesterase 3, neutral membrane (neutral sphingomyelinase II) | SMPD3 | -0,04 | 0,03 | -0,05 | 0,64 |
| 16817790 | aldolase A, fructose-bisphosphate | ALDOA | -0,04 | 0,01 | 0,05 | 0,63 |
| 16839388 | inositol polyphosphate-5-phosphatase K | INPP5K | -0,03 | 0,04 | 0,04 | 0,58 |

^1^From Student’s t-test.

**Table D. Pathways significantly downregulated in response to S6K1 siRNA, but not to S6K2 siRNA.**

| p-value | Term | Term ID | Term description | Genes |
| --- | --- | --- | --- | --- |
| 3.14e-08 | BIOGRID:00000 | bi | BioGRID interaction data | ANXA9, GPRC5A, KRT13, FOS, KRT4, FTL, DHRS3, NPNT, CRISP3, FOSB, ATF3, SLC31A2, COL12A1, PAPSS2, PMP22, FREM2, ZNF358, MESDC1, STX3, LITAF, NQO1, SLC7A11, DHCR24, S100A9, ABCC3, RGS17, DENND2D, FBXL22, TP53TG5, CENPQ, KCTD12, NR4A1, PDLIM1, OSTM1, ARHGAP31, PDGFD |
| 1.63e-02 | GO:0044707 | BP | single-multicellular organism process | CLEC3A, KRT13, FOS, KRT4, DHRS3, NPNT, FOSB, ATF3, COL12A1, PAPSS2, PMP22, FREM2, ZNF358, STX3, LITAF, NQO1, SLC7A11, DHCR24, S100A9, NR4A1, OSTM1, PDGFD, NTSR1, RAB14, PTGDS, SLC18A2, UTS2R, TSPEAR, ELF3, ANGPT1, PLK2, ERAP1, WWTR1, TAGLN3, PCSK4, NFAM1, DHRS2, NGRN, TCF7L2, DOCK8, LCE1A, TMPRSS6, ITPKA, WWOX, EIF4EBP1, CCK, EYA4, DGKB, ADAP2, GRM6, YPEL1, TRPC1, CIITA, SLC1A6, ADAM21, SELPLG, CDK6, SULF1, FBLN1, CATSPER1, AGR2, NLRP14, FEV, HMX1, NYAP2, HMOX1, RIPPLY1, FOXF2, SLC38A1, LEPREL1, LRP5L, ETV7, TFPI2, NR0B1, RAB8B, USH2A, HUNK, PCDH18, NEUROD4, SCN8A, MYO15A, PAX9, SIX2, DNAAF1, GGN, CLIC5, FUT7, ARX, REG3G, MYOF, NINJ2, MT3, TFIP11, TMEM119, CYP24A1, AGRP, SLC22A3, NR2F2, FABP1, SLITRK6, CLPTM1, IMPG1, NEK8, IL5, PFN1, ITGA2B, SIX5, HTR1E, HEY1, NFE2L1, KCNQ4, MOGAT2, CNTN3, VAMP2, PTPRB, TMC1, GABRA1, STOX2, OVOL2, SMPD3, ALDOA, INPP5K |
| 2.72e-02 | GO:0044699 | BP | single-organism process | ANXA9, CLEC3A, GPRC5A, KRT13, FOS, KRT4, FTL, DHRS3, NPNT, FOSB, ATF3, SLC31A2, COL12A1, PAPSS2, PMP22, FREM2, ZNF358, STX3, LITAF, NQO1, SLC7A11, DHCR24, S100A9, ABCC3, CYP2F1, RGS17, TP53TG5, CENPQ, NR4A1, OSTM1, ARHGAP31, PDGFD, NTSR1, RAB14, PTGDS, SMAD5-AS1, NME9, SLC18A2, RERG, UTS2R, TSPEAR, ELF3, ANGPT1, SLC25A24, PLK2, IL10RA, ATP8B1, ERAP1, WWTR1, TOMM20L, GLP1R, TAGLN3, RGSL1, PCSK4, NFAM1, PNMA3, DHRS2, NGRN, TCF7L2, KCNE3, TFDP3, OAS1, DOCK8, APOBEC3C, LCE1A, TMPRSS6, ITPKA, PTTG1IP, WWOX, EIF4EBP1, SLC20A2, JDP2, CCK, ALDH1A1, EYA4, SLC4A9, DGKB, SYNPO, CEACAM6, ADAP2, HAPLN4, TPD52L1, GRM6, ADIPOR2, YPEL1, TRPC1, CXCL16, CIITA, VAMP7, SLC1A6, ADAM21, RGS4, SELPLG, CDK6, SULF1, TMEM109, FBLN1, CATSPER1, AGR2, NLRP14, FEV, ACOT11, RBPMS, HMX1, NYAP2, PSME1, KIF18B, ZSWIM2, HIST1H1E, HMOX1, RIPPLY1, FOXF2, PVRL2, GOLM1, LY86, GOLPH3, SLC38A1, LEPREL1, LRP5L, SAP130, WWC1, GPX3, ETV7, ABCG2, TFPI2, RAB11B, NR0B1, RAB8B, USH2A, NGB, HUNK, PCDH18, SIGLEC11, NEUROD4, CR1, SCN8A, INPP4A, DDHD2, UVSSA, MYO15A, KPNA3, PAX9, TBC1D1, SIX2, DNAAF1, CA12, MBL2, GGN, WBP2NL, CLIC5, RETNLB, FUT7, UBE2Z, TRPM6, ARX, REG3G, MYOF, NINJ2, MT3, SPCS1, TFIP11, KLHDC5, TMEM119, CYP24A1, AGRP, ZDHHC16, SLC22A3, NR2F2, FABP1, SLITRK6, CLPTM1, IMPG1, KRT86, IFT140, NEK8, IL5, ATP6V1C2, PFN1, ITGA2B, SIX5, HTR1E, CLDN1, SCG5, HEY1, NFE2L1, KCNQ4, MOGAT2, CNTN3, VAMP2, KIF7, IQGAP2, HTRA4, EIF4G2, PTPRB, TOMM40L, TMC1, GGA2, GABRA1, FANCE, STOX2, OVOL2, HIST1H2BK, SMPD3, ALDOA, INPP5K |
