# Supplementary material for: Revealing Different Roles of the mTOR-Targets S6K1 and S6K2 in Breast Cancer by Expression Profiling and Structural Analysis
Source: PLoS One. 2015 Dec 23;10(12):e0145013. doi: 10.1371/journal.pone.0145013 (PMC4689523; doi:10.1371/journal.pone.0145013)
Supplement: S11 Table — Genes upregulated in response to S6K2 siRNA, but not to S6K1 siRNA (Table A). Pathways upregulated in response to S6K2 siRNA, but not to S6K1 siRNA (Table B). Genes upregulated in response to S6K2 siRNA, but not to S6K1 siRNA (Table C). Pathways downregulated in response to S6K2 siRNA, but not to S6K1 siRNA (Table D). (DOCX) [file pone.0145013.s015.docx]

**Table A. Genes upregulated in response to S6K2 siRNA, but not to S6K1 siRNA.**

| Transcripts Cluster ID | Gene description | Gene symbol | S6K2 siRNA  Fold change | S6K2 siRNA  p-value^1^ | S6K1 siRNA  Fold change | S6K1 siRNA  p-value^1^ |
| --- | --- | --- | --- | --- | --- | --- |
| 16701398 | SMYD3 intronic transcript 1 (non-protein coding) | SMYD3-IT1 | 0,68 | 0,0068 | 0,65 | 0,1610 |
| 16826552 | RPGRIP1-like | RPGRIP1L | 0,64 | 0,0245 | 0,43 | 0,2748 |
| 16905521 | tetratricopeptide repeat domain 30A | TTC30A | 0,63 | 0,0420 | 0,59 | 0,0534 |
| 16823825 | epithelial membrane protein 2 | EMP2 | 0,62 | 0,0150 | 0,43 | 0,0754 |
| 16808876 | centrosomal protein 152kDa | CEP152 | 0,61 | 0,0441 | 0,43 | 0,1896 |
| 17044692 | proline rich 15 | PRR15 | 0,59 | 0,0225 | 0,30 | 0,2158 |
| 16920180 | spermatogenesis associated 2 | SPATA2 | 0,58 | 0,0158 | 0,49 | 0,1839 |
| 17084428 | ubiquitin associated protein 1 | UBAP1 | 0,55 | 0,0123 | 0,28 | 0,1288 |
| 16820676 | pyruvate dehydrogenase phosphatase regulatory subunit | PDPR | 0,54 | 0,0396 | 0,44 | 0,0609 |
| 16819264 | metallothionein 1X | MT1X | 0,53 | 0,0437 | 0,46 | 0,1219 |
| 16774271 | N(alpha)-acetyltransferase 16, NatA auxiliary subunit | NAA16 | 0,53 | 0,0135 | 0,45 | 0,0719 |
| 16673525 | ATPase, Na+/K+ transporting, beta 1 polypeptide | ATP1B1 | 0,47 | 0,0368 | 0,27 | 0,0761 |
| 16812717 | BTB (POZ) domain containing 1 | BTBD1 | 0,47 | 0,0086 | 0,12 | 0,3449 |
| 16862548 | carcinoembryonic antigen-related cell adhesion molecule 5 | CEACAM5 | 0,47 | 0,0068 | 0,32 | 0,1482 |
| 17014963 | exocyst complex component 2 | EXOC2 | 0,47 | 0,0051 | 0,35 | 0,1452 |
| 16996298 | interleukin 6 signal transducer (gp130, oncostatin M receptor) | IL6ST | 0,47 | 0,0023 | 0,26 | 0,0500 |
| 16866031 | zinc finger protein 17 | ZNF17 | 0,47 | 0,0198 | 0,33 | 0,2548 |
| 16711881 | BEN domain containing 7 | BEND7 | 0,46 | 0,0063 | 0,50 | 0,0917 |
| 16882945 | fumarylacetoacetate hydrolase domain containing 2C, pseudogene | FAHD2CP | 0,46 | 0,0368 | 0,15 | 0,3485 |
| 16831381 | Meis homeobox 3 pseudogene 1 | MEIS3P1 | 0,46 | 0,0068 | 0,07 | 0,8143 |
| 16799577 | carbohydrate (N-acetylgalactosamine 4-0) sulfotransferase 14 | CHST14 | 0,45 | 0,0285 | 0,33 | 0,2494 |
| 16810473 | death-associated protein kinase 2 | DAPK2 | 0,45 | 0,0147 | 0,21 | 0,4261 |
| 16868576 | DNA (cytosine-5-)-methyltransferase 1 | DNMT1 | 0,45 | 0,0386 | 0,34 | 0,1200 |
| 16852241 | katanin p60 subunit A-like 2 | KATNAL2 | 0,45 | 0,0405 | 0,35 | 0,1038 |
| 16707030 | phosphatase and tensin homolog | PTEN | 0,45 | 0,0428 | 0,40 | 0,2078 |
| 16826004 | PYD (pyrin domain) containing 1 | PYDC1 | 0,45 | 0,0007 | 0,30 | 0,0941 |
| 16781136 | tubulin, gamma complex associated protein 3 | TUBGCP3 | 0,45 | 0,0011 | 0,33 | 0,0927 |
| 16855184 | acetyl-CoA acyltransferase 2 | ACAA2 | 0,44 | 0,0299 | 0,12 | 0,1002 |
| 17004879 | human immunodeficiency virus type I enhancer binding protein 1 | HIVEP1 | 0,44 | 0,0217 | 0,32 | 0,2561 |
| 16778460 | kelch repeat and BTB (POZ) domain containing 7 | KBTBD7 | 0,44 | 0,0203 | 0,50 | 0,1010 |
| 16996387 | mesoderm induction early response 1, family member 3 | MIER3 | 0,44 | 0,0443 | 0,45 | 0,0875 |
| 16807976 | tau tubulin kinase 2 | TTBK2 | 0,44 | 0,0168 | 0,24 | 0,1451 |
| 16764305 | tubulin, alpha 1a | TUBA1A | 0,44 | 0,0224 | 0,15 | 0,3856 |
| 16665447 | ubiquitin specific peptidase 1 | USP1 | 0,44 | 0,0106 | 0,28 | 0,1207 |
| 16747568 | destrin (actin depolymerizing factor) pseudogene 2 | DSTNP2 | 0,43 | 0,0239 | 0,23 | 0,3795 |
| 16670081 | chromodomain helicase DNA binding protein 1-like | CHD1L | 0,42 | 0,0151 | 0,16 | 0,2800 |
| 17022097 | HECT domain and ankyrin repeat containing E3 ubiquitin protein ligase 1 | HACE1 | 0,42 | 0,0042 | 0,16 | 0,2530 |
| 17092852 | kelch-like 9 (Drosophila) | KLHL9 | 0,42 | 0,0325 | 0,21 | 0,3031 |
| 17100420 | NOTCH-regulated ankyrin repeat protein | NRARP | 0,42 | 0,0380 | 0,27 | 0,3199 |
| 16868481 | olfactomedin 2 | OLFM2 | 0,42 | 0,0058 | 0,28 | 0,2460 |
| 16870821 | zinc finger protein 100 | ZNF100 | 0,42 | 0,0078 | 0,28 | 0,0720 |
| 17095948 | zinc finger protein 484 | ZNF484 | 0,42 | 0,0118 | 0,38 | 0,1913 |
| 16803710 | aryl-hydrocarbon receptor nuclear translocator 2 | ARNT2 | 0,41 | 0,0497 | 0,11 | 0,3562 |
| 16832852 | ATPase family, AAA domain containing 5 | ATAD5 | 0,41 | 0,0025 | 0,20 | 0,1146 |
| 16815971 | excision repair cross-complementing rodent repair deficiency, complementation group 4 | ERCC4 | 0,41 | 0,0155 | 0,30 | 0,1940 |
| 16807613 | NADH dehydrogenase (ubiquinone) complex I, assembly factor 1 | NDUFAF1 | 0,41 | 0,0266 | 0,32 | 0,1777 |
| 17005515 | tripartite motif containing 38 | TRIM38 | 0,41 | 0,0275 | 0,19 | 0,1866 |
| 17005862 | histone cluster 1, H3h \| histone cluster 1, H3f \| histone cluster 1, H3b \| histone cluster 1, H3j \| histone cluster 1, H3g \| histone cluster 1, H3i \| histone cluster 1, H3e \| histone cluster 1, H3c \| histone cluster 1, H3d \| histone cluster 1, H3a | HIST1H3H\| HIST1H3F\| HIST1H3B\| HIST1H3J\| HIST1H3G\| HIST1H3I\| HIST1H3E\| HIST1H3C\| HIST1H3D\| HIST1H3A | 0,4 | 0,0418 | 0,14 | 0,6206 |
| 16927718 | BMS1 homolog, ribosome assembly protein (yeast) pseudogene | LOC96610 | 0,4 | 0,0416 | 0,34 | 0,2875 |
| 16681775 | methylenetetrahydrofolate reductase (NAD(P)H) | MTHFR | 0,4 | 0,0234 | 0,19 | 0,2931 |
| 16990267 | protocadherin beta 5 | PCDHB5 | 0,4 | 0,0068 | 0,17 | 0,4205 |
| 16833616 | suppressor of cytokine signaling 7 | SOCS7 | 0,4 | 0,0277 | 0,20 | 0,0746 |
| 16948366 | tetratricopeptide repeat domain 14 | TTC14 | 0,4 | 0,0396 | 0,49 | 0,0861 |
| 16866002 | zinc finger protein 543 | ZNF543 | 0,4 | 0,0002 | 0,24 | 0,3178 |
| 16870956 | zinc finger protein 91 | ZNF91 | 0,4 | 0,0054 | 0,21 | 0,0667 |
| 17016383 | histone cluster 1, H4d \| histone cluster 2, H4b \| histone cluster 4, H4 \| histone cluster 2, H4a \| histone cluster 1, H4l \| histone cluster 1, H4e \| histone cluster 1, H4b \| histone cluster 1, H4h \| histone cluster 1, H4c \| histone cluster 1, H4j \| histone cluster 1, H4k \| histone cluster 1, H4f \| histone cluster 1, H4a \| histone cluster 1, H4i | HIST1H4D\| HIST2H4B\| HIST4H4\| HIST2H4A\| HIST1H4L\| HIST1H4E\| HIST1H4B\| HIST1H4H\| HIST1H4C\| HIST1H4J\| HIST1H4K\| HIST1H4F\| HIST1H4A\| HIST1H4I | 0,39 | 0,0345 | 0,20 | 0,1528 |
| 16922243 | interleukin 10 receptor, beta | IL10RB | 0,39 | 0,0216 | 0,35 | 0,1247 |
| 17113110 | RNA binding motif protein 41 | RBM41 | 0,39 | 0,0091 | 0,23 | 0,1415 |
| 16955048 |  | RFT1 | 0,39 | 0,0083 | 0,22 | 0,1618 |
| 16824463 | ribosomal protein S15a | RPS15A | 0,39 | 0,0026 | 0,38 | 0,1036 |
| 17038817 | tripartite motif containing 27 | TRIM27 | 0,39 | 0,0222 | 0,22 | 0,3235 |
| 16995241 | UDP glycosyltransferase 3 family, polypeptide A2 | UGT3A2 | 0,39 | 0,0304 | 0,13 | 0,5812 |
| 16709964 | WD repeat domain 11 \| uncharacterized LOC100506158 | WDR11\| LOC100506158 | 0,39 | 0,0205 | 0,30 | 0,2400 |
| 16786780 | chromosome 14 open reading frame 118 | C14orf118 | 0,38 | 0,0183 | 0,19 | 0,1809 |
| 16826212 | chromosome 16 open reading frame 87 | C16orf87 | 0,38 | 0,0205 | 0,23 | 0,1458 |
| 16950196 | CHL1 antisense RNA 1 (non-protein coding) | CHL1-AS1 | 0,38 | 0,0201 | 0,17 | 0,2416 |
| 16950912 | IQ motif and Sec7 domain 1 | IQSEC1 | 0,38 | 0,0448 | 0,34 | 0,1415 |
| 16808310 | diphosphoinositol pentakisphosphate kinase 1 | PPIP5K1 | 0,38 | 0,0150 | 0,16 | 0,2653 |
| 16825561 | seizure related 6 homolog (mouse)-like 2 | SEZ6L2 | 0,38 | 0,0196 | 0,21 | 0,2404 |
| 16916920 | solute carrier family 23 (nucleobase transporters), member 2 | SLC23A2 | 0,38 | 0,0106 | 0,34 | 0,0975 |
| 16879791 | suppressor of cytokine signaling 5 | SOCS5 | 0,38 | 0,0326 | 0,19 | 0,0996 |
| 16898728 |  | TIA1 | 0,38 | 0,0117 | 0,32 | 0,0914 |
| 17026472 | tripartite motif containing 27 | TRIM27 | 0,38 | 0,0223 | 0,22 | 0,3228 |
| 17016624 | tripartite motif containing 27 | TRIM27 | 0,38 | 0,0213 | 0,22 | 0,3229 |
| 17041432 | tripartite motif containing 27 | TRIM27 | 0,38 | 0,0279 | 0,17 | 0,4038 |
| 16862324 | inositol-trisphosphate 3-kinase C | ITPKC | 0,37 | 0,0434 | 0,16 | 0,0596 |
| 17076694 | K(lysine) acetyltransferase 6A | KAT6A | 0,37 | 0,0053 | 0,29 | 0,0701 |
| 16844929 | kelch-like 11 (Drosophila) | KLHL11 | 0,37 | 0,0289 | 0,19 | 0,2958 |
| 16971573 | meiotic nuclear divisions 1 homolog (S. cerevisiae) | MND1 | 0,37 | 0,0248 | 0,12 | 0,5137 |
| 16675558 | NIMA (never in mitosis gene a)-related kinase 7 | NEK7 | 0,37 | 0,0119 | 0,35 | 0,0701 |
| 16992989 | nuclear receptor binding SET domain protein 1 | NSD1 | 0,37 | 0,0281 | 0,32 | 0,0998 |
| 16730180 | pannexin 1 | PANX1 | 0,37 | 0,0011 | 0,29 | 0,1236 |
| 16814988 |  | RAB26 | 0,37 | 0,0012 | 0,31 | 0,1406 |
| 16817973 | Snf2-related CREBBP activator protein \| uncharacterized LOC100862671 | SRCAP\| LOC100862671 | 0,37 | 0,0411 | 0,19 | 0,2292 |
| 17025070 | transcription factor B1, mitochondrial | TFB1M | 0,37 | 0,0007 | 0,22 | 0,4154 |
| 16850477 | thymidylate synthetase \| enolase superfamily member 1 | TYMS\| ENOSF1 | 0,37 | 0,0027 | 0,16 | 0,0602 |
| 16908373 | ubiquitin specific peptidase 37 | USP37 | 0,37 | 0,0162 | 0,16 | 0,2079 |
| 16668288 | adenosine monophosphate deaminase 2 | AMPD2 | 0,36 | 0,0106 | 0,25 | 0,1201 |
| 16932804 | RIMS binding protein 3C | RIMBP3C | 0,36 | 0,0147 | 0,29 | 0,2886 |
| 16898292 | vacuolar protein sorting 54 homolog (S. cerevisiae) | VPS54 | 0,36 | 0,0410 | 0,23 | 0,2560 |
| 16865845 | zinc finger protein 444 | ZNF444 | 0,36 | 0,0070 | 0,33 | 0,2200 |
| 17010147 | chromosome 6 open reading frame 57 | C6orf57 | 0,35 | 0,0445 | 0,30 | 0,0765 |
| 16833750 | cyclin-dependent kinase 12 | CDK12 | 0,35 | 0,0202 | 0,21 | 0,3168 |
| 16712458 | DnaJ (Hsp40) homolog, subfamily C, member 1 | DNAJC1 | 0,35 | 0,0076 | 0,12 | 0,0671 |
| 16859711 | microtubule associated serine/threonine kinase 3 | MAST3 | 0,35 | 0,0261 | 0,15 | 0,2815 |
| 16867074 | major facilitator superfamily domain containing 12 | MFSD12 | 0,35 | 0,0189 | 0,41 | 0,1458 |
| 16830988 | nudE nuclear distribution E homolog (A. nidulans)-like 1 | NDEL1 | 0,35 | 0,0190 | 0,26 | 0,1039 |
| 16723318 | proline rich Gla (G-carboxyglutamic acid) 4 (transmembrane) | PRRG4 | 0,35 | 0,0058 | 0,13 | 0,2387 |
| 16763853 | SUMO1/sentrin specific peptidase 1 | SENP1 | 0,35 | 0,0001 | 0,14 | 0,3863 |
| 16944055 | SID1 transmembrane family, member 1 | SIDT1 | 0,35 | 0,0375 | -0,09 | 0,3142 |
| 16970409 | spermatogenesis associated 5 | SPATA5 | 0,35 | 0,0396 | 0,23 | 0,0598 |
| 17061433 | SRSF protein kinase 2 | SRPK2 | 0,35 | 0,0405 | 0,12 | 0,4430 |
| 16829629 | serine racemase | SRR | 0,35 | 0,0433 | -0,03 | 0,8100 |
| 16865992 | zinc finger protein 460 | ZNF460 | 0,35 | 0,0195 | 0,24 | 0,2141 |
| 16881514 | Alstrom syndrome 1 | ALMS1 | 0,34 | 0,0155 | 0,22 | 0,0675 |
| 16911394 | ankyrin repeat domain 5 | ANKRD5 | 0,34 | 0,0131 | 0,19 | 0,1607 |
| 16860644 | CCAAT/enhancer binding protein (C/EBP), gamma | CEBPG | 0,34 | 0,0436 | 0,25 | 0,0655 |
| 16974713 | DDB1 and CUL4 associated factor 16 | DCAF16 | 0,34 | 0,0367 | 0,22 | 0,4137 |
| 16844967 | DnaJ (Hsp40) homolog, subfamily C, member 7 | DNAJC7 | 0,34 | 0,0105 | 0,18 | 0,1695 |
| 16683445 | fucosidase, alpha-L- 1, tissue | FUCA1 | 0,34 | 0,0390 | 0,22 | 0,2661 |
| 16833454 | gametogenetin binding protein 2 | GGNBP2 | 0,34 | 0,0117 | 0,27 | 0,0649 |
| 16912057 | GDNF-inducible zinc finger protein 1 | GZF1 | 0,34 | 0,0178 | 0,08 | 0,4633 |
| 16705356 | heterogeneous nuclear ribonucleoprotein H3 (2H9) | HNRNPH3 | 0,34 | 0,0087 | 0,25 | 0,0996 |
| 16972764 | inhibitor of growth family, member 2 | ING2 | 0,34 | 0,0210 | 0,15 | 0,4826 |
| 16753588 | LEM domain containing 3 | LEMD3 | 0,34 | 0,0095 | 0,29 | 0,3755 |
| 16819082 | lysophosphatidylcholine acyltransferase 2 | LPCAT2 | 0,34 | 0,0124 | -0,02 | 0,9100 |
| 17080022 | low density lipoprotein receptor-related protein 12 | LRP12 | 0,34 | 0,0217 | 0,29 | 0,1892 |
| 16816833 | polymerase (RNA) III (DNA directed) polypeptide E (80kD) | POLR3E | 0,34 | 0,0226 | 0,24 | 0,1253 |
| 16869570 | protein kinase, cAMP-dependent, catalytic, alpha | PRKACA | 0,34 | 0,0415 | 0,04 | 0,8312 |
| 16795878 | SMEK homolog 1, suppressor of mek1 (Dictyostelium) | SMEK1 | 0,34 | 0,0045 | 0,24 | 0,1024 |
| 16935593 | transcription factor 20 (AR1) | TCF20 | 0,34 | 0,0494 | 0,11 | 0,1071 |
| 16860418 | cyclin E1 | CCNE1 | 0,33 | 0,0113 | 0,24 | 0,1031 |
| 16737151 | cleavage stimulation factor, 3' pre-RNA, subunit 3, 77kDa | CSTF3 | 0,33 | 0,0466 | 0,29 | 0,0958 |
| 17104983 | chromosome X open reading frame 26 | CXorf26 | 0,33 | 0,0322 | 0,33 | 0,1883 |
| 16771632 | diablo, IAP-binding mitochondrial protein | DIABLO | 0,33 | 0,0368 | 0,09 | 0,6529 |
| 16832067 | EPN2 intronic transcript 1 (non-protein coding) | EPN2-IT1 | 0,33 | 0,0399 | 0,37 | 0,0669 |
| 16984992 | GC-rich promoter binding protein 1 | GPBP1 | 0,33 | 0,0281 | 0,26 | 0,1097 |
| 16744125 | KDEL (Lys-Asp-Glu-Leu) containing 2 | KDELC2 | 0,33 | 0,0005 | 0,14 | 0,0741 |
| 16681210 | kelch-like 21 (Drosophila) | KLHL21 | 0,33 | 0,0444 | 0,15 | 0,4492 |
| 17114728 | melanoma antigen family C, 2 | MAGEC2 | 0,33 | 0,0386 | -0,10 | 0,7249 |
| 17008412 | nuclear transcription factor Y, alpha | NFYA | 0,33 | 0,0071 | 0,07 | 0,5169 |
| 16825104 | partner and localizer of BRCA2 | PALB2 | 0,33 | 0,0180 | 0,22 | 0,0545 |
| 16910341 | PAS domain containing serine/threonine kinase | PASK | 0,33 | 0,0378 | 0,22 | 0,1369 |
| 16998727 | praja ring finger 2, E3 ubiquitin protein ligase | PJA2 | 0,33 | 0,0365 | 0,33 | 0,1684 |
| 16749105 | pyridine nucleotide-disulphide oxidoreductase domain 1 | PYROXD1 | 0,33 | 0,0220 | 0,23 | 0,2221 |
| 16809596 |  | RAB27A | 0,33 | 0,0207 | 0,10 | 0,3203 |
| 16947113 |  | RAP2B | 0,33 | 0,0455 | 0,20 | 0,2013 |
| 17102129 | spermidine/spermine N1-acetyltransferase 1 | SAT1 | 0,33 | 0,0197 | -0,15 | 0,0648 |
| 16775546 | sciellin | SCEL | 0,33 | 0,0068 | 0,05 | 0,7558 |
| 16778745 | solute carrier family 25, member 30 | SLC25A30 | 0,33 | 0,0421 | 0,25 | 0,2762 |
| 16799231 | sprouty-related, EVH1 domain containing 1 | SPRED1 | 0,33 | 0,0464 | 0,13 | 0,3058 |
| 17048754 | transformation/transcription domain-associated protein | TRRAP | 0,33 | 0,0326 | 0,16 | 0,2592 |
| 16763021 | tyrosyl-tRNA synthetase 2, mitochondrial | YARS2 | 0,33 | 0,0223 | 0,21 | 0,1413 |
| 16979638 | ankyrin repeat domain 50 | ANKRD50 | 0,32 | 0,0316 | 0,17 | 0,2046 |
| 16689289 | cysteine conjugate-beta lyase 2 \| RNA binding motif protein, X-linked-like 1 | CCBL2\| RBMXL1 | 0,32 | 0,0424 | 0,30 | 0,0856 |
| 16909081 | dedicator of cytokinesis 10 | DOCK10 | 0,32 | 0,0405 | 0,21 | 0,0714 |
| 16896442 | eukaryotic translation initiation factor 2-alpha kinase 2 | EIF2AK2 | 0,32 | 0,0349 | 0,11 | 0,3127 |
| 16997199 | ectodermal-neural cortex 1 (with BTB-like domain) | ENC1 | 0,32 | 0,0128 | -0,07 | 0,6999 |
| 16998700 | F-box and leucine-rich repeat protein 17 | FBXL17 | 0,32 | 0,0483 | 0,15 | 0,1576 |
| 16673617 | golgin, RAB6-interacting | GORAB | 0,32 | 0,0341 | 0,36 | 0,0776 |
| 16963542 | succinate dehydrogenase complex, subunit A, flavoprotein (Fp) pseudogene | LOC220729 | 0,32 | 0,0149 | 0,23 | 0,1431 |
| 16928867 | myotubularin related protein 3 | MTMR3 | 0,32 | 0,0157 | 0,30 | 0,0686 |
| 16880433 | peroxisomal biogenesis factor 13 | PEX13 | 0,32 | 0,0254 | 0,25 | 0,1369 |
| 17111219 | structural maintenance of chromosomes 1A | SMC1A | 0,32 | 0,0128 | 0,15 | 0,2385 |
| 16843049 | slingshot homolog 2 (Drosophila) | SSH2 | 0,32 | 0,0322 | 0,34 | 0,0588 |
| 17099083 | torsin family 1, member A (torsin A) | TOR1A | 0,32 | 0,0082 | 0,22 | 0,0973 |
| 17028529 | tripartite motif containing 27 | TRIM27 | 0,32 | 0,0160 | 0,17 | 0,4026 |
| 16907087 | tRNA-yW synthesizing protein 5 | TYW5 | 0,32 | 0,0280 | 0,29 | 0,2500 |
| 16954013 | ubiquitin specific peptidase 4 (proto-oncogene) | USP4 | 0,32 | 0,0345 | 0,32 | 0,0833 |
| 16872313 | zinc finger protein 780B | ZNF780B | 0,32 | 0,0090 | 0,22 | 0,1122 |
| 16886308 | activin A receptor, type IIA | ACVR2A | 0,31 | 0,0073 | 0,17 | 0,0754 |
| 16746057 | Rho GTPase activating protein 32 | ARHGAP32 | 0,31 | 0,0116 | 0,17 | 0,1049 |
| 16675174 | chromosome 1 open reading frame 27 | C1orf27 | 0,31 | 0,0099 | 0,32 | 0,0591 |
| 17097955 | complement component 5 | C5 | 0,31 | 0,0315 | 0,03 | 0,6358 |
| 16763970 | cyclin T1 | CCNT1 | 0,31 | 0,0219 | 0,09 | 0,3023 |
| 16869922 | cytochrome P450, family 4, subfamily F, polypeptide 24, pseudogene | CYP4F24P | 0,31 | 0,0183 | 0,04 | 0,5334 |
| 16810792 | dipeptidyl-peptidase 8 | DPP8 | 0,31 | 0,0119 | 0,20 | 0,1312 |
| 17005234 | E2F transcription factor 3 | E2F3 | 0,31 | 0,0063 | 0,04 | 0,7664 |
| 16767851 | E2F transcription factor 7 | E2F7 | 0,31 | 0,0223 | 0,16 | 0,3096 |
| 16870417 | elongation factor RNA polymerase II | ELL | 0,31 | 0,0168 | 0,35 | 0,0863 |
| 17102861 | lysine (K)-specific demethylase 6A | KDM6A | 0,31 | 0,0388 | 0,10 | 0,1770 |
| 16707468 | kinesin family member 11 | KIF11 | 0,31 | 0,0235 | 0,18 | 0,0790 |
| 16910222 | kinesin family member 1A | KIF1A | 0,31 | 0,0420 | 0,18 | 0,2388 |
| 16933760 | leukemia inhibitory factor | LIF | 0,31 | 0,0105 | 0,21 | 0,2505 |
| 16824928 | nuclear pore complex-interacting protein-like 3-like \| nuclear pore complex interacting protein-like 3 | LOC728888\| NPIPL3 | 0,31 | 0,0185 | 0,17 | 0,1204 |
| 17097132 | lysophosphatidic acid receptor 1 | LPAR1 | 0,31 | 0,0207 | 0,23 | 0,1308 |
| 16965338 | mediator complex subunit 28 | MED28 | 0,31 | 0,0324 | 0,04 | 0,8106 |
| 16854316 | oxysterol binding protein-like 1A | OSBPL1A | 0,31 | 0,0284 | 0,24 | 0,1067 |
| 17102963 | PHD finger protein 16 | PHF16 | 0,31 | 0,0019 | 0,08 | 0,3754 |
| 16887289 | peptidylprolyl isomerase G (cyclophilin G) | PPIG | 0,31 | 0,0041 | 0,22 | 0,1418 |
| 16873581 | protein kinase D2 | PRKD2 | 0,31 | 0,0497 | 0,17 | 0,0799 |
| 17047652 | protein tyrosine phosphatase, non-receptor type 12 | PTPN12 | 0,31 | 0,0258 | -0,04 | 0,1642 |
| 16702147 | RNA binding motif protein 17 | RBM17 | 0,31 | 0,0088 | 0,23 | 0,1549 |
| 16829580 | replication protein A1, 70kDa | RPA1 | 0,31 | 0,0219 | 0,19 | 0,1636 |
| 16913493 | regulation of nuclear pre-mRNA domain containing 1B | RPRD1B | 0,31 | 0,0045 | 0,23 | 0,1430 |
| 16906000 | SEC14 and spectrin domains 1 | SESTD1 | 0,31 | 0,0212 | 0,19 | 0,2794 |
| 16848070 | solute carrier family 16, member 6 (monocarboxylic acid transporter 7) | SLC16A6 | 0,31 | 0,0486 | 0,04 | 0,7726 |
| 16898403 | sprouty-related, EVH1 domain containing 2 | SPRED2 | 0,31 | 0,0487 | 0,22 | 0,2315 |
| 16793644 | transmembrane protein 30B | TMEM30B | 0,31 | 0,0132 | 0,16 | 0,3793 |
| 16766348 | zinc finger and BTB domain containing 39 | ZBTB39 | 0,31 | 0,0109 | 0,16 | 0,1050 |
| 17099769 | calmodulin regulated spectrin-associated protein 1 | CAMSAP1 | 0,3 | 0,0088 | 0,27 | 0,1160 |
| 16706128 | coiled-coil-helix-coiled-coil-helix domain containing 1 | CHCHD1 | 0,3 | 0,0400 | 0,24 | 0,1722 |
| 16884877 | DEAD (Asp-Glu-Ala-Asp) box polypeptide 18 | DDX18 | 0,3 | 0,0398 | 0,00 | 0,9869 |
| 16855781 | dermatan sulfate epimerase-like | DSEL | 0,3 | 0,0234 | 0,18 | 0,3274 |
| 16778921 | esterase D | ESD | 0,3 | 0,0383 | 0,40 | 0,1145 |
| 16960567 | G protein-coupled receptor 87 | GPR87 | 0,3 | 0,0335 | 0,11 | 0,5930 |
| 16966459 |  | GUF1 | 0,3 | 0,0068 | 0,27 | 0,2031 |
| 16985688 | MARVEL domain containing 2 | MARVELD2 | 0,3 | 0,0050 | 0,09 | 0,5112 |
| 16962754 | Mab-21 domain containing 2 | MB21D2 | 0,3 | 0,0361 | 0,20 | 0,5338 |
| 16928967 | mitochondrial fission process 1 | MTFP1 | 0,3 | 0,0058 | 0,24 | 0,1652 |
| 16824132 | N-terminal asparagine amidase | NTAN1 | 0,3 | 0,0248 | 0,25 | 0,2896 |
| 16960705 | phospholipase C, eta 1 | PLCH1 | 0,3 | 0,0205 | 0,19 | 0,1977 |
| 16999245 | peptidylprolyl isomerase C (cyclophilin C) | PPIC | 0,3 | 0,0397 | 0,28 | 0,0613 |
| 17099076 | prostaglandin E synthase | PTGES | 0,3 | 0,0405 | 0,11 | 0,4155 |
| 16779995 | ring finger protein 219 | RNF219 | 0,3 | 0,0053 | 0,20 | 0,0587 |
| 16855820 | rotatin | RTTN | 0,3 | 0,0155 | 0,27 | 0,0643 |
| 16809929 | SAFB-like, transcription modulator | SLTM | 0,3 | 0,0350 | 0,14 | 0,1647 |
| 16896710 | son of sevenless homolog 1 (Drosophila) | SOS1 | 0,3 | 0,0156 | 0,17 | 0,3996 |
| 17096471 | TBC1 domain family, member 2 | TBC1D2 | 0,3 | 0,0135 | 0,03 | 0,5222 |
| 17106640 | zinc finger and BTB domain containing 33 | ZBTB33 | 0,3 | 0,0423 | 0,23 | 0,1452 |
| 16831789 | alkB, alkylation repair homolog 5 (E. coli) \| uncharacterized LOC100653189 \| uncharacterized LOC100652858 | ALKBH5\| LOC100653189\| LOC100652858 | 0,29 | 0,0251 | 0,10 | 0,0500 |
| 16699138 | angel homolog 2 (Drosophila) | ANGEL2 | 0,29 | 0,0244 | 0,18 | 0,0909 |
| 16982011 | caspase 3, apoptosis-related cysteine peptidase | CASP3 | 0,29 | 0,0005 | 0,20 | 0,0898 |
| 16936097 | cadherin, EGF LAG seven-pass G-type receptor 1 (flamingo homolog, Drosophila) | CELSR1 | 0,29 | 0,0052 | 0,12 | 0,3447 |
| 16830782 | cytochrome b5 domain containing 1 | CYB5D1 | 0,29 | 0,0459 | 0,23 | 0,0854 |
| 16719393 | DEAH (Asp-Glu-Ala-His) box polypeptide 32 | DHX32 | 0,29 | 0,0481 | 0,25 | 0,1330 |
| 16677324 | feline leukemia virus subgroup C cellular receptor 1 | FLVCR1 | 0,29 | 0,0181 | 0,18 | 0,2979 |
| 16795351 | general transcription factor IIA, 1, 19/37kDa | GTF2A1 | 0,29 | 0,0458 | 0,28 | 0,0946 |
| 16662077 | histone deacetylase 1 | HDAC1 | 0,29 | 0,0432 | 0,13 | 0,3341 |
| 16681082 | isoprenylcysteine carboxyl methyltransferase | ICMT | 0,29 | 0,0110 | 0,23 | 0,0925 |
| 16678851 | mixed lineage kinase 4 | KIAA1804 | 0,29 | 0,0334 | 0,13 | 0,3271 |
| 16699009 | lysophosphatidylglycerol acyltransferase 1 | LPGAT1 | 0,29 | 0,0447 | 0,11 | 0,5759 |
| 16889375 | NIF3 NGG1 interacting factor 3-like 1 (S. cerevisiae) | NIF3L1 | 0,29 | 0,0069 | 0,15 | 0,0531 |
| 17087758 | nipsnap homolog 3A (C. elegans) | NIPSNAP3A | 0,29 | 0,0169 | 0,22 | 0,0706 |
| 16947875 | protein kinase C, iota | PRKCI | 0,29 | 0,0256 | 0,28 | 0,1216 |
| 16969093 | RAP1, GTP-GDP dissociation stimulator 1 | RAP1GDS1 | 0,29 | 0,0452 | 0,19 | 0,2550 |
| 16729025 | ring finger protein 169 | RNF169 | 0,29 | 0,0127 | 0,16 | 0,1271 |
| 17117474 | ribosomal protein L23a pseudogene 82 \| CENPB DNA-binding domains containing 1 pseudogene \| ribosomal protein L23a pseudogene 7 \| ribosomal protein L23a pseudogene 4 \| uncharacterized LOC100508047 \| uncharacterized LOC100653267 \| 60S ribosomal protein L23a-like | RPL23AP82\| MGC2752\| RPL23AP7\| RPL23AP4\| LOC100508047\| LOC100653267\| LOC100653047\| LOC100289034\| LOC100287195\| FLJ43681 | 0,29 | 0,0203 | 0,15 | 0,6457 |
| 17099263 | senataxin | SETX | 0,29 | 0,0090 | 0,20 | 0,0874 |
| 16896374 | striatin, calmodulin binding protein | STRN | 0,29 | 0,0361 | 0,15 | 0,0982 |
| 16798755 | unc-51-like kinase 4 (C. elegans) pseudogene 2 \| unc-51-like kinase 4 (C. elegans) pseudogene 3 \| unc-51-like kinase 4 (C. elegans) pseudogene 1 \| CHRNA7 (cholinergic receptor, nicotinic, alpha 7, exons 5-10) and FAM7A (family with sequence similarity 7A, exons A-E) fusion | ULK4P2\| ULK4P3\| ULK4P1\| CHRFAM7A | 0,29 | 0,0144 | 0,20 | 0,2711 |
| 16722032 | ubiquitin specific peptidase 47 | USP47 | 0,29 | 0,0235 | 0,39 | 0,0572 |
| 16866131 | zinc finger protein 776 | ZNF776 | 0,29 | 0,0008 | 0,10 | 0,2679 |
| 16903028 | zinc finger, RAN-binding domain containing 3 | ZRANB3 | 0,29 | 0,0117 | 0,09 | 0,3729 |
| 16922920 | beta-site APP-cleaving enzyme 2 | BACE2 | 0,28 | 0,0457 | 0,01 | 0,8783 |
| 16797285 |  | BRF1 | 0,28 | 0,0337 | 0,16 | 0,4317 |
| 17090042 | chromosome 9 open reading frame 106 | C9orf106 | 0,28 | 0,0426 | 0,08 | 0,3887 |
| 16829422 | CENPB DNA-binding domains containing 1 | CENPBD1 | 0,28 | 0,0102 | 0,10 | 0,5959 |
| 16800406 | CTD (carboxy-terminal domain, RNA polymerase II, polypeptide A) small phosphatase like 2 | CTDSPL2 | 0,28 | 0,0394 | 0,03 | 0,8344 |
| 16717498 | CWF19-like 1, cell cycle control (S. pombe) | CWF19L1 | 0,28 | 0,0125 | 0,24 | 0,1553 |
| 16881615 | deoxyguanosine kinase | DGUOK | 0,28 | 0,0139 | -0,02 | 0,8413 |
| 16922724 | dual-specificity tyrosine-(Y)-phosphorylation regulated kinase 1A | DYRK1A | 0,28 | 0,0104 | 0,20 | 0,3026 |
| 17089711 |  | GLE1 | 0,28 | 0,0486 | 0,06 | 0,5464 |
| 17113725 | lysosomal-associated membrane protein 2 | LAMP2 | 0,28 | 0,0154 | 0,30 | 0,0731 |
| 16984945 | mitogen-activated protein kinase kinase kinase 1, E3 ubiquitin protein ligase | MAP3K1 | 0,28 | 0,0362 | 0,14 | 0,1848 |
| 17010461 | myosin VI | MYO6 | 0,28 | 0,0056 | 0,20 | 0,0830 |
| 16854268 | Niemann-Pick disease, type C1 | NPC1 | 0,28 | 0,0231 | 0,16 | 0,2412 |
| 16785952 | pecanex homolog (Drosophila) | PCNX | 0,28 | 0,0212 | 0,19 | 0,0523 |
| 16710095 | pleckstrin homology domain containing, family A (phosphoinositide binding specific) member 1 | PLEKHA1 | 0,28 | 0,0104 | 0,15 | 0,0935 |
| 17026002 | proteasome (prosome, macropain) subunit, beta type, 1 | PSMB1 | 0,28 | 0,0297 | 0,23 | 0,0953 |
| 16695558 | poliovirus receptor-related 4 | PVRL4 | 0,28 | 0,0263 | 0,22 | 0,1352 |
| 17083847 | Ras-related GTP binding A | RRAGA | 0,28 | 0,0227 | 0,15 | 0,0573 |
| 16989293 | SEC24 family, member A (S. cerevisiae) | SEC24A | 0,28 | 0,0359 | 0,24 | 0,1819 |
| 16702479 | Sec61 alpha 2 subunit (S. cerevisiae) | SEC61A2 | 0,28 | 0,0283 | 0,17 | 0,2450 |
| 17083261 | solute carrier family 1 (neuronal/epithelial high affinity glutamate transporter, system Xag), member 1 | SLC1A1 | 0,28 | 0,0374 | 0,27 | 0,2046 |
| 16869417 | syntaxin 10 | STX10 | 0,28 | 0,0414 | 0,18 | 0,2755 |
| 17077284 | transmembrane protein 68 | TMEM68 | 0,28 | 0,0409 | 0,03 | 0,5377 |
| 17060360 | tripartite motif containing 4 | TRIM4 | 0,28 | 0,0089 | 0,12 | 0,2504 |
| 16850896 | twisted gastrulation homolog 1 (Drosophila) | TWSG1 | 0,28 | 0,0055 | 0,02 | 0,6491 |
| 16705064 | ubiquitin-conjugating enzyme E2D 1 | UBE2D1 | 0,28 | 0,0208 | 0,21 | 0,2144 |
| 16769007 | UHRF1 binding protein 1-like | UHRF1BP1L | 0,28 | 0,0406 | 0,25 | 0,0764 |
| 16793805 | zinc finger and BTB domain containing 25 | ZBTB25 | 0,28 | 0,0244 | 0,13 | 0,2069 |
| 16891752 | ArfGAP with FG repeats 1 | AGFG1 | 0,27 | 0,0004 | 0,17 | 0,1301 |
| 16763600 | adhesion molecule with Ig-like domain 2 | AMIGO2 | 0,27 | 0,0354 | 0,07 | 0,6973 |
| 16730197 | ankyrin repeat domain 49 | ANKRD49 | 0,27 | 0,0043 | 0,19 | 0,2329 |
| 16793263 | autophagy related 14 | ATG14 | 0,27 | 0,0054 | 0,24 | 0,0577 |
| 16960807 | cyclin L1 | CCNL1 | 0,27 | 0,0424 | 0,22 | 0,2406 |
| 16819497 | coenzyme Q9 homolog (S. cerevisiae) | COQ9 | 0,27 | 0,0113 | 0,21 | 0,0908 |
| 16982269 | FAT tumor suppressor homolog 1 (Drosophila) | FAT1 | 0,27 | 0,0465 | 0,33 | 0,1245 |
| 16824301 | FGFR1OP N-terminal like | FOPNL | 0,27 | 0,0457 | 0,28 | 0,0689 |
| 16932103 | gamma-glutamyltransferase 3 pseudogene \| gamma-glutamyltransferase 1 \| gamma-glutamyltransferase 2 \| gamma-glutamyltransferase 8 pseudogene \| gamma-glutamyltransferase light chain 2 \| gamma-glutamyltransferase light chain 1 \| gamma-glutamyltransferase light chain 3 | GGT3P\| GGT1\| GGT2\| GGT8P\| GGTLC2\| GGTLC1\| GGTLC3 | 0,27 | 0,0464 | 0,26 | 0,1720 |
| 16938935 | golgin A4 | GOLGA4 | 0,27 | 0,0493 | 0,39 | 0,1270 |
| 16699320 | G patch domain containing 2 | GPATCH2 | 0,27 | 0,0015 | 0,16 | 0,1809 |
| 17032758 | HLA complex group 15 | HCG15 | 0,27 | 0,0264 | 0,17 | 0,1674 |
| 17006003 | HLA complex group 15 \| HLA complex group 16 | HCG15\| HCG16 | 0,27 | 0,0329 | 0,15 | 0,1809 |
| 16837689 | immature colon carcinoma transcript 1 | ICT1 | 0,27 | 0,0310 | 0,25 | 0,1580 |
| 16978301 | late endosomal/lysosomal adaptor, MAPK and MTOR activator 3 | LAMTOR3 | 0,27 | 0,0462 | 0,13 | 0,4043 |
| 17108816 | matrix-remodelling associated 5 | MXRA5 | 0,27 | 0,0326 | 0,11 | 0,3509 |
| 16752703 | NGFI-A binding protein 2 (EGR1 binding protein 2) | NAB2 | 0,27 | 0,0100 | -0,03 | 0,8384 |
| 16923289 | PBX/knotted 1 homeobox 1 | PKNOX1 | 0,27 | 0,0143 | 0,17 | 0,4918 |
| 16748888 | pleckstrin homology domain containing, family A member 5 | PLEKHA5 | 0,27 | 0,0355 | 0,27 | 0,1133 |
| 17108943 | patatin-like phospholipase domain containing 4 | PNPLA4 | 0,27 | 0,0050 | 0,13 | 0,0736 |
| 16756334 | polymerase (RNA) III (DNA directed) polypeptide B | POLR3B | 0,27 | 0,0391 | 0,30 | 0,0534 |
| 16791962 | protein phosphatase 2, regulatory subunit B'', gamma | PPP2R3C | 0,27 | 0,0263 | -0,05 | 0,7294 |
| 17101517 | phosphoribosyl pyrophosphate synthetase 2 | PRPS2 | 0,27 | 0,0345 | 0,22 | 0,1107 |
| 16990597 | RNA binding motif protein 27 | RBM27 | 0,27 | 0,0052 | 0,29 | 0,1204 |
| 16667801 | RNA-binding region (RNP1, RRM) containing 3 | RNPC3 | 0,27 | 0,0308 | 0,25 | 0,2253 |
| 16956244 | RING1 and YY1 binding protein | RYBP | 0,27 | 0,0200 | 0,27 | 0,0882 |
| 16677071 | SERTA domain containing 4 | SERTAD4 | 0,27 | 0,0070 | 0,06 | 0,4955 |
| 17045303 | STARD3 N-terminal like | STARD3NL | 0,27 | 0,0276 | 0,14 | 0,4864 |
| 16757720 | suppressor of defective silencing 3 homolog (S. cerevisiae) | SUDS3 | 0,27 | 0,0035 | 0,19 | 0,1170 |
| 16825763 | TBC1 domain family, member 10B | TBC1D10B | 0,27 | 0,0483 | 0,14 | 0,1842 |
| 16929882 | TRIO and F-actin binding protein \| nucleolar protein 12 | TRIOBP\| NOL12 | 0,27 | 0,0208 | -0,01 | 0,9211 |
| 16687132 | thioredoxin domain containing 12 (endoplasmic reticulum) | TXNDC12 | 0,27 | 0,0466 | 0,20 | 0,2845 |
| 16806692 | unc-51-like kinase 4 (C. elegans) pseudogene 3 \| unc-51-like kinase 4 (C. elegans) pseudogene 2 \| unc-51-like kinase 4 (C. elegans) pseudogene 1 \| CHRNA7 (cholinergic receptor, nicotinic, alpha 7, exons 5-10) and FAM7A (family with sequence similarity 7A, exons A-E) fusion | ULK4P3\| ULK4P2\| ULK4P1\| CHRFAM7A | 0,27 | 0,0397 | 0,12 | 0,3368 |
| 16770100 | vacuolar protein sorting 29 homolog (S. cerevisiae) | VPS29 | 0,27 | 0,0360 | 0,24 | 0,2547 |
| 16853171 | zinc finger protein 236 | ZNF236 | 0,27 | 0,0347 | 0,11 | 0,2955 |
| 17019365 | zinc finger protein 318 | ZNF318 | 0,27 | 0,0110 | 0,06 | 0,3610 |
| 17062580 | zinc finger protein 800 | ZNF800 | 0,27 | 0,0291 | 0,21 | 0,1526 |
| 16804333 | A kinase (PRKA) anchor protein 13 | AKAP13 | 0,26 | 0,0016 | 0,24 | 0,0613 |
| 17096546 | asparagine-linked glycosylation 2, alpha-1,3-mannosyltransferase homolog (S. cerevisiae) | ALG2 | 0,26 | 0,0278 | 0,21 | 0,2982 |
| 16762837 | caprin family member 2 | CAPRIN2 | 0,26 | 0,0316 | 0,31 | 0,1776 |
| 16952769 | CUB domain containing protein 1 | CDCP1 | 0,26 | 0,0233 | 0,24 | 0,0867 |
| 16823433 | CREB binding protein | CREBBP | 0,26 | 0,0432 | 0,14 | 0,3189 |
| 16688024 | dedicator of cytokinesis 7 | DOCK7 | 0,26 | 0,0281 | 0,22 | 0,2174 |
| 16979956 | E74-like factor 2 (ets domain transcription factor) | ELF2 | 0,26 | 0,0395 | 0,20 | 0,0747 |
| 17011593 |  | FIG4 | 0,26 | 0,0030 | 0,14 | 0,3812 |
| 17027222 | HLA complex group 15 | HCG15 | 0,26 | 0,0293 | 0,17 | 0,1775 |
| 17037562 | HLA complex group 15 | HCG15 | 0,26 | 0,0360 | 0,17 | 0,1794 |
| 17030069 | HLA complex group 15 | HCG15 | 0,26 | 0,0269 | 0,16 | 0,1808 |
| 17040272 | HLA complex group 15 | HCG15 | 0,26 | 0,0337 | 0,15 | 0,1864 |
| 17026042 | HLA complex group 15 | HCG15 | 0,26 | 0,0301 | 0,15 | 0,1873 |
| 17087034 | hippocampus abundant transcript-like 1 | HIATL1 | 0,26 | 0,0195 | 0,16 | 0,1552 |
| 16818114 | hydroxy-delta-5-steroid dehydrogenase, 3 beta- and steroid delta-isomerase 7 | HSD3B7 | 0,26 | 0,0294 | 0,28 | 0,0708 |
| 17068462 | inhibitor of kappa light polypeptide gene enhancer in B-cells, kinase beta | IKBKB | 0,26 | 0,0099 | 0,14 | 0,1603 |
| 16828734 | membrane-bound transcription factor peptidase, site 1 | MBTPS1 | 0,26 | 0,0474 | 0,01 | 0,9526 |
| 16981426 | microfibrillar-associated protein 3-like | MFAP3L | 0,26 | 0,0121 | 0,18 | 0,0954 |
| 16726164 | N(alpha)-acetyltransferase 40, NatD catalytic subunit, homolog (S. cerevisiae) | NAA40 | 0,26 | 0,0405 | 0,14 | 0,0870 |
| 16738205 | nucleoporin 160kDa | NUP160 | 0,26 | 0,0037 | 0,13 | 0,4831 |
| 17058812 | polymerase (RNA) II (DNA directed) polypeptide J4, pseudogene | POLR2J4 | 0,26 | 0,0374 | 0,05 | 0,8194 |
| 16882478 | required for meiotic nuclear division 5 homolog A (S. cerevisiae) | RMND5A | 0,26 | 0,0449 | 0,04 | 0,5917 |
| 16774917 | ribonuclease H2, subunit B | RNASEH2B | 0,26 | 0,0354 | -0,02 | 0,8443 |
| 16816978 | sodium channel, non-voltage-gated 1, beta subunit | SCNN1B | 0,26 | 0,0408 | 0,14 | 0,5339 |
| 17022295 |  | SEC63 | 0,26 | 0,0291 | 0,26 | 0,0756 |
| 16841941 | sterol regulatory element binding transcription factor 1 | SREBF1 | 0,26 | 0,0198 | 0,15 | 0,3451 |
| 17108562 | von Hippel-Lindau binding protein 1 | VBP1 | 0,26 | 0,0149 | 0,18 | 0,4001 |
| 16855697 | vacuolar protein sorting 4 homolog B (S. cerevisiae) | VPS4B | 0,26 | 0,0410 | 0,25 | 0,2103 |
| 16931558 | zinc finger, BED-type containing 4 | ZBED4 | 0,26 | 0,0152 | 0,07 | 0,3856 |
| 16774362 | A kinase (PRKA) anchor protein 11 | AKAP11 | 0,25 | 0,0234 | 0,16 | 0,1390 |
| 16847432 | BRCA1 interacting protein C-terminal helicase 1 | BRIP1 | 0,25 | 0,0477 | 0,17 | 0,0598 |
| 16819961 | chromosome 16 open reading frame 70 | C16orf70 | 0,25 | 0,0303 | 0,06 | 0,5898 |
| 16981330 | carbonyl reductase 4 | CBR4 | 0,25 | 0,0439 | 0,13 | 0,4488 |
| 16818724 | CTD nuclear envelope phosphatase 1 regulatory subunit 1 | CNEP1R1 | 0,25 | 0,0139 | 0,25 | 0,2467 |
| 16992557 | cytoplasmic polyadenylation element binding protein 4 | CPEB4 | 0,25 | 0,0115 | 0,08 | 0,0527 |
| 16779725 |  | DIS3 | 0,25 | 0,0191 | 0,14 | 0,5351 |
| 16744138 | exophilin 5 | EXPH5 | 0,25 | 0,0466 | 0,14 | 0,2770 |
| 16785483 | fucosyltransferase 8 (alpha (1,6) fucosyltransferase) | FUT8 | 0,25 | 0,0092 | 0,06 | 0,4633 |
| 17090646 | general transcription factor IIIC, polypeptide 4, 90kDa | GTF3C4 | 0,25 | 0,0101 | 0,04 | 0,2586 |
| 16932261 | HIR histone cell cycle regulation defective homolog A (S. cerevisiae) \| chromosome 22 open reading frame 39 | HIRA\| C22orf39 | 0,25 | 0,0163 | 0,09 | 0,1054 |
| 17099027 | immediate early response 5-like \| mannosyl (alpha-1,6-)-glycoprotein beta-1,2-N-acetylglucosaminyltransferase | IER5L\| MGAT2 | 0,25 | 0,0305 | 0,11 | 0,0738 |
| 17048706 | lemur tyrosine kinase 2 | LMTK2 | 0,25 | 0,0074 | 0,18 | 0,1396 |
| 16866702 | mex-3 homolog D (C. elegans) | MEX3D | 0,25 | 0,0086 | 0,04 | 0,7389 |
| 16815557 | mahogunin ring finger 1, E3 ubiquitin protein ligase | MGRN1 | 0,25 | 0,0298 | 0,16 | 0,1032 |
| 17093031 | MOB kinase activator 3B | MOB3B | 0,25 | 0,0261 | -0,04 | 0,7927 |
| 16816140 | pyridoxal-dependent decarboxylase domain containing 1 | PDXDC1 | 0,25 | 0,0146 | 0,11 | 0,2103 |
| 16759604 | phosphoglycerate mutase family member 5 \| peroxisomal membrane protein 2, 22kDa | PGAM5\| PXMP2 | 0,25 | 0,0038 | 0,15 | 0,0871 |
| 16967091 | RE1-silencing transcription factor | REST | 0,25 | 0,0114 | 0,20 | 0,2153 |
| 17018834 | SAYSVFN motif domain containing 1 | SAYSD1 | 0,25 | 0,0458 | 0,21 | 0,3510 |
| 16923200 | solute carrier family 37 (glycerol-3-phosphate transporter), member 1 | SLC37A1 | 0,25 | 0,0078 | 0,13 | 0,1034 |
| 16833384 |  | TAF15 | 0,25 | 0,0262 | 0,23 | 0,1416 |
| 17050522 | testis derived transcript (3 LIM domains) | TES | 0,25 | 0,0438 | 0,11 | 0,3763 |
| 17019805 | tumor necrosis factor receptor superfamily, member 21 | TNFRSF21 | 0,25 | 0,0262 | 0,20 | 0,4277 |
| 17016528 | transducer of ERBB2, 2 pseudogene 1 | TOB2P1 | 0,25 | 0,0247 | 0,17 | 0,4587 |
| 17052591 | T cell receptor beta variable 3-1 | TRBV3-1 | 0,25 | 0,0099 | 0,17 | 0,2904 |
| 16694429 | ubiquilin 4 | UBQLN4 | 0,25 | 0,0381 | 0,16 | 0,1306 |
| 17002813 | ubiquitin domain containing 2 | UBTD2 | 0,25 | 0,0323 | 0,19 | 0,3518 |
| 16970998 | ubiquitin specific peptidase 38 | USP38 | 0,25 | 0,0073 | 0,11 | 0,3812 |
| 16954743 | WD repeat domain 82 | WDR82 | 0,25 | 0,0097 | 0,08 | 0,6642 |
| 17096250 | zinc finger protein 510 | ZNF510 | 0,25 | 0,0288 | 0,22 | 0,0958 |
| 16928699 | zinc and ring finger 3 | ZNRF3 | 0,25 | 0,0299 | 0,13 | 0,3113 |
| 16729298 | alkaline ceramidase 3 | ACER3 | 0,24 | 0,0413 | 0,07 | 0,6315 |
| 16770200 | ataxin 2 | ATXN2 | 0,24 | 0,0067 | 0,18 | 0,0697 |
| 16887259 | Bardet-Biedl syndrome 5 | BBS5 | 0,24 | 0,0118 | 0,03 | 0,7379 |
| 16855673 | B-cell CLL/lymphoma 2 | BCL2 | 0,24 | 0,0396 | 0,08 | 0,3063 |
| 16860580 | chromosome 19 open reading frame 40 | C19orf40 | 0,24 | 0,0381 | 0,04 | 0,8199 |
| 17018993 | chromosome 6 open reading frame 130 | C6orf130 | 0,24 | 0,0257 | 0,03 | 0,8609 |
| 16813286 | calcium and integrin binding 1 (calmyrin) | CIB1 | 0,24 | 0,0195 | 0,10 | 0,2679 |
| 17025805 | dapper, antagonist of beta-catenin, homolog 2 (Xenopus laevis) | DACT2 | 0,24 | 0,0466 | 0,17 | 0,2896 |
| 16677913 | delta(4)-desaturase, sphingolipid 1 | DEGS1 | 0,24 | 0,0272 | 0,02 | 0,7938 |
| 16941067 | HemK methyltransferase family member 1 | HEMK1 | 0,24 | 0,0261 | 0,06 | 0,4881 |
| 16810376 | HECT and RLD domain containing E3 ubiquitin protein ligase family member 1 | HERC1 | 0,24 | 0,0443 | 0,10 | 0,0875 |
| 17016366 | histone cluster 1, H2ab \| histone cluster 1, H2ae | HIST1H2AB\| HIST1H2AE | 0,24 | 0,0205 | 0,10 | 0,5382 |
| 17005593 | histone cluster 1, H3e \| histone cluster 1, H3f \| histone cluster 1, H3b \| histone cluster 1, H3h \| histone cluster 1, H3j \| histone cluster 1, H3g \| histone cluster 1, H3i \| histone cluster 1, H3c \| histone cluster 1, H3d \| histone cluster 1, H3a | HIST1H3E\| HIST1H3F\| HIST1H3B\| HIST1H3H\| HIST1H3J\| HIST1H3G\| HIST1H3I\| HIST1H3C\| HIST1H3D\| HIST1H3A | 0,24 | 0,0079 | -0,01 | 0,9570 |
| 16937405 | jagunal homolog 1 (Drosophila) | JAGN1 | 0,24 | 0,0203 | 0,20 | 0,0821 |
| 16752715 | low density lipoprotein receptor-related protein 1 | LRP1 | 0,24 | 0,0341 | 0,28 | 0,0815 |
| 16669255 | mannosidase, alpha, class 1A, member 2 | MAN1A2 | 0,24 | 0,0182 | 0,21 | 0,1305 |
| 16866363 | CENPB DNA-binding domains containing 1 pseudogene \| uncharacterized LOC100653267 \| 60S ribosomal protein L23a-like \| ribosomal protein L23a pseudogene | MGC2752\| LOC100653267\| LOC100653047\| LOC100289034\| LOC100287195 | 0,24 | 0,0104 | 0,20 | 0,0946 |
| 17019208 | mitochondrial ribosomal protein S10 | MRPS10 | 0,24 | 0,0337 | 0,21 | 0,0955 |
| 16938034 | nuclear receptor subfamily 2, group C, member 2 | NR2C2 | 0,24 | 0,0179 | 0,06 | 0,4299 |
| 17013197 | peroxisomal biogenesis factor 3 | PEX3 | 0,24 | 0,0293 | 0,10 | 0,5517 |
| 16792519 | polymerase (DNA directed), epsilon 2, accessory subunit | POLE2 | 0,24 | 0,0046 | 0,12 | 0,1596 |
| 16976158 | phosphoribosyl pyrophosphate amidotransferase | PPAT | 0,24 | 0,0034 | 0,19 | 0,3465 |
| 16766923 | protein phosphatase, Mg2+/Mn2+ dependent, 1H | PPM1H | 0,24 | 0,0209 | 0,10 | 0,5678 |
| 17073865 | protein phosphatase 1, regulatory subunit 16A | PPP1R16A | 0,24 | 0,0208 | 0,22 | 0,1344 |
| 17097000 | protein tyrosine phosphatase, non-receptor type 3 | PTPN3 | 0,24 | 0,0233 | 0,02 | 0,8578 |
| 17022623 | REV3-like, polymerase (DNA directed), zeta, catalytic subunit | REV3L | 0,24 | 0,0280 | 0,20 | 0,2006 |
| 16830752 | ribosomal protein L29 pseudogene 2 | RPL29P2 | 0,24 | 0,0410 | 0,11 | 0,5171 |
| 17069545 |  | RRS1 | 0,24 | 0,0085 | 0,10 | 0,1646 |
| 17015084 | serpin peptidase inhibitor, clade B (ovalbumin), member 1 | SERPINB1 | 0,24 | 0,0046 | 0,09 | 0,6961 |
| 16989018 | solute carrier family 22 (organic cation/carnitine transporter), member 5 | SLC22A5 | 0,24 | 0,0474 | 0,13 | 0,1216 |
| 17057525 | tensin 3 | TNS3 | 0,24 | 0,0044 | -0,20 | 0,0871 |
| 16661298 | zinc finger, DHHC-type containing 18 | ZDHHC18 | 0,24 | 0,0372 | 0,22 | 0,2935 |
| 16873183 | zinc finger protein 180 | ZNF180 | 0,24 | 0,0318 | 0,11 | 0,5624 |
| 16858509 | zinc finger protein 627 | ZNF627 | 0,24 | 0,0307 | 0,09 | 0,7219 |
| 17078539 | zinc finger protein 704 | ZNF704 | 0,24 | 0,0370 | -0,08 | 0,2117 |
| 16815425 | zinc finger protein 75a | ZNF75A | 0,24 | 0,0007 | 0,19 | 0,1536 |
| 16902611 | AMME chromosomal region gene 1-like | AMMECR1L | 0,23 | 0,0276 | 0,08 | 0,5236 |
| 17093147 | aprataxin \| L antigen family, member 3 pseudogene | APTX\| LOC646808 | 0,23 | 0,0100 | 0,24 | 0,0554 |
| 16719066 | arginyltransferase 1 | ATE1 | 0,23 | 0,0008 | 0,11 | 0,4262 |
| 16996722 | centromere protein K | CENPK | 0,23 | 0,0039 | 0,11 | 0,2908 |
| 16785437 |  | CHURC1-FNTB | 0,23 | 0,0160 | 0,12 | 0,2162 |
| 16927401 | v-crk sarcoma virus CT10 oncogene homolog (avian)-like | CRKL | 0,23 | 0,0046 | -0,01 | 0,8608 |
| 16818842 | cylindromatosis (turban tumor syndrome) | CYLD | 0,23 | 0,0286 | 0,04 | 0,7498 |
| 16913689 | DEAH (Asp-Glu-Ala-His) box polypeptide 35 | DHX35 | 0,23 | 0,0258 | 0,07 | 0,6436 |
| 16921134 | death inducer-obliterator 1 | DIDO1 | 0,23 | 0,0104 | 0,00 | 0,9851 |
| 17065525 | exoribonuclease 1 | ERI1 | 0,23 | 0,0015 | 0,24 | 0,0635 |
| 16658950 | exosome component 10 | EXOSC10 | 0,23 | 0,0350 | 0,33 | 0,1204 |
| 16890005 | FAST kinase domains 2 | FASTKD2 | 0,23 | 0,0325 | 0,20 | 0,1332 |
| 16690036 | ferric-chelate reductase 1 | FRRS1 | 0,23 | 0,0330 | 0,19 | 0,2932 |
| 16801428 | GRINL1A complex locus 1 \| myocardial zonula adherens protein \| polymerase (RNA) II (DNA directed) polypeptide M \| GRINL1B complex locus 2, pseudogene | GCOM1\| MYZAP\| POLR2M\| GCOM2 | 0,23 | 0,0038 | 0,12 | 0,0555 |
| 16925466 | holocarboxylase synthetase (biotin-(proprionyl-CoA-carboxylase (ATP-hydrolysing)) ligase) | HLCS | 0,23 | 0,0261 | 0,08 | 0,3848 |
| 17014364 | insulin-like growth factor 2 receptor | IGF2R | 0,23 | 0,0485 | 0,23 | 0,1645 |
| 17009676 | leucine rich repeat containing 1 | LRRC1 | 0,23 | 0,0316 | 0,01 | 0,8821 |
| 16677259 | neudesin neurotrophic factor | NENF | 0,23 | 0,0040 | 0,25 | 0,2581 |
| 16920315 | nuclear factor of activated T-cells, cytoplasmic, calcineurin-dependent 2 | NFATC2 | 0,23 | 0,0205 | 0,15 | 0,3267 |
| 16929866 | nucleolar protein 12 | NOL12 | 0,23 | 0,0391 | 0,04 | 0,7058 |
| 17080171 | NudC domain containing 1 | NUDCD1 | 0,23 | 0,0486 | 0,12 | 0,0697 |
| 17041368 | PHD finger protein 1 | PHF1 | 0,23 | 0,0386 | 0,05 | 0,5798 |
| 16848032 | proteasome (prosome, macropain) 26S subunit, non-ATPase, 12 | PSMD12 | 0,23 | 0,0006 | 0,13 | 0,0709 |
| 17101616 |  | RAB9A | 0,23 | 0,0319 | 0,23 | 0,0547 |
| 17050055 | RAD50 interactor 1 | RINT1 | 0,23 | 0,0225 | 0,29 | 0,0751 |
| 16926050 | radial spoke head 1 homolog (Chlamydomonas) | RSPH1 | 0,23 | 0,0171 | 0,16 | 0,2656 |
| 16714965 | RUN and FYVE domain containing 2 | RUFY2 | 0,23 | 0,0338 | 0,16 | 0,2636 |
| 16840245 | SLP adaptor and CSK interacting membrane protein | SCIMP | 0,23 | 0,0331 | 0,06 | 0,7751 |
| 16663033 | small ArfGAP2 | SMAP2 | 0,23 | 0,0077 | 0,12 | 0,0875 |
| 16861630 | serine peptidase inhibitor, Kunitz type, 2 | SPINT2 | 0,23 | 0,0444 | 0,11 | 0,2391 |
| 16902254 | transmembrane protein 185B | TMEM185B | 0,23 | 0,0218 | 0,02 | 0,7139 |
| 16882304 | ubiquitin specific peptidase 39 | USP39 | 0,23 | 0,0201 | 0,13 | 0,1544 |
| 16898245 | WD repeat containing planar cell polarity effector | WDPCP | 0,23 | 0,0337 | 0,04 | 0,7558 |
| 16662359 | zinc finger, MYM-type 4 | ZMYM4 | 0,23 | 0,0363 | 0,04 | 0,4687 |
| 16777167 | zinc finger, MYM-type 5 | ZMYM5 | 0,23 | 0,0404 | 0,16 | 0,2063 |
| 16818431 | zinc finger protein 267 | ZNF267 | 0,23 | 0,0257 | 0,15 | 0,0591 |
| 16933591 | adaptor-related protein complex 1, beta 1 subunit | AP1B1 | 0,22 | 0,0460 | 0,16 | 0,1975 |
| 16892075 | armadillo repeat containing 9 | ARMC9 | 0,22 | 0,0234 | 0,17 | 0,1708 |
| 16955119 | choline dehydrogenase | CHDH | 0,22 | 0,0146 | 0,18 | 0,0916 |
| 16991527 | cytoplasmic FMR1 interacting protein 2 | CYFIP2 | 0,22 | 0,0372 | 0,12 | 0,1009 |
| 16768478 | early endosome antigen 1 | EEA1 | 0,22 | 0,0187 | 0,12 | 0,1295 |
| 16757657 | F-box and WD repeat domain containing 8 | FBXW8 | 0,22 | 0,0005 | 0,04 | 0,4785 |
| 16901393 | four and a half LIM domains 2 | FHL2 | 0,22 | 0,0146 | 0,09 | 0,5911 |
| 16685958 | forkhead box J3 | FOXJ3 | 0,22 | 0,0017 | 0,04 | 0,5487 |
| 17005396 | geminin, DNA replication inhibitor | GMNN | 0,22 | 0,0414 | 0,20 | 0,2334 |
| 16928668 | HscB iron-sulfur cluster co-chaperone homolog (E. coli) | HSCB | 0,22 | 0,0313 | 0,11 | 0,2371 |
| 16876278 | killer cell immunoglobulin-like receptor, two domains, short cytoplasmic tail, 1 \| killer cell immunoglobulin-like receptor, two domains, long cytoplasmic tail, 1 | KIR2DS1\| KIR2DL1 | 0,22 | 0,0128 | 0,18 | 0,1180 |
| 16736821 | leucine-rich repeat containing G protein-coupled receptor 4 | LGR4 | 0,22 | 0,0327 | 0,24 | 0,1303 |
| 17084932 | endogenous Bornavirus-like nucleoprotein 2 pseudogene | LOC100506710 | 0,22 | 0,0432 | 0,20 | 0,1470 |
| 16852331 | malic enzyme 2, NAD(+)-dependent, mitochondrial | ME2 | 0,22 | 0,0453 | 0,14 | 0,4584 |
| 16719515 | antigen identified by monoclonal antibody Ki-67 | MKI67 | 0,22 | 0,0099 | 0,15 | 0,2347 |
| 17044382 | membrane protein, palmitoylated 6 (MAGUK p55 subfamily member 6) | MPP6 | 0,22 | 0,0071 | 0,20 | 0,1920 |
| 16984542 | mitochondrial ribosomal protein S30 | MRPS30 | 0,22 | 0,0494 | 0,20 | 0,1799 |
| 16746104 | nuclear factor related to kappaB binding protein | NFRKB | 0,22 | 0,0277 | 0,21 | 0,1029 |
| 17071776 | oxidation resistance 1 | OXR1 | 0,22 | 0,0388 | 0,11 | 0,0633 |
| 16824082 | poly(A)-specific ribonuclease | PARN | 0,22 | 0,0228 | 0,08 | 0,0684 |
| 16846157 | proline rich 15-like | PRR15L | 0,22 | 0,0130 | 0,22 | 0,3272 |
| 17015752 | RAN binding protein 9 | RANBP9 | 0,22 | 0,0414 | 0,18 | 0,1580 |
| 16965642 | recombination signal binding protein for immunoglobulin kappa J region | RBPJ | 0,22 | 0,0135 | 0,17 | 0,1583 |
| 16843340 | ring finger and FYVE-like domain containing E3 ubiquitin protein ligase \| RAD51L3-RFFL readthrough \| RAD51 homolog D (S. cerevisiae) | RFFL\| RAD51L3-RFFL\| RAD51D | 0,22 | 0,0196 | 0,07 | 0,4410 |
| 17021596 | Ras-related GTP binding D | RRAGD | 0,22 | 0,0058 | 0,15 | 0,3671 |
| 16771756 | arginine/serine-rich coiled-coil 2 | RSRC2 | 0,22 | 0,0447 | 0,06 | 0,6936 |
| 17098364 | suppressor of cancer cell invasion | SCAI | 0,22 | 0,0459 | 0,13 | 0,2379 |
| 16811936 | S-phase cyclin A-associated protein in the ER | SCAPER | 0,22 | 0,0307 | 0,26 | 0,1290 |
| 16670632 | SET domain, bifurcated 1 | SETDB1 | 0,22 | 0,0314 | 0,07 | 0,6830 |
| 17025595 | SFT2 domain containing 1 | SFT2D1 | 0,22 | 0,0136 | 0,25 | 0,0542 |
| 16890621 | SWI/SNF related, matrix associated, actin dependent regulator of chromatin, subfamily a-like 1 | SMARCAL1 | 0,22 | 0,0082 | 0,15 | 0,2558 |
| 16787236 | spermatogenesis associated 7 | SPATA7 | 0,22 | 0,0030 | 0,24 | 0,2646 |
| 16966897 | steroid 5 alpha-reductase 3 | SRD5A3 | 0,22 | 0,0108 | 0,14 | 0,0800 |
| 16723369 | t-complex 11 (mouse)-like 1 | TCP11L1 | 0,22 | 0,0024 | 0,14 | 0,1547 |
| 16847795 | testis expressed 2 | TEX2 | 0,22 | 0,0306 | 0,16 | 0,2060 |
| 16687985 | TM2 domain containing 1 | TM2D1 | 0,22 | 0,0166 | 0,14 | 0,1978 |
| 16919663 | troponin C type 2 (fast) | TNNC2 | 0,22 | 0,0401 | 0,30 | 0,1437 |
| 16893958 | tumor suppressing subtransferable candidate 1 | TSSC1 | 0,22 | 0,0339 | 0,19 | 0,0823 |
| 17010552 |  | TTK | 0,22 | 0,0123 | 0,09 | 0,7098 |
| 16982124 | UFM1-specific peptidase 2 | UFSP2 | 0,22 | 0,0264 | 0,17 | 0,1545 |
| 16972710 | WW and C2 domain containing 2 \| claudin 22 | WWC2\| CLDN22 | 0,22 | 0,0032 | 0,06 | 0,6423 |
| 16767540 | zinc finger, C3H1-type containing | ZFC3H1 | 0,22 | 0,0178 | 0,29 | 0,0880 |
| 16891575 | acyl-CoA synthetase long-chain family member 3 | ACSL3 | 0,21 | 0,0039 | 0,06 | 0,4131 |
| 16680323 | ankyrin repeat domain 65 | ANKRD65 | 0,21 | 0,0171 | 0,02 | 0,8556 |
| 16733435 | amyloid beta (A4) precursor-like protein 2 | APLP2 | 0,21 | 0,0176 | 0,13 | 0,2287 |
| 16773552 | ATP synthase, H+ transporting, mitochondrial F1 complex, epsilon subunit pseudogene 2 | ATP5EP2 | 0,21 | 0,0284 | 0,07 | 0,1990 |
| 16797014 | BCL2-associated athanogene 5 | BAG5 | 0,21 | 0,0425 | 0,10 | 0,1240 |
| 16796590 | B-cell CLL/lymphoma 11B (zinc finger protein) | BCL11B | 0,21 | 0,0497 | 0,49 | 0,0796 |
| 16707949 | chromosome 10 open reading frame 12 | C10orf12 | 0,21 | 0,0262 | 0,14 | 0,0856 |
| 17062878 | centrosomal protein 41kDa | CEP41 | 0,21 | 0,0453 | 0,21 | 0,1240 |
| 17098222 | DENN/MADD domain containing 1A | DENND1A | 0,21 | 0,0497 | 0,08 | 0,2710 |
| 17050300 | dihydrolipoamide dehydrogenase | DLD | 0,21 | 0,0337 | 0,26 | 0,0793 |
| 17099114 | formin binding protein 1 | FNBP1 | 0,21 | 0,0235 | 0,10 | 0,3742 |
| 16702656 | heat shock 70kDa protein 14 | HSPA14 | 0,21 | 0,0152 | 0,24 | 0,0983 |
| 16881786 | HtrA serine peptidase 2 | HTRA2 | 0,21 | 0,0093 | 0,22 | 0,1742 |
| 16745041 | intraflagellar transport 46 homolog (Chlamydomonas) | IFT46 | 0,21 | 0,0313 | 0,04 | 0,6775 |
| 16892446 | inositol polyphosphate-5-phosphatase, 145kDa | INPP5D | 0,21 | 0,0406 | 0,20 | 0,0558 |
| 16874347 | interferon regulatory factor 3 | IRF3 | 0,21 | 0,0048 | 0,15 | 0,0762 |
| 16746831 | integrin alpha FG-GAP repeat containing 2 \| uncharacterized LOC100507424 | ITFG2\| LOC100507424 | 0,21 | 0,0168 | 0,11 | 0,3207 |
| 16728889 | mitochondrial ribosomal protein L48 | MRPL48 | 0,21 | 0,0374 | 0,04 | 0,8781 |
| 16883912 | NCK adaptor protein 2 | NCK2 | 0,21 | 0,0354 | 0,13 | 0,2375 |
| 16755433 | neural precursor cell expressed, developmentally down-regulated 1 | NEDD1 | 0,21 | 0,0374 | 0,05 | 0,6330 |
| 16820584 | nuclear import 7 homolog (S. cerevisiae) | NIP7 | 0,21 | 0,0143 | 0,24 | 0,1241 |
| 16839412 | phosphatidylinositol transfer protein, alpha | PITPNA | 0,21 | 0,0280 | 0,12 | 0,3107 |
| 16892039 | proteasome (prosome, macropain) 26S subunit, non-ATPase, 1 | PSMD1 | 0,21 | 0,0388 | 0,10 | 0,1400 |
| 16802106 | protein tyrosine phosphatase-like A domain containing 1 | PTPLAD1 | 0,21 | 0,0493 | 0,15 | 0,0912 |
| 16941024 | sema domain, immunoglobulin domain (Ig), short basic domain, secreted, (semaphorin) 3B | SEMA3B | 0,21 | 0,0197 | 0,10 | 0,3465 |
| 16770553 | solute carrier family 24 (sodium/lithium/calcium exchanger), member 6 | SLC24A6 | 0,21 | 0,0330 | 0,12 | 0,1497 |
| 16951756 | solute carrier family 4, sodium bicarbonate cotransporter, member 7 | SLC4A7 | 0,21 | 0,0468 | -0,01 | 0,9216 |
| 16850063 | stimulated by retinoic acid 13 homolog (mouse) | STRA13 | 0,21 | 0,0311 | 0,22 | 0,0655 |
| 16794297 |  | SYNJ2BP-COX16 | 0,21 | 0,0470 | 0,18 | 0,1218 |
| 16775166 | tudor domain containing 3 | TDRD3 | 0,21 | 0,0409 | 0,19 | 0,1648 |
| 17062853 | transmembrane protein 209 | TMEM209 | 0,21 | 0,0096 | 0,16 | 0,1031 |
| 16697843 | transmembrane protein 9 | TMEM9 | 0,21 | 0,0303 | 0,18 | 0,2960 |
| 17065958 | tumor suppressor candidate 3 | TUSC3 | 0,21 | 0,0005 | 0,09 | 0,5088 |
| 16954730 | twinfilin, actin-binding protein, homolog 2 (Drosophila) | TWF2 | 0,21 | 0,0060 | -0,04 | 0,6792 |
| 17008595 | ubiquitin protein ligase E3 component n-recognin 2 | UBR2 | 0,21 | 0,0448 | 0,14 | 0,1583 |
| 17102689 | ubiquitin specific peptidase 9, X-linked | USP9X | 0,21 | 0,0435 | 0,12 | 0,2502 |
| 16763764 | vitamin D (1,25- dihydroxyvitamin D3) receptor | VDR | 0,21 | 0,0339 | 0,07 | 0,4941 |
| 16786492 | YLP motif containing 1 \| uncharacterized LOC100506536 | YLPM1\| LOC100506536 | 0,21 | 0,0415 | 0,08 | 0,6832 |
| 16929347 | tyrosine 3-monooxygenase/tryptophan 5-monooxygenase activation protein, eta polypeptide | YWHAH | 0,21 | 0,0141 | 0,01 | 0,9528 |
| 17043418 | zinc finger, DHHC-type containing 4 | ZDHHC4 | 0,21 | 0,0104 | 0,19 | 0,1199 |
| 17010789 | zinc finger protein 292 | ZNF292 | 0,21 | 0,0467 | 0,16 | 0,1836 |
| 16833441 | zinc finger, HIT-type containing 3 | ZNHIT3 | 0,21 | 0,0111 | 0,21 | 0,1129 |
| 16853345 | ADNP homeobox 2 | ADNP2 | 0,2 | 0,0125 | 0,03 | 0,8288 |
| 16770040 | anaphase promoting complex subunit 7 | ANAPC7 | 0,2 | 0,0169 | 0,04 | 0,4110 |
| 16975084 | ArfGAP with RhoGAP domain, ankyrin repeat and PH domain 2 | ARAP2 | 0,2 | 0,0090 | 0,11 | 0,2193 |
| 16783215 | Rho GTPase activating protein 5 | ARHGAP5 | 0,2 | 0,0179 | 0,14 | 0,2531 |
| 16943090 | ADP-ribosylation factor-like 6 | ARL6 | 0,2 | 0,0263 | 0,22 | 0,2394 |
| 16942392 | ataxin 7 | ATXN7 | 0,2 | 0,0354 | 0,05 | 0,4405 |
| 16954770 | BRCA1 associated protein-1 (ubiquitin carboxy-terminal hydrolase) | BAP1 | 0,2 | 0,0299 | 0,08 | 0,1912 |
| 16852218 | chromosome 18 open reading frame 25 | C18orf25 | 0,2 | 0,0026 | 0,12 | 0,1336 |
| 16851461 | chromosome 18 open reading frame 8 | C18orf8 | 0,2 | 0,0475 | 0,08 | 0,4803 |
| 16662676 | chromosome 1 open reading frame 122 | C1orf122 | 0,2 | 0,0288 | 0,01 | 0,9485 |
| 16980998 | chromosome 4 open reading frame 46 | C4orf46 | 0,2 | 0,0237 | 0,14 | 0,1653 |
| 16924785 | claudin 17 | CLDN17 | 0,2 | 0,0028 | 0,08 | 0,3836 |
| 16678611 | component of oligomeric golgi complex 2 | COG2 | 0,2 | 0,0309 | 0,08 | 0,5618 |
| 16974873 | DEAH (Asp-Glu-Ala-His) box polypeptide 15 | DHX15 | 0,2 | 0,0362 | 0,12 | 0,1996 |
| 16746640 | ELKS/RAB6-interacting/CAST family member 1 | ERC1 | 0,2 | 0,0402 | 0,18 | 0,2099 |
| 17064105 | enhancer of zeste homolog 2 (Drosophila) | EZH2 | 0,2 | 0,0475 | 0,14 | 0,3406 |
| 16781285 | growth arrest-specific 6 | GAS6 | 0,2 | 0,0408 | 0,28 | 0,1097 |
| 16884062 | GRIP and coiled-coil domain containing 2 | GCC2 | 0,2 | 0,0371 | 0,12 | 0,2663 |
| 16947418 | G elongation factor, mitochondrial 1 | GFM1 | 0,2 | 0,0034 | 0,03 | 0,3861 |
| 16811437 | hexosaminidase A (alpha polypeptide) | HEXA | 0,2 | 0,0493 | 0,08 | 0,5718 |
| 16754111 | potassium large conductance calcium-activated channel, subfamily M, beta member 4 | KCNMB4 | 0,2 | 0,0170 | 0,08 | 0,2118 |
| 16874702 | kallikrein-related peptidase 7 | KLK7 | 0,2 | 0,0367 | 0,32 | 0,0539 |
| 16937795 | makorin ring finger protein 2 | MKRN2 | 0,2 | 0,0034 | 0,22 | 0,0908 |
| 16794864 | mutL homolog 3 (E. coli) | MLH3 | 0,2 | 0,0110 | 0,18 | 0,2499 |
| 16967602 | MOB kinase activator 1B | MOB1B | 0,2 | 0,0311 | 0,10 | 0,6186 |
| 17105903 | melanoma associated antigen (mutated) 1-like 1 | MUM1L1 | 0,2 | 0,0350 | 0,14 | 0,2602 |
| 16954925 | NIMA (never in mitosis gene a)-related kinase 4 | NEK4 | 0,2 | 0,0337 | 0,06 | 0,1651 |
| 17063480 | poly (ADP-ribose) polymerase family, member 12 | PARP12 | 0,2 | 0,0075 | -0,13 | 0,0784 |
| 16777460 | poly (ADP-ribose) polymerase family, member 4 | PARP4 | 0,2 | 0,0219 | -0,09 | 0,3229 |
| 16852789 | PH domain and leucine rich repeat protein phosphatase 1 | PHLPP1 | 0,2 | 0,0368 | 0,10 | 0,3352 |
| 16886757 | plakophilin 4 | PKP4 | 0,2 | 0,0473 | 0,03 | 0,8122 |
| 16997347 |  | POC5 | 0,2 | 0,0302 | 0,09 | 0,1137 |
| 16753994 | RAB3A interacting protein (rabin3) \| chromosome 12 open reading frame 28 \| myelin gene regulatory factor-like | RAB3IP\| C12orf28\| LOC100652962 | 0,2 | 0,0201 | 0,06 | 0,1201 |
| 16855629 | ring finger protein 152 | RNF152 | 0,2 | 0,0205 | -0,10 | 0,1948 |
| 16731670 | ring finger protein 214 | RNF214 | 0,2 | 0,0070 | 0,16 | 0,1549 |
| 16820787 | splicing factor 3b, subunit 3, 130kDa | SF3B3 | 0,2 | 0,0102 | 0,09 | 0,0924 |
| 16727770 | slingshot homolog 3 (Drosophila) | SSH3 | 0,2 | 0,0412 | 0,10 | 0,4123 |
| 17112956 | transcription elongation factor A (SII)-like 8 | TCEAL8 | 0,2 | 0,0274 | 0,40 | 0,0556 |
| 16899619 | trans-golgi network protein 2 | TGOLN2 | 0,2 | 0,0022 | 0,03 | 0,7148 |
| 17113866 | THO complex 2 | THOC2 | 0,2 | 0,0453 | 0,19 | 0,2121 |
| 16676071 | transmembrane protein 183B \| transmembrane protein 183A | TMEM183B\| TMEM183A | 0,2 | 0,0441 | 0,33 | 0,2753 |
| 17014169 | tubby like protein 4 | TULP4 | 0,2 | 0,0043 | 0,17 | 0,1997 |
| 17101323 | variable charge, X-linked 3B \| variable charge, X-linked 2 \| variable charge, X-linked 3A | VCX3B\| VCX2\| VCX3A | 0,2 | 0,0210 | 0,10 | 0,3066 |
| 16771652 | vacuolar protein sorting 33 homolog A (S. cerevisiae) | VPS33A | 0,2 | 0,0207 | 0,19 | 0,2096 |
| 16856828 | zinc finger protein 554 | ZNF554 | 0,2 | 0,0258 | 0,15 | 0,0747 |
| 16866292 | zinc finger protein 584 | ZNF584 | 0,2 | 0,0442 | 0,13 | 0,5487 |
| 16939534 | zinc finger protein 621 | ZNF621 | 0,2 | 0,0491 | 0,07 | 0,6885 |
| 16965559 | anaphase promoting complex subunit 4 | ANAPC4 | 0,19 | 0,0016 | 0,17 | 0,1179 |
| 16670114 | B-cell CLL/lymphoma 9 | BCL9 | 0,19 | 0,0488 | 0,24 | 0,1144 |
| 16849625 | chromobox homolog 8 | CBX8 | 0,19 | 0,0306 | 0,03 | 0,5219 |
| 17108764 | DHRSX intronic transcript 1 (non-protein coding) | DHRSX-IT1 | 0,19 | 0,0097 | 0,10 | 0,2264 |
| 16909319 | delta/notch-like EGF repeat containing | DNER | 0,19 | 0,0460 | 0,11 | 0,0685 |
| 16698262 | ethanolamine kinase 2 | ETNK2 | 0,19 | 0,0300 | -0,03 | 0,6936 |
| 16858235 | interleukin enhancer binding factor 3, 90kDa | ILF3 | 0,19 | 0,0258 | 0,13 | 0,0534 |
| 16802519 | kinesin family member 23 | KIF23 | 0,19 | 0,0235 | 0,07 | 0,4452 |
| 16711089 | La ribonucleoprotein domain family, member 4B | LARP4B | 0,19 | 0,0251 | 0,01 | 0,8650 |
| 16994170 | NOP2/Sun RNA methyltransferase family, member 2 | NSUN2 | 0,19 | 0,0071 | 0,07 | 0,1047 |
| 16963798 | polycomb group ring finger 3 | PCGF3 | 0,19 | 0,0038 | 0,19 | 0,2165 |
| 16914374 | PDX1 C-terminal inhibiting factor 1 | PCIF1 | 0,19 | 0,0198 | 0,10 | 0,0882 |
| 16975345 | PDS5, regulator of cohesion maintenance, homolog A (S. cerevisiae) | PDS5A | 0,19 | 0,0188 | 0,11 | 0,1181 |
| 16727413 | pellino E3 ubiquitin protein ligase family member 3 | PELI3 | 0,19 | 0,0073 | 0,08 | 0,3898 |
| 16703036 | plexin domain containing 2 | PLXDC2 | 0,19 | 0,0162 | 0,12 | 0,2109 |
| 16713454 | RasGEF domain family, member 1A | RASGEF1A | 0,19 | 0,0026 | 0,09 | 0,2441 |
| 16803317 | reticulocalbin 2, EF-hand calcium binding domain | RCN2 | 0,19 | 0,0007 | 0,03 | 0,6572 |
| 16911835 | Ras and Rab interactor 2 | RIN2 | 0,19 | 0,0428 | 0,08 | 0,4622 |
| 16691078 | round spermatid basic protein 1 | RSBN1 | 0,19 | 0,0325 | 0,22 | 0,1572 |
| 16892750 | SH3-domain binding protein 4 | SH3BP4 | 0,19 | 0,0365 | 0,09 | 0,2645 |
| 16658664 | solute carrier family 25 (pyrimidine nucleotide carrier), member 33 | SLC25A33 | 0,19 | 0,0375 | 0,02 | 0,7881 |
| 16835386 | sorting nexin 11 | SNX11 | 0,19 | 0,0134 | 0,25 | 0,1919 |
| 16969414 | tet methylcytosine dioxygenase 2 | TET2 | 0,19 | 0,0018 | 0,14 | 0,2830 |
| 16816604 | acyl-CoA synthetase medium-chain family member 3 | ACSM3 | 0,18 | 0,0486 | 0,05 | 0,0580 |
| 17064235 | ARP3 actin-related protein 3 homolog C (yeast) | ACTR3C | 0,18 | 0,0121 | 0,11 | 0,3811 |
| 17048246 | A kinase (PRKA) anchor protein (yotiao) 9 | AKAP9 | 0,18 | 0,0182 | 0,08 | 0,2130 |
| 16936255 | asparagine-linked glycosylation 12, alpha-1,6-mannosyltransferase homolog (S. cerevisiae) | ALG12 | 0,18 | 0,0239 | 0,01 | 0,7711 |
| 16805474 | arrestin domain containing 4 | ARRDC4 | 0,18 | 0,0022 | 0,20 | 0,1041 |
| 17080749 | ATPase family, AAA domain containing 2 | ATAD2 | 0,18 | 0,0380 | 0,17 | 0,2681 |
| 16731189 | chromosome 11 open reading frame 57 | C11orf57 | 0,18 | 0,0361 | 0,15 | 0,1481 |
| 16918011 | chromosome 20 open reading frame 3 | C20orf3 | 0,18 | 0,0099 | 0,07 | 0,0894 |
| 16900910 | chromosome 2 open reading frame 55 | C2orf55 | 0,18 | 0,0496 | 0,16 | 0,0765 |
| 16896882 | cytochrome c oxidase subunit VIIa polypeptide 2 like | COX7A2L | 0,18 | 0,0143 | 0,15 | 0,0623 |
| 17093377 | DDB1 and CUL4 associated factor 12 | DCAF12 | 0,18 | 0,0419 | -0,04 | 0,0585 |
| 16668730 | DEAD (Asp-Glu-Ala-Asp) box polypeptide 20 | DDX20 | 0,18 | 0,0055 | 0,11 | 0,5661 |
| 17080693 | derlin 1 | DERL1 | 0,18 | 0,0008 | 0,06 | 0,2688 |
| 16812997 | de-etiolated homolog 1 (Arabidopsis) | DET1 | 0,18 | 0,0425 | 0,30 | 0,1208 |
| 17116748 | dehydrogenase/reductase (SDR family) X-linked \| zinc finger, BED-type containing 1 | DHRSX\| ZBED1 | 0,18 | 0,0248 | -0,09 | 0,4585 |
| 16781219 | growth hormone regulated TBC protein 1 | GRTP1 | 0,18 | 0,0419 | -0,07 | 0,4071 |
| 16957843 | glycogen synthase kinase 3 beta | GSK3B | 0,18 | 0,0365 | 0,17 | 0,1254 |
| 16946762 | glycogenin 1 | GYG1 | 0,18 | 0,0087 | 0,11 | 0,2136 |
| 16713470 | heterogeneous nuclear ribonucleoprotein F | HNRNPF | 0,18 | 0,0341 | 0,10 | 0,3885 |
| 16797490 | immunoglobulin heavy variable 3-20 | IGHV3-20 | 0,18 | 0,0272 | 0,24 | 0,4981 |
| 16980160 | inositol polyphosphate-4-phosphatase, type II, 105kDa | INPP4B | 0,18 | 0,0388 | -0,09 | 0,5123 |
| 17018841 | potassium channel, subfamily K, member 5 | KCNK5 | 0,18 | 0,0278 | 0,10 | 0,2319 |
| 16658802 | kinesin family member 1B | KIF1B | 0,18 | 0,0411 | 0,09 | 0,3671 |
| 16985132 | kinesin heavy chain member 2A | KIF2A | 0,18 | 0,0189 | 0,14 | 0,3944 |
| 16844828 | keratin 17 | KRT17 | 0,18 | 0,0274 | 0,09 | 0,3642 |
| 16880669 | lectin, galactoside-binding-like | LGALSL | 0,18 | 0,0273 | 0,19 | 0,2400 |
| 16742115 | lipoyl(octanoyl) transferase 2 (putative) | LIPT2 | 0,18 | 0,0163 | 0,16 | 0,5629 |
| 16817692 | major vault protein | MVP | 0,18 | 0,0112 | 0,13 | 0,2283 |
| 16827170 | NEDD8 activating enzyme E1 subunit 1 | NAE1 | 0,18 | 0,0081 | 0,12 | 0,0848 |
| 16918953 | NDRG family member 3 | NDRG3 | 0,18 | 0,0156 | 0,10 | 0,3743 |
| 16995409 | nucleoporin 155kDa | NUP155 | 0,18 | 0,0442 | -0,01 | 0,8115 |
| 16929015 | oxysterol binding protein 2 | OSBP2 | 0,18 | 0,0113 | 0,13 | 0,0890 |
| 16954641 | poly(rC) binding protein 4 | PCBP4 | 0,18 | 0,0258 | 0,13 | 0,2275 |
| 16996234 | phosphatidic acid phosphatase type 2A | PPAP2A | 0,18 | 0,0141 | 0,07 | 0,6256 |
| 16664731 | PRP38 pre-mRNA processing factor 38 (yeast) domain containing A | PRPF38A | 0,18 | 0,0455 | 0,18 | 0,3086 |
| 17088185 | PRP4 pre-mRNA processing factor 4 homolog (yeast) | PRPF4 | 0,18 | 0,0445 | 0,13 | 0,0651 |
| 16950622 | proline-rich transmembrane protein 3 | PRRT3 | 0,18 | 0,0036 | -0,02 | 0,9062 |
| 16942202 | PX domain containing serine/threonine kinase | PXK | 0,18 | 0,0170 | 0,18 | 0,0917 |
| 16674927 | ral guanine nucleotide dissociation stimulator-like 1 | RGL1 | 0,18 | 0,0301 | 0,16 | 0,2648 |
| 17025152 | serine active site containing 1 | SERAC1 | 0,18 | 0,0054 | 0,18 | 0,0860 |
| 16710020 | transforming, acidic coiled-coil containing protein 2 | TACC2 | 0,18 | 0,0104 | -0,06 | 0,3105 |
| 16937181 | THUMP domain containing 3 | THUMPD3 | 0,18 | 0,0392 | 0,17 | 0,1460 |
| 16828462 | transmembrane protein 170A | TMEM170A | 0,18 | 0,0140 | 0,07 | 0,6193 |
| 16945451 | thyrotropin-releasing hormone | TRH | 0,18 | 0,0029 | 0,05 | 0,3149 |
| 17038998 | tripartite motif containing 26 | TRIM26 | 0,18 | 0,0403 | 0,14 | 0,0895 |
| 16673056 | UDP-N-acteylglucosamine pyrophosphorylase 1 | UAP1 | 0,18 | 0,0306 | 0,01 | 0,5954 |
| 16811020 | alpha- and gamma-adaptin binding protein | AAGAB | 0,17 | 0,0179 | 0,01 | 0,8071 |
| 16898617 | AP2 associated kinase 1 | AAK1 | 0,17 | 0,0348 | 0,02 | 0,9067 |
| 17021478 | akirin 2 | AKIRIN2 | 0,17 | 0,0129 | 0,13 | 0,3917 |
| 17104313 | androgen receptor | AR | 0,17 | 0,0336 | 0,09 | 0,1642 |
| 16826718 | Bardet-Biedl syndrome 2 | BBS2 | 0,17 | 0,0166 | 0,20 | 0,1959 |
| 16968077 | cyclin G2 | CCNG2 | 0,17 | 0,0466 | -0,10 | 0,4680 |
| 17086167 | centrosomal protein 78kDa | CEP78 | 0,17 | 0,0077 | -0,06 | 0,5230 |
| 16912871 | charged multivesicular body protein 4B | CHMP4B | 0,17 | 0,0321 | 0,06 | 0,2460 |
| 16724922 | catenin (cadherin-associated protein), delta 1 \| thioredoxin-related transmembrane protein 2 \| TMX2-CTNND1 readthrough (non-protein coding) | CTNND1\| TMX2\| TMX2-CTNND1 | 0,17 | 0,0288 | 0,04 | 0,7196 |
| 16940705 | dystroglycan 1 (dystrophin-associated glycoprotein 1) | DAG1 | 0,17 | 0,0399 | 0,02 | 0,7841 |
| 16748502 | DEAD (Asp-Glu-Ala-Asp) box polypeptide 47 | DDX47 | 0,17 | 0,0163 | -0,06 | 0,2583 |
| 16960647 | DEAH (Asp-Glu-Ala-His) box polypeptide 36 | DHX36 | 0,17 | 0,0034 | 0,06 | 0,4611 |
| 16925239 | downstream neighbor of SON | DONSON | 0,17 | 0,0166 | 0,05 | 0,4583 |
| 17082362 | epiplakin 1 | EPPK1 | 0,17 | 0,0104 | 0,03 | 0,7897 |
| 16951085 | FGD5 antisense RNA 1 (non-protein coding) | FGD5-AS1 | 0,17 | 0,0248 | -0,02 | 0,6337 |
| 16906749 | HECT, C2 and WW domain containing E3 ubiquitin protein ligase 2 | HECW2 | 0,17 | 0,0417 | 0,23 | 0,1169 |
| 17005042 | jumonji, AT rich interactive domain 2 | JARID2 | 0,17 | 0,0378 | 0,09 | 0,2996 |
| 16882150 | potassium channel modulatory factor 1 | KCMF1 | 0,17 | 0,0271 | 0,10 | 0,1341 |
| 16928716 | kringle containing transmembrane protein 1 | KREMEN1 | 0,17 | 0,0167 | 0,09 | 0,3945 |
| 17078121 | lactamase, beta 2 | LACTB2 | 0,17 | 0,0180 | 0,10 | 0,3452 |
| 16844894 | leprecan-like 4 | LEPREL4 | 0,17 | 0,0042 | 0,17 | 0,5459 |
| 16688992 | lysophosphatidic acid receptor 3 | LPAR3 | 0,17 | 0,0297 | 0,11 | 0,0643 |
| 16913811 | lipin 3 | LPIN3 | 0,17 | 0,0129 | 0,09 | 0,4402 |
| 16677698 | mitochondrial amidoxime reducing component 1 | MARC1 | 0,17 | 0,0062 | -0,03 | 0,7575 |
| 16947061 | muscleblind-like splicing regulator 1 | MBNL1 | 0,17 | 0,0195 | 0,16 | 0,2313 |
| 16822801 | NME/NM23 nucleoside diphosphate kinase 3 | NME3 | 0,17 | 0,0289 | 0,17 | 0,3922 |
| 16976201 | nitric oxide associated 1 | NOA1 | 0,17 | 0,0358 | 0,19 | 0,0656 |
| 16872460 | numb homolog (Drosophila)-like \| uncharacterized LOC100130713 | NUMBL\| LOC100130713 | 0,17 | 0,0112 | 0,10 | 0,4605 |
| 16894402 | ornithine decarboxylase 1 | ODC1 | 0,17 | 0,0016 | 0,10 | 0,1983 |
| 16703242 | OTU domain containing 1 | OTUD1 | 0,17 | 0,0200 | 0,12 | 0,1972 |
| 17001005 | protocadherin 1 | PCDH1 | 0,17 | 0,0388 | 0,01 | 0,4997 |
| 16988686 | phosphorylated adaptor for RNA export | PHAX | 0,17 | 0,0043 | 0,15 | 0,1804 |
| 16686557 | phosphoinositide-3-kinase, regulatory subunit 3 (gamma) \| uncharacterized LOC100507408 | PIK3R3\| LOC100507408 | 0,17 | 0,0303 | 0,08 | 0,1069 |
| 16913761 | phospholipase C, gamma 1 | PLCG1 | 0,17 | 0,0374 | 0,07 | 0,1658 |
| 16793721 | protein phosphatase 2, regulatory subunit B', epsilon isoform | PPP2R5E | 0,17 | 0,0393 | 0,03 | 0,3236 |
| 16867432 | protein tyrosine phosphatase, receptor type, S | PTPRS | 0,17 | 0,0247 | 0,20 | 0,2151 |
| 17091356 | RAB, member RAS oncogene family-like 6 | RABL6 | 0,17 | 0,0074 | 0,01 | 0,9519 |
| 17117588 | RAN binding protein 1 \| RAN binding protein 1 pseudogene | RANBP1\| LOC727803 | 0,17 | 0,0144 | 0,13 | 0,1760 |
| 16747991 | ribosomal modification protein rimK-like family member B | RIMKLB | 0,17 | 0,0185 | 0,07 | 0,4571 |
| 17086353 |  | RMI1 | 0,17 | 0,0495 | 0,10 | 0,6074 |
| 16667385 | RWD domain containing 3 \| TMEM56-RWDD3 readthrough \| transmembrane protein 56 | RWDD3\| TMEM56-RWDD3 | 0,17 | 0,0197 | 0,10 | 0,2533 |
| 16965719 | stromal interaction molecule 2 | STIM2 | 0,17 | 0,0126 | -0,08 | 0,3708 |
| 16743328 | TATA box binding protein (TBP)-associated factor, RNA polymerase I, D, 41kDa \| microRNA 1304 \| small nucleolar RNA, C/D box 5 \| small nucleolar RNA, H/ACA box 32 \| small nucleolar RNA, H/ACA box 40 \| small nucleolar RNA, H/ACA box 18 \| small nucleolar RNA, H/ACA box 1 \| small nucleolar RNA, H/ACA box 8 | TAF1D\| MIR1304\| SNORD5\| SNORA32\| SNORA40\| SNORA18\| SNORA8 | 0,17 | 0,0237 | 0,15 | 0,2140 |
| 16853042 | translocase of inner mitochondrial membrane 21 homolog (yeast) | TIMM21 | 0,17 | 0,0374 | 0,11 | 0,1824 |
| 16750901 | transmembrane BAX inhibitor motif containing 6 | TMBIM6 | 0,17 | 0,0302 | 0,10 | 0,3990 |
| 17046702 | transmembrane protein 248 | TMEM248 | 0,17 | 0,0145 | -0,03 | 0,5093 |
| 16672754 | ubiquitin specific peptidase 21 | USP21 | 0,17 | 0,0387 | 0,08 | 0,2779 |
| 16662692 | UTP11-like, U3 small nucleolar ribonucleoprotein, (yeast) | UTP11L | 0,17 | 0,0330 | 0,13 | 0,0518 |
| 16876050 | zinc finger protein 418 | ZNF418 | 0,17 | 0,0008 | 0,03 | 0,8793 |
| 17068266 | ADAM metallopeptidase domain 18 | ADAM18 | 0,16 | 0,0085 | 0,14 | 0,4584 |
| 17045806 | adenylate cyclase 1 (brain) | ADCY1 | 0,16 | 0,0262 | 0,15 | 0,1457 |
| 17011671 | adenosylmethionine decarboxylase 1 | AMD1 | 0,16 | 0,0148 | 0,06 | 0,5847 |
| 16780867 | arginine and glutamate rich 1 | ARGLU1 | 0,16 | 0,0084 | 0,09 | 0,4175 |
| 16702311 | ATP synthase, H+ transporting, mitochondrial F1 complex, gamma polypeptide 1 | ATP5C1 | 0,16 | 0,0470 | 0,08 | 0,6924 |
| 16854540 | UDP-Gal:betaGlcNAc beta 1,4- galactosyltransferase, polypeptide 6 | B4GALT6 | 0,16 | 0,0186 | 0,07 | 0,3960 |
| 16901755 | budding uninhibited by benzimidazoles 1 homolog (yeast) | BUB1 | 0,16 | 0,0047 | 0,04 | 0,6162 |
| 16710271 | budding uninhibited by benzimidazoles 3 homolog (yeast) | BUB3 | 0,16 | 0,0112 | 0,17 | 0,2204 |
| 16984336 | chromosome 5 open reading frame 51 | C5orf51 | 0,16 | 0,0297 | 0,27 | 0,1763 |
| 16658479 | calmodulin binding transcription activator 1 | CAMTA1 | 0,16 | 0,0023 | 0,10 | 0,1218 |
| 16776493 | carbohydrate kinase domain containing | CARKD | 0,16 | 0,0009 | -0,04 | 0,6369 |
| 16846928 | coilin | COIL | 0,16 | 0,0238 | 0,05 | 0,6092 |
| 16741287 | carnitine palmitoyltransferase 1A (liver) | CPT1A | 0,16 | 0,0153 | 0,11 | 0,4010 |
| 16753047 | deltex homolog 3 (Drosophila) | DTX3 | 0,16 | 0,0148 | -0,03 | 0,5945 |
| 16776784 | coagulation factor VII (serum prothrombin conversion accelerator) | F7 | 0,16 | 0,0363 | 0,14 | 0,0625 |
| 17026529 | gamma-aminobutyric acid (GABA) B receptor, 1 | GABBR1 | 0,16 | 0,0467 | 0,32 | 0,0677 |
| 16991018 | HMG box domain containing 3 | HMGXB3 | 0,16 | 0,0210 | 0,02 | 0,8704 |
| 16895208 | intersectin 2 | ITSN2 | 0,16 | 0,0278 | 0,13 | 0,3086 |
| 16923666 | keratin associated protein 10-11 | KRTAP10-11 | 0,16 | 0,0435 | 0,19 | 0,2688 |
| 16836072 | LUC7-like 3 (S. cerevisiae) | LUC7L3 | 0,16 | 0,0114 | 0,24 | 0,0536 |
| 16840408 | mediator complex subunit 31 | MED31 | 0,16 | 0,0147 | 0,17 | 0,1374 |
| 16914888 | molybdenum cofactor synthesis 3 | MOCS3 | 0,16 | 0,0127 | -0,01 | 0,8928 |
| 17015889 | nucleoporin 153kDa | NUP153 | 0,16 | 0,0362 | -0,01 | 0,9021 |
| 17098127 | phosducin-like | PDCL | 0,16 | 0,0453 | 0,13 | 0,2810 |
| 16692135 | polymerase (RNA) III (DNA directed) polypeptide C (62kD) | POLR3C | 0,16 | 0,0294 | 0,18 | 0,1718 |
| 16728336 | protein tyrosine phosphatase, receptor type, f polypeptide (PTPRF), interacting protein (liprin), alpha 1 | PPFIA1 | 0,16 | 0,0141 | 0,02 | 0,6031 |
| 16980005 | SET domain containing (lysine methyltransferase) 7 | SETD7 | 0,16 | 0,0176 | 0,15 | 0,0832 |
| 16953906 | ubiquitin specific peptidase 19 | USP19 | 0,16 | 0,0078 | -0,04 | 0,7449 |
| 16904024 | WD repeat, sterile alpha motif and U-box domain containing 1 | WDSUB1 | 0,16 | 0,0193 | 0,31 | 0,1775 |
| 16813372 | vacuolar protein sorting 33 homolog B (yeast) | VPS33B | 0,16 | 0,0327 | -0,01 | 0,9230 |
| 17069412 | YTH domain family, member 3 | YTHDF3 | 0,16 | 0,0492 | -0,05 | 0,7504 |
| 16748939 | AE binding protein 2 | AEBP2 | 0,15 | 0,0154 | 0,09 | 0,1736 |
| 16710017 | arginyltransferase 1 | ATE1 | 0,15 | 0,0202 | 0,08 | 0,3696 |
| 16937309 | bromodomain and PHD finger containing, 1 | BRPF1 | 0,15 | 0,0093 | 0,00 | 0,9398 |
| 16958303 | coiled-coil domain containing 14 | CCDC14 | 0,15 | 0,0473 | 0,03 | 0,8121 |
| 17066845 | charged multivesicular body protein 7 | CHMP7 | 0,15 | 0,0253 | 0,15 | 0,1432 |
| 16969344 | CDGSH iron sulfur domain 2 | CISD2 | 0,15 | 0,0308 | 0,17 | 0,1129 |
| 16956661 | claudin domain containing 1 | CLDND1 | 0,15 | 0,0363 | 0,20 | 0,2072 |
| 16986983 | cytochrome c oxidase subunit VIIc | COX7C | 0,15 | 0,0223 | 0,15 | 0,1192 |
| 16673842 | dynamin 3 | DNM3 | 0,15 | 0,0477 | -0,09 | 0,0558 |
| 16690769 | DNA-damage regulated autophagy modulator 2 | DRAM2 | 0,15 | 0,0376 | 0,10 | 0,3171 |
| 16839106 | fructosamine 3 kinase related protein | FN3KRP | 0,15 | 0,0098 | 0,19 | 0,0995 |
| 16706712 | growth hormone inducible transmembrane protein | GHITM | 0,15 | 0,0381 | 0,12 | 0,2517 |
| 17050765 | potassium voltage-gated channel, Shal-related subfamily, member 2 | KCND2 | 0,15 | 0,0339 | 0,04 | 0,8690 |
| 16661956 | karyopherin alpha 6 (importin alpha 7) \| uncharacterized LOC100653335 \| uncharacterized LOC100652828 | KPNA6\| LOC100653335\| LOC100652828 | 0,15 | 0,0431 | 0,11 | 0,0973 |
| 16901699 | LIM and senescent cell antigen-like domains 3 \| LIM and senescent cell antigen-like domains 3-like \| LIM and senescent cell antigen-like domains 1 \| LIMS3-LOC440895 readthrough | LIMS3\| LIMS3L\| LIMS1\| LIMS3-LOC440895 | 0,15 | 0,0477 | 0,01 | 0,8315 |
| 16777651 | ligand of numb-protein X 2 | LNX2 | 0,15 | 0,0202 | 0,09 | 0,5302 |
| 16834327 | N-acetylglucosaminidase, alpha | NAGLU | 0,15 | 0,0396 | 0,12 | 0,3817 |
| 16810104 | NMDA receptor regulated 2 | NARG2 | 0,15 | 0,0091 | 0,13 | 0,0513 |
| 16812824 | neuromedin B | NMB | 0,15 | 0,0024 | 0,04 | 0,8410 |
| 16951883 | oxysterol binding protein-like 10 | OSBPL10 | 0,15 | 0,0222 | 0,06 | 0,6091 |
| 16872203 | Paf1, RNA polymerase II associated factor, homolog (S. cerevisiae) | PAF1 | 0,15 | 0,0266 | 0,09 | 0,1049 |
| 16763467 | pleckstrin homology domain containing, family A member 8 pseudogene 1 | PLEKHA8P1 | 0,15 | 0,0460 | 0,20 | 0,2343 |
| 16661477 | protein phosphatase 1, regulatory subunit 8 | PPP1R8 | 0,15 | 0,0013 | 0,01 | 0,7015 |
| 17095194 | RAS and EF-hand domain containing | RASEF | 0,15 | 0,0281 | 0,08 | 0,1447 |
| 17049676 | serpin peptidase inhibitor, clade E (nexin, plasminogen activator inhibitor type 1), member 1 | SERPINE1 | 0,15 | 0,0071 | -0,18 | 0,1866 |
| 17106574 | solute carrier family 25 (mitochondrial carrier; adenine nucleotide translocator), member 5 | SLC25A5 | 0,15 | 0,0161 | 0,05 | 0,1737 |
| 16698980 | solute carrier family 30 (zinc transporter), member 1 | SLC30A1 | 0,15 | 0,0479 | 0,00 | 0,9739 |
| 16920100 | staufen, RNA binding protein, homolog 1 (Drosophila) | STAU1 | 0,15 | 0,0498 | 0,12 | 0,1398 |
| 17013567 | TGF-beta activated kinase 1/MAP3K7 binding protein 2 | TAB2 | 0,15 | 0,0208 | 0,06 | 0,1543 |
| 16743338 | TATA box binding protein (TBP)-associated factor, RNA polymerase I, D, 41kDa \| microRNA 1304 \| small nucleolar RNA, C/D box 5 \| small nucleolar RNA, H/ACA box 32 \| small nucleolar RNA, H/ACA box 40 \| small nucleolar RNA, H/ACA box 18 \| small nucleolar RNA, H/ACA box 1 \| small nucleolar RNA, H/ACA box 8 | TAF1D\| MIR1304\| SNORD5\| SNORA32\| SNORA40\| SNORA18\| SNORA8 | 0,15 | 0,0119 | 0,27 | 0,1628 |
| 16921123 | transcription factor-like 5 (basic helix-loop-helix) \| DPH3, KTI11 homolog (S. cerevisiae) pseudogene 1 | TCFL5\| DPH3P1 | 0,15 | 0,0302 | 0,03 | 0,8211 |
| 17009545 | transcription factor AP-2 beta (activating enhancer binding protein 2 beta) | TFAP2B | 0,15 | 0,0404 | 0,09 | 0,0585 |
| 16793460 | translocase of inner mitochondrial membrane 9 homolog (yeast) | TIMM9 | 0,15 | 0,0295 | 0,16 | 0,4485 |
| 16947287 | TCDD-inducible poly(ADP-ribose) polymerase | TIPARP | 0,15 | 0,0430 | 0,15 | 0,2235 |
| 17000650 | transmembrane protein 173 | TMEM173 | 0,15 | 0,0460 | -0,12 | 0,0942 |
| 16660919 | transmembrane protein 57 | TMEM57 | 0,15 | 0,0228 | 0,05 | 0,2680 |
| 16659737 | transmembrane protein 82 | TMEM82 | 0,15 | 0,0316 | 0,05 | 0,4310 |
| 17026623 | tripartite motif containing 26 | TRIM26 | 0,15 | 0,0037 | 0,08 | 0,2483 |
| 17103993 |  | TSR2 | 0,15 | 0,0285 | 0,10 | 0,2785 |
| 16976583 | UDP glucuronosyltransferase 2 family, polypeptide A1, complex locus \| UDP glucuronosyltransferase 2 family, polypeptide A2 | UGT2A1\| UGT2A2 | 0,15 | 0,0371 | 0,17 | 0,1669 |
| 16850264 | WDR45-like | WDR45L | 0,15 | 0,0289 | -0,14 | 0,2435 |
| 16871124 | ankyrin repeat domain 27 (VPS9 domain) | ANKRD27 | 0,14 | 0,0043 | -0,08 | 0,3148 |
| 16975419 | amyloid beta (A4) precursor protein-binding, family B, member 2 | APBB2 | 0,14 | 0,0285 | 0,00 | 0,9718 |
| 17011470 | armadillo repeat containing 2 | ARMC2 | 0,14 | 0,0028 | 0,14 | 0,2618 |
| 16791781 | chromosome 14 open reading frame 126 | C14orf126 | 0,14 | 0,0004 | 0,04 | 0,7626 |
| 17099731 | chromosome 9 open reading frame 116 | C9orf116 | 0,14 | 0,0495 | 0,09 | 0,1853 |
| 16667206 | coiled-coil domain containing 18 | CCDC18 | 0,14 | 0,0022 | -0,01 | 0,8533 |
| 16940669 | coiled-coil domain containing 36 \| uncharacterized LOC100131840 | CCDC36\| LOC100131840 | 0,14 | 0,0035 | 0,05 | 0,6396 |
| 17110463 | complement factor properdin | CFP | 0,14 | 0,0063 | 0,11 | 0,2581 |
| 16780929 | collagen, type IV, alpha 1 | COL4A1 | 0,14 | 0,0008 | 0,10 | 0,0933 |
| 16981266 | DEAD (Asp-Glu-Ala-Asp) box polypeptide 60-like | DDX60L | 0,14 | 0,0272 | 0,14 | 0,3240 |
| 16883426 | eukaryotic translation initiation factor 5B | EIF5B | 0,14 | 0,0026 | 0,17 | 0,1756 |
| 16968378 | enolase-phosphatase 1 | ENOPH1 | 0,14 | 0,0168 | 0,19 | 0,0702 |
| 17066446 | erythrocyte membrane protein band 4.9 (dematin) | EPB49 | 0,14 | 0,0378 | 0,15 | 0,1479 |
| 16752397 | v-erb-b2 erythroblastic leukemia viral oncogene homolog 3 (avian) | ERBB3 | 0,14 | 0,0270 | 0,15 | 0,1690 |
| 16937505 | Fanconi anemia, complementation group D2 | FANCD2 | 0,14 | 0,0066 | 0,05 | 0,6463 |
| 17033830 | gamma-aminobutyric acid (GABA) B receptor, 1 | GABBR1 | 0,14 | 0,0138 | 0,28 | 0,0788 |
| 16710635 | glutaredoxin 3 | GLRX3 | 0,14 | 0,0181 | -0,01 | 0,6836 |
| 16976655 | G-rich RNA sequence binding factor 1 | GRSF1 | 0,14 | 0,0083 | 0,10 | 0,5390 |
| 16931384 | G-2 and S-phase expressed 1 | GTSE1 | 0,14 | 0,0470 | 0,11 | 0,1997 |
| 16803436 | isocitrate dehydrogenase 3 (NAD+) alpha | IDH3A | 0,14 | 0,0079 | 0,13 | 0,1860 |
| 16999776 | interferon regulatory factor 1 | IRF1 | 0,14 | 0,0484 | -0,03 | 0,1401 |
| 16965313 | leucine aminopeptidase 3 | LAP3 | 0,14 | 0,0312 | 0,13 | 0,0971 |
| 16966304 | LIM and calponin homology domains 1 | LIMCH1 | 0,14 | 0,0268 | -0,01 | 0,9185 |
| 16728066 | low density lipoprotein receptor-related protein 5 | LRP5 | 0,14 | 0,0329 | -0,03 | 0,6811 |
| 16847485 | mediator complex subunit 13 | MED13 | 0,14 | 0,0019 | 0,04 | 0,2327 |
| 16764160 | myeloid/lymphoid or mixed-lineage leukemia 2 | MLL2 | 0,14 | 0,0051 | 0,02 | 0,8686 |
| 16914395 | matrix metallopeptidase 9 (gelatinase B, 92kDa gelatinase, 92kDa type IV collagenase) | MMP9 | 0,14 | 0,0416 | 0,11 | 0,2946 |
| 16899096 | MOB kinase activator 1A | MOB1A | 0,14 | 0,0390 | 0,00 | 0,9826 |
| 17111656 | myotubularin related protein 8 \| ankyrin repeat and SOCS box containing 12 | MTMR8\| ASB12 | 0,14 | 0,0059 | 0,00 | 0,9471 |
| 17061189 | N-acyl phosphatidylethanolamine phospholipase D | NAPEPLD | 0,14 | 0,0011 | 0,01 | 0,8901 |
| 17064939 | non-SMC condensin II complex, subunit G2 | NCAPG2 | 0,14 | 0,0197 | 0,04 | 0,1878 |
| 16820367 | nuclear factor of activated T-cells, cytoplasmic, calcineurin-dependent 3 | NFATC3 | 0,14 | 0,0008 | 0,09 | 0,1115 |
| 16915635 | oxysterol binding protein-like 2 | OSBPL2 | 0,14 | 0,0382 | 0,11 | 0,3301 |
| 16731589 | platelet-activating factor acetylhydrolase 1b, catalytic subunit 2 (30kDa) | PAFAH1B2 | 0,14 | 0,0147 | -0,01 | 0,9034 |
| 17096091 | patched 1 | PTCH1 | 0,14 | 0,0167 | 0,03 | 0,1139 |
| 16668550 | RNA binding motif protein 15 | RBM15 | 0,14 | 0,0055 | 0,06 | 0,6245 |
| 16790529 | RNA binding motif protein 23 | RBM23 | 0,14 | 0,0448 | 0,04 | 0,8163 |
| 16809587 | ribosomal L24 domain containing 1 | RSL24D1 | 0,14 | 0,0089 | 0,00 | 0,9993 |
| 17099947 | serologically defined colon cancer antigen 3 | SDCCAG3 | 0,14 | 0,0314 | 0,17 | 0,4469 |
| 16848793 | solute carrier family 25 (mitochondrial thiamine pyrophosphate carrier), member 19 | SLC25A19 | 0,14 | 0,0185 | 0,09 | 0,6138 |
| 16916104 | SLC2A4 regulator | SLC2A4RG | 0,14 | 0,0189 | 0,20 | 0,1740 |
| 16777777 | solute carrier family 7 (cationic amino acid transporter, y+ system), member 1 | SLC7A1 | 0,14 | 0,0306 | 0,00 | 0,9429 |
| 17077222 | transcription elongation factor A (SII), 1 \| transcription elongation factor A (SII), 1 pseudogene 2 | TCEA1\| TCEA1P2 | 0,14 | 0,0233 | 0,14 | 0,0586 |
| 17105543 | transcription elongation factor A (SII)-like 2 | TCEAL2 | 0,14 | 0,0307 | 0,00 | 0,9915 |
| 17043702 | transmembrane protein 106B | TMEM106B | 0,14 | 0,0320 | 0,22 | 0,1240 |
| 16893773 | transmembrane protein 18 | TMEM18 | 0,14 | 0,0440 | 0,01 | 0,8834 |
| 16859253 | tropomyosin 4 | TPM4 | 0,14 | 0,0397 | 0,05 | 0,4071 |
| 16848961 | tripartite motif containing 65 | TRIM65 | 0,14 | 0,0432 | 0,01 | 0,7651 |
| 16811697 | unc-51-like kinase 3 (C. elegans) | ULK3 | 0,14 | 0,0036 | 0,32 | 0,1102 |
| 16972553 | WD repeat domain 17 | WDR17 | 0,14 | 0,0042 | 0,13 | 0,3664 |
| 16846976 | vascular endothelial zinc finger 1 | VEZF1 | 0,14 | 0,0129 | 0,05 | 0,7076 |
| 16964676 | Wolfram syndrome 1 (wolframin) | WFS1 | 0,14 | 0,0347 | 0,04 | 0,6518 |
| 16746591 | WNK lysine deficient protein kinase 1 | WNK1 | 0,14 | 0,0134 | 0,06 | 0,2961 |
| 16777224 | exportin 4 | XPO4 | 0,14 | 0,0023 | -0,05 | 0,5472 |
| 16932809 | YdjC homolog (bacterial) | YDJC | 0,14 | 0,0202 | 0,09 | 0,3001 |
| 16741556 | zinc finger protein 705E | ZNF705E | 0,14 | 0,0103 | 0,00 | 0,9888 |
| 17049702 | zinc finger, HIT-type containing 1 | ZNHIT1 | 0,14 | 0,0003 | 0,11 | 0,2425 |
| 17044773 | zinc and ring finger 2 | ZNRF2 | 0,14 | 0,0029 | 0,15 | 0,3666 |
| 17090373 | c-abl oncogene 1, non-receptor tyrosine kinase | ABL1 | 0,13 | 0,0370 | 0,13 | 0,3136 |
| 16900628 | ankyrin repeat domain 39 \| ankyrin repeat domain 23 | ANKRD39\| ANKRD23 | 0,13 | 0,0363 | -0,01 | 0,9404 |
| 16914628 | ADP-ribosylation factor guanine nucleotide-exchange factor 2 (brefeldin A-inhibited) | ARFGEF2 | 0,13 | 0,0214 | 0,09 | 0,0723 |
| 17060143 | BAI1-associated protein 2-like 1 | BAIAP2L1 | 0,13 | 0,0428 | 0,02 | 0,8240 |
| 16722385 | chromosome 11 open reading frame 58 | C11orf58 | 0,13 | 0,0183 | 0,14 | 0,1590 |
| 16831102 | chromosome 17 open reading frame 48 | C17orf48 | 0,13 | 0,0446 | -0,14 | 0,1300 |
| 16871180 | centrosomal protein 89kDa | CEP89 | 0,13 | 0,0344 | 0,04 | 0,8368 |
| 16737783 | cytoskeleton associated protein 5 | CKAP5 | 0,13 | 0,0094 | -0,05 | 0,4643 |
| 16914704 | DEAD (Asp-Glu-Ala-Asp) box polypeptide 27 \| synovial sarcoma translocation, chromosome 18 | DDX27\| SS18 | 0,13 | 0,0102 | 0,02 | 0,8018 |
| 16869653 | DnaJ (Hsp40) homolog, subfamily B, member 1 | DNAJB1 | 0,13 | 0,0044 | -0,09 | 0,2899 |
| 16757902 | dynein, light chain, LC8-type 1 | DYNLL1 | 0,13 | 0,0055 | 0,14 | 0,1444 |
| 16689371 | guanylate binding protein 7 \| guanylate binding protein 2, interferon-inducible | GBP7\| GBP2 | 0,13 | 0,0084 | 0,21 | 0,1020 |
| 16887014 | grancalcin, EF-hand calcium binding protein | GCA | 0,13 | 0,0050 | 0,30 | 0,3206 |
| 16688323 | guanine nucleotide binding protein (G protein), gamma 12 | GNG12 | 0,13 | 0,0186 | 0,13 | 0,3223 |
| 17114272 | glypican 4 | GPC4 | 0,13 | 0,0075 | 0,01 | 0,9448 |
| 17000485 | heat shock 70kDa protein 9 (mortalin) | HSPA9 | 0,13 | 0,0162 | 0,09 | 0,0766 |
| 16719159 | IKAROS family zinc finger 5 (Pegasus) | IKZF5 | 0,13 | 0,0090 | 0,00 | 0,9947 |
| 17113286 | KCNE1-like | KCNE1L | 0,13 | 0,0239 | 0,07 | 0,0789 |
| 16806920 | lysophosphatidylcholine acyltransferase 4 | LPCAT4 | 0,13 | 0,0052 | 0,02 | 0,8444 |
| 16728586 | leucine rich transmembrane and 0-methyltransferase domain containing | LRTOMT | 0,13 | 0,0111 | 0,04 | 0,5926 |
| 16987844 | mannosidase, alpha, class 2A, member 1 | MAN2A1 | 0,13 | 0,0210 | 0,11 | 0,5418 |
| 16913403 | mannosidase, beta A, lysosomal-like | MANBAL | 0,13 | 0,0094 | 0,04 | 0,6711 |
| 16821139 | MON1 homolog B (yeast) | MON1B | 0,13 | 0,0303 | -0,05 | 0,5906 |
| 16837205 | nucleolar protein 11 | NOL11 | 0,13 | 0,0477 | 0,17 | 0,0986 |
| 16846093 | oxysterol binding protein-like 7 | OSBPL7 | 0,13 | 0,0205 | 0,07 | 0,4041 |
| 17106214 | p21 protein (Cdc42/Rac)-activated kinase 3 | PAK3 | 0,13 | 0,0204 | 0,17 | 0,2686 |
| 16726790 | polymerase (DNA directed), alpha 2, accessory subunit | POLA2 | 0,13 | 0,0448 | -0,05 | 0,6841 |
| 17091090 | protein phosphatase 1, regulatory subunit 26 | PPP1R26 | 0,13 | 0,0013 | 0,15 | 0,2351 |
| 16984287 | prostaglandin E receptor 4 (subtype EP4) | PTGER4 | 0,13 | 0,0235 | 0,09 | 0,4448 |
| 17059355 | sema domain, immunoglobulin domain (Ig), short basic domain, secreted, (semaphorin) 3D | SEMA3D | 0,13 | 0,0206 | 0,04 | 0,4280 |
| 16765491 | single-strand-selective monofunctional uracil-DNA glycosylase 1 | SMUG1 | 0,13 | 0,0025 | 0,00 | 0,9999 |
| 16658655 | splA/ryanodine receptor domain and SOCS box containing 1 | SPSB1 | 0,13 | 0,0483 | 0,09 | 0,3426 |
| 16736554 | SPT2, Suppressor of Ty, domain containing 1 (S. cerevisiae) | SPTY2D1 | 0,13 | 0,0308 | 0,11 | 0,0781 |
| 16816424 | synaptotagmin XVII | SYT17 | 0,13 | 0,0036 | 0,05 | 0,6260 |
| 16722189 | TEA domain family member 1 (SV40 transcriptional enhancer factor) | TEAD1 | 0,13 | 0,0309 | -0,11 | 0,3318 |
| 16781958 | T cell receptor alpha variable 30 | TRAV30 | 0,13 | 0,0016 | -0,10 | 0,2616 |
| 17036239 | tripartite motif containing 26 | TRIM26 | 0,13 | 0,0413 | 0,05 | 0,4842 |
| 16917030 | tRNA methyltransferase 6 homolog (S. cerevisiae) | TRMT6 | 0,13 | 0,0400 | 0,16 | 0,0919 |
| 16674953 | tRNA splicing endonuclease 15 homolog (S. cerevisiae) | TSEN15 | 0,13 | 0,0344 | 0,13 | 0,0540 |
| 16877637 | UBX domain protein 2A | UBXN2A | 0,13 | 0,0317 | 0,11 | 0,0778 |
| 16690343 | vav 3 guanine nucleotide exchange factor | VAV3 | 0,13 | 0,0016 | 0,04 | 0,1929 |
| 16814389 | WD repeat domain 90 | WDR90 | 0,13 | 0,0021 | 0,03 | 0,7788 |
| 16927222 | zinc finger, DHHC-type containing 8 | ZDHHC8 | 0,13 | 0,0390 | 0,13 | 0,4158 |
| 16862962 | zinc finger protein 227 | ZNF227 | 0,13 | 0,0277 | 0,09 | 0,2169 |
| 16758885 | acetoacetyl-CoA synthetase | AACS | 0,12 | 0,0370 | 0,07 | 0,4122 |
| 17008544 | bystin-like | BYSL | 0,12 | 0,0091 | 0,14 | 0,0889 |
| 16751769 | chromosome 12 open reading frame 10 | C12orf10 | 0,12 | 0,0003 | 0,14 | 0,2210 |
| 16847949 | centrosomal protein 112kDa | CEP112 | 0,12 | 0,0208 | 0,12 | 0,2460 |
| 16929203 | developmentally regulated GTP binding protein 1 | DRG1 | 0,12 | 0,0274 | 0,09 | 0,1182 |
| 17118160 |  | FLJ38717 | 0,12 | 0,0297 | -0,01 | 0,8713 |
| 17043157 | forkhead box K1 | FOXK1 | 0,12 | 0,0366 | 0,03 | 0,8061 |
| 16814895 | growth factor, augmenter of liver regeneration | GFER | 0,12 | 0,0379 | 0,05 | 0,7846 |
| 16777896 | heat shock 105kDa/110kDa protein 1 | HSPH1 | 0,12 | 0,0298 | 0,08 | 0,2277 |
| 16984646 | integrin, alpha 1 \| pelota homolog (Drosophila) | ITGA1\| PELO | 0,12 | 0,0462 | 0,09 | 0,4760 |
| 16899263 | mitochondrial ribosomal protein L53 \| coiled-coil domain containing 142 | MRPL53\| CCDC142 | 0,12 | 0,0046 | 0,05 | 0,2675 |
| 16704865 | microseminoprotein, beta- | MSMB | 0,12 | 0,0075 | 0,02 | 0,9001 |
| 16966548 | NIPA-like domain containing 1 | NIPAL1 | 0,12 | 0,0132 | 0,07 | 0,6915 |
| 16911688 | PET117 homolog (S. cerevisiae) \| CSRP2 binding protein | PET117\| CSRP2BP | 0,12 | 0,0243 | 0,18 | 0,1063 |
| 16959007 | plexin D1 | PLXND1 | 0,12 | 0,0314 | -0,02 | 0,7105 |
| 16673767 | proline-rich coiled-coil 2C | PRRC2C | 0,12 | 0,0149 | 0,03 | 0,5264 |
| 16708097 | R3H domain and coiled-coil containing 1-like | R3HCC1L | 0,12 | 0,0420 | 0,06 | 0,6461 |
| 16890651 | ribosomal protein L37a | RPL37A | 0,12 | 0,0323 | 0,05 | 0,3016 |
| 16890915 | RCD1 required for cell differentiation1 homolog (S. pombe) | RQCD1 | 0,12 | 0,0465 | 0,06 | 0,2525 |
| 16742520 | remodeling and spacing factor 1 | RSF1 | 0,12 | 0,0309 | 0,17 | 0,1700 |
| 16873332 | reticulon 2 | RTN2 | 0,12 | 0,0084 | 0,26 | 0,2691 |
| 16919022 | SAM domain and HD domain 1 | SAMHD1 | 0,12 | 0,0476 | 0,09 | 0,1060 |
| 17101876 | sex comb on midleg-like 1 (Drosophila) | SCML1 | 0,12 | 0,0089 | 0,08 | 0,5521 |
| 16950686 |  | SEC13 | 0,12 | 0,0411 | 0,10 | 0,3668 |
| 17066650 | sorbin and SH3 domain containing 3 | SORBS3 | 0,12 | 0,0206 | 0,04 | 0,7517 |
| 16852550 | ST8 alpha-N-acetyl-neuraminide alpha-2,8-sialyltransferase 3 | ST8SIA3 | 0,12 | 0,0276 | 0,08 | 0,3810 |
| 17003815 | TBC1 domain family, member 9B (with GRAM domain) | TBC1D9B | 0,12 | 0,0028 | 0,02 | 0,9102 |
| 16770685 | T-box 3 | TBX3 | 0,12 | 0,0023 | -0,10 | 0,2628 |
| 16830412 | tyrosine kinase, non-receptor, 1 | TNK1 | 0,12 | 0,0342 | 0,10 | 0,2045 |
| 16664078 | target of EGR1, member 1 (nuclear) | TOE1 | 0,12 | 0,0298 | -0,06 | 0,6422 |
| 17041583 | tripartite motif containing 26 | TRIM26 | 0,12 | 0,0294 | 0,05 | 0,5122 |
| 16801995 | thyroid hormone receptor interactor 4 | TRIP4 | 0,12 | 0,0440 | 0,05 | 0,4731 |
| 16814636 | ubiquitin-conjugating enzyme E2I | UBE2I | 0,12 | 0,0227 | 0,02 | 0,6868 |
| 16858668 | WD repeat domain 83 | WDR83 | 0,12 | 0,0245 | 0,12 | 0,2927 |
| 16918496 | adenosylhomocysteinase | AHCY | 0,11 | 0,0409 | 0,01 | 0,9487 |
| 16869588 | ASF1 anti-silencing function 1 homolog B (S. cerevisiae) | ASF1B | 0,11 | 0,0084 | 0,04 | 0,6849 |
| 16739763 | atlastin GTPase 3 | ATL3 | 0,11 | 0,0203 | 0,01 | 0,9410 |
| 16931278 | ataxin 10 | ATXN10 | 0,11 | 0,0081 | 0,02 | 0,7764 |
| 17095936 | bicaudal D homolog 2 (Drosophila) | BICD2 | 0,11 | 0,0396 | 0,02 | 0,7902 |
| 16928127 | calcineurin binding protein 1 | CABIN1 | 0,11 | 0,0467 | 0,00 | 0,9560 |
| 16840170 | calmodulin binding transcription activator 2 | CAMTA2 | 0,11 | 0,0336 | 0,03 | 0,7616 |
| 16848489 | CDC42 effector protein (Rho GTPase binding) 4 | CDC42EP4 | 0,11 | 0,0002 | -0,10 | 0,5138 |
| 16859486 | DET1 and DDB1 associated 1 | DDA1 | 0,11 | 0,0190 | 0,05 | 0,1352 |
| 17055039 | KDEL (Lys-Asp-Glu-Leu) endoplasmic reticulum protein retention receptor 2 | KDELR2 | 0,11 | 0,0092 | 0,09 | 0,1851 |
| 16717176 |  | MMS19 | 0,11 | 0,0292 | 0,07 | 0,4864 |
| 16723507 | N-acetyltransferase 10 (GCN5-related) | NAT10 | 0,11 | 0,0206 | 0,06 | 0,5399 |
| 16708552 | nucleolar and coiled-body phosphoprotein 1 | NOLC1 | 0,11 | 0,0481 | 0,05 | 0,2890 |
| 16950932 | nucleoporin 210kDa | NUP210 | 0,11 | 0,0066 | -0,06 | 0,4468 |
| 16990205 | protocadherin alpha 11 \| protocadherin alpha 12 \| protocadherin alpha 1 \| protocadherin alpha 10 \| protocadherin alpha 2 \| protocadherin alpha 3 \| protocadherin alpha subfamily C, 1 \| protocadherin alpha subfamily C, 2 \| protocadherin alpha 5 \| protocadherin alpha 7 \| protocadherin alpha 4 \| protocadherin alpha 6 \| protocadherin alpha 13 \| protocadherin alpha 8 \| protocadherin alpha 9 | PCDHA11\| PCDHA12\| PCDHA10\| PCDHA2\| PCDHA3\| PCDHAC1\| PCDHAC2\| PCDHA5\| PCDHA7\| PCDHA4\| PCDHA6\| PCDHA13\| PCDHA8\| PCDHA9 | 0,11 | 0,0323 | 0,04 | 0,3345 |
| 16861193 | presenilin enhancer 2 homolog (C. elegans) | PSENEN | 0,11 | 0,0327 | -0,03 | 0,5461 |
| 16885978 | R3H domain containing 1 | R3HDM1 | 0,11 | 0,0140 | 0,06 | 0,6142 |
| 16697004 | ribonuclease L (2',5'-oligoisoadenylate synthetase-dependent) | RNASEL | 0,11 | 0,0221 | -0,08 | 0,4406 |
| 17059771 | sterile alpha motif domain containing 9 | SAMD9 | 0,11 | 0,0283 | -0,12 | 0,3581 |
| 16936535 | SET binding factor 1 | SBF1 | 0,11 | 0,0339 | 0,04 | 0,6754 |
| 16689042 | synapse defective 1, Rho GTPase, homolog 2 (C. elegans) | SYDE2 | 0,11 | 0,0391 | 0,01 | 0,9148 |
| 16960618 | transmembrane protein 14E | TMEM14E | 0,11 | 0,0032 | 0,01 | 0,8707 |
| 16762759 | transmembrane and tetratricopeptide repeat containing 1 | TMTC1 | 0,11 | 0,0114 | 0,21 | 0,2500 |
| 16827248 | TNFRSF1A-associated via death domain | TRADD | 0,11 | 0,0076 | 0,01 | 0,9384 |
| 16972773 | trafficking protein particle complex 11 | TRAPPC11 | 0,11 | 0,0151 | 0,02 | 0,8556 |
| 17056853 | T cell receptor gamma variable 2 | TRGV2 | 0,11 | 0,0038 | -0,01 | 0,9681 |
| 16736508 | tumor susceptibility gene 101 | TSG101 | 0,11 | 0,0389 | 0,08 | 0,3489 |
| 16875970 | zinc finger protein 550 | ZNF550 | 0,11 | 0,0427 | 0,00 | 0,9429 |
| 16864109 | aldehyde dehydrogenase 16 family, member A1 | ALDH16A1 | 0,1 | 0,0290 | 0,02 | 0,8426 |
| 16783974 | ADP-ribosylation factor 6 | ARF6 | 0,1 | 0,0375 | -0,06 | 0,1747 |
| 16739383 | beta-1,3-glucuronyltransferase 3 (glucuronosyltransferase I) | B3GAT3 | 0,1 | 0,0447 | -0,07 | 0,0663 |
| 16803185 | COMM domain containing 4 | COMMD4 | 0,1 | 0,0035 | 0,01 | 0,9247 |
| 16671305 | cAMP responsive element binding protein 3-like 4 | CREB3L4 | 0,1 | 0,0227 | 0,05 | 0,5434 |
| 16953187 | chondroitin sulfate proteoglycan 5 (neuroglycan C) | CSPG5 | 0,1 | 0,0438 | 0,02 | 0,8119 |
| 16840587 | CTD nuclear envelope phosphatase 1 | CTDNEP1 | 0,1 | 0,0348 | 0,13 | 0,3728 |
| 16825794 | dCTP pyrophosphatase 1 | DCTPP1 | 0,1 | 0,0210 | 0,05 | 0,4891 |
| 16739276 | eukaryotic translation elongation factor 1 gamma \| microRNA 3654 | EEF1G\| MIR3654 | 0,1 | 0,0046 | 0,09 | 0,2568 |
| 17054843 | fascin homolog 1, actin-bundling protein (Strongylocentrotus purpuratus) \| uncharacterized LOC100653350 | FSCN1\| LOC100653350 | 0,1 | 0,0344 | 0,04 | 0,7326 |
| 16917004 | glycerophosphocholine phosphodiesterase GDE1 homolog (S. cerevisiae) | GPCPD1 | 0,1 | 0,0277 | 0,02 | 0,8704 |
| 16992217 | Kv channel interacting protein 1 | KCNIP1 | 0,1 | 0,0251 | 0,05 | 0,8134 |
| 16934385 | myoglobin | MB | 0,1 | 0,0217 | 0,27 | 0,0707 |
| 16958382 | mucin 13, cell surface associated | MUC13 | 0,1 | 0,0063 | 0,07 | 0,1402 |
| 16823692 | N-acetylglucosamine-1-phosphodiester alpha-N-acetylglucosaminidase | NAGPA | 0,1 | 0,0498 | 0,19 | 0,2258 |
| 17108113 | plexin B3 \| SRSF protein kinase 3 | PLXNB3\| SRPK3 | 0,1 | 0,0270 | 0,05 | 0,6280 |
| 17113184 | proteasome (prosome, macropain) 26S subunit, non-ATPase, 10 | PSMD10 | 0,1 | 0,0152 | 0,14 | 0,0566 |
| 16834486 | proteasome (prosome, macropain) activator subunit 3 (PA28 gamma; Ki) | PSME3 | 0,1 | 0,0490 | 0,06 | 0,1582 |
| 16962493 | replication factor C (activator 1) 4, 37kDa | RFC4 | 0,1 | 0,0088 | 0,09 | 0,2987 |
| 16731205 | succinate dehydrogenase complex, subunit D, integral membrane protein | SDHD | 0,1 | 0,0316 | 0,16 | 0,1506 |
| 16828696 | short chain dehydrogenase/reductase family 42E, member 1 | SDR42E1 | 0,1 | 0,0262 | 0,15 | 0,0787 |
| 17089606 | solute carrier family 27 (fatty acid transporter), member 4 | SLC27A4 | 0,1 | 0,0091 | 0,03 | 0,7086 |
| 16870047 | solute carrier family 35, member E1 | SLC35E1 | 0,1 | 0,0445 | 0,10 | 0,0768 |
| 16674355 | sterol O-acyltransferase 1 | SOAT1 | 0,1 | 0,0021 | -0,06 | 0,5472 |
| 16895963 | suppressor of Ty 7 (S. cerevisiae)-like | SUPT7L | 0,1 | 0,0201 | -0,04 | 0,6337 |
| 16916946 | transmembrane protein 230 | TMEM230 | 0,1 | 0,0490 | 0,09 | 0,3196 |
| 16885432 | UDP-glucose glycoprotein glucosyltransferase 1 | UGGT1 | 0,1 | 0,0447 | 0,12 | 0,0961 |
| 16849528 | ubiquitin specific peptidase 36 | USP36 | 0,1 | 0,0090 | 0,03 | 0,1711 |
| 16921354 | zinc finger and BTB domain containing 46 | ZBTB46 | 0,1 | 0,0362 | -0,12 | 0,4189 |
| 16964532 | zinc finger and BTB domain containing 49 | ZBTB49 | 0,1 | 0,0230 | 0,03 | 0,6303 |
| 17017763 | advanced glycosylation end product-specific receptor \| pre-B-cell leukemia homeobox 2 \| pre-B-cell leukemia homeobox 2 pseudogene 1 | AGER\| PBX2\| PBX2P1 | 0,09 | 0,0479 | 0,10 | 0,2889 |
| 16969528 | aminoacyl tRNA synthetase complex-interacting multifunctional protein 1 | AIMP1 | 0,09 | 0,0353 | 0,05 | 0,5890 |
| 16674375 | axonemal dynein light chain domain containing 1 | AXDND1 | 0,09 | 0,0190 | 0,03 | 0,6451 |
| 16863074 | basal cell adhesion molecule (Lutheran blood group) | BCAM | 0,09 | 0,0048 | -0,14 | 0,3820 |
| 16945357 | chromosome 3 open reading frame 37 | C3orf37 | 0,09 | 0,0081 | -0,03 | 0,7967 |
| 17117110 |  | CD24 | 0,09 | 0,0184 | -0,04 | 0,6697 |
| 16784299 | cyclin-dependent kinase inhibitor 3 | CDKN3 | 0,09 | 0,0311 | 0,13 | 0,1113 |
| 16693601 | CREB regulated transcription coactivator 2 | CRTC2 | 0,09 | 0,0090 | -0,04 | 0,6962 |
| 16782576 | DDB1 and CUL4 associated factor 11 | DCAF11 | 0,09 | 0,0470 | 0,04 | 0,7946 |
| 16842027 | flightless I homolog (Drosophila) | FLII | 0,09 | 0,0105 | 0,01 | 0,9625 |
| 16886564 | formin-like 2 | FMNL2 | 0,09 | 0,0294 | 0,15 | 0,0577 |
| 16839086 | forkhead box K2 | FOXK2 | 0,09 | 0,0251 | 0,04 | 0,3611 |
| 16944512 | general transcription factor IIE, polypeptide 1, alpha 56kDa | GTF2E1 | 0,09 | 0,0363 | 0,19 | 0,0622 |
| 16787799 | interferon, alpha-inducible protein 27-like 1 | IFI27L1 | 0,09 | 0,0215 | 0,13 | 0,1305 |
| 16926813 | interleukin 17 receptor A | IL17RA | 0,09 | 0,0251 | -0,05 | 0,2952 |
| 16753853 | Mdm2, p53 E3 ubiquitin protein ligase homolog (mouse) | MDM2 | 0,09 | 0,0022 | 0,04 | 0,4932 |
| 16859395 | myosin IXB | MYO9B | 0,09 | 0,0332 | -0,08 | 0,4853 |
| 16918546 | nuclear receptor coactivator 6 | NCOA6 | 0,09 | 0,0226 | 0,01 | 0,5962 |
| 16672808 | NADH dehydrogenase (ubiquinone) Fe-S protein 2, 49kDa (NADH-coenzyme Q reductase) | NDUFS2 | 0,09 | 0,0181 | 0,08 | 0,0834 |
| 16660785 | NIPA-like domain containing 3 | NIPAL3 | 0,09 | 0,0299 | -0,02 | 0,8517 |
| 16811500 | neuroplastin | NPTN | 0,09 | 0,0270 | 0,03 | 0,6629 |
| 16719233 | ornithine aminotransferase | OAT | 0,09 | 0,0473 | 0,07 | 0,0760 |
| 17078415 | peroxisomal biogenesis factor 2 | PEX2 | 0,09 | 0,0132 | 0,08 | 0,2669 |
| 17110670 | pim-2 oncogene | PIM2 | 0,09 | 0,0481 | 0,05 | 0,3327 |
| 16735738 | ring finger protein 141 | RNF141 | 0,09 | 0,0127 | 0,01 | 0,7781 |
| 16708249 | stearoyl-CoA desaturase (delta-9-desaturase) | SCD | 0,09 | 0,0497 | 0,03 | 0,5844 |
| 16829570 | serpin peptidase inhibitor, clade F (alpha-2 antiplasmin, pigment epithelium derived factor), member 1 | SERPINF1 | 0,09 | 0,0062 | -0,06 | 0,7923 |
| 16701364 | SET and MYND domain containing 3 | SMYD3 | 0,09 | 0,0417 | 0,15 | 0,2519 |
| 17025230 | T-cell activation RhoGTPase activating protein | TAGAP | 0,09 | 0,0206 | 0,05 | 0,5827 |
| 16776052 | transmembrane 9 superfamily member 2 | TM9SF2 | 0,09 | 0,0404 | 0,06 | 0,2386 |
| 17021585 | ubiquitin-conjugating enzyme E2, J1 | UBE2J1 | 0,09 | 0,0472 | -0,05 | 0,7081 |
| 16821519 | ubiquitin specific peptidase 10 | USP10 | 0,09 | 0,0012 | 0,07 | 0,1723 |
| 16790202 | zinc finger protein 219 | ZNF219 | 0,09 | 0,0051 | 0,19 | 0,1700 |
| 16884797 | ARP3 actin-related protein 3 homolog (yeast) | ACTR3 | 0,08 | 0,0285 | 0,15 | 0,2292 |
| 16930299 | activating transcription factor 4 (tax-responsive enhancer element B67) | ATF4 | 0,08 | 0,0188 | -0,02 | 0,1138 |
| 16873534 | coiled-coil domain containing 8 | CCDC8 | 0,08 | 0,0474 | -0,07 | 0,5866 |
| 16705844 | cadherin-related 23 \| cadherin-23-like | CDH23\| LOC100653137 | 0,08 | 0,0437 | 0,15 | 0,1629 |
| 16826909 | CCR4-NOT transcription complex, subunit 1 | CNOT1 | 0,08 | 0,0200 | 0,03 | 0,4200 |
| 16666738 | cysteine-rich, angiogenic inducer, 61 | CYR61 | 0,08 | 0,0112 | -0,19 | 0,2027 |
| 16865752 | epsin 1 | EPN1 | 0,08 | 0,0306 | -0,05 | 0,6535 |
| 16968331 | fibroblast growth factor 5 | FGF5 | 0,08 | 0,0024 | 0,02 | 0,4175 |
| 17114288 | glypican 3 | GPC3 | 0,08 | 0,0432 | 0,06 | 0,2484 |
| 17112471 | highly divergent homeobox | HDX | 0,08 | 0,0207 | 0,18 | 0,1475 |
| 16941167 | mesencephalic astrocyte-derived neurotrophic factor | MANF | 0,08 | 0,0347 | 0,06 | 0,2096 |
| 17114478 | membrane magnesium transporter 1 | MMGT1 | 0,08 | 0,0155 | 0,00 | 0,9721 |
| 16708154 | NK2 homeobox 3 | NKX2-3 | 0,08 | 0,0101 | 0,04 | 0,5504 |
| 16831598 | 5',3'-nucleotidase, mitochondrial | NT5M | 0,08 | 0,0397 | 0,16 | 0,4439 |
| 16977217 | progestin and adipoQ receptor family member III | PAQR3 | 0,08 | 0,0463 | 0,03 | 0,4555 |
| 16681924 | PRAME family member 14 | PRAMEF14 | 0,08 | 0,0173 | 0,18 | 0,2592 |
| 16898989 | RAB11 family interacting protein 5 (class I) | RAB11FIP5 | 0,08 | 0,0015 | -0,10 | 0,3274 |
| 16801529 | ring finger protein 111 | RNF111 | 0,08 | 0,0228 | -0,02 | 0,8232 |
| 16696779 | SEC16 homolog B (S. cerevisiae) | SEC16B | 0,08 | 0,0282 | -0,03 | 0,7581 |
| 16696783 | SEC16 homolog B (S. cerevisiae) | SEC16B | 0,08 | 0,0011 | 0,01 | 0,8008 |
| 16950569 | transcriptional adaptor 3 | TADA3 | 0,08 | 0,0013 | 0,08 | 0,3829 |
| 16752233 | transmembrane protein 198B, pseudogene | TMEM198B | 0,08 | 0,0410 | 0,06 | 0,1190 |
| 16929056 | taurine upregulated 1 (non-protein coding) | TUG1 | 0,08 | 0,0455 | 0,03 | 0,6459 |
| 17101143 | acetylserotonin O-methyltransferase \| A kinase (PRKA) anchor protein 17A | ASMT\| AKAP17A | 0,07 | 0,0053 | 0,03 | 0,5112 |
| 16886558 | calcium channel, voltage-dependent, beta 4 subunit | CACNB4 | 0,07 | 0,0294 | 0,09 | 0,1587 |
| 16725556 | dihydroxyacetone kinase 2 homolog (S. cerevisiae) | DAK | 0,07 | 0,0342 | -0,04 | 0,8450 |
| 16847921 | guanine nucleotide binding protein (G protein), alpha 13 | GNA13 | 0,07 | 0,0483 | 0,09 | 0,3222 |
| 16816542 | IQ motif containing K | IQCK | 0,07 | 0,0244 | 0,00 | 0,9848 |
| 17056493 | kelch repeat and BTB (POZ) domain containing 2 | KBTBD2 | 0,07 | 0,0059 | 0,05 | 0,7180 |
| 16955939 | leucine-rich repeats and immunoglobulin-like domains 1 | LRIG1 | 0,07 | 0,0176 | 0,05 | 0,4719 |
| 16806640 | OTU domain containing 7A | OTUD7A | 0,07 | 0,0252 | 0,02 | 0,6472 |
| 16845158 | polymerase I and transcript release factor | PTRF | 0,07 | 0,0130 | 0,19 | 0,0921 |
| 17118394 | RAP1B, member of RAS oncogene family \| RAP1B, member of RAS oncogene family pseudogene | RAP1B\| LOC100506390 | 0,07 | 0,0169 | 0,13 | 0,1670 |
| 17061097 | RAS p21 protein activator 4 \| RAS p21 protein activator 4C, pseudogene \| RAS p21 protein activator 4B \| uroplakin 3B-like | RASA4\| RASA4CP\| RASA4B\| UPK3BL | 0,07 | 0,0476 | 0,24 | 0,1009 |
| 17008676 | ribosomal protein L7-like 1 | RPL7L1 | 0,07 | 0,0382 | 0,15 | 0,2157 |
| 17084669 | RUN and SH3 domain containing 2 | RUSC2 | 0,07 | 0,0321 | 0,15 | 0,3346 |
| 16707616 | solute carrier family 35, member G1 | SLC35G1 | 0,07 | 0,0167 | 0,11 | 0,5935 |
| 16823938 | thioredoxin domain containing 11 | TXNDC11 | 0,07 | 0,0289 | 0,02 | 0,7741 |
| 17074608 | XK, Kell blood group complex subunit-related family, member 6 | XKR6 | 0,07 | 0,0370 | -0,02 | 0,0945 |
| 16834621 | ADP-ribosylation factor-like 4D | ARL4D | 0,06 | 0,0055 | 0,18 | 0,2624 |
| 16890970 | BCS1-like (S. cerevisiae) | BCS1L | 0,06 | 0,0091 | 0,08 | 0,3365 |
| 16995938 | chromosome 5 open reading frame 34 | C5orf34 | 0,06 | 0,0346 | 0,06 | 0,0660 |
| 17013072 | headcase homolog (Drosophila) | HECA | 0,06 | 0,0251 | 0,04 | 0,3617 |
| 16927446 | leucine-zipper-like transcription regulator 1 | LZTR1 | 0,06 | 0,0056 | 0,06 | 0,7196 |
| 17071041 | NADH dehydrogenase (ubiquinone) complex I, assembly factor 6 \| uncharacterized LOC100506538 | NDUFAF6\| LOC100506538 | 0,06 | 0,0370 | 0,11 | 0,2208 |
| 16698348 | protein phosphatase 1, regulatory subunit 15B | PPP1R15B | 0,06 | 0,0381 | -0,05 | 0,1637 |
| 17050847 | protein tyrosine phosphatase, receptor-type, Z polypeptide 1 | PTPRZ1 | 0,06 | 0,0467 | 0,01 | 0,9053 |
| 17098103 | RNA binding motif protein 18 | RBM18 | 0,06 | 0,0171 | 0,17 | 0,1868 |
| 16977016 | SDA1 domain containing 1 | SDAD1 | 0,06 | 0,0096 | 0,04 | 0,3810 |
| 16680098 | stromal cell derived factor 4 | SDF4 | 0,06 | 0,0220 | 0,02 | 0,9295 |
| 17086871 | WNK lysine deficient protein kinase 2 | WNK2 | 0,06 | 0,0144 | 0,03 | 0,6186 |
| 16860398 | V-set and transmembrane domain containing 2B | VSTM2B | 0,06 | 0,0401 | -0,05 | 0,5504 |
| 16776537 | Rho guanine nucleotide exchange factor (GEF) 7 | ARHGEF7 | 0,05 | 0,0466 | -0,02 | 0,8771 |
| 17079293 | cyclin E2 | CCNE2 | 0,05 | 0,0448 | 0,11 | 0,3260 |
| 17082548 | diacylglycerol O-acyltransferase 1 | DGAT1 | 0,05 | 0,0338 | 0,06 | 0,5298 |
| 17020802 | eukaryotic translation elongation factor 1 alpha 1 \| uncharacterized LOC100653236 \| eukaryotic translation elongation factor 1 alpha 1 pseudogene 5 \| eukaryotic translation elongation factor 1 alpha 1 pseudogene 6 \| eukaryotic translation elongation factor 1 alpha 1 pseudogene 12 | EEF1A1\| LOC100653236\| EEF1A1P5\| EEF1A1P6\| EEF1A1P12 | 0,05 | 0,0145 | 0,11 | 0,1181 |
| 16922074 | keratin associated protein 22-1 | KRTAP22-1 | 0,05 | 0,0019 | 0,01 | 0,8486 |
| 16714170 | oxoglutarate dehydrogenase-like | OGDHL | 0,05 | 0,0312 | -0,10 | 0,4811 |
| 16895337 | proopiomelanocortin | POMC | 0,05 | 0,0114 | -0,06 | 0,4861 |
| 17010544 | SH3 domain binding glutamic acid-rich protein like 2 | SH3BGRL2 | 0,05 | 0,0142 | -0,09 | 0,1979 |
| 16754935 | transmembrane and tetratricopeptide repeat containing 3 | TMTC3 | 0,05 | 0,0438 | 0,05 | 0,3515 |
| 16815246 | zymogen granule protein 16 homolog B (rat) | ZG16B | 0,05 | 0,0407 | 0,12 | 0,1697 |
| 16947449 | major facilitator superfamily domain containing 1 \| cytokine receptor CRL2 | MFSD1\| LOC100287290 | 0,04 | 0,0489 | 0,04 | 0,4019 |
| 16829170 | piezo-type mechanosensitive ion channel component 1 | PIEZO1 | 0,04 | 0,0305 | -0,18 | 0,1792 |
| 17093871 | sperm associated antigen 8 | SPAG8 | 0,04 | 0,0054 | 0,03 | 0,6582 |
| 17012692 | trace amine associated receptor 8 | TAAR8 | 0,04 | 0,0062 | -0,02 | 0,8888 |
| 16658525 | CAMTA1 intronic transcript 1 (non-protein coding) | CAMTA1-IT1 | 0,03 | 0,0440 | -0,03 | 0,8403 |
| 17061104 | RAS p21 protein activator 4 \| RAS p21 protein activator 4B | RASA4\| RASA4B | 0,03 | 0,0133 | -0,02 | 0,8431 |

**Table B. Pathways upregulated in response to S6K2 siRNA, but not to S6K1 siRNA.**

| p-value | Term | Term ID | Term description | Genes |
| --- | --- | --- | --- | --- |
| 1.67e-07 | GO:0044237 | BP | cellular metabolic process | ARHGEF7, CCNE2, OGDHL, POMC, ARL4D, LZTR1, PPP1R15B, PTPRZ1, WNK2, CACNB4, DAK, GNA13, PTRF, ATF4, CCDC8, CNOT1, CYR61, GPC3, NKX2-3, NT5M, PRAMEF14, RNF111, TADA3, AIMP1, CDKN3, CRTC2, DCAF11, FLII, FOXK2, GTF2E1, MDM2, MYO9B, NCOA6, NDUFS2, NPTN, OAT, PEX2, PIM2, RNF141, SCD, UBE2J1, USP10, ZNF219, ARF6, B3GAT3, CREB3L4, CSPG5, CTDNEP1, DCTPP1, GPCPD1, MUC13, NAGPA, PSMD10, PSME3, RFC4, SLC27A4, SOAT1, SUPT7L, UGGT1, USP36, ZBTB46, ZBTB49, AHCY, ASF1B, ATL3, CAMTA2, MMS19, NOLC1, PSENEN, RNASEL, SBF1, SYDE2, TSG101, AACS, DRG1, FOXK1, GFER, HSPH1, RPL37A, RQCD1, RSF1, SCML1, SEC13, SORBS3, ST8SIA3, TBX3, TNK1, TRIP4, UBE2I, WDR83, ABL1, ARFGEF2, DNAJB1, DYNLL1, GNG12, GPC4, HSPA9, IKZF5, LPCAT4, LRTOMT, MAN2A1, PAK3, POLA2, PPP1R26, SMUG1, SPSB1, TEAD1, TRMT6, TSEN15, UBXN2A, VAV3, ZNF227, APBB2, C14ORF126, EIF5B, ENOPH1, ERBB3, FANCD2, GRSF1, IDH3A, IRF1, LRP5, MED13, MLL2, NAPEPLD, NCAPG2, NFATC3, PAFAH1B2, PTCH1, RBM15, RBM23, SLC2A4RG, TCEAL2, TMEM18, ULK3, VEZF1, WFS1, WNK1, AEBP2, ATE1, BRPF1, CISD2, COX7C, DNM3, PAF1, PPP1R8, SERPINE1, SLC25A5, TAB2, TFAP2B, TIMM9, TIPARP, TMEM173, TSR2, WDR45L, ADCY1, AMD1, ARGLU1, ATP5C1, B4GALT6, BUB3, CAMTA1, CPT1A, DTX3, F7, HMGXB3, LUC7L3, MED31, MOCS3, SETD7, USP19, AAK1, AKIRIN2, AR, DDX47, HECW2, JARID2, LPAR3, LPIN3, MARC1, MBNL1, NME3, NOA1, ODC1, PHAX, PLCG1, RIMKLB, RMI1, TIMM21, USP21, UTP11L, ZNF418, ACSM3, ALG12, ATAD2, COX7A2L, DCAF12, DDX20, DERL1, GSK3B, GYG1, HNRNPF, INPP4B, KRT17, LIPT2, NAE1, PPAP2A, PRPF38A, PRPF4, PXK, RGL1, SERAC1, UAP1, ANAPC4, DNER, ETNK2, ILF3, NSUN2, PCGF3, PCIF1, PDS5A, PELI3, ADNP2, ANAPC7, ARAP2, ARHGAP5, ATXN7, BAP1, COG2, DHX15, ERC1, EZH2, GAS6, GFM1, HEXA, MKRN2, MLH3, MOB1B, NEK4, PARP4, PKP4, RNF152, SF3B3, SSH3, TCEAL8, THOC2, TULP4, ZNF554, ZNF584, ZNF621, ACSL3, APLP2, ATP5EP2, BAG5, BCL11B, DLD, HSPA14, HTRA2, INPP5D, IRF3, MRPL48, NCK2, PSMD1, PTPLAD1, STRA13, TUSC3, UBR2, USP9X, VDR, YWHAH, ZNF292, CHDH, FHL2, FOXJ3, GMNN, HSCB, LGR4, ME2, MKI67, MRPS30, NFRKB, PARN, RBPJ, SCAI, SETDB1, SMARCAL1, SRD5A3, TTK, ZFC3H1, CENPK, CYLD, DHX35, DIDO1, ERI1, EXOSC10, FASTKD2, FRRS1, HLCS, NFATC2, PSMD12, RAB9A, SMAP2, USP39, ZNF267, ATXN2, BCL2, C19ORF40, C6ORF130, CIB1, DEGS1, HEMK1, HERC1, LRP1, MAN1A2, MRPS10, NR2C2, POLE2, PPAT, PTPN3, REV3L, ZNF180, ZNF627, ZNF75A, BRIP1, CBR4, CNEP1R1, DIS3, FUT8, GTF3C4, LMTK2, MGRN1, PDXDC1, REST, TAF15, TNFRSF21, UBQLN4, USP38, WDR82, ZNF510, ZNRF3, ALG2, CAPRIN2, DOCK7, ELF2, FIG4, HSD3B7, IKBKB, MBTPS1, RNASEH2B, SEC63, SREBF1, VBP1, VPS4B, AGFG1, ANKRD49, ATG14, CCNL1, COQ9, GPATCH2, ICT1, NAB2, PKNOX1, POLR3B, PRPS2, RBM27, RNPC3, RYBP, SUDS3, TBC1D10B, ZNF236, ZNF318, ZNF800, BACE2, BRF1, DGUOK, DYRK1A, MAP3K1, MYO6, NPC1, PLEKHA1, PSMB1, SEC24A, TWSG1, UBE2D1, ZBTB25, CASP3, GTF2A1, HDAC1, ICMT, KIAA1804, LPGAT1, NIF3L1, PRKCI, RAP1GDS1, RNF169, SETX, USP47, ZNF776, ZRANB3, ESD, GUF1, PPIC, SLTM, SOS1, TBC1D2, ZBTB33, ACVR2A, C5, CCNT1, E2F3, E2F7, ELL, KDM6A, LIF, LPAR1, MED28, PHF16, PPIG, PRKD2, PTPN12, RBM17, RPA1, RPRD1B, SPRED2, ZBTB39, EIF2AK2, ENC1, MTMR3, PEX13, SMC1A, SSH2, TOR1A, TYW5, USP4, ZNF780B, CCNE1, CSTF3, GPBP1, KLHL21, MAGEC2, NFYA, PALB2, PASK, PJA2, RAP2B, SAT1, SPRED1, YARS2, ALMS1, CEBPG, DCAF16, GZF1, HNRNPH3, ING2, LEMD3, LPCAT2, POLR3E, PRKACA, CDK12, DNAJC1, NDEL1, SENP1, SRPK2, SRR, ZNF460, AMPD2, ZNF444, KAT6A, MND1, NEK7, NSD1, TFB1M, USP37, PPIP5K1, SLC23A2, SOCS5, TIA1, RFT1, RPS15A, MTHFR, ZNF543, ZNF91, ARNT2, ERCC4, NDUFAF1, HACE1, KLHL9, NRARP, ZNF484, ACAA2, HIVEP1, MIER3, TTBK2, USP1, CHST14, DAPK2, DNMT1, PTEN, PYDC1, ATP1B1, BTBD1, IL6ST, ZNF17, NAA16, PDPR |
| 2.96e-03 | GO:0006996 | BP | organelle organization | BCS1L, ACTR3, TADA3, FMNL2, PEX2, PIM2, ARF6, CTDNEP1, NAGPA, PSMD10, RFC4, SUPT7L, ASF1B, ATL3, BICD2, CABIN1, NOLC1, GFER, RSF1, SEC13, SORBS3, UBE2I, ABL1, ARFGEF2, BAIAP2L1, CKAP5, DYNLL1, HSPA9, MAN2A1, PAK3, POLA2, PTGER4, VAV3, EPB49, FANCD2, LIMCH1, LRP5, MLL2, NCAPG2, AEBP2, BRPF1, PAF1, TIMM9, WDR45L, BUB1, BUB3, SETD7, VPS33B, BBS2, CCNG2, DAG1, JARID2, NOA1, TIMM21, USP21, KIF2A, KRT17, NUP155, TACC2, ANAPC4, KIF23, NSUN2, PDS5A, ANAPC7, ATXN7, BAP1, COG2, EZH2, GCC2, GFM1, HEXA, MLH3, NEK4, SSH3, HTRA2, NCK2, NEDD1, NIP7, STRA13, SYNJ2BP-COX16, TDRD3, TWF2, UBR2, USP9X, EEA1, RANBP9, SMARCAL1, TTK, CENPK, CYLD, HLCS, WDPCP, ZMYM4, BCL2, LRP1, PEX3, POLE2, RRS1, GTF3C4, REST, WDR82, DOCK7, FIG4, NUP160, SREBF1, VPS4B, AGFG1, ATG14, FAT1, FOPNL, ICT1, RYBP, SUDS3, MAP3K1, SEC24A, UBE2D1, CASP3, CELSR1, HDAC1, PRKCI, CAMSAP1, MTFP1, KDM6A, KIF11, PHF16, RPA1, PEX13, SMC1A, SSH2, TOR1A, KLHL21, YARS2, ING2, NDEL1, SRPK2, VPS54, KAT6A, NEK7, NSD1, USP37, IQSEC1, ERCC4, HACE1, KLHL9, DNMT1, PTEN, TUBGCP3, CEP152 |
| 4.10e-13 | GO:0044424 | CC | intracellular part | PIEZO1, SPAG8, ARHGEF7, CCNE2, KRTAP22-1, OGDHL, POMC, SH3BGRL2, ARL4D, BCS1L, PPP1R15B, SDAD1, SDF4, WNK2, CACNB4, DAK, GNA13, OTUD7A, PTRF, RPL7L1, RUSC2, TXNDC11, ACTR3, ATF4, CNOT1, EPN1, GPC3, HDX, MMGT1, NKX2-3, NT5M, PAQR3, RAB11FIP5, RNF111, TADA3, AIMP1, CDKN3, CRTC2, DCAF11, FLII, FMNL2, FOXK2, GTF2E1, MDM2, MYO9B, NCOA6, NDUFS2, OAT, PEX2, SCD, SERPINF1, SMYD3, TAGAP, TM9SF2, UBE2J1, USP10, ZNF219, ARF6, B3GAT3, COMMD4, CREB3L4, CSPG5, CTDNEP1, DCTPP1, GPCPD1, MUC13, NAGPA, PSMD10, PSME3, RFC4, SLC27A4, SOAT1, SUPT7L, UGGT1, USP36, ZBTB46, ZBTB49, AHCY, ASF1B, ATL3, ATXN10, BICD2, CABIN1, CAMTA2, CDC42EP4, KDELR2, MMS19, NAT10, NOLC1, NUP210, PSENEN, RNASEL, SAMD9, SBF1, SYDE2, TMTC1, TRADD, TRAPPC11, TSG101, AACS, BYSL, C12ORF10, CEP112, DRG1, FOXK1, GFER, HSPH1, RPL37A, RQCD1, RSF1, RTN2, SAMHD1, SCML1, SEC13, SORBS3, ST8SIA3, TBX3, TNK1, TOE1, TRIP4, UBE2I, WDR83, ABL1, ARFGEF2, BAIAP2L1, CEP89, CKAP5, DNAJB1, DYNLL1, GCA, GNG12, GPC4, HSPA9, IKZF5, LPCAT4, LRTOMT, MAN2A1, NOL11, PAK3, POLA2, PPP1R26, SMUG1, SPSB1, SYT17, TEAD1, TRMT6, TSEN15, UBXN2A, VAV3, ZDHHC8, ZNF227, ANKRD27, APBB2, C14ORF126, COL4A1, EIF5B, ENOPH1, EPB49, ERBB3, FANCD2, GLRX3, GRSF1, GTSE1, IDH3A, IRF1, LAP3, LRP5, MED13, MLL2, MOB1A, NCAPG2, NFATC3, PAFAH1B2, PTCH1, RBM15, RBM23, RSL24D1, SDCCAG3, SLC25A19, SLC2A4RG, TCEAL2, TMEM106B, TMEM18, TPM4, ULK3, VEZF1, WFS1, WNK1, XPO4, ZNRF2, AEBP2, ATE1, BRPF1, CCDC14, CHMP7, CISD2, COX7C, DNM3, DRAM2, GHITM, KCND2, NAGLU, NARG2, PAF1, PLEKHA8P1, PPP1R8, RASEF, SERPINE1, SLC25A5, STAU1, TAB2, TFAP2B, TIMM9, TMEM173, TMEM57, WDR45L, ADCY1, AMD1, ARGLU1, ATP5C1, B4GALT6, BUB1, BUB3, CAMTA1, CARKD, COIL, CPT1A, DTX3, F7, HMGXB3, ITSN2, KRTAP10-11, LUC7L3, MED31, MOCS3, NUP153, PPFIA1, SETD7, USP19, WDSUB1, VPS33B, AAGAB, AAK1, AKIRIN2, AR, BBS2, CCNG2, CEP78, CHMP4B, DAG1, DDX47, DHX36, DONSON, HECW2, JARID2, LACTB2, LPAR3, LPIN3, MARC1, MBNL1, NME3, ODC1, PHAX, PLCG1, PPP2R5E, RABL6, RIMKLB, RMI1, STIM2, TIMM21, TMBIM6, USP21, UTP11L, ZNF418, ACSM3, AKAP9, ALG12, ATAD2, COX7A2L, DCAF12, DDX20, DERL1, DET1, GSK3B, GYG1, HNRNPF, INPP4B, KIF1B, KIF2A, KRT17, LIPT2, MVP, NAE1, NDRG3, NUP155, PCBP4, PRPF38A, PRPF4, PXK, RGL1, SERAC1, TACC2, TRH, UAP1, ANAPC4, DNER, ETNK2, ILF3, KIF23, NSUN2, PCGF3, PCIF1, PDS5A, PELI3, RCN2, RIN2, RSBN1, SH3BP4, SLC25A33, ADNP2, ANAPC7, ARAP2, ARHGAP5, ARL6, ATXN7, BAP1, CLDN17, COG2, DHX15, ERC1, EZH2, GAS6, GCC2, GFM1, HEXA, KLK7, MLH3, MOB1B, NEK4, PARP12, PARP4, PHLPP1, PKP4, POC5, RNF152, SF3B3, SSH3, TCEAL8, TGOLN2, THOC2, TULP4, VPS33A, ZNF554, ZNF584, ZNF621, ACSL3, APLP2, ATP5EP2, BAG5, BCL11B, DENND1A, DLD, FNBP1, HSPA14, HTRA2, IFT46, INPP5D, IRF3, MRPL48, NCK2, NEDD1, NIP7, PITPNA, PSMD1, PTPLAD1, SLC4A7, STRA13, SYNJ2BP-COX16, TDRD3, TMEM9, TUSC3, TWF2, UBR2, USP9X, VDR, YWHAH, ZNF292, AP1B1, CHDH, CYFIP2, EEA1, FBXW8, FHL2, FOXJ3, GMNN, HSCB, ME2, MKI67, MRPS30, NFRKB, OXR1, PARN, RANBP9, RBPJ, RRAGD, SCAI, SCAPER, SETDB1, SMARCAL1, SRD5A3, TCP11L1, TNNC2, TTK, UFSP2, CENPK, CRKL, CYLD, DHX35, DIDO1, ERI1, EXOSC10, FASTKD2, HLCS, IGF2R, LRRC1, NFATC2, NOL12, NUDCD1, PSMD12, RAB9A, RINT1, RSPH1, RUFY2, SMAP2, SPINT2, USP39, WDPCP, ZMYM5, ZNF267, ATXN2, BCL2, C19ORF40, CIB1, DACT2, DEGS1, HEMK1, HERC1, JAGN1, LRP1, MAN1A2, MRPS10, NR2C2, PEX3, POLE2, PPAT, PTPN3, REV3L, RRS1, SERPINB1, ZNF180, ZNF627, ZNF75A, AKAP11, BRIP1, C16ORF70, CBR4, CNEP1R1, DIS3, EXPH5, FUT8, GTF3C4, LMTK2, MEX3D, MGRN1, REST, SAYSD1, TAF15, TES, TNFRSF21, UBQLN4, UBTD2, WDR82, ZNF510, AKAP13, ALG2, CAPRIN2, ELF2, FIG4, HSD3B7, IKBKB, MBTPS1, NUP160, RNASEH2B, SEC63, SREBF1, VBP1, VPS4B, ZBED4, AGFG1, AMIGO2, ANKRD49, ATG14, CCNL1, COQ9, FAT1, FOPNL, GOLGA4, ICT1, LAMTOR3, NAB2, PKNOX1, POLR3B, PPP2R3C, RBM27, RNPC3, RYBP, STARD3NL, SUDS3, TBC1D10B, TXNDC12, VPS29, ZNF236, ZNF318, ZNF800, BACE2, BRF1, CENPBD1, DGUOK, DYRK1A, GLE1, LAMP2, MAP3K1, MYO6, NPC1, PLEKHA1, PSMB1, RRAGA, SEC24A, SEC61A2, STX10, TRIM4, UBE2D1, ZBTB25, CASP3, DHX32, GTF2A1, HDAC1, ICMT, LPGAT1, NIF3L1, NIPSNAP3A, PRKCI, RNF169, SETX, STRN, USP47, ZNF776, ZRANB3, CAMSAP1, CHCHD1, DDX18, ESD, GUF1, MTFP1, NTAN1, PLCH1, PPIC, PTGES, SLTM, SOS1, TBC1D2, ZBTB33, ACVR2A, ARHGAP32, CCNT1, DPP8, E2F3, E2F7, ELL, KDM6A, KIF11, KIF1A, LIF, LPAR1, MED28, OSBPL1A, PHF16, PPIG, PRKD2, PTPN12, RBM17, RPA1, RPRD1B, SPRED2, ZBTB39, EIF2AK2, ENC1, GORAB, MTMR3, PEX13, SMC1A, SSH2, TOR1A, USP4, ZNF780B, CCNE1, CSTF3, DIABLO, EPN2-IT1, GPBP1, KDELC2, KLHL21, MAGEC2, NFYA, PALB2, PASK, PJA2, RAB27A, RAP2B, SAT1, SCEL, SLC25A30, SPRED1, YARS2, ALMS1, CEBPG, DCAF16, FUCA1, GZF1, HNRNPH3, ING2, LEMD3, LPCAT2, POLR3E, PRKACA, SMEK1, C6ORF57, CDK12, DNAJC1, NDEL1, PRRG4, SENP1, SPATA5, SRPK2, SRR, ZNF460, AMPD2, VPS54, ZNF444, ITPKC, KAT6A, MND1, NEK7, NSD1, PANX1, TFB1M, USP37, IQSEC1, PPIP5K1, SEZ6L2, SLC23A2, TIA1, RPS15A, MTHFR, ZNF543, ZNF91, ARNT2, ATAD5, ERCC4, NDUFAF1, HACE1, KLHL9, ZNF484, ACAA2, HIVEP1, MIER3, USP1, CHST14, DAPK2, DNMT1, KATNAL2, PTEN, PYDC1, TUBGCP3, MEIS3P1, BTBD1, EXOC2, ZNF17, MT1X, NAA16, PDPR, UBAP1, SPATA2, CEP152, RPGRIP1L |
| 5.99e-07 | GO:0005488 | MF | binding | SPAG8, ARHGEF7, CCNE2, OGDHL, POMC, SH3BGRL2, ZG16B, ARL4D, BCS1L, RBM18, SDF4, WNK2, CACNB4, DAK, GNA13, OTUD7A, PTRF, ACTR3, ATF4, CNOT1, CYR61, EPN1, FGF5, GPC3, HDX, NKX2-3, NT5M, PRAMEF14, RAB11FIP5, RNF111, TADA3, AIMP1, BCAM, CDKN3, DCAF11, FLII, FMNL2, FOXK2, GTF2E1, IL17RA, MDM2, MYO9B, NCOA6, NDUFS2, NPTN, OAT, PEX2, PIM2, RNF141, SCD, SMYD3, UBE2J1, USP10, ZNF219, ARF6, B3GAT3, COMMD4, CREB3L4, CSPG5, CTDNEP1, DCTPP1, GPCPD1, KCNIP1, MB, PSMD10, PSME3, RFC4, SDR42E1, SLC27A4, SOAT1, UGGT1, ZBTB46, ZBTB49, ASF1B, ATL3, ATXN10, BICD2, CABIN1, CAMTA2, CDC42EP4, KDELR2, MMS19, NAT10, NOLC1, NUP210, PSENEN, R3HDM1, RNASEL, SBF1, TRADD, TSG101, AACS, BYSL, DRG1, FOXK1, GFER, HSPH1, PRRC2C, R3HCC1L, RPL37A, RQCD1, RSF1, RTN2, SAMHD1, SCML1, SEC13, SORBS3, ST8SIA3, TBC1D9B, TBX3, TNK1, TOE1, TRIP4, UBE2I, ABL1, ARFGEF2, BAIAP2L1, C17ORF48, CKAP5, DNAJB1, DYNLL1, GCA, GNG12, GPC4, HSPA9, IKZF5, LPCAT4, MAN2A1, MON1B, OSBPL7, PAK3, POLA2, PPP1R26, PTGER4, SMUG1, TEAD1, TRMT6, UBXN2A, VAV3, ZDHHC8, ZNF227, ANKRD27, APBB2, COL4A1, DDX60L, EIF5B, ENOPH1, EPB49, ERBB3, FANCD2, GLRX3, GRSF1, IDH3A, IRF1, LAP3, LIMCH1, LRP5, MED13, MLL2, MMP9, MOB1A, NAPEPLD, NCAPG2, NFATC3, OSBPL2, PAFAH1B2, PTCH1, RBM15, RBM23, SLC2A4RG, TMEM18, TPM4, TRIM65, ULK3, VEZF1, WFS1, WNK1, XPO4, ZNHIT1, ZNRF2, AEBP2, BRPF1, CISD2, DNM3, GHITM, KCND2, LNX2, NMB, OSBPL10, PAF1, PLEKHA8P1, PPP1R8, RASEF, SERPINE1, SLC25A5, STAU1, TAB2, TFAP2B, TIMM9, TIPARP, TMEM173, TSR2, WDR45L, ADAM18, ADCY1, B4GALT6, BUB1, BUB3, CAMTA1, CARKD, COIL, CPT1A, DTX3, F7, HMGXB3, ITSN2, LUC7L3, MED31, MOCS3, NUP153, PPFIA1, SETD7, USP19, VPS33B, YTHDF3, AAK1, AR, BBS2, CHMP4B, DAG1, DDX47, DHX36, JARID2, KCMF1, KREMEN1, LACTB2, LPAR3, MARC1, MBNL1, NME3, NOA1, PCDH1, PHAX, PLCG1, PPP2R5E, PTPRS, RABL6, RIMKLB, RMI1, STIM2, USP21, UTP11L, ZNF418, ACSM3, AKAP9, ATAD2, DDX20, DERL1, GSK3B, GYG1, HNRNPF, KIF1B, KIF2A, KRT17, LGALSL, MVP, NAE1, OSBP2, PCBP4, PRPF4, PXK, TACC2, THUMPD3, TRH, ANAPC4, DNER, ETNK2, ILF3, KIF23, LARP4B, NSUN2, PCGF3, PDS5A, PLXDC2, RCN2, SH3BP4, SNX11, TET2, ADNP2, ANAPC7, ARAP2, ARHGAP5, ARL6, ATXN7, BAP1, CLDN17, COG2, DHX15, ERC1, EZH2, GAS6, GCC2, GFM1, HEXA, KCNMB4, MKRN2, MLH3, MOB1B, NEK4, PARP12, PARP4, PHLPP1, PKP4, RNF152, RNF214, SF3B3, SSH3, TCEAL8, TGOLN2, THOC2, VPS33A, ZNF554, ZNF584, ZNF621, ACSL3, APLP2, BAG5, BCL11B, DENND1A, DLD, FNBP1, HSPA14, HTRA2, IFT46, INPP5D, IRF3, MRPL48, NCK2, NIP7, PITPNA, STRA13, SYNJ2BP-COX16, TDRD3, TWF2, UBR2, USP9X, VDR, YWHAH, ZDHHC4, ZNF292, AP1B1, CHDH, CYFIP2, EEA1, FBXW8, FHL2, FOXJ3, GMNN, HSCB, ME2, MKI67, MPP6, NFRKB, PARN, RANBP9, RBPJ, RRAGD, SCAI, SCAPER, SETDB1, SMARCAL1, TM2D1, TNNC2, TTK, ZFC3H1, CRKL, CYLD, DHX35, DIDO1, ERI1, EXOSC10, FRRS1, HLCS, IGF2R, NENF, NFATC2, NOL12, RAB9A, RINT1, RUFY2, SCIMP, SMAP2, USP39, ZMYM4, ZMYM5, ZNF267, ATXN2, BCL2, C19ORF40, C6ORF130, CIB1, DACT2, HEMK1, LRP1, MAN1A2, NR2C2, PEX3, POLE2, PPAT, PPP1R16A, PTPN3, REV3L, SLC22A5, TNS3, ZDHHC18, ZNF180, ZNF627, ZNF704, ZNF75A, AKAP11, BRIP1, C16ORF70, CBR4, CNEP1R1, CPEB4, DIS3, EXPH5, FUT8, GTF3C4, LMTK2, MEX3D, MGRN1, MOB3B, PDXDC1, REST, TAF15, TES, TNFRSF21, UBQLN4, WDR82, ZNF510, ZNRF3, AKAP13, ALG2, CAPRIN2, DOCK7, ELF2, FIG4, HSD3B7, IKBKB, NUP160, SCNN1B, SEC63, SREBF1, VBP1, VPS4B, ZBED4, AGFG1, AMIGO2, ATG14, CCNL1, FAT1, GOLGA4, GPATCH2, ICT1, LAMTOR3, NAB2, PKNOX1, PLEKHA5, POLR3B, PPP2R3C, PRPS2, RBM27, RNPC3, RYBP, SUDS3, VPS29, ZNF236, ZNF318, ZNF800, BRF1, CENPBD1, DGUOK, DYRK1A, MAP3K1, MYO6, NPC1, PLEKHA1, PSMB1, PVRL4, RRAGA, SEC24A, SEC61A2, SLC1A1, STX10, TRIM4, UBE2D1, ZBTB25, CASP3, CELSR1, CYB5D1, DHX32, FLVCR1, GTF2A1, HDAC1, KIAA1804, NIF3L1, NIPSNAP3A, PRKCI, RNF169, SETX, STRN, USP47, ZNF776, ZRANB3, CAMSAP1, CHCHD1, DDX18, GUF1, PLCH1, PPIC, PTGES, RNF219, SLTM, SOS1, TBC1D2, ZBTB33, ACVR2A, ARHGAP32, C1ORF27, C5, CCNT1, E2F3, E2F7, ELL, KDM6A, KIF11, KIF1A, LIF, LPAR1, MED28, OSBPL1A, PHF16, PPIG, PRKD2, PTPN12, RBM17, RPA1, RPRD1B, SESTD1, SPRED2, TMEM30B, ZBTB39, DOCK10, EIF2AK2, ENC1, GORAB, MTMR3, PEX13, SMC1A, SSH2, TOR1A, TYW5, USP4, ZNF780B, CCNE1, CSTF3, DIABLO, EPN2-IT1, GPBP1, MAGEC2, NFYA, PALB2, PASK, PJA2, RAB27A, RAP2B, SAT1, SCEL, SPRED1, YARS2, ANKRD5, CEBPG, DCAF16, FUCA1, GZF1, HNRNPH3, ING2, LEMD3, LPCAT2, LRP12, PRKACA, CDK12, DNAJC1, MAST3, NDEL1, PRRG4, SPATA5, SRPK2, SRR, ZNF460, AMPD2, VPS54, ZNF444, ITPKC, KAT6A, MND1, NEK7, NSD1, PANX1, RAB26, TFB1M, USP37, IQSEC1, PPIP5K1, SOCS5, TIA1, IL10RB, RBM41, RPS15A, MTHFR, PCDHB5, TTC14, ZNF543, ZNF91, ARNT2, ATAD5, ERCC4, NDUFAF1, TRIM38, HACE1, ZNF484, ACAA2, HIVEP1, KBTBD7, MIER3, TTBK2, USP1, CHST14, DAPK2, DNMT1, KATNAL2, PTEN, PYDC1, TUBGCP3, BEND7, MEIS3P1, ATP1B1, BTBD1, EXOC2, IL6ST, ZNF17, MT1X, CEP152, RPGRIP1L |
| 2.72e-02 | GO:0019783 | MF | small conjugating protein-specific protease activity | USP10, USP19, USP21, BAP1, UFSP2, CYLD, USP47, USP4, SENP1, USP37, USP1 |
| 8.18e-10 | BIOGRID:00000 | bi | BioGRID interaction data | PIEZO1, SPAG8, ARHGEF7, CCNE2, OGDHL, POMC, SH3BGRL2, TMTC3, ZG16B, ARL4D, BCS1L, C5ORF34, LZTR1, PPP1R15B, PTPRZ1, SDAD1, SDF4, WNK2, CACNB4, DAK, GNA13, IQCK, KBTBD2, LRIG1, OTUD7A, PTRF, RPL7L1, RUSC2, TXNDC11, ACTR3, ATF4, CCDC8, CNOT1, CYR61, EPN1, GPC3, HDX, MMGT1, NKX2-3, PAQR3, RAB11FIP5, RNF111, TADA3, AIMP1, BCAM, C3ORF37, CDKN3, CRTC2, DCAF11, FLII, FMNL2, FOXK2, GTF2E1, IL17RA, MDM2, MYO9B, NCOA6, NDUFS2, NPTN, OAT, PEX2, PIM2, SCD, SERPINF1, SMYD3, TM9SF2, UBE2J1, USP10, ZNF219, ALDH16A1, ARF6, B3GAT3, COMMD4, CREB3L4, CSPG5, CTDNEP1, DCTPP1, GPCPD1, MB, MUC13, NAGPA, PSMD10, PSME3, RFC4, SDR42E1, SLC27A4, SLC35E1, SOAT1, SUPT7L, TMEM230, UGGT1, USP36, AHCY, ASF1B, ATL3, ATXN10, BICD2, CABIN1, CAMTA2, CDC42EP4, DDA1, KDELR2, MMS19, NAT10, NOLC1, NUP210, PSENEN, RNASEL, SAMD9, SBF1, SYDE2, TRADD, TSG101, AACS, BYSL, C12ORF10, DRG1, FOXK1, GFER, HSPH1, PLXND1, PRRC2C, RPL37A, RQCD1, RSF1, RTN2, SAMHD1, SEC13, SORBS3, TBC1D9B, TBX3, TNK1, TOE1, TRIP4, UBE2I, ABL1, ARFGEF2, BAIAP2L1, C11ORF58, CEP89, CKAP5, DNAJB1, DYNLL1, GCA, GNG12, GPC4, HSPA9, IKZF5, LPCAT4, MANBAL, MON1B, NOL11, PAK3, POLA2, PTGER4, SMUG1, SPSB1, SPTY2D1, SYT17, TEAD1, TRMT6, TSEN15, UBXN2A, VAV3, ZDHHC8, ZNF227, ANKRD27, APBB2, CFP, COL4A1, DDX60L, EIF5B, ENOPH1, EPB49, ERBB3, FANCD2, GLRX3, GRSF1, GTSE1, IDH3A, IRF1, LAP3, LRP5, MED13, MLL2, MMP9, MOB1A, NAPEPLD, NCAPG2, NFATC3, OSBPL2, PAFAH1B2, PTCH1, RBM15, RBM23, RSL24D1, SDCCAG3, SLC25A19, SLC2A4RG, SLC7A1, TCEAL2, TMEM106B, TMEM18, TPM4, TRIM65, ULK3, VEZF1, WFS1, WNK1, XPO4, ZNHIT1, ZNRF2, AEBP2, ATE1, BRPF1, CCDC14, CHMP7, CISD2, CLDND1, COX7C, DNM3, FN3KRP, GHITM, KCND2, LNX2, NAGLU, NARG2, NMB, OSBPL10, PAF1, PPP1R8, RASEF, SERPINE1, SLC25A5, SLC30A1, STAU1, TAB2, TFAP2B, TIMM9, TMEM173, TMEM57, TSR2, WDR45L, AMD1, ARGLU1, ATP5C1, BUB1, BUB3, C5ORF51, CARKD, COIL, CPT1A, DTX3, F7, HMGXB3, ITSN2, LUC7L3, MED31, MOCS3, NUP153, PDCL, PPFIA1, SETD7, USP19, VPS33B, YTHDF3, AAGAB, AKIRIN2, AR, BBS2, CCNG2, CEP78, CHMP4B, DAG1, DDX47, DHX36 |
| 2.33e-02 | HP:0001410 | hp | Decreased liver function | BCS1L, NDUFS2, PEX2, CPT1A, BBS2, ARL6, GFM1, DLD, FASTKD2, WDPCP, PEX3, HSD3B7, DGUOK, NPC1, PEX13, ALMS1, NDUFAF1, RPGRIP1L |
| 2.51e-02 | HP:0009121 | hp | Abnormal axial skeleton morphology | PTRF, CCDC8, GPC3, AIMP1, NDUFS2, PEX2, B3GAT3, ATXN10, SAMHD1, TBX3, PAK3, COL4A1, ERBB3, FANCD2, LRP5, MLL2, PTCH1, SLC25A19, WNK1, NAGLU, TAB2, TFAP2B, VPS33B, AR, BBS2, DAG1, NSUN2, RIN2, TET2, ARL6, EZH2, GFM1, HEXA, DLD, VDR, RBPJ, SMARCAL1, SPATA7, SRD5A3, WDPCP, PEX3, BRIP1, RNASEH2B, SEC63, COQ9, PRPS2, DGUOK, GLE1, FLVCR1, SETX, SOS1, KDM6A, KIF11, KIF1A, GORAB, PEX13, SMC1A, TOR1A, PALB2, SPRED1, YARS2, ALMS1, FUCA1, LEMD3, NSD1, MTHFR, NDUFAF1, CHST14, PTEN, CEP152, RPGRIP1L |
| 2.56e-02 | HP:0000553 | hp | Abnormality of the uvea | GPC3, OAT, PEX2, ATXN10, TEAD1, COL4A1, FANCD2, LRP5, MLL2, PTCH1, SRD5A3, WDPCP, PEX3, BRIP1, ALG2, SOS1, KDM6A, KIF11, PEX13, PALB2, RAB27A, ALMS1, PTEN, RPGRIP1L |
| 3.21e-03 | HP:0000240 | hp | Abnormality of skull size | GPC3, AIMP1, NDUFS2, PEX2, SAMHD1, PAK3, COL4A1, FANCD2, LRP5, MLL2, PTCH1, SLC25A19, TAB2, VPS33B, BBS2, NSUN2, ARL6, EZH2, GFM1, HEXA, DLD, RBPJ, SRD5A3, WDPCP, PEX3, BRIP1, RNASEH2B, COQ9, DGUOK, SOS1, KDM6A, KIF11, PEX13, SMC1A, PALB2, SPRED1, YARS2, LEMD3, NSD1, MTHFR, NDUFAF1, PTEN, CEP152, RPGRIP1L |
| 4.67e-03 | HP:0001252 | hp | Muscular hypotonia | BCS1L, GPC3, AIMP1, NDUFS2, PEX2, B3GAT3, ATXN10, COL4A1, LRP5, MLL2, SLC25A19, WNK1, TAB2, CAMTA1, CPT1A, VPS33B, AR, ALG12, NSUN2, RIN2, GFM1, HEXA, DLD, VDR, SPATA7, FASTKD2, HLCS, ATXN2, PEX3, SLC22A5, PRPS2, DGUOK, NPC1, SOS1, KDM6A, KIF11, GORAB, PEX13, SMC1A, TOR1A, YARS2, ALMS1, FUCA1, LEMD3, NSD1, NDUFAF1, PTEN, RPGRIP1L |
| 2.39e-02 | HP:0007319 | hp | Morphological abnormality of the central nervous system | BCS1L, CCDC8, GPC3, AIMP1, NDUFS2, PEX2, B3GAT3, ATXN10, SAMHD1, PAK3, COL4A1, FANCD2, LRP5, MLL2, PTCH1, SLC25A19, WFS1, TAB2, CAMTA1, F7, VPS33B, AR, BBS2, KIF1B, SERAC1, TRH, NSUN2, TET2, ARL6, ATXN7, GFM1, DLD, RBPJ, SMARCAL1, SPATA7, SRD5A3, FASTKD2, WDPCP, ATXN2, PEX3, BRIP1, RNASEH2B, SCNN1B, COQ9, PRPS2, DGUOK, NPC1, SETX, RTTN, KDM6A, KIF11, KIF1A, PEX13, SMC1A, PALB2, RAB27A, YARS2, ALMS1, FUCA1, LEMD3, NSD1, MTHFR, NDUFAF1, TTBK2, DNMT1, PTEN, CEP152, RPGRIP1L |
| 1.60e-02 | HP:0000707 | hp | Abnormality of the nervous system | BCS1L, PTRF, CCDC8, GPC3, AIMP1, IL17RA, NDUFS2, PEX2, B3GAT3, AHCY, ATXN10, RTN2, SAMHD1, PAK3, COL4A1, ERBB3, FANCD2, LRP5, MLL2, PTCH1, SLC25A19, WFS1, WNK1, NAGLU, TAB2, TFAP2B, CAMTA1, CPT1A, F7, VPS33B, AR, BBS2, DAG1, ALG12, KIF1B, KRT17, SERAC1, TRH, NSUN2, TET2, ARL6, ATXN7, BAP1, EZH2, GFM1, HEXA, DLD, VDR, RBPJ, SMARCAL1, SPATA7, SRD5A3, FASTKD2, HLCS, WDPCP, ATXN2, PEX3, SLC22A5, BRIP1, ALG2, HSD3B7, RNASEH2B, SCNN1B, SEC63, COQ9, POLR3B, PRPS2, DGUOK, GLE1, LAMP2, NPC1, SLC1A1, FLVCR1, SETX, RTTN, SOS1, KDM6A, KIF11, KIF1A, GORAB, PEX13, SMC1A, TOR1A, PALB2, RAB27A, SAT1, SPRED1, YARS2, ALMS1, FUCA1, LEMD3, NSD1, MTHFR, ERCC4, NDUFAF1, TTBK2, CHST14, DNMT1, PTEN, CEP152, RPGRIP1L |
| 3.41e-02 | KEGG:04141 | ke | Protein processing in endoplasmic reticulum | ATF4, UBE2J1, UGGT1, HSPH1, SEC13, DNAJB1, WFS1, DERL1, TUSC3, BCL2, MAN1A2, UBQLN4, MBTPS1, SEC63, SEC24A, SEC61A2, UBE2D1, EIF2AK2, DNAJC1 |
| 4.31e-03 | KEGG:03460 | ke | Fanconi anemia pathway | FANCD2, RMI1, STRA13, C19ORF40, REV3L, BRIP1, RPA1, PALB2, ERCC4, USP1 |
| 2.53e-02 | KEGG:05215 | ke | Prostate cancer | CCNE2, ATF4, MDM2, CREB3L4, AR, GSK3B, BCL2, IKBKB, SOS1, E2F3, CCNE1, PTEN |
| 9.38e-03 | MI:hsa-miR-101 | mi | MI:hsa-miR-101 | PIEZO1, CACNB4, XKR6, ATF4, GTF2E1, NCOA6, CSPG5, RSF1, HSPA9, PTGER4, ANKRD27, CCDC18, EIF5B, GRSF1, ZNHIT1, AEBP2, CLDND1, NAGLU, NMB, PPP1R8, BBS2, DDX47, DHX36, TIMM21, INPP4B, SERAC1, RCN2, RIN2, TET2, C1ORF122, EZH2, GCC2, NEK4, PARP4, NEDD1, TUSC3, EEA1, RANBP9, TM2D1, RSPH1, BRIP1, FUT8, USP38, CAPRIN2, ELF2, RMND5A, ZBED4, AGFG1, SLC1A1, TRIM4, KIAA1804, USP47, ZRANB3, KIF11, MAGEC2, SAT1, SRPK2, NEK7, TTC14, KATNAL2, PRR15 |
| 2.70e-02 | MI:hsa-miR-655 | mi | MI:hsa-miR-655 | CACNB4, GPC3, PAQR3, C3ORF37, NCOA6, PEX2, TAGAP, SUPT7L, USP36, ZBTB49, ATXN10, KDELR2, HSPH1, C11ORF58, NOL11, CCDC18, PLEKHA8P1, SLC25A5, WDSUB1, AKIRIN2, NOA1, OTUD1, DDX20, NAE1, PRPF4, CLDN17, GAS6, KCNMB4, HSPA14, GMNN, RANBP9, SCAPER, SETDB1, CENPK, RUFY2, CIB1, DEGS1, CAPRIN2, MBTPS1, SEC63, CCNL1, ICT1, UHRF1BP1L, ZRANB3, EIF2AK2, PASK, ING2, DNMT1, KATNAL2, UBAP1 |
| 1.84e-05 | REAC:69278 | re | Cell Cycle, Mitotic | CCNE2, PSMD10, PSME3, RFC4, SEC13, CKAP5, DYNLL1, POLA2, BUB1, BUB3, CEP78, AKAP9, KIF2A, ANAPC4, KIF23, ANAPC7, NEDD1, PSMD1, GMNN, CENPK, PSMD12, POLE2, NUP160, PSMB1, UBE2D1, E2F3, RPA1, SMC1A, CCNE1, ALMS1, PRKACA, NDEL1, TUBGCP3, CEP152 |
| 3.16e-08 | TF:M00803_0 | tf | Factor: E2F; motif: GGCGSG; match class: 0 | ARHGEF7, CCNE2, OGDHL, ARL4D, BCS1L, HECA, PPP1R15B, PTPRZ1, RBM18, SDF4, CACNB4, GNA13, KBTBD2, LRIG1, PTRF, RPL7L1, RUSC2, SLC35G1, TXNDC11, ATF4, CCDC8, CNOT1, CYR61, EPN1, FGF5, GPC3, MMGT1, NT5M, RAB11FIP5, TADA3, AXDND1, BCAM, CDKN3, FLII, FOXK2, IFI27L1, IL17RA, MDM2, NCOA6, NDUFS2, OAT, PEX2, PIM2, SCD, SERPINF1, TM9SF2, UBE2J1, USP10, ALDH16A1, ARF6, B3GAT3, CREB3L4, CSPG5, DCTPP1, GPCPD1, PSME3, RFC4, SLC27A4, SLC35E1, SOAT1, SUPT7L, TMEM230, UGGT1, USP36, ZBTB49, AHCY, ASF1B, ATL3, ATXN10, BICD2, CABIN1, CDC42EP4, DDA1, KDELR2, MMS19, NOLC1, NUP210, PSENEN, SBF1, TRADD, TRAPPC11, DRG1, FOXK1, GFER, NIPAL1, PLXND1, R3HCC1L, RPL37A, RTN2, SAMHD1, SCML1, SEC13, SORBS3, TBC1D9B, TBX3, UBE2I, WDR83, ABL1, C17ORF48, CEP89, CKAP5, DNAJB1, DYNLL1, GNG12, GPC4, HSPA9, LPCAT4, LRTOMT, MAN2A1, MANBAL, MON1B, NOL11, POLA2, PPP1R26, PTGER4, SPSB1, SPTY2D1, SYT17, TSEN15, UBXN2A, VAV3, WDR90, ZDHHC8, ZNF227, ANKRD27, CCDC18, COL4A1, EIF5B, ENOPH1, ERBB3, FANCD2, GTSE1, LIMCH1, LRP5, MED13, MOB1A, NAPEPLD, NCAPG2, NFATC3, OSBPL2, PTCH1, RBM15, SLC25A19, SLC2A4RG, SLC7A1, TCEAL2, TMEM106B, TPM4, TRIM65, VEZF1, YDJC, ZNHIT1, AEBP2, ATE1, CISD2, CLDND1, COX7C, DNM3, DRAM2, FN3KRP, KCND2, NMB, OSBPL10, PAF1, PPP1R8, RASEF, SLC25A5, SLC30A1, STAU1, TAB2, TIMM9, TIPARP, TMEM57, WDR45L, ADCY1, ATP5C1, B4GALT6, BUB3, CAMTA1, CARKD, CPT1A, KRTAP10-11, LUC7L3, MOCS3, PDCL, PPFIA1, SETD7, USP19, WDSUB1, VPS33B, AAGAB, AAK1, AKIRIN2, CCNG2, CHMP4B, DAG1, DDX47, HECW2, KCMF1, KREMEN1, LACTB2, MARC1, MBNL1, NME3, NOA1, ODC1, PCDH1, PLCG1, RABL6, RMI1, STIM2, TIMM21, TMEM248, UTP11L, ALG12, ATAD2, C20ORF3, C2ORF55, DERL1, GYG1, HNRNPF, KCNK5, KIF1B, LGALSL, NDRG3, NUP155, OSBP2, PCBP4, PRPF38A, PRPF4, PRRT3, PXK, RGL1, SERAC1, TACC2, TMEM170A, TRH, UAP1, ANAPC4, DNER, ETNK2, ILF3, KIF23, NSUN2, PCGF3, PCIF1, PLXDC2, RASGEF1A, RCN2, RSBN1, SH3BP4, SLC25A33, SNX11, TET2, ADNP2, ARAP2, ARHGAP5, ARL6, ATXN7, BAP1, C18ORF25, C18ORF8, C1ORF122, COG2, ERC1, EZH2, GAS6, GFM1, KCNMB4, KLK7, MLH3, PARP4, PHLPP1, PKP4, RNF152, RNF214, SF3B3, SSH3, VPS33A, ZNF584, ACSL3, APLP2, BAG5, DENND1A, FNBP1, HSPA14, IFT46, IRF3, NCK2, NEDD1, NIP7, PITPNA, PSMD1, PTPLAD1, STRA13, TDRD3, TMEM209, TUSC3, TWF2, USP9X, YWHAH, ZDHHC4, CHDH, CYFIP2, EEA1, FHL2, GMNN, HSCB, ME2, MKI67, MPP6, PARN, RANBP9, RBPJ, RRAGD, SCAI, SETDB1, SRD5A3, TCP11L1, TM2D1, TSSC1, UFSP2, CENPK, CRKL, DHX35, DIDO1, ERI1, FASTKD2, FRRS1, HLCS, IGF2R, LRRC1, NENF, NFATC2, PSMD12, RAB9A, RUFY2, SMAP2, USP39, WDPCP, ZMYM4, ZMYM5, BCL2, C19ORF40, C6ORF130, DACT2, DEGS1, HEMK1, LRP1, MAN1A2, NR2C2, PEX3, POLE2, PPAT, REV3L, RRS1, SERPINB1, SLC22A5, ZDHHC18, ZNF180, ZNF627, ZNF704, ZNF75A, AKAP11, C16ORF70, CBR4, CNEP1R1, FUT8, GTF3C4, MEX3D, PDXDC1, REST, TAF15, TNFRSF21, UBTD2, USP38, ZNF510, CAPRIN2, DOCK7, ELF2, HIATL1, IKBKB, MBTPS1, NAA40, RMND5A, RNASEH2B, SCNN1B, SEC63, VPS4B, ZBED4, AGFG1, AMIGO2, CCNL1, FAT1, FOPNL, LAMTOR3, NAB2, PKNOX1, PLEKHA5, PNPLA4, PPP2R3C, RNPC3, SERTAD4, SUDS3, TBC1D10B, VPS29, ZNF236, ZNF318, ZNF800, BACE2, BRF1, CENPBD1, CTDSPL2, CWF19L1, DYRK1A, GLE1, MYO6, NPC1, PCNX, PLEKHA1, RRAGA, SEC61A2, SLC1A1, TMEM68, TRIM4, TWSG1, UBE2D1, ZBTB25, ANGEL2, CASP3, CYB5D1, FLVCR1, GTF2A1, ICMT, KIAA1804, NIF3L1, NIPSNAP3A, PRKCI, RAP1GDS1, SETX, STRN, ZRANB3, CHCHD1, PPIC, SLTM, TBC1D2, ACVR2A, C1ORF27, CCNT1, DPP8, E2F3, E2F7, ELL, KDM6A, KIF11, KIF1A, LPAR1, MED28, OSBPL1A, PRKD2, PTPN12, RBM17, RPA1, RPRD1B, SESTD1, SPRED2, ENC1, USP4, CCNE1, CSTF3, DIABLO, EPN2-IT1, GPBP1, KDELC2, KLHL21, NFYA, PALB2, PASK, RAB27A, RAP2B, SAT1, SLC25A30, SPRED1, ANKRD5, HNRNPH3, ING2, LEMD3, LRP12, POLR3E, PRKACA, SMEK1, CDK12, DNAJC1, MFSD12, NDEL1, PRRG4, SPATA5, SRPK2, SRR, ZNF460, AMPD2, VPS54, ZNF444, ITPKC, KAT6A, MND1, NSD1, PANX1, RAB26, USP37, C16ORF87, IQSEC1, SEZ6L2, SLC23A2, SOCS5, IL10RB, RFT1, RPS15A, TTC14, ZNF91, ARNT2, ATAD5, NDUFAF1, NRARP, OLFM2, ACAA2, HIVEP1, KBTBD7, MIER3, USP1, CHST14, DAPK2, PTEN, BEND7, ATP1B1, EXOC2, IL6ST, UBAP1, PRR15 |
| 6.60e-05 | TF:M00800_4 | tf | Factor: AP-2; motif: GSCCSCRGGCNRNRNN; match class: 4 | ARHGEF7, CCNE2, OGDHL, TMTC3, ZG16B, ARL4D, LZTR1, PPP1R15B, PTPRZ1, SDF4, GNA13, KBTBD2, LRIG1, PTRF, RUSC2, SLC35G1, TXNDC11, ATF4, CCDC8, CNOT1, EPN1, GPC3, MMGT1, NKX2-3, NT5M, RAB11FIP5, TADA3, AXDND1, BCAM, C3ORF37, DCAF11, FLII, FMNL2, FOXK2, GTF2E1, IFI27L1, MDM2, NDUFS2, OAT, PEX2, PIM2, UBE2J1, USP10, ZNF219, ARF6, B3GAT3, COMMD4, CREB3L4, CSPG5, CTDNEP1, GPCPD1, KCNIP1, PSMD10, SLC27A4, SLC35E1, TMEM230, USP36, ZBTB46, ATL3, BICD2, CAMTA2, CDC42EP4, DDA1, KDELR2, MMS19, NUP210, PSENEN, R3HDM1, SBF1, TRADD, TRAPPC11, AACS, BYSL, C12ORF10, CEP112, FOXK1, GFER, NIPAL1, PLXND1, RSF1, RTN2, SAMHD1, SORBS3, ST8SIA3, TBC1D9B, TBX3, TNK1, UBE2I, WDR83, ABL1, BAIAP2L1, C11ORF58, C17ORF48, CKAP5, DNAJB1, DYNLL1, GNG12, GPC4, HSPA9, IKZF5, KCNE1L, LPCAT4, MAN2A1, NOL11, OSBPL7, PAK3, POLA2, PPP1R26, PTGER4, SPSB1, SYT17, UBXN2A, VAV3, ZDHHC8, ANKRD27, APBB2, C9ORF116, CCDC18, COL4A1, ENOPH1, EPB49, GLRX3, GRSF1, GTSE1, IRF1, LIMCH1, LRP5, MED13, MMP9, NAPEPLD, NCAPG2, NFATC3, OSBPL2, PTCH1, RBM15, SDCCAG3, SLC25A19, SLC2A4RG, TCEAL2, TMEM18, TPM4, TRIM65, WDR17, VEZF1, WFS1, ZNHIT1, ZNRF2, AEBP2, ATE1, CHMP7, CISD2, CLDND1, COX7C, DNM3, DRAM2, LNX2, NARG2, PAF1, SLC25A5, SLC30A1, STAU1, TAB2, TIPARP, TMEM57, TMEM82, ADCY1, AMD1, ATP5C1, B4GALT6, BUB3, CAMTA1, CARKD, CPT1A, F7, ITSN2, LUC7L3, MED31, MOCS3, PDCL, PPFIA1, SETD7, USP19, WDSUB1, VPS33B, AAK1, CCNG2, DAG1, DONSON, HECW2, JARID2, KCMF1, KREMEN1, LACTB2, LPIN3, MARC1, NME3, ODC1, PCDH1, PHAX, PLCG1, RABL6, RMI1, TIMM21, USP21, ALG12, ATAD2, C2ORF55, COX7A2L, DCAF12, DERL1, GSK3B, KCNK5, KIF1B, KRT17, LGALSL, MVP, NAE1, NDRG3, NUP155, OSBP2, PCBP4, PPAP2A, PRPF38A, PXK, RGL1, SERAC1, TACC2, TRH, UAP1, ANAPC4, DNER, ETNK2, KIF23, NSUN2, PCGF3, PELI3, PLXDC2, RASGEF1A, RCN2, RIN2, RSBN1, SH3BP4, ARAP2, ARHGAP5, ATXN7, C18ORF25, C18ORF8, C1ORF122, DHX15, ERC1, EZH2, GAS6, GCC2, KCNMB4, KLK7, MLH3, MOB1B, PARP4, PHLPP1, PKP4, RNF152, RNF214, SSH3, VPS33A, ZNF584, ZNF621, ACSL3, APLP2, BAG5, BCL11B, DENND1A, FNBP1, HTRA2, INPP5D, IRF3, MRPL48, NCK2, NEDD1, NIP7, PITPNA, PSMD1, STRA13, TDRD3, TMEM209, TUSC3, VDR, YWHAH, AP1B1, ARMC9, CHDH, CYFIP2, EEA1, FHL2, HSCB, ME2, MKI67, MPP6, OXR1, PRR15L, RANBP9, RBPJ, RRAGD, SPATA7, TCP11L1, TSSC1, UFSP2, ZFC3H1, AMMECR1L, CYLD, DIDO1, ERI1, EXOSC10, FASTKD2, FRRS1, IGF2R, LRRC1, NENF, NFATC2, NUDCD1, PSMD12, RAB9A, RSPH1, RUFY2, SMAP2, USP39, ZMYM4, ZMYM5, BCL2, C19ORF40, C6ORF130, CIB1, DACT2, DEGS1, HEMK1, HERC1, JAGN1, LRP1, MAN1A2, NR2C2, PPAT, PPP1R16A, REV3L, SERPINB1, SLC22A5, ZDHHC18, ZNF180, ZNF627, ZNF75A, AKAP11, C16ORF70, CBR4, CNEP1R1, CPEB4, DIS3, FUT8, GTF3C4, MEX3D, MGRN1, PDXDC1, REST, TAF15, UBQLN4, UBTD2, CAPRIN2, CDCP1, ELF2, FIG4, HSD3B7, IKBKB, MFAP3L, NAA40, NUP160, RMND5A, RNASEH2B, SREBF1, VPS4B, ZBED4, AMIGO2, FAT1, GPATCH2, LAMTOR3, NAB2, PKNOX1, PLEKHA5, PPP2R3C, PRPS2, SERTAD4, STARD3NL, SUDS3, TBC1D10B, ZNF236, ZNF800, BRF1, CENPBD1, CTDSPL2, DGUOK, DYRK1A, GLE1, LAMP2, MYO6, NPC1, PLEKHA1, PVRL4, SEC61A2, TMEM68, TRIM4, TWSG1, UHRF1BP1L, ZBTB25, CASP3, CYB5D1, FLVCR1, GTF2A1, HDAC1, NIF3L1, RAP1GDS1, SETX, STRN, ZRANB3, CAMSAP1, CHCHD1, DDX18, MB21D2, NTAN1, PPIC, RTTN, TBC1D2, ACVR2A, C1ORF27, E2F3, E2F7, ELL, KDM6A, KIF11, KIF1A, LIF, LPAR1, MED28, OSBPL1A, PHF16, PRKD2, PTPN12, RBM17, RPA1, RPRD1B, SESTD1, TMEM30B, ZBTB39, ENC1, TOR1A, USP4, CCNE1, CSTF3, DIABLO, EPN2-IT1, KDELC2, KLHL21, NFYA, PASK, RAB27A, RAP2B, SAT1, SLC25A30, SPRED1, ALMS1, CEBPG, DCAF16, FUCA1, ING2, LEMD3, LPCAT2, LRP12, PRKACA, SMEK1, CDK12, DNAJC1, MFSD12, NDEL1, PRRG4, SIDT1, SPATA5, SRPK2, SRR, ZNF460, AMPD2, VPS54, ZNF444, ITPKC, KAT6A, MND1, NSD1, RAB26, USP37, C14ORF118, C16ORF87, PPIP5K1, SEZ6L2, SLC23A2, TIA1, IL10RB, MTHFR, PCDHB5, ZNF91, ARNT2, NDUFAF1, HACE1, NRARP, OLFM2, ZNF484, ACAA2, HIVEP1, KBTBD7, MIER3, TTBK2, USP1, CHST14, PTEN, BEND7, ATP1B1, BTBD1, EXOC2, IL6ST, SPATA2, EMP2, RPGRIP1L |
| 1.49e-03 | TF:M00189_4 | tf | Factor: AP-2; motif: MKCCCSCNGGCG; match class: 4 | ARHGEF7, OGDHL, POMC, TMTC3, ZG16B, BCS1L, PPP1R15B, SDF4, GNA13, KBTBD2, LRIG1, RUSC2, SLC35G1, TXNDC11, ATF4, FGF5, GPC3, MMGT1, NT5M, AXDND1, DCAF11, FLII, IFI27L1, IL17RA, MDM2, NDUFS2, OAT, PEX2, RNF141, SCD, TAGAP, UBE2J1, USP10, ARF6, COMMD4, CREB3L4, CTDNEP1, DCTPP1, GPCPD1, PSMD10, SUPT7L, ZBTB49, ATXN10, BICD2, CDC42EP4, MMS19, NOLC1, NUP210, PSENEN, TRADD, TRAPPC11, AACS, CEP112, DRG1, FOXK1, GFER, NIPAL1, PLXND1, R3HCC1L, RTN2, SORBS3, TBC1D9B, TNK1, UBE2I, WDR83, ABL1, BAIAP2L1, C11ORF58, C17ORF48, DNAJB1, DYNLL1, GPC4, HSPA9, IKZF5, KCNE1L, LPCAT4, LRTOMT, MAN2A1, PAK3, PPP1R26, PTGER4, SPSB1, SYT17, TSEN15, ZDHHC8, ANKRD27, APBB2, CCDC18, COL4A1, EPB49, FANCD2, GLRX3, GRSF1, IRF1, LIMCH1, LRP5, MED13, MMP9, MOB1A, OSBPL2, PTCH1, RBM15, SDCCAG3, SLC2A4RG, TMEM106B, TPM4, TRIM65, WDR17, VEZF1, YDJC, ZNHIT1, ATE1, BRPF1, CHMP7, CISD2, DNM3, DRAM2, FN3KRP, KCND2, SERPINE1, SLC30A1, TAB2, TIPARP, TMEM57, TMEM82, ADCY1, ATP5C1, B4GALT6, CAMTA1, ITSN2, LUC7L3, PPFIA1, SETD7, WDSUB1, AKIRIN2, CHMP4B, HECW2, JARID2, KCMF1, KREMEN1, LACTB2, LPIN3, MARC1, NME3, ODC1, PLCG1, TIMM21, ACSM3, ALG12, ARRDC4, C2ORF55, COX7A2L, DERL1, GRTP1, GYG1, HNRNPF, KIF1B, OSBP2, PCBP4, PRPF4, PRRT3, PXK, RGL1, SERAC1, TRH, ANAPC4, DNER, ETNK2, PCGF3, PCIF1, PLXDC2, RASGEF1A, RIN2, RSBN1, SH3BP4, SNX11, ARAP2, ARHGAP5, ATXN7, BAP1, C18ORF25, C18ORF8, DHX15, ERC1, GCC2, KCNMB4, KLK7, MKRN2, RNF152, RNF214, ZNF621, APLP2, BAG5, DENND1A, FNBP1, HSPA14, HTRA2, NIP7, PITPNA, PSMD1, TDRD3, USP9X, CHDH, CYFIP2, EEA1, FHL2, ME2, MKI67, PARN, RANBP9, RBPJ, RRAGD, SETDB1, SPATA7, TCP11L1, TSSC1, TTK, UFSP2, CRKL, DIDO1, FASTKD2, NENF, NFATC2, PSMD12, RSPH1, RUFY2, SMAP2, ZMYM5, BCL2, C6ORF130, CIB1, DACT2, DEGS1, HEMK1, HERC1, JAGN1, LRP1, NR2C2, PPAT, PPP1R16A, REV3L, ZNF180, ZNF75A, AKAP11, C16ORF70, CBR4, CPEB4, FUT8, MEX3D, PDXDC1, REST, UBTD2, USP38, CAPRIN2, DOCK7, ELF2, HSD3B7, MBTPS1, MFAP3L, RMND5A, RNASEH2B, SCNN1B, ZBED4, AMIGO2, CCNL1, FAT1, ICT1, NAB2, PKNOX1, PLEKHA5, PPP2R3C, PRPS2, TBC1D10B, TXNDC12, ZNF318, ZNF800, BACE2, BRF1, CTDSPL2, DYRK1A, GLE1, LAMP2, MYO6, NPC1, PCNX, PVRL4, SEC61A2, TMEM68, TWSG1, FLVCR1, ICMT, KIAA1804, RAP1GDS1, SETX, ZRANB3, DDX18, MB21D2, NTAN1, RTTN, C1ORF27, DPP8, E2F3, E2F7, ELL, KIF1A, LPAR1, PRKD2, PTPN12, RBM17, RPA1, RPRD1B, SPRED2, ZBTB39, TYW5, USP4, DIABLO, EPN2-IT1, GPBP1, KDELC2, PASK, RAB27A, SAT1, SPRED1, ALMS1, CEBPG, FUCA1, HNRNPH3, ING2, LRP12, PRKACA, SMEK1, DNAJC1, PRRG4, SRPK2, SRR, AMPD2, VPS54, ITPKC, KAT6A, MND1, NSD1, RAB26, USP37, IQSEC1, SEZ6L2, ARNT2, ATAD5, NRARP, OLFM2, ZNF484, ACAA2, HIVEP1, KBTBD7, MIER3, USP1, PTEN, BTBD1, IL6ST, EMP2 |
| 1.11e-02 | TF:M00008_3 | tf | Factor: Sp1; motif: GGGGCGGGGT; match class: 3 | ARHGEF7, CCNE2, OGDHL, TMTC3, ZG16B, BCS1L, PPP1R15B, PTPRZ1, RBM18, CACNB4, GNA13, KBTBD2, LRIG1, PTRF, RPL7L1, TXNDC11, XKR6, ATF4, CYR61, EPN1, FGF5, GPC3, MMGT1, NKX2-3, NT5M, RAB11FIP5, TADA3, BCAM, C3ORF37, CDKN3, CRTC2, DCAF11, FLII, FMNL2, MDM2, NCOA6, NDUFS2, NIPAL3, NPTN, OAT, PIM2, RNF141, SCD, UBE2J1, ZNF219, ALDH16A1, ARF6, B3GAT3, COMMD4, CSPG5, CTDNEP1, GPCPD1, KCNIP1, RFC4, SLC35E1, SOAT1, UGGT1, USP36, AHCY, ATL3, CAMTA2, DDA1, KDELR2, MMS19, NUP210, PSENEN, R3HDM1, SBF1, TMTC1, TSG101, AACS, BYSL, CEP112, DRG1, FOXK1, GFER, HSPH1, NIPAL1, PLXND1, RPL37A, RSF1, RTN2, SCML1, SEC13, SORBS3, TBC1D9B, TBX3, TNK1, UBE2I, WDR83, ABL1, BAIAP2L1, C17ORF48, DNAJB1, DYNLL1, GCA, GNG12, GPC4, HSPA9, IKZF5, KCNE1L, LPCAT4, LRTOMT, MAN2A1, OSBPL7, PAK3, PPP1R26, PTGER4, SPSB1, SPTY2D1, TRMT6, UBXN2A, VAV3, ZDHHC8, ANKRD27, C14ORF126, ENOPH1, EPB49, ERBB3, GLRX3, GRSF1, GTSE1, IDH3A, IRF1, LAP3, LIMCH1, LRP5, NAPEPLD, NCAPG2, NFATC3, OSBPL2, PTCH1, RBM15, RBM23, RSL24D1, SDCCAG3, SLC25A19, SLC2A4RG, SLC7A1, TPM4, TRIM65, WDR17, VEZF1, WFS1, YDJC, ZNHIT1, AEBP2, ATE1, BRPF1, CHMP7, CISD2, CLDND1, DNM3, FN3KRP, GHITM, KCND2, LNX2, NAGLU, NARG2, SLC25A5, SLC30A1, TMEM57, WDR45L, ADCY1, AMD1, ARGLU1, ATP5C1, B4GALT6, BUB1, CAMTA1, CPT1A, ITSN2, LUC7L3, MED31, PPFIA1, SETD7, WDSUB1, AAGAB, CCNG2, DAG1, DONSON, HECW2, KCMF1, KREMEN1, LACTB2, LPIN3, NME3, ODC1, PCDH1, PLCG1, RMI1, STIM2, USP21, ALG12, ARRDC4, C20ORF3, C2ORF55, COX7A2L, HNRNPF, KCNK5, KIF1B, KRT17, LGALSL, NDRG3, NUP155, OSBP2, PCBP4, PPAP2A, PRPF38A, RGL1, SERAC1, TACC2, TRH, UAP1, ANAPC4, DNER, ETNK2, ILF3, NSUN2, PCGF3, PCIF1, PELI3, PLXDC2, RIN2, RSBN1, SH3BP4, SLC25A33, ADNP2, ARAP2, ARHGAP5, ARL6, BAP1, C18ORF8, C1ORF122, C4ORF46, ERC1, EZH2, HEXA, KCNMB4, KLK7, MOB1B, NEK4, PARP4, PHLPP1, RNF152, SSH3, ZNF584, ZNF621, ACSL3, APLP2, BAG5, BCL11B, DENND1A, FNBP1, INPP5D, IRF3, NCK2, NEDD1, PITPNA, PSMD1, PTPLAD1, SLC24A6, STRA13, TMEM9, TWF2, USP9X, VDR, YWHAH, ZDHHC4, AP1B1, CYFIP2, EEA1, FHL2, FOXJ3, GMNN, HSCB, ME2, MKI67, MPP6, OXR1, PARN, PRR15L, RBPJ, RRAGD, RSRC2, SRD5A3, TCP11L1, TNNC2, TTK, UFSP2, ZFC3H1, CYLD, DHX35, DIDO1, HLCS, IGF2R, NENF, NFATC2, NUDCD1, RAB9A, RSPH1, RUFY2, SMAP2, SPINT2, WDPCP, ZMYM5, ZNF267, BCL2, C19ORF40, CIB1, DACT2, DEGS1, HEMK1, HERC1, JAGN1, LRP1, MAN1A2, NR2C2, PPAT, PPP1R16A, REV3L, SLC22A5, ZNF180, ZNF627, EXPH5, FUT8, GTF3C4, LMTK2, MEX3D, PDXDC1, REST, TES, TNFRSF21, UBQLN4, UBTD2, AKAP13, ALG2, CAPRIN2, FIG4, HSD3B7, IKBKB, MBTPS1, NAA40, NUP160, RNASEH2B, SCNN1B, SEC63, SREBF1, ZBED4, ANKRD49, ATG14, FAT1, FOPNL, LAMTOR3, NAB2, PKNOX1, PRPS2, RNPC3, SERTAD4, TBC1D10B, TXNDC12, VPS29, ZNF318, ZNF800, BACE2, BRF1, CTDSPL2, DGUOK, GLE1, LAMP2, MYO6, NPC1, PLEKHA1, PSMB1, SEC61A2, TWSG1, UBE2D1, UHRF1BP1L, CASP3, CYB5D1, GTF2A1, ICMT, KIAA1804, LPGAT1, NIPSNAP3A, RAP1GDS1, SETX, STRN, ZRANB3, CHCHD1, ESD, GPR87, MB21D2, NTAN1, PPIC, PTGES, RTTN, TBC1D2, ACVR2A, C1ORF27, E2F3, E2F7, ELL, KDM6A, KIF11, KIF1A, LPAR1, OSBPL1A, PHF16, PRKD2, PTPN12, RPA1, SESTD1, ZBTB39, ENC1, MTMR3, PEX13, SMC1A, TYW5, USP4, CCNE1, CSTF3, DIABLO, EPN2-IT1, GPBP1, KDELC2, KLHL21, MAGEC2, NFYA, PJA2, RAB27A, RAP2B, SAT1, SLC25A30, SPRED1, ANKRD5, HNRNPH3, ING2, POLR3E, PRKACA, C6ORF57, MFSD12, NDEL1, SRPK2, SRR, ZNF460, AMPD2, VPS54, ITPKC, KAT6A, MND1, NEK7, PANX1, RAB26, TFB1M, USP37, C16ORF87, IQSEC1, SEZ6L2, SLC23A2, SOCS5, IL10RB, RFT1, MTHFR, ZNF543, TRIM38, NRARP, OLFM2, ACAA2, HIVEP1, KBTBD7, USP1, CHST14, DAPK2, KATNAL2, PTEN, ATP1B1, CEACAM5, IL6ST, MT1X, TTC30A, RPGRIP1L |
| 1.46e-02 | TF:M00427_4 | tf | Factor: E2F; motif: TTTSGCGS; match class: 4 | ARHGEF7, POMC, TMTC3, SDF4, CACNB4, KBTBD2, RPL7L1, CNOT1, MMGT1, RAB11FIP5, TADA3, AXDND1, MDM2, NIPAL3, PIM2, ARF6, CREB3L4, DCTPP1, TMEM230, USP36, ATL3, MMS19, NAT10, NUP210, TRAPPC11, CEP112, HSPH1, NIPAL1, SAMHD1, SORBS3, TBC1D9B, TNK1, TRIP4, ABL1, C11ORF58, C17ORF48, DYNLL1, GCA, GPC4, IKZF5, LRTOMT, MANBAL, MON1B, POLA2, TRMT6, TSEN15, ZNF227, COL4A1, IDH3A, LRP5, RBM23, SLC25A19, SLC7A1, VEZF1, ZNHIT1, ATE1, COX7C, DRAM2, KCND2, LNX2, OSBPL10, SERPINE1, SLC30A1, STAU1, TIMM9, TIPARP, ARGLU1, ATP5C1, B4GALT6, BUB3, COIL, MOCS3, PDCL, SETD7, USP19, WDSUB1, DONSON, HECW2, LPAR3, LPIN3, ODC1, RABL6, RMI1, TIMM21, USP21, UTP11L, ZNF418, ALG12, ATAD2, GYG1, KCNK5, KIF1B, KRT17, NUP155, OSBP2, PRPF38A, RGL1, DNER, ILF3, NSUN2, PELI3, PLXDC2, RSBN1, ARHGAP5, ARL6, C18ORF25, ERC1, KCNMB4, RNF152, TGOLN2, C10ORF12, HSPA14, INPP5D, NIP7, PSMD1, PTPLAD1, TDRD3, TMEM209, USP9X, CHDH, FBXW8, FHL2, FOXJ3, HSCB, CRKL, CYLD, HLCS, NENF, NFATC2, RUFY2, WDPCP, ZNF267, C6ORF130, MAN1A2, MRPS10, POLE2, PPAT, AKAP11, C16ORF70, EXPH5, FUT8, MEX3D, CAPRIN2, MBTPS1, MFAP3L, NUP160, VBP1, AMIGO2, NAB2, PLEKHA5, PPP2R3C, PRPS2, TBC1D10B, VPS29, CTDSPL2, GLE1, PVRL4, SEC61A2, SLC1A1, TWSG1, CYB5D1, GTF2A1, ICMT, DDX18, GUF1, E2F3, E2F7, KIF11, LPAR1, OSBPL1A, RPA1, MTMR3, CSTF3, KDELC2, KLHL21, NFYA, PALB2, SAT1, SPRED1, ANKRD5, CEBPG, LRP12, POLR3E, C6ORF57, AMPD2, VPS54, ZNF444, MND1, C16ORF87, IQSEC1, SEZ6L2, RFT1, RPS15A, TTC14, ATAD5, ACAA2, KBTBD7, USP1, PYDC1, BTBD1, NAA16, UBAP1, RPGRIP1L |
| 2.25e-03 | TF:M00470_4 | tf | Factor: AP-2gamma; motif: GCCYNNGGS; match class: 4 | ARHGEF7, CCNE2, ZG16B, BCS1L, LZTR1, PTPRZ1, SDF4, LRIG1, SLC35G1, CCDC8, EPN1, NT5M, C3ORF37, FLII, MDM2, PEX2, SCD, UBE2J1, USP10, CSPG5, ZBTB49, BICD2, KDELR2, NUP210, SBF1, TRADD, TRAPPC11, CEP112, NIPAL1, SORBS3, TBX3, TNK1, UBE2I, WDR83, ABL1, BAIAP2L1, C11ORF58, DYNLL1, GNG12, HSPA9, PAK3, PPP1R26, PTGER4, SYT17, ZDHHC8, C9ORF116, COL4A1, ENOPH1, IRF1, LIMCH1, LRP5, MED13, OSBPL2, PTCH1, RBM15, SLC2A4RG, TCEAL2, TPM4, TRIM65, WDR17, VEZF1, WFS1, ZNHIT1, ATE1, CLDND1, DNM3, DRAM2, KCND2, LNX2, TAB2, TMEM57, ADCY1, ATP5C1, CPT1A, MED31, PPFIA1, WDSUB1, BBS2, JARID2, KCMF1, KREMEN1, MARC1, TIMM21, GYG1, KCNK5, KIF1B, KRT17, LGALSL, PRPF4, TACC2, UAP1, NSUN2, ATXN7, C1ORF122, ERC1, KCNMB4, PKP4, ACSL3, APLP2, BCL11B, FNBP1, CHDH, FHL2, ME2, RANBP9, RBPJ, AMMECR1L, NENF, RSPH1, RUFY2, ZMYM5, C6ORF130, CIB1, DACT2, LRP1, NR2C2, PPP1R16A, REV3L, ZNF180, ZNF627, AKAP11, CNEP1R1, MEX3D, UBQLN4, UBTD2, CAPRIN2, IKBKB, RMND5A, RNASEH2B, AMIGO2, COQ9, PLEKHA5, TBC1D10B, ZNF318, DYRK1A, GLE1, NPC1, PCNX, KIAA1804, SETX, STRN, ZRANB3, MB21D2, NTAN1, E2F3, KIF1A, LPAR1, PRKD2, TMEM30B, ENC1, KDELC2, PASK, RAP2B, SAT1, DCAF16, ING2, CDK12, MFSD12, SPATA5, SRPK2, SRR, ITPKC, KAT6A, PANX1, SLC23A2, ARNT2, NRARP, ACAA2, HIVEP1, USP1, PTEN, EMP2 |
| 1.61e-04 | TF:M00931_4 | tf | Factor: Sp1; motif: GGGGCGGGGC; match class: 4 | OGDHL, TMTC3, BCS1L, PPP1R15B, PTPRZ1, RBM18, GNA13, KBTBD2, LRIG1, PTRF, TXNDC11, XKR6, ATF4, CYR61, EPN1, FGF5, GPC3, MMGT1, NKX2-3, NT5M, RAB11FIP5, TADA3, BCAM, C3ORF37, DCAF11, FLII, FMNL2, NCOA6, NPTN, OAT, PIM2, RNF141, SCD, UBE2J1, ALDH16A1, ARF6, B3GAT3, COMMD4, CSPG5, CTDNEP1, GPCPD1, KCNIP1, RFC4, SLC35E1, SOAT1, UGGT1, USP36, AHCY, ATL3, ATXN10, CAMTA2, DDA1, KDELR2, MMS19, NUP210, PSENEN, R3HDM1, SBF1, TMTC1, AACS, BYSL, CEP112, DRG1, FOXK1, GFER, NIPAL1, PLXND1, RSF1, RTN2, SCML1, SEC13, SORBS3, TBC1D9B, TBX3, TNK1, UBE2I, WDR83, ABL1, BAIAP2L1, DNAJB1, DYNLL1, GCA, GNG12, GPC4, HSPA9, IKZF5, KCNE1L, LPCAT4, LRTOMT, MAN2A1, OSBPL7, PPP1R26, PTGER4, SPSB1, TRMT6, UBXN2A, VAV3, ZDHHC8, ANKRD27, C14ORF126, ENOPH1, ERBB3, GLRX3, GRSF1, IDH3A, IRF1, LAP3, LIMCH1, LRP5, NCAPG2, NFATC3, OSBPL2, PTCH1, RBM15, RBM23, SDCCAG3, SLC2A4RG, SLC7A1, TPM4, TRIM65, WDR17, VEZF1, WFS1, YDJC, ZNHIT1, AEBP2, ATE1, BRPF1, CISD2, CLDND1, DNM3, FN3KRP, GHITM, KCND2, LNX2, NARG2, SLC25A5, SLC30A1, STAU1, TMEM57, WDR45L, ADCY1, ATP5C1, B4GALT6, CAMTA1, CPT1A, ITSN2, LUC7L3, MED31, PPFIA1, SETD7, USP19, WDSUB1, AAGAB, CCNG2, DAG1, KCMF1, KREMEN1, LACTB2, LPIN3, NME3, ODC1, PCDH1, PLCG1, PPP2R5E, RABL6, RMI1, STIM2, ALG12, ARRDC4, C20ORF3, C2ORF55, COX7A2L, HNRNPF, KCNK5, KIF1B, KRT17, LGALSL, NDRG3, OSBP2, PCBP4, PPAP2A, PRPF38A, RGL1, SERAC1, TRH, UAP1, ANAPC4, DNER, ETNK2, ILF3, NSUN2, PCGF3, PELI3, PLXDC2, RSBN1, SH3BP4, SLC25A33, ADNP2, ARAP2, ARHGAP5, ATXN7, BAP1, C18ORF8, C4ORF46, COG2, ERC1, EZH2, GAS6, KCNMB4, KLK7, PARP4, PHLPP1, RNF152, SSH3, ZNF584, ZNF621, ACSL3, APLP2, BAG5, BCL11B, DENND1A, FNBP1, HTRA2, NCK2, NEDD1, PITPNA, PSMD1, PTPLAD1, SLC24A6, STRA13, TMEM9, TWF2, VDR, YWHAH, AP1B1, CYFIP2, EEA1, FHL2, FOXJ3, GMNN, ME2, MKI67, MPP6, OXR1, PARN, RANBP9, RBPJ, RRAGD, RSRC2, SRD5A3, UFSP2, ZFC3H1, AMMECR1L, CYLD, DHX35, DIDO1, EXOSC10, IGF2R, NENF, NFATC2, NUDCD1, RAB9A, RSPH1, RUFY2, SMAP2, SPINT2, WDPCP, ZNF267, ATXN2, C6ORF130, DACT2, DEGS1, HERC1, JAGN1, LRP1, MAN1A2, PPAT, PPP1R16A, REV3L, SERPINB1, SLC22A5, ZNF180, ZNF627, AKAP11, CNEP1R1, EXPH5, FUT8, GTF3C4, LMTK2, MEX3D, PDXDC1, REST, TNFRSF21, UBQLN4, UBTD2, AKAP13, ALG2, CAPRIN2, FIG4, IKBKB, NAA40, NUP160, RNASEH2B, SCNN1B, SEC63, SREBF1, ZBED4, ANKRD49, FAT1, FOPNL, ICT1, LAMTOR3, NAB2, PKNOX1, PRPS2, RNPC3, SERTAD4, TXNDC12, ZNF318, ZNF800, BACE2, BRF1, DYRK1A, LAMP2, MYO6, NPC1, PSMB1, SEC61A2, TWSG1, UBE2D1, UHRF1BP1L, ZBTB25, CASP3, CYB5D1, GTF2A1, HDAC1, ICMT, KIAA1804, LPGAT1, NIPSNAP3A, RAP1GDS1, SETX, STRN, ZRANB3, CHCHD1, NTAN1, PPIC, PTGES, TBC1D2, ACVR2A, C1ORF27, DPP8, E2F3, ELL, KDM6A, KIF11, KIF1A, LPAR1, OSBPL1A, PHF16, PPIG, PTPN12, SESTD1, SPRED2, MTMR3, PEX13, SMC1A, USP4, CCNE1, CSTF3, DIABLO, EPN2-IT1, GPBP1, KDELC2, KLHL21, MAGEC2, NFYA, PJA2, RAB27A, RAP2B, SLC25A30, SPRED1, ANKRD5, HNRNPH3, ING2, POLR3E, PRKACA, C6ORF57, DNAJC1, MFSD12, NDEL1, SRPK2, SRR, AMPD2, VPS54, ITPKC, KAT6A, NSD1, PANX1, RAB26, USP37, C16ORF87, IQSEC1, SLC23A2, SOCS5, IL10RB, RFT1, NRARP, OLFM2, ACAA2, HIVEP1, USP1, CHST14, PTEN, ATP1B1, IL6ST |
| 7.58e-05 | TF:M00932_4 | tf | Factor: Sp1; motif: NNGGGGCGGGGNN; match class: 4 | ARHGEF7, OGDHL, TMTC3, BCS1L, HECA, PPP1R15B, PTPRZ1, RBM18, GNA13, KBTBD2, LRIG1, PTRF, TXNDC11, XKR6, ATF4, CYR61, EPN1, FGF5, GPC3, MMGT1, NKX2-3, NT5M, RAB11FIP5, RNF111, TADA3, BCAM, C3ORF37, DCAF11, FLII, FMNL2, NCOA6, NDUFS2, NPTN, OAT, PIM2, RNF141, SCD, UBE2J1, ZNF219, ALDH16A1, ARF6, B3GAT3, COMMD4, CSPG5, CTDNEP1, GPCPD1, KCNIP1, RFC4, SLC35E1, SOAT1, UGGT1, USP36, AHCY, ATL3, ATXN10, CAMTA2, DDA1, KDELR2, MMS19, NUP210, PSENEN, R3HDM1, SBF1, TMTC1, AACS, BYSL, CEP112, DRG1, FOXK1, GFER, NIPAL1, PLXND1, RSF1, RTN2, SCML1, SEC13, SORBS3, TBC1D9B, TBX3, TNK1, UBE2I, WDR83, ABL1, BAIAP2L1, DNAJB1, DYNLL1, GCA, GNG12, GPC4, IKZF5, KCNE1L, LPCAT4, LRTOMT, MAN2A1, OSBPL7, PPP1R26, PTGER4, SPSB1, TRMT6, UBXN2A, VAV3, ZDHHC8, ANKRD27, C14ORF126, COL4A1, ENOPH1, EPB49, ERBB3, GLRX3, GRSF1, IDH3A, IRF1, LAP3, LIMCH1, LRP5, NCAPG2, NFATC3, OSBPL2, PTCH1, RBM15, RBM23, SDCCAG3, SLC2A4RG, SLC7A1, TPM4, TRIM65, WDR17, VEZF1, WFS1, YDJC, ZNHIT1, AEBP2, ATE1, BRPF1, CISD2, CLDND1, DNM3, FN3KRP, GHITM, KCND2, LNX2, NARG2, SLC25A5, SLC30A1, STAU1, TMEM57, WDR45L, ADCY1, ATP5C1, B4GALT6, CAMTA1, CPT1A, ITSN2, LUC7L3, MED31, PPFIA1, SETD7, USP19, WDSUB1, AAGAB, AAK1, AR, CCNG2, DAG1, JARID2, KCMF1, KREMEN1, LACTB2, LPIN3, NME3, ODC1, PCDH1, PLCG1, RABL6, RMI1, STIM2, ALG12, ARRDC4, C20ORF3, C2ORF55, COX7A2L, GSK3B, HNRNPF, KCNK5, KIF1B, KRT17, LGALSL, NDRG3, OSBP2, PCBP4, PPAP2A, PRPF38A, RGL1, SERAC1, TRH, UAP1, ANAPC4, DNER, ETNK2, ILF3, NSUN2, PCGF3, PELI3, PLXDC2, RSBN1, SH3BP4, SLC25A33, ADNP2, ARAP2, ARHGAP5, ATXN7, BAP1, C18ORF8, C4ORF46, COG2, ERC1, EZH2, GAS6, KCNMB4, KLK7, MUM1L1, PARP4, PHLPP1, RNF152, RNF214, SSH3, ZNF584, ZNF621, ACSL3, APLP2, BAG5, BCL11B, DENND1A, FNBP1, HTRA2, INPP5D, NCK2, NEDD1, PITPNA, PSMD1, PTPLAD1, SLC24A6, STRA13, TMEM9, TWF2, USP9X, VDR, YWHAH, AP1B1, CYFIP2, EEA1, FHL2, FOXJ3, GMNN, ME2, MKI67, MPP6, OXR1, PARN, RANBP9, RBPJ, RRAGD, RSRC2, SRD5A3, UFSP2, ZFC3H1, AMMECR1L, CYLD, DHX35, DIDO1, EXOSC10, IGF2R, NENF, NFATC2, NUDCD1, PSMD12, RAB9A, RSPH1, RUFY2, SMAP2, SPINT2, WDPCP, ZNF267, ATXN2, C6ORF130, CIB1, DACT2, DEGS1, HERC1, JAGN1, LRP1, MAN1A2, PPAT, PPP1R16A, REV3L, SERPINB1, SLC22A5, ZNF180, ZNF627, AKAP11, CNEP1R1, EXPH5, FUT8, GTF3C4, LMTK2, MEX3D, PDXDC1, REST, TNFRSF21, UBQLN4, UBTD2, AKAP13, ALG2, CAPRIN2, FIG4, IKBKB, NAA40, NUP160, RNASEH2B, SCNN1B, SEC63, SREBF1, ZBED4, ANKRD49, FAT1, FOPNL, ICT1, LAMTOR3, NAB2, PKNOX1, PRPS2, RNPC3, SERTAD4, SUDS3, TXNDC12, ZNF318, ZNF800, BACE2, BRF1, DYRK1A, LAMP2, MYO6, NPC1, PSMB1, SEC61A2, TWSG1, UBE2D1, UHRF1BP1L, CASP3, CYB5D1, GTF2A1, HDAC1, ICMT, KIAA1804, LPGAT1, NIPSNAP3A, RAP1GDS1, SETX, STRN, ZRANB3, CHCHD1, NTAN1, PPIC, PTGES, SLTM, TBC1D2, ACVR2A, C1ORF27, DPP8, E2F3, E2F7, ELL, KDM6A, KIF11, KIF1A, LPAR1, OSBPL1A, PHF16, PPIG, PTPN12, SESTD1, SPRED2, MTMR3, PEX13, SMC1A, USP4, CCNE1, CSTF3, DIABLO, EPN2-IT1, GPBP1, KDELC2, KLHL21, MAGEC2, NFYA, PJA2, RAB27A, RAP2B, SLC25A30, SPRED1, ANKRD5, DCAF16, HNRNPH3, ING2, POLR3E, PRKACA, C6ORF57, CDK12, DNAJC1, MFSD12, NDEL1, SRPK2, SRR, AMPD2, VPS54, ITPKC, KAT6A, NSD1, PANX1, RAB26, USP37, C16ORF87, IQSEC1, SEZ6L2, SLC23A2, SOCS5, IL10RB, RFT1, MTHFR, NRARP, OLFM2, ACAA2, HIVEP1, KBTBD7, CHST14, PTEN, ATP1B1, IL6ST |
| 8.87e-06 | TF:M00695_0 | tf | Factor: ETF; motif: GVGGMGG; match class: 0 | ARHGEF7, CCNE2, TMTC3, ARL4D, PTPRZ1, SDF4, CACNB4, GNA13, KBTBD2, PTRF, RUSC2, TXNDC11, ATF4, GPC3, RAB11FIP5, C3ORF37, CRTC2, DCAF11, FLII, FOXK2, IFI27L1, MDM2, NPTN, PIM2, SCD, UBE2J1, ARF6, CSPG5, KCNIP1, RFC4, SLC27A4, SLC35E1, USP36, ZBTB49, ATXN10, BICD2, KDELR2, MMS19, NUP210, R3HDM1, AACS, CEP112, FOXK1, NIPAL1, PLXND1, SAMHD1, SORBS3, TBC1D9B, TBX3, UBE2I, ABL1, BAIAP2L1, DNAJB1, DYNLL1, GNG12, GPC4, HSPA9, IKZF5, LPCAT4, MAN2A1, PPP1R26, SPSB1, SYT17, VAV3, ZDHHC8, COL4A1, ENOPH1, ERBB3, GLRX3, GTSE1, LIMCH1, LRP5, MED13, NAPEPLD, NFATC3, OSBPL2, PTCH1, SLC2A4RG, SLC7A1, TPM4, TRIM65, VEZF1, ZNHIT1, AEBP2, ATE1, CISD2, CLDND1, DNM3, GHITM, KCND2, LNX2, SLC30A1, STAU1, TAB2, TIPARP, TMEM57, WDR45L, ADCY1, ATP5C1, B4GALT6, BUB1, CAMTA1, CPT1A, LUC7L3, NUP153, PPFIA1, USP19, AAK1, AKIRIN2, CCNG2, HECW2, JARID2, KCMF1, MARC1, ODC1, PCDH1, PHAX, PLCG1, PPP2R5E, RABL6, RMI1, TIMM21, TMEM248, ALG12, ATAD2, C2ORF55, DCAF12, DERL1, GSK3B, HNRNPF, KIF1B, LGALSL, NDRG3, NUP155, PCBP4, PPAP2A, PRPF4, PXK, THUMPD3, TRH, DNER, ETNK2, NSUN2, PCIF1, PLXDC2, RASGEF1A, RCN2, RSBN1, ADNP2, ARAP2, ARHGAP5, ATXN7, BAP1, C18ORF8, C4ORF46, COG2, ERC1, EZH2, GAS6, KCNMB4, MOB1B, PKP4, RNF152, RNF214, ZNF621, ACSL3, APLP2, BAG5, DENND1A, FNBP1, HSPA14, IRF3, NCK2, NEDD1, PITPNA, PSMD1, PTPLAD1, STRA13, TDRD3, USP9X, YWHAH, ARMC9, CHDH, CYFIP2, FBXW8, FHL2, FOXJ3, LGR4, ME2, MKI67, MPP6, PARN, RANBP9, RBPJ, RSRC2, SCAI, SETDB1, TCP11L1, ZFC3H1, AMMECR1L, CENPK, CYLD, DIDO1, EXOSC10, IGF2R, NENF, RUFY2, ZMYM5, ATXN2, BCL2, C19ORF40, C6ORF130, CIB1, HERC1, MAN1A2, NR2C2, PEX3, POLE2, PPAT, REV3L, SERPINB1, ZDHHC18, AKAP11, BRIP1, C16ORF70, CPEB4, MEX3D, PDXDC1, TAF15, TES, UBTD2, USP38, AKAP13, ALG2, CAPRIN2, DOCK7, MBTPS1, RMND5A, ZBED4, AGFG1, AMIGO2, ATG14, FAT1, GPATCH2, PKNOX1, PLEKHA5, PPP2R3C, SERTAD4, TBC1D10B, TXNDC12, ZNF318, ZNF800, BACE2, BRF1, CENPBD1, DGUOK, DYRK1A, NPC1, PCNX, PLEKHA1, SLC1A1, TMEM68, TWSG1, UBE2D1, ZBTB25, KIAA1804, LPGAT1, NIPSNAP3A, RAP1GDS1, STRN, ZRANB3, CHCHD1, ESD, GUF1, NTAN1, PPIC, RTTN, SLTM, ACVR2A, C1ORF27, DPP8, E2F3, E2F7, ELL, KDM6A, KIF1A, LPAR1, MED28, PHF16, PRKD2, PTPN12, RPA1, RPRD1B, SESTD1, SPRED2, ZBTB39, ENC1, CCNE1, GPBP1, KDELC2, NFYA, PALB2, PASK, PJA2, RAP2B, SLC25A30, SPRED1, CEBPG, DCAF16, ING2, LRP12, POLR3E, PRKACA, SMEK1, DNAJC1, SRPK2, SRR, ZNF460, VPS54, ITPKC, KAT6A, NSD1, RAB26, TFB1M, USP37, C16ORF87, IQSEC1, RBM41, TTC14, ZNF543, ARNT2, HACE1, NRARP, OLFM2, ACAA2, HIVEP1, MIER3, TTBK2, USP1, DAPK2, ATP1B1, EXOC2, IL6ST, ZNF17, SPATA2 |
| 1.73e-03 | TF:M00327_4 | tf | Factor: Pax-3; motif: NNNNNNCGTCACGSTYNNNNN; match class: 4 | ARHGEF7, BCS1L, HECA, RBM18, PTRF, RPL7L1, CNOT1, HDX, NKX2-3, RAB11FIP5, TADA3, AXDND1, DCAF11, FLII, MDM2, PEX2, RNF141, SERPINF1, UBE2J1, ZNF219, CREB3L4, GPCPD1, SOAT1, USP36, ATL3, TMTC1, RPL37A, SEC13, TNK1, UBE2I, C11ORF58, CEP89, LRTOMT, SPTY2D1, WDR90, ANKRD27, CFP, EIF5B, ERBB3, RBM15, SLC25A19, TMEM106B, ZNRF2, CLDND1, SLC25A5, SLC30A1, TMEM57, AMD1, CAMTA1, ITSN2, LUC7L3, NUP153, WDSUB1, CCNG2, CHMP4B, DHX36, JARID2, ODC1, PCDH1, PHAX, PLCG1, C20ORF3, DDX20, HNRNPF, PPAP2A, TACC2, THUMPD3, DNER, ILF3, PLXDC2, RIN2, RSBN1, SLC25A33, TET2, ATXN7, C18ORF25, C1ORF122, ERC1, EZH2, GCC2, KCNMB4, MKRN2, PARP4, PHLPP1, RNF152, ZNF621, BAG5, BCL11B, DLD, IRF3, NCK2, PITPNA, SLC24A6, TDRD3, CYFIP2, EEA1, FHL2, FOXJ3, ME2, RBPJ, RRAGD, RSRC2, SETDB1, SMARCAL1, SPATA7, TSSC1, UFSP2, CRKL, CYLD, USP39, WDPCP, C19ORF40, CIB1, DACT2, DEGS1, PEX3, PPAT, SERPINB1, ZNF180, C16ORF70, CPEB4, FUT8, GTF3C4, LMTK2, REST, TNFRSF21, CDCP1, IKBKB, COQ9, FAT1, LAMTOR3, PLEKHA5, RNPC3, STARD3NL, BRF1, MYO6, PCNX, ZBTB25, CYB5D1, GTF2A1, STRN, CAMSAP1, CHCHD1, MTFP1, PPIC, PTGES, ARHGAP32, PPIG, PTPN12, RBM17, RPRD1B, EIF2AK2, GORAB, MTMR3, SMC1A, DIABLO, KDELC2, KLHL21, MAGEC2, ALMS1, DCAF16, FUCA1, LPCAT2, CDK12, AMPD2, USP37, C16ORF87, SOCS5, RFT1, ZNF543, ATAD5, HACE1, NRARP, KBTBD7, TTBK2, USP1, TUBGCP3, EMP2, RPGRIP1L |
| 2.79e-04 | TF:M00431_4 | tf | Factor: E2F-1; motif: TTTSGCGS; match class: 4 | ARHGEF7, TMTC3, BCS1L, SDF4, KBTBD2, RPL7L1, RUSC2, CNOT1, CYR61, MMGT1, RAB11FIP5, AIMP1, C3ORF37, CRTC2, FOXK2, MDM2, NIPAL3, PEX2, PIM2, SERPINF1, COMMD4, CREB3L4, DCTPP1, UGGT1, USP36, ZBTB49, ATL3, CDC42EP4, DDA1, NOLC1, NUP210, TRAPPC11, BYSL, CEP112, GFER, NIPAL1, R3HCC1L, TBX3, C11ORF58, C17ORF48, DYNLL1, GCA, GPC4, HSPA9, MANBAL, MON1B, POLA2, PPP1R26, SPSB1, TRMT6, TSEN15, ZDHHC8, ZNF227, COL4A1, IDH3A, LRP5, NFATC3, OSBPL2, PTCH1, RBM23, SDCCAG3, SLC7A1, ZNHIT1, ATE1, COX7C, GHITM, KCND2, LNX2, OSBPL10, SLC30A1, STAU1, TIMM9, TMEM57, WDR45L, ADCY1, ARGLU1, ATP5C1, B4GALT6, BUB3, CAMTA1, MOCS3, SETD7, USP19, WDSUB1, DONSON, HECW2, LPIN3, ODC1, RABL6, RMI1, STIM2, TIMM21, USP21, UTP11L, ZNF418, ARRDC4, ATAD2, DDX20, HNRNPF, KCNK5, MVP, NUP155, PPAP2A, PRPF38A, RGL1, DNER, ILF3, NSUN2, PCIF1, PELI3, PLXDC2, RSBN1, ARHGAP5, ATXN7, C18ORF25, C4ORF46, ERC1, KCNMB4, RNF152, SF3B3, INPP5D, IRF3, PSMD1, PTPLAD1, USP9X, VDR, CHDH, CYFIP2, EEA1, FBXW8, FHL2, FOXJ3, GMNN, OXR1, TTK, CYLD, HLCS, NENF, NFATC2, NOL12, RSPH1, RUFY2, WDPCP, C6ORF130, MAN1A2, PPAT, SLC22A5, C16ORF70, CNEP1R1, DIS3, EXPH5, FUT8, MEX3D, TES, CAPRIN2, IKBKB, MBTPS1, VBP1, AMIGO2, FAT1, NAB2, PLEKHA5, PNPLA4, PPP2R3C, SERTAD4, STARD3NL, TXNDC12, BRF1, CTDSPL2, PLEKHA1, SEC61A2, SLC1A1, TWSG1, UHRF1BP1L, GTF2A1, STRN, GUF1, MTFP1, RTTN, SLTM, CCNT1, E2F3, E2F7, LPAR1, OSBPL1A, PTPN12, RPA1, RPRD1B, TMEM30B, KDELC2, KLHL21, NFYA, RAB27A, SAT1, SPRED1, HNRNPH3, LEMD3, LRP12, PRKACA, SPATA5, SRR, VPS54, ZNF444, MND1, NSD1, USP37, C16ORF87, IQSEC1, SEZ6L2, TIA1, RPS15A, ZNF91, ATAD5, NRARP, ACAA2, HIVEP1, KBTBD7, TTBK2, USP1, PTEN, PYDC1, ATP1B1, BTBD1, IL6ST, UBAP1, RPGRIP1L |
| 9.38e-08 | TF:M00428_4 | tf | Factor: E2F-1; motif: NKTSSCGC; match class: 4 | ARHGEF7, CCNE2, POMC, TMTC3, ZG16B, ARL4D, BCS1L, HECA, PPP1R15B, PTPRZ1, SDF4, CACNB4, KBTBD2, RPL7L1, RUSC2, TXNDC11, ATF4, CCDC8, CNOT1, CYR61, EPN1, FGF5, GPC3, MMGT1, NKX2-3, NT5M, RAB11FIP5, TADA3, AIMP1, AXDND1, BCAM, C3ORF37, CRTC2, DCAF11, FLII, FOXK2, IFI27L1, IL17RA, MDM2, NCOA6, NIPAL3, NPTN, PEX2, PIM2, SCD, SERPINF1, UBE2J1, ALDH16A1, ARF6, B3GAT3, COMMD4, CREB3L4, CSPG5, CTDNEP1, DCTPP1, PSMD10, PSME3, RFC4, SLC27A4, SOAT1, SUPT7L, TMEM230, UGGT1, USP36, ZBTB49, AHCY, ASF1B, ATL3, BICD2, CDC42EP4, DDA1, MMS19, NOLC1, NUP210, PSENEN, R3HDM1, SBF1, TRADD, TRAPPC11, BYSL, CEP112, DRG1, FOXK1, GFER, NIPAL1, PLXND1, R3HCC1L, RTN2, SCML1, SEC13, SORBS3, TBC1D9B, TBX3, UBE2I, C11ORF58, C17ORF48, CEP89, DYNLL1, GCA, GPC4, HSPA9, IKZF5, KCNE1L, LRTOMT, MANBAL, MON1B, NOL11, OSBPL7, POLA2, PPP1R26, SPSB1, SPTY2D1, SYT17, TRMT6, TSEN15, UBXN2A, WDR90, ZDHHC8, ZNF227, ANKRD27, APBB2, ARMC2, C9ORF116, CCDC18, COL4A1, EIF5B, ENOPH1, ERBB3, GLRX3, GRSF1, GTSE1, IDH3A, IRF1, LAP3, LIMCH1, LRP5, MED13, MOB1A, NAPEPLD, NCAPG2, NFATC3, OSBPL2, PTCH1, RBM15, RBM23, RSL24D1, SDCCAG3, SLC25A19, SLC2A4RG, SLC7A1, TMEM106B, TRIM65, WDR17, VEZF1, ZNHIT1, AEBP2, ATE1, BRPF1, CISD2, COX7C, DRAM2, FN3KRP, GHITM, KCND2, LNX2, NMB, OSBPL10, PAF1, RASEF, SLC30A1, STAU1, TAB2, TIMM9, TMEM57, TSR2, WDR45L, ADCY1, AMD1, ARGLU1, ATP5C1, B4GALT6, BUB3, CAMTA1, CARKD, CPT1A, KRTAP10-11, LUC7L3, MED31, MOCS3, PDCL, PPFIA1, SETD7, USP19, WDSUB1, VPS33B, AAGAB, AAK1, AKIRIN2, CHMP4B, DAG1, DONSON, HECW2, LACTB2, LPIN3, MARC1, MBNL1, NME3, NOA1, ODC1, PCDH1, RABL6, RMI1, STIM2, TIMM21, TMEM248, USP21, UTP11L, ZNF418, AKAP9, ALG12, ARRDC4, ATAD2, DDX20, HNRNPF, KCNK5, KIF1B, KRT17, LGALSL, MVP, NUP155, PCBP4, PPAP2A, PRPF38A, RGL1, TMEM170A, TRH, DNER, ETNK2, ILF3, KIF23, NSUN2, PCGF3, PCIF1, PELI3, PLXDC2, RASGEF1A, RCN2, RSBN1, SH3BP4, TET2, ARAP2, ARHGAP5, ARL6, ATXN7, BAP1, C18ORF25, C18ORF8, C4ORF46, COG2, DHX15, ERC1, EZH2, KCNMB4, KLK7, MKRN2, MLH3, PHLPP1, PKP4, RNF152, RNF214, SF3B3, SSH3, ZNF584, ZNF621, ACSL3, BAG5, FNBP1, HTRA2, INPP5D, IRF3, NCK2, NEDD1, NIP7, PSMD1, PTPLAD1, STRA13, TDRD3, USP9X, VDR, YWHAH, ZDHHC4, CHDH, CYFIP2, EEA1, FBXW8, FHL2, FOXJ3, GMNN, HSCB, MKI67, MPP6, NFRKB, OXR1, RANBP9, RBPJ, RRAGD, SCAI, SETDB1, SPATA7, SRD5A3, TCP11L1, TM2D1, TTK, UFSP2, AMMECR1L, CENPK, CRKL, CYLD, DHX35, DIDO1, FASTKD2, FRRS1, HLCS, NENF, NFATC2, NOL12, NUDCD1, PSMD12, RSPH1, RUFY2, SMAP2, USP39, WDPCP, ZMYM5, ZNF267, BCL2, C19ORF40, C6ORF130, DACT2, DEGS1, HERC1, LRP1, MAN1A2, NR2C2, PEX3, POLE2, PPAT, SLC22A5, ZNF180, ZNF627, ZNF75A, BRIP1, C16ORF70, CBR4, CNEP1R1, DIS3, EXPH5, FUT8, GTF3C4, MEX3D, MGRN1, PDXDC1, TES, UBTD2, USP38, ZNF510, AKAP13, CAPRIN2, DOCK7, ELF2, HIATL1, IKBKB, MBTPS1, MFAP3L, NAA40, NUP160, SCNN1B, SEC63, SREBF1, VBP1, VPS4B, AMIGO2, CCNL1, FAT1, ICT1, NAB2, PKNOX1, PLEKHA5, PNPLA4, PPP2R3C, RNPC3, SERTAD4, STARD3NL, SUDS3, TBC1D10B, TXNDC12, ZNF318, ZNF800, BACE2, BRF1, CENPBD1, CTDSPL2, DYRK1A, GLE1, MYO6, NPC1, PLEKHA1, PVRL4, RRAGA, SEC61A2, SLC1A1, TMEM68, TWSG1, UHRF1BP1L, CASP3, CYB5D1, FLVCR1, GTF2A1, ICMT, KIAA1804, PRKCI, SETX, STRN, ZRANB3, GUF1, MB21D2, MTFP1, RNF219, RTTN, SLTM, TBC1D2, ACVR2A, C1ORF27, CCNT1, DPP8, E2F3, E2F7, ELL, KDM6A, KIF11, KIF1A, LPAR1, MED28, OSBPL1A, PHF16, PTPN12, RBM17, RPA1, RPRD1B, SESTD1, SPRED2, TMEM30B, ZBTB39, ENC1, MTMR3, USP4, CCNE1, CSTF3, EPN2-IT1, GPBP1, KDELC2, KLHL21, NFYA, RAB27A, RAP2B, SAT1, SLC25A30, SPRED1, ALMS1, ANKRD5, CEBPG, DCAF16, FUCA1, HNRNPH3, ING2, LEMD3, LRP12, PRKACA, SMEK1, CDK12, DNAJC1, MFSD12, NDEL1, PRRG4, SPATA5, SRPK2, SRR, AMPD2, VPS54, ZNF444, ITPKC, KAT6A, MND1, NSD1, PANX1, RAB26, USP37, C16ORF87, IQSEC1, SEZ6L2, SLC23A2, SOCS5, TIA1, RPS15A, PCDHB5, ZNF91, ARNT2, ATAD5, NDUFAF1, HACE1, NRARP, OLFM2, ACAA2, HIVEP1, KBTBD7, TTBK2, USP1, DAPK2, PTEN, PYDC1, ATP1B1, BTBD1, EXOC2, IL6ST, NAA16, UBAP1, PRR15, CEP152, EMP2, RPGRIP1L |
| 7.44e-05 | TF:M00196_4 | tf | Factor: Sp1; motif: NGGGGGCGGGGYN; match class: 4 | CCNE2, OGDHL, TMTC3, BCS1L, C5ORF34, HECA, PPP1R15B, PTPRZ1, RBM18, GNA13, KBTBD2, LRIG1, PTRF, TXNDC11, XKR6, ATF4, CYR61, EPN1, FGF5, GPC3, MMGT1, NKX2-3, NT5M, RAB11FIP5, RNF111, TADA3, BCAM, C3ORF37, DCAF11, FLII, FMNL2, FOXK2, IFI27L1, NCOA6, NPTN, OAT, PIM2, RNF141, SCD, UBE2J1, ALDH16A1, ARF6, B3GAT3, COMMD4, CSPG5, CTDNEP1, GPCPD1, KCNIP1, RFC4, SLC35E1, SOAT1, UGGT1, USP36, AHCY, ATL3, BICD2, CAMTA2, DDA1, KDELR2, MMS19, NUP210, PSENEN, R3HDM1, SBF1, TRADD, AACS, BYSL, CEP112, DRG1, FOXK1, GFER, NIPAL1, PLXND1, R3HCC1L, RSF1, RTN2, SCML1, SEC13, SORBS3, TBC1D9B, TBX3, TNK1, UBE2I, WDR83, ABL1, BAIAP2L1, DNAJB1, DYNLL1, GCA, GNG12, IKZF5, KCNE1L, LPCAT4, LRTOMT, MAN2A1, OSBPL7, PPP1R26, PTGER4, SPSB1, TRMT6, UBXN2A, VAV3, ZDHHC8, ANKRD27, C14ORF126, ENOPH1, ERBB3, GLRX3, GRSF1, IDH3A, IRF1, LAP3, LIMCH1, LRP5, NCAPG2, NFATC3, OSBPL2, PAFAH1B2, PTCH1, RBM15, SDCCAG3, SLC2A4RG, SLC7A1, TPM4, TRIM65, WDR17, VEZF1, WFS1, YDJC, ZNHIT1, AEBP2, ATE1, BRPF1, CISD2, CLDND1, DNM3, FN3KRP, GHITM, KCND2, LNX2, NARG2, PAF1, SLC25A5, SLC30A1, STAU1, TIPARP, TMEM57, WDR45L, ADCY1, ATP5C1, B4GALT6, CAMTA1, CPT1A, ITSN2, LUC7L3, MED31, PPFIA1, SETD7, USP19, WDSUB1, AAGAB, AKIRIN2, AR, CCNG2, DAG1, DONSON, KCMF1, KREMEN1, LACTB2, LPIN3, NME3, ODC1, PCDH1, PLCG1, RABL6, RMI1, STIM2, ALG12, ARRDC4, C20ORF3, C2ORF55, COX7A2L, DERL1, HNRNPF, KCNK5, KIF1B, LGALSL, NDRG3, OSBP2, PCBP4, PPAP2A, PRPF38A, RGL1, SERAC1, TRH, UAP1, ANAPC4, DNER, ETNK2, ILF3, NSUN2, PCGF3, PELI3, PLXDC2, RCN2, RSBN1, SH3BP4, SLC25A33, ADNP2, ARAP2, ARHGAP5, BAP1, C18ORF8, ERC1, EZH2, GAS6, GCC2, KCNMB4, KLK7, MUM1L1, PARP4, PHLPP1, RNF152, SSH3, ZNF584, ZNF621, ACSL3, APLP2, BAG5, BCL11B, DENND1A, FNBP1, HTRA2, NCK2, NEDD1, PITPNA, PSMD1, PTPLAD1, SLC24A6, STRA13, TWF2, USP9X, VDR, YWHAH, AP1B1, ARMC9, CYFIP2, EEA1, FHL2, FOXJ3, GMNN, HSCB, ME2, MKI67, MPP6, OXR1, PARN, RANBP9, RBPJ, RRAGD, RSRC2, SRD5A3, UFSP2, ZFC3H1, AMMECR1L, CYLD, DHX35, DIDO1, EXOSC10, IGF2R, NENF, NFATC2, NUDCD1, PSMD12, RAB9A, RSPH1, RUFY2, SMAP2, SPINT2, WDPCP, ZNF267, ATXN2, BCL2, CIB1, DACT2, DEGS1, HERC1, JAGN1, LRP1, MAN1A2, MRPS10, PPAT, PPP1R16A, REV3L, SERPINB1, SLC22A5, ZDHHC18, ZNF180, ZNF627, AKAP11, EXPH5, FUT8, GTF3C4, LMTK2, MEX3D, PDXDC1, REST, TNFRSF21, UBQLN4, UBTD2, AKAP13, ALG2, CAPRIN2, FIG4, IKBKB, NAA40, NUP160, RNASEH2B, SCNN1B, SEC63, SREBF1, ZBED4, ANKRD49, FAT1, FOPNL, LAMTOR3, NAB2, PKNOX1, PRPS2, RNPC3, SERTAD4, SUDS3, TBC1D10B, TXNDC12, ZNF800, BACE2, BRF1, DYRK1A, LAMP2, MYO6, NPC1, PSMB1, SEC61A2, TMEM68, TWSG1, UBE2D1, UHRF1BP1L, CASP3, CYB5D1, FLVCR1, GTF2A1, HDAC1, ICMT, KIAA1804, LPGAT1, NIPSNAP3A, RAP1GDS1, SETX, STRN, ZRANB3, NTAN1, PTGES, TBC1D2, ACVR2A, C1ORF27, DPP8, E2F3, E2F7, ELL, KDM6A, KIF11, KIF1A, LPAR1, OSBPL1A, PHF16, PTPN12, RPA1, SESTD1, MTMR3, PEX13, SMC1A, USP4, CCNE1, CSTF3, DIABLO, EPN2-IT1, GPBP1, KDELC2, KLHL21, MAGEC2, NFYA, PJA2, RAB27A, RAP2B, SLC25A30, SPRED1, ANKRD5, HNRNPH3, ING2, POLR3E, PRKACA, C6ORF57, DNAJC1, MFSD12, NDEL1, SRPK2, SRR, AMPD2, VPS54, ITPKC, KAT6A, NSD1, PANX1, RAB26, USP37, C16ORF87, IQSEC1, SLC23A2, SOCS5, IL10RB, RFT1, NRARP, OLFM2, ACAA2, HIVEP1, CHST14, DAPK2, PTEN, ATP1B1, IL6ST |
| 3.35e-03 | TF:M00933_3 | tf | Factor: Sp1; motif: CCCCGCCCCN; match class: 3 | OGDHL, BCS1L, PPP1R15B, PTPRZ1, RBM18, KBTBD2, LRIG1, PTRF, TXNDC11, XKR6, ATF4, CCDC8, CYR61, FGF5, GPC3, MMGT1, NKX2-3, NT5M, RAB11FIP5, TADA3, BCAM, C3ORF37, DCAF11, FLII, FMNL2, NCOA6, NDUFS2, NPTN, OAT, PIM2, RNF141, SCD, UBE2J1, ALDH16A1, ARF6, B3GAT3, CSPG5, CTDNEP1, GPCPD1, KCNIP1, SLC35E1, SOAT1, USP36, AHCY, ATL3, ATXN10, CAMTA2, DDA1, KDELR2, MMS19, NUP210, PSENEN, SBF1, AACS, BYSL, CEP112, DRG1, FOXK1, GFER, NIPAL1, PLXND1, RSF1, RTN2, SCML1, SORBS3, TBC1D9B, TBX3, UBE2I, WDR83, ABL1, BAIAP2L1, DNAJB1, DYNLL1, GNG12, GPC4, IKZF5, KCNE1L, LPCAT4, LRTOMT, MAN2A1, OSBPL7, PPP1R26, PTGER4, SPSB1, SYT17, TRMT6, VAV3, ZDHHC8, ANKRD27, C9ORF116, COL4A1, ENOPH1, ERBB3, GLRX3, IDH3A, IRF1, LIMCH1, NCAPG2, NFATC3, OSBPL2, PTCH1, RBM15, SDCCAG3, SLC2A4RG, SLC7A1, TMEM106B, TPM4, TRIM65, WDR17, VEZF1, WFS1, YDJC, ZNHIT1, AEBP2, ATE1, BRPF1, CLDND1, DNM3, FN3KRP, GHITM, KCND2, LNX2, NARG2, SLC25A5, SLC30A1, STAU1, TAB2, TMEM57, WDR45L, ADCY1, ATP5C1, B4GALT6, CAMTA1, CPT1A, F7, ITSN2, LUC7L3, MED31, PPFIA1, USP19, WDSUB1, AAGAB, AAK1, CCNG2, DAG1, JARID2, KCMF1, KREMEN1, LACTB2, LPIN3, NME3, ODC1, PCDH1, PLCG1, RMI1, STIM2, AKAP9, ALG12, ARRDC4, C20ORF3, C2ORF55, COX7A2L, GSK3B, HNRNPF, KCNK5, KIF1B, LGALSL, NDRG3, OSBP2, PCBP4, PPAP2A, PRPF38A, RGL1, SERAC1, TRH, UAP1, ANAPC4, DNER, ETNK2, ILF3, NSUN2, PCGF3, PELI3, PLXDC2, RSBN1, SH3BP4, SLC25A33, ADNP2, ARAP2, ARHGAP5, BAP1, C18ORF8, ERC1, EZH2, KCNMB4, KLK7, MUM1L1, PARP4, RNF152, RNF214, SSH3, ZNF584, ACSL3, APLP2, BAG5, BCL11B, DENND1A, FNBP1, INPP5D, NCK2, PITPNA, PTPLAD1, SLC24A6, STRA13, TWF2, USP9X, VDR, YWHAH, AP1B1, CYFIP2, EEA1, FHL2, FOXJ3, GMNN, MKI67, MPP6, OXR1, PARN, RRAGD, SETDB1, SMARCAL1, SRD5A3, UFSP2, ZFC3H1, AMMECR1L, CYLD, DHX35, DIDO1, IGF2R, NFATC2, NUDCD1, RAB9A, RSPH1, RUFY2, SMAP2, SPINT2, WDPCP, ZNF267, DEGS1, JAGN1, LRP1, MAN1A2, PPAT, PPP1R16A, ZNF180, ZNF627, EXPH5, FUT8, GTF3C4, MEX3D, PDXDC1, REST, TNFRSF21, UBQLN4, UBTD2, AKAP13, ALG2, CAPRIN2, FIG4, IKBKB, NUP160, RMND5A, RNASEH2B, SCNN1B, SREBF1, ZBED4, ANKRD49, FOPNL, LAMTOR3, NAB2, PKNOX1, PRPS2, RNPC3, SUDS3, TXNDC12, ZNF800, BACE2, BRF1, LAMP2, MYO6, NPC1, PSMB1, SEC61A2, TWSG1, UBE2D1, UHRF1BP1L, CASP3, CYB5D1, GTF2A1, HDAC1, ICMT, KIAA1804, LPGAT1, NIPSNAP3A, RAP1GDS1, SETX, STRN, ZRANB3, NTAN1, PTGES, SLTM, TBC1D2, ACVR2A, C1ORF27, DPP8, E2F3, E2F7, ELL, KDM6A, KIF11, KIF1A, LPAR1, OSBPL1A, PHF16, SESTD1, MTMR3, PEX13, SMC1A, USP4, CCNE1, CSTF3, DIABLO, EPN2-IT1, GPBP1, MAGEC2, NFYA, PJA2, RAB27A, RAP2B, SLC25A30, SPRED1, ANKRD5, DCAF16, ING2, LRP12, POLR3E, PRKACA, CDK12, NDEL1, SRPK2, SRR, AMPD2, VPS54, ITPKC, KAT6A, NSD1, PANX1, RAB26, USP37, C16ORF87, IQSEC1, SLC23A2, SOCS5, IL10RB, RFT1, NRARP, OLFM2, ACAA2, HIVEP1, CHST14, PTEN, ATP1B1, IL6ST |

**Table C. Genes upregulated in response to S6K2 siRNA, but not to S6K1 siRNA.**

| Transcripts Cluster ID | Gene description | Gene symbol | S6K2 siRNA  Fold change | S6K2 siRNA  p-value^1^ | S6K1 siRNA  Fold change | S6K1 siRNA  p-value^1^ |
| --- | --- | --- | --- | --- | --- | --- |
| 16740994 | ribosomal protein S6 kinase, 70kDa, polypeptide 2 | RPS6KB2 | -1,5 | 0,0387 | -0,47 | 0,2927 |
| 16727861 | ribosomal protein S6 kinase, 70kDa, polypeptide 2 | RPS6KB2 | -1,36 | 0,0349 | 0,08 | 0,5838 |
| 16797423 | immunoglobulin heavy diversity 2-15 | IGHD2-15 | -0,61 | 0,0309 | -0,17 | 0,6235 |
| 16670367 | Fc fragment of IgG, high affinity Ia, receptor (CD64) \| Fc fragment of IgG, high affinity Ib, receptor (CD64) \| Fc fragment of IgG, high affinity Ic, receptor (CD64), pseudogene | FCGR1A\| FCGR1B\| FCGR1C | -0,5 | 0,0490 | -0,18 | 0,4563 |
| 17111545 | MT-RNR2-like 10 | MTRNR2L10 | -0,49 | 0,0387 | -0,32 | 0,4820 |
| 16948648 | 5-hydroxytryptamine (serotonin) receptor 3C, ionotropic | HTR3C | -0,47 | 0,0475 | -0,39 | 0,0659 |
| 16927844 | immunoglobulin lambda-like polypeptide 5 \| immunoglobulin lambda constant 1 (Mcg marker) \| immunoglobulin lambda joining 1 | IGLL5\| IGLC1\| IGLJ1 | -0,47 | 0,0472 | -0,44 | 0,1096 |
| 16804141 | elongation factor Tu GTP-binding domain-containing protein 1 pseudogene | LOC648809 | -0,44 | 0,0480 | -0,26 | 0,4570 |
| 16889160 | phospholipase C-like 1 | PLCL1 | -0,42 | 0,0493 | -0,23 | 0,1058 |
| 16734829 | olfactory receptor, family 52, subfamily A, member 5 | OR52A5 | -0,39 | 0,0420 | -0,09 | 0,5778 |
| 16724679 | olfactory receptor, family 4, subfamily A, member 47 | OR4A47 | -0,38 | 0,0303 | -0,22 | 0,0751 |
| 16764127 | dendrin | DDN | -0,37 | 0,0335 | -0,33 | 0,0503 |
| 17002898 | stanniocalcin 2 | STC2 | -0,37 | 0,0494 | -0,25 | 0,0574 |
| 16797415 | immunoglobulin heavy constant alpha 1 \| immunoglobulin heavy constant mu \| immunoglobulin heavy constant alpha 2 (A2m marker) \| immunoglobulin heavy locus \| immunoglobulin heavy joining 3 | IGHA1\| IGHM\| IGHA2\| IGH@\| IGHJ3 | -0,36 | 0,0453 | -0,12 | 0,3192 |
| 16924155 | transmembrane phosphatase with tensin homology | TPTE | -0,36 | 0,0076 | 0,20 | 0,2543 |
| 17052635 | T cell receptor beta variable 27 | TRBV27 | -0,36 | 0,0054 | -0,25 | 0,1020 |
| 17109146 | ataxin 3-like | ATXN3L | -0,35 | 0,0135 | -0,30 | 0,1018 |
| 16797467 | immunoglobulin heavy constant delta \| immunoglobulin heavy variable 3-9 | IGHD\| IGHV3-9 | -0,35 | 0,0091 | -0,22 | 0,2022 |
| 16796752 | retrotransposon-like 1 | RTL1 | -0,35 | 0,0338 | -0,43 | 0,1160 |
| 17012087 | solute carrier family 35, member F1 | SLC35F1 | -0,35 | 0,0446 | -0,26 | 0,0905 |
| 16766655 | osteosarcoma amplified 9, endoplasmic reticulum lectin | OS9 | -0,34 | 0,0289 | -0,24 | 0,3257 |
| 16712086 | integrin, alpha 8 | ITGA8 | -0,33 | 0,0204 | -0,23 | 0,0560 |
| 16837391 | potassium inwardly-rectifying channel, subfamily J, member 2 | KCNJ2 | -0,33 | 0,0257 | -0,12 | 0,0619 |
| 17110235 | A20-binding inhibitor of NF-kappaB activation 2 pseudogene | LOC100132831 | -0,33 | 0,0371 | -0,13 | 0,5521 |
| 16920610 | MT-RNR2-like 3 | MTRNR2L3 | -0,33 | 0,0480 | -0,20 | 0,4715 |
| 16924113 | transmembrane phosphatase with tensin homology | TPTE | -0,33 | 0,0281 | 0,03 | 0,8687 |
| 16929738 | C1q and tumor necrosis factor related protein 6 | C1QTNF6 | -0,32 | 0,0199 | -0,31 | 0,0694 |
| 16949673 | FGF12 antisense RNA 2 (non-protein coding) | FGF12-AS2 | -0,32 | 0,0234 | -0,36 | 0,1049 |
| 16924828 | keratin associated protein 19-2 | KRTAP19-2 | -0,32 | 0,0412 | -0,12 | 0,5295 |
| 16738395 | olfactory receptor, family 5, subfamily AP, member 2 | OR5AP2 | -0,32 | 0,0469 | -0,13 | 0,5007 |
| 16987902 | thymic stromal lymphopoietin | TSLP | -0,32 | 0,0087 | -0,28 | 0,1092 |
| 16806249 | oculocutaneous albinism II | OCA2 | -0,31 | 0,0336 | -0,23 | 0,0826 |
| 16910959 | oxytocin, prepropeptide | OXT | -0,31 | 0,0070 | -0,12 | 0,1583 |
| 16914084 | WNT1 inducible signaling pathway protein 2 | WISP2 | -0,31 | 0,0256 | -0,29 | 0,1809 |
| 16709245 | adrenoceptor alpha 2A | ADRA2A | -0,3 | 0,0066 | -0,12 | 0,0927 |
| 16938007 | FYVE, RhoGEF and PH domain containing 5 | FGD5 | -0,3 | 0,0285 | 0,09 | 0,6255 |
| 16912041 | somatostatin receptor 4 | SSTR4 | -0,3 | 0,0241 | -0,49 | 0,0753 |
| 16828228 | chromosome 16 open reading frame 47 | C16orf47 | -0,29 | 0,0083 | -0,16 | 0,1402 |
| 16852702 | cadherin 20, type 2 | CDH20 | -0,29 | 0,0487 | -0,29 | 0,1723 |
| 17021510 | cannabinoid receptor 1 (brain) | CNR1 | -0,29 | 0,0398 | -0,12 | 0,3356 |
| 17005740 | HLA complex group 11 (non-protein coding) | HCG11 | -0,29 | 0,0402 | -0,37 | 0,1629 |
| 16910536 | programmed cell death 1 | PDCD1 | -0,29 | 0,0110 | -0,17 | 0,3208 |
| 16758931 | transmembrane protein 132B | TMEM132B | -0,29 | 0,0001 | -0,19 | 0,3109 |
| 16760792 |  | CD163 | -0,28 | 0,0030 | -0,20 | 0,0663 |
| 16777675 | caudal type homeobox 2 | CDX2 | -0,28 | 0,0318 | -0,08 | 0,6984 |
| 16826444 | sorting nexin 20 | SNX20 | -0,28 | 0,0131 | -0,02 | 0,8273 |
| 16882930 | tripartite motif containing 43B \| tripartite motif containing 43 | TRIM43B | -0,28 | 0,0226 | -0,09 | 0,1377 |
| 16835112 | wingless-type MMTV integration site family, member 9B | WNT9B | -0,28 | 0,0122 | -0,16 | 0,1122 |
| 16983263 | ANKRD33B intronic transcript 1 (non-protein coding) | ANKRD33B-IT1 | -0,27 | 0,0258 | -0,09 | 0,3483 |
| 16881199 | ATPase, H+ transporting, lysosomal 56/58kDa, V1 subunit B1 | ATP6V1B1 | -0,27 | 0,0330 | -0,14 | 0,2181 |
| 17107505 | chromosome X open reading frame 1 | CXorf1 | -0,27 | 0,0051 | -0,18 | 0,2727 |
| 16859314 | Kruppel-like factor 2 (lung) | KLF2 | -0,27 | 0,0027 | -0,26 | 0,1106 |
| 16748095 | killer cell lectin-like receptor subfamily G, member 1 | KLRG1 | -0,27 | 0,0419 | -0,29 | 0,1020 |
| 16725656 | leucine rich repeat containing 10B | LRRC10B | -0,27 | 0,0147 | -0,25 | 0,0503 |
| 16918210 | myeloid/lymphoid or mixed-lineage leukemia (trithorax homolog, Drosophila); translocated to, 10 pseudogene 1 | MLLT10P1 | -0,27 | 0,0138 | -0,25 | 0,1662 |
| 16778155 | SMAD family member 9 | SMAD9 | -0,27 | 0,0199 | -0,22 | 0,0597 |
| 17012761 | transcription factor 21 | TCF21 | -0,27 | 0,0301 | -0,15 | 0,4397 |
| 16877904 | coiled-coil domain containing 164 | CCDC164 | -0,26 | 0,0287 | -0,08 | 0,3359 |
| 17091212 |  | ESP33 | -0,26 | 0,0255 | -0,16 | 0,3098 |
| 16670131 | gap junction protein, alpha 5, 40kDa | GJA5 | -0,26 | 0,0001 | -0,07 | 0,4437 |
| 16731422 | 5-hydroxytryptamine (serotonin) receptor 3A, ionotropic | HTR3A | -0,26 | 0,0447 | -0,29 | 0,1770 |
| 17040702 | lymphotoxin alpha (TNF superfamily, member 1) | LTA | -0,26 | 0,0172 | -0,21 | 0,0627 |
| 16922212 | oligodendrocyte transcription factor 1 | OLIG1 | -0,26 | 0,0070 | -0,19 | 0,1766 |
| 16752181 | olfactory receptor, family 2, subfamily AP, member 1 | OR2AP1 | -0,26 | 0,0118 | -0,21 | 0,3197 |
| 16714237 | poly (ADP-ribose) glycohydrolase \| family with sequence similarity 25, member A | PARG\| FAM25A | -0,26 | 0,0279 | -0,06 | 0,5883 |
| 16722825 | solute carrier family 6 (neurotransmitter transporter, glycine), member 5 | SLC6A5 | -0,26 | 0,0140 | -0,23 | 0,1107 |
| 16724849 | smoothelin-like 1 | SMTNL1 | -0,26 | 0,0075 | -0,17 | 0,1509 |
| 16704347 | chromosome 10 open reading frame 10 | C10orf10 | -0,25 | 0,0424 | -0,18 | 0,3363 |
| 17029685 | chromosome 6 open reading frame 10 | C6orf10 | -0,25 | 0,0103 | -0,15 | 0,1911 |
| 16895590 | calcium and integrin binding family member 4 | CIB4 | -0,25 | 0,0007 | -0,17 | 0,3477 |
| 16800921 | fibroblast growth factor 7 | FGF7 | -0,25 | 0,0065 | -0,14 | 0,0929 |
| 16933131 | glutathione S-transferase theta pseudogene 2 | GSTTP2 | -0,25 | 0,0197 | -0,10 | 0,2550 |
| 16945434 | H1 histone family, member O, oocyte-specific | H1FOO | -0,25 | 0,0169 | -0,01 | 0,9053 |
| 17094233 | keratinocyte growth factor-like protein 2 | KGFLP2 | -0,25 | 0,0291 | -0,17 | 0,2968 |
| 16965427 | SLIT2 intronic transcript 1 (non-protein coding) | SLIT2-IT1 | -0,25 | 0,0284 | -0,09 | 0,5022 |
| 16972198 | tolloid-like 1 | TLL1 | -0,25 | 0,0334 | 0,00 | 0,9633 |
| 17114575 | G protein-coupled receptor 101 | GPR101 | -0,24 | 0,0334 | -0,20 | 0,2800 |
| 17042548 | major histocompatibility complex, class II, DP alpha 1 | HLA-DPA1 | -0,24 | 0,0058 | -0,22 | 0,1787 |
| 16937671 | histamine receptor H1 | HRH1 | -0,24 | 0,0455 | -0,12 | 0,1238 |
| 16882759 | immunoglobulin kappa variable 2D-24 (non-functional) \| immunoglobulin kappa variable 3D-20 | IGKV2D-24\| IGKV3D-20 | -0,24 | 0,0024 | -0,26 | 0,2622 |
| 16949348 | kininogen 1 | KNG1 | -0,24 | 0,0083 | -0,15 | 0,0628 |
| 16752172 | olfactory receptor, family 6, subfamily C, member 76 | OR6C76 | -0,24 | 0,0228 | 0,02 | 0,6712 |
| 16844061 | plexin domain containing 1 | PLXDC1 | -0,24 | 0,0289 | -0,13 | 0,3769 |
| 17109498 | PPEF1 antisense RNA 1 (non-protein coding) | PPEF1-AS1 | -0,24 | 0,0241 | -0,05 | 0,6571 |
| 17106601 | ribosomal protein L39 \| uncharacterized LOC100652821 | RPL39\| LOC100652821 | -0,24 | 0,0159 | -0,16 | 0,2261 |
| 16962523 | ribosomal protein L39-like | RPL39L | -0,24 | 0,0207 | 0,00 | 0,9971 |
| 16679298 | ribosomal protein S7 pseudogene 5 | RPS7P5 | -0,24 | 0,0467 | -0,02 | 0,6529 |
| 17023810 | serum/glucocorticoid regulated kinase 1 | SGK1 | -0,24 | 0,0467 | -0,05 | 0,6670 |
| 16874911 | sialic acid binding Ig-like lectin 5 | SIGLEC5 | -0,24 | 0,0027 | -0,21 | 0,0535 |
| 16755826 | Spi-C transcription factor (Spi-1/PU.1 related) | SPIC | -0,24 | 0,0273 | -0,22 | 0,1950 |
| 17062368 | taste receptor, type 2, member 16 | TAS2R16 | -0,24 | 0,0407 | -0,23 | 0,2851 |
| 16744939 | TMPRSS4 antisense RNA 1 (non-protein coding) | TMPRSS4-AS1 | -0,24 | 0,0433 | -0,15 | 0,2192 |
| 16926778 | transmembrane phosphatase with tensin homology pseudogene 1 | TPTEP1 | -0,24 | 0,0449 | -0,49 | 0,1824 |
| 17018601 | chromosome 6 open reading frame 222 | C6orf222 | -0,23 | 0,0416 | -0,03 | 0,8214 |
| 16833231 | chemokine (C-C motif) ligand 13 | CCL13 | -0,23 | 0,0135 | -0,26 | 0,0717 |
| 17107329 | CD40 ligand | CD40LG | -0,23 | 0,0393 | -0,09 | 0,3209 |
| 17051601 | carboxypeptidase A1 (pancreatic) | CPA1 | -0,23 | 0,0287 | -0,14 | 0,1663 |
| 16769882 | forkhead box N4 | FOXN4 | -0,23 | 0,0460 | -0,10 | 0,0662 |
| 16729858 | GRM5 antisense RNA 1 (non-protein coding) | GRM5-AS1 | -0,23 | 0,0357 | -0,11 | 0,1759 |
| 17037331 | major histocompatibility complex, class II, DP alpha 1 | HLA-DPA1 | -0,23 | 0,0197 | -0,18 | 0,2214 |
| 17034851 | major histocompatibility complex, class II, DP alpha 1 | HLA-DPA1 | -0,23 | 0,0259 | -0,19 | 0,2343 |
| 16776160 | integrin, beta-like 1 (with EGF-like repeat domains) | ITGBL1 | -0,23 | 0,0143 | -0,30 | 0,1696 |
| 16944269 | LSAMP antisense RNA 3 (non-protein coding) | LSAMP-AS3 | -0,23 | 0,0420 | -0,12 | 0,1232 |
| 16966809 | platelet-derived growth factor receptor, alpha polypeptide \| FIP1 like 1 (S. cerevisiae) | PDGFRA\| FIP1L1 | -0,23 | 0,0153 | -0,13 | 0,4148 |
| 17078751 | REX1, RNA exonuclease 1 homolog (S. cerevisiae)-like 1 \| exonuclease GOR-like | REXO1L1\| LOC100288562 | -0,23 | 0,0262 | -0,22 | 0,4470 |
| 16762053 | solute carrier organic anion transporter family, member 1A2 | SLCO1A2 | -0,23 | 0,0218 | -0,07 | 0,3240 |
| 16875192 | vomeronasal 1 receptor 4 | VN1R4 | -0,23 | 0,0102 | 0,08 | 0,5296 |
| 16988874 | ADAM metallopeptidase with thrombospondin type 1 motif, 19 | ADAMTS19 | -0,22 | 0,0016 | -0,02 | 0,2866 |
| 16801063 | DDB1 and CUL4 associated factor 13 pseudogene 3 | DCAF13P3 | -0,22 | 0,0156 | 0,05 | 0,7960 |
| 16854509 | desmocollin 1 | DSC1 | -0,22 | 0,0246 | -0,24 | 0,1513 |
| 16872767 | platelet-activating factor acetylhydrolase 1b, catalytic subunit 3 (29kDa) | PAFAH1B3 | -0,22 | 0,0057 | -0,14 | 0,1875 |
| 16908928 | paired box 3 | PAX3 | -0,22 | 0,0477 | -0,32 | 0,1520 |
| 16741725 | phosphodiesterase 2A, cGMP-stimulated | PDE2A | -0,22 | 0,0102 | -0,06 | 0,7236 |
| 16959745 | proline rich 23A | PRR23A | -0,22 | 0,0443 | -0,29 | 0,4031 |
| 16952349 | sodium channel, voltage-gated, type XI, alpha subunit | SCN11A | -0,22 | 0,0078 | 0,01 | 0,9714 |
| 17061624 | SLC26A4 antisense RNA 1 (non-protein coding) | SLC26A4-AS1 | -0,22 | 0,0100 | -0,26 | 0,1273 |
| 17018562 | solute carrier family 26, member 8 | SLC26A8 | -0,22 | 0,0499 | -0,12 | 0,5468 |
| 16840210 | solute carrier family 52, riboflavin transporter, member 1 | SLC52A1 | -0,22 | 0,0370 | -0,08 | 0,3385 |
| 17000608 | spermatogenesis associated 24 | SPATA24 | -0,22 | 0,0095 | -0,23 | 0,1616 |
| 17084321 | ankyrin repeat domain 18B | ANKRD18B | -0,21 | 0,0153 | 0,07 | 0,6043 |
| 16961172 | butyrylcholinesterase | BCHE | -0,21 | 0,0160 | -0,05 | 0,6916 |
| 16897719 | coiled-coil domain containing 88A | CCDC88A | -0,21 | 0,0185 | -0,23 | 0,0557 |
| 16745016 | CD3d molecule, delta (CD3-TCR complex) | CD3D | -0,21 | 0,0365 | -0,24 | 0,1691 |
| 16945497 | collagen, type VI, alpha 5 | COL6A5 | -0,21 | 0,0476 | -0,11 | 0,5968 |
| 16658726 | catenin, beta interacting protein 1 | CTNNBIP1 | -0,21 | 0,0268 | -0,12 | 0,3129 |
| 17074331 | defensin, alpha 3, neutrophil-specific \| defensin, alpha 1B \| defensin, alpha 1 | DEFA3\| DEFA1B | -0,21 | 0,0196 | -0,05 | 0,4731 |
| 16795285 | deiodinase, iodothyronine, type II | DIO2 | -0,21 | 0,0202 | -0,17 | 0,0821 |
| 16812779 | golgin A6 family-like 5 (pseudogene) | GOLGA6L5 | -0,21 | 0,0265 | 0,13 | 0,2607 |
| 17027187 | major histocompatibility complex, class II, DP alpha 1 | HLA-DPA1 | -0,21 | 0,0156 | -0,17 | 0,2849 |
| 16675539 | LIM homeobox 9 | LHX9 | -0,21 | 0,0368 | -0,22 | 0,2461 |
| 16987874 | phosphoglycerate mutase family member 5 pseudogene | LOC100289673 | -0,21 | 0,0397 | 0,03 | 0,5721 |
| 16753914 | lysozyme | LYZ | -0,21 | 0,0249 | -0,21 | 0,2582 |
| 16936624 | outer dense fiber of sperm tails 3B | ODF3B | -0,21 | 0,0103 | 0,00 | 0,9786 |
| 17096726 | olfactory receptor, family 13, subfamily C, member 9 | OR13C9 | -0,21 | 0,0493 | -0,02 | 0,7677 |
| 16947107 | purinergic receptor P2Y, G-protein coupled, 1 | P2RY1 | -0,21 | 0,0314 | -0,22 | 0,0978 |
| 16959752 | proline rich 23C | PRR23C | -0,21 | 0,0077 | -0,04 | 0,7474 |
| 16927581 | RIMS binding protein 3B | RIMBP3B | -0,21 | 0,0298 | -0,10 | 0,5144 |
| 17075198 | solute carrier family 18 (vesicular monoamine), member 1 | SLC18A1 | -0,21 | 0,0070 | -0,09 | 0,4506 |
| 16891373 | acid-sensing (proton-gated) ion channel family member 4 | ASIC4 | -0,2 | 0,0096 | -0,11 | 0,3017 |
| 17005998 | chromosome 6 open reading frame 100 | C6orf100 | -0,2 | 0,0215 | -0,06 | 0,5379 |
| 16955172 | CACNA2D3 antisense RNA 1 (non-protein coding) | CACNA2D3-AS1 | -0,2 | 0,0212 | -0,16 | 0,4409 |
| 16776100 | citrate lyase beta like | CLYBL | -0,2 | 0,0103 | -0,27 | 0,1530 |
| 17021489 | cannabinoid receptor 1 (brain) | CNR1 | -0,2 | 0,0432 | -0,04 | 0,7773 |
| 17048473 | collagen, type I, alpha 2 | COL1A2 | -0,2 | 0,0428 | -0,19 | 0,0976 |
| 16791427 | cathepsin G | CTSG | -0,2 | 0,0045 | -0,29 | 0,2732 |
| 16688799 | EGF, latrophilin and seven transmembrane domain containing 1 | ELTD1 | -0,2 | 0,0061 | -0,12 | 0,2195 |
| 17053872 | 5-hydroxytryptamine (serotonin) receptor 5A, G protein-coupled | HTR5A | -0,2 | 0,0476 | -0,08 | 0,5378 |
| 16844430 | keratin 25 | KRT25 | -0,2 | 0,0070 | -0,19 | 0,2486 |
| 16844610 | keratin associated protein 4-6 | KRTAP4-6 | -0,2 | 0,0027 | -0,19 | 0,0769 |
| 16693331 | late cornified envelope 3E | LCE3E | -0,2 | 0,0389 | -0,15 | 0,2903 |
| 16828270 | nuclear pore complex-interacting protein-like 2-like | LOC100507607\| LOC728734 | -0,2 | 0,0316 | -0,11 | 0,3093 |
| 17030610 | lymphotoxin alpha (TNF superfamily, member 1) | LTA | -0,2 | 0,0070 | -0,18 | 0,0586 |
| 17038107 | lymphotoxin alpha (TNF superfamily, member 1) | LTA | -0,2 | 0,0088 | -0,19 | 0,0587 |
| 16743707 | matrix metallopeptidase 10 (stromelysin 2) | MMP10 | -0,2 | 0,0481 | -0,14 | 0,2390 |
| 16734616 | MAS-related GPR, member E | MRGPRE | -0,2 | 0,0099 | 0,02 | 0,9427 |
| 16732386 |  | OAF | -0,2 | 0,0152 | -0,14 | 0,1937 |
| 16682771 | phospholipase A2, group IIA (platelets, synovial fluid) | PLA2G2A | -0,2 | 0,0221 | -0,04 | 0,7269 |
| 16855564 | retina and anterior neural fold homeobox | RAX | -0,2 | 0,0136 | -0,07 | 0,6306 |
| 16863367 | SIX homeobox 5 | SIX5 | -0,2 | 0,0364 | -0,33 | 0,0908 |
| 17023697 | trace amine associated receptor 3 (gene/pseudogene) | TAAR3 | -0,2 | 0,0475 | -0,11 | 0,5156 |
| 16766352 | tachykinin 3 | TAC3 | -0,2 | 0,0073 | -0,06 | 0,2010 |
| 17104450 | arrestin 3, retinal (X-arrestin) | ARR3 | -0,19 | 0,0227 | 0,03 | 0,4861 |
| 16814297 | chromosome 16 open reading frame 11 | C16orf11 | -0,19 | 0,0104 | -0,17 | 0,0646 |
| 16695437 |  | CD48 | -0,19 | 0,0398 | -0,08 | 0,4157 |
| 17092526 | cerberus 1, cysteine knot superfamily, homolog (Xenopus laevis) | CER1 | -0,19 | 0,0243 | 0,01 | 0,9757 |
| 16921064 | GATA binding protein 5 | GATA5 | -0,19 | 0,0341 | -0,17 | 0,1093 |
| 16669285 | ganglioside induced differentiation associated protein 2 | GDAP2 | -0,19 | 0,0255 | -0,02 | 0,8681 |
| 17055824 | KLHL7 antisense RNA 1 (non-protein coding) | KLHL7-AS1 | -0,19 | 0,0322 | -0,04 | 0,6047 |
| 17047088 | linker for activation of T cells family, member 2 | LAT2 | -0,19 | 0,0024 | -0,14 | 0,0908 |
| 17010292 | OOEP antisense RNA 1 (non-protein coding) | OOEP-AS1 | -0,19 | 0,0120 | -0,22 | 0,1416 |
| 17103597 | protein phosphatase 1, regulatory subunit 3F | PPP1R3F | -0,19 | 0,0004 | -0,17 | 0,0775 |
| 17028297 | ring finger protein 5, E3 ubiquitin protein ligase | RNF5 | -0,19 | 0,0046 | -0,08 | 0,4666 |
| 16952280 | sodium channel, voltage-gated, type V, alpha subunit | SCN5A | -0,19 | 0,0263 | 0,00 | 0,9723 |
| 17023298 | six-twelve leukemia | STL | -0,19 | 0,0257 | -0,15 | 0,0847 |
| 17036406 | tigger transposable element derived 1-like | TIGD1L | -0,19 | 0,0215 | -0,08 | 0,7166 |
| 17063924 | transmembrane protein 139 | TMEM139 | -0,19 | 0,0059 | -0,27 | 0,0768 |
| 17117383 | testis-specific transcript, Y-linked 4 (non-protein coding) \| testis-specific transcript, Y-linked 4B (non-protein coding) \| testis-specific transcript, Y-linked 4C (non-protein coding) | TTTY4\| TTTY4B\| TTTY4C | -0,19 | 0,0254 | -0,28 | 0,0619 |
| 16958667 | urocanase domain containing 1 | UROC1 | -0,19 | 0,0009 | -0,04 | 0,6542 |
| 16821794 | zinc finger protein, multitype 1 | ZFPM1 | -0,19 | 0,0263 | -0,15 | 0,2232 |
| 16697471 | UDP-Gal:betaGlcNAc beta 1,3-galactosyltransferase, polypeptide 2 | B3GALT2 | -0,18 | 0,0328 | 0,05 | 0,5725 |
| 16670479 | chromosome 1 open reading frame 51 | C1orf51 | -0,18 | 0,0242 | -0,01 | 0,9295 |
| 17054631 | caspase recruitment domain family, member 11 | CARD11 | -0,18 | 0,0353 | -0,27 | 0,0937 |
| 17075731 | clusterin | CLU | -0,18 | 0,0024 | -0,15 | 0,1532 |
| 16921793 |  | FLJ42200 | -0,18 | 0,0271 | -0,08 | 0,1894 |
| 16713982 | FERM and PDZ domain containing 2 | FRMPD2 | -0,18 | 0,0184 | -0,03 | 0,5306 |
| 16875448 | leukocyte immunoglobulin-like receptor, subfamily A (with TM domain), member 5 | LILRA5 | -0,18 | 0,0110 | -0,10 | 0,2847 |
| 17117657 | AGVR6190 | LOC643797 | -0,18 | 0,0445 | -0,15 | 0,2655 |
| 16803006 | peptidylprolyl isomerase A (cyclophilin A) pseudogene | LOC729739 | -0,18 | 0,0110 | 0,13 | 0,2728 |
| 16745470 | mir-100-let-7a-2 cluster host gene (non-protein coding) | MIR100HG | -0,18 | 0,0347 | -0,13 | 0,1687 |
| 17099742 | mitochondrial ribosomal protein S2 | MRPS2 | -0,18 | 0,0440 | -0,21 | 0,0877 |
| 16738819 | membrane-spanning 4-domains, subfamily A, member 6A | MS4A6A | -0,18 | 0,0140 | 0,00 | 0,9771 |
| 16819257 | metallothionein 1H | MT1H | -0,18 | 0,0105 | -0,06 | 0,2865 |
| 16819224 | metallothionein 1M | MT1M | -0,18 | 0,0424 | -0,22 | 0,4131 |
| 16835876 | MYCBP associated protein | MYCBPAP | -0,18 | 0,0206 | -0,13 | 0,0880 |
| 17075542 | NK2 homeobox 6 | NKX2-6 | -0,18 | 0,0117 | -0,04 | 0,7955 |
| 16710540 | neuropeptide S | NPS | -0,18 | 0,0173 | 0,01 | 0,9323 |
| 16872990 | pleckstrin homology-like domain, family B, member 3 | PHLDB3 | -0,18 | 0,0263 | -0,03 | 0,8520 |
| 16761982 | RERG/RAS-like | RERGL | -0,18 | 0,0099 | -0,08 | 0,2301 |
| 16802886 | TBC1 domain family, member 21 | TBC1D21 | -0,18 | 0,0278 | -0,03 | 0,3733 |
| 16674106 | tenascin N | TNN | -0,18 | 0,0074 | -0,08 | 0,2195 |
| 16856182 | tubulin polyglutamylase complex subunit 1 | TPGS1 | -0,18 | 0,0216 | -0,14 | 0,4750 |
| 16875904 | zinc finger protein 835 | ZNF835 | -0,18 | 0,0070 | -0,19 | 0,2546 |
| 16725372 | zona pellucida glycoprotein 1 (sperm receptor) | ZP1 | -0,18 | 0,0061 | -0,26 | 0,1325 |
| 17050746 | ankyrin repeat domain 7 | ANKRD7 | -0,17 | 0,0406 | -0,06 | 0,4272 |
| 16668229 | ataxin 7-like 2 | ATXN7L2 | -0,17 | 0,0187 | 0,03 | 0,2110 |
| 17090640 | BarH-like homeobox 1 | BARHL1 | -0,17 | 0,0156 | -0,17 | 0,1862 |
| 16740317 | chromosome 11 open reading frame 2 | C11orf2 | -0,17 | 0,0069 | -0,23 | 0,1528 |
| 16837055 | chromosome 17 open reading frame 72 | C17orf72 | -0,17 | 0,0481 | -0,18 | 0,0821 |
| 16661102 | cation channel, sperm associated 4 | CATSPER4 | -0,17 | 0,0205 | -0,09 | 0,1006 |
| 17039228 | coiled-coil alpha-helical rod protein 1 | CCHCR1 | -0,17 | 0,0142 | -0,22 | 0,1073 |
| 16693810 | cholinergic receptor, nicotinic, beta 2 (neuronal) | CHRNB2 | -0,17 | 0,0358 | -0,19 | 0,2136 |
| 16741000 | coronin, actin binding protein, 1B | CORO1B | -0,17 | 0,0033 | -0,08 | 0,4211 |
| 16780838 | DAOA antisense RNA 1 (non-protein coding) | DAOA-AS1 | -0,17 | 0,0308 | -0,03 | 0,8760 |
| 16965041 | defensin, beta 131 | DEFB131 | -0,17 | 0,0447 | 0,13 | 0,6850 |
| 17008056 | FYVE, RhoGEF and PH domain containing 2 | FGD2 | -0,17 | 0,0238 | -0,17 | 0,2271 |
| 16825647 | glycerophosphodiester phosphodiesterase domain containing 3 | GDPD3 | -0,17 | 0,0086 | -0,06 | 0,1232 |
| 16991210 | GM2 ganglioside activator | GM2A | -0,17 | 0,0422 | -0,20 | 0,1723 |
| 17006546 | HLA complex group 22 (non-protein coding) | HCG22 | -0,17 | 0,0277 | -0,17 | 0,1413 |
| 17069976 | potassium voltage-gated channel, Shab-related subfamily, member 2 | KCNB2 | -0,17 | 0,0130 | -0,09 | 0,3889 |
| 16948569 | KLHL6 antisense RNA 1 (non-protein coding) | KLHL6-AS1 | -0,17 | 0,0396 | 0,04 | 0,7826 |
| 16990852 | hCG1980447 | LOC255187 | -0,17 | 0,0186 | 0,06 | 0,5144 |
| 17027784 | lymphotoxin alpha (TNF superfamily, member 1) | LTA | -0,17 | 0,0341 | -0,15 | 0,2183 |
| 16752061 | NCK-associated protein 1-like | NCKAP1L | -0,17 | 0,0054 | -0,18 | 0,1224 |
| 17028564 | olfactory receptor, family 2, subfamily W, member 1 | OR2W1 | -0,17 | 0,0366 | -0,16 | 0,2316 |
| 17014543 | PARK2 co-regulated | PACRG | -0,17 | 0,0040 | -0,17 | 0,4276 |
| 16761997 | phospholipase C, zeta 1 | PLCZ1 | -0,17 | 0,0471 | -0,15 | 0,1588 |
| 16842774 | protein interacting with cyclin A1 | PROCA1 | -0,17 | 0,0280 | 0,00 | 0,9295 |
| 16681611 | spermidine synthase | SRM | -0,17 | 0,0226 | -0,08 | 0,2637 |
| 16943892 | transmembrane protease, serine 7 | TMPRSS7 | -0,17 | 0,0477 | -0,08 | 0,6313 |
| 17008461 | triggering receptor expressed on myeloid cells-like 5, pseudogene | TREML5P | -0,17 | 0,0088 | -0,06 | 0,8546 |
| 17098066 | TTLL11 intronic transcript 1 (non-protein coding) | TTLL11-IT1 | -0,17 | 0,0289 | 0,00 | 0,9877 |
| 16834441 | vacuolar protein sorting 25 homolog (S. cerevisiae) | VPS25 | -0,17 | 0,0420 | -0,08 | 0,1189 |
| 16873811 | binder of sperm protein homolog 1 | BSPH1 | -0,16 | 0,0409 | -0,18 | 0,0888 |
| 16676547 | cathepsin E | CTSE | -0,16 | 0,0157 | -0,13 | 0,4230 |
| 16808046 | erythrocyte membrane protein band 4.2 | EPB42 | -0,16 | 0,0402 | -0,01 | 0,7967 |
| 16830214 | F-box protein 39 | FBXO39 | -0,16 | 0,0254 | -0,10 | 0,2519 |
| 16984801 | granzyme A (granzyme 1, cytotoxic T-lymphocyte-associated serine esterase 3) | GZMA | -0,16 | 0,0364 | -0,09 | 0,5442 |
| 16872491 | inositol-trisphosphate 3-kinase C | ITPKC | -0,16 | 0,0172 | -0,13 | 0,1600 |
| 16725240 | membrane-spanning 4-domains, subfamily A, member 5 | MS4A5 | -0,16 | 0,0229 | -0,20 | 0,0612 |
| 17026505 | olfactory receptor, family 2, subfamily W, member 1 | OR2W1 | -0,16 | 0,0467 | -0,14 | 0,2766 |
| 17036093 | olfactory receptor, family 2, subfamily W, member 1 | OR2W1 | -0,16 | 0,0500 | -0,14 | 0,2805 |
| 16894735 | RAD51 associated protein 2 | RAD51AP2 | -0,16 | 0,0235 | 0,02 | 0,7690 |
| 16850366 | saitohin | STH | -0,16 | 0,0489 | -0,03 | 0,8276 |
| 16769840 | SV2 related protein homolog (rat) | SVOP | -0,16 | 0,0018 | -0,15 | 0,4556 |
| 16910785 | transglutaminase 6 | TGM6 | -0,16 | 0,0228 | -0,17 | 0,2639 |
| 17063873 | transient receptor potential cation channel, subfamily V, member 5 | TRPV5 | -0,16 | 0,0044 | 0,02 | 0,8563 |
| 17018980 | unc-5 homolog C (C. elegans)-like \| chromosome 6 open reading frame 130 | UNC5CL\| C6orf130 | -0,16 | 0,0225 | -0,16 | 0,3036 |
| 16932988 | zinc finger, DHHC-type containing 8 pseudogene 1 | ZDHHC8P1 | -0,16 | 0,0085 | -0,21 | 0,0599 |
| 16674348 | v-abl Abelson murine leukemia viral oncogene homolog 2 | ABL2 | -0,15 | 0,0120 | -0,14 | 0,1026 |
| 17073044 | chromosome 8 open reading frame 17 | C8orf17 | -0,15 | 0,0322 | 0,01 | 0,8846 |
| 16893260 | calpain 10 | CAPN10 | -0,15 | 0,0304 | -0,11 | 0,0936 |
| 16902847 | cripto, FRL-1, cryptic family 1 \| cripto, FRL-1, cryptic family 1B | CFC1\| CFC1B | -0,15 | 0,0471 | 0,01 | 0,9162 |
| 16763104 | copine VIII | CPNE8 | -0,15 | 0,0407 | -0,12 | 0,3280 |
| 16887228 | glucose-6-phosphatase, catalytic, 2 | G6PC2 | -0,15 | 0,0267 | -0,02 | 0,8713 |
| 16742244 | glycerophosphodiester phosphodiesterase domain containing 5 | GDPD5 | -0,15 | 0,0369 | -0,09 | 0,5625 |
| 16734858 | hemoglobin, beta pseudogene 1 | HBBP1 | -0,15 | 0,0234 | 0,21 | 0,3116 |
| 16720842 | IGF2 antisense RNA (non-protein coding) \| IGF2 antisense RNA 1 (non-protein coding) | IGF2-AS\| IGF2-AS1 | -0,15 | 0,0127 | -0,02 | 0,3490 |
| 16927749 | immunoglobulin lambda variable 1-44 | IGLV1-44 | -0,15 | 0,0408 | -0,06 | 0,7297 |
| 16767666 | potassium voltage-gated channel, Shaw-related subfamily, member 2 | KCNC2 | -0,15 | 0,0476 | -0,03 | 0,8671 |
| 17040838 | lymphocyte antigen 6 complex, locus G6F \| lymphocyte antigen 6 complex, locus G6D | LY6G6F\| LY6G6D | -0,15 | 0,0319 | -0,09 | 0,5589 |
| 16979056 | neurogenin 2 | NEUROG2 | -0,15 | 0,0036 | -0,12 | 0,4608 |
| 17019888 | phosphoglycerate kinase 2 | PGK2 | -0,15 | 0,0217 | -0,06 | 0,2463 |
| 16781684 | ribonuclease, RNase A family, 7 | RNASE7 | -0,15 | 0,0360 | -0,09 | 0,1863 |
| 17105939 | ring finger protein 128, E3 ubiquitin protein ligase | RNF128 | -0,15 | 0,0003 | -0,01 | 0,9733 |
| 16693421 | S100 calcium binding protein A7-like 2 | S100A7L2 | -0,15 | 0,0475 | -0,20 | 0,0540 |
| 17107492 | SLIT and NTRK-like family, member 2 | SLITRK2 | -0,15 | 0,0428 | 0,01 | 0,9323 |
| 16767006 | SLIT-ROBO Rho GTPase activating protein 1 | SRGAP1 | -0,15 | 0,0456 | -0,20 | 0,1189 |
| 17116468 | testis-specific transcript, Y-linked 6 (non-protein coding) \| testis-specific transcript, Y-linked 6B (non-protein coding) | TTTY6\| TTTY6B | -0,15 | 0,0453 | -0,11 | 0,2108 |
| 16871857 | zinc finger protein 573 | ZNF573 | -0,15 | 0,0245 | 0,17 | 0,2930 |
| 16699611 | axin interactor, dorsalization associated | AIDA | -0,14 | 0,0019 | -0,03 | 0,6215 |
| 16819469 | ADP-ribosylation factor-like 2 binding protein | ARL2BP | -0,14 | 0,0377 | -0,08 | 0,2084 |
| 17022952 | BET3 like (S. cerevisiae) | BET3L | -0,14 | 0,0312 | -0,13 | 0,4046 |
| 16979317 | chromosome 4 open reading frame 3 | C4orf3 | -0,14 | 0,0023 | -0,07 | 0,1210 |
| 16954567 | cytokine inducible SH2-containing protein | CISH | -0,14 | 0,0098 | -0,27 | 0,2047 |
| 16662427 | claspin | CLSPN | -0,14 | 0,0425 | -0,20 | 0,1851 |
| 16803492 | cellular retinoic acid binding protein 1 | CRABP1 | -0,14 | 0,0076 | -0,02 | 0,9372 |
| 17103210 | CXXC finger protein 1 pseudogene 1 | CXXC1P1 | -0,14 | 0,0100 | -0,01 | 0,9598 |
| 16827099 | FLJ27243 protein \| brain expressed, associated with NEDD4, 1 | FLJ27243\| BEAN1 | -0,14 | 0,0478 | -0,03 | 0,7967 |
| 17020240 | 3-hydroxymethyl-3-methylglutaryl-CoA lyase-like 1 | HMGCLL1 | -0,14 | 0,0448 | 0,08 | 0,6030 |
| 17068319 | indoleamine 2,3-dioxygenase 2 | IDO2 | -0,14 | 0,0241 | 0,07 | 0,3062 |
| 16922759 | potassium inwardly-rectifying channel, subfamily J, member 15 | KCNJ15 | -0,14 | 0,0009 | -0,03 | 0,7682 |
| 17107874 | melanoma antigen family A, 2 \| melanoma antigen family A, 2B | MAGEA2\| MAGEA2B | -0,14 | 0,0379 | -0,03 | 0,7351 |
| 16681595 | mannan-binding lectin serine peptidase 2 | MASP2 | -0,14 | 0,0020 | -0,14 | 0,1982 |
| 16873501 | peptidoglycan recognition protein 1 | PGLYRP1 | -0,14 | 0,0324 | -0,06 | 0,4714 |
| 16995715 | ribosomal protein L37 \| uncharacterized LOC100506548 | RPL37\| LOC100506548 | -0,14 | 0,0398 | 0,00 | 0,9960 |
| 16763060 | synaptotagmin X | SYT10 | -0,14 | 0,0352 | -0,12 | 0,5025 |
| 16673489 | T-box 19 | TBX19 | -0,14 | 0,0175 | 0,02 | 0,7288 |
| 16806796 | transmembrane and coiled-coil domains 5B, pseudogene | TMCO5B | -0,14 | 0,0099 | 0,01 | 0,9503 |
| 16831028 | WD repeat domain 16 | WDR16 | -0,14 | 0,0489 | -0,06 | 0,3627 |
| 17107983 | zinc finger protein 92 homolog (mouse) | ZFP92 | -0,14 | 0,0179 | -0,10 | 0,6094 |
| 16870828 | zinc finger protein 43 | ZNF43 | -0,14 | 0,0045 | -0,11 | 0,4478 |
| 16700195 | chromosome 1 open reading frame 145 | C1orf145 | -0,13 | 0,0192 | -0,09 | 0,5159 |
| 16934157 | chromosome 22 open reading frame 24 | C22orf24 | -0,13 | 0,0367 | -0,22 | 0,1947 |
| 16843541 | chemokine (C-C motif) ligand 16 | CCL16 | -0,13 | 0,0321 | 0,09 | 0,4740 |
| 17093543 | chemokine (C-C motif) ligand 21 | CCL21 | -0,13 | 0,0168 | -0,08 | 0,4156 |
| 17007109 | cytochrome P450, family 21, subfamily A, polypeptide 1 pseudogene | CYP21A1P | -0,13 | 0,0258 | -0,09 | 0,6007 |
| 17117730 | dead end homolog 1 (zebrafish) \| WD repeat domain 55 \| dead end protein homolog 1-like | DND1\| WDR55\| LOC100134173 | -0,13 | 0,0277 | 0,02 | 0,4947 |
| 17080554 | ectonucleotide pyrophosphatase/phosphodiesterase 2 | ENPP2 | -0,13 | 0,0067 | -0,18 | 0,2057 |
| 16723058 | fin bud initiation factor homolog (zebrafish) | FIBIN | -0,13 | 0,0017 | -0,01 | 0,9426 |
| 17031139 | major histocompatibility complex, class II, DR alpha | HLA-DRA | -0,13 | 0,0454 | -0,02 | 0,7824 |
| 16720956 | KCNQ1 downstream neighbor (non-protein coding) | KCNQ1DN | -0,13 | 0,0225 | -0,12 | 0,1438 |
| 16755671 | nuclear receptor subfamily 1, group H, member 4 | NR1H4 | -0,13 | 0,0277 | 0,03 | 0,6596 |
| 17108244 | opsin 1 (cone pigments), medium-wave-sensitive \| opsin 1 (cone pigments), medium-wave-sensitive 2 \| opsin 1 (cone pigments), long-wave-sensitive | OPN1MW\| OPN1MW2\| OPN1LW | -0,13 | 0,0417 | -0,08 | 0,3005 |
| 17034972 | olfactory receptor, family 12, subfamily D, member 2 | OR12D2 | -0,13 | 0,0058 | -0,05 | 0,7375 |
| 16852226 | ring finger protein 165 | RNF165 | -0,13 | 0,0452 | -0,25 | 0,1128 |
| 16999083 | sema domain, transmembrane domain (TM), and cytoplasmic domain, (semaphorin) 6A | SEMA6A | -0,13 | 0,0132 | -0,08 | 0,0592 |
| 17063194 | solute carrier family 13 (sodium/sulfate symporters), member 4 | SLC13A4 | -0,13 | 0,0349 | -0,10 | 0,2584 |
| 17008950 | solute carrier family 22 (organic anion transporter), member 7 | SLC22A7 | -0,13 | 0,0169 | 0,07 | 0,1207 |
| 17000566 | solute carrier family 23 (nucleobase transporters), member 1 | SLC23A1 | -0,13 | 0,0100 | -0,02 | 0,7482 |
| 16699376 | solute carrier family 30, member 10 | SLC30A10 | -0,13 | 0,0323 | 0,00 | 0,9590 |
| 16957323 | solute carrier family 9, subfamily C (Na+-transporting carboxylic acid decarboxylase), member 1 | SLC9C1 | -0,13 | 0,0422 | -0,04 | 0,7429 |
| 17112710 | TAF7-like RNA polymerase II, TATA box binding protein (TBP)-associated factor, 50kDa | TAF7L | -0,13 | 0,0026 | -0,03 | 0,7703 |
| 16743193 | tripartite motif containing 64C \| tripartite motif containing 64B \| tripartite motif containing 64 | TRIM64C\| TRIM64B | -0,13 | 0,0222 | -0,18 | 0,2604 |
| 16662108 | testis-specific serine kinase 3 \| uncharacterized LOC100128071 | TSSK3\| LOC100128071 | -0,13 | 0,0209 | 0,04 | 0,7468 |
| 16994521 | ankylosis, progressive homolog (mouse) | ANKH | -0,12 | 0,0366 | -0,04 | 0,8227 |
| 16940738 | N-acylaminoacyl-peptide hydrolase | APEH | -0,12 | 0,0468 | -0,03 | 0,8160 |
| 16763011 | bicaudal D homolog 1 (Drosophila) | BICD1 | -0,12 | 0,0178 | -0,17 | 0,5350 |
| 16712505 | chromosome 10 open reading frame 115 | C10orf115 | -0,12 | 0,0078 | -0,11 | 0,1321 |
| 17024221 | coiled-coil domain containing 28A \| uncharacterized LOC100507462 | CCDC28A\| LOC100507462 | -0,12 | 0,0194 | -0,15 | 0,1940 |
| 16663288 | coiled-coil domain containing 30 | CCDC30 | -0,12 | 0,0346 | -0,12 | 0,4705 |
| 17052796 | chloride channel, voltage-sensitive 1 | CLCN1 | -0,12 | 0,0032 | -0,28 | 0,1559 |
| 17015331 | coagulation factor XIII, A1 polypeptide | F13A1 | -0,12 | 0,0396 | -0,02 | 0,8005 |
| 16908197 | insulin-like growth factor binding protein 5 | IGFBP5 | -0,12 | 0,0366 | -0,10 | 0,2835 |
| 16742738 | odz, odd Oz/ten-m homolog 4 (Drosophila) | ODZ4 | -0,12 | 0,0003 | -0,03 | 0,5726 |
| 17032787 | olfactory receptor, family 12, subfamily D, member 2 | OR12D2 | -0,12 | 0,0027 | -0,05 | 0,7496 |
| 16805799 | olfactory receptor, family 4, subfamily F, member 13 pseudogene | OR4F13P | -0,12 | 0,0384 | -0,15 | 0,3923 |
| 17116420 | ribosomal protein S4, Y-linked 2 | RPS4Y2 | -0,12 | 0,0461 | -0,03 | 0,8371 |
| 16819109 | solute carrier family 6 (neurotransmitter transporter, noradrenalin), member 2 | SLC6A2 | -0,12 | 0,0232 | -0,09 | 0,2491 |
| 16946331 | splA/ryanodine receptor domain and SOCS box containing 4 | SPSB4 | -0,12 | 0,0297 | -0,19 | 0,0660 |
| 16940687 |  | STGC3 | -0,12 | 0,0473 | 0,01 | 0,7365 |
| 16862804 | testis expressed 101 | TEX101 | -0,12 | 0,0229 | -0,09 | 0,4668 |
| 16902979 | transmembrane protein 163 | TMEM163 | -0,12 | 0,0155 | -0,03 | 0,8378 |
| 17082578 | transmembrane protein 249 | TMEM249 | -0,12 | 0,0303 | -0,04 | 0,5239 |
| 16993311 | zinc finger protein 454 | ZNF454 | -0,12 | 0,0104 | -0,02 | 0,7431 |
| 17110523 | zinc finger protein 630 | ZNF630 | -0,12 | 0,0058 | -0,09 | 0,6652 |
| 16716350 | actin, alpha 2, smooth muscle, aorta | ACTA2 | -0,11 | 0,0005 | -0,09 | 0,2809 |
| 17052083 | aldo-keto reductase family 1, member D1 (delta 4-3-ketosteroid-5-beta-reductase) | AKR1D1 | -0,11 | 0,0269 | 0,00 | 0,9864 |
| 16661031 | exostoses (multiple)-like 1 | EXTL1 | -0,11 | 0,0089 | -0,06 | 0,6659 |
| 16859611 | FCH domain only 1 | FCHO1 | -0,11 | 0,0485 | 0,07 | 0,2697 |
| 16837382 | potassium inwardly-rectifying channel, subfamily J, member 16 | KCNJ16 | -0,11 | 0,0286 | -0,15 | 0,0746 |
| 16844466 | keratin 28 | KRT28 | -0,11 | 0,0062 | -0,09 | 0,0584 |
| 16974212 | hCG2042714-like | LOC100133683 | -0,11 | 0,0352 | -0,04 | 0,2281 |
| 16961331 | MDS1 and EVI1 complex locus | MECOM | -0,11 | 0,0176 | -0,11 | 0,1064 |
| 16866646 | NADH dehydrogenase (ubiquinone) Fe-S protein 7, 20kDa (NADH-coenzyme Q reductase) | NDUFS7 | -0,11 | 0,0228 | -0,07 | 0,2683 |
| 16739911 | neurexin 2 | NRXN2 | -0,11 | 0,0076 | 0,05 | 0,7256 |
| 17027251 | olfactory receptor, family 12, subfamily D, member 2 | OR12D2 | -0,11 | 0,0317 | -0,02 | 0,8923 |
| 16849903 | phosphodiesterase 6G, cGMP-specific, rod, gamma | PDE6G | -0,11 | 0,0088 | -0,06 | 0,5016 |
| 17077277 | SET binding factor 1 pseudogene 1 | SBF1P1 | -0,11 | 0,0050 | -0,12 | 0,1371 |
| 17013954 | T-cell lymphoma invasion and metastasis 2 \| uncharacterized LOC100505519 | TIAM2\| LOC100505519 | -0,11 | 0,0035 | -0,22 | 0,1637 |
| 16891782 | WD repeat domain 69 | WDR69 | -0,11 | 0,0478 | 0,00 | 0,9973 |
| 16939815 | abhydrolase domain containing 5 | ABHD5 | -0,1 | 0,0439 | -0,02 | 0,7435 |
| 16802413 | coronin, actin binding protein, 2B | CORO2B | -0,1 | 0,0184 | -0,11 | 0,4230 |
| 16717970 | cytochrome P450, family 17, subfamily A, polypeptide 1 | CYP17A1 | -0,1 | 0,0036 | -0,03 | 0,7963 |
| 16695504 | F11 receptor | F11R | -0,1 | 0,0253 | 0,13 | 0,3380 |
| 16995461 | glial cell derived neurotrophic factor | GDNF | -0,1 | 0,0452 | 0,00 | 0,9132 |
| 16687467 | heat shock protein family B (small), member 11 | HSPB11 | -0,1 | 0,0225 | -0,02 | 0,8470 |
| 16844693 | keratin 38 | KRT38 | -0,1 | 0,0288 | -0,14 | 0,2632 |
| 16974925 | leucine-rich repeat LGI family, member 2 | LGI2 | -0,1 | 0,0204 | -0,21 | 0,0978 |
| 17030753 | lymphocyte antigen 6 complex, locus G6D | LY6G6D | -0,1 | 0,0109 | -0,08 | 0,5066 |
| 16865957 | MER1 repeat containing imprinted transcript 1 (non-protein coding) | MIMT1 | -0,1 | 0,0350 | -0,01 | 0,9239 |
| 16874153 | NUCB1 antisense RNA 1 (non-protein coding) | NUCB1-AS1 | -0,1 | 0,0077 | -0,02 | 0,5563 |
| 16836409 | olfactory receptor, family 4, subfamily D, member 1 | OR4D1 | -0,1 | 0,0230 | 0,03 | 0,7385 |
| 16923805 | poly(rC) binding protein 3 | PCBP3 | -0,1 | 0,0397 | 0,11 | 0,4465 |
| 16977153 | shroom family member 3 | SHROOM3 | -0,1 | 0,0171 | 0,06 | 0,6858 |
| 16804268 | solute carrier family 28 (sodium-coupled nucleoside transporter), member 1 | SLC28A1 | -0,1 | 0,0394 | -0,07 | 0,3452 |
| 17056625 | T-box 20 | TBX20 | -0,1 | 0,0459 | 0,00 | 0,9559 |
| 16774384 | tumor necrosis factor (ligand) superfamily, member 11 | TNFSF11 | -0,1 | 0,0088 | -0,06 | 0,2413 |
| 16695643 | apolipoprotein A-II | APOA2 | -0,09 | 0,0432 | 0,06 | 0,5669 |
| 16705051 | CDGSH iron sulfur domain 1 | CISD1 | -0,09 | 0,0215 | 0,03 | 0,7128 |
| 16868063 | fibrillin 3 | FBN3 | -0,09 | 0,0454 | -0,09 | 0,4513 |
| 17005542 | hemochromatosis | HFE | -0,09 | 0,0210 | 0,01 | 0,9529 |
| 16874097 | hydroxysteroid (17-beta) dehydrogenase 14 | HSD17B14 | -0,09 | 0,0213 | 0,05 | 0,6454 |
| 16970971 | interleukin 15 | IL15 | -0,09 | 0,0119 | 0,00 | 0,9800 |
| 16870401 | inositol-3-phosphate synthase 1 | ISYNA1 | -0,09 | 0,0325 | -0,18 | 0,2148 |
| 17006794 | lymphocyte antigen 6 complex, locus G6D \| lymphocyte antigen 6 complex, locus G6F | LY6G6D\| LY6G6F | -0,09 | 0,0302 | -0,08 | 0,5048 |
| 17059089 | MAGI2 antisense RNA 3 (non-protein coding) | MAGI2-AS3 | -0,09 | 0,0279 | -0,20 | 0,0846 |
| 16933406 | myocardial infarction associated transcript (non-protein coding) \| uncharacterized LOC100129936 | MIAT\| LOC100129936 | -0,09 | 0,0274 | 0,05 | 0,7608 |
| 16725890 | retinal outer segment membrane protein 1 | ROM1 | -0,09 | 0,0132 | 0,02 | 0,8440 |
| 16879601 | solute carrier family 3 (cystine, dibasic and neutral amino acid transporters, activator of cystine, dibasic and neutral amino acid transport), member 1 | SLC3A1 | -0,09 | 0,0102 | -0,08 | 0,4025 |
| 16679860 | WAS protein family homolog 7 pseudogene \| WAS protein family homolog 1 \| WAS protein family homolog 2 pseudogene \| WAS protein family homolog 3 pseudogene \| WAS protein family homolog 5 pseudogene \| WAS protein family homolog 1 pseudogene \| WAS protein family homolog 4 pseudogene \| WAS protein family homolog 6 pseudogene | WASH7P\| WASH1\| WASH2P\| WASH3P\| WASH5P\| LOC100288778\| WASH4P\| WASH6P | -0,09 | 0,0421 | -0,12 | 0,3310 |
| 16927710 | pre-B lymphocyte 1 | VPREB1 | -0,09 | 0,0173 | -0,01 | 0,9191 |
| 16791810 | ARHGAP5 antisense RNA 1 (non-protein coding) | ARHGAP5-AS1 | -0,08 | 0,0236 | -0,25 | 0,2394 |
| 16823522 |  | CORO7-PAM16 | -0,08 | 0,0089 | -0,16 | 0,0907 |
| 17022553 | D-aspartate oxidase | DDO | -0,08 | 0,0477 | 0,09 | 0,5984 |
| 16838988 | G protein pathway suppressor 1 | GPS1 | -0,08 | 0,0408 | -0,03 | 0,7050 |
| 16762413 | intermediate filament tail domain containing 1 | IFLTD1 | -0,08 | 0,0179 | -0,12 | 0,1246 |
| 16887420 | myosin IIIB | MYO3B | -0,08 | 0,0048 | 0,08 | 0,3182 |
| 16724736 | olfactory receptor, family 4, subfamily C, member 6 | OR4C6 | -0,08 | 0,0475 | -0,03 | 0,5853 |
| 16775263 | PCDH9 antisense RNA 2 (non-protein coding) | PCDH9-AS2 | -0,08 | 0,0433 | 0,02 | 0,8188 |
| 17115940 | ribosomal protein S4, Y-linked 1 | RPS4Y1 | -0,08 | 0,0289 | 0,12 | 0,2358 |
| 16975234 | transmembrane protein 156 | TMEM156 | -0,08 | 0,0282 | -0,04 | 0,3103 |
| 16735152 | tripeptidyl peptidase I | TPP1 | -0,08 | 0,0053 | -0,05 | 0,7577 |
| 17058445 | tripartite motif containing 50 | TRIM50 | -0,08 | 0,0064 | 0,10 | 0,2886 |
| 16938228 | potassium voltage-gated channel, subfamily H (eag-related), member 8 | KCNH8 | -0,07 | 0,0422 | 0,02 | 0,6904 |
| 16844663 | keratin 34 \| keratin, type I cuticular Ha4-like | KRT34\| LOC100653049 | -0,07 | 0,0480 | -0,14 | 0,1815 |
| 16911382 | lysosomal-associated membrane protein family, member 5 | LAMP5 | -0,07 | 0,0124 | -0,05 | 0,6655 |
| 16857177 | MPN domain containing | MPND | -0,07 | 0,0264 | 0,05 | 0,6532 |
| 16715079 | neurogenin 3 | NEUROG3 | -0,07 | 0,0241 | -0,08 | 0,6184 |
| 16724777 | olfactory receptor, family 5, subfamily T, member 3 | OR5T3 | -0,07 | 0,0373 | -0,02 | 0,6862 |
| 16867334 | perilipin 5 | PLIN5 | -0,07 | 0,0101 | -0,06 | 0,1835 |
| 16850625 | TGFB-induced factor homeobox 1 | TGIF1 | -0,07 | 0,0489 | -0,09 | 0,4171 |
| 16980716 | transmembrane protein 154 | TMEM154 | -0,07 | 0,0036 | 0,04 | 0,4649 |
| 16944339 | B4GALT4 antisense RNA 1 (non-protein coding) | B4GALT4-AS1 | -0,06 | 0,0488 | 0,00 | 0,9613 |
| 16881485 | chaperonin containing TCP1, subunit 7 (eta) | CCT7 | -0,06 | 0,0057 | -0,01 | 0,8867 |
| 16912304 | defensin, beta 123 | DEFB123 | -0,06 | 0,0110 | 0,05 | 0,6930 |
| 16873247 | exocyst complex component 3-like 2 | EXOC3L2 | -0,06 | 0,0133 | 0,15 | 0,1640 |
| 16997383 | coagulation factor II (thrombin) receptor-like 2 | F2RL2 | -0,06 | 0,0368 | 0,07 | 0,2189 |
| 16856980 | high mobility group 20B | HMG20B | -0,06 | 0,0082 | 0,09 | 0,1762 |
| 16844533 | keratin 40 | KRT40 | -0,06 | 0,0068 | -0,10 | 0,3607 |
| 16844626 | keratin associated protein 4-1 \| keratin associated protein 4-2 | KRTAP4-1\| KRTAP4-2 | -0,06 | 0,0358 | 0,02 | 0,8031 |
| 17043633 | period homolog 3 (Drosophila) pseudogene | PER4 | -0,06 | 0,0251 | -0,09 | 0,3096 |
| 16691090 | protein tyrosine phosphatase, non-receptor type 22 (lymphoid) | PTPN22 | -0,06 | 0,0125 | 0,02 | 0,7433 |
| 16709713 | solute carrier family 18 (vesicular monoamine), member 2 | SLC18A2 | -0,06 | 0,0278 | 0,02 | 0,7433 |
| 16695205 | transgelin 2 | TAGLN2 | -0,06 | 0,0427 | -0,05 | 0,1573 |
| 17008928 | tau tubulin kinase 1 | TTBK1 | -0,06 | 0,0447 | -0,12 | 0,1146 |
| 16868995 | zinc finger protein 653 | ZNF653 | -0,06 | 0,0143 | -0,23 | 0,3066 |
| 16907743 | chromosome 2 open reading frame 80 | C2orf80 | -0,05 | 0,0413 | -0,10 | 0,2396 |
| 17073739 | HEAT repeat containing 7A \| HEAT repeat-containing protein 7A-like | HEATR7A\| LOC100652949\| LOC377711 | -0,05 | 0,0192 | -0,16 | 0,3710 |
| 16678361 | obscurin, cytoskeletal calmodulin and titin-interacting RhoGEF | OBSCN | -0,05 | 0,0281 | -0,12 | 0,1335 |
| 16701634 | olfactory receptor, family 14, subfamily I, member 1 | OR14I1 | -0,05 | 0,0087 | -0,03 | 0,5646 |
| 17058605 | Williams Beuren syndrome chromosome region 27 | WBSCR27 | -0,05 | 0,0256 | 0,08 | 0,3483 |
| 16889923 | INO80 complex subunit D | INO80D | -0,04 | 0,0442 | -0,02 | 0,7747 |
| 16885736 | sphingomyelin phosphodiesterase 4, neutral membrane (neutral sphingomyelinase-3) pseudogene | LOC150776 | -0,04 | 0,0286 | -0,02 | 0,8605 |
| 16940550 | PRKAR2A antisense RNA 1 (non-protein coding) | PRKAR2A-AS1 | -0,04 | 0,0391 | -0,04 | 0,1064 |
| 16802711 | HEXA antisense RNA 1 (non-protein coding) | HEXA-AS1 | -0,03 | 0,0250 | 0,10 | 0,2999 |
| 16815791 | activating transcription factor 7 interacting protein 2 \| uncharacterized LOC100287628 | ATF7IP2\| LOC100287628 | -0,02 | 0,0135 | 0,02 | 0,5505 |
| 16918609 | transient receptor potential cation channel, subfamily C, member 4 associated protein | TRPC4AP | -0,02 | 0,0448 | -0,07 | 0,6218 |
| 17032462 | proteasome (prosome, macropain) subunit, beta type, 8 (large multifunctional peptidase 7) | PSMB8 | -0,01 | 0,0196 | -0,10 | 0,2201 |

^1^From Student’s t-test.

**Table D. Pathways downregulated in response to S6K2 siRNA, but not to S6K1 siRNA.**

| p-value | Term | Term ID | Term description | Genes |
| --- | --- | --- | --- | --- |
| 3.24e-04 | GO:0015844 | BP | monoamine transport | OXT, ADRA2A, CNR1, P2RY1, SLC18A1, CHRNB2, SLC6A2, GDNF, SLC18A2 |
| 3.07e-04 | GO:0044707 | BP | single-multicellular organism process | OR52A5, STC2, RTL1, ITGA8, KCNJ2, OCA2, OXT, ADRA2A, CNR1, PDCD1, CDX2, WNT9B, ATP6V1B1, KLF2, SMAD9, TCF21, HTR3A, OLIG1, SLC6A5, SMTNL1, FGF7, TLL1, HRH1, KNG1, PLXDC1, RPL39L, SGK1, SPIC, TAS2R16, CD40LG, FOXN4, PAFAH1B3, PAX3, PDE2A, SLC26A8, SPATA24, BCHE, CCDC88A, CD3D, CTNNBIP1, LHX9, P2RY1, SLC18A1, COL1A2, HTR5A, KRT25, LCE3E, MMP10, PLA2G2A, RAX, SIX5, TAC3, ARR3, CD48, CER1, SCN5A, ZFPM1, CARD11, CLU, MYCBPAP, NKX2-6, NPS, TNN, TPGS1, ANKRD7, BARHL1, CATSPER4, CHRNB2, GM2A, KCNB2, NCKAP1L, PACRG, PLCZ1, EPB42, ABL2, GDPD5, KCNC2, NEUROG2, RNF128, SLITRK2, SRGAP1, AIDA, CRABP1, KCNJ15, PGLYRP1, TBX19, CCL21, NR1H4, SEMA6A, SLC23A1, SLC9C1, TAF7L, ANKH, CLCN1, F13A1, IGFBP5, ODZ4, SLC6A2, ACTA2, AKR1D1, EXTL1, KCNJ16, MECOM, NRXN2, PDE6G, CYP17A1, F11R, GDNF, OR4D1, SHROOM3, TBX20, TNFSF11, APOA2, HFE, IL15, ROM1, DDO, MYO3B, RPS4Y1, TPP1, KCNH8, NEUROG3, TGIF1, F2RL2, HMG20B, PTPN22, SLC18A2, TAGLN2, OBSCN |
| 3.65e-03 | GO:0006811 | BP | ion transport | HTR3C, KCNJ2, OCA2, OXT, ADRA2A, CNR1, ATP6V1B1, HTR3A, SGK1, SLCO1A2, PDE2A, SCN11A, SLC26A8, SLC52A1, P2RY1, SLC18A1, ASIC4, SCN5A, CATSPER4, CHRNB2, KCNB2, PLCZ1, KCNJ15, CCL21, SLC13A4, SLC22A7, SLC23A1, SLC30A10, SLC9C1, ANKH, KCNJ16, GDNF, TNFSF11, HFE, SLC3A1 |
| 1.51e-02 | GO:0060359 | BP | response to ammonium ion | OXT, CNR1, HTR3A, HRH1, TAC3, CHRNB2, SLC18A2 |
| 1.07e-02 | GO:0015077 | MF | monovalent inorganic cation transmembrane transporter activity | KCNJ2, ATP6V1B1, HTR3A, SLC6A5, SCN11A, ASIC4, SCN5A, KCNB2, KCNC2, KCNJ15, SLC13A4, SLC23A1, SLC9C1, SLC6A2, KCNJ16, SLC28A1, KCNH8 |
| 1.44e-04 | GO:0015075 | MF | ion transmembrane transporter activity | HTR3C, TPTE, KCNJ2, OCA2, ATP6V1B1, HTR3A, SLC6A5, SLCO1A2, PDE2A, SCN11A, SLC26A8, SLC18A1, ASIC4, SCN5A, CATSPER4, CHRNB2, KCNB2, SVOP, KCNC2, KCNJ15, SLC13A4, SLC22A7, SLC23A1, SLC30A10, SLC9C1, ANKH, CLCN1, SLC6A2, KCNJ16, SLC28A1, SLC3A1, KCNH8, SLC18A2 |
| 2.80e-09 | BIOGRID:00000 | bi | BioGRID interaction data | RPS6KB2, DDN, STC2, TPTE, ATXN3L, OS9, ITGA8, KCNJ2, TSLP, OXT, WISP2, ADRA2A, SSTR4, CNR1, PDCD1, CD163 |
| 2.49e-02 | REAC:373076 | re | Class A/1 (Rhodopsin-like receptors) | OXT, ADRA2A, SSTR4, HRH1, KNG1, HTR5A, TAAR3, TAC3, CCL16, CCL21, F2RL2 |
